# Inhibitory Potential of New Phenolic Hydrazide-Hydrazones with a Decoy Substrate Fragment towards Laccase from a Phytopathogenic Fungus: SAR and Molecular Docking Studies

**DOI:** 10.3390/ijms222212307

**Published:** 2021-11-14

**Authors:** Halina Maniak, Michał Talma, Mirosław Giurg

**Affiliations:** 1 Department of Micro, Nano and Bioprocess Engineering, Faculty of Chemistry, Wroclaw University of Science and Technology, Norwida 4/6, 50-373 Wrocław, Poland; 2 Department of Bioorganic Chemistry, Faculty of Chemistry, Wroclaw University of Science and Technology, Wybrzeże Wyspiańskiego 27, 50-370 Wrocław, Poland; michal.talma@pwr.edu.pl; 3 Department of Organic and Medicinal Chemistry, Faculty of Chemistry, Wroclaw University of Science and Technology, Wybrzeże Wyspiańskiego 27, 50-370 Wrocław, Poland

**Keywords:** fungal oxidoreductase, electron-rich arene derivatives, enzyme kinetics, molecular modeling, structure-activity relationship

## Abstract

Laccase from pathogenic fungi participates in both the delignification and neutralization of phytoantibiotics. Furthermore, it interferes with the hormone signaling in plants and catalyzes melanization. Infections of these pathogens contribute to loss in forestry, agriculture, and horticulture. As there is still a need to expand knowledge on efficient defense strategies against phytopathogenic fungi, the present study aimed to reveal more information on the molecular mechanisms of laccase inhibition with natural and natural-like carboxylic acid semi-synthetic derivatives. A set of hydrazide-hydrazones derived from carboxylic acids, generally including electron-rich arene units that serve as a decoy substrate, was synthesized and tested with laccase from *Trametes versicolor*. The classic synthesis of the title inhibitors proceeded with good to almost quantitative yield. Ninety percent of the tested molecules were active in the range of *K*_I_ = 8–233 µM and showed different types of action. Such magnitude of inhibition constants qualified the hydrazide-hydrazones as strong laccase inhibitors. Molecular docking studies supporting the experimental data explained the selected derivatives’ interactions with the enzyme. The results are promising in developing new potential antifungal agents mitigating the damage scale in the plant cultivation, gardening, and horticulture sectors.

## 1. Introduction

Laccase (benzenediol: oxygen oxidoreductase, EC 1.10.3.2) is a blue multi-copper oxidase specific to a broad group of electron-rich arenes such as anilines and phenols. It has the exceptional ability to one electron oxidize both low- and high-molecular-weight substrates via direct or mediated reactions applied in green chemistry [1]. This nonspecific property has been reflected in numerous industrial, medical, and environmental applications [2,3,4,5]. Prokaryotes [6] and eukaryotes such as insects [7], plants, and fungi [8] produce laccase. Among the fungi, *Ascomycota* and *Basidiomycota* are well-known producers of this enzyme. Laccase participates in fungal metabolic pathways such as developing fruit bodies, the pigmentation of spores, and sexual differentiation [9]. It is also known as the virulence factor involved in the pathogenicity of humans [10,11,12] and plants [13,14,15,16]. Among the most troublesome filamentous fungi that produce laccase are *Botrytis cinerea* [17,18], *Sclerotinia sclerotiorum* [19], and *Rhizoctonia* sp. [20,21]. Furthermore, fungi parasitizing on trees use this enzyme in the degradation of lignin. Fungi release laccase into the plant tissues. It catalyzes lignin decomposition and neutralization of the auxins and phytoalexins, a primary barrier against pathogen attack. Such an infection strategy contributes to white-root of wood, crop, and ornamental plants diseases resulting in an enormous ecological and economic loss in forestry and agriculture [22,23]. 

**Figure 1:**
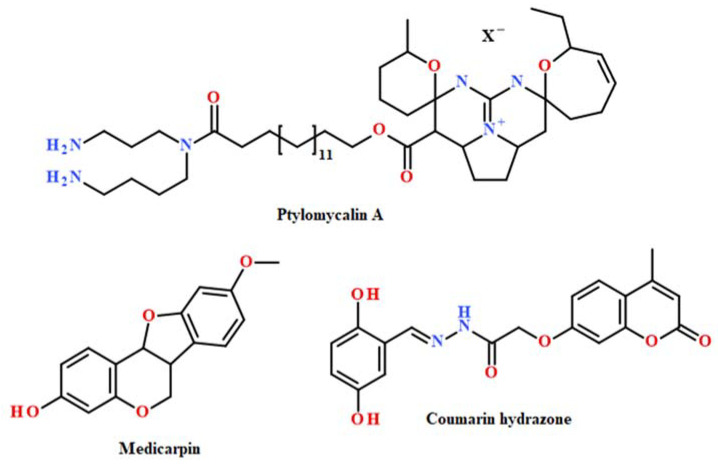
Structures of laccase inhibitors: ptylomycalin A, medicarpin, and coumarin hydrazone.

Laccase inhibitors are a diversified group of organic and inorganic compounds [24]. To date, only a few examples of natural-derived compounds with inhibition potency towards laccase have been reported (Figure 1) [25,26,27,28]. These are hard-to-access medicarpin isolated from *Dalbergia congestiflora* Pittier heartwood [25], ptylomycalin A isolated from the marine sponge *Monanchora arbuscula* [26], and a mixture of humic acids with an undefined composition [27]. Another example is a semi-synthetic hydrazide-hydrazone, the derivative of coumarin and gentisaldehyde (2,5-dihydroxybenzaldehyde) [28]. Among them, the humic acids seem to be applicable as laccase inhibitors because of their environmental abundance. Nonetheless, the studies on laccase inhibition had only a preliminary character. Unfortunately, their applications have disadvantages because of the undefined chemical structure of the humic acid mixture [27] and their activity against a broad range of enzymes [29].

Hydrazide-hydrazones are usually crystalline, easily available organic compounds. They may be synthesized in organic solvents [30] in the presence, or without, of acid additives with generally good to quantitative yields [31]. These are composed of carbonyl compounds linked by the hydrazine unit with carboxylic acid (RR′C=N–NH–(C=O)R″) or sulfonic acids (RR′C=N–NH–(SO_2_)R″) fragments [32]. They undergo easy dehydration to 1,3,4-oxadiazoles that present a variety of biological activities [33,34]. Hydrazide-hydrazones provide interesting properties in the field of supramolecular architecture [35,36,37,38,39], organic synthesis [33,40], chemo- and physical imaging [41,42,43,44], coordination [45,46,47,48,49], and in inorganic chemistry [50,51,52,53]. The numerous biologically active hydrazide-hydrazones are particularly worth paying attention to [54]. Depending on the character of the present functional groups, these molecules are anticancer [43,55,56,57,58], antimicrobial [32,59,60,61,62], antiviral agents [63,64], and enzyme inhibitors [31,48,65,66,67,68,69,70,71,72,73,74,75,76,77,78]. Depending on the class of inhibited enzymes, the latter group may be further divided into monoamine oxidases [67,71,79], lipid metabolism enzymes [74,75], and metalloenzyme inhibitors [31,65,66,68,69,70,77,78,80]. Nevertheless, knowledge about the hydrazide-hydrazones as inhibitors of metalloenzymes is still limited only to several studies reported in the literature so far. These compounds differ from the acyl unit, a heterocycle such as an imidazole, pyrazole, thiazole, or pyridyl derivatives, or an alkylated and/or electron-rich substituted benzene ring. There is no simple pattern that could link the activity of the hydrazide-hydrazones with metalloenzymes. In some cases, the hydrazide unit might act as a chelator agent of O and N coordination centers. Still, depending on the location of metal atoms in the protein, this mechanism must be carefully considered. The benzylidene unit is more diversified, but generally, the most common substituents are OH, OCH_3_, CH_3_, or halogen [31,65,66,68,69,70,77,78,80]. The salicylidene unit is common in tested metalloenzyme inhibitors. Furthermore, there are only two examples in which the hydroxy group is localized in the benzene ring of the acyl unit. An example is a 3,4-dihydroxybenzoic acid [80], a catechol, and a well-known laccase substrate [81,82,83]. The next examples are the derivatives of 3- or 4-monohydroxybenzoic acid, both described in our previous work. We investigated the role of the benzylidene unit of hydrazide-hydrazones in laccase inhibition [31]. We showed that the 4-hydroxybenzoyl fragment derived from 4-hydroxybenzoic acid (4-HBA) constitutes a specific decoy being the mediator of reactions catalyzed by laccase [84]. However, the units from salicylic aldehydes are also responsible for specific interaction with amino acids in the substrate cavity of the enzyme. These results showed that, among the slim-shaped molecules, the most active representatives act as competitive inhibitors with the *K*_I_ in the range of 24.0–26.4 µM. Interestingly, the sterically hindered imine derivative with a 3,5-di-*tert*-butyl-salicylidene unit has the most promising *K*_I_ = 17.9 µM and acts as an uncompetitive inhibitor. Generally, the values of the constant inhibition classify these compounds as effective agents that inhibit laccase.

**Figure 2:**
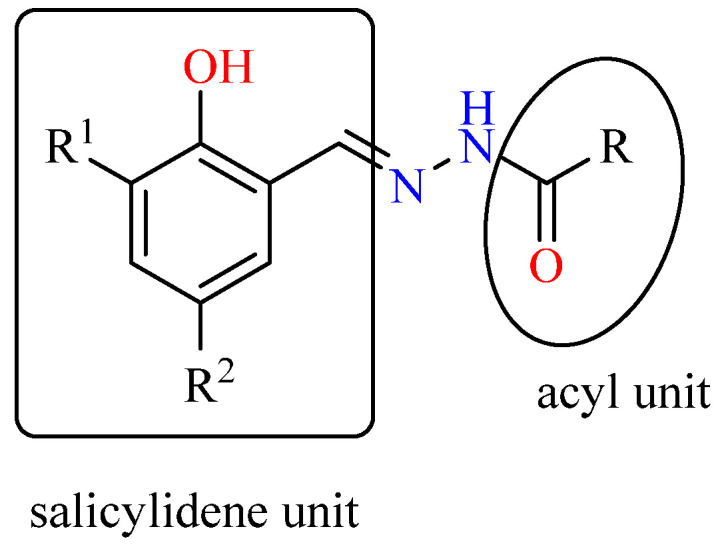
The general structure of investigated hydrazide-hydrazones. The fragment derived from salicylic aldehydes is a salicylidene unit, and the acyl unit originated from acid hydrazides.

We aimed this enzyme as a target since it is secreted by pathogenic fungi, contributing to various plant diseases. We believe that laccase inhibition might prevent or weaken pathogen attacks by chemical protection. Continuing our previous study, in the present article, we have addressed the effect of changing the structure of the acyl unit in hydrazide-hydrazones while retaining their salicylidene character on laccase activity, see Figure 2.

## 2. Results and Discussion

### 2.1. Syntheses and Characterizations

**Scheme 1:**
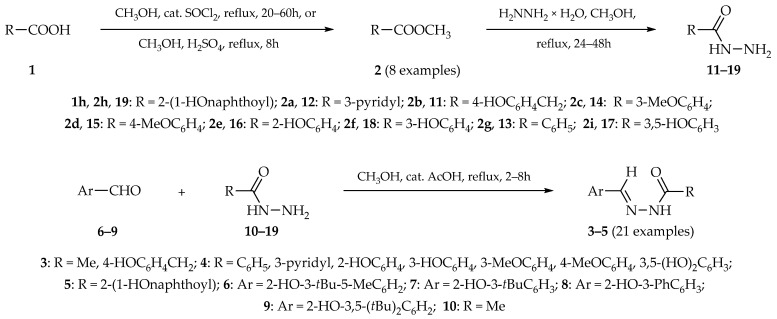
Preparation of hydrazide-hydrazones **3a**–**b**, **4a**–**o**, and **5a**–**d** scheme started from carboxylic acids **1** via carboxylic acid methyl esters **2** and carboxylic acid hydrazides **11**–**19**.

We synthesized and investigated the new phenolic hydrazide-hydrazones as a part of our ongoing project to discover aromatic compounds as antifungal and antiviral agents and key enzyme inhibitors of pathogenic microorganisms and viruses [31,85,86,87,88,89,90]. The target products were synthesized in a three-step procedure starting from carboxylic acid **1** via carboxylic acid methyl ester **2** [91] as the first intermediate product. The final hydrazide-hydrazone products **3**–**5** [31] were obtained by the direct condensation reaction of salicylic aldehydes **6**–**9** [92,93] and carboxylic acid hydrazides **10**–**19** [94], using the literature procedure [31], Scheme 1, Table 1. Products **3**–**5** were formed quantitatively and characterized. The high purity samples for the enzymatic study were prepared by direct crystallization from the reaction mixtures with a good to almost quantitative yield between 81 and 99%. 

Among the spectroscopic methods used, the infrared spectra are the most diagnostic in the analysis of product formation due to the vanishing of the band with a very strong intensity usually observed around 1700 cm^−1^ that corresponded to stretching CH=O group vibration. Furthermore, the IR method is also helpful in monitoring the reaction progress and determining the product purity. Generally, the ATR-IR spectra of the functionalized salicylaldehyde derivatives **3**–**5** had an extensive absorption band between 3300–2500 cm^−1^. This absorption contained bands corresponding to stretching vibrations of O-H, N-H, C_Ar_-H, and CH_2_ or CH_3_ of alkyls. The band from stretching vibration of O-H bond was not observed in most of the analyzed spectra as a characteristic one. It probably contributed to a mentioned broad absorption typical for crystalline compounds with strong H-bonding [97]. The vibrations of the hydrazide fragment are frequently described as amide vibrations, in which stretching vibration of C=O bond is named *amide I* and bending vibration of NH group—*amide II*. These are relatively strong and the medium absorptions found between 1700–1600 cm^−1^ and 1650–1550 cm^−1^, respectively. The other characteristic bands expected in the FT-IR spectrum correspond to stretching vibrations of C=N and C-O bonds. For all of the hydrazide-hydrazones **3**–**5**, the wavenumbers corresponding to the following vibrations were assigned as following 3248–3164 cm^−1^ to stretching N-H bond, 1655–1591 cm^−1^ the *amide I*, 1615–1580 cm^−1^ *amide II*, 1567–1527 cm^−1^ stretching imine bond (C=N), and 1265–1227 cm^−1^ to stretching C-O phenolic bond. In the cases of methoxylated compounds **4c** and **4d**, the following stretching O-CH_3_ vibration was observed at 1044 cm^−1^ and 1029 cm^−1^, respectively. 

The structural similarities of the synthesized hydrazides **11**–**19** to the corresponding hydrazide-hydrazones **3**–**5** resulted in partial coverage of the wavelength values in their IR spectra. The broad absorption between 3300–2500 cm^−1^ contained stretching vibrations the bands of NH_2_ (3251–3182 cm^−1^), and N-H (3329–3283 cm^−1^), and C-H alkyl groups vibration in hydrazides **11**, **14**, and **15** (2963–2809 cm^−1^). Band of O-H stretching vibration was also not observed in this region. Absorptions with medium and strong intensities of *amide I* and *amide II* vibrations were attributed to bands between 1673–1605 cm^−1^ and 1604–1547 cm^−1^, respectively. The other characteristic bands corresponded to the stretching vibration of C-O in phenolic and O-CH_3_ in alkyl substituents found between 1269–1249 cm^−1^ and 1034–1030 cm^−1^, respectively. The stretching vibration of the N-N bond had a weak absorption around 1000–1200 cm^−1^. Although one nitrogen atom in the N-N group is acylated, it remains highly symmetrical and is not a diagnostic band in infrared spectra [98].

All di-aryl hydrazide-hydrazones numbered **4** and **5** were isolated as pure geometric isomers *E*. These having acetyl units numbered **3a** and **3b** were isolated as *E* isomer predominantly with the ratio 79:21 and 87:13, respectively. We determined the ratio of isomers based on CH=N integration in ^1^H-NMR spectra.

### 2.2. Kinetic Studies

**Figure 3:**
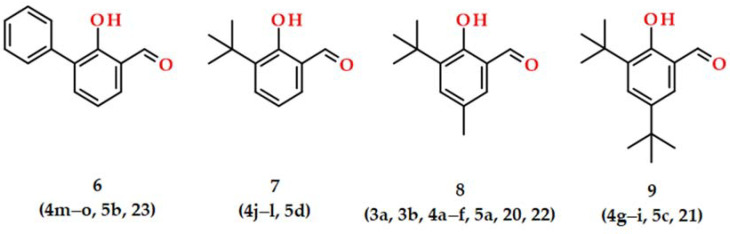
The structure of the aldehydes **6**–**9** used in the synthesis of corresponding target salicylic hydrazide-hydrazones **3**–**5** and **20**–**23** control compounds (enumerated in parentheses).

In our previous work, a 4-hydroxybenzoic acid (4-HBA) fragment of choice played a pivotal role in laccase inhibition as a decoy similar to its substrate. We showed that the salicylic aldehyde fragment with a bulky group localized neighbor to the hydroxy group, presented in Figure 3, contributes to a strong stabilizing effect with the hydrophobic area in a substrate-binding pocket [31]. In the present work, we extended the structure–activity relationship (SAR) after the change of acyl fragment (Figure 2) introduced from the carboxylic acid. We preserved the salicylidene unit in hydrazide-hydrazone molecules introduced directly from salicylic aldehyde substrates **6**–**9**. 

We synthesized a set of twenty-one hydrazide-hydrazones from four salicylic aldehydes of choice **6**–**9** (Figure 3) and ten differently substituted acid hydrazides **10**–**19**. 

Considering the possible interactions of the salicylic acid fragment with a substrate-binding site in the enzyme, we used two alkyl hydrazides **10**–**11** and eight different aromatic acid hydrazides **12**–**19**. The alkyl hydrazides were acetic acid hydrazide **10** and 2-(4-hydroxyphenyl)acetic acid hydrazide **11**. The aromatic unit contained 3-pyridyl **12**, benzoic **13**, and the potential substrates for laccase such as OH and OMe differently substituted arenes **14**–**19**. Among them, 3-, 4-methoxy-, 2-, 3-hydroxy-, and 3,5-dihydroxybenzoic, **14**–**15**, **16**–**17**, and **18**, respectively, and also 2-(1-hydroxy)naphthoic acid hydrazide (**19**) were used.

Based on the character of the acyl fragment, we formally divided the tested hydrazide-hydrazones numbered **3**–**5** into three groups. The first group contained two alkyl representatives, methyl and 4-hydroxybenzyl in **3a** and **3b**, respectively. The second group included a strong electron-deficient 3-pyridyl or phenyl rings in **4a** and **4b**, respectively, and an electron-rich phenyl ring having a strong electron-donating methoxy **4c**, **4d**, hydroxy **4e**, **4g**, **4h**, **4j**, **4k**, **4m**, **4n**, and dihydroxy **4f**, **4i**, **4l**, **4o** substituents. The structures of the compounds **5a**–**d** in the third group contained a bigger 1-hydroxy-2-naphthoyl ring that provides their slim-like character (Table 2). To compare the inhibition potency of new compounds, we chose NaN_3_ as a classic standard reference. Additionally, we used hydrazide-hydrazone derivatives of 4-HBA numbered **20**–**22** and 4-methoxybenoic acid derivative **23** negative standards described in our previous work (Table 2) [31]. 

**Figure 4:**
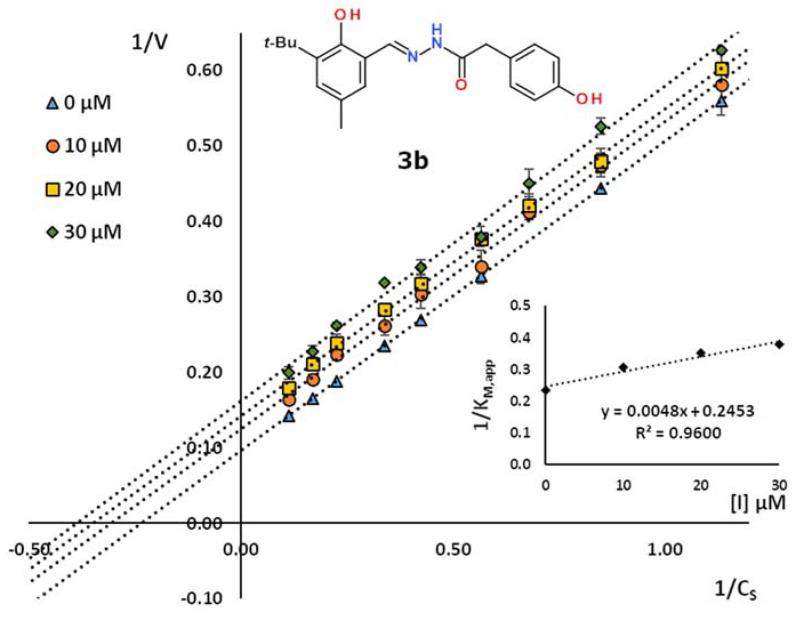
The Lineweaver–Burk plots (1/V [min·µM^−1^] = f(1/C_S_) [µM^−1^]) for **3b** performed for the inhibitor concentration up to 30 µM. The additional graphs show the linear dependence of 1/K_M,app_ = f(I) [µM] related to the uncompetitive mechanism.

The kinetic studies showed that the nineteen hydrazide-hydrazones exhibited an inhibition potency in the micromolar range of *K*_I_ between 8.0–233 µM and different modes of interaction with the enzyme molecules. Nine compounds **3a**–**b**, **4a**–**f**, and **5a** (Table 2) contain the 3-*tert*-butyl-5-methyl-salicylidene leading motif introduced from aldehyde **8** (Figure 2). We used previously reported hydrazide-hydrazone 20 derived from leading aldehyde 8 and 4-hydroxybenzoic acid hydrazide to compare the kinetic results. It acted as the competitive inhibitor with a constant *K*_I_ = 26.4 µM. Among the hydrazide-hydrazones, the acetyl derivative **3a**, and nicotinoyl derivative **4a**, were not active at the desirable micromolar range. The benzoyl derivative **4b** had a moderate activity with *K*_I_ = 82.0 µM. Comparing it with **3b** derivative with *para* hydroxylated benzene ring with methylene linker, almost a two-fold improvement of inhibition activity was observed (*K*_I_ = 49.1 µM). On the other hand, the alteration from standard **20** having the 4-hydroxyphenyl framework to above compound **3b** resulted in an almost two-fold drop in the activity, which confirmed our predictions that the hydroxylated aromatic fragment serves as a decoy and is required on the benzoyl fragment. Despite the **3b** seeming structurally like laccase substrate, the distance of the *para*-hydroxylated phenyl ring of the molecule core resulted in its higher affinity to the enzyme-substrate complex (Figure 4). Similar to **3b**, the more hindered salicylic acid derivatives, the **4e**, **4g**, **4j**, and **4m**, also did not act as competitive inhibitors. Among the discussed compounds, **4e** molecule acted as an uncompetitive laccase inhibitor, which corresponded to dropped inhibition potency approximately six-fold compared to the control **20**, both derivatives of our leading aldehyde. As it resulted from kinetic measurements, removing the smallest alkyl group in the 5-position of the aldehyde fragment increases the inhibition potency from 150 µM to 65.6 µM for **4e** and **4j** compounds, respectively, simultaneously preserving the uncompetitive type of action. 

On the other hand, replacing the 3-*tert*-butyl group in **4j** on a phenyl ring in **4m** resulted in a change of inhibition mechanism to a mixed type. Such structural modification in **4m** improved the value of constant inhibition of competitive component to 26.4 µM, at the same value as for the control **20**. Comparing the compounds having bulky 3,5-di-*tert*-butyl-2-hydroxy-benzylidene unit showed that a change of 4-hydroxybenzoyl group in control **21** to 2-hydroxybenzoyl in **4g** resulted in a three-fold decrease in inhibition activity. Interestingly, the only exception was the derivative **4m** of 2-hydroxybenzoyl and 3-phenyl-salicylidene units, for which the value of the competitive component *K*_I_ = 26.4 µM remained the same as for control **20**. 

Well-known facts supported the choice of the salicylic acid hydrazide as the substrate for synthesizing potent hydrazide-hydrazone laccase inhibitors. Salicylic acid (SA) is a natural signaling molecule that activates plant defense responses to a pathogen attack [100,101]. The SA functional analogs are applied in the crop protection sector [102], and the salicylic acid [103] and a sulphonyl salicylic acid [104] are not laccase substrates. Moreover, the SA is not a promotor of laccase production in white-rot fungi culture [105].

**Figure 5:**
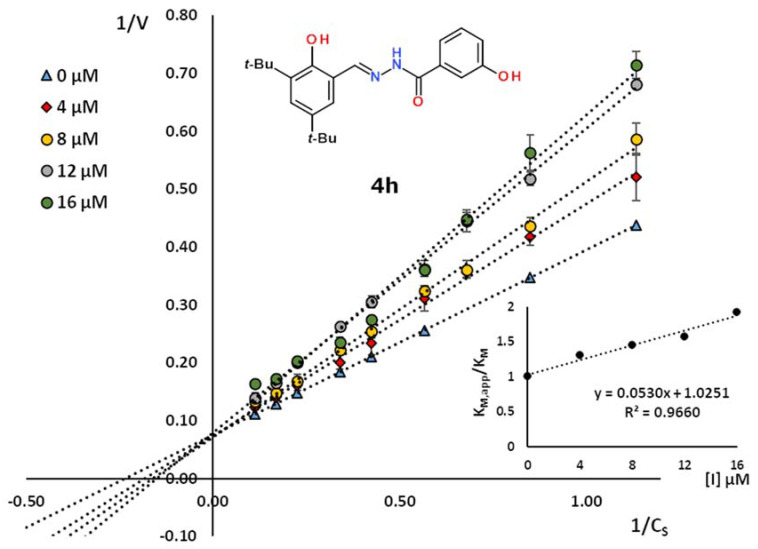
The Lineweaver–Burk plots (1/V [min µM^−1^] = f(1/C_S_) [µM^−1^]) for **4h** performed for the inhibitor concentration up to 16 µM. The additional graphs show the linear dependence of *K*_M,app_/*K*_M_ = f(I) concerning the competitive mechanism.

The remaining hydrazide-hydrazones of the second group are also benzoyl mono- and di-hydroxylated in 3-position or 3,5-positions derivatives **4h**, **4k**, **4n** or **4f**, **4i**, **4l**, **4o**, respectively. Five of seven compounds were pure competitive inhibitors with good inhibition potency between 18.9–35.8 µM and 32.3–55.6 µM for mono- and di-substituted, respectively. Comparison of appropriate pairs of 3-hydroxy and 3,5-dihydroxy (*α*-resorcinic acid unit) derivatives **22** and **4f**, **4h** and **4i**, and **4k** and **4l** showed their inhibition potency and the beneficial effect of benzoyl fragments on laccase inhibition. Compounds **4k** and **4l** and **22** and **4f** had comparable inhibition effects, and these pairs derived from aldehyde **7** and **8** differ the CH_3_ group in 5-position, respectively, see Figure 3. We observed the most significant difference between the last pair of inhibitors **4h** and **4i** with a common backbone of di-*tert*-butyl-salicylic aldehyde (**9**). These compounds acted as competitive inhibitors. For example, see Figure 5 for mono-hydroxylated on acyl unit derivative **4h**. This molecule was three-fold more effective than its disubstituted counterpart **4i**, because the crowded phenolic rings seemed too large to effectively interact with the substrate cavity. 

In general, a hydroxy group in *meta*-position may offer preferential interactions with the substrate cavity in the enzyme. Changes in the positioning of the single 3-OH group may occur by flipping around the axis C1–C4 of the benzoyl ring. We expected a similar but strengthened interaction for disubstituted *α*-resorcinic acid hydrazide. In this case, interaction in symmetrically localized hydroxy groups was probably slightly weaker or unfavorable to the fit of the molecules in the active site. 

**Figure 6:**
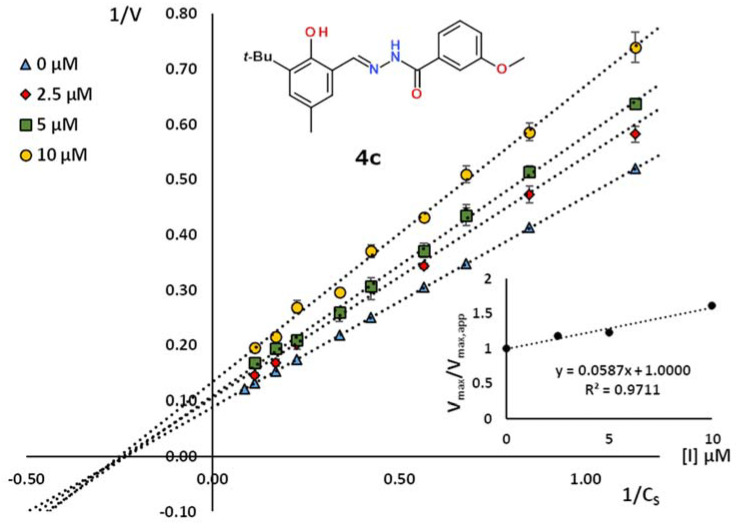
The Lineweaver–Burk plots (1/V [min µM^−1^] = f(1/C_S_) [µM^−1^]) for **4c** the inhibitor concentration up to 10 µM. The additional graphs show the linear dependence of V_max_/V_max,app_ = f(I) [µM] for non-competitive mechanism.

Two remaining compounds, **4o** and **4n**, have a common 3-phenyl salicylaldehyde fragment. These present the lowest inhibition potency between 69.9 and 233 µM with the mixed or non-competitive type of inhibition. On the other hand, these derivatives showed improved activities compared to an inactive control **23** with the aldehyde **6** core but methoxy substituent in acyl fragment. It seems that the bulky electron-rich phenolic substituent represents adverse properties to bind to the substrate-binding cavity. Interestingly, the hydrazide-hydrazone **4c** and **4d,** which are methoxylated in *meta*- and *para* position of benzoyl unit and containing the common aldehyde **8** core, are the most active in this group. The constants inhibition of **4c** and **4d** are 17.4 µM and 25.8 µM, respectively. The slim **4d** hydrazide-hydrazone has a high affinity to substrate-binding pocket. A mobile methoxy group localized in *meta* position in **4c** prevented the interaction with the active site of the enzyme (Figure 6). It was in agreement with the reactivity of *para*-methoxylated arenes such as *para*-anisic alcohol in the presence of laccase [106]. Further discussion of OCH_3_ needs developing experimental and theoretical studies, which might also open a new issue about laccase inhibitors. 

**Figure 7:**
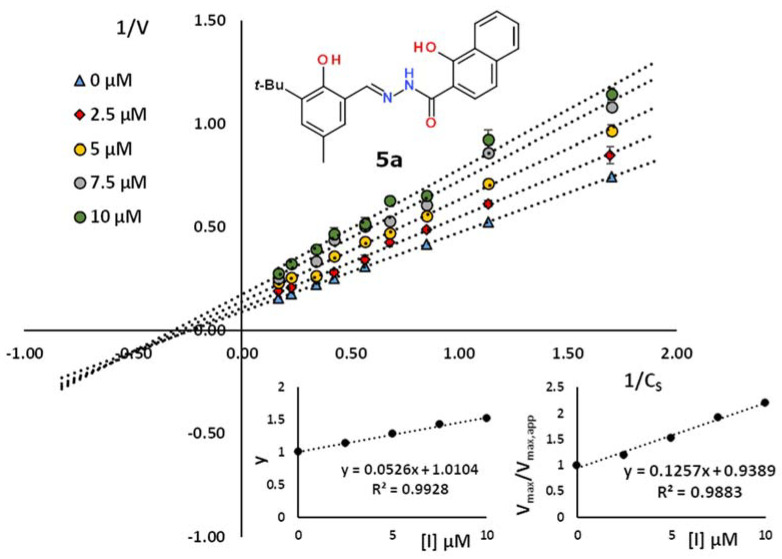
The Lineweaver–Burk plots (1/V [min·µM^−1^] = f(1/C_S_) [µM^−1^]) for **5a** performed for the inhibitor concentration up to 10 µM. The additional graphs show the linear dependence of *K*_M,app_ ·V_max_/(*K*_M_ ·V_max,app_) = f(I) concerning the competitive component in a mixed type mechanism.

Finally, the third group numbered **5a**–**d** contains a 2-hydroxynaphtalene hydrazide leading fragment (Table 2). Such substituent would require a large area in the enzyme-substrate cavity for hydrophobic interaction of such a bulk polycyclic arene and provide possible hydrogen bonding with hydrophilic amino acids. As it resulted from form experiments, three out of four compounds acted as mixed inhibitors. For **5a**, **5b**, and **5d**, the non-competitive component *K*_I_ had a consistently lower value than the competitive counterpart. Together with the **5b** derivative, they acted as non-competitive inhibitors preferentially. Furthermore, the magnitude of inhibition constants indicates that the effect of mono-substitution of aldehyde fragment in 3-position (**5b** and **5d**) is insufficient to obtain higher inhibition potency than 24.3 µM (Table 2). The additional alkyl substituent in 5-position improved inhibition potency. The small CH_3_ group in **5a** preserved the mixed type of inhibition (Figure 7) with a very good value of non-competitive component *K*_I_ = 8.0 µM and a good value of competitive constant equal to 19.0 µM. The additional bulk *tert*-butyl group in position 5 in compound **5c** changed the mechanism to pure non-competitive with relatively good *K*_I_ = 16.2 µM. The found hydrazide-hydrazones with the highest activity towards laccase will be the subject of further research on SAR and future application to protect cultivating plans against phytopathogenic fungi [107]. 

### 2.3. Docking Studies

**Figure 8:**
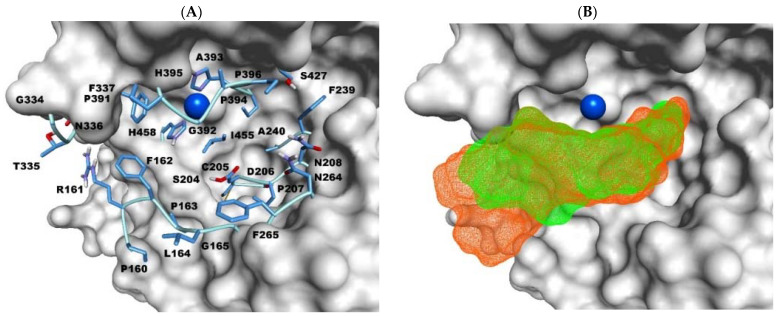
The active center of the laccase (PDB: 1GYC) with selected amino acids building the cavity (**A**) and the position of investigated inhibitors shown as grids (**B**). The sidechains of amino acids are shown as blue sticks and the protein is shown as a light blue tube and its surface is colored gray. The Cu ion is shown as a blue sphere. The mesh of the competitive inhibitors is green, uncompetitive, non-competitive, and mixed is orange.

The compounds used in the enzymatic assay were docked in the active center of the laccase (PDB 1GYC). Used procedures were the same as in the previous publication on our laccase research [25]. This time, however, we decided to put forward a hypothesis and check whether the changes in the inhibition type may depend only on the construction of the active center, previously called the substrate cavity. Especially, that several of the inhibitors we have tested showed a mixed mechanism of inhibition. The amino acids Asp206, Asn208, Asn264, and His258 played a key role in interacting with active compounds. They carry an electron from the substrate to the oxygen cavity. However, the other numerous amino acids building the active site (Figure 8A) have a significant influence on the location of the substrate in the active site. The laccase active center can be divided into two hydrophobic parts and one creating numerous hydrogen bonds. Rather, the compounds we investigated fill the volume of one of the hydrophobic cavities and the area of hydrophilic interactions. The position of selected inhibitors is shown in Figure 8B: compounds with a competitive mechanism are shown as a green mesh, while the others are shown as an orange mesh. The inhibitors with the lowest values of the inhibition constants are shifted deeper into the active center.

The compounds used in the enzyme assay were docked to the active site of laccase (PDB 1GYC). The procedures used were the same as in the previous publication on our research on laccase [31]. This time, however, we decided to make a hypothesis and check whether the changes in the type of braking may depend only on the construction of the active center, previously called the ground cavity. Especially since several of the inhibitors we have tested exhibited a mixed inhibition mechanism. The amino acids Asp206, Asn208, Asn264, and His258 played a crucial role in interacting with active compounds. They transfer an electron from the substrate to the oxygen cavity. However, numerous other amino acids building the active site (Figure 8A) have a significant influence on the location of the substrate in the active site. The active site of laccase can be divided into two hydrophobic parts and one forming numerous hydrogen bonds. Rather, the compounds we investigated fill the volume of one of the hydrophobic cavities and the area of hydrophilic interactions. The position of selected inhibitors is shown in Figure 8B: compounds with a competition mechanism are shown as a green grid, while others are shown as an orange grid. Inhibitors with the lowest values of inhibition constants are shifted deeper into the active center. 

**Figure 9:**
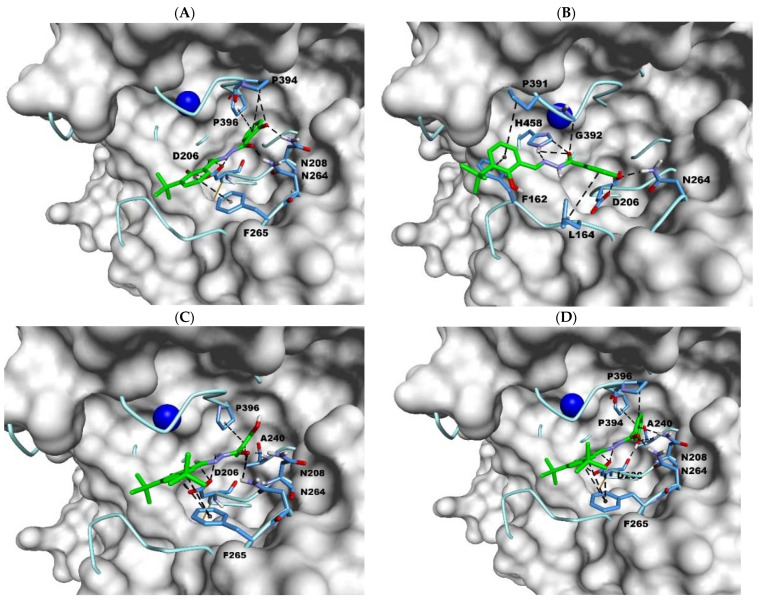
The active center of the laccase (PDB: 1GYC) with the interaction between selected amino acid and inhibitors: **4j** (**A**), **4k** (**B**), **4g** (**C**), **4h** (**D**). The amino acids are shown as blue sticks and the protein is shown as a light blue tube, and its surface is colored gray. The Cu ion is shown as a blue sphere. The interactions are black dashed lines.

The pairs of the inhibitors like **4j** and **4k**, and **4g** and **4h** have a common backbone. The slide difference between them is the position of the hydroxyl group from the hydrazide fragment. That difference plays a key role in the type of binding and inhibition of the compounds. Derivative of salicylic acid **4j** having one *tert*-butyl group (Figure 9A) is placed like inhibitors from our earlier studies [31]. The hydrophobic ring with the *tert*-butyl substituent is directed toward the Phe265 with medium-range aromatic interactions (4.29 Å). Asp206 is involved in a hydrogen bond with its hydroxyl group (1.86 Å) and with the backbone nitrogen atom (2.22 Å). The other hydrogen positioning of the inhibitor is seen by the interactions with Asn208 (2.05 Å) and Asn264 (1.85 Å). Those close distance non-bond bindings are strengthened by Pro394 (3.60 Å, 5.17 Å) and Pro396 (4.93 Å). The derivative **4k** (Figure 9B) with a hydroxy group in *meta* position has a different inhibition mechanism, and it is slightly moved of the cavity depth. In this case, the hydrazide hydroxyl group interacts with Asn206 (2.20 Å) and Asn264 (2.04 Å). The position of the ring is stabilized by extra hydrophobic interactions with Leu164 (5.20 Å). The carbonyl oxygen atom and the hydrazide nitrogen create a hydrogen bond triangle with His458, and the position of the ring is stabilized by Phe162 (4.22 Å) and Pro391 (5.06 Å). 

The larger molecules having two *tert*-butyl substituents in the salicylidene framework, such as **4g** (Figure 9C) and **4h** (Figure 9D) have less freedom in the binding. They are placed deep in the pocket engaging the same set of amino acids. The different position of a hydroxyl localized on benzoyl unit in *ortho* vs *meta* in **4g** and **4h**, respectively, causes the flip of the phenyl ring. Consequently, the extra hydrogen bond between 4h and the carbonyl oxygen of the Asp206 (1.67 Å) is created. The hydrogen interaction of the hydroxyl group with Asp206 is of the most significant importance in the inhibition mechanism by blocking the flow of electrons. In other instances, the inhibition is by blocking the substrate’s access site for the amino acid. The presence of a hydrogen bond between the hydroxy group and Asn208 can also change the type of inhibition from uncompetitive to non-competitive (**4j** vs **4g**). Compounds with methoxy substituents **4c** and **4d** are also pairs of inhibitors with different inhibition types. The *meta* position of the methoxy group **4c** creates an unpreferred position in the cavity space. The freedom of the rotation is not appreciated in the deep binding of the inhibitor. That creates a phenomenon, the **4c** is turned opposite to the projected mechanism. In contrast, the **4d** binds properly to the inhibition types and interacts with the Asp206. 

**Figure 10:**
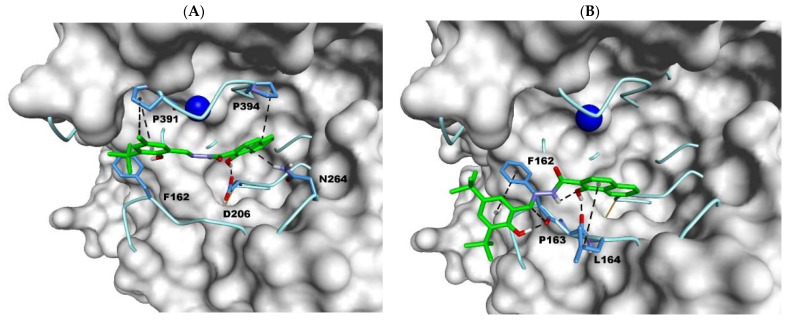
The active center of the laccase (PDB: 1GYC) with the interaction between selected amino acid and inhibitors: **5a** (**A**), **5c** (**B**). The amino acids are shown as blue sticks, and the protein is shown as a light blue tube, and its surface is colored gray. The Fe ion is shown as a blue sphere. The interactions are black dashed lines.

The thesis is also confirmed by analyzing the interaction of more hydrophobic compounds having a naphthoic ring in their structure with amino acids in the active center. Compound **5a** (Figure 10A) had the lowest value of the inhibition constant, but it has a mixed mode of inhibition. The low value of the non-competitive inhibition constant for the binding method of this inhibitor is related to the fact that it is located deep but also in its upper part in the active center, blocking access to His458. Additionally, **5a** forms a strong hydrogen bond with Asp206 (1.79 Å). The lack of interaction with the amino acids that make up the cavity’s lower area is one of the reasons for the dual binding mechanism. However, **5c** (Figure 10B) is bound to amino acids of the lower part of the active center but does not interact with the Asp206, and it has the non-competitive type of inhibition. 

This leads to the conclusion, the compound to inhibit laccase competitively must interact with amino acids from the depths and bottom of the active center, such as Asp206, Asn264, Leu 164, or Phe265. Another criterion required for this type of inhibition is the formation of a strong hydrogen bond between Asp206 and the inhibitor using heteroatoms that do not build the backbone of the inhibitor, blocking the flow of electrons. 

## 3. Materials and Methods

All commercially available chemicals were purchased as pure for synthesis or analytical grade reagents (Sigma-Aldrich Poznan, Poland, ARMAR, Wroclaw, Poland) and solvents were used mostly without further purification. In particular, nicotinic acid methyl ester (**2a**), 2-(4-hydroxyphenyl)-acetic acid methyl ester (**2b**), 3-*tert*-butyl-salicylic aldehyde (**7**), 3,5-di-*tert*-butyl-salicylic aldehyde (**9**), acetic acid hydrazide (**10**), and hydrazine monohydrate purchased in Sigma-Aldrich, and syringaldazine (SNG, 4-hydroxy-3,5-dimethoxybenzaldehyde azine), dimethylsulfoxide (DMSO), citric acid monohydrate, and sodium phosphate dibasic dodecahydrate, purchased in POCh (Poland) were used without further purification. Laccase from *Trametes versicolor* was purchased in lyophilized powder from Sigma-Aldrich (Poznan, Poland). 

Methanol (CH_3_OH) was distilled prior to reactions from Mg element shavings in the presence of I_2_. The following substrates such as 3-phenylsalicylicaldehyde (**6**), 3-*tert*-butyl-5-methylsalicylic aldehyde (**8**), 4-methoxybenzoic acid hydrazide (**15**), 3-hydroxybenzoic acid hydrazide (**18**), and reference hydrazide-hydrazones 4-hydroxy-*N*’-[(*E*)-3-*tert*-butyl-2-hydroxy-5-methylbenzylidene]benzohydrazide (**20**), 4-hydroxy-*N*’-[(*E*)-(3,5-di-*tert*-butyl-2-hydroxyphenyl)methylidene]benzohydrazide (**21**), 3-hydroxy*-N*’-[(*E*)-(3-*tert*-butyl-2-hydroxy-5-methylphenyl)methylidene]benzohydrazide (**22**), and 4-methoxy-*N*’-[(*E*)-2-hydroxy-3-phenylbenzylidene]benzohydrazide (**23**) were synthesized and characterized in our previous work [31]. Analytical TLC was performed on PET foils precoated with silica gel (silica gel, 60 F254, Merck, Darmstadt, Germany), and were made visual under UV light (λ_max_ = 254 nm), or by staining with iodine vapor. Melting points were determined on an Electrothermal IA 91100 (ST. Louis, MO, USA) digital melting-point apparatus using the standard open capillary method. FT-IR spectra (4000–400 cm^−1^) were recorded as KBr plates on a Perkin–Elmer 2000 FT-IR (Manchester, UK) or Bruker VERTEX 70V spectrometer using a diamond ATR accessory and Nicolet iS50 spectrometer (Thermo Fisher SCIENTIFIC, Waltham, MA, USA) with a resolution of 4 cm^−1^ at wavenumbers in the range of 500–4000 cm^−1^. Absorption maxima were reported in wavenumbers (cm^−1^). ^1^H-NMR and ^13^C-NMR spectra were recorded on a Bruker DRX 300 Spectrometer (300.13 for ^1^H and 75.475 for ^13^C) on Jeol 400yh (Tokio, Japan) (399.78 for ^1^H and 100.52 for ^13^C) or a Bruker Advance 600 Spectrometer (Poznan, Poland) (600.58 for ^1^H and 151.03 for ^13^C) at 295 K. Chemical shifts (δ) are given in parts per million (ppm) downfield relative to TMS, and coupling constants (*J*) are in Hz. Residual solvent central signals were recorded as follows: DMSO-*d*_6_, δ_H_ = 2.500, δ_C_ = 39.43; CH_3_OH-*d*_4_, δ_H_ = 3.310, δ_C_ = 49.05; CDCl_3_, δ_H_ = 7.263, δ_C_ = 77.00. When measured, DEPT and ATP experiments signals are referred to as (+) or (–). High-resolution mass spectra (HRMS) were recorded on a Waters LCD Premier XE instrument (Manchester, UK), and only the [M + H]^+^ or [M + Na]^+^ molecular species are reported. Purity and homogeneity of known bezohydrazide **11**–**19**, methyl ester **2a** and **2b**, and aldehydes **6**–**9** were confirmed by measuring their melting points, FT-IR, ^1^H-NMR, and ^13^C-NMR spectra and/or HRMS and compared them with literature data. All new phenolic aroylhydrazide-hydrazones **3**–**5** were fully characterized. The position of hydrogen and carbon atoms in the NMR data was determined by supporting the standard dept-135 and ATP experiments and by the 2D-NMR (HMQC, HSQC, HMBC, and NOESY) experiments map analysis if measured. The spectra images of FT-IR and NMR for the following compounds are placed in Supplementary Materials as follows: Figures S1 and S2 Spectroscopic characterization of **1h**, Figure S3–S18 Spectroscopic characterization of **2a**, **2c**, **2e**, **2g**, **2h**, **2i**, Figure S19–S289 Spectroscopic characterization of hydrazide-hydrazones **3**–**5**, Figure S290–S293 Spectroscopic characterization of salicylic aldehydes **6** and **8**, Figure S294–S348 Spectroscopic characterization of acid hydrazides **11**–**19**.

### 3.1. Synthesis of Aroylhydrazones 3–5

To a mixture of salicylaldehyde **6**–**9** (2.0 mmol), and carboxylic acid hydrazide **10**–**19** (2.0 mmol) in CH_3_OH (5.0 mL), AcOH (200 µL) was added at RT and then the resulting solution was gently refluxed during stirring. The reaction progress was monitored by TLC. Then the reaction was finished. After slow cooling to room temperature (RT) and cooled to ca. +4 °C, the reaction mixture was left in a refrigerator (−24 °C) overnight [31]. The crystals formed were collected by filtration to give pure products **3**–**5**, fully characterized by determination melting point, analysis NMR, IR, and HRMS spectra. The known compound was identified by comparing its melting points and FT-IR and/or NMR spectra with literature data and/or by HRMS determination. The error of molar mass determination by HRMS method was ≤0.0005%. 

**(*E*/*Z*)-*N*’-(3-*tert*-butyl-2-hydroxy-5-methyl-benzylidene)acetohydrazide** (**3a**) 

The general procedure starting from 3-*tert*-butyl-5-methyl-salicylic aldehyde (**8**) (192 mg, 1.0 mmol) [108], ≥90% acetic acid hydrazide (**10**) (82 mg, ≥1.0 mmol), CH_3_OH (10 mL), and AcOH (100 µL) was employed with a 2.5 h reaction time to obtain colorless prisms of (*E*/Z)-*N*’-(3-*tert*-butyl-2-hydroxy-5-methyl-benzylidene)acetohydrazide (**3a**) (208 mg, 0.84 mmol) with 84% yield, which melts at 231–233 °C. Selected FT-IR (ATR): ν_max_ 3203 (N-H), 3065 (C_Ar_-H), 1654 (C=O), 1614 (N-H), 1591 (C=C), 1565 (C=N), 1428, 1439, 1360, 1276, 1239 (C_Ar_-O), 1208, 1123, 958, 860, 769, 710, 643, 569, 483 cm^−1^; (*E*) ^1^H NMR (400 MHz, DMSO-*d*_6_): δ 12.06 (s, 1H, OH), 11.70 (s, 1H, NHCO), 8.21 (s, 1H, CH=N), 7.07 (d, ^4^*J* = 1.6 Hz, 1H, ArH-4), 7.04 (d, ^4^*J* = 1.6 Hz, 1H, ArH-6), 2.23 (s, 3H, Ar-Me), 1.98 (s, 3H, Ac), 1.37 (s, 9H, *t*-Bu) ppm; (*Z*) ^1^H NMR (400 MHz, DMSO-*d*_6_): δ 11.38 (s, 1H, NHCO), 10.96 (s, 1H, OH), 8.11 (s, 1H, CH=N), 7.07 (d, ^4^*J* = 1.6 Hz, 1H, ArH-4), 7.00 (d, ^4^*J* = 1.6 Hz, 1H, ArH-6), 2.23 (s, 3H, Ar-Me), 2.17 (s, 3H, Ac), 1.37 (s, 9H, *t*-Bu) ppm; (*E*) ^13^C NMR (100 MHz, DMSO-*d*_6_): δ 165.23 (C=O), 154.48 (ArC-2-OH), 148.72 (CH=N), 135.95 (ArC-3), 129.19 (ArC-4), 129.07 (ArC-6), 126.84 (ArC-5), 117.20 (ArC-1), 34.28 (C—*t*-Bu), 29.15 (3 × CH_3_—*t*-Bu), 21.15 (Me—Ac), 20.15 (Ar-Me) ppm; (*Z*) ^13^C NMR (100 MHz, DMSO-d_6_): δ 170.62 (C=O), 153.71 (ArC-2-OH), 146.62 (CH=N), 136.01 (ArC-3), 129.11 (ArC-4), 129.00 (ArC-6), 127.39 (ArC-5), 117.63 (ArC-1), 34.28 (C—*t*-Bu), 29.21 (3 × CH_3_—*t*-Bu), 20.40 (Me—Ac), 20.15 (Ar-Me) ppm; HRMS (TOF, MS, ESI) *m*/*z* for C_14_H_20_N_2_O_2_ + H^+^ calculated: 249.1598; found: 249.1598. 

**2-(4-Hydroxyphenyl)-*N*’-[(*E*)-(3-*tert*-butyl-2-hydroxy-5-methylphenyl)methylidene]acetohydrazide** (**3b**) 

The general procedure starting from 3-*tert*-butyl-5-methyl-salicylic aldehyde (**8**) (192 mg, 1.0 mmol) [108], (4-hydroxyphenyl)-acetic acid hydrazide (**11**) (166 mg, 1.0 mmol), CH_3_OH (20 mL), and AcOH (100 µL) was employed with a 2 h reaction time to obtain the colorless prisms of 2-(4-hydroxyphenyl)-*N*’-[(*E*)-(3-*tert*-butyl-2-hydroxy-5-methylphenyl)methylidene]acetohydrazide (**3b**) (318 mg, 0.854 mmol) crystallized with one molecule of CH_3_OH) with 85% yield, which melts at 242–244 °C (CH_3_OH). Selected FT-IR (ATR): ν_max_ 3236 (N-H), 3064 (C_Ar_-H), 3032 (C_Ar_-H), 1654 (C=O), 1612 (N-H), 1585 (C=C), 1552 (C=N), 1515, 1468, 1363, 1299, 1261 (C_Ar_-O), 1240, 1143, 1022, 799, 769, 651, 642, 623, 521, 417, 435 cm^−1^; ^1^H NMR (400 MHz, DMSO-*d*_6_): δ 12.03 (s, 1H, Ar-2-OH), 11.86 (s, 1H, NHCO), 9.30 (s, 1H, C-4-OH), 8.27 (s, 1H, CH=N), 7.11 (d, ^3^*J* = 8.5 Hz, 2H, H-2,6), 7.07 (d, ^4^*J* = 2.1 Hz, 1H, ArH-4), 7.04 (d, ^4^*J* = 2.1 Hz, 1H, ArH-6), 6.72 (d, ^3^*J* = 8.5 Hz, 2H, H-3,5), 4.12 (k, ^3^*J* = 5.2 Hz, 1H, CH_3_OH), 3.44 (s, 2H, CH_2_), 3.17 (d, ^3^*J* = 5.2 Hz, 3H, CH_3_OH), 2.23 (s, 3H, Me), 1.38 (s, 9H, *t*-Bu) ppm; ^13^C NMR (100 MHz, DMSO-*d*_6_): δ 166.77 (C=O), 156.11 (C-4), 154.55 (ArC-2-OH), 149.49 (CH=N), 136.01 (ArC-3), 129.93 (C-2, C-6), 129.33 (ArC-4), 129.12 (ArC-6), 126.90 (ArC-5), 125.33 (C-1), 117.21 (ArC-1), 115.09 (C-3, C-5), 48.55 (CH_3_OH), 39.43 (CH_2_), 34.30 (C—*t*-Bu), 29.15 (3 × CH_3_—*t*-Bu), 20.18 (Me) ppm; HRMS (TOF, MS, ESI) *m*/*z* for C_20_H_24_N_2_O_3_ + Na^+^ calculated: 363.1679; found: 363.1698. 

***N*’-[(*E*)-(3-*tert*-butyl-2-hydroxy-5-methylphenyl)methylidene]-3-pyridohydrazide** (**4a**) 

The general procedure starting from 3-*tert*-butyl-5-methyl-salicylic aldehyde (**8**) (192 mg, 1.0 mmol) [108], nicotinic acid hydrazide (**12**) (137 mg, 1.0 mmol), MeOH (6.5 mL), and AcOH (100 µL) was employed with a 90 min. reaction time and concentrated before crystallization to obtain colorless plates of *N*’-[(*E*)-(3-*tert*-butyl-2-hydroxy-5-methylphenyl)methylidene]-3-pyridohydrazide (**4a**) (291 mg, 0.93 mmol) with 93 % yield, which melts at 230–231 °C. Selected FT-IR (ATR): ν_max_ 3183 (N-H), 3011 (C_Ar_-H), 1638 (C=O), 1613 (C=C), 1590 (N-H), 1552 (C=N), 1418, 1360, 1314, 1294, 1261 (C_Ar_-O), 1236, 1207, 1167, 1093, 1028, 955, 893, 867, 784, 768, 709, 670, 619, 517, 465, 409 cm^−1^; ^1^H NMR (400 MHz, DMSO-*d*_6_): δ 12.38 (s, 1H, NHCO), 12.15 (s, 1H, OH), 9.09 (dd, ^4^*J* = 2.4 Hz, ^5^*J* = 0.8 Hz, 1H, H-2), 8.79 (dd, ^3^*J* = 4.8 Hz, ^4^*J* = 1.7 Hz, 1H, H-4), 8.16 (s, 1H, CH=N), 8.28 (ddd, ^3^*J* = 7.9 Hz, ^4^*J* = 2.4 Hz, ^4^*J* = 1.7 Hz, 1H, H-6), 7.60 (ddd, ^3^*J* = 7.9 Hz, ^3^*J* = 4.8 Hz, ^5^*J* = 0.8 Hz, 1H, H-5), 7.12 (d, ^4^*J* = 1.8 Hz, 1H, ArH-4), 7.01 (d, ^4^*J* = 1.8 Hz, 1H, ArH-6), 2.26 (s, 3H, Me), 1.40 (s, 9H, *t*-Bu) ppm; ^13^C NMR (100 MHz, DMSO-*d*_6_): δ 161.29 (C=O), 154.77 (ArC-2-OH), 152.51 (C-4), 151.35 (CH=N), 148.53 (C-2), 136.14 (ArC-3), 135.41 (C-6), 129.71 (ArC-4), 129.33 (ArC-6), 128.37 (C-1), 127.06 (ArC-5), 123.64 (C-5), 117.13 (ArC-1), 34.34 (C—*t*-Bu), 29.17 (3 × CH_3_—*t*-Bu), 20.16 (Ar-CH_3_) ppm; HRMS (TOF, MS, ESI) *m*/*z* for C_18_H_21_N_3_O_2_ + H^+^ calculated: 312.1706; found: 312.1713. 

***N*’-[(*E*)-(3-*tert*-butyl-2-hydroxy-5-methylphenyl)methylidene]benzohydrazide** (**4b**) 

The general procedure starting from 3-*tert*-butyl-5-methyl-salicylic aldehyde (**8**) (384 mg, 2.0 mmol) [108], benzoic acid hydrazide (**13**) (272 mg, 2.0 mmol), CH_3_OH (65 mL), and AcOH (100 µL) was employed with a 2 h reaction time to obtain colorless powder of *N*’-[(*E*)-(3-*tert*-butyl-2-hydroxy-5-methylphenyl)methylidene]benzohydrazide (**4b**) (597 mg, 1.92 mmol) with 96%, which melts at 246–248 °C. Selected FT-IR (ATR): ν_max_ 3164 (N-H), 3058 (C_Ar_-H), 3032 (C_Ar_-H), 1635 (C=O), 1612 (N-H), 1578 (C=C), 1561 (C=N), 1440, 1364, 1317, 1264 (C_Ar_-O) 1285, 1207 (C_Ar_-O), 1170, 1088, 968, 889, 860, 768, 690, 672, 571, 512, 423 cm^−1^; ^1^H NMR (400 MHz, DMSO-*d*_6_): δ 12.45 (s, 1H, OH), 12.22 (s, 1H, NHCO), 8.53 (s, 1H, CH=N), 7.95 (dd, ^3^*J* = 7.3 Hz, ^4^*J* = 1.3 Hz, 2H, H-2,6), 7.63 (tt, ^3^*J* = 7.3 Hz, ^4^*J* = 1.3 Hz, 1H, H-4), 7.56 (dd, ^3^*J* = 7.3 Hz, ^3^*J* = 7.3 Hz, 2H, H-3,5), 7.10 (d, ^4^*J* = 1.8 Hz, 1H, ArH-4), 7.07 (d, ^4^*J* = 1.8 Hz, 1H, ArH-6), 2.26 (s, 3H, Me), 1.40 (s, 9H, *t*-Bu) ppm; ^13^C NMR (100 MHz, DMSO-*d*_6_): δ 162.67 (C=O), 154.71 (ArC-2), 150.75 (CH=N), 136.09 (ArC-3), 132.50 (C-1), 132.02 (C-4), 129.48 (ArC-4), 129.18 (ArC-6), 128.53 (C-3, C-5), 127.57 (C-2, C-6), 126.96 (ArC-5), 117.26 (ArC-1), 34.32 (C—*t*-Bu), 29.17 (3 × CH_3_—*t*-Bu), 20.17 (Ar-Me) ppm; HRMS (TOF, MS, ESI) *m*/*z* for C_19_H_22_N_2_O_2_ + H^+^ calculated: 311.1754; found: 311.1771. 

**3-Methoxy-*N*’-[(*E*)-(3-*tert*-butyl-2-hydroxy-5-methylphenyl)methylidene]benzohydrazide** (**4c**) 

The general procedure starting from 3-*tert*-butyl-5-methyl-salicylic aldehyde (**8**) (192 mg, 1.0 mmol) [108], 3-methoxybenzoic acid hydrazide (**14**) (166 mg, 1.0 mmol), CH_3_OH (45 mL), and AcOH (100 µL) was employed with a 4.5 h reaction time to obtain of 3-methoxy-*N*’-[(*E*)-(3-*tert*-butyl-2-hydroxy-5-methylphenyl)methylidene]benzohydrazide (**4c**) (317 mg, 0.931 mmol) with 93% yield, as a pale yellow crystals, as watt, which melts at 238–240 °C. Selected FT-IR (ATR): ν_max_ 3173 (N-H), 3064 (C_Ar_-H), 3008 (C_Ar_-H), 1633 (C=O), 1584 (N-H), 1560 (C=N), 1486, 1462, 1427, 1364, 1318, 1295, 1279, 1241 (C_Ar_-O), 1140, 1083, 1044, 970, 861, 811, 755, 714, 693, 570, 493, 466 cm^−1^; ^1^H NMR (400 MHz, DMSO-*d*_6_): δ 12.23 (s, 1H, NH), 12.17 (s, 1H, OH), 8.52 (s, 1H, CH=N), 7.53 (ddd, ^3^*J* = 7.8 Hz, ^4^*J* = 1.5 Hz, ^4^*J* = 1.1 Hz, 1H, H-6), 7.48 (dd, ^4^*J* = 2.5 Hz, ^4^*J* = 1.5 Hz, 1H, H-2), 7.47 (dd, ^3^*J* = 8.0 Hz, ^3^*J* = 7.8 Hz, 1H, H-5), 7.19 (ddd, ^3^*J* = 8.0 Hz, ^4^*J* = 2.5 Hz, ^4^*J* = 1.1 Hz, 1H, H-4), 7.11 (d, ^4^*J* = 1.7 Hz, 1H, ArH-4), 7.06 (d, ^4^*J* = 1.7 Hz, 1H, ArH-6), 3.84 (s, 1H, OMe), 2.26 (s, 3H, Me), 1.40 (s, 9H, *t*-Bu) ppm; ^13^C NMR (100 MHz, DMSO-*d*_6_): δ 162.41 (C=O), 159.20 (C-3), 154.73 (ArC-2-OH), 150.84 (CH=N), 136.10 (ArC-3), 133.88 (C-1), 129.73 (C-5), 129.49 (ArC-4), 129.18 (ArC-6), 126.97 (ArC-5), 119.76 (C-6), 117.65 (C-4), 117.27 (ArC-1), 112.88 (C-2), 55.30 (OMe), 34.33 (C—*t*-Bu), 29.17 (3 × CH_3_—*t*-Bu), 20.18 (Me) ppm; HRMS (TOF, MS, ESI) *m*/*z* for C_20_H_24_N_2_O_3_ + H^+^ calculated: 341.1860; found: 341.1852.

**4-Methoxy-*N*’-[(*E*)-(3-*tert*-butyl-2-hydroxy-5-methylphenyl)methylidene]benzohydrazide** (**4d**) 

The general procedure starting from 3-*tert*-butyl-5-methyl-salicylic aldehyde (**8**) [108] (192 mg, 1.0 mmol), 4-methoxybenzoic acid hydrazide (**15**) (166 mg, 1.0 mmol), CH_3_OH (20 mL), and AcOH (100 µL) was employed with a 2 h reaction time and concentrated by slow distillation before crystallization to obtain the 4-methoxy-*N*’-[(*E*)-(3-*tert*-butyl-2-hydroxy-5-methylphenyl)methylidene]benzohydrazide (**4d**) (276 mg, 0.81 mmol) as colorless needles with 81% yield, which melts at 243–244 °C. Selected FT-IR (ATR): ν_max_ 3165 (N-H), 3008 (C_Ar_-H), 1636 (C=O), 1605 (N-H), 1585 (C=C), 1537 (C=N), 1508, 1440, 1432, 1358, 1286, 1235 (C_Ar_-O), 1207, 1183, 1173, 1088, 1029, 971, 896, 838, 765, 689, 614, 500, 465 cm^−1^; ^1^H NMR (400 MHz, DMSO-*d*_6_): δ 12.28 (s, 1H, OH), 12.08 (s, 1H, NHCO), 8.51 (s, 1H, CH=N), 7.94 (d, ^3^*J* = 8.9 Hz, 2H, H-2,6), 7.09 (d, ^3^*J* = 8.9 Hz, 2H, H-3,5), 7.09 (d, ^4^*J* = 1.6 Hz, 1H, ArH-4), 7.06 (d, ^4^*J* = 1.6 Hz, 1H, ArH-6), 3.84 (s, 1H, OMe), 2.25 (s, 3H, Me), 1.39 (s, 9H, *t*-Bu) ppm; ^13^C NMR (100 MHz, DMSO-*d*_6_): δ 162.20 (C-4), 162.06 (C=O), 154.65 (ArC-2-OH), 150.09 (CH=N), 136.04 (ArC-3), 129.52 (C-2, C-6), 129.30 (ArC-4), 129.06 (ArC-6), 126.90 (ArC-5), 124.48 (C-1), 117.36 (ArC-1), 113.76 (C-3, C-5), 55.39 (OMe), 34.31 (C—*t*-Bu), 29.17 (3 × CH_3_—*t*-Bu), 20.18 (Me) ppm; HRMS (TOF, MS, ESI) *m*/*z* for C_20_H_24_N_2_O_3_ + H^+^ calculated: 341.1860; found: 341.1870. 

**2-Hydroxy*-N*’-[(*E*)-(3-*tert*-butyl-2-hydroxy-5-methylphenyl)methylidene]benzohydrazide** (**4e**) 

The general procedure starting from 3-*tert*-butyl-5-methyl-salicylic aldehyde (**8**) (192 mg, 1.0 mmol) [108], 2-hydroxybenzoic acid hydrazide (**16**) (152 mg, 1.0 mmol), CH_3_OH (15 mL), and AcOH (100 µL) was employed with a 2 h reaction time and concentrated by slow distillation before crystallization to obtain the 2-hydroxy-*N*’-[(*E*)-(3-*tert*-butyl-2-hydroxy-5-methylphenyl)methylidene]benzohydrazide (**4e**) (309 mg, 0.95 mmol) as a colorless prisms with 95% yield, which melts at 252–253 °C. Selected FT-IR (ATR): ν_max_ 3250 (N-H), 3033 (C_Ar_-H), 2999 (C_Ar_-H), 1637 (C=O), 1615 (N-H), 1581 (C=C), 1560 (C=N), 1456, 1436, 1361, 1319, 1308, 1235 (C_Ar_-O), 1204, 1158, 1098, 942, 904, 786, 768, 743, 719, 617, 530, 469, 447, 442 cm^−1^; ^1^H NMR (400 MHz, DMSO-*d*_6_): δ 12.17 (s, 1H, ArOH), 12.12 (s, 1H, NHCO or OH), 11.67 (s, 1H, NHCO or OH), 8.56 (s, 1H, CH=N), 7.88 (dd, ^3^*J* = 7.9 Hz, ^4^*J* = 1.6 Hz, 1H, H-6), 7.46 (ddd, ^3^*J* = 8.4 Hz, ^3^*J* = 7.2 Hz, ^4^*J* = 1.6 Hz, 1H, H-4), 7.11 (d, ^4^*J* = 1.7 Hz, 1H, ArH-4), 7.08 (d, ^4^*J* = 1.7 Hz, 1H, ArH-6), 7.00 (dd, ^3^*J* = 8.4 Hz, ^4^*J* = 1.0 Hz, 1H, H-3), 6.98 (ddd, ^3^*J* = 7.8 Hz, ^3^*J* = 7.2 Hz, ^4^*J* = 1.0 Hz, 1H, H-5), 2.26 (s, 3H, Me), 1.40 (s, 9H, *t*-Bu) ppm; ^13^C NMR (100 MHz, DMSO-d_6_): δ 164.15 (C=O), 158.70 (C-2), 154.76 (ArC-2-OH), 151.50 (CH=N), 136.14 (ArC-3), 133.94 (C-4), 129.68 (ArC-4), 129.30 (ArC-6), 128.61 (C-6), 127.02 (ArC-5), 119.02 (C-5), 117.20 (C-3, ArC-1), 115.61 (C-1), 34.33 (C—*t*-Bu), 29.17 (3 × CH_3_—*t*-Bu), 20.18 (CH_3_) ppm; HRMS (TOF, MS, ESI): *m*/*z* for C_19_H_22_N_2_O_3_ + H^+^ calculated: 327.1703; found: 327.1701.

**3,5-Dihydroxy-*N*’-[(*E*)-(3-*tert*-butyl-2-hydroxy-5-methylphenyl)methylidene]benzohydrazide** (**4f**) 

The general procedure starting from 3-*tert*-butyl-5-methyl-salicylic aldehyde (**8**) (192 mg, 1.0 mmol) [108], 3,5-dihydroxybenzoic acid hydrazide (**17**) (168 mg, 1.0 mmol), CH_3_OH (5.0 mL), and AcOH (100 µL) was employed with a 2 h reaction time, to obtain colorless powder of 3,5-dihydroxy-*N*’-[(*E*)-(3-*tert*-butyl-2-hydroxy-5-methylphenyl)methylidene]benzohydrazide (**4f**) (285 mg, 0.83 mmol) with 83% yield, which melts at 246 °C (CH_3_OH) with decompositions. Selected FT-IR (ATR): ν_max_ 3189 (br., N-H), 3046 (C-H), 1643 (C=O), 1583 (N-H, C=C), 1556 (C=N), 1435, 1363, 1341, 1314, 1265 (br., C_Ar_-O), 1208, 1164, 1001, 863, 798, 681, 518, 427 cm^−1^; ^1^H NMR (400 MHz, DMSO-*d*_6_): δ 12.25 (s, 1H, Ar-2-OH), 12.04 (s, 1H, CONH), 9.63 (s, 2H, 3-OH, 5-OH), 8.50 (s, 1H, CH=N), 7.09 (d, ^4^*J* = 1.4 Hz, 1H, ArH-4), 7.03 (d, ^4^*J* = 1.4 Hz, 1H, ArH-6), 6.78 (d, ^4^*J* = 2.1 Hz, 2H, H-2,6), 6.45 (t, ^4^*J* = 2.1 Hz, 1H, H-4), 2.25 (s, 3H, Me), 1.39 (s, 9H, *t*-Bu) ppm; ^13^C NMR (100 MHz, DMSO-*d*_6_): δ 162.86 (C=O), 158.46 (C-3, C-5), 154.71 (ArC-2-OH), 150.54 (CH=N), 136.09 (ArC-3), 134.52 (C-1), 129.41 (ArC-4), 129.15 (ArC-6), 126.95 (ArC-5), 117.33 (ArC-1), 105.90 (C-4), 105.71 (C-2, C-6), 34.34 (C—*t*-Bu), 29.19 (3 × CH_3_—*t*-Bu), 20.19 (Me) ppm; HRMS (TOF, MS, ESI): *m*/*z* for C_19_H_22_N_2_O_4_ + H^+^ calculated: 343.1652; found: 343.1650. 

**2-Hydroxy-*N*’-[(*E*)-(3,5-di-*tert*-butyl-2-hydroxyphenyl)methylidene]benzohydrazide** (**4g**) 

The general procedure starting from 3,5-di-*tert*-butyl-salicylic aldehyde (**9**) (234 mg, 1.0 mmol) [108], 2-hydroxybenzoic acid hydrazide (**16**) (152 mg, 1.0 mmol), CH_3_OH (3.8 mL), and AcOH (100 µL) was employed with a 4 h reaction time, to obtain 2-hydroxy-*N*’-[(*E*)-(3,5-di-*tert*-butyl-2-hydroxyphenyl)methylidene]benzohydrazide (**4g**) (359 mg, 0.97 mmol) with 97% yield as colorless powder, which melts at 233–235 °C (CH_3_OH) (m.p. 218–222 °C [89]), (m.p. 230–232 °C [90]). Selected FT-IR (ATR): ν_max_ 3238 (br., N-H, O-H), 3078 (C-H), 1636 (C=O), 1611 (C=C), 1584 (N-H), 1554 (C=N), 1486, 1461, 1436, 1359, 1329, 1302, 1269, 1247 (C_Ar_-O), 1215, 1173, 825, 770, 745, 714, 665, 534, 505, 428 cm^−1^; ^1^H NMR (600 MHz, DMSO-*d*_6_): δ 12.20 (s, 1H, Ar-2-OH), 12.08 (s, br, 1H, NHCO or 2-OH), 11.67 (s, br, 1H, NHCO or 2-OH), 8.63 (s, 1H, CH=N), 7.89 (dd, ^3^*J* = 7.8 Hz, ^4^*J* = 1.5 Hz, 1H, H-6), 7.46 (ddd, ^3^*J* = 8.3 Hz, *J* = 7.7 Hz, ^4^*J* = 1.5 Hz, 1H, H-4), 7.32 (d, ^4^*J* = 2.3 Hz, 1H, ArH-4), 7.25 (d, ^4^*J* = 2.3 Hz, 1H, ArH-6), 7.01 (d, ^3^*J* = 8.3 Hz, 1H, H-3), 6.98 (dd, ^3^*J* = 7.8 Hz, ^3^*J* = 7.4 Hz, 1H, H-5), 1.41 (s, 9H, Ar-3-*t*-Bu), 1.28 (s, 9H, Ar-5-*t*-Bu) ppm; ^1^H NMR (400 MHz, DMSO-*d*_6_): δ 12.21 (s, 1H, Ar-2-OH), 12.08 (s, br, 1H, NHCO or 2-OH), 11.67 (s, br, 1H, NHCO or 2-OH), 8.63 (s, 1H, CH=N), 7.89 (d, ^3^*J* = 7.4 Hz, 1H, H-6), 7.46 (dd, ^3^*J* = 7.5 Hz, ^3^*J* = 7.3 Hz, 1H, H-4), 7.32 (d, ^4^*J* = 1.7 Hz, 1H, ArH-4), 7.25 (d, ^4^*J* = 1.7 Hz, 1H, ArH-6), 7.01 (d, ^3^*J* = 7.3 Hz, 1H, H-3), 6.98 (dd, ^3^*J* = 7.5 Hz, ^3^*J* = 7.4 Hz, 1H, H-5), 1.41 (s, 9H, Ar-3-*t*-Bu), 1.28 (s, 9H, Ar-5-*t*-Bu) ppm; ^13^C NMR (100 MHz, DMSO-*d*_6_): δ 163.96 (C=O), 158.51 (C-2), 154.70 (ArC-2-OH), 151.92 (CH=N), 140.39 (ArC-5), 135.62 (ArC-3), 133.90 (C-4), 128.79 (C-6), 125.85 (ArC-6), 125.70 (ArC-4), 119.08 (C-5), 117.18 (C-3), 116.86 (ArC-1), 115.79 (C-1), 34.59 (C—ArC-3-*t*-Bu), 33.82 (C—ArC-5-*t*-Bu), 31.22 (3 × CH_3_—ArC-5-*t*-Bu), 29.22 (3 × CH_3_—ArC-3-*t*-Bu) ppm; HRMS (TOF, MS, ESI) *m*/*z* for C_22_H_28_N_2_O_3_ + H^+^: calculated 369.2173; found: 369.2186.

**3-Hydroxy-*N*’-[(*E*)-(3,5-di-*tert*-butyl-2-hydroxyphenyl)methylidene]benzohydrazide** (**4h**) 

The general procedure starting from 3,5-di-*tert*-butyl-salicylic aldehyde (**9**) (234 mg, 1.0 mmol) [108], 3-hydroxybenzoic acid hydrazide (**18**) (152 mg, 1.0 mmol), CH_3_OH (5.0 mL), and AcOH (100 µL) was employed with a 4 h reaction time, to obtain 3-hydroxy-*N*’-[(*E*)-(3,5-di-*tert*-butyl-2-hydroxyphenyl)methylidene]benzohydrazide (**4h**) (359 mg, 0.97 mmol) with 97% yield as white wool, which melts at 273–274 °C (CH_3_OH). Selected FT-IR (ATR): ν_max_ 3201 (br., N-H, O-H), 3064 (C-H), 1638 (C=O), 1611 (N-H), 1567 (C=N), 1480, 1435, 1358, 1318, 1302, 1270, 1251 (C_Ar_-O), 1201, 1172, 1133, 1086, 832, 769, 695, 683, 642, 587, 533, 441 cm^−1^; ^1^H NMR (600 MHz, DMSO-*d*_6_): δ 12.31 (s, 1H, Ar-2-OH), 12.12 (s, 1H, NHCO), 9.83 (s, 1H, 3-OH), 8.57 (s, 1H, CH=N), 7.36 (d, ^3^*J* = 8.1 Hz, 1H, H-6), 7.34 (dd, ^3^*J* = 8.1 Hz, ^3^*J* = 7.3 Hz, 1H, H-5), 7.33 (s, 1H, H-2), 7.31 (d, ^4^*J* = 2.3 Hz, 1H, ArH-4), 7.20 (d, ^4^*J* = 2.3 Hz, 1H, ArH-6), 7.01 (ddd, ^3^*J* = 7.3 Hz, ^4^*J* = 2.1 Hz, ^4^*J* = 2.1 Hz, 1H, H-4), 1.41 (s, 9H, Ar-3-*t*-Bu), 1.28 (s, 9H, Ar-5-*t*-Bu) ppm; ^13^C NMR (100 MHz, DMSO-*d*_6_): δ 162.76 (C=O), 157.44 (C-3-OH), 154.67 (ArC-2-OH), 151.10 (CH=N), 140.32 (ArC-5), 135.58 (ArC-3), 133.93 (C-1), 129.61 (C-5), 125.66 (ArC-4 or ArC-6), 125.47 (ArC-4 or ArC-6), 118.97 (C-4), 118.07 (C-6), 116.94 (ArC-1), 114.45 (C-2), 34.59 (C—ArC-3-*t*-Bu), 33.82 (C—ArC-5-*t*-Bu), 31.23 (3 × CH_3_—ArC-5-*t*-Bu), 29.23 (3 × CH_3_—ArC-3-*t*-Bu) ppm; HRMS (TOF, MS, ESI) *m*/*z* for C_22_H_28_N_2_O_3_ + H^+^: calculated 369.2173; found: 369.2183. 

**3,5-Dihydroxy-*N*’-[(*E*)-(3,5-di-*tert*-butyl-2-hydroxyphenyl)methylidene]benzohydrazide** (**4i**) 

The general procedure starting from 3,5-di-*tert*-butyl-salicylic aldehyde (**9**) (117 mg, 0.5 mmol) [108], 3,5-dihydroxybenzoic acid hydrazide (**17**) (84 mg, 0.5 mmol), CH_3_OH (3.0 mL), and AcOH (50 µL) was employed with a 2 h reaction time, to obtain 3,5-dihydroxy-*N*’-[(*E*)-(3,5-di-*tert*-butyl-2-hydroxyphenyl)methylidene]benzohydrazide (**4i**) (190 mg, 0.494 mmol) with 99% yield as colorless powder, which melts at 266 °C (CH_3_OH) with decompositions. Selected FT-IR (ATR): ν_max_ 3228 (br., N-H), 3076 (C_Ar_-H), 1629 (C=C), 1599 (C=O), 1583 (N-H), 1549 (C=N), 1464, 1435, 1358, 1300, 1269, 1249, 1234 (C_Ar_-O), 1172, 1162, 1138, 1007, 855, 810, 751, 714, 609, 542, 514, 433 cm^−1^; ^1^H NMR (400 MHz, DMSO-*d*_6_): δ 12.32 (s, 1H, Ar-2-OH), 12.05 (s, 1H, CONH), 9.65 (s, 2H, 3-OH, 5-OH), 8.55 (s, 1H, CH=N), 7.30 (d, ^4^*J* = 2.2 Hz, 1H, ArH-4), 7.18 (d, ^4^*J* = 2.2 Hz, 1H, ArH-6), 6.78 (d, ^4^*J* = 2.0 Hz, 2H, H-2,6), 6.45 (t, ^4^*J* = 2.0 Hz, 1H, H-4), 1.41 (s, 9H, Ar-3-*t*-Bu), 1.28 (s, 9H, Ar-5-*t*-Bu) ppm; ^13^C NMR (100 MHz, DMSO-*d*_6_): δ 162.96 (C=O), 158.48 (C-3, C-5), 154.69 (ArC-2-OH), 150.98 (CH=N), 140.33 (ArC-5), 135.60 (ArC-3), 134.58 (C-1), 125.66 (ArC-4), 126.46 (ArC-6), 116.99 (ArC-1), 105.92 (C-4), 105.75 (C-2, C-6), 34.62 (C—3-*t*-Bu), 33.85 (C—5-*t*-Bu), 31.27 (3 × CH_3_—5-*t*-Bu), 29.25 (3 × CH_3_—3-*t*-Bu) ppm; HRMS (TOF, MS, ESI): *m*/*z* for C_22_H_28_N_2_O_4_ + H^+^ calculated: 385.2122; found: 385.2120;HRMS (TOF, MS, ESI): *m*/*z* for C_22_H_28_N_2_O_4_ + Na^+^ calculated: 407.1941; found: 407.1948. 

**2-Hydroxy*-N*’-[(*E*)-(3-*tert*-butyl-2-hydroxyphenyl)methylidene]benzohydrazide** (**4j**) 

The general procedure starting from 3-*tert*-butyl-salicylic aldehyde (**7**) (190 mg, 1.0 mmol) [108], 2-hydroxybenzohydrazide (**16**) (152 mg, 1.0 mmol), CH_3_OH (3.0 mL), and AcOH (100 µL) was employed with a 3.5 h reaction time and crystallization in open vessel to obtain pale 2-hydroxy-*N*’-[(*E*)-(3-*tert*-butyl-2-hydroxyphenyl)methylidene]benzohydrazide (**4j**) (306 mg, 0.98 mmol) as a with 98% yield, which melts at 218–220 °C. Selected FT-IR (ATR): ν_max_ 3248 (br., N-H), 3064 (C_Ar_-H), 1630 (C=O), 1610 (N-H), 1583 (C=C), 1552 (C=N), 1455, 1429 (C-N), 1356, 1305, 1261, 1231 (C_Ar_-O), 1198 1158, 1146, 1100, 1086, 942, 902, 852, 798, 737, 691, 662, 590, 565, 524, 422 cm^−1^; ^1^H NMR (600 MHz, DMSO-*d*_6_): δ 12.40 (s, 1H, ArOH), 12.12 (s, 1H), 11.66 (s, 1H), 8.62 (s, 1H, CH=N), 7.88 (d, ^3^*J* = 7.9 Hz, 1H, H-6), 7.46 (dd, ^3^*J* = 7.9 Hz, ^3^*J* = 7.5 Hz, 1H, H-4), 7.31 (d, ^3^*J* = 7.6 Hz, 1H, ArH-4), 7.30 (d, ^3^*J* = 7.6 Hz, 1H, ArH-6), 7.01 (d, ^3^*J* = 7.9 Hz, 1H, H-3), 6.98 (dd, ^3^*J* = 7.9 Hz, ^3^*J* = 7.5 Hz, 1H, H-5), 6.89 (dd, ^3^*J* = 7.6 Hz, ^3^*J* = 7.6 Hz, 1H, ArH-5), 1.41 (s, 9H, *t*-Bu) ppm; ^1^H NMR (400 MHz, DMSO-*d*_6_): δ 12.41 (s, 1H, ArOH), 12.13 (s, 1H), 11.68 (s, 1H), 8.63 (s, 1H, CH=N), 7.89 (dd, ^3^*J* = 7.9 Hz, ^4^*J* = 1.3 Hz, 1H, H-6), 7.46 (ddd, ^3^*J* = 7.7 Hz, ^3^*J* = 7.5 Hz, 1H, H-4), 7.30 (d, ^3^*J* = 7.7 Hz, 1H, ArH-4), 7.30 (d, ^3^*J* = 7.6 Hz, 1H, ArH-6), 7.01 (d, ^3^*J* = 7.7 Hz, 1H, H-3), 6.98 (dd, ^3^*J* = 7.9 Hz, ^3^*J* = 7.5 Hz, 1H, H-5), 6.89 (dd, ^3^*J* = 7.7 Hz, ^3^*J* = 7.6 Hz, 1H, ArH-5), 1.41 (s, 9H, *t*-Bu) ppm; ^13^C NMR (100 MHz, DMSO-*d*_6_): δ 164.14 (C=O), 158.69 (C-2), 156.95 (ArC-2-OH), 151.52 (CH=N), 136.34 (ArC-3), 133.97 (C-4), 129.61 (ArC-6), 128.67 (C-6, ArC-4), 119.06 (C-5), 118.75 (ArC-5), 117.53 (ArC-1), 117.22 (C-3), 115.61 (C-1), 34.43 (C—*t*-Bu), 29.15 (3 × CH_3_—*t*-Bu) ppm; HRMS (TOF, MS, ESI): *m*/*z* for C_18_H_20_N_2_O_3_ + H^+^ calculated: 313.1547; found: 313.1558. 

**3-Hydroxy*-N*’-[(*E*)-(3-*tert*-butyl-2-hydroxyphenyl)methylidene]benzohydrazide** (**4k**) 

The general procedure starting from 3-*tert*-butyl-salicylic aldehyde (**7**) (95 mg, 0.50 mmol) [108], 3-hydroxybenzoic acid hydrazide (**18**) (76 mg, 0.50 mmol), CH_3_OH (3.0 mL), and AcOH (100 µL) was employed with a 3.0 h reaction time and crystallized with addition of water (0.7 mL) before decolorization with charcoal to obtain the 3-hydroxy-*N*’-[(*E*)-(3-*tert*-butyl-2-hydroxyphenyl)methylidene]benzohydrazide (**4k**) (142 mg, 0.455 mmol) as a pale solid with 91% yield, which melts at 225–228 °C. Selected FT-IR (ATR): ν_max_ 3227 (br., N-H), 3068 (C_Ar_-H), 1648 (C=O), 1590 (N-H, C=C), 1567 (C=N), 1456, 1429, 1362, 1320, 1259, 1240 (C_Ar_-O), 1197, 1143, 1084, 846, 745, 701, 685, 595, 494 cm^−1^; ^1^H NMR (600 MHz, DMSO-*d*_6_): δ 12.49 (s, 1H, Ar-2-OH), 12.15 (s, 1H, NHCO), 9.83 (s, 1H, 3-OH), 8.57 (s, 1H, CH=N), 7.38 (d, *J* = 7.6 Hz, 1H, H-6), 7.35 (dd, ^3^*J* = 7.6 Hz, ^3^*J* = 7.6 Hz, 1H, H-5), 7.34 (s, 1H, H-2), 7.29 (dd, ^3^*J* = 7.7 Hz, ^4^*J* = 1.1 Hz, 1H, ArH-4), 7.27 (dd, ^3^*J* = 7.7 Hz, ^4^*J* = 1.1 Hz, 1H, ArH-6), 7.01 (ddd, ^3^*J* = 7.6 Hz, ^4^*J* = 2.3 Hz, ^4^*J* = 1.4 Hz, 1H, H-4), 6.88 (dd, ^3^*J* = 7.7 Hz, ^3^*J* = 7.6 Hz, 1H, ArH-5), 1.41 (s, 9H, *t*-Bu) ppm; ^13^C NMR (100 MHz, DMSO-*d*_6_): δ 162.73 (C=O), 157.46 (C-3-OH), 156.90 (ArC-2-OH), 150.68 (CH=N), 136.30 (ArC-3), 133.84 (C-1), 129.63 (C-5), 129.46 (ArC-6), 128.44 (ArC-4), 119.02 (C-4), 118.69 (ArC-5), 118.06 (C-6), 117.62 (ArC-1), 114.46 (C-2), 34.43 (C—*t*-Bu), 29.17 (3 × CH_3_—*t*-Bu) ppm; HRMS (TOF, MS, ESI): *m*/*z* for C_18_H_20_N_2_O_3_ + H^+^ calculated: 313.1547; found: 313.1547. 

**3,5-Dihydroxy*-N*’-[(*E*)-(3-*tert*-butyl-2-hydroxyphenyl)methylidene]benzohydrazide** (**4l**) 

The general procedure starting from 3-*tert*-butyl-salicylic aldehyde (**7**) (95 mg, 0.50 mmol) [108] 3,5-dihydroxybenzoic acid hydrazide (**17**) (84 mg, 0.50 mmol), CH_3_OH (3.0 mL), and AcOH (50 µL) was employed with a 3.0 h reaction time and crystallized with addition of water (1.0 mL) before concentration at reduced pressure to obtain the 3,5-dihydroxy-*N*’-[(*E*)-(3-*tert*-butyl-2-hydroxyphenyl)methylidene]benzohydrazide (**4l**) (138 mg, 0.42 mmol) as a pale solid with 84% yield, which melts at 217–220 °C. Selected FT-IR (ATR): ν_max_ 3208 (br., N-H), 3070 (C_Ar_-H), 1591 (C=O, N-H), 1557 (C=N), 1429, 1337, 1302, 1262 (br., C_Ar_-O), 1200, 1162, 1003, 806, 792, 746, 674, 623, 557, 521, 471, 441 cm^−1^; ^1^H NMR (400 MHz, DMSO-*d*_6_): δ 12.46 (s, 1H, Ar-2-OH), 12.03 (s, 1H, NHCO), 9.61 (s, 2H, 3-OH, 5-OH), 8.51 (s, 1H, CH=N), 7.25 (dd, ^3^*J* = 7.7 Hz, ^4^*J* = 1.3 Hz, 1H, ArH-4), 7.21 (dd, ^3^*J* = 7.7 Hz, ^4^*J* = 1.3 Hz, 1H, ArH-6), 6.83 (dd, ^3^*J* = 7.7 Hz, ^3^*J* = 7.7 Hz, 1H, ArH-5), 6.74 (d, ^4^*J* = 2.1 Hz, 2H, H-2,6), 6.42 (t, ^4^*J* = 2.1 Hz, 1H, H-4), 1.36 (s, 9H, *t*-Bu) ppm; ^13^C NMR (100 MHz, DMSO-*d*_6_): δ 162.93 (C=O), 158.50 (C-3-OH, C-5-OH), 156.92 (ArC-2-OH), 150.58 (CH=N), 136.33 (ArC-3), 134.49 (C-1), 129.45 (ArC-6), 128.43 (ArC-4), 118.71 (ArC-5), 117.67 (ArC-1), 105.98 (C-4), 105.75 (C-2, C-6), 34.46 (C—*t*-Bu), 29.19 (3 × CH_3_—*t*-Bu) ppm; HRMS (TOF, MS, ESI): *m*/*z* for C_18_H_20_N_2_O_4_ + H^+^ calculated: 329.1496; found: 329.1503. 

**2-Hydroxy*-N*’-[(*E*)-(2-hydroxy-3-phenyl-phenyl)methylidene]benzohydrazide** (**4m**) 

The general procedure starting from 3-phenyl-salicylic aldehyde (**6**) (149 mg, 0.75 mmol) [108], 2-hydroxybenzohydrazide (**16**) (114 mg, 0.75 mmol), CH_3_OH (30 mL), and AcOH (75 µL) was employed with a 3.5 h reaction time and crystallization with concentration to obtain a white solid the 2-hydroxy-*N*’-[(*E*)-(2-hydroxy-3-phenyl-phenyl)methylidene]benzohydrazide (**4m**) (206 mg, 0.62 mmol) with 83% yield, which melts in 224–227 °C. Selected FT-IR (ATR): ν_max_ 3231 (br., N-H), 3100-3000 (br., C_Ar_-H), 1601 (C=O), 1580 (N-H), 1559 (C=N), 1453, 1436, 1371, 1307, 1231 (br., C_Ar_-O), 1153, 1071, 892, 747, 696, 664, 625, 582, 568, 532, 469 cm^−1^; ^1^H NMR (600 MHz, DMSO-*d*_6_): δ 12.26 (s, 1H, Ar-2-OH), 12.18 (s, 1H, OH or NH), 11.65 (s, 1H, OH or NH), 8.71 (s, 1H, CH=N), 7.88 (dd, ^3^*J* = 7.9 Hz, ^4^*J* = 1.6 Hz, 1H, H-6), 7.61 (dd, ^3^*J* = 8.1 Hz, ^4^*J* = 1.2 Hz, 2H, PhH-2,6), 7.48 (dd, ^3^*J* = 7.6 Hz, ^4^*J* = 1.5 Hz, 1H, ArH-6), 7.47 (ddd, ^3^*J* = 8.3 Hz, ^3^*J* = 7.2 Hz, ^4^*J* = 1.6 Hz, 1H, H-4), 7.45 (ddd, ^3^*J* = 8.1 Hz, ^3^*J* = 7.4 Hz, 2H, PhH-3,5), 7.41 (dd, ^3^*J* = 7.5 Hz, ^4^*J* = 1.5 Hz, 1H, ArH-4), 7.36 (tt, ^3^*J* = 7.4 Hz, ^4^*J* = 1.2 Hz, 1H, PhH-4), 7.06 (dd, ^3^*J* = 7.6 Hz, ^3^*J* = 7.5 Hz, 1H, ArH-5), 7.01 (dd, ^3^*J* = 8.3 Hz, ^4^*J* = 1.0 Hz, 1H, H-3), 6.98 (ddd, ^3^*J* = 7.9 Hz, ^3^*J* = 7.2 Hz, ^4^*J* = 1.0 Hz, 1H, H-5) ppm; ^13^C NMR (100 MHz, DMSO-*d*_6_): δ 164.19 (C=O), 158.65 (C-2-OH), 154.96 (ArC-2-OH), 150.75 (CH=N), 137.36 (PhC-1), 134.31 (C-4), 132.38 (ArC-4), 130.66 (ArC-6), 129.15 (PhC-2, PhC-6), 128.82 (ArC-3), 128.75 (C-6), 128.00 (PhC-3, PhC-5), 126.99 (PhC-4), 119.53 (ArC-5), 119.08 (C-5), 118.09 (ArC-1), 117.21 (C-3), 115.65 (C-1) ppm; HRMS (TOF, MS, ESI): *m*/*z* for C_20_H_16_N_2_O_3_ + H^+^ calculated: 333.1234; found: 333.1237.

**3-Hydroxy*-N*’-[(*E*)-(2-hydroxy-3-phenyl-phenyl)methylidene]benzohydrazide** (**4n**) 

The general procedure starting from 3-phenyl-salicylic aldehyde (**6**) (99 mg, 0.50 mmol) [108], 3-hydroxybenzohydrazide (**18**) (76 mg, 0.50 mmol), CH_3_OH (3.0 mL), and AcOH (50 µL) was employed with a 2.0 h reaction time to obtain the 3-hydroxy-*N*’-[(*E*)-(2-hydroxy-3-phenyl-phenyl)methylidene]benzohydrazide (**4n**) (157 mg, 0.47 mmol) as a white solid with 94% yield, which melts at 207–209 °C. Selected FT-IR (ATR): ν_max_ 3227 (br., N-H), 3056 (br., C_Ar_-H), 1651 (C=O), 1592 (N-H), 1527 (C=N), 1448, 1428, 1352, 1284, 1227 (br., C_Ar_-O), 1137, 1071, 830, 755, 706, 691, 680, 642, 580, 549, 534 cm^−1^; ^1^H NMR (600 MHz, DMSO-*d*_6_): δ 12.37 (s, 1H, Ar-2-OH), 12.23 (s, 1H, NHCO), 9.84 (s, 1H, 3-OH), 8.65 (s, 1H, CH=N), 7.60 (d, ^3^*J* = 7.3 Hz, 2H, PhH-2,6), 7.45 (d, ^3^*J* = 7.6 Hz, 1H, ArH-6), 7.44 (dd, ^3^*J* = 7.9 Hz, ^3^*J* =7.3 Hz, 2H, PhH-3.5), 7.40 (dd, ^3^*J* = 7.6 Hz, ^4^*J* = 1.4 Hz, 1H, ArH-4), 7.37 (t, ^3^*J* = 7.9 Hz, 1H, PhH-4), 7.32–7.37 (m, 3H, H-2, H-5, H-6), 7.04 (dd, ^3^*J* = 7.6 Hz, ^3^*J* = 7.6 Hz, 1H, ArH-5), 7.02 (ddd, ^3^*J* = 7.8 Hz, ^4^*J* = 2.3 Hz, ^4^*J* = 1.2 Hz, 1H, H-4) ppm; ^13^C NMR (100 MHz, DMSO-*d*_6_): δ 162.79 (C=O), 157.47 (C-3), 154.94 (ArC-2-OH), 149.93 (CH=N), 137.42 (PhC-1), 133.79 (C-1), 132.18 (ArC-4), 130.54 (ArC-6), 129.65 (C-5), 129.16 (PhC-2, PhC-6), 128.77 (ArC-3), 128.01 (PhC-3, PhC-5), 126.98 (PhC-4), 119.47 (ArC-5), 119.07 (C-4), 118.17 (ArC-1), 118.11 (C-6), 114.49 (C-2) ppm; HRMS (TOF, MS, ESI): *m*/*z* for C_20_H_16_N_2_O_3_ + H^+^ calculated: 333.1234; found: 333.1241. 

**3,5-Dihydroxy*-N*’-[(*E*)-(2-hydroxy-3-phenyl-phenyl)methylidene]benzohydrazide** (**4o**) 

The general procedure starting from 3-phenyl-salicylic aldehyde (**6**) (99 mg, 0.50 mmol) [108], 3,5-dihydroxybenzohydrazide (**17**) (84 mg, 0.50 mmol), CH_3_OH (3.0 mL), and AcOH (50 µL) was employed with a 4.0 h reaction time to obtain the 3,5-dihydroxy-*N*’-[(*E*)-(2-hydroxy-3-phenyl-phenyl)methylidene]benzohydrazide (**4o**) (159 mg, 0.456 mmol) as a white solid with 91% yield, which melts at 241–243 °C. Selected FT-IR (ATR): ν_max_ 3234 (br., N-H), 3056 (br., C_Ar_-H), 1655 (C=O), 1592 (N-H), 1544 (C=N), 1449, 1428, 1337, 1301, 1277, 1254 (br., C_Ar_–O), 1159, 1141, 1000, 800, 754, 696, 676, 680, 642, 580, 549, 534 cm^−1^; ^1^H NMR (600 MHz, DMSO-*d*_6_): δ 12.38 (s, 1H, Ar-2-OH), 12.14 (s, 1H, NHCO), 9.65 (s, 2H, 3-OH, 5-OH), 8.63 (s, 1H, CH=N), 7.60 (d, ^3^*J* = 7.2 Hz, 2H, PhH-2,6), 7.44 (dd, ^3^*J* = 7.4 Hz, ^3^*J* = 7.2 Hz, 2H, PhH-3.5), 7.43 (dd, ^3^*J* = 7.7 Hz, ^4^*J* = 1.3 Hz, 1H, ArH), 7.39 (dd, ^3^*J* = 7.5 Hz, ^4^*J* = 1.3 Hz, 1H, ArH), 7.35 (t, ^3^*J* = 7.4 Hz, 1H, PhH-4), 7.04 (dd, ^3^*J* =7.7 Hz, ^3^*J* = 7.5 Hz, 1H, ArH-5), 6.79 (d, ^4^*J* = 2.1 Hz, 2H, H-2,6), 6.46 (t, ^4^*J* = 2.1 Hz, 1H, H-4) ppm; ^13^C NMR (100 MHz, DMSO-*d*_6_): δ 162.97 (C=O), 158.51 (C-3, C-5), 154.95 (ArC-2-OH), 149.83 (CH=N), 137.45 (PhC-1), 134.44 (C-1), 132.16 (ArC-4), 130.54 (ArC-6), 129.19 (PhC-2, PhC-6), 128.78 (ArC-3), 128.04 (PhC-3, PhC-5), 127.01 (PhC-4), 119.49 (ArC-5), 118.21 (ArC-1), 106.02 (C-4), 105.78 (C-2, C-6) ppm; HRMS (TOF, MS, ESI): *m*/*z* for C_20_H_16_N_2_O_4_ + H^+^ calculated: 349.1183; found: 349.1197. 

***N*’-[(*E*)-(3-*tert*-butyl-2-hydroxy-5-methylphenyl)methylidene]-2-(1-hydroxynaphtho)hydrazide** (**5a**) 

The general procedure starting from 3-*tert*-butyl-5-methyl-salicylic aldehyde (**8**) (192 mg, 1.0 mmol) [108], 1-hydroxy-2-naphthohydrazide (**19**) (202 mg, 1.0 mmol) [109], CH_3_OH (65 mL), and AcOH (100 µL) was employed with a 6 h reaction time and concentrated by slow distillation before crystallization to obtain the colorless prisms of *N*’-[(*E*)-(3-*tert*-butyl-2-hydroxy-5-methylphenyl)methylidene]-2-(1-hydroxynaphtho)hydrazide (**5a**) (345 mg, 0.916 mmol) with 92% yield, which melts at 215–217 °C. Selected FT-IR (ATR): ν_max_ 3379 (O-H), 3233 (N-H), 3062 (C_Ar_-H), 1616 (C=O), 1581 (N-H), 1532 (C=N), 1467, 1388, 1356, 1310, 1275, 1250 (C_Ar_-O), 1207 (C_Ar_-O), 1175, 1155, 813, 788, 757, 707, 566, 496, 423 cm^−1^; ^1^H NMR (400 MHz, DMSO-*d*_6_): δ 13.85 (s, 1H, OH), 12.50 (s, 1H, OH), 12.19 (s, 1H, NHCO), 8.65 (s, 1H, CH=N), 8.32 (d, ^3^*J* = 8.2 Hz, 1H, H-4), 7.97 (d, ^3^*J* = 8.9 Hz, 1H, H-8), 7.92 (d, ^3^*J* = 8.2 Hz, 1H, H-3), 7.69 (dd, ^3^*J* = 8.8 Hz, ^3^*J* = 6.1 Hz, 1H, H-6), 7.60 (dd, ^3^*J* = 8.9 Hz, ^3^*J* = 6.1 Hz, 1H, H-7), 7.49 (d, ^3^*J* = 8.9 Hz, 1H, H-5), 7.14 (s, 2H, ArH-4, ArH-6), 2.27 (s, 3H, CH_3_), 1.42 (s, 9H, *t*-Bu) ppm; ^13^C NMR (100 MHz, DMSO-*d*_6_): δ 166.58 (C=O), 156.15 (C), 154.88 (C), 152.27 (CH=N), 136.23 (C), 135.97 (C), 129.94 (CH), 129.50 (CH), 129.34 (CH), 127.51 (C), 126.08 (CH), 124.52 (C), 123.04 (CH), 122.22 (CH), 118.05 (CH), 117.14 (C), 115.77 (C), 34.36 (C), 29.19 (3 × CH_3_—*t*-Bu), 20.18 (CH_3_) ppm; HRMS (TOF, MS, ESI) *m*/*z* for C_23_H_24_N_2_O_3_ + H^+^: calculated 377.1860; found: 377.1858. 

***N*’-[(*E*)-(3-*tert*-butyl-2-hydroxy-3-phenyl-phenyl)methylidene]-2-(1-hydroxynaphtho)hydrazide** (**5b**) 

The general procedure starting from 3-phenyl-salicylic aldehyde (**9**) (198 mg, 1.0 mmol) [108], 1-hydroxy-2-naphthohydrazide (**19**) (202 mg, 1.0 mmol) [109], CH_3_OH (100 mL), and AcOH (100 µL) was employed with a 3 h reaction time to obtain the *N*’-[(*E*)-(2-hydroxy-3-phenylphenyl)methylidene]-2-(1-hydroxynaphtho)hydrazide (**5b**) (313 mg, 0.819 mmol) as pale powders with 82% yield, which melts at 226–228 °C. Selected FT-IR (ATR): ν_max_ 3373 (O-H), 3100–3000 (N-H, C_Ar_-H), 1628 (C=O), 1600 (N-H), 1573 (C=C), 1532 (C=N), 1461, 1389, 1274, 1249 (C_Ar_-O), 1206 (C_Ar_-O), 1158, 1075, 968, 789, 754, 738, 693, 573, 491, 429, 411 cm^−1^; ^1^H NMR (400 MHz, DMSO-*d*_6_): δ 13.83 (s, 1H, OH), 12.59 (s, 1H, OH), 12.27 (s, 1H, NHCO), 8.77 (s, 1H, CH=N), 8.32 (d, ^3^*J* = 8.2 Hz, 1H, H-8), 7.98 (d, ^3^*J* = 8.9 Hz, 1H, H-3), 7.92 (d, ^3^*J* = 8.1 Hz, 1H, H-5), 7.69 (ddd, ^3^*J* = 6.8 Hz, ^3^*J* = 6.8 Hz, ^4^*J* = 1.2 Hz, 1H, H-6), 7.62 (d, ^3^*J* = 7.7 Hz, 2H, PhH-2,6), 7.60 (ddd, ^3^*J* = 8.2 Hz, ^3^*J* = 6.8 Hz, ^4^*J* = 1.1 Hz, 1H, H-7), 7.53 (dd, ^3^*J* = 7.7 Hz, ^4^*J* = 1.4 Hz, 1H, ArH-6), 7.49 (d, ^3^*J* = 8.9 Hz, H-4), 7.46 (dd, ^3^*J* = 7.7 Hz, ^3^*J* = 7.3 Hz, 2H, PhH-3,5), 7.43 (dd, ^3^*J* = 7.5 Hz, ^4^*J* = 1.4 Hz, 1H, ArH-4), 7.36 (t, ^3^*J* = 7.3 Hz, 1H, PhH-4), 7.07 (t, ^3^*J* = 7.7 Hz, ^3^*J* = 7.5 Hz, 1H, ArH-5) ppm; ^13^C NMR (100 MHz, DMSO-*d*_6_): δ 166.71 (C=O), 160.21 (C), 154.04 (C), 151.45 (CH=N), 137.29 (C), 135.99 (C), 132.59 (CH), 130.85 (CH), 129.36 (CH), 129.16 (2 × CH), 128.89 (C), 128.01 (2 × CH), 127.50 (CH), 127.02 (CH), 126.07 (CH), 124.51 (C), 123.03 (CH), 122.18 (CH), 119.59 (CH), 118.08 (CH), 118.01 (C), 105.72 (C) ppm; HRMS (TOF, MS, ESI) *m*/*z* for C_24_H_18_N_2_O_3_ + H^+^: calculated 383.1390; found: 383.1410. 

***N*’-[(*E*)-(3,5-di-*tert*-butyl-2-hydroxy-phenyl)methylidene]-2-(1-hydroxynaphtho)hydrazide monomethanolate** (**5c**) 

The general procedure starting from 3,5-di-*tert*-butyl-salicylic aldehyde (**9**) (234 mg, 1.0 mmol) [108], 1-hydroxy-2-naphthohydrazide (**19**) (202 mg, 1.0 mmol) [109], CH_3_OH (75 mL), and AcOH (100 µL) was employed with a 2 h reaction time, decolorization with charcoal and concentration slow by distillation before crystallization to obtain *N*’-[(*E*)-(3,5-di-*tert*-butyl-2-hydroxy-phenyl)methylidene]-2-(1-hydroksynaphtho)hydrazide (**5c**) (397 mg, 0.881 mmol) as yellow needles with 88% yield crystallized with one molecule of methyl alcohol, which melts at 122–123 °C. Selected FT-IR (ATR): ν_max_ 3650 (CH_3_OH), 3392 (O-H), 3322 (N-H), 3060 (C_Ar_-H), 1611 (C=O), 1596 (C=C), 1584 (N-H), 1563 (C=N), 1531, 1467, 1390, 1356, 1278, 1250 (C_Ar_-O), 1207 (C_Ar_-O), 1173, 1027, 815, 759, 709, 535, 494, 419 cm^−1^; ^1^H NMR (400 MHz, DMSO-*d*_6_): δ 13.85 (s, 1H, O-H), 12.50 (s, 1H, NHCO), 12.24 (s, 1H, O-H), 8.70 (s, 1H, CH=N), 8.32 (d, ^3^*J* = 8.2 Hz, 1H, H-8), 7.98 (d, ^3^*J* = 8.8 Hz, 1H, H-3), 7.92 (d, ^3^*J* = 8.0 Hz, 1H, H-5), 7.69 (dd, ^3^*J* = 8.0 Hz, ^3^*J* = 7.2 Hz, 1H, H-6), 7.60 (dd, ^3^*J* = 8.2 Hz, ^3^*J* = 7.2 Hz, 1H, H-7), 7.50 (d, ^3^*J* = 8.8 Hz, 1H, H-4), 7.35 (d, ^4^*J* = 2.0 Hz, 1H, ArH-4), 7.28 (d, ^4^*J* = 2.0 Hz, 1H, ArH-6), 4.10 (s, 1H, CH_3_OH), 3.17 (s, 3H, CH_3_OH), 1.43 (s, 9H, Ar-3-*t*-Bu), 1.30 (s, 9H, Ar-5-*t*-Bu) ppm; ^13^C NMR (100 MHz, DMSO-*d*_6_): δ 166.59 (C), 160.14 (C=O), 154.83 (C), 152.72 (CH=N), 140.52 (C), 135.97 (C), 135.73 (C), 129.34 (CH), 127.51 (CH), 126.08 (CH), 126.01 (CH), 125.97 (CH), 124.53 (C), 123.04 (CH), 122.25 (CH), 118.06 (CH), 116.78 (C), 105.79 (C), 48.52 (CH_3_OH), 34.62 (C), 33.85 (C), 31.22 (3 × CH_3_—*t*-Bu), 29.23 (3 × CH_3_—*t*-Bu) ppm; HRMS (TOF, MS, ESI) *m*/*z* for C_26_H_30_N_2_O_3_ + Na^+^: calculated 441.2149; found: 441.2152.

***N*’-[(*E*)-(3-*tert*-butyl-2-hydroxy-phenyl)methylidene]-2-(1-hydroxynaphtho)hydrazide** (**5d**) 

The general procedure starting from 3-*tert*-butyl-salicylic aldehyde (**7**) (89 mg, 0.50 mmol) [108], 1-hydroxy-2-naphthohydrazide (**19**) (101 mg, 0.50 mmol) [109], CH_3_OH (22.5 mL), and AcOH (50 µL) was employed with a 2 h reaction time, decolorization with charcoal and concentration slow by distillation before crystallization to obtain *N*’-[(*E*)-(3-*tert*-butyl-2-hydroxy-phenyl)methylidene]-2-(1-hydroxynaphtho)hydrazide (**5d**) (180 mg, 0.497 mmol) as yellow crystals with 99% yield, which melts at 220–222 °C. Selected FT-IR (ATR): ν_max_ 3420 (O-H), 3275 (N-H), 3056 (C_Ar_-H), 2948 (CH_3_), 1623 (C=O), 1602 (N-H, C=C), 1565 (C=N), 1389, 1358, 1279, 1252 (C_Ar_-O), 1208 (C_Ar_-O), 1147, 807, 789, 745, 495, 420 cm^−1^; ^1^H NMR (400 MHz, DMSO-*d*_6_): δ 13.84 (s, 1H, OH), 12.51 (s, 1H, NHCO), 12.41 (s, 1H, OH), 8.70 (s, 1H, CH=N), 8.32 (d, ^3^*J* = 8.3 Hz, 1H, H-8), 7.98 (d, ^3^*J* = 8.9 Hz, 1H, H-3), 7.92 (d, ^3^*J* = 8.1 Hz, 1H, H-5), 7.69 (dd, ^3^*J* = 8.1 Hz, ^3^*J* = 6.9 Hz, ^4^*J* = 1.2 Hz, 1H, H-6), 7.60 (dd, ^3^*J* = 8.3 Hz, ^3^*J* = 6.7 Hz, ^4^*J* = 1.2 Hz, 1H, H-7), 7.49 (d, ^3^*J* = 8.9 Hz, 1H, H-4), 7.34 (d, ^3^*J* = 7.6 Hz, 1H, ArH-6), 7.33 (d, ^3^*J* = 7.8 Hz, 1H, ArH-4), 6.91 (dd, ^3^*J* = 7.8 Hz, ^3^*J* = 7.6 Hz, 1H, ArH-5), 1.42 (s, 9H, *t*-Bu) ppm; ^13^C NMR (100 MHz, DMSO-*d*_6_): δ 166.61 (C-1), 160.18 (C=O), 157.05 (ArC-2-OH), 152.27 (CH=N), 136.42 (C), 135.98 (C), 129.84 (CH), 129.34 (CH), 128.90 (CH), 127.50 (CH), 126.07 (CH), 124.53 (C), 123.05 (CH), 122.19 (CH), 118.84 (CH), 118.07 (CH), 117.45 (C), 105.74 (C), 34.45 (C), 29.16 (3 × CH_3_) ppm; HRMS (TOF, MS, ESI) *m*/*z* for C_22_H_22_N_2_O_3_ + Na^+^: calculated 385.1523; found: 385.1535.

### 3.2. Reaction Condition and Kinetic Calculations

Reaction conditions and kinetic assays were performed as reported previously [25]. Briefly, the kinetic measurements were conducted in pH 5.3 at 25 ℃. A reaction was carried out in a plastique cuvette with a total capacity of 2.0 mL. It contained 1.5 mL of a diluted in McIlvain buffer enzyme (10–50 nM), 0.1 mL of inhibitor (1 × 10^3^ µM), and 0.1 mL of syringaldazine (0.5–15 µM). Hydrazide-hydrazones, sodium azide, and syringaldazine were dissolved in methanol as co-solvent before use. Before reaction, the enzyme was preincubated with an inhibitor for 30 min. The progress of the reaction was measured at 525 nm (Shimadzu, UV-VIS 1800, Tokyo, Japan) using UVProbe 2.70 software. Each reaction was performed at least in triplicate. The velocity of the syringaldazine conversion was expressed as a change in product concentration in time (µM/min) and calculated from the region of a first-order kinetic using an extinction coefficient ε_0_ = 65,000 cm^−1^ [110]. The kinetic parameters for all tested compounds were calculated by using Lineweaver–Burk transformation and the linear least square method in Matlab (The Mathworks, R2019R Version 9.6, academic license) using steady-state kinetics. The goodness of fit was assessed using the coefficient of determination (R^2^), the sum of square errors (SSE), and root mean squared error (RMSE). The following equations were applied for the determination of the competitive Equation (1), non-competitive Equation (2), uncompetitive Equation (3), and mixed Equation (4) types of inhibition [99]. Mixed type of inhibition Equation (4) was determined based the Segel’s mathematical model where *K*_I_ component corresponds to the competitive component, whereas α·*K*_I_ is a non-competitive or uncompetitive component for α < 1 or α > 1, respectively. The resulted from inhibition changes in V_max_ [µM·min^−1^] and *K*_M_ [µM] parameters were expressed as their apparent counterparts V_max,app_ [µM·min^−1^] and *K*_M,app_ [µM]. The constant inhibition values (*K*_I_) were determined by replotting fixed apparent values versus inhibitor concentrations into a linear function (Figure 2 and Figure 3).

1/V = (*K*_M,app_/V_max_)·(1/C_s_) + 1/V_max_ (1)

where *K*_M,app_ is defined as *K*_M_·(1 + I/*K*_I_)

1/V = (*K*_M_/V_max,app_)·(1/C_s_) + 1/V_max,app_ (2)

where V_max,app_ is defined as (1 + I/*K*_I_)/V_max_

1/V = (*K*_M_/V_max_)·(1/C_s_) + 1/V_max,app_ (3)

where V_max,app_ is defined as (1 + I/*K*_I_)/V_max_ or 1/*K*_M,app_ = (1/*K*_M_)·(1 + I/*K*_I_)

1/V = (*K*_M,app_/V_max_)·(1/C_s_) + 1/V_max,app_ (4)

where *K*_M,app_ is defined as *K*_M_·(1 + I/*K*_I_), and V_max,app_ defined as (1 + I/*α*·*K*_I_)/V_max_, where *K*_M_—a Michaelis–Menten constant [µM], K_M,app_—an apparent Michaelis–Menten constant [µM], V_max_—a maximal enzyme velocity [µM·min^−1^], V_max,app_—an apparent maximal enzyme velocity [µM·min^−1^], C_s_—a substrate (syringaldazine) concentration, I—an inhibitor concentration [µM], *K*_I_—an inhibition constant [µM], α·*K*_I_ is a non-competitive or uncompetitive component for *α* < 1 or *α* > 1 in the mixed type of inhibition, respectively.

## 4. Conclusions

In this work, we aimed to investigate the inhibition activity of hydrazide-hydrazones towards laccase from *Trametes versicolor*. The hydrazide-hydrazones were synthesized using four salicylic aldehydes of choice and eleven hydrazides to obtain 21 derivatives, of which 20 have not been described in the literature so far. The most active compounds have the constant inhibition in the range of 8 and 25.8 µM and present diversified types of inhibition, but none of them interact with the enzyme-substrate complex. Kinetic results revealed that the presence of an arene electron-rich benzoyl fragment containing hydroxy and methoxy group in 3 and 4 positions of the benzene ring is necessary to inhibit laccase activity effectively. Performed for selected derivatives, the structure–activity relationship analysis showed that the slice differences in the built of the inhibitors might reflect the inhibition type. We evaluated the contribution of the selected amino acids from the substrate center with the possibility of changing the type of inhibition. The deep and substrate-like binding is preferred to interact competitively, but the mixed systems with uncommon interactions resulted in lower *K*_I_ values. The found hydrazide-hydrazones with the highest activity towards laccase will be the subject of further research on SAR and future application to protect cultivating plans against phytopathogenic fungi [107]. 

## Figures and Tables

**Table 1:** Characteristics of the hydrazide-hydrazones **3a**–**b**, **4a**–**o**, and **5a**–**d**.

No.	Ar	R	Yield, (%)	M_p_, (°C)	C=O ^a,b^	N–H ^b,c^	C=N ^b^	C_Ar_-O ^b^
**3a**	3-*t*Bu-2-HO-5-MeC_6_H_2_	Me	84	231–233	1654	1614	1565	1239
**3b**	3-*t*Bu-2-HO-5-MeC_6_H_2_	4-HOC_6_H_4_CH_2_	85	242–244	1654	1612	1552	1261
**4a**	3-*t*Bu-2-HO-5-MeC_6_H_2_	3-pyridyl	93	230–232	1638	1590	1552	1261
**4b**	3-*t*Bu-2-HO-5-MeC_6_H_2_	C_6_H_5_	96	246–248	1635	1612	1561	1264
**4c**	3-*t*Bu-2-HO-5-MeC_6_H_2_	3-MeOC_6_H_4_	93	238–240	1633	1584	1560	1241
**4d**	3-*t*Bu-2-HO-5-MeC_6_H_2_	4-MeOC_6_H_4_	81	243–244	1636	1605	1537	1235
**4e**	3-*t*Bu-2-HO-5-MeC_6_H_2_	2-HOC_6_H_4_	95	252–254	1637	1615	1560	1235
**4f**	3-*t*Bu-2-HO-5-MeC_6_H_2_	3,5-(HO)_2_C_6_H_3_	83	246 ^d^	1643	1583	1556	1265
**4g**	3,5-(*t*Bu)_2_-2-HOC_6_H_2_	2-HOC_6_H_4_	97	233–235 ^e^	1636	1584	1554	1247
**4h**	3,5-(*t*Bu)_2_-2-HOC_6_H_2_	3-HOC_6_H_4_	98	273–274	1638	1611	1567	1251
**4i**	3,5-(*t*Bu)_2_-2-HOC_6_H_2_	3,5-(HO)_2_C_6_H_3_	99	266 ^d^	1599	1583	1549	1234
**4j**	3-*t*Bu-2-HOC_6_H_3_	2-HOC_6_H_4_	98	218–220	1630	1610	1552	1231
**4k**	3-*t*Bu-2-HOC_6_H_3_	3-HOC_6_H_4_	90	225–228	1648	1590	1567	1240
**4l**	3-*t*Bu-2-HOC_6_H_3_	3,5-(HO)_2_C_6_H_3_	84	217–220	1591	1591	1557	1262
**4m**	2-HO-3-PhC_6_H_3_	2-HOC_6_H_4_	83	224–227	1601	1580	1559	1231
**4n**	2-HO-3-PhC_6_H_3_	3-HOC_6_H_4_	94	207–209	1651	1592	1527	1227
**4o**	2-HO-3-PhC_6_H_3_	3,5-(HO)_2_C_6_H_3_	91	241–243	1655	1592	1544	1254
**5a**	3-*t*Bu-2-HO-5-MeC_6_H_2_	2-(1-hydroxynaphthyl)	92	215–217	1616	1581	1532	1250
**5b**	2-HO-3-PhC_6_H_3_	2-(1-hydroxynaphthyl)	82	226–228	1628	1600	1532	1249
**5c**	3,5-(*t*Bu)_2_-2-HOC_6_H_2_	2-(1-hydroxynaphthyl)	95 ^f^	121–123	1611	1584	1563	1250
**5d**	3-*t*Bu-2-HOC_6_H_3_	2-(1-hydroxynaphthyl)	99	220–222	1623	1602	1565	1252

^a^ Amide I bond vibrations; ^b^ in cm^−1^; ^c^ amide II bond vibrations; ^d^ Melts with decomposition; ^e^ Literature melting point: 218–222 °C [95], 230–232 °C [96]; ^f^ Crystallize with one molecule of CH_3_OH.

**Table 2:** Structures and inhibition constant (*K*_I_, µM) values of the hydrazide-hydrazones **3a**–**b**, **4a**–**o**, **5a**–**d** determined for laccase (*T. versicolor*). Crucial *K*_I_ values were bolded. The reference compounds are **20**–**23** and NaN_3_ [31].

No.	Ar	R	*K*_I_, µM	R^2^	Inhibition Type
**3a**	3-*t*Bu-2-HO-5-MeC_6_H_2_	Me	– ^a^	–	– ^b^
**3b**	3-*t*Bu-2-HO-5-MeC_6_H_2_	4-HOC_6_H_4_CH_2_	49.2	0.960	uncompetitive
**4a**	3-*t*Bu-2-HO-5-MeC_6_H_2_	3-pyridyl	– ^c^	–	– ^b^
**4b**	3-*t*Bu-2-HO-5-MeC_6_H_2_	C_6_H_5_	82.0	0.996	competitive
**4c**	3-*t*Bu-2-HO-5-MeC_6_H_2_	3-MeOC_6_H_4_	**17.4**	0.974	non-competitive
**4d**	3-*t*Bu-2-HO-5-MeC_6_H_2_	4-MeOC_6_H_4_	**25.8**	0.984	competitive
**4e**	3-*t*Bu-2-HO-5-MeC_6_H_2_	2-HOC_6_H_4_	150.0	0.958	uncompetitive
**4f**	3-*t*Bu-2-HO-5-MeC_6_H_2_	3,5-(HO)_2_C_6_H_3_	**32.3**	0.982	competitive
**4g**	3,5-(*t*Bu)_2_-2-HOC_6_H_2_	2-HOC_6_H_4_	52.6	0.998	non-competitive
**4h**	3,5-(*t*Bu)_2_-2-HOC_6_H_2_	3-HOC_6_H_4_	**18.9**	0.996	competitive
**4i**	3,5-(*t*Bu)_2_-2-HOC_6_H_2_	3,5-(HO)_2_C_6_H_3_	55.6	0.961	competitive
**4j**	3-*t*Bu-2-HOC_6_H_3_	2-HOC_6_H_4_	65.6	0.965	uncompetitive
**4k**	3-*t*Bu-2-HOC_6_H_3_	3-HOC_6_H_4_	**35.8**	0.994	competitive
**4l**	3-*t*Bu-2-HOC_6_H_3_	3,5-(HO)_2_C_6_H_3_	**38.0**	0.923	competitive
**4m**	2-HO-3-PhC_6_H_3_	2-HOC_6_H_4_	26.4	0.982	competitive
			57.1	0.959	uncompetitive
**4n**	2-HO-3-PhC_6_H_3_	3-HOC_6_H_4_	233	0.976	competitive
			139	0.964	non-competitive
**4o**	2-HO-3-PhC_6_H_3_	3,5-(HO)_2_C_6_H_3_	69.9	0.977	non-competitive
**5a**	3-*t*Bu-2-HO-5-MeC_6_H_2_	2-(1-hydroxynaphthyl)	**19.0**	0.993	competitive
			**8.0**	0.982	non-competitive
**5b**	2-HO-3-PhC_6_H_3_	2-(1-hydroxynaphthyl)	**25.8**	0.950	competitive
			**24.3**	0.999	non-competitive
**5c**	3,5-(*t*Bu)_2_-2-HOC_6_H_2_	2-(1-hydroxynaphthyl)	**16.2**	0.998	non-competitive
**5d**	3-*t*Bu-2-HOC_6_H_3_	2-(1-hydroxynaphthyl)	75.2	0.971	competitive
			55.6	0.999	non-competitive
**20 ^d^**	3-*t*Bu-2-HO-5-MeC_6_H_2_	4-HOC_6_H_4_	26.4 [31]	–	competitive
**21 ^d^**	3,5-(*t*Bu)_2_-2-HOC_6_H_2_	4-HOC_6_H_4_	17.9 [31]	–	uncompetitive
**22 ^d^**	3-*t*Bu-2-HO-5-MeC_6_H_2_	3-HOC_6_H_4_	32.0 [31]	–	non-competitive
**23 ^d^**	2-HO-3-PhC_6_H_3_	4-MeOC_6_H_4_	≥1000 [31]	–	– ^b^
**–**	NaN_3_^d^		2.7 [31]		non-competitive

^a^ Not active up to 1000 µM of concentration tested, ^b^ not determined, ^c^ insoluble in medium, *K*_I_—an inhibition constant, determined for a related type of inhibition according to reference [99], ^d^ controls [31].

## Data Availability

Not applicable.

## Supplementary Materials

It is available online at https://www.mdpi.com/article/10.3390/ijms222212307/s1.
