# Supplementary material for: Inhibitory Potential of New Phenolic Hydrazide-Hydrazones with a Decoy Substrate Fragment towards Laccase from a Phytopathogenic Fungus: SAR and Molecular Docking Studies
Source: Int J Mol Sci. 2021 Nov 14;22(22):12307. doi: 10.3390/ijms222212307 (PMC8617976; doi:10.3390/ijms222212307)
Supplement: Supplementary file 1 [file ijms-22-12307-s001.zip › ijms-1463750-supplementary.pdf]

SUPPORTING INFORMATION

Inhibitory potential of new phenolic hydrazide-hydrazones with a decoy substrate fragment towards laccase from a phytopathogenic fungus: SAR and molecular docking studies

Halina Maniak [1,*], Michał Talma [2] and Mirosław Giurg [3,*]

[1] Department of Micro, Nano and Bioprocess Engineering, Faculty of Chemistry, Wroclaw University of Science and Technology, Wybrzeże Wyspiańskiego 27, Wrocław 50-370, Poland (H.M.);

[2] Department of Bioorganic Chemistry, Faculty of Chemistry, Wroclaw University of Science and Technology, Wybrzeże Wyspiańskiego 27, Wrocław 50-370, Poland; <michal.talma@pwr.edu.pl> (M.T.);

[3] Department of Organic and Medicinal Chemistry, Faculty of Chemistry, Wroclaw University of Science and Technology, Wybrzeże Wyspiańskiego 27, Wrocław 50-370, Poland (M.G.)

* Correspondence:

Tel.: +48-71-320-3314; fax: +48-71-328-1318/e-mail: halina.maniak@pwr.edu.pl (H.M.);

Tel.: +48-71-320-3616/ e-mail: miroslaw.giurg@pwr.edu.pl (M.G.)

List of contents

1. Preparation of benzoic acid methyl esters **2c–d** and **2f** in the presence of $SOCl_2$ — 2
2. Preparation of acid methyl esters **2e**, **2g–i** in the presence of H_2SO_4 additive — 2–3
3. Preparation of 3-phenyl-salicylic aldehyde (**6**) — 3
4. Preparation of 3-*tert*-butyl-5-methyl-salicylic aldehyde (**8**) — 3
5. Synthesis of benzoic acid hydrazides **11–19** — 4–6
6. Selected ^{1}H, ^{13}C NMR and FT-IR spectra, DEPT-135 and 2D experiments of used compounds — 6–188
7. References — 189–190

1. Preparation of benzoic acid methyl esters **2c–d** and **2f** in the presence of $SOCl_2$

3-Methoxy-benzoic acid methyl esters (2c)
To a solution of 3-methoxybenzoic acid (**1c**) (15.1g, 100 mmol) in MeOH (150 mL) $SOCl_2$ (1.1 mL, 15 mmol) was added dropwise during vigorous stirring and the reaction was gently refluxed by 20 hours. Solvent was distilled off under 20 mmHg and reevaporated before adding toluene (2 × 50 mL) to vanish the rest of the $SOCl_2$. The residue was dissolved in $CHCl_3$ and filtered up by pad of silica gel (70–230 mesh) eluted with $CHCl_3$ to obtain 3-methoxybenzoic acid methyl ester (**2c**) (12.6g, 76 mmol) with 76% yield, which boils at 223–225 °C (b.p. 109.0–109.5 °C at 6 mmHg [1] [^{1}H-NMR (400 MHz, $CDCl_3$) δ 7.64 (ddd, ^3J = 7.6 Hz, ^4J = 1.5 Hz, ^5J = 1.0 Hz, 1H, H-6), 7.56 (dd, ^4J = 2.7 Hz, ^4J = 1.5 Hz, 1H, H-2), 7.34 (ddd, ^3J = 8.3 Hz, ^3J = 7.6 Hz, ^5J = 0.4 Hz, 1H, H-5), 7.10 (ddd, ^3J = 8.3 Hz, ^4J = 2.7 Hz, ^4J = 1.0 Hz, 1H, H-4), 3.92 (s, 3H, OCH_3), 3.85 (s, 3H, OCH_3) ppm. ^{1}H NMR characteristic is consistent with literature values [2].

4-Methoxybenzoic acid methyl ester (2d)
The literature procedure was applied using 4-methoxybenzoic acid (**1d**) (15.2g, 100 mmol) dissolved in MeOH (150 mL) [3]. To the solution $SOCl_2$ (1.1 mL, 15 mmol) was added dropwise during vigorous stirring and the reaction was gently refluxed by 60 hours. The solvent was evaporated and reevaporated twice before toluene (2 × 60 mL) adding to vanish rest of $SOCl_2$, and was filtered up by silica gel (70–230 mesh) eluted with $CHCl_3$ to obtain 4-methoxybenzoic acid methyl ester (**2d**) (15.6g, 94 mmol) with 94% yield, which melts at 48–50 °C (from CH_3OH) (m.p. 48–49 °C [1]).

3-Hydroxybenzoic acid methyl ester (2f)

The literature procedure was applied using 3-hydroxybenzoic acid (**1f**) (13.8g, 100 mmol) in MeOH (150 mL) [3]. At ice-water bath the $SOCl_2$ (5.0 mL, 69 mmol) was added dropwise during vigorous stirring and stored by 20 hours at room temperature. The reaction mixture was distilled off under 20 mmHg and evaporated twice before adding toluene (2 × 75 mL) to vanish the rest of the $SOCl_2$, dissolved at $CHCl_3$ (300 mL) and filtered up by a pad of silica gel (70–230 mesh) eluted by $CHCl_3$ to obtain 3-hydroxybenzoic acid methyl ester (**2c**) (13.1g, 86 mmol) with 86% yield, which melts at 70–71 °C (m.p. 69–71 °C [4]).

2. Preparation of acid methyl esters **2e**, **2g–i** in the presence of H_2SO_4 additive

General procedure: the synthesis procedure was adapted from the literature [5]. To a solution of carboxylic acid (0.05–0.10 mmol) in dry methanol (50–200 mL], H_2SO_4 (1.3–2.0 mL) was added dropwise during vigorous stirring and the reaction was gently refluxed by 16–60 h. The reaction mixture was concentrated under reduced pressure, the residue was dissolved in DEE, and washed hardly with ice water (2 × 50 ml). The organic solution was washed with saturated $NaHCO_3$, with brine, and dried with anhydrous Na_2SO_4. The solvent was distilled off from water bath to obtain carboxylic acid methyl ester.

Synthesis of salicylic acid methyl ester (2e)

The general procedure starting from salicylic acid (**1e**) (6.91g, 50 mmol), MeOH (100 mL), and H_2SO_4 (1.0 mL, 19 mmol) was employed with a 20 hours reaction time using DEE (300 mL), to obtain crude product (6.09g, 40 mmol) with 80% yield and the product was distilled under reduced pressure to obtain transparent oil of pure salicylic acid methyl ester (**2e**) (5.80g, 38 mmol) with 76% yield, which boils in 222–223 °C (221–222 °C [6]**)**. ^{1}H-NMR (300 MHz, $CDCl_3$) δ 10.74 (s, 1H, OH), 7.81 (ddd, ^3J = 8.0 Hz, ^4J = 1.8 Hz, 1H, H-6), 7.44 (ddd, ^3J = 8.4 Hz, ^3J = 7.2 Hz, ^4J = 1.8 Hz, 1H, H-4), 6.96 (dd, ^3J = 8.4 Hz, ^4J = 1.1 Hz, 1H, H-3), 6.86 (ddd, ^3J = 8.0 Hz, ^3J = 7.2 Hz, ^4J = 1.0 Hz, 1H, H-5), 3.94 (s, 3H, OCH_3) ppm. ^{1}H NMR characteristic is consistent with literature values [7].
The connected $NaHCO_3$ solution was acidified with hydrochloric acid to pH ca 2.5 and extracted with DEE (150 + 100 mL), extract dried with anhydrous Na_2SO_4, and solvent was distilled off from water bath to obtain recovered salicylic acid (**1e**) (1.4g, 10 mmol) with 20% yield, which melts in 155–157 °C (m.p. 158 °C [8]).

Benzoic acid methyl ester (2g)

The general procedure starting from benzoic acid (**1g**) (6.11g, 50 mmol), MeOH (50 mL), H_2SO_4 (2.0 mL, 38 mmol) was employed with a 16 h using DEE (100 mL) to obtain crude benzoic acid methyl ester (**2g**) (6.06g, 44.5 mmol) with 89% yield. The analytical sample was obtained by distillation under 20 mmHg to obtain transparent oil of benzoic acid methyl ester (**2g**) (5.58g, 41 mmol) with 82% yield, which boils in 81–82 °C at 20 mmHg (b.p. 83–85 °C in 20 mmHg) [9]. ^{1}H-NMR (300 MHz, $CDCl_3$): δ 8.05 (dd, ^3J = 7.6 Hz, ^4J = 1.3 Hz, 2H, H-2,6), 7.56 (tt, ^3J = 7.4 Hz, ^4J = 1.3 Hz, 1H, H-4), 7.44 (dd, ^3J = 7.6 Hz, ^3J = 7.4 Hz, 2H, H-3,5), 3.92 (s, 3H, OCH_3) ppm. A spectrum image of ^{1}H-NMR is consistent with literature reference (Supporting information, Figure S93 [10]).

1-Hydroxy-2-naphthoic acid methyl ester (2h)

The general procedure starting from 1-hydroxy-2-naphthoic acid (**1h**) (18.8g, 100 mmol), MeOH (200 mL), H_2SO_4 (2.0 mL, 38 mmol) was employed with a 60 h using DEE (200 mL) to obtain 1-hydroxy-2-naphthoic acid methyl ester (**2h**) (10.9g, 54 mmol) with 54% yield, which melts in 76–78 °C (m.p. 76–78 °C [10]). ^{1}H-NMR (300 MHz, $CDCl_3$): δ 11.98 (s, 1H, OH), 8.41 (ddd, ^3J = 8.2 Hz, ^4J = 1.4 Hz, ^5J = 0.8 Hz, 1H, ArH), 7.73 (d, ^3J = 8.2 Hz, 1H, ArH), 7.73 (d, ^3J = 8.9 Hz, 1H, ArH), 7.58 (ddd, ^3J = 8.2 Hz, ^3J = 6.8 Hz, ^4J = 1.4 Hz, 1H, ArH), 7.50 (ddd, ^3J = 8.2 Hz, ^3J = 6.8 Hz, ^4J = 1.3 Hz, 1H, ArH), 7.25 (d, ^3J = 8.9 Hz, 1H, ArH), 3.96 (s, 3H, OCH_3) ppm; ^{13}C-NMR (75 MHz, $CDCl_3$): δ 171.36 (C=O), 160.86 (C), 137.13 (C), 129.31 (CH), 127.38 (CH), 125.67 (CH), 124.69 (C), 124.16 (CH), 123.81 (CH), 118.53 (CH), 105.52 (C), 52.21 (OCH_3) ppm. The characteristics of NMR spectra are consistent with literature value [10].
The connected $NaHCO_3$ solution was acidified with hydrochloric acid to pH ca 2.5 and extracted with DEE (150 mL), extract dried with anhydrous Na_2SO_4, and solvent was distilled off from water bath to obtain recovered 1-hydroxy-2-naphthoic acid (**1h**) (8.6g, 46 mmol) with 46% yield, which melts in 198–199 °C (m.p. 198.8°C [11]), which was reused for esterification. ^{1}H-NMR (300 MHz, $CDCl_3$): δ 11.69 (s, 1H, OH), 8.45 (d, ^3J = 8.5 Hz, 1H, ArH), 7.84 (d, ^3J = 8.8 Hz, 1H, ArH), 7.80 (d, ^3J = 8.1 Hz, 1H, ArH), 7.65 (ddd, ^3J = 8.1 Hz, ^3J = 6.9 Hz, ^4J = 1.1 Hz, 1H, ArH), 7.56 (ddd, ^3J = 8.9 Hz, ^3J = 6.9 Hz, ^4J = 0.9 Hz, 1H, ArH), 7.34 (d, ^3J = 8.8 Hz, 1H, ArH) ppm. A spectrum image of ^{1}H-NMR is consistent with literature spectrum recorded at acetone-d_6 [12].

3,5-Dihydroxy-benzoic acid methyl ester (2i)

The general procedure starting from 3,5-dihydroxy-benzoic acid (**1i**) (7.71g, 50 mmol), MeOH (130 mL), and H_2SO_4 (1.3 mL, 24.7 mmol) was employed with a 60 h reaction time using DEE (150 mL) to obtain pale fine crystalline powder of 3,5-dihydroxy-benzoic acid methyl ester (**2i**) (8.16g, 48.5 mmol) with 97% yield, which melts in 168.5–170.5 °C (DEE) (m.p. 165.0–168.5 °C [13]). ^{1}H-NMR (400 MHz, $CDCl_3$): δ 7.08 (d, ^4J = 2.3 Hz, 2H, ArH-2,6), 6.56 (t, ^4J = 2.3 Hz, 1H, ArH-4), 5.20 (s, 2H, OH), 3.89 (s, 3H, OCH_3) ppm. The characteristic of ^{1}H NMR spectra are consistent with literature value [10].

3. Preparation of 3-phenyl-salicylic aldehyde (**6**)

The salicylic aldehyde **6** was obtained from 2-hydroxybiphenyl via 2-(methoxymethoxy)-1,1′-biphenyl regiospecific methalation step by LICTMEDA and formylation of the formed intermediate with DMF according to the literature procedure [14] with 87% yield, which melts at 47.5–48.5 °C (m.p. 47–48 °C [15]). FT-IR, ^{1}H-NMR, and ^{13}C-NMR data were consistent with literature values [3,16].

4. Preparation of 3-*tert*-butyl-5-methyl-salicylic aldehyde (**8**)

The salicylic aldehyde **8** was prepared by 2-*tert*-butyl-4-methyl-phenol formylation with paraformaldehyde applied the literature procedure [14] with 86% yield, which melts at 77–78 °C (m.p. 74–75 °C [17]). The ^{1}H-NMR, and ^{13}C-NMR data are consistent with the literature values [18], and [3], respectively.

5. Synthesis of benzoic acid hydrazides **11–19**

General procedure: the synthesis procedure was adapted from the literature [19]. To a solution of carboxylic acid methyl ester (1.0 eq.) in dry methanol, hydrazine monohydrate (98%, 1.0–1.5 eq.) was added. The reaction mixture was stirred under gentle reflux for two days. The reaction mixture was left to crystallization to obtain the appropriate hydrazides **11–19**.

2-(4-Hydroxyphenyl)acetic acid hydrazide (11)

The general procedure starting from 2-(4-hydroxyphenyl)-acetic acid methyl ester (**2b**) (3.32g, 20 mmol), hydrazine monohydrate (1.50 mL, 31 mmol) at methanol (20 mL) was employed with gentle reflux at 18 h, to obtain colorless powder of 2-(4-hydroxyphenyl)acetic acid hydrazide (**11**) (3.00g, 18.1 mmol) with 90% yield, which melts at 204–205 °C (from CH_3OH) (m.p. 200–202 °C [20]). Selected FT-IR (ATR): ν_{max} 3307 (N-H), 3021 (br., C_{Ar}-H), 2809 (CH_2), 1644 (C=O), 1609 (C=C), 1598 (N-H), 1509, 1456, 1438, 1382, 1344, 1263, 1249 (C_{Ar}-O), 1198, 1171, 1099, 1034, 964, 809, 745, 711, 664, 607, 522, 472, 424 cm^{-1}; ^{1}H-NMR (DMSO-d_6, 400 MHz): δ 9.21 (s, 1H, OH), 9.12 (s, 1H, NH), 7.04 (d, ^{3}J = 8.3 Hz, 2H, H-2,6), 6.67 (d, ^{3}J = 8.3 Hz, 2H, H-3,5), 4.18 (s, 2H, NH_2), 3.21 (s, 2H, CH_2); ^{13}C-NMR (DMSO-d_6, 100 MHz): δ 170.12 (C=O), 155.86 (C-4), 129.83 (C-2, C-6), 126.34 (C-1), 114.93 (C-3, C-5), 39.60 (CH_2) ppm; HRMS (TOF, MS, ESI) m/z for $C_8H_{10}N_2O_2$ + H^+: calculated 167.0815, found: 167.0815. ^{1}H-NMR and ^{13}C-NMR data are consistent with literature values [21].

Nicotinic acid hydrazide (12)

The general procedure starting from nicotynic acid methyl ester (**2a**) (6.86g, 50 mmol), hydrazine monohydrate (2.67 mL, 55 mmol) in methanol (25 mL) was employed at gentle reflux at 38 h to obtain colorless powder of nicotinic acid hydrazide (**12**) (5.86g, 42.7 mmol) with 85% yield, which melt at 165–167 °C (from CH_3OH) (m.p. 164–167 °C [22]). Selected FT-IR (ATR): ν_{max} 3322 (N-H), 3199 (NH_2), 3000 (br., C_{Ar}-H), 1673 (C=O), 1645 (C=C), 1594 (N-H), 1541 (C=C, C=N), 1474, 1422, 1338, 1190, 1113, 1032, 951, 881, 832, 704, 680, 628, 525, 420 cm^{-1}; ^{1}H-NMR (300 MHz, DMSO-d_6): δ 9.98 (s, 1H, NH), 8.97 (dd, ^{3}J = 2.2 Hz, ^{4}J = 0.8 Hz, 1H, H-2), 8.69 (dd, ^{3}J = 4.8 Hz, ^{4}J =1.7 Hz, 1H, H-6), 8.15 (ddd, ^{3}J =8.0 Hz, ^{4}J = 2.2 Hz, ^{4}J = 1.7 Hz, 1H, H-4), 7.48 (ddd, ^{3}J = 8.0 Hz, ^{4}J = 4.8 Hz, ^{5}J = 0.8 Hz, 1H, H-5), 4.59 (s, 2H, NH_2) ppm; ^{1}H-NMR (400 MHz, DMSO-d_6): δ 9.96 (s, 1H, NH), 8.97 (dd, ^{4}J = 2.3 Hz, ^{5}J = 0.8 Hz, 1H, H-2), 8.68 (dd, ^{3}J = 4.8 Hz, ^{4}J = 1.7 Hz, 1H, H-6), 8.15 (ddd, ^{3}J = 7.9 Hz, ^{4}J = 2.3 Hz, ^{4}J = 1.7 Hz, 1H, H-4), 7.48 (ddd, ^{3}J = 7.9 Hz, ^{3}J = 4.8 Hz, ^{5}J = 0.8 Hz, 1H, H-5), 4.57 (s, 2H, NH_2) ppm; ^{13}C-NMR (DMSO-d_6, 100 MHz): δ 164.29 (C=O), 151.70 (C-H), 148.02 (C-H), 134.61 (C-H), 128.83 (C), 123.43 (C-H) ppm; HRMS (TOF, MS, ESI) m/z for $C_6H_7N_3O$ + H^+: calculated 138.0662, found: 138.0668. ^{1}H-NMR and ^{13}C-NMR data are generally consistent with literature values [23].

Benzoic acid hydrazide (13)

The general procedure starting from benzoic acid methyl ester (**2g**) (1.36g, 10 mmol), hydrazine monohydrate (0.53 mL, 0.55g, 11 mmol) at boiling methanol (6.0 mL) was employed with obtain colorless powder of benzoic acid hydrazide (**13**) (1.16 g, 8.5 mmol) with 85% yield, which melts at 114–117 °C (from CH_3OH) (m.p. 114–115 °C [23]). Selected FT-IR (ATR): ν_{max} 3298 (N-H), 3195 (NH_2), 3000 (C_{Ar}-H), 1659 (C=O), 1604 (N-H), 1557 (C=C), 1486, 1446, 1343, 1298, 1117, 1003, 985, 919, 882, 801, 682, 672, 666, 516 cm^{-1} [24]; ^{1}H-NMR (DMSO-d_6, 400 MHz): δ 9.77 (s, 1H, NH), 7.82 (ddd, J = 7.5 Hz, J = 2.2 Hz, J = 1.4 Hz, 2H, H-2,6), 7.50 (tt, J = 7.3 Hz, J = 1.4 Hz, 1H, H-4), 7.44 (dddd, J = 7.5 Hz, J = 7.3 Hz, J = 2.2 Hz, J = 1.4 Hz, 2H, H-3,5), 4.50 (s, 2H, NH_2) ppm; ^{13}C-NMR (DMSO-d_6, 100 MHz): δ 165.84 (C=O), 133.23 (C), 130.99 (C-H), 128.24 (2 × C-H), 126.88 (2 × C-H) ppm; HRMS (TOF, MS, ESI) m/z for $C_7H_8N_2O$ + H^+: calculated 137.0709, found: 137.0714. ^{1}H-NMR and ^{13}C-NMR data are consistent with literature values [25].

3-Methoxybenzoic acid hydrazide (14)

The general procedure starting from 3-methoxybenzoic acid methyl ester (**2c**) (16.6g, 100 mmol), hydrazine monohydrate (7.3 mL, 150 mmol) and methanol (60 mL) was employed at gentle reflux with 48h reaction time. Solvent was evaporated to dryness, and organic product was crystallized from DEE

(110 mL/g) by slow distillation to obtain colorless powder of 3-methoxybenzoic acid hydrazide (**14**) (14.3g, 86 mmol) with 86% yield, which melts at 96–98 °C (from DEE) (m.p. 93–95 °C [26]). Selected FT-IR (ATR): ν_{max} 3287 (N-H), 3205 (NH_2), 3071 (C_{Ar}-H), 3003 (C_{Ar}-H), 2942 (CH_3), 2840 (CH_3), 1637 (C=O), 1620 (C=C), 1580 (N-H), 1526, 1470, 1328, 1316, 1296, 1249 (C_{Ar}-O), 1124, 1035 (C_{Alkyl}-O), 995, 894, 862, 803, 693, 649, 539 cm^{-1}; ^{1}H-NMR (DMSO-d_6, 400 MHz): δ 9.77 (s, 1H, NH), 7.41 (ddd, ^3J = 7.6 Hz, ^4J = 1.5 Hz, ^4J = 1.2 Hz, 1H, H-4), 7.37 (dd, ^4J = 2.7 Hz, ^4J = 1.5 Hz, 1H, H-2), 7.35 (dd, ^3J = 8.1 Hz, ^3J = 7.6 Hz, 1H, H-5), 4.51 (s, 2H, NH_2), 3.79 (s, 3H, OMe); ^{13}C-NMR (DMSO-d_6, 100 MHz): δ 165.71 (C=O), 159.13 (C), 134.61 (C), 129.43 (C-H), 119.19 (C-H), 116.90 (C-H), 112.11 (C-H), 55.16 (OMe) ppm; HRMS (TOF, MS, ESI) m/z for $C_8H_{10}N_2O_2$ + H^+: calculated 167.0815, found: 167.0816. ^{1}H-NMR and ^{13}C-NMR data are consistent with literature values [27].

4-Methoxybenzoic acid hydrazide (15)

The literature procedure was applied [3] to obtain colorless powder of 4-methoxybenzoic acid hydrazide (**15**) with 85% yield, which melts at 137–139 °C (from CH_3OH) (m.p. 136°C [25]). Selected FT-IR (ATR): ν_{max} 3315 (N-H), 3182 (NH_2), 3059 (C_{Ar}-H), 3019 (C_{Ar}-H), 2963 (CH_3), 2839 (CH_3), 1650 (C=O), 1609 (N-H), 1575, 1558 (C=C), 1501, 1469, 1455, 1344, 1304, 1252 (C_{Ar}-O), 1188, 1173, 1112, 1030 (C_{Alkyl}-O), 983, 884, 835, 758, 657, 634, 608, 501 cm^{-1}; ^{1}H-NMR (DMSO-d_6, 400 MHz): δ 9.62 (s, 1H, NH), 7.81 (d, ^3J = 9.0 Hz, 1H, H-2,6), 6.97 (d, ^3J = 9.0 Hz, 1H, H-3,5), 4.43 (s, 2H, NH_2), 3.79 (s, 3H, OMe); HRMS (TOF, MS, ESI) m/z for $C_8H_{10}N_2O_2$ + H^+: calculated 167.0815, found: 167.0821. ^{1}H-NMR and ^{13}C-NMR data are consistent with literature values [25].

2-Hydroxybenzoic acid hydrazide (16)

The general procedure starting from 2-hydroxy-benzoic acid methyl ester (**2e**) (7.61g, 50 mmol), and hydrazine monohydrate (2.67 mL, 2.75g, 55 mmol) in methanol (25 mL) at gentle reflux with 24 h was employed. Solvent was evaporation and dry under 20 mmHg under P_2O_5 to obtain oily material (7.52g) almost quantitatively. Analytical sample was obtained by direct crystallization at –24 °C, and recrystallization from methanol to obtain colorless powder of 2-hydroxybenzoic acid hydrazide (**16**) (4.6g, 30 mmol, 60% yield) which melts at 149–151 °C (from CH_3OH) (m.p. 147–150 °C [28]). Selected FT-IR (ATR): ν_{max} 3319 (N-H), 3248 (NH_2), 3100-3000 (br., C_{Ar}-H), 1644 (C=O), 1583 (NH_2, C=C), 1527, 1480, 1438, 1348, 1300, 1250 (C_{Ar}-O), 1236, 1133, 962, 946, 820, 790, 753, 663, 529, 510, 434 cm^{-1}; ^{1}H-NMR (300MHz, DMSO-d_6): δ 12.42 (s, 1H, OH), 10.05 (s, 1H, NH), 7.80 (d, J = 7.9 Hz, 1H, H-6), 7.37 (dd, J = 8.9 Hz, J = 8.5 Hz, 1H, H-4), 6.89 (d, J = 8.5 Hz, 1H, H-3), 6.83 (d, J = 8.9 Hz, 1H, H-5), 4.66 (s, 2H, NH_2) ppm; ^{13}C-NMR (DMSO-d_6, 100 MHz): δ 167.74 (C=O), 159.52 (C-6), 133.30 (C-4), 127.00 (C-6), 118.54 (C-5), 117.21 (C-3), 114.30 (C-1) ppm; HRMS (TOF, MS, ESI) m/z for $C_7H_8N_2O$ + H^+: calculated 153.0659, found: 153.0666. ^{1}H-NMR and ^{13}C-NMR data are consistent with literature values [29].

3,5-Dihydroxybenzoic acid hydrazide (17)

The general procedure starting from 3,5-dihydroxybenzoic acid methyl ester (**2i**) (1.86g, 10 mmol), and hydrazine monohydrate (0.53 mL, 11 mmol) in methanol (5.0 mL) at gentle reflux with 60h was employed, and crystallized with adding water to obtain colorless powder of 3,5-dihydroxybenzoic acid hydrazide (**17**) (1.40g, 8.3 mmol) with 83% yield, which melts at 262–264 °C with decomposition (from CH_3OH) (m.p. 264.5–265 °C [30]). Selected FT-IR (ATR): ν_{max} 3329 (N-H), 3251 (NH_2), 3100-3000 (br., C_{Ar}-H), 1605 (C=O), 1579 (NH_2, C=C), 1514, 1440, 1355, 1269 (C_{Ar}-O), 1154, 1111, 992, 940, 837, 828, 792, 757, 672, 616, 557, 506 cm^{-1}; ^{1}H-NMR (DMSO-d_6, 400 MHz): δ 9.51 (s, 1H, NH), 9.43 (s, 2H, OH), 6.63 (d, ^4J = 2.2 Hz, 2H, H-2,6), 6.33 (t, ^4J = 2.2 Hz, 1H, H-4), 4.39 (s, 2H, NH_2) ppm; ^{13}C-NMR (DMSO-d_6, 100 MHz): δ 166.19 (C=O), 158.16 (C-3, C-5), 135.42 (C-1), 105.17 (C-2, C-6), 104.90 (C-4) ppm; HRMS (TOF, MS, ESI) m/z for $C_7H_8N_2O_3$ + H^+: calculated 169.0608, found: 169.0614. ^{1}H-NMR and ^{13}C-NMR data are consistent with literature values [31].

3-Hydroxybenzoic acid hydrazide (18)

The literature procedure was applied [3] to obtain colorless powder of 3-hydroxybenzoic acid hydrazide (**18**) with 71% yield, which melts at 158–160 °C (from CH_3OH) (m.p. 157–159 °C [32]). Selected FT-IR (ATR): ν_{max} 3283 (N-H), 3196 (NH_2), 3100-3000 (br., C_{Ar}-H), 1629 (C=O), 1587 (NH_2), 1537 (C=C), 1502,

1447, 1341, 1313, 1275, 1252 (C_{Ar}-O), 1126, 1009, 997, 954, 869, 806, 765, 685, 675, 655, 557, 537, 430 cm^{-1}; ^{1}H-NMR (DMSO-d_6, 400 MHz): δ 9.66 (s, 1H, OH), 9.48 (s, 1H, NH), 7.18–7.24 (m, 3H, ArH), 6.85–6.92 (m, 1H, ArH), 4.45 (s, 2H, NH_2); ^{13}C-NMR (DMSO-d_6, 100 MHz): δ 165.97 (C=O), 157.25 (C), 134.72 (C), 129.27 (CH), 117.91 (CH), 117.34 (CH), 114.02 (CH) ppm; HRMS (TOF, MS, ESI) m/z for $C_7H_8N_2O + H^+$: calculated 153.0659, found: 153.0666. ^{1}H-NMR and ^{13}C-NMR data are consistent with literature values [29].

2-(1-Hydroxy)naphthoic acid hydrazide (19)

The general procedure starting from 2-(1-hydroxy)naphthoic acid methyl ester (**2h**) (15.2g, 75 mmol), and hydrazine monohydrate (4.0 mL, 82.5 mmol) with methanol (90 mL) was employed at gentle reflux with 72 h to obtain colorless powder of 2-(1-hydroxy)naphthoic acid hydrazide (**19**) (11.4g, 56 mmol) with 75% yield, which melt at 212–213 °C (from CH_3OH) (m.p. 211–213 °C [30]). Selected FT-IR (ATR): $ν_{max}$ 3307 (N-H), 3227 (NH_2), 3100-3000 (br., C_{Ar}-H), 1593 (C=O, NH_2), 1537 (C=C), 1499 (C=C), 1467, 1350, 1291, 1246 (C_{Ar}-O), 1163, 1146, 1023, 990, 924, 864, 771, 722, 665, 596, 566, 534, 490, 418 cm^{-1}; ^{1}H-NMR (DMSO-d_6, 300 MHz): δ 14.41 (s, 1H, OH), 10.30 (s, 1H, NH), 8.26 (d, ^3J = 8.2 Hz, 1H, ArH), 7.85 (d, ^3J = 7.9 Hz, 1H, ArH), 7.83 (d, ^3J = 8.8 Hz, 1H, ArH), 7.62 (ddd, ^3J = 7.9 Hz, ^3J = 6.9 Hz, ^4J = 1.4 Hz, 1H, ArH), 7.54 (ddd, ^3J = 8.2 Hz, ^3J = 6.9 Hz, ^4J = 1.2 Hz, 1H, ArH), 7.34 (d, ^3J = 8.8 Hz, 1H, ArH), 4.76 (s, 2H, NH_2) ppm; ^{13}C-NMR (75 MHz, DMSO-d_6) δ 169.28 (C=O), 158.98 (C), 135.62 (C), 128.62 (CH), 127.41 (CH), 125.68 (CH), 124.73 (C), 122.95 (CH), 122.06 (CH), 117.72 (CH), 106.27 (C) ppm; HRMS (TOF, MS, ESI) m/z for $C_{11}H_{10}N_2O_2 + H^+$: calculated 203.0815, found: 203.0822.

6. Selected ^{1}H, ^{13}C NMR and FT-IR spectra, DEPT-135 and 2D experiments of used compounds

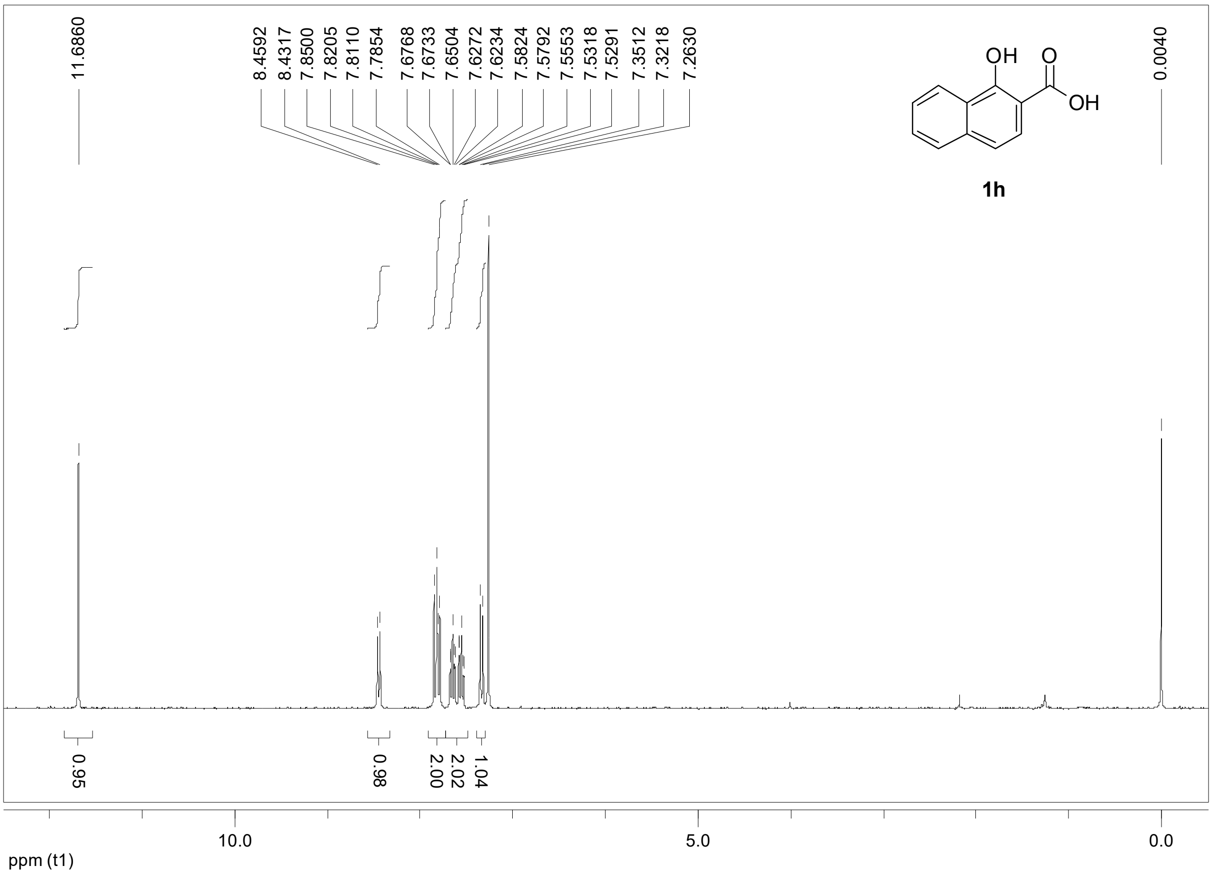

Figure S1. ^{1}H-NMR (300 MHz, $CDCl_3$) spectrum of 1-hydroxy-2-naphthoic acid (**1h**)

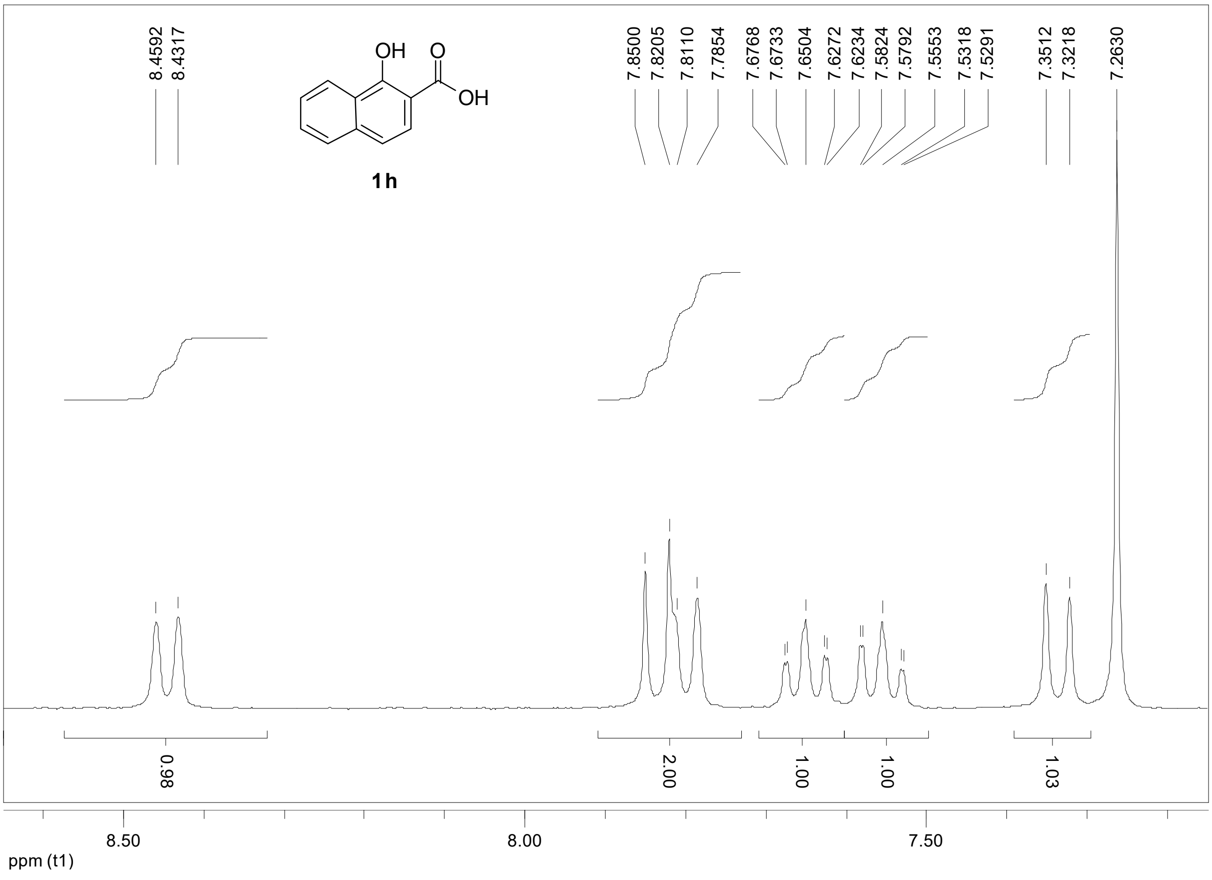

Figure S2. Expansion of ^{1}H-NMR (300 MHz, $CDCl_3$) spectrum of 1-hydroxy-2-naphthoic acid (**1h**)

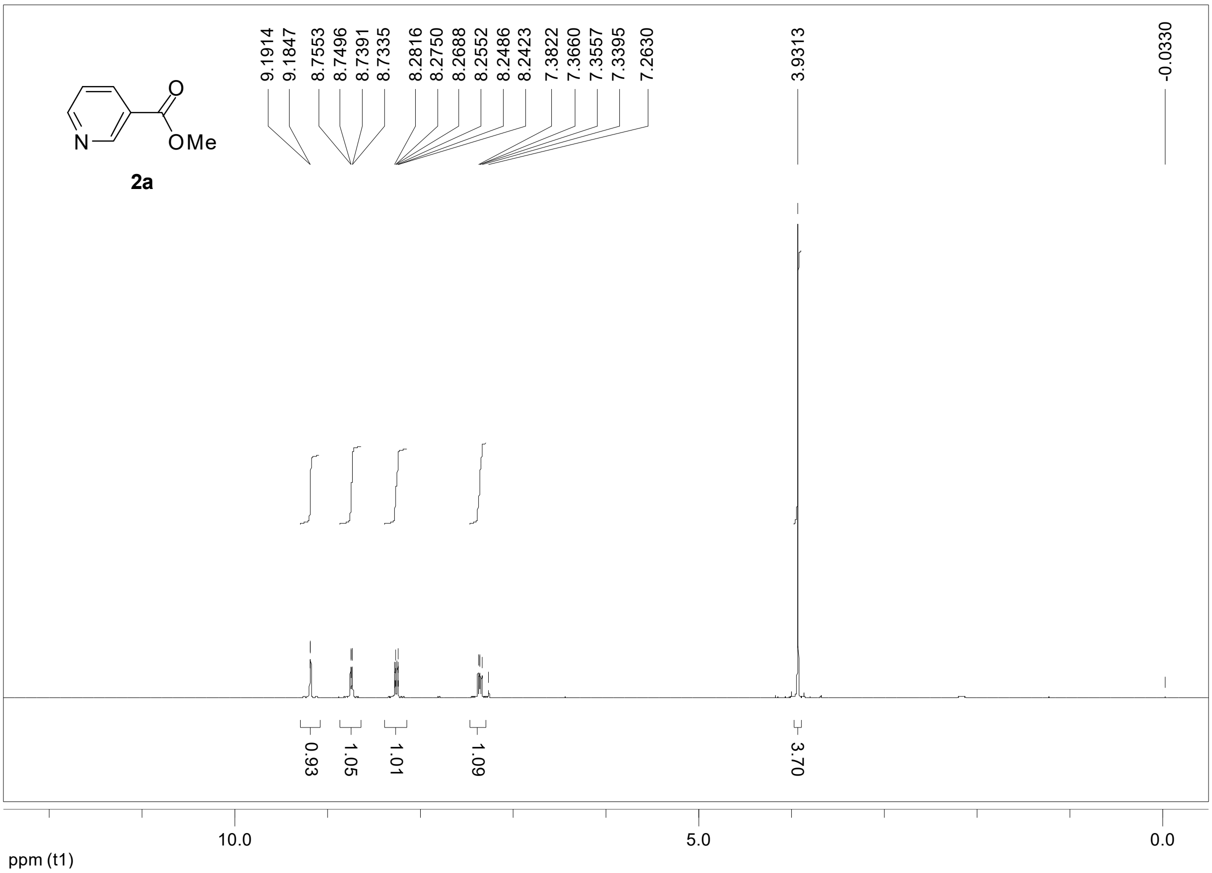

Figure S3. ^{1}H-NMR (300 MHz, $CDCl_3$) spectrum of nicotinic acid methyl ester (**2a**)

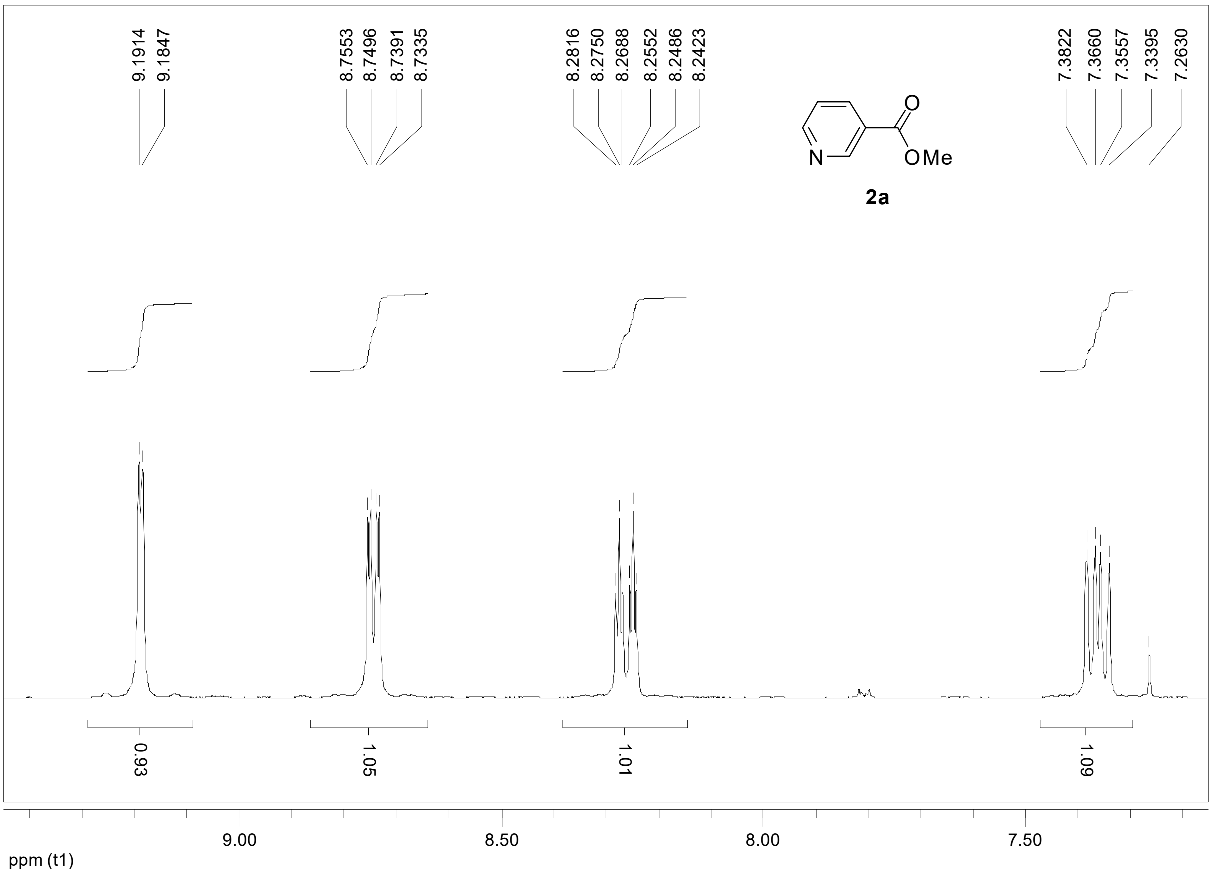

Figure S4. Expansion of ^{1}H-NMR (300 MHz, $CDCl_3$) spectrum of nicotinic acid methyl ester (**2a**)

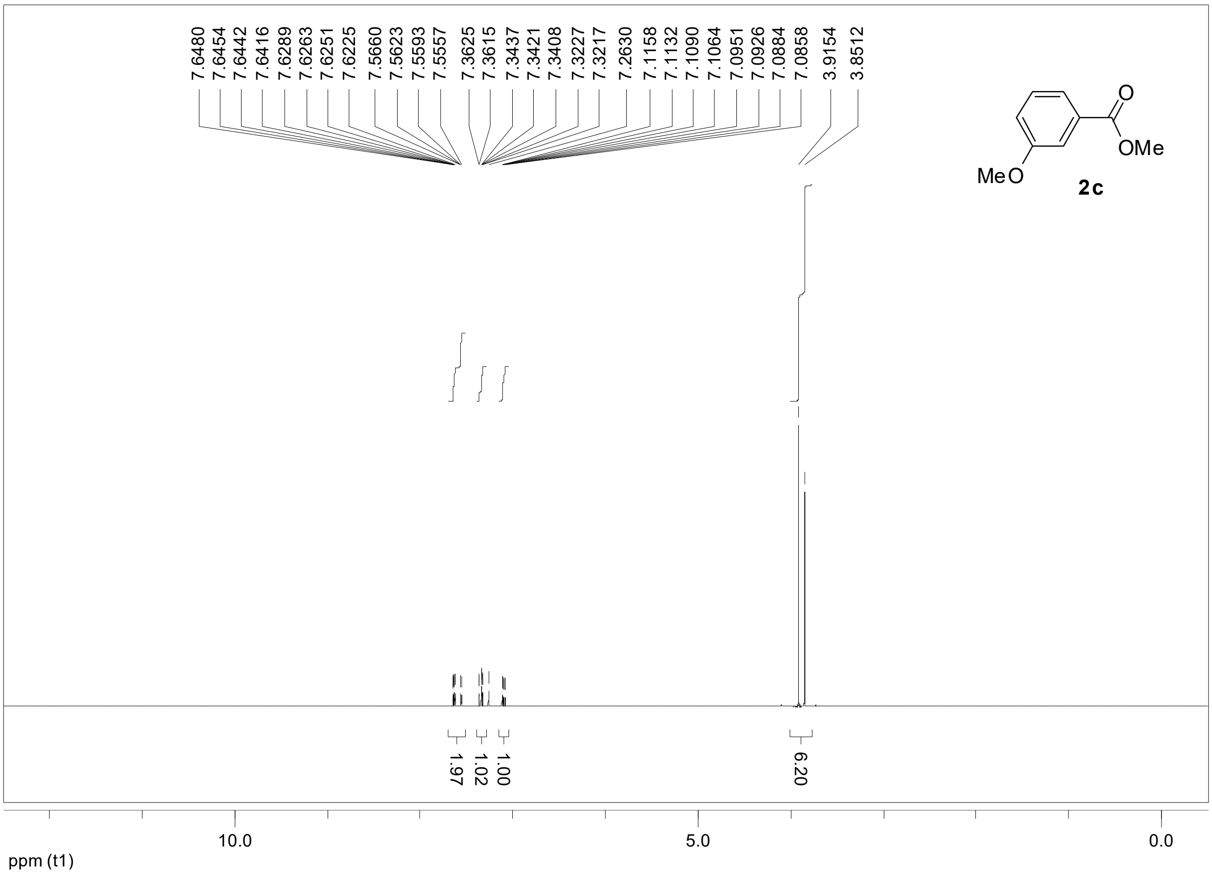

Figure S5. ^{1}H-NMR (300 MHz, $CDCl_3$) spectrum of 3-methoxybenzoic acid methyl ester (**2c**)

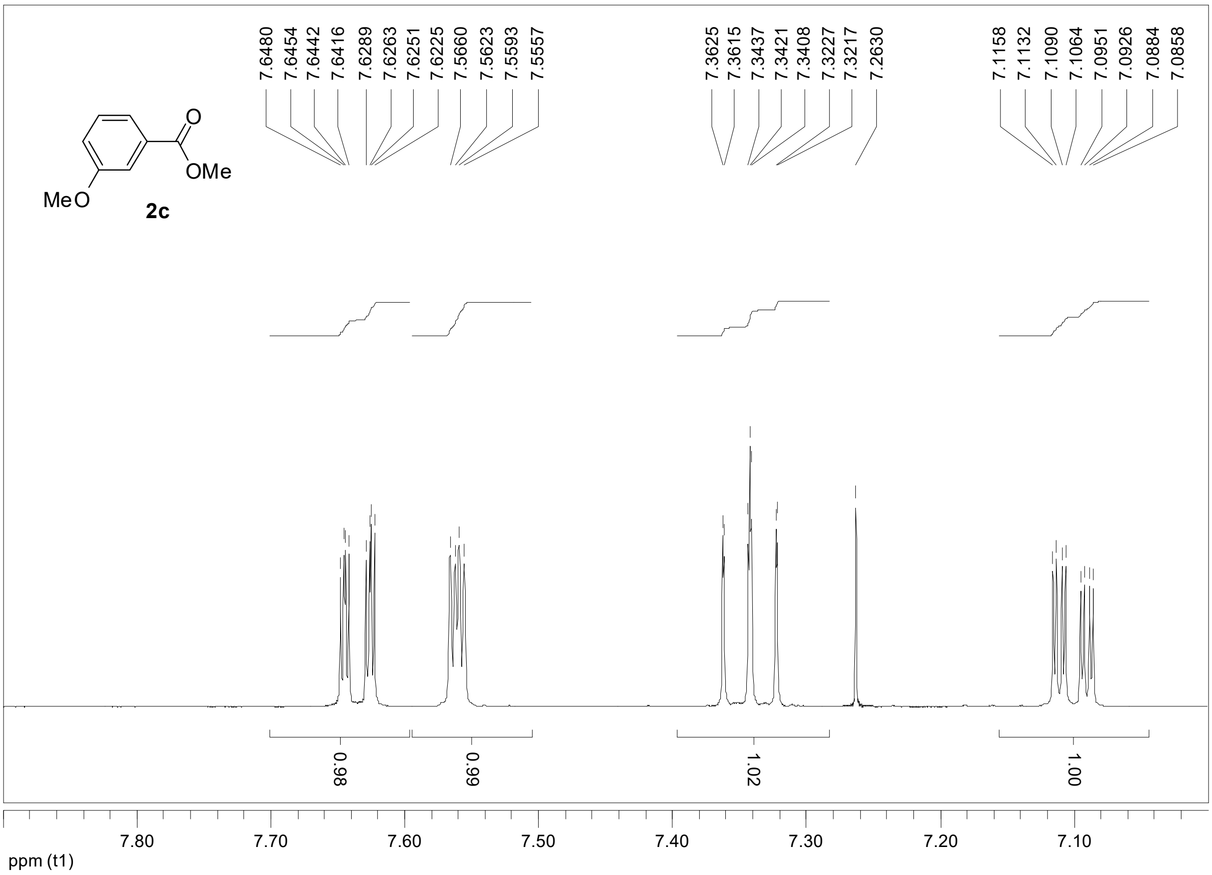

Figure S6. Expansion of ^{1}H-NMR (300 MHz, $CDCl_3$) spectrum of 3-methoxybenzoic acid methyl ester (**2c**)

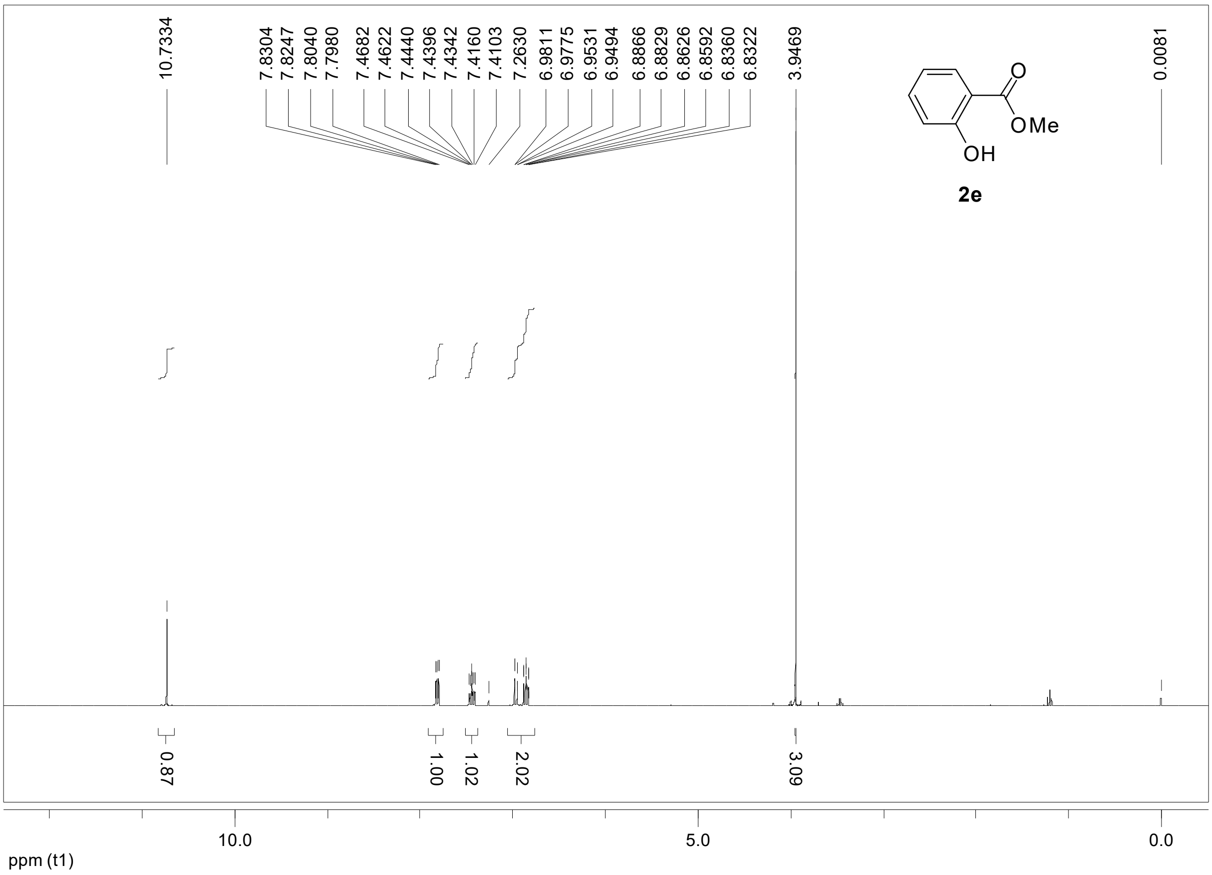

Figure S7. ^{1}H-NMR (300 MHz, $CDCl_3$) spectrum of salicylic acid methyl ester (**2e**)

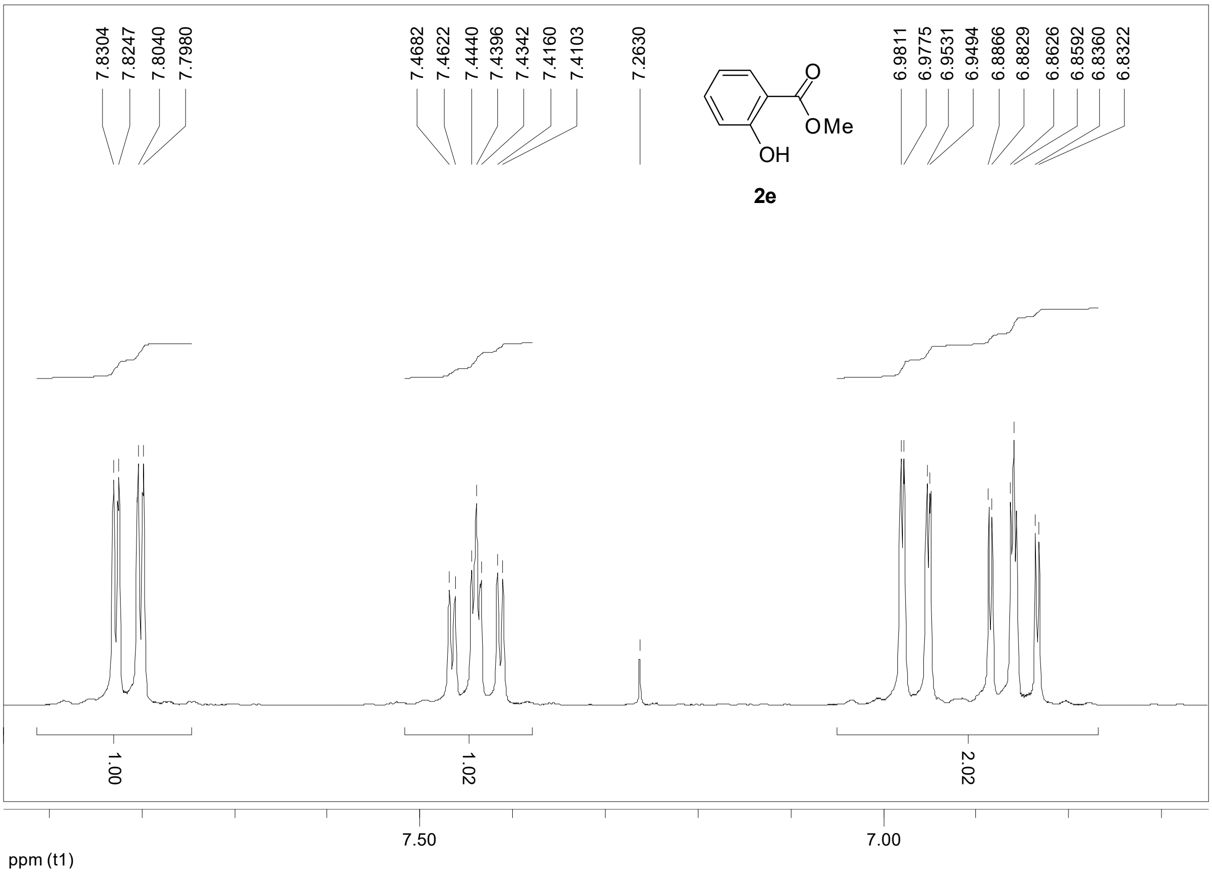

Figure S8. Expansion of ^{1}H-NMR (300 MHz, $CDCl_3$) spectrum of salicylic acid methyl ester (**2e**)

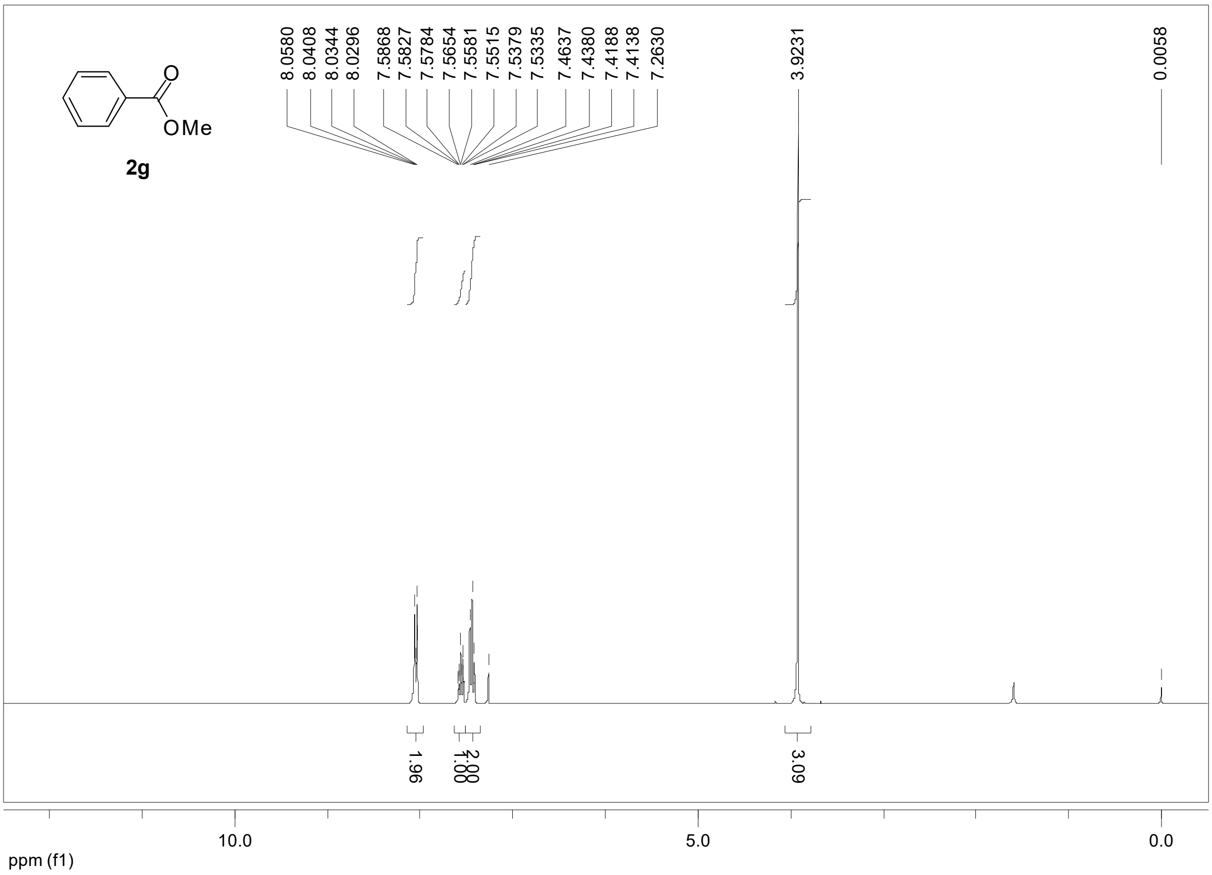

Figure S9. ^{1}H-NMR (300 MHz, $CDCl_3$) spectrum of benzoic acid methyl ester (**2g**)

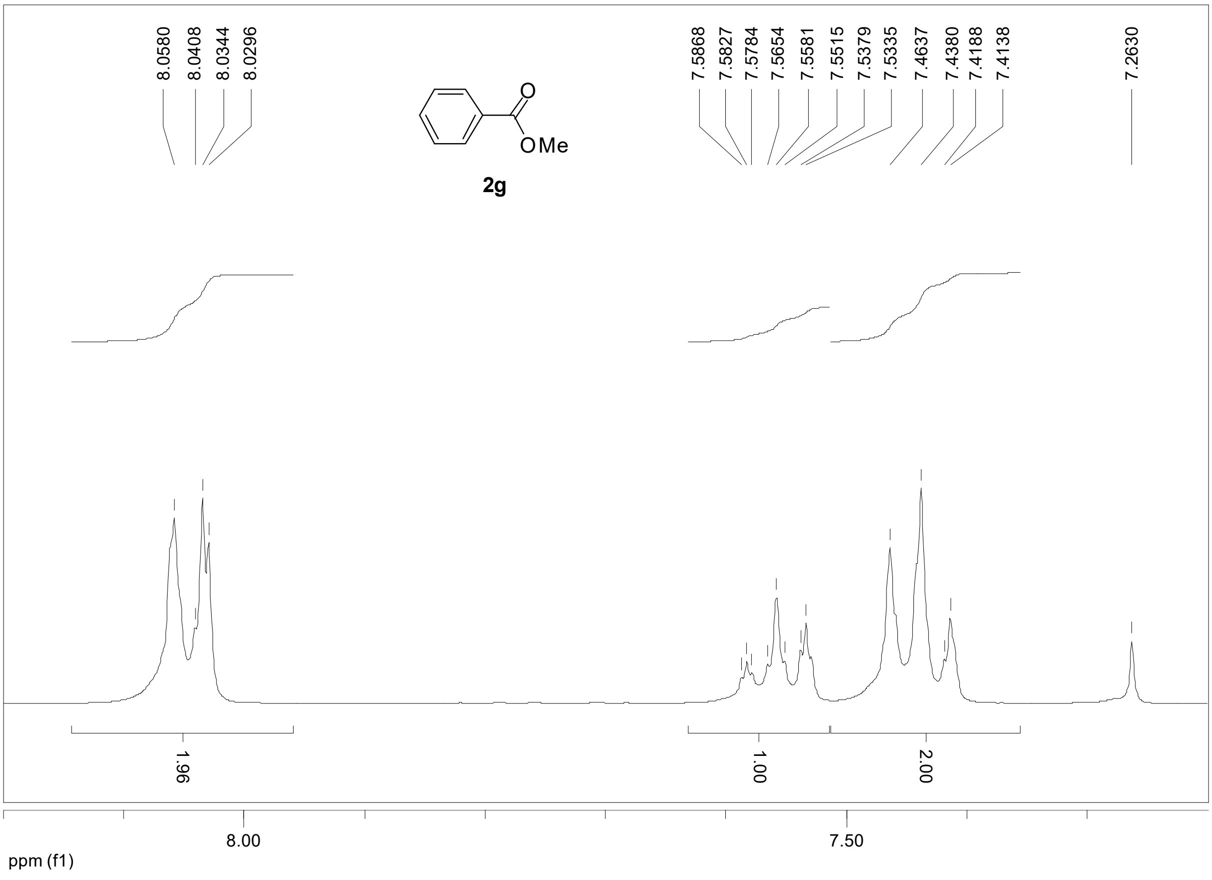

Figure S10. Expansion of ^{1}H-NMR (300 MHz, $CDCl_3$) spectrum of benzoic acid methyl ester (**2g**)

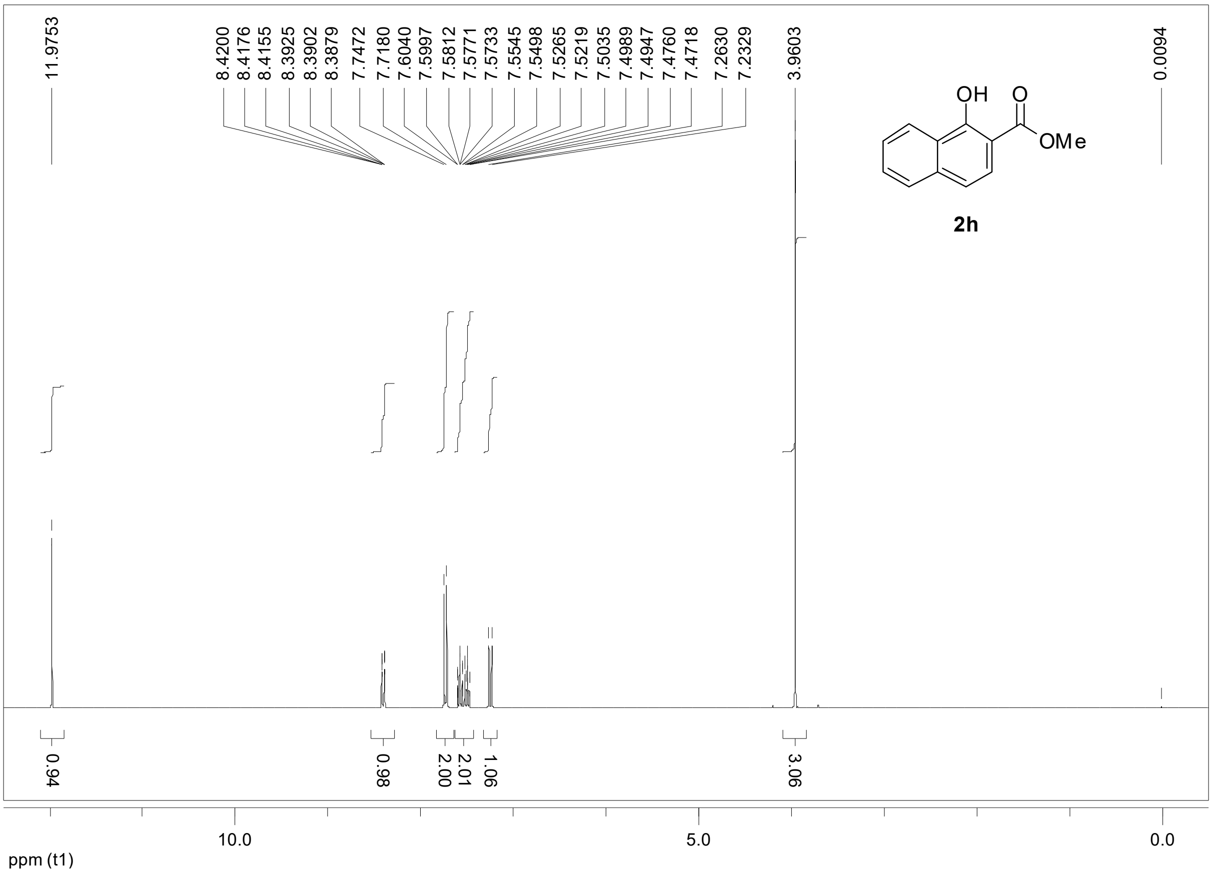

Figure S11. ¹H-NMR (300 MHz, $CDCl_3$) spectrum of 1-hydroxy-2-naphthoic acid methyl ester (**2h**)

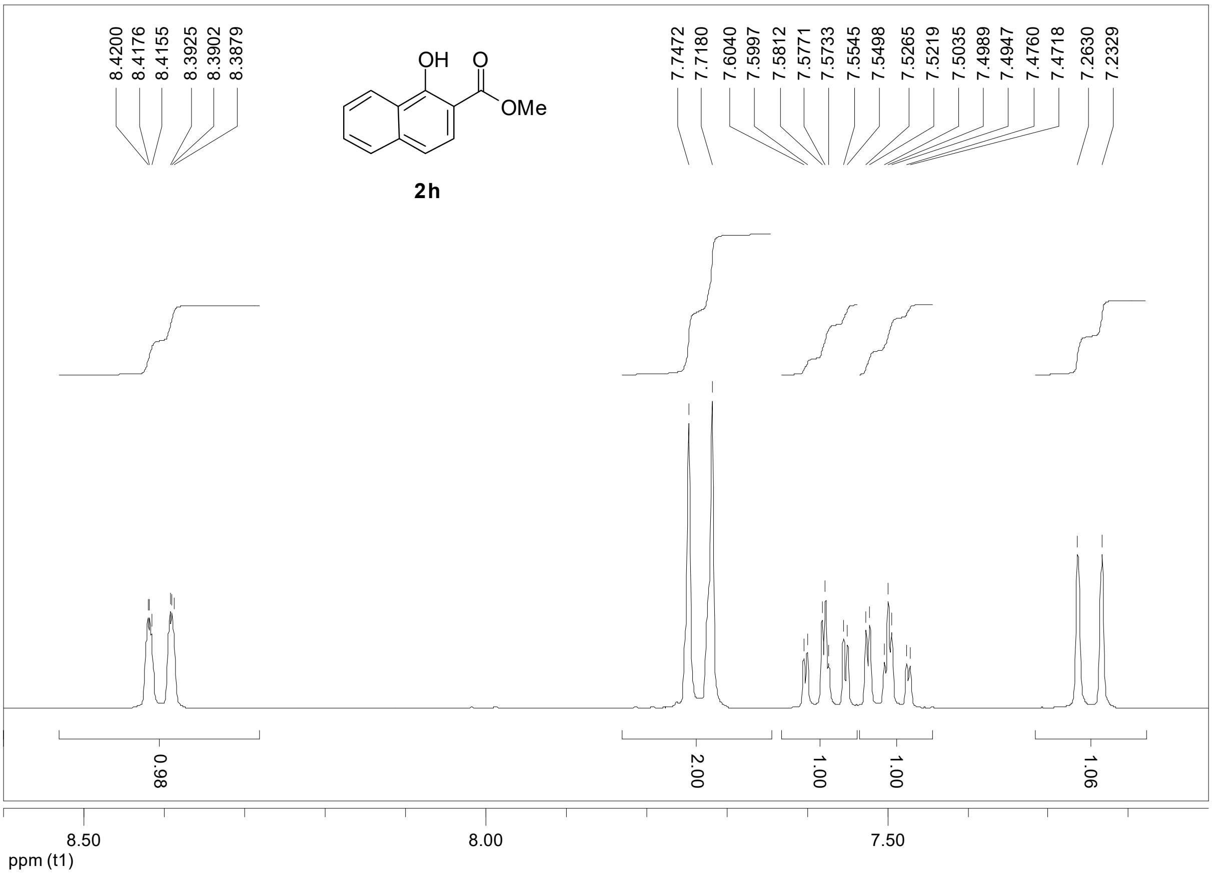

Figure S12. Expansion of ¹H-NMR (300 MHz, $CDCl_3$) spectrum of 1-hydroxy-2-naphthoic acid methyl ester (**2h**)

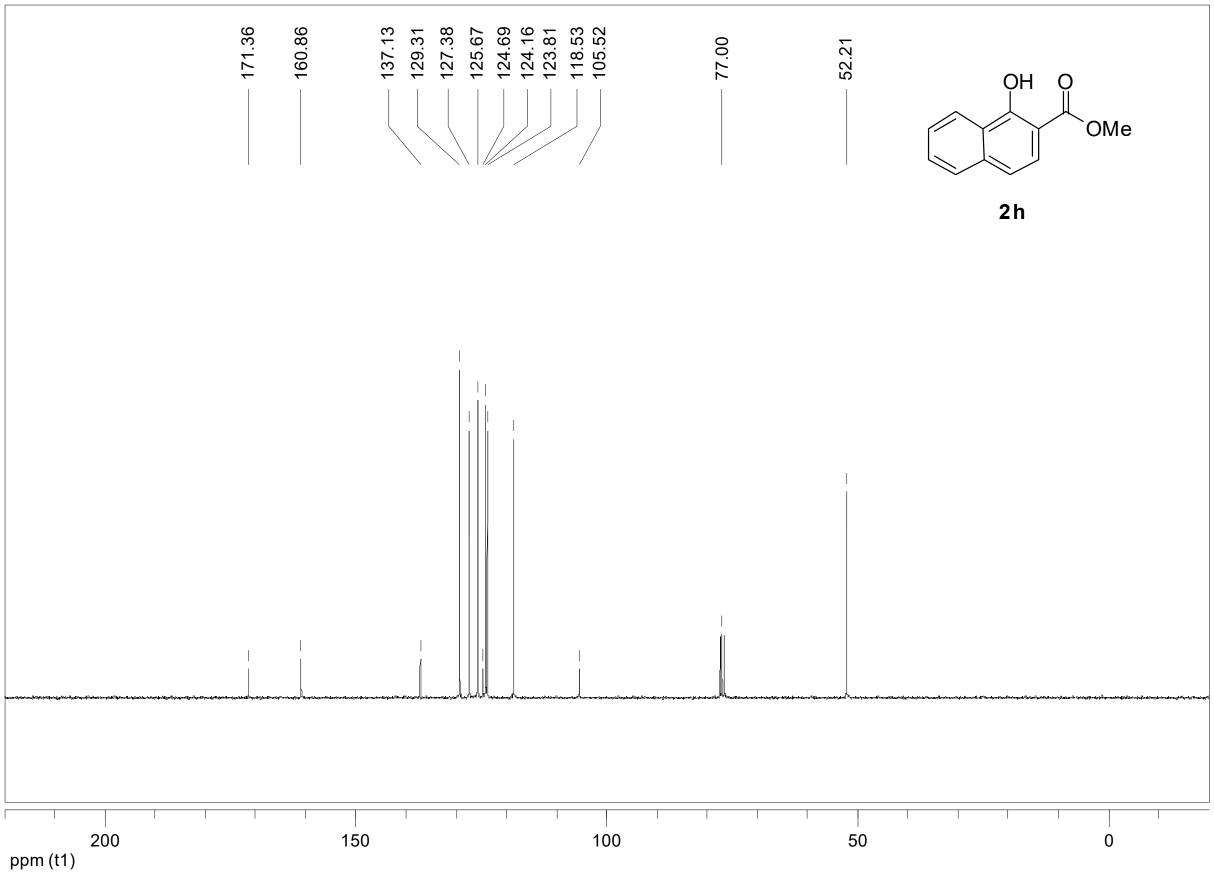

Figure S13. ^{13}C-NMR (75 MHz, $CDCl_3$) spectrum of 1-hydroxy-2-naphthoic acid methyl ester (**2h**)

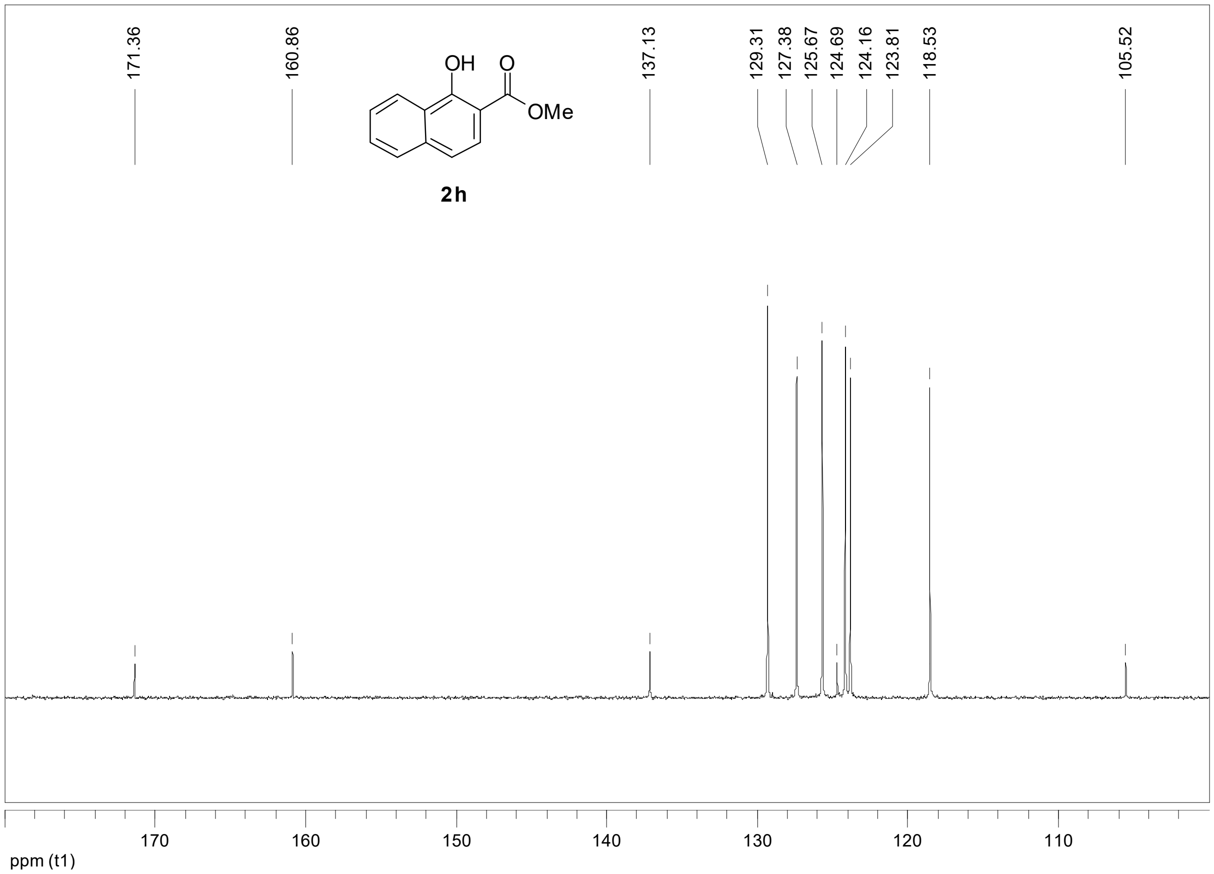

Figure S14. Expansion of ^{13}C-NMR (75 MHz, $CDCl_3$) spectrum of 1-hydroxy-2-naphthoic acid methyl ester (**2h**)

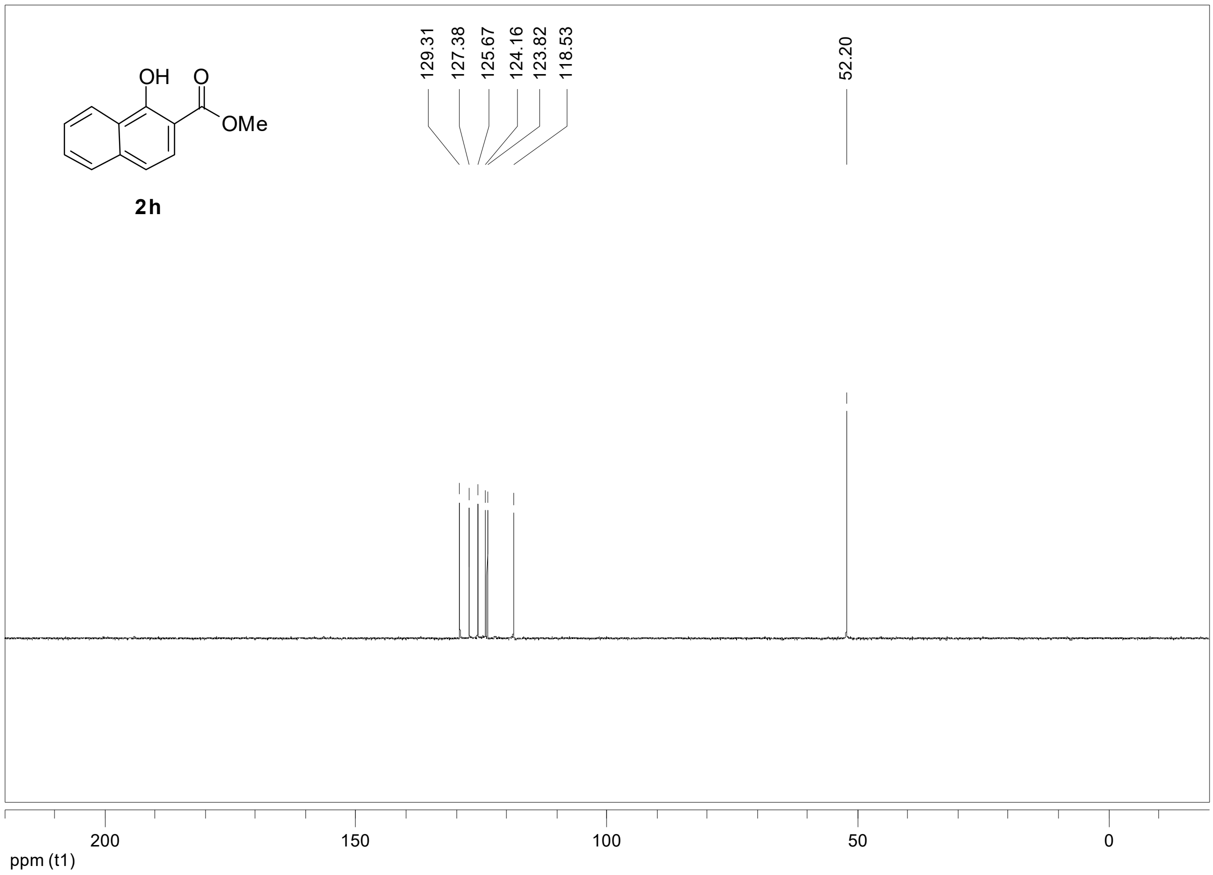

Figure S15. ^{13}C-NMR (75 MHz, $CDCl_3$) dept-135 experiment of 1-hydroxy-2-naphthoic acid methyl ester (**2h**)

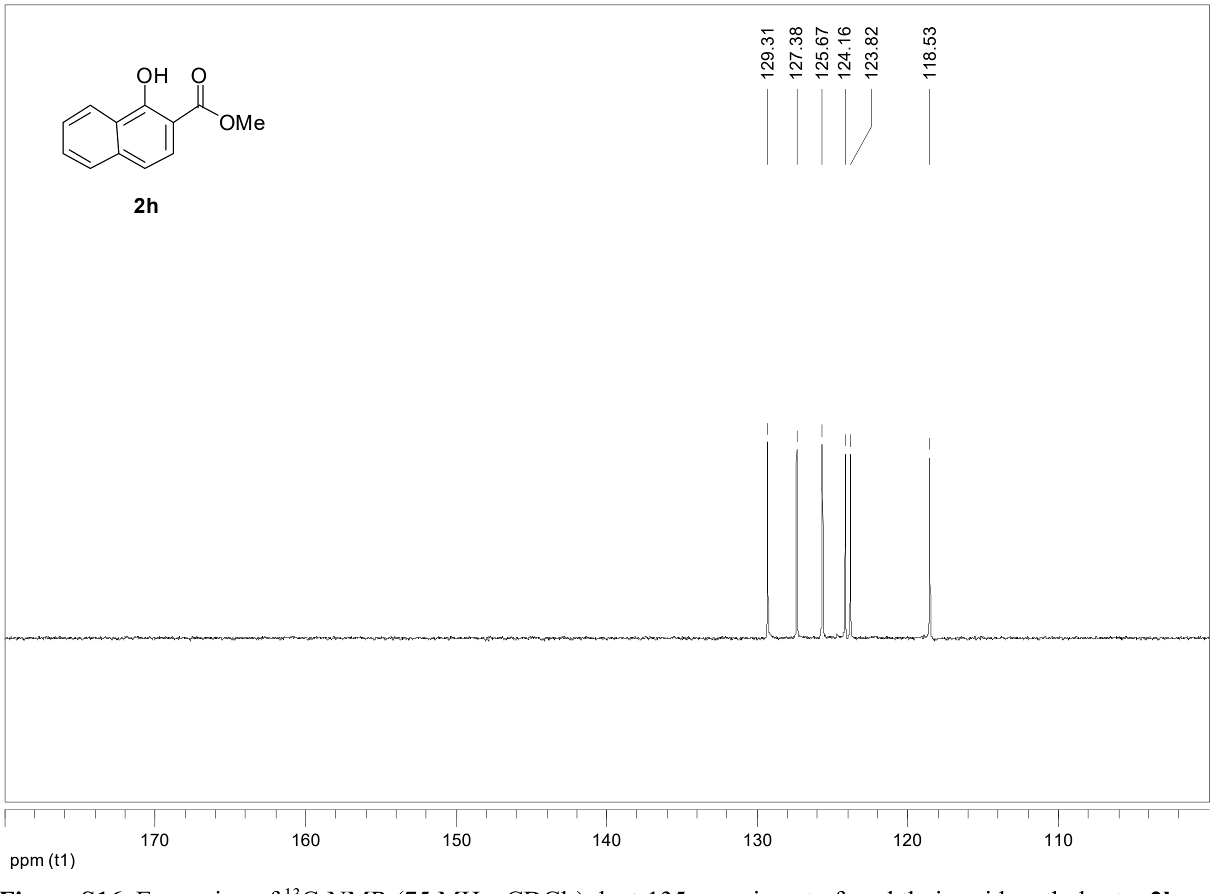

Figure S16. Expansion of ^{13}C-NMR (75 MHz, $CDCl_3$) dept-135 experiment of naphthoic acid methyl ester **2h**

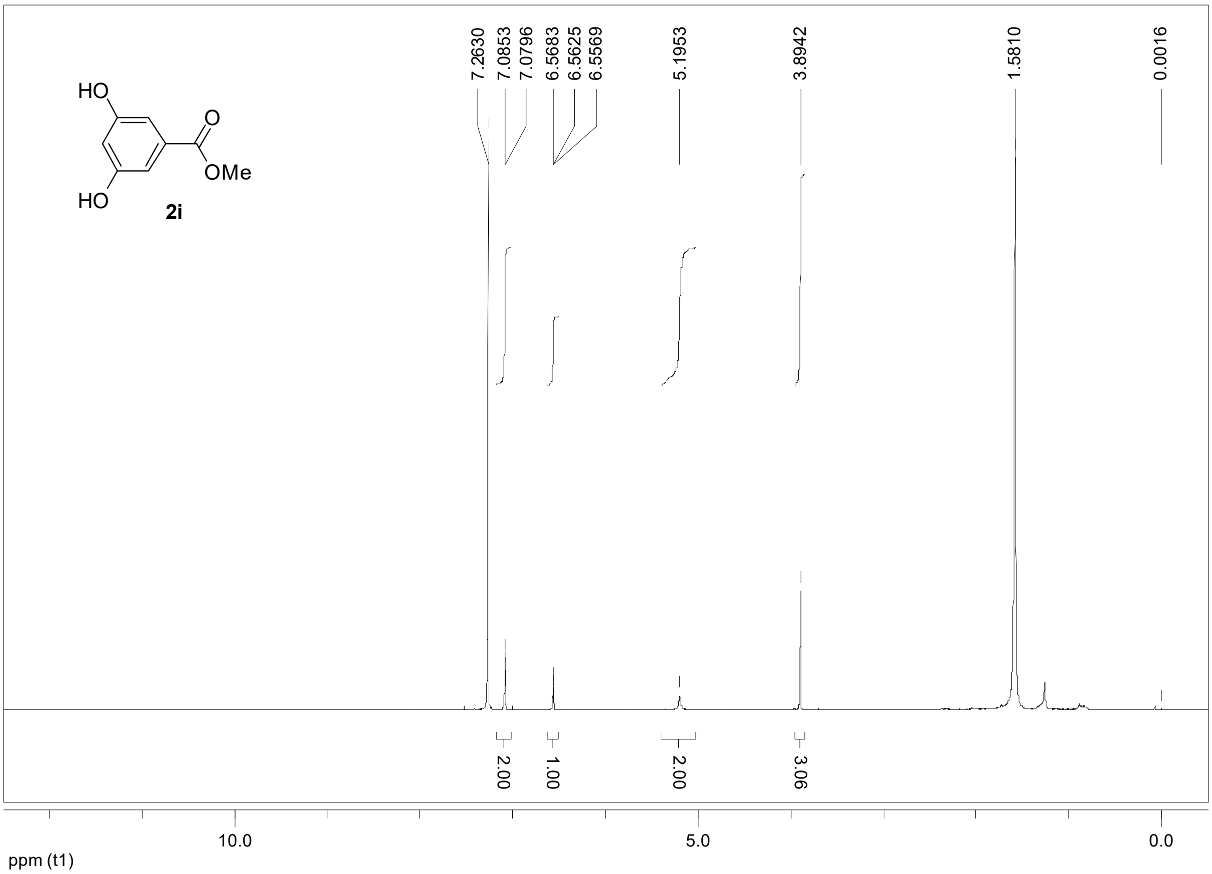

Figure S17. ^{1}H-NMR (400 MHz, $CDCl_3$) spectrum of α-resorcylic acid methyl ester (**2i**)

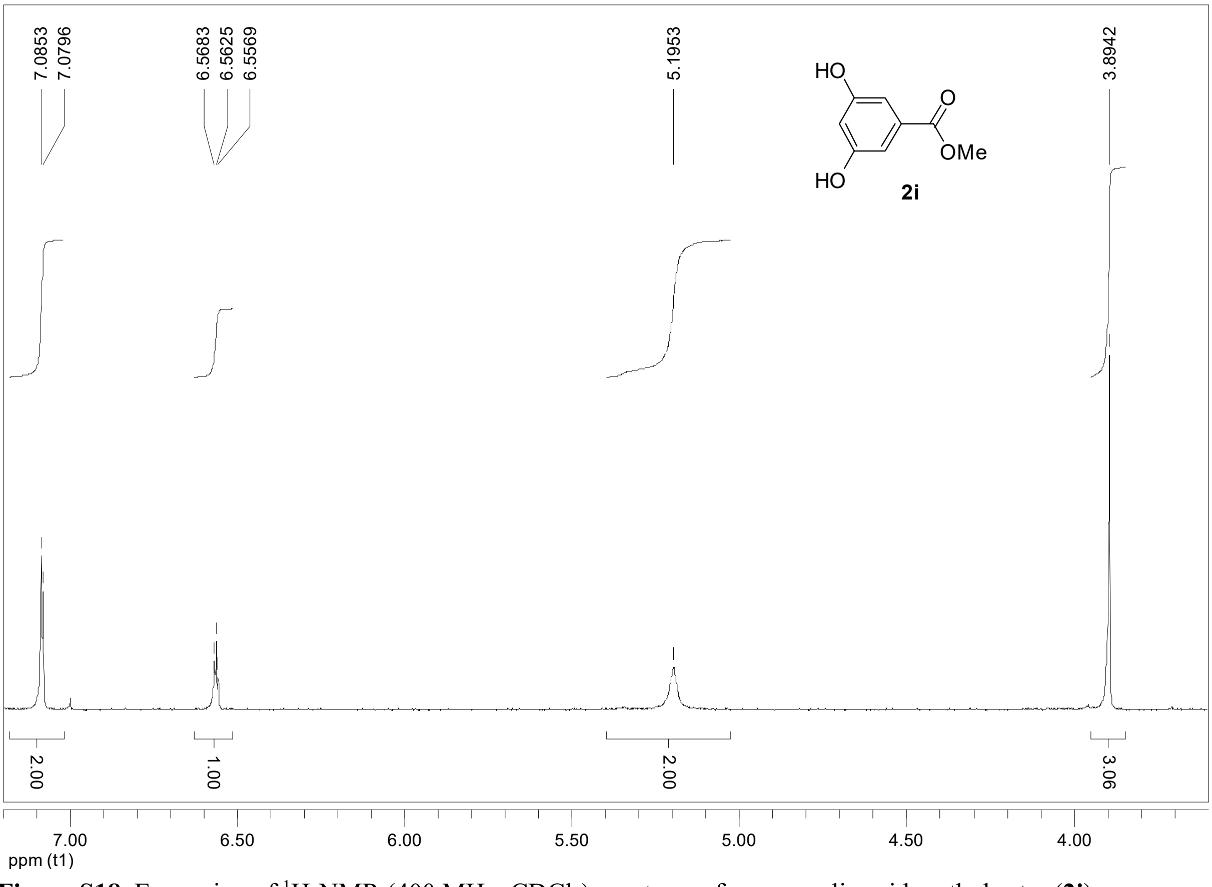

Figure S18. Expansion of ^{1}H-NMR (400 MHz, $CDCl_3$) spectrum of α-resorcylic acid methyl ester (**2i**)

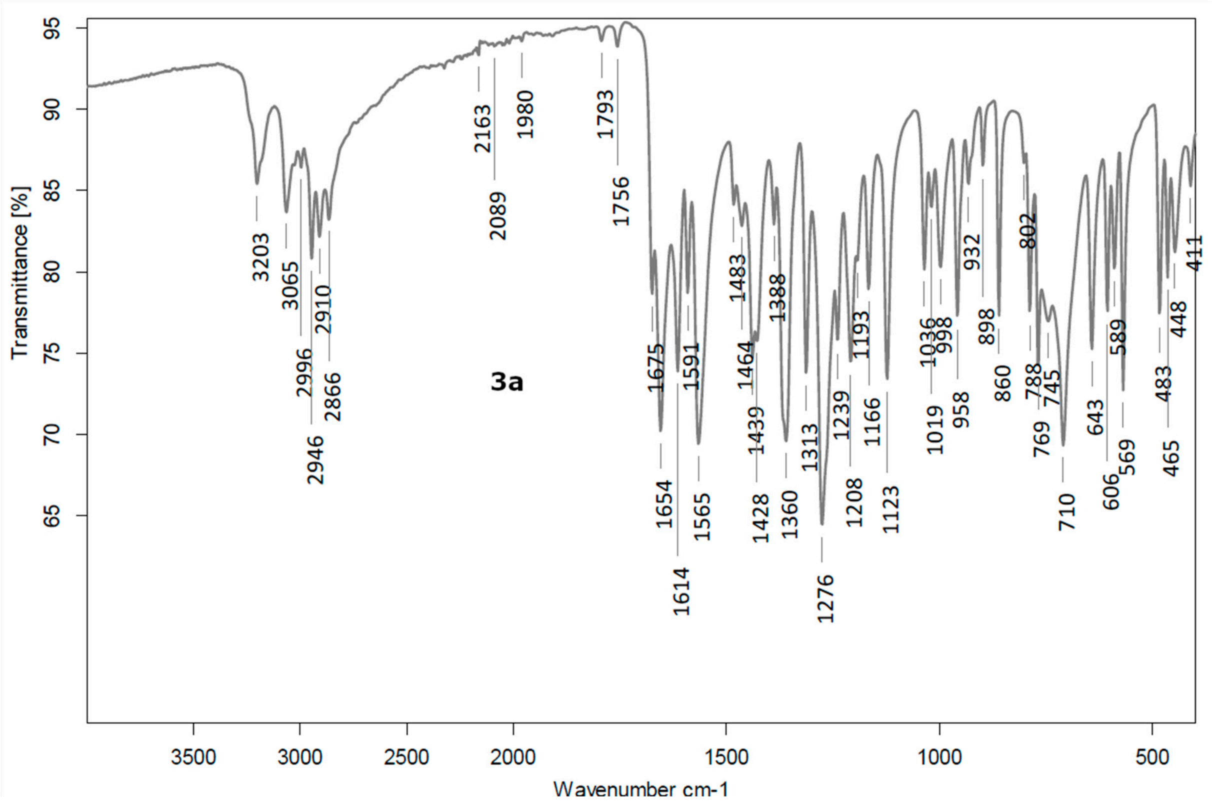

Figure S19. FT-IR (ATR) spectrum of (*E*/Z)-*N*'-(3-*tert*-butyl-2-hydroxy-5-methyl-benzylidene)acetohydrazide (**3a**)

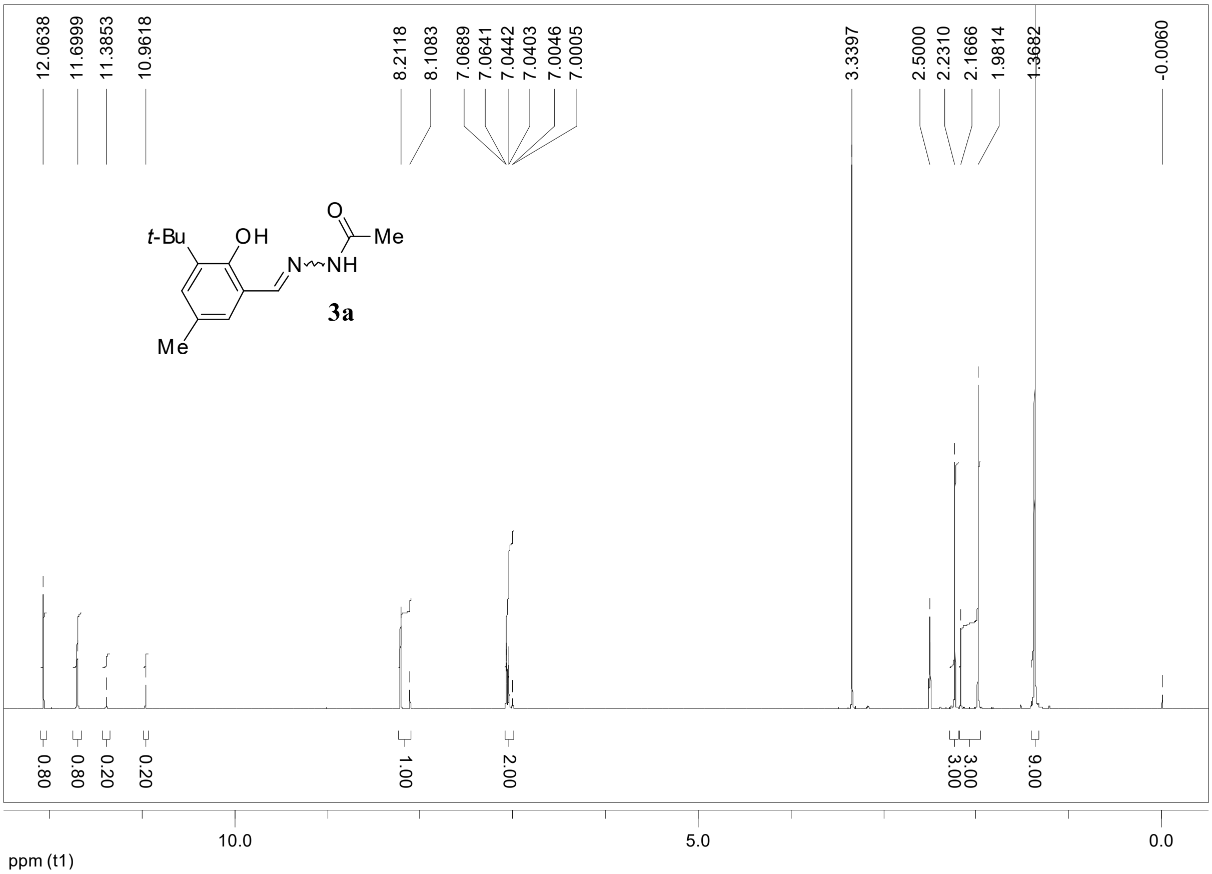

Figure S20. ^{1}H-NMR (400 MHz, DMSO-d_6) spectrum of (*E*/Z)-*N*'-(3-*tert*-butyl-2-hydroxy-5-methyl-benzylidene)acetohydrazide (**3a**)

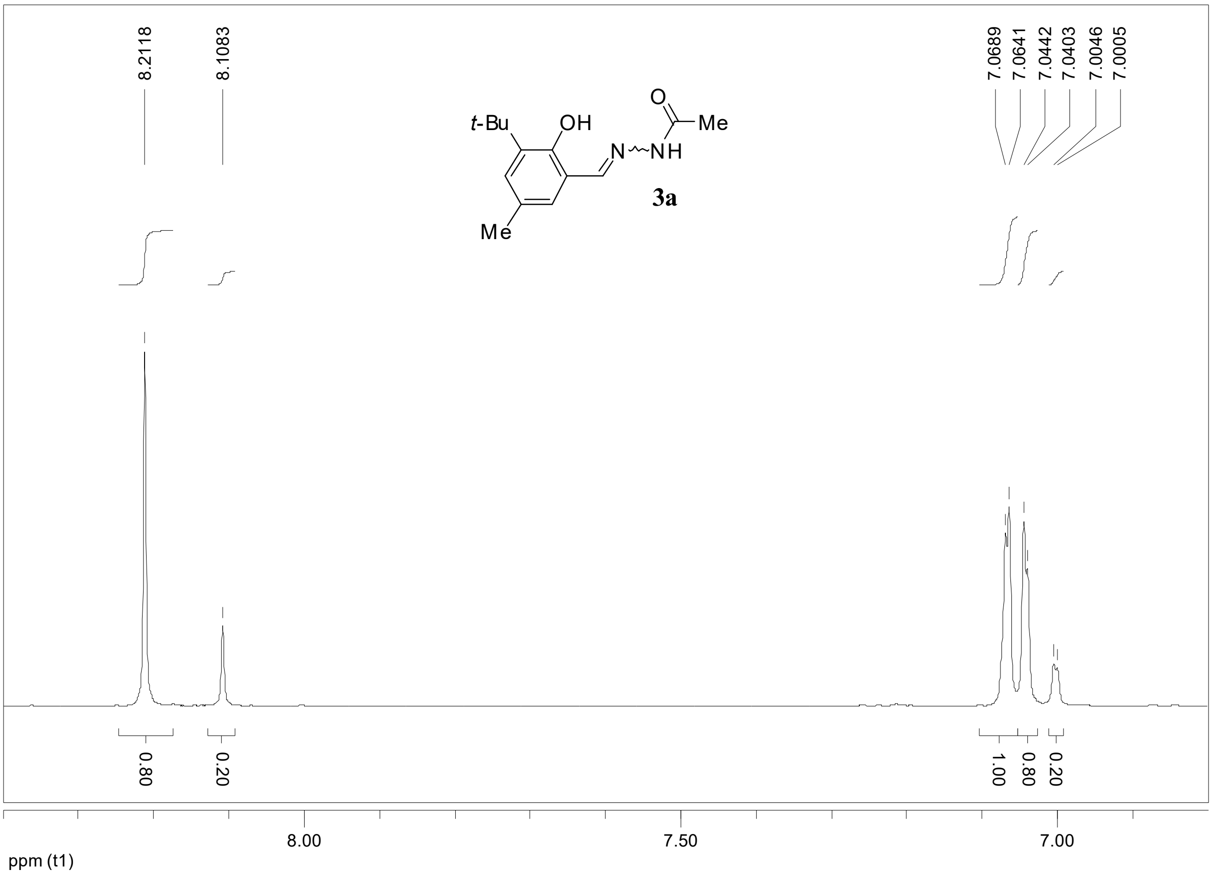

Figure S21. Expansion of ^{1}H-NMR (400 MHz, DMSO-d_6) spectrum of compound **3a**

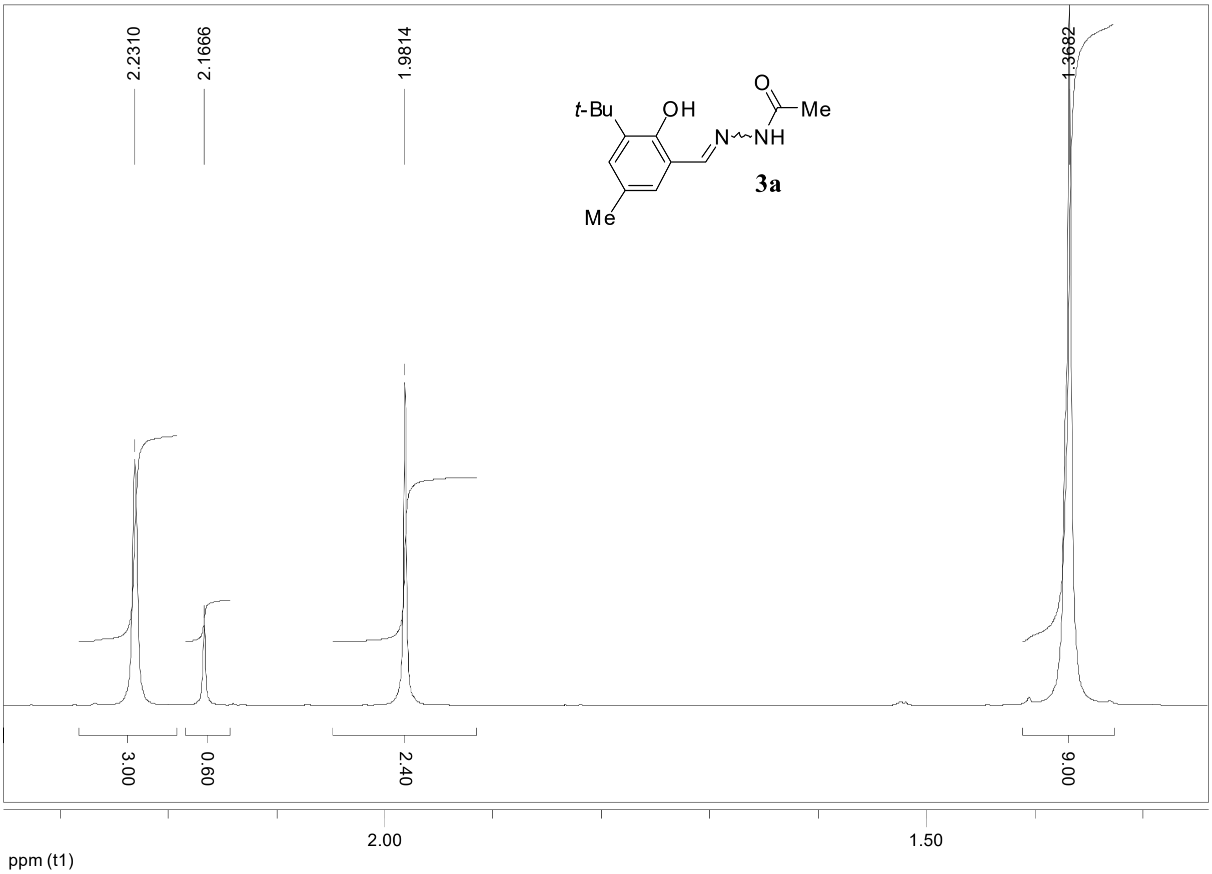

Figure S22. Expansion of ^{1}H-NMR (400 MHz, DMSO-d_6) spectrum of compound **3a**

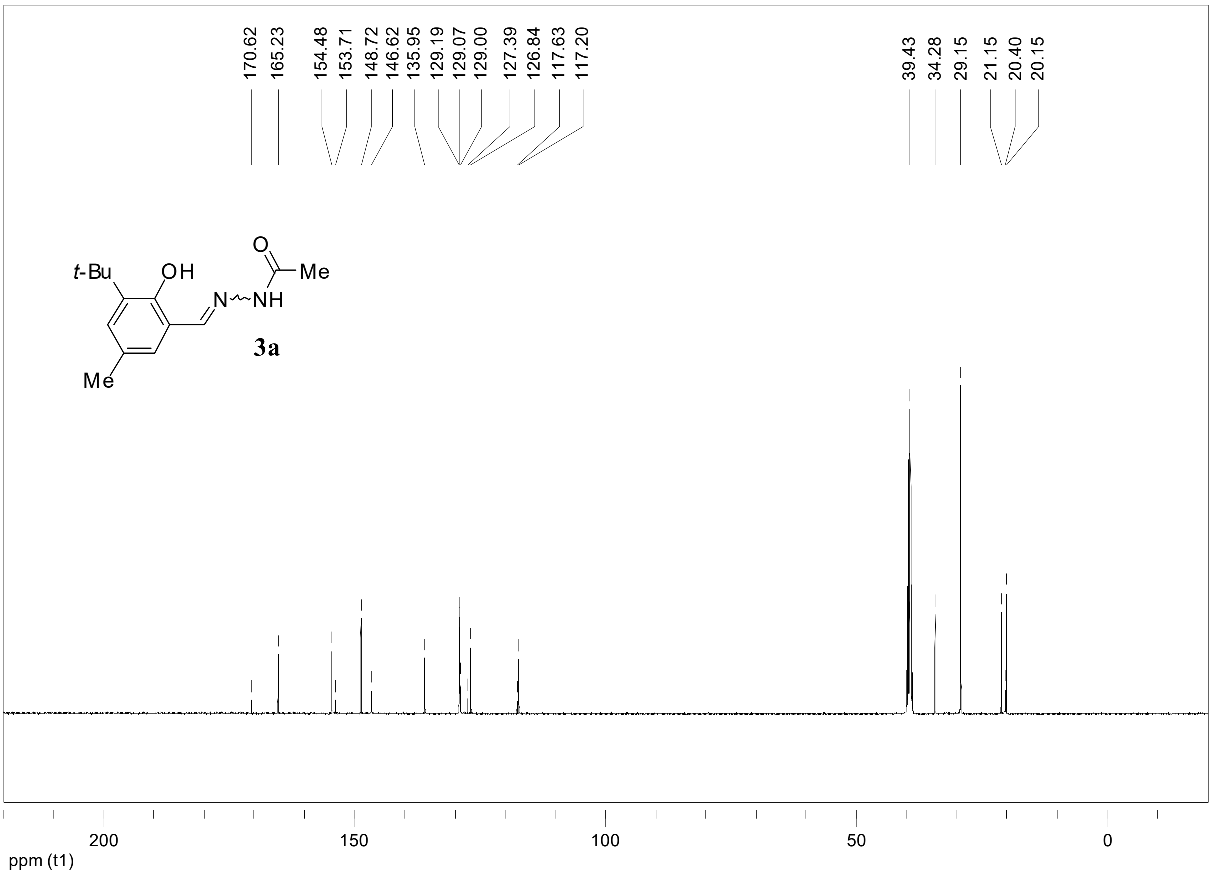

Figure S23. ^{13}C-NMR (100 MHz, DMSO-d_6) spectrum of compound **3a**

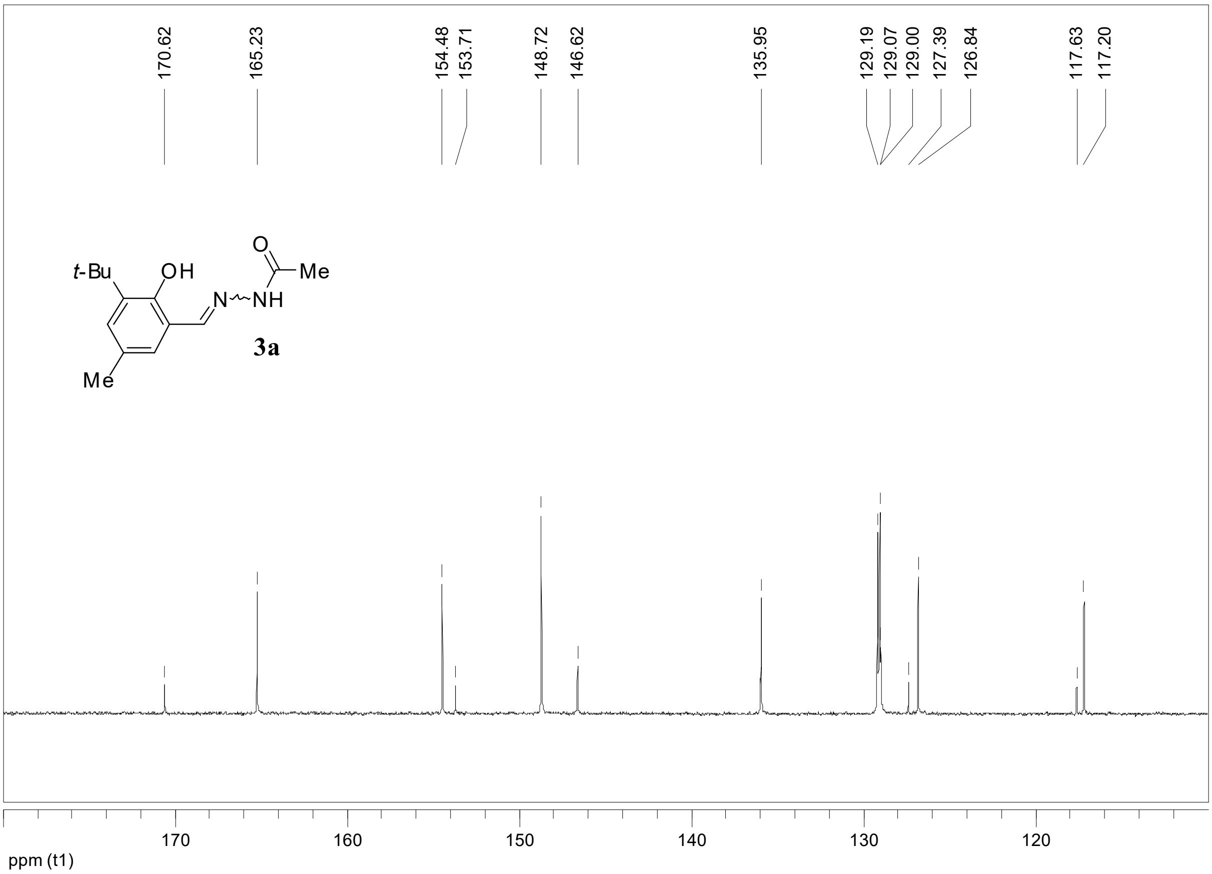

Figure S24. Expansion of ^{13}C-NMR (100 MHz, DMSO-d_6) spectrum of compound **3a**

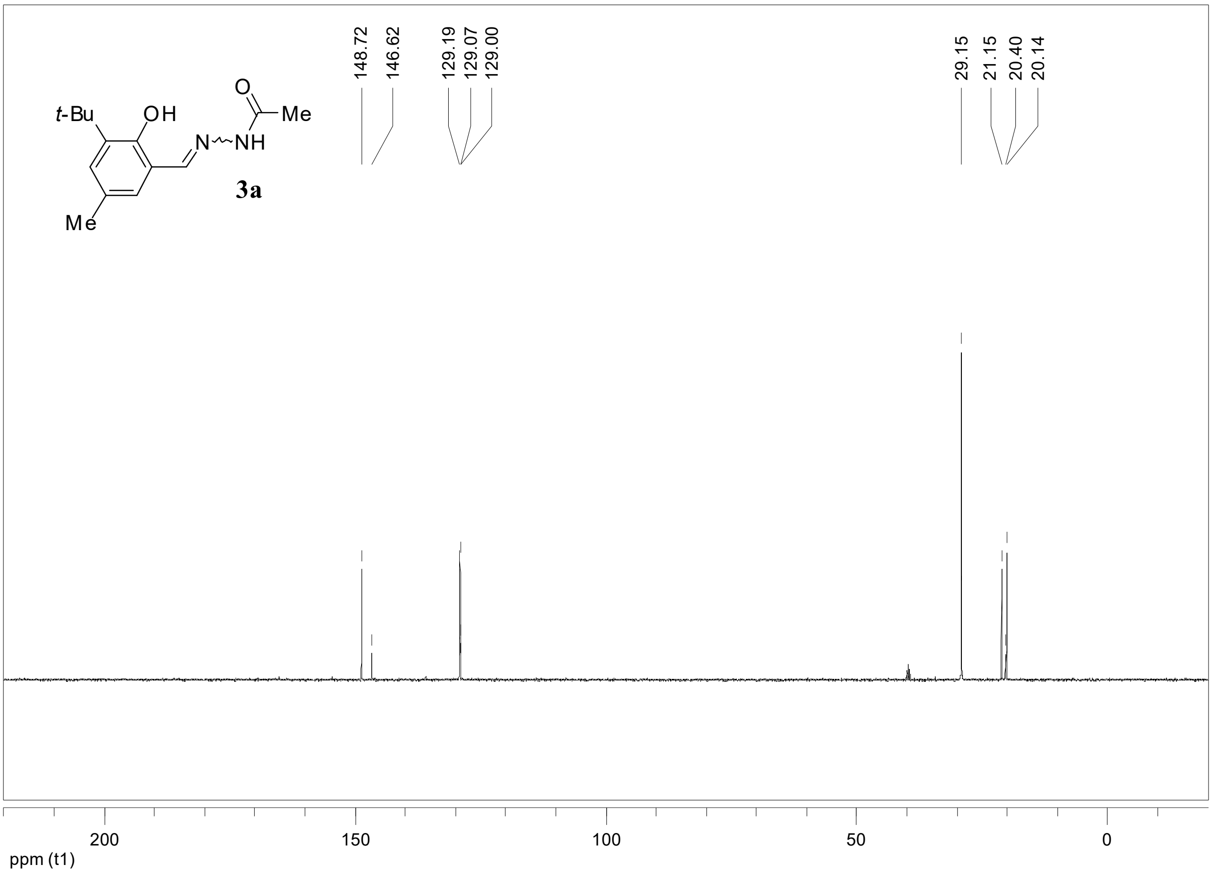

Figure S25. ^{13}C-NMR (100 MHz, DMSO-d_6) dept-135 experiment of compound **3a**

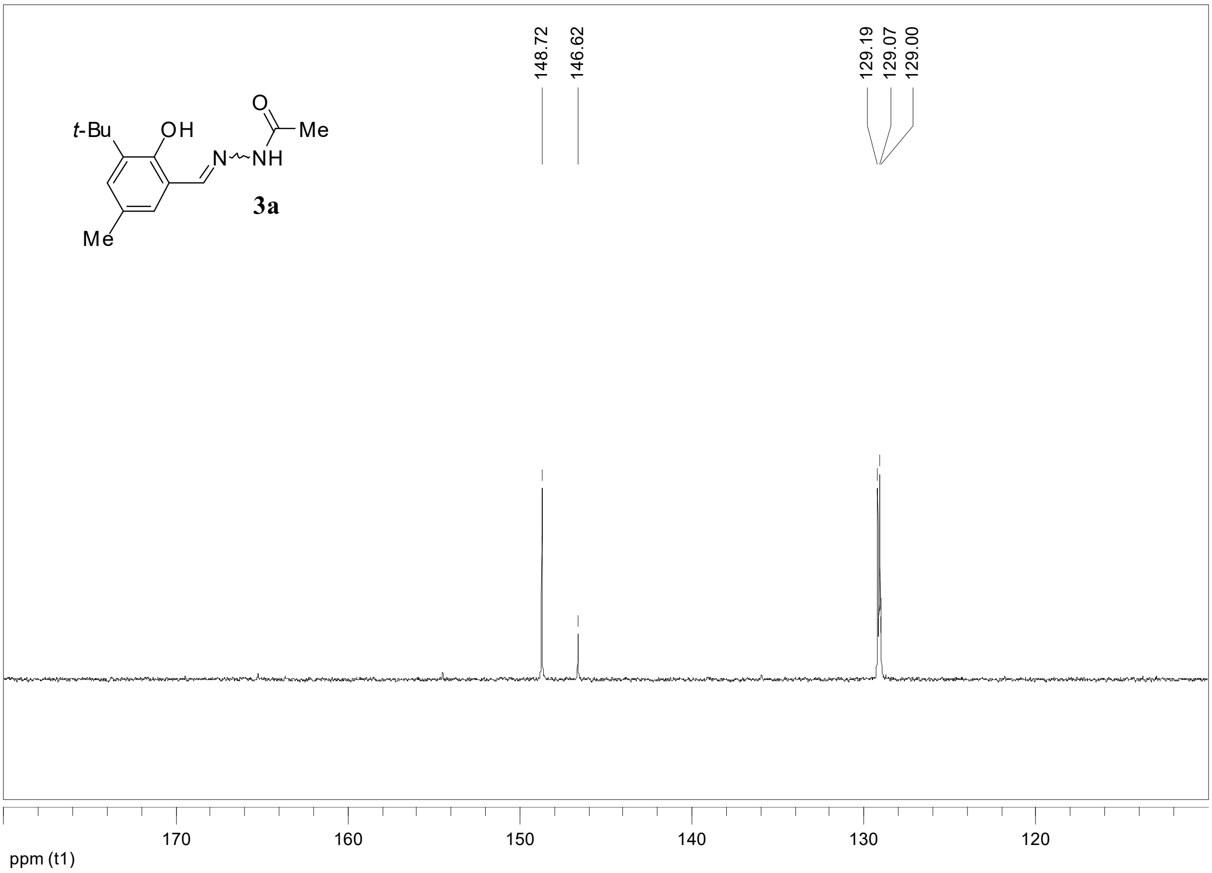

Figure S26. Expansion of ^{13}C-NMR (100 MHz, DMSO-d_6) dept-135 experiment of compound **3a**

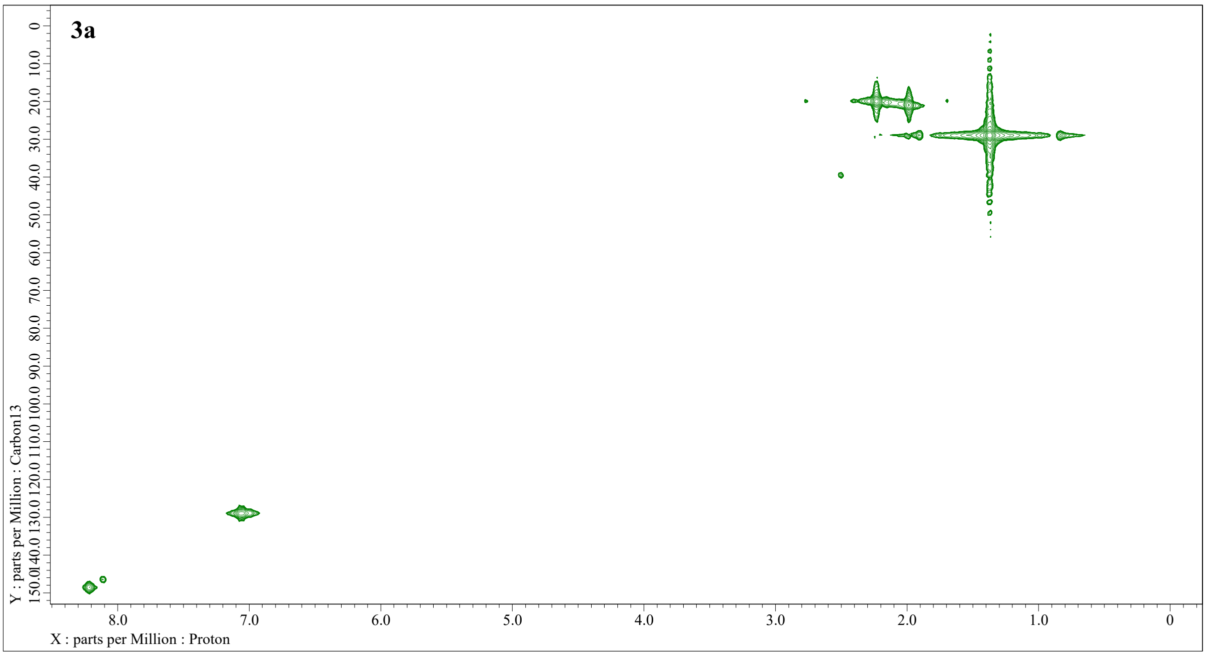

Figure S27. 2D-NMR (400 MHz, DMSO-d_6) HMQC experiment of (*E*/*Z*)-*N*'-(3-*tert*-butyl-2-hydroxy-5-methyl-benzylidene)acetohydrazide (**3a**)

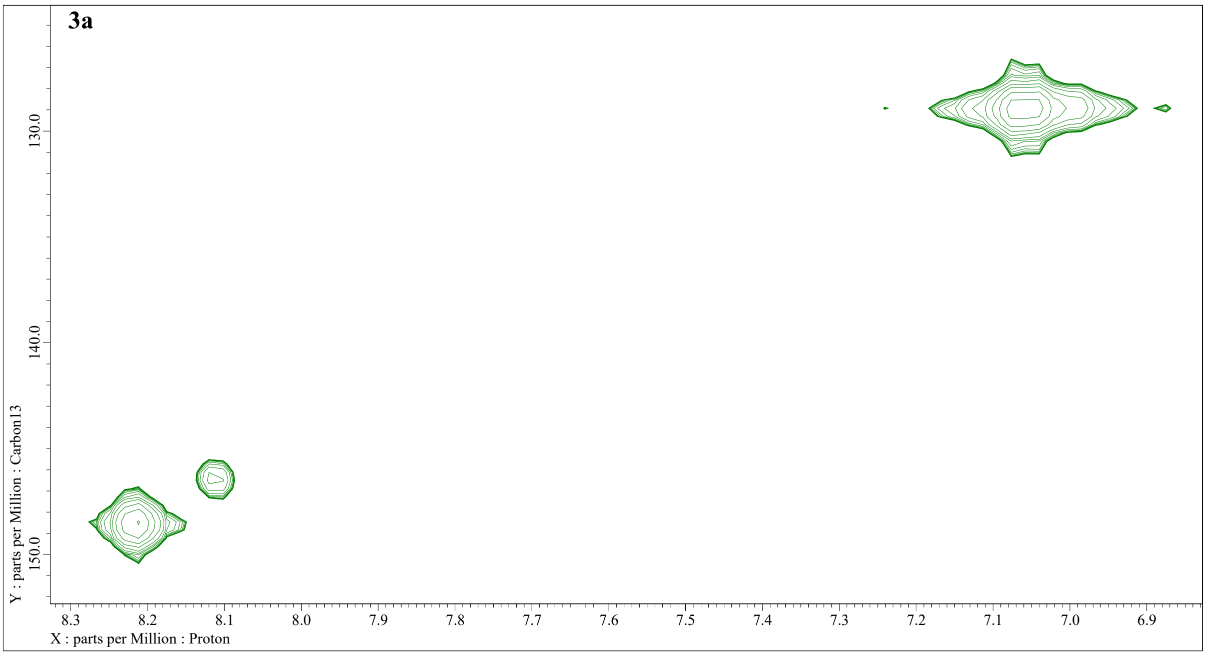

Figure S28. Expansion 2D-NMR (400 MHz, DMSO-d_6) HMQC experiment of (*E*/*Z*)-*N*'-(3-*tert*-butyl-2-hydroxy-5-methyl-benzylidene)acetohydrazide (**3a**)

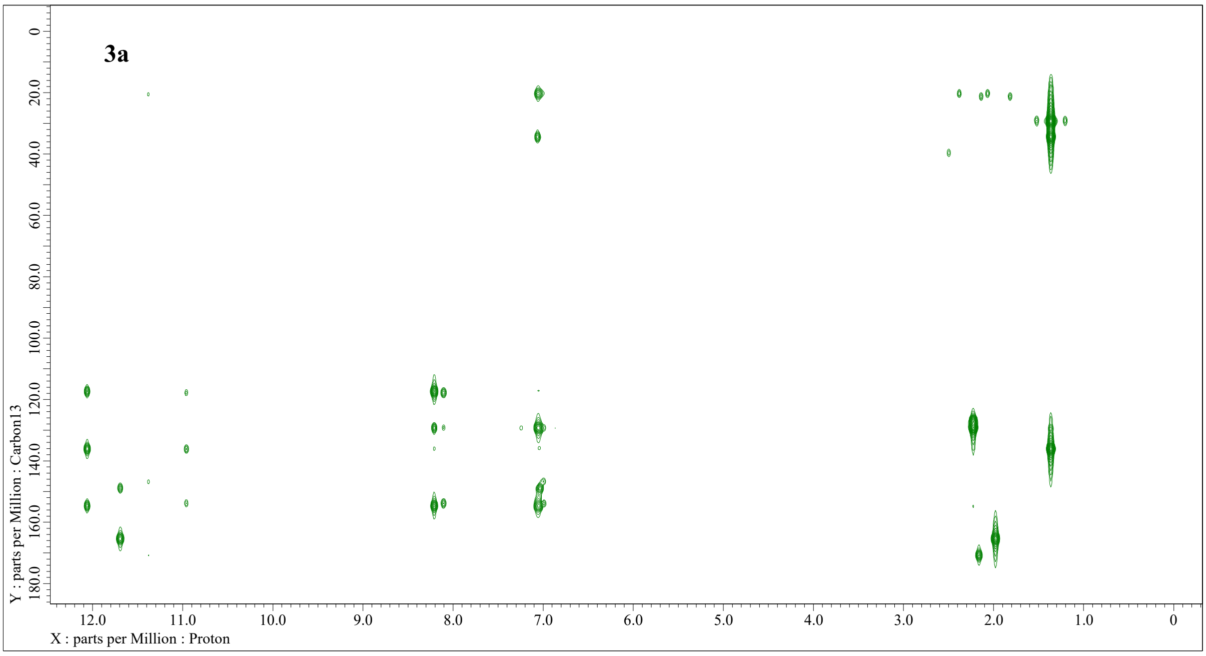

Figure S29. 2D-NMR (400 MHz, DMSO-d_6) HMBC experiment of (*E*/*Z*)-*N*'-(3-*tert*-butyl-2-hydroxy-5-methyl-benzylidene)acetohydrazide (**3a**)

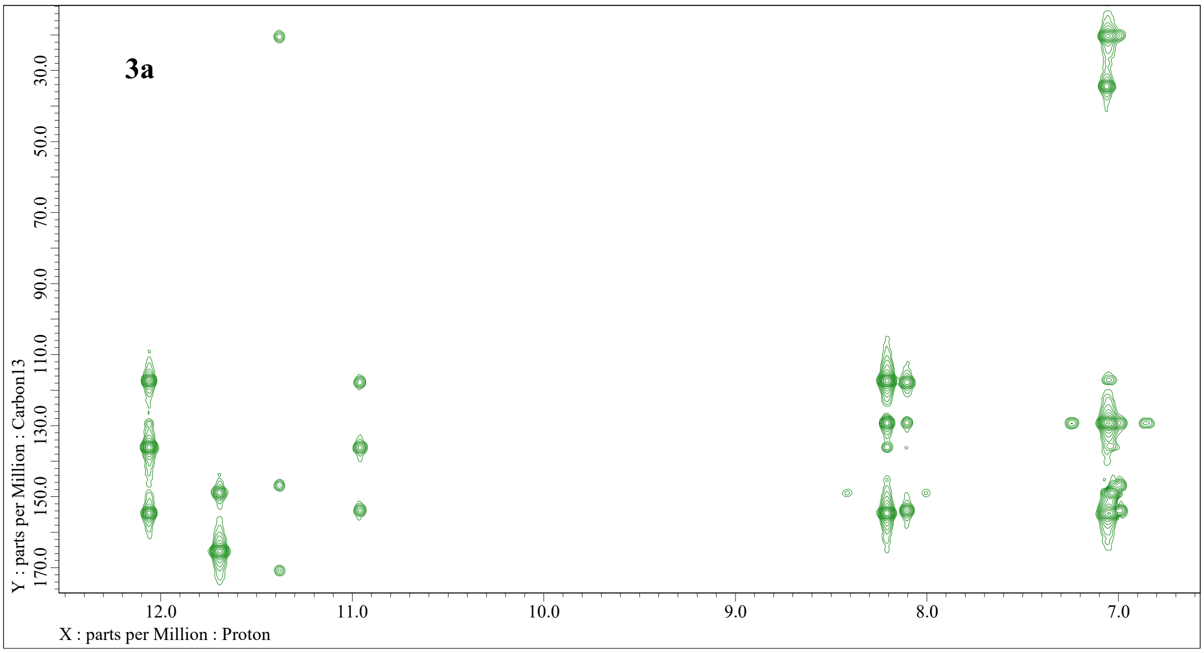

Figure S30. Expansion 2D-NMR (400 MHz, DMSO-d_6) HMBC experiment of (*E*/*Z*)-*N*'-(3-*tert*-butyl-2-hydroxy-5-methyl-benzylidene)acetohydrazide (**3a**)

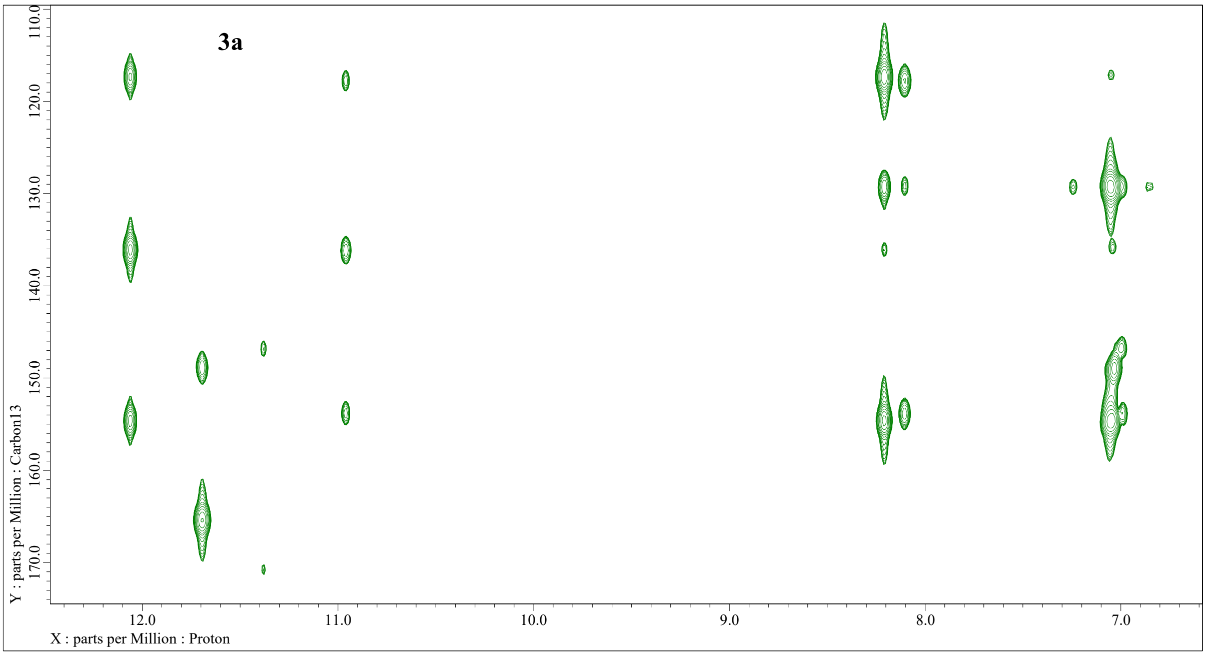

Figure S31. Expansion of 2D-NMR (400 MHz, DMSO-d_6) HMBC experiment of (*E*/*Z*)-*N*'-(3-*tert*-butyl-2-hydroxy-5-methyl-benzylidene)acetohydrazide (**3a**)

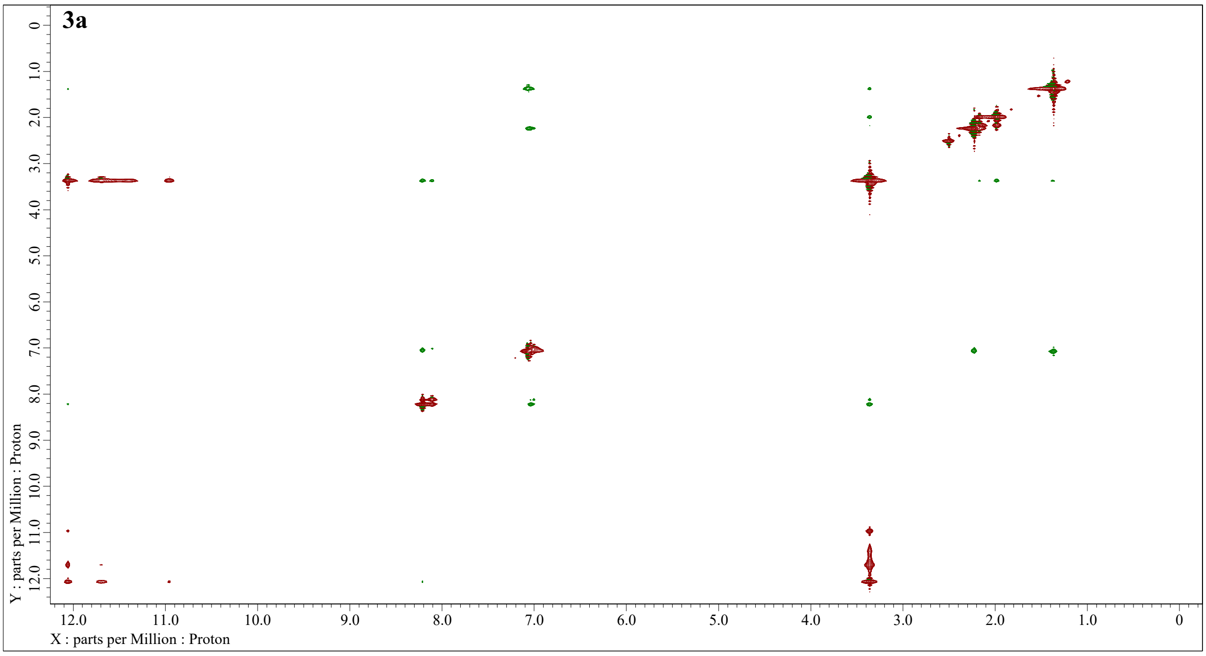

Figure S32. ^{1}H-^{1}H NMR (400MHz, DMSO-d_6) NOESY experiment of (*E*/*Z*)-*N*'-(3-*tert*-butyl-2-hydroxy-5-methyl-benzylidene)acetohydrazide (**3a**)

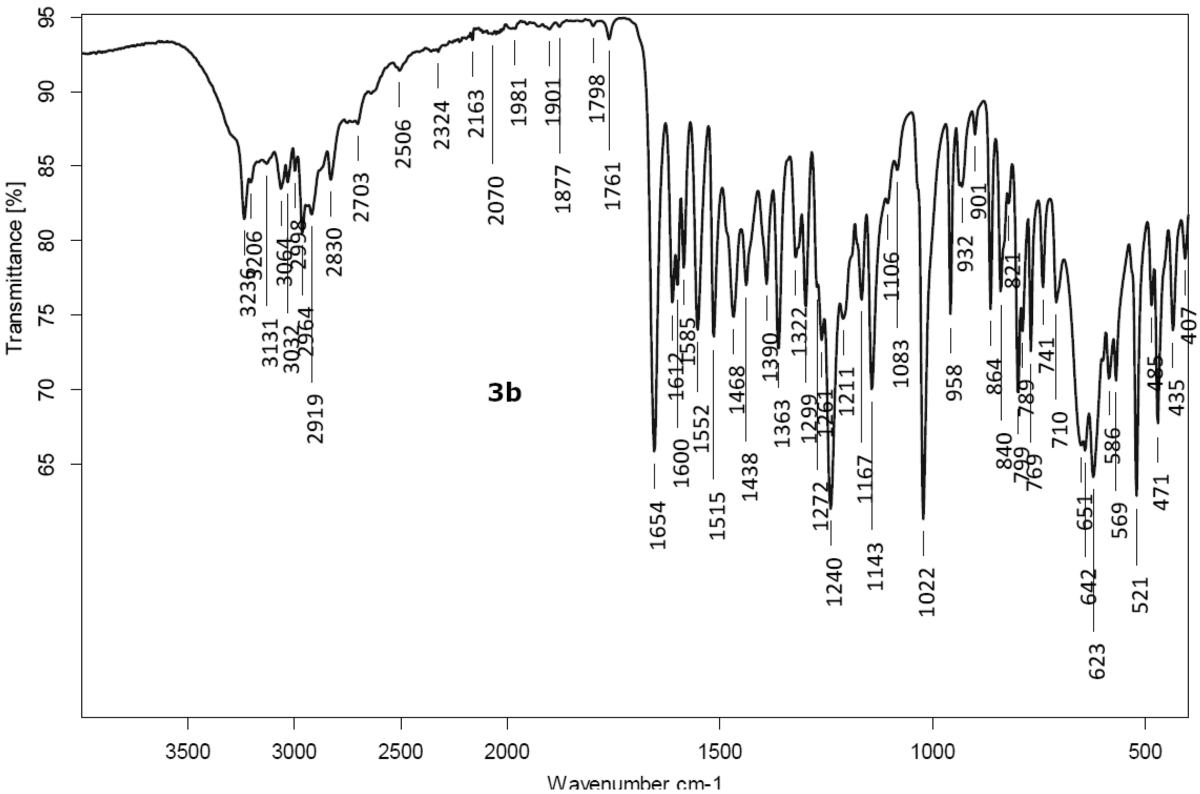

Figure S33. FT-IR (ATR) spectrum of 2-(4-hydroxyphenyl)-*N*'-[(*E*)-(3-*tert*-butyl-2-hydroxy-5-methylphenyl)methylidene]acetohydrazide (**3b**)

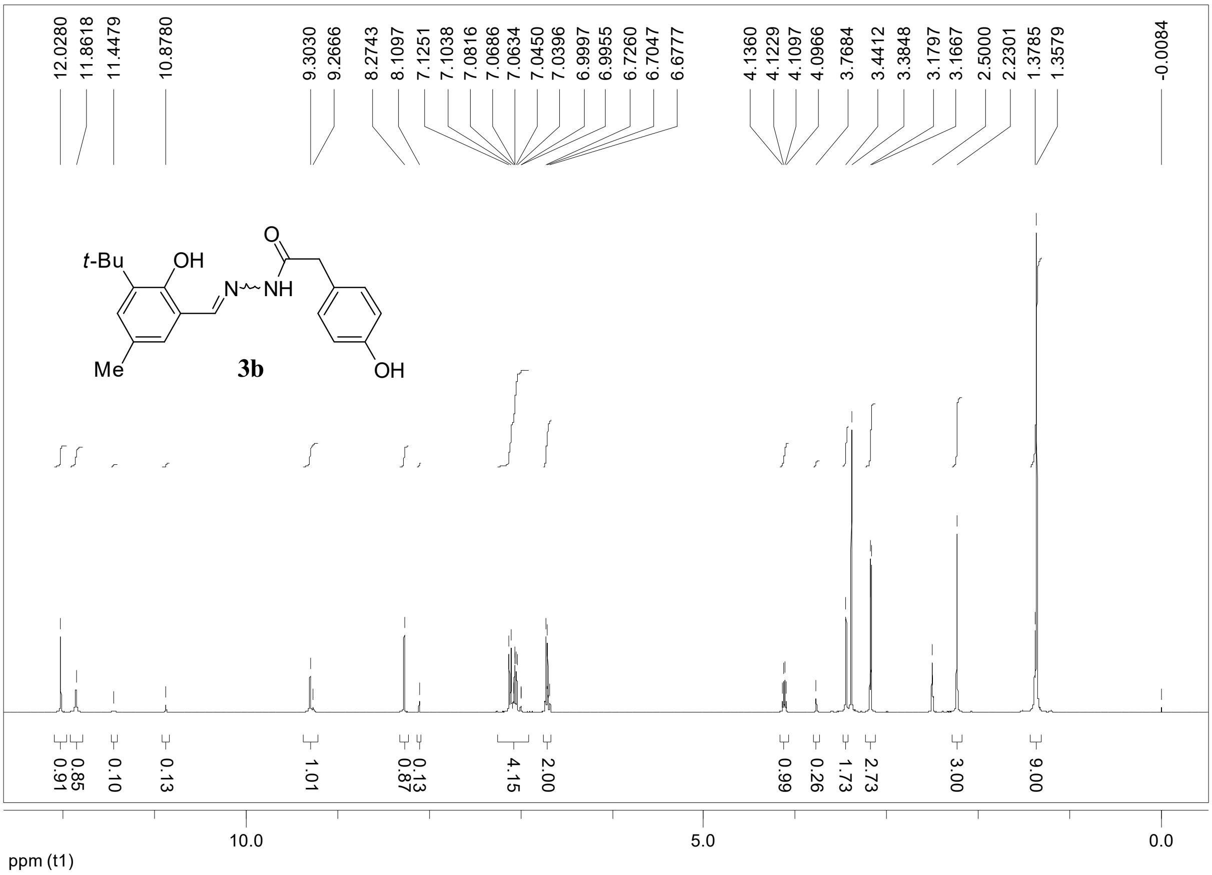

Figure S34. ^{1}H-NMR (400 MHz, DMSO-d_6) spectrum of compound **3b**

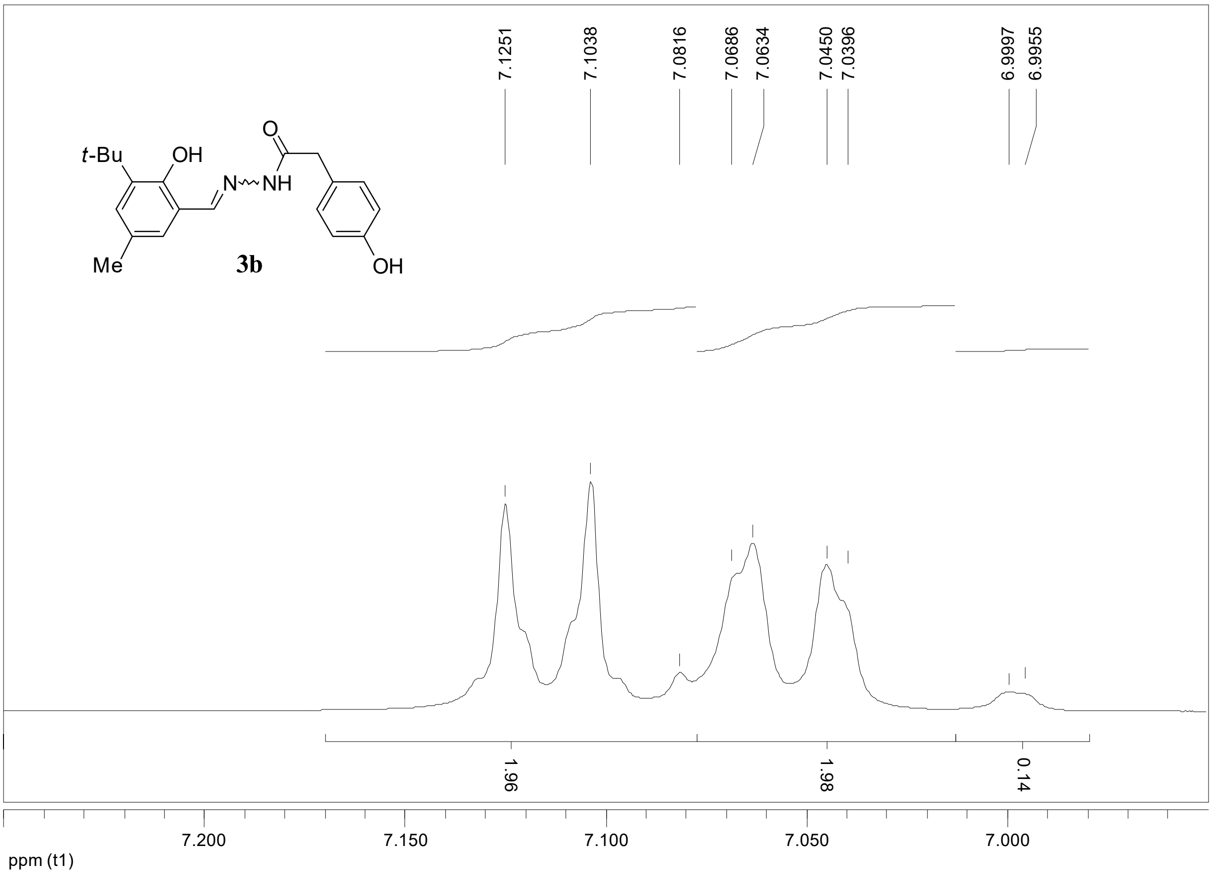

Figure S35. Expansion of ^{1}H-NMR (400 MHz, DMSO-d_6) spectrum of compound **3b**

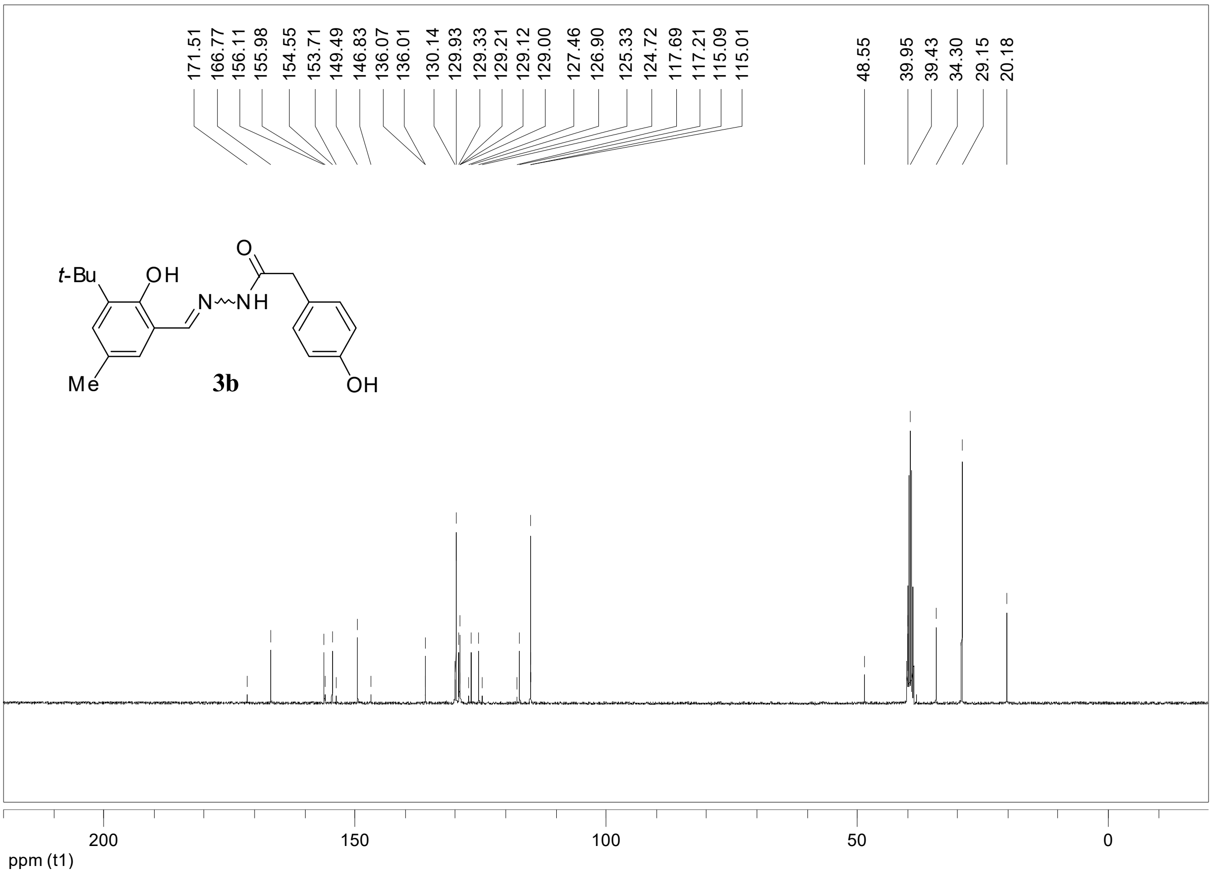

Figure S36. ^{13}C-NMR (100 MHz, DMSO-d_6) spectrum of compound **3b**

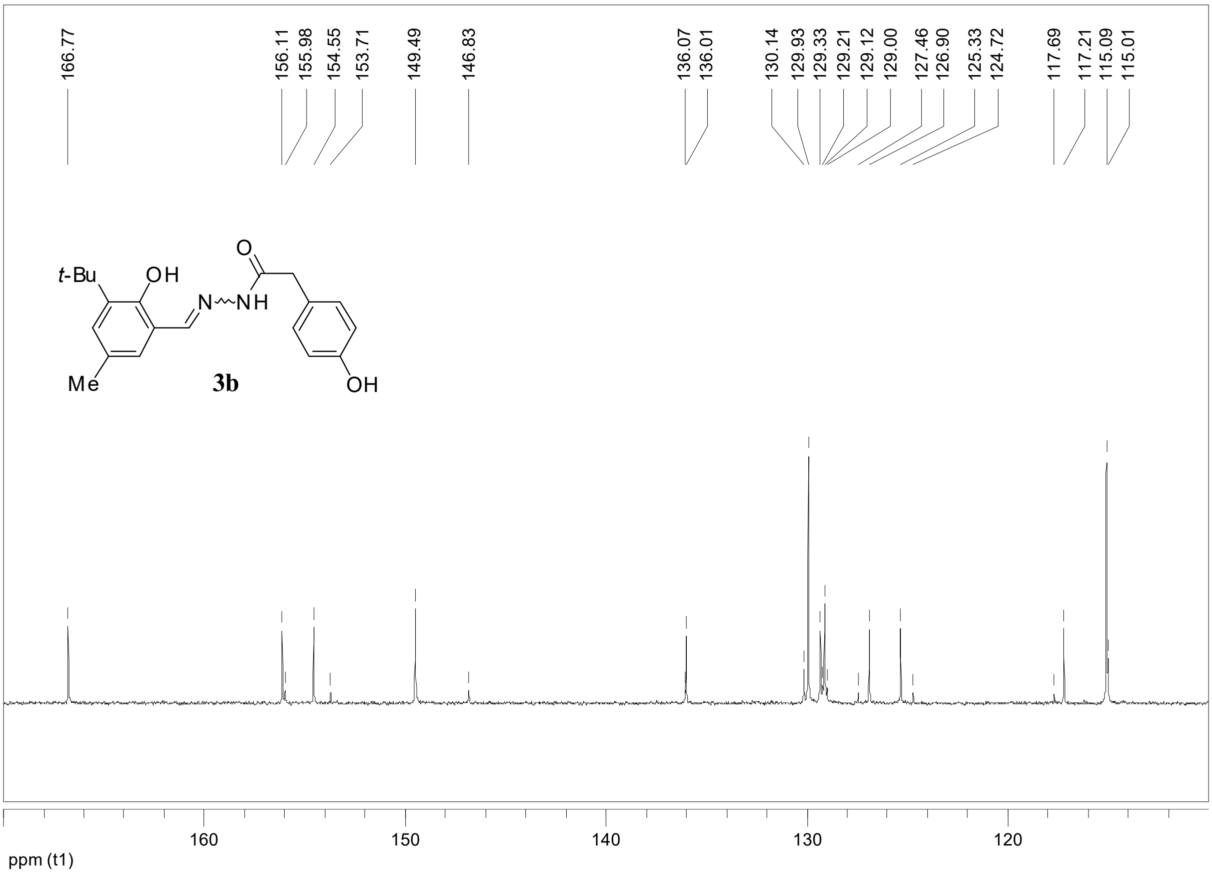

Figure S37. Expansion of ^{13}C-NMR (100 MHz, DMSO-d_6) spectrum of compound **3b**

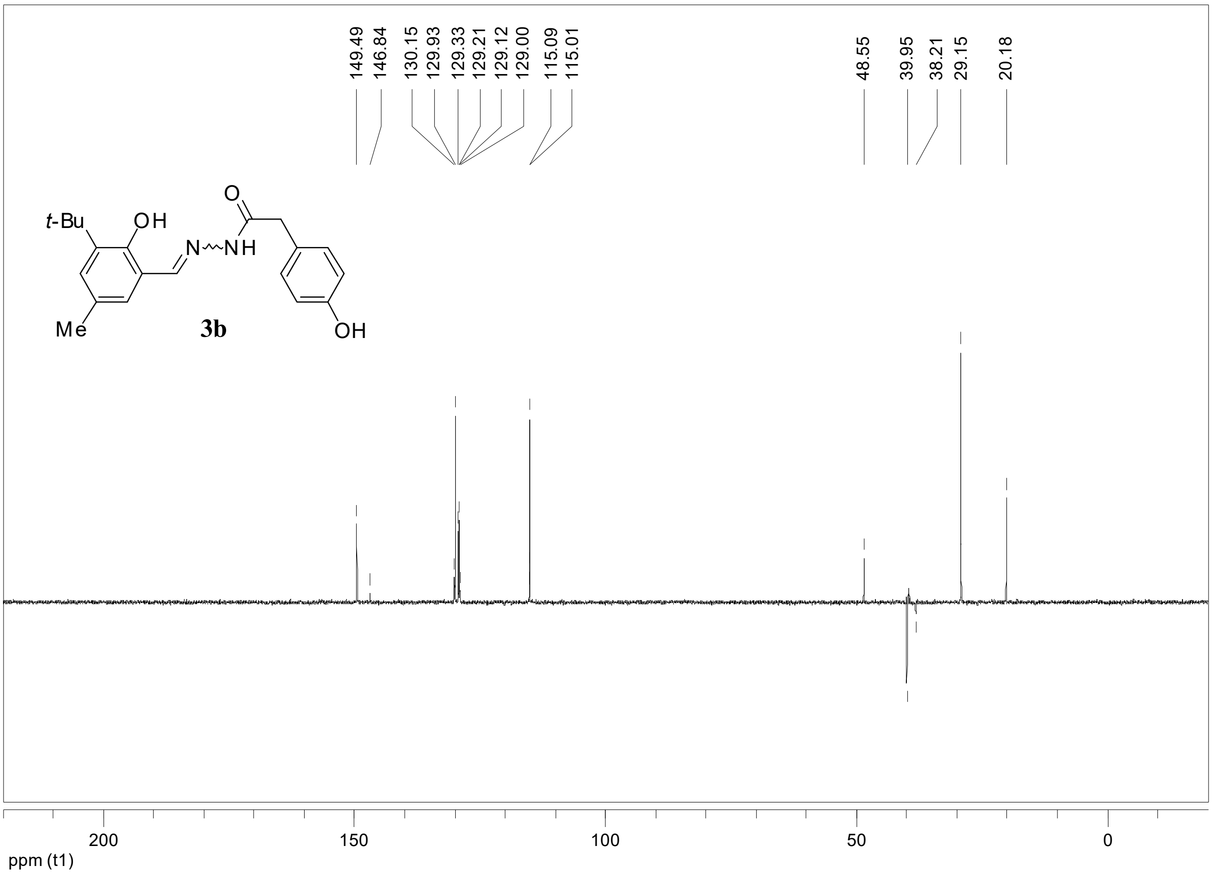

Figure S38. ^{13}C-NMR (100 MHz, DMSO-d_6) dept-135 experiment of compound **3b**

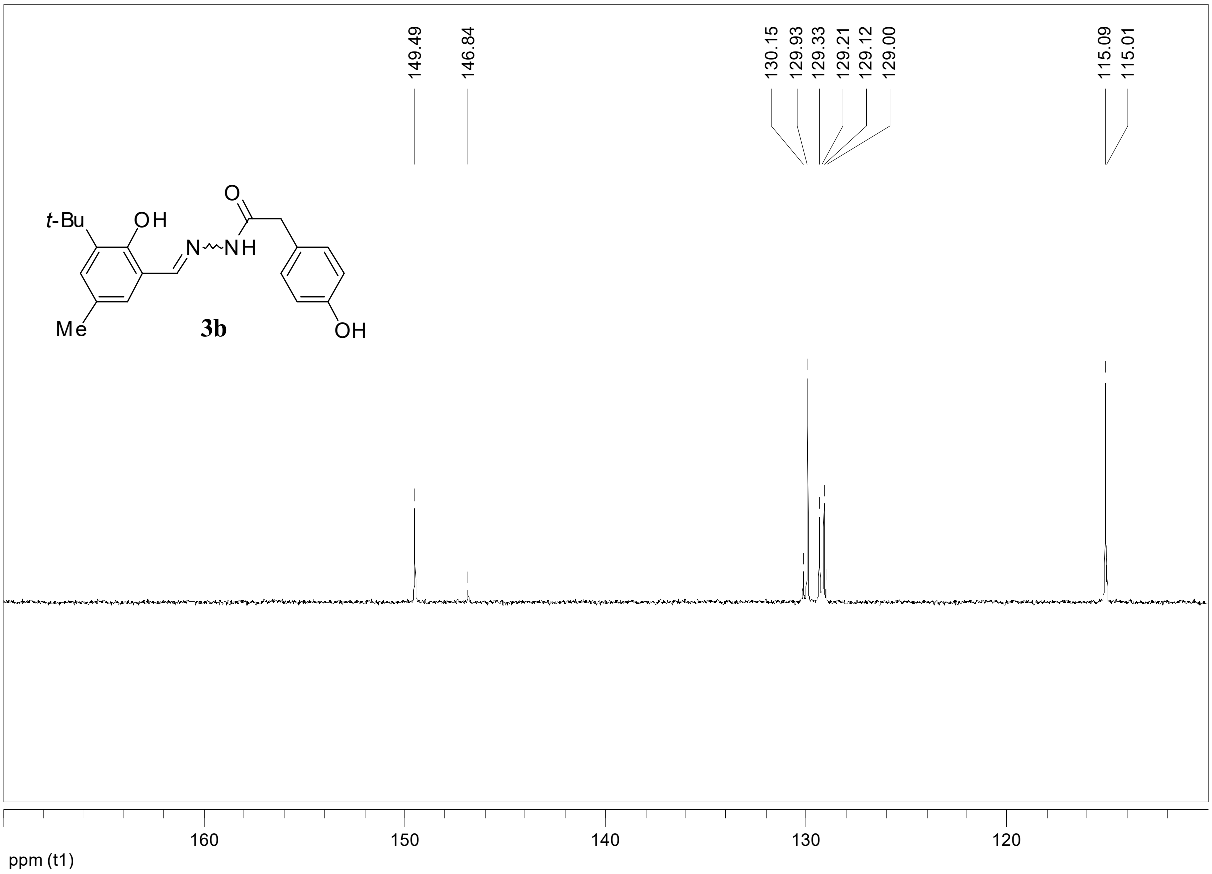

Figure S39. Expansion of ^{13}C-NMR (100 MHz, DMSO-d_6) dept-135 experiment of compound **3b**

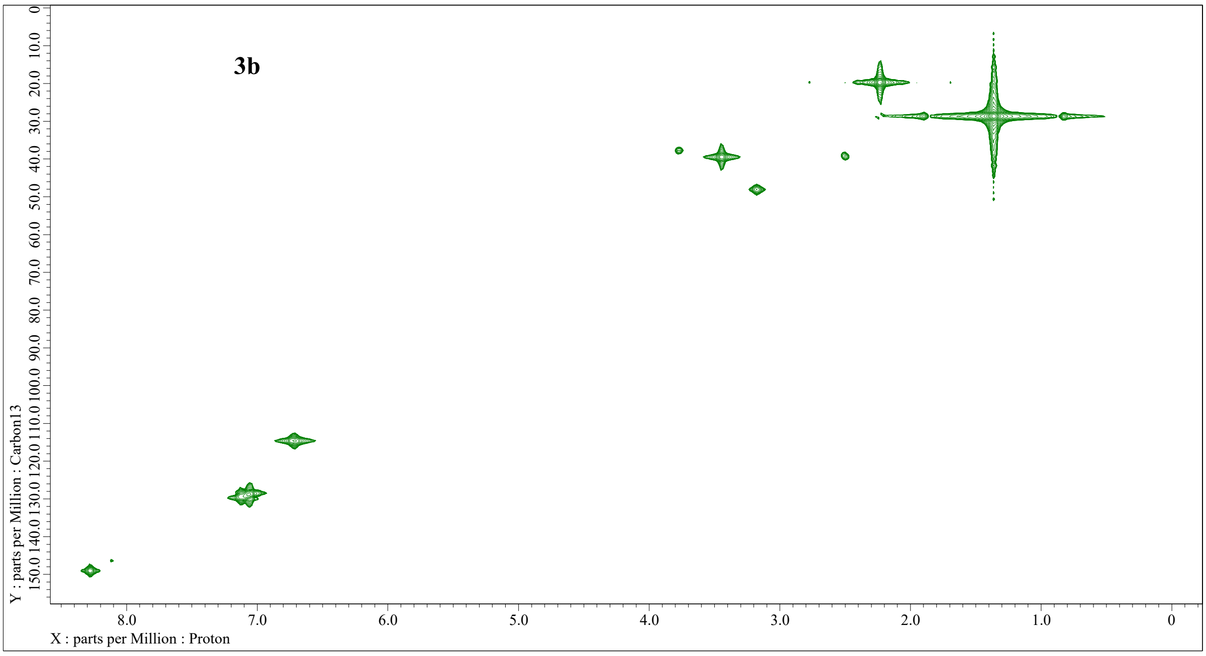

Figure S40. 2D-NMR (400 MHz, DMSO-d_6) HMQC experiment of 2-(4-hydroxyphenyl)-*N*'-[(*E*)-(3-*tert*-butyl-2-hydroxy-5-methylphenyl)methylidene]acetohydrazide (**3b**)

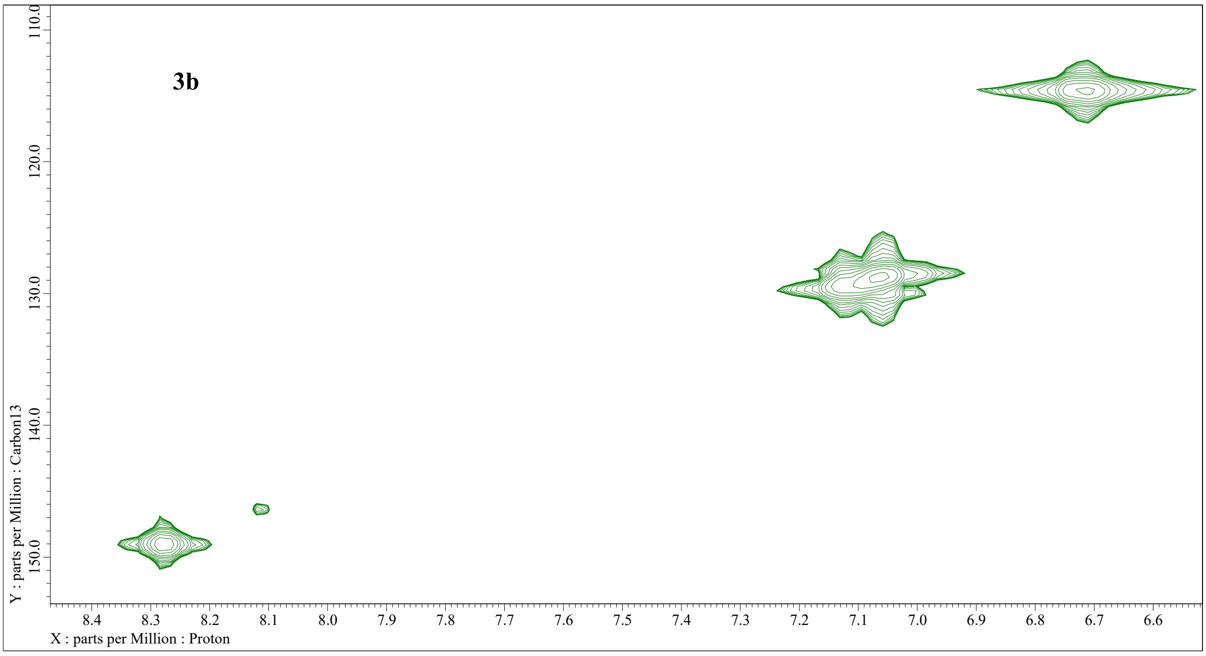

Figure S41. Expansion of 2D-NMR (400 MHz, DMSO-d_6) HMQC experiment of 2-(4-hydroxyphenyl)-*N*'-[(*E*)-(3-*tert*-butyl-2-hydroxy-5-methylphenyl)methylidene]acetohydrazide (**3b**)

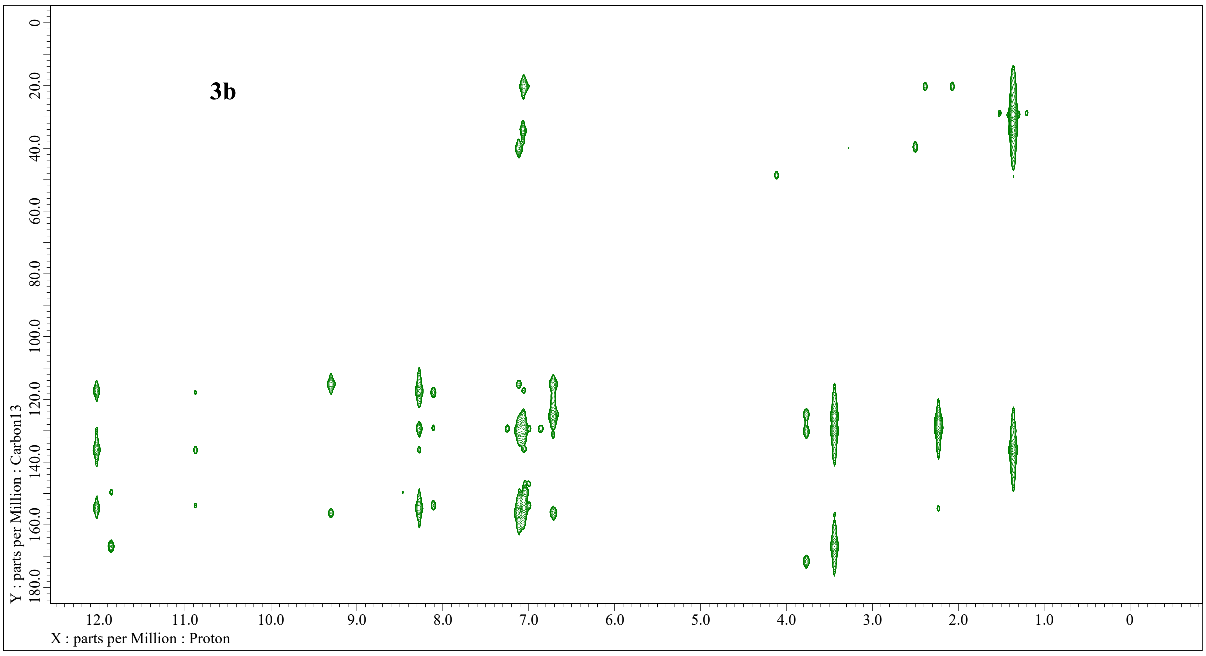

Figure S42. 2D-NMR (400 MHz, DMSO-d_6) HMBC experiment of 2-(4-hydroxyphenyl)-*N*'-[(*E*)-(3-*tert*-butyl-2-hydroxy-5-methylphenyl)methylidene]acetohydrazide (**3b**)

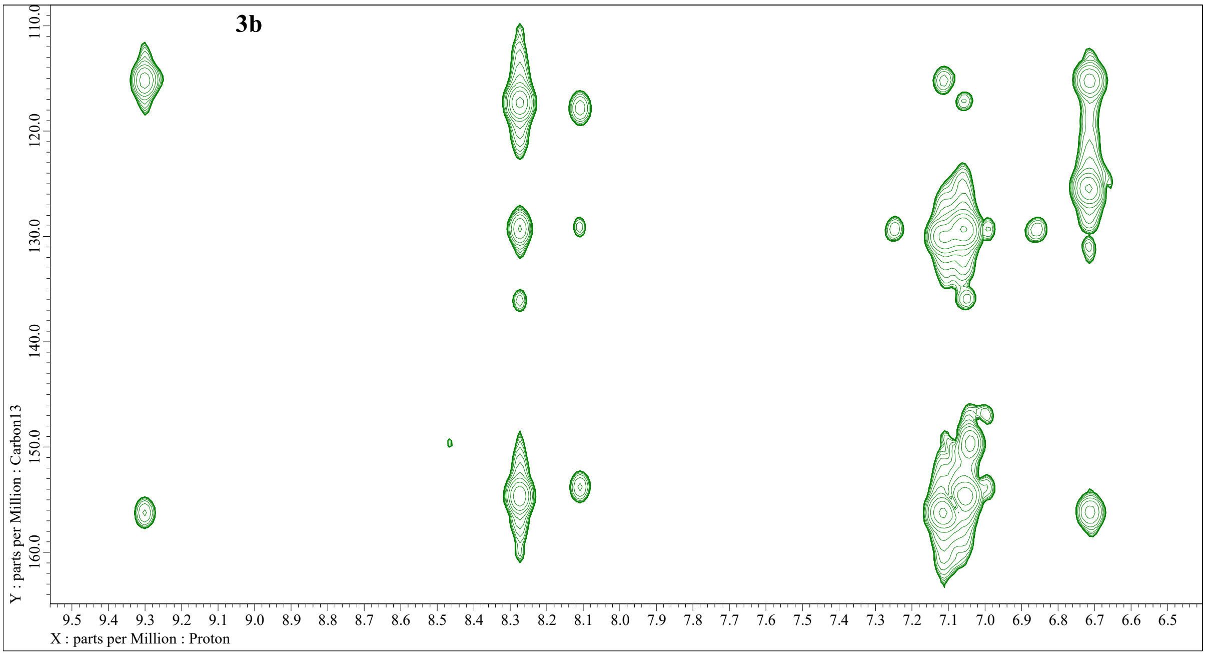

Figure S43. Expansion of 2D-NMR (400 MHz, DMSO-d_6) HMBC experiment of 2-(4-hydroxyphenyl)-*N*'-[(*E*)-(3-*tert*-butyl-2-hydroxy-5-methylphenyl)methylidene]acetohydrazide (**3b**)

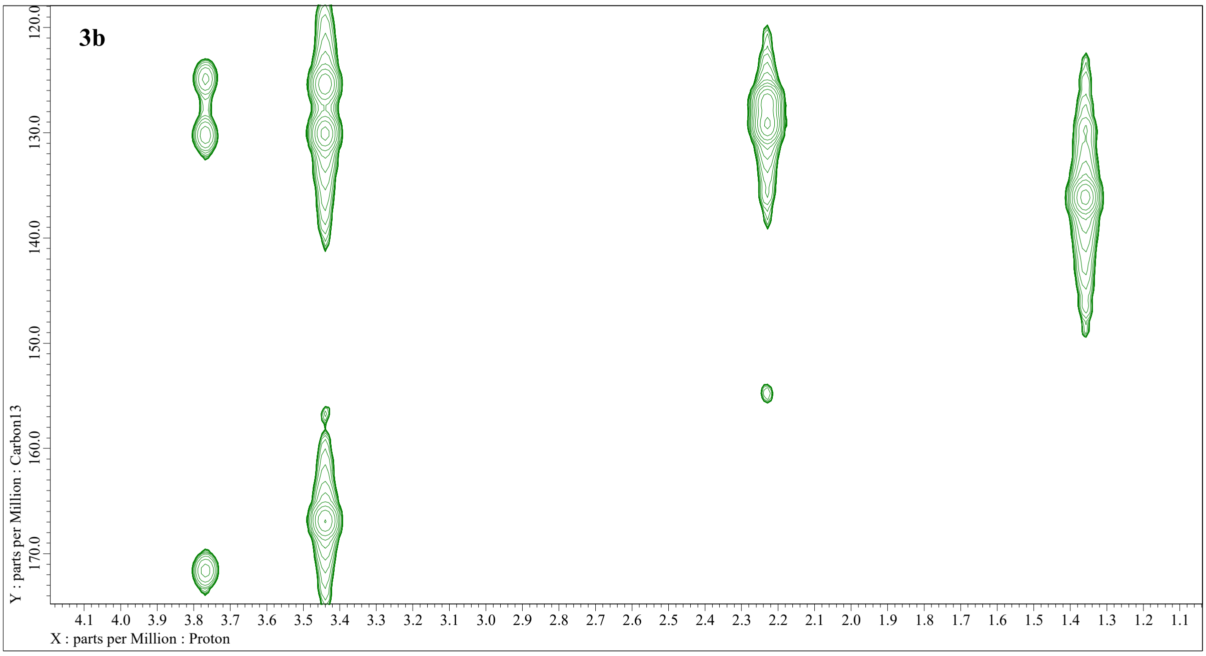

Figure S44. Expansion of 2D-NMR (400 MHz, DMSO-d_6) HMBC experiment of 2-(4-hydroxyphenyl)-*N'*-[(*E*)-(3-*tert*-butyl-2-hydroxy-5-methylphenyl)methylidene]acetohydrazide (**3b**)

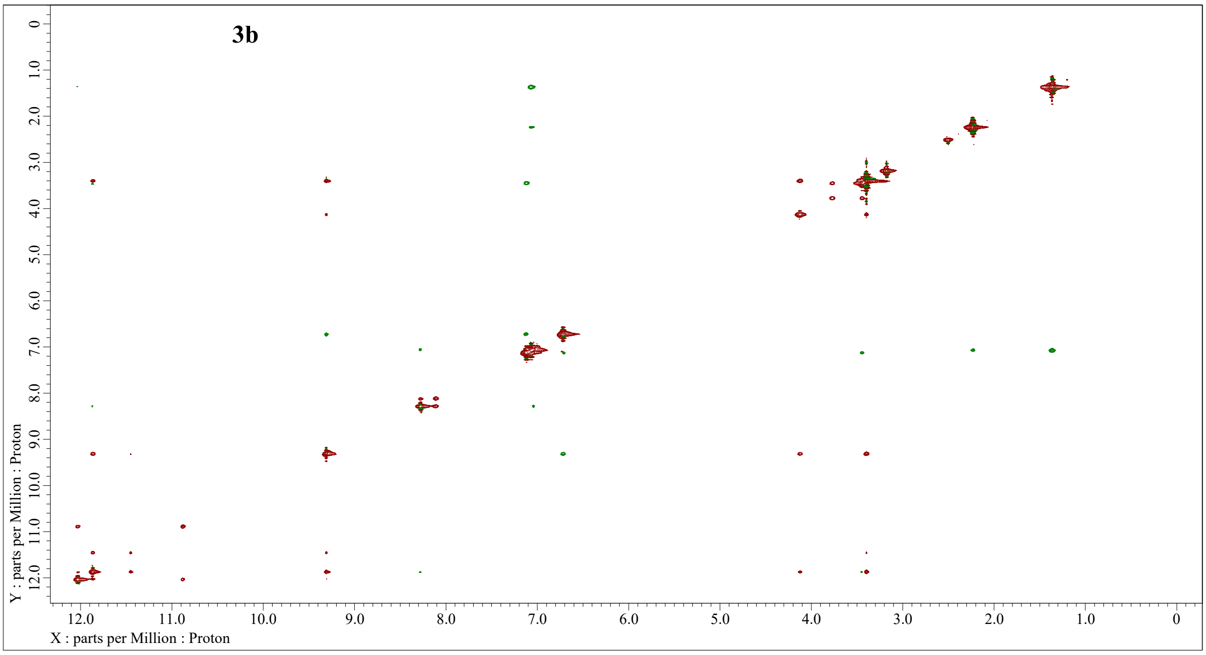

Figure S45. ^{1}H-^{1}H NMR (400 MHz, DMSO-d_6) NOESY experiment of 2-(4-hydroxyphenyl)-*N'*-[(*E*)-(3-*tert*-butyl-2-hydroxy-5-methylphenyl)methylidene]acetohydrazide (**3b**)

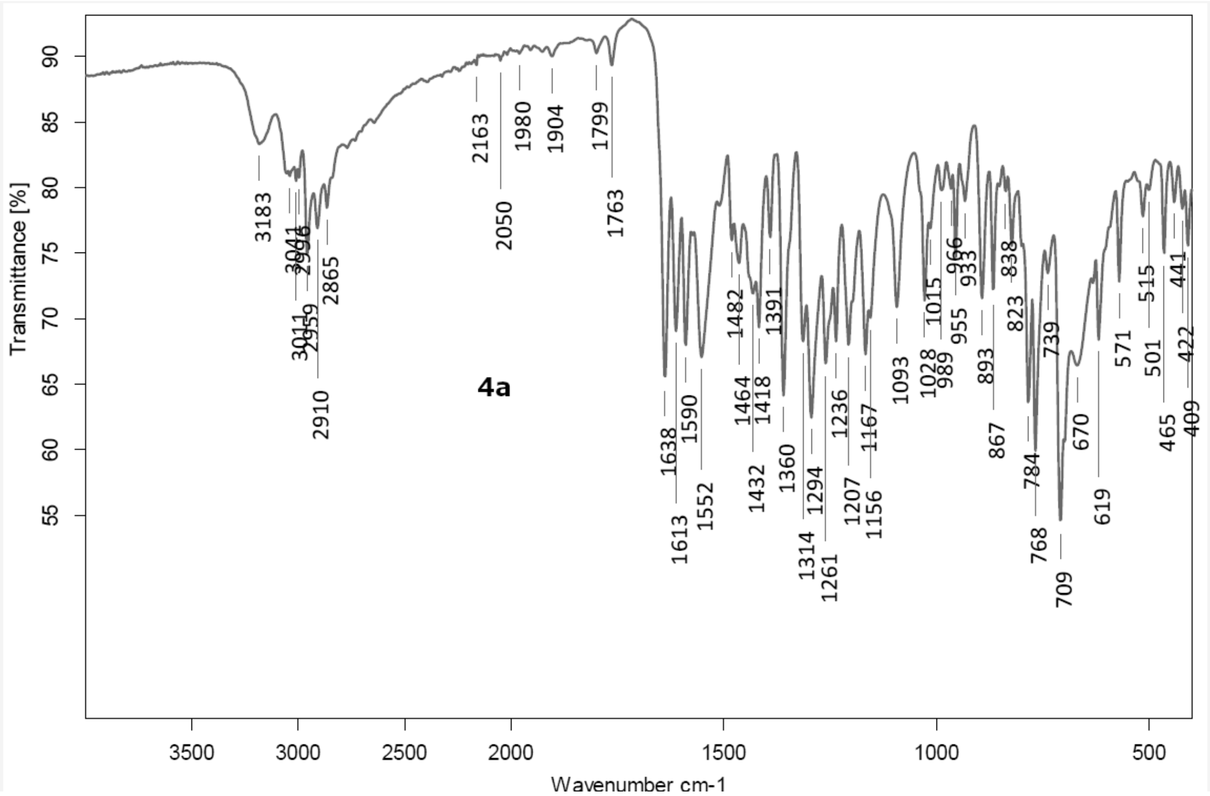

Figure S46. FT-IR (ATR) spectrum of *N*'-[(*E*)-(3-*tert*-butyl-2-hydroxy-5-methylphenyl)methylidene]-3-pyridohydrazide (**4a**)

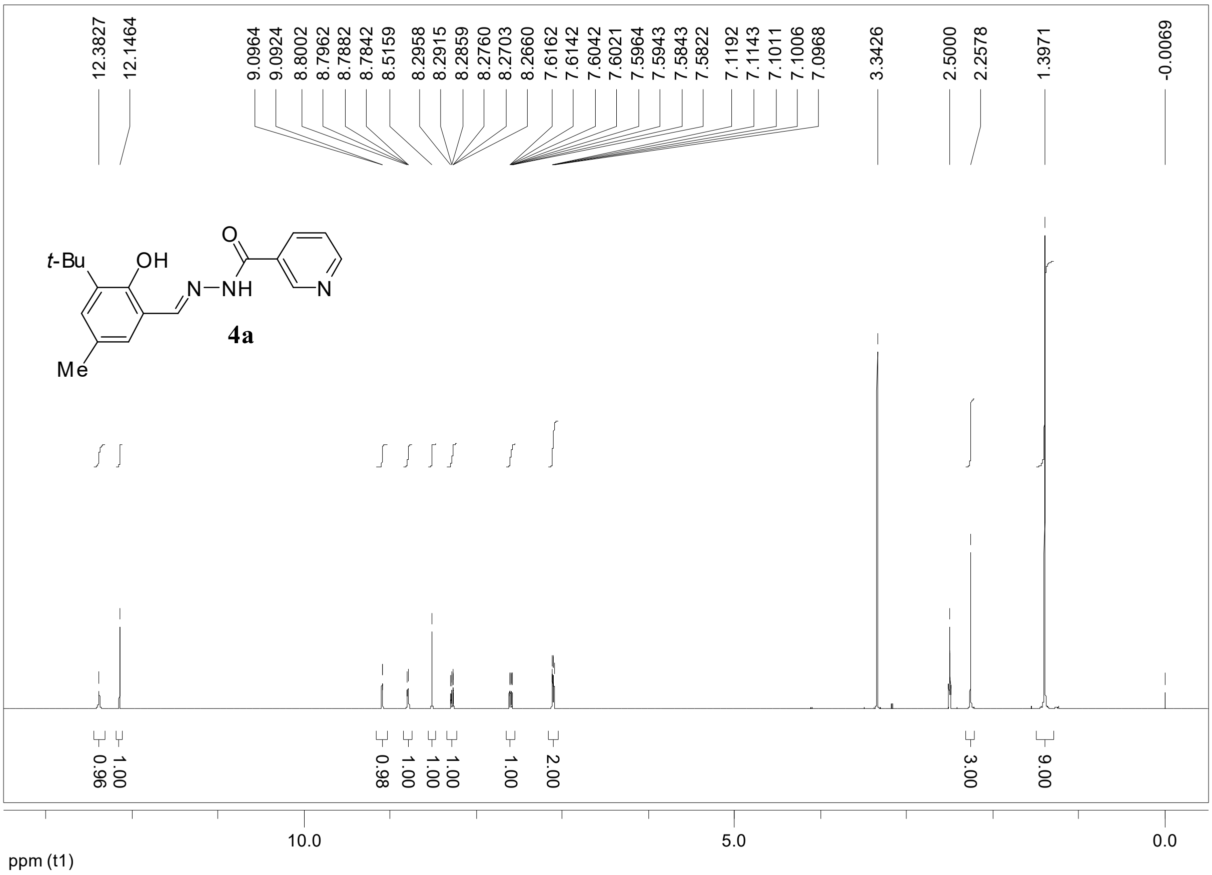

Figure S47. ^{1}H-NMR (400 MHz, DMSO-d_6) spectrum of compound **4a**

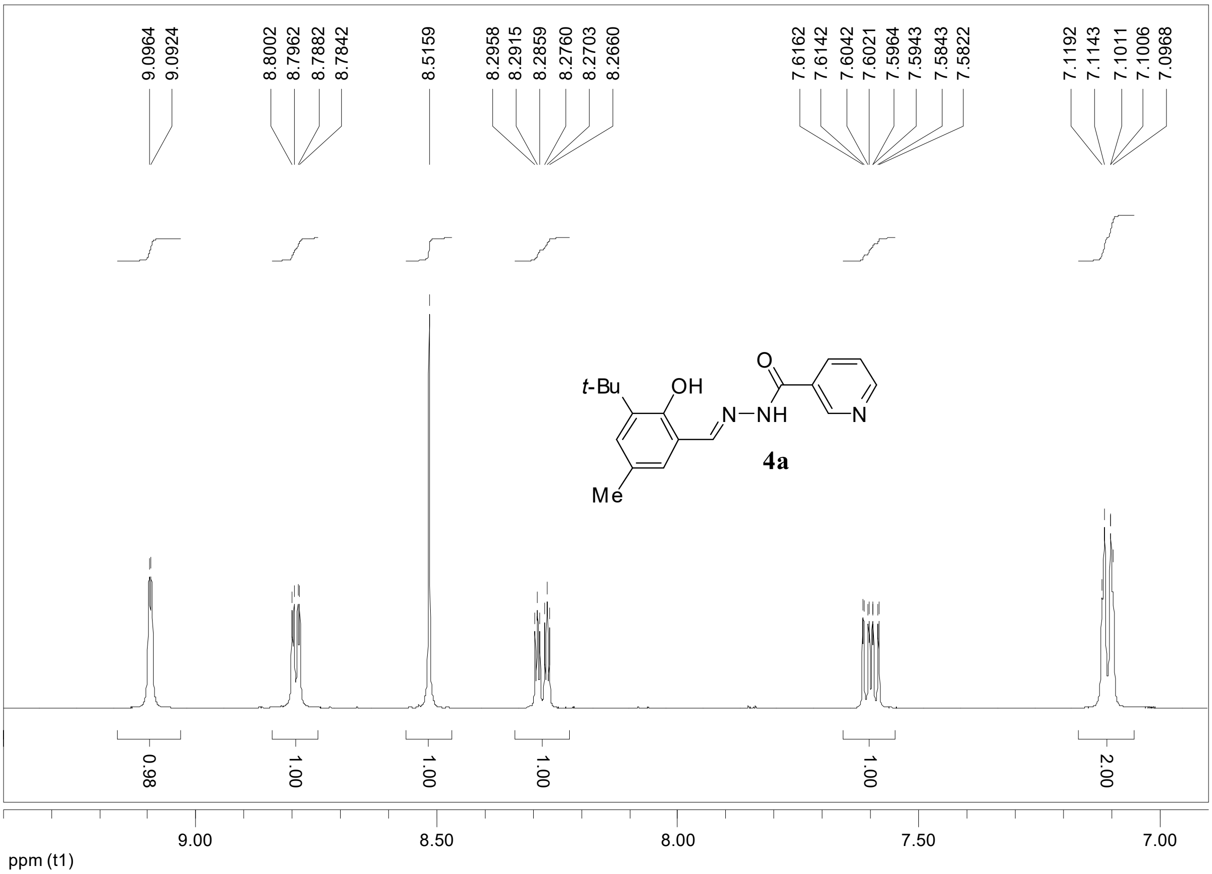

Figure S48. Expansion of ^{1}H-NMR (400 MHz, DMSO-d_6) spectrum of compound **4a**

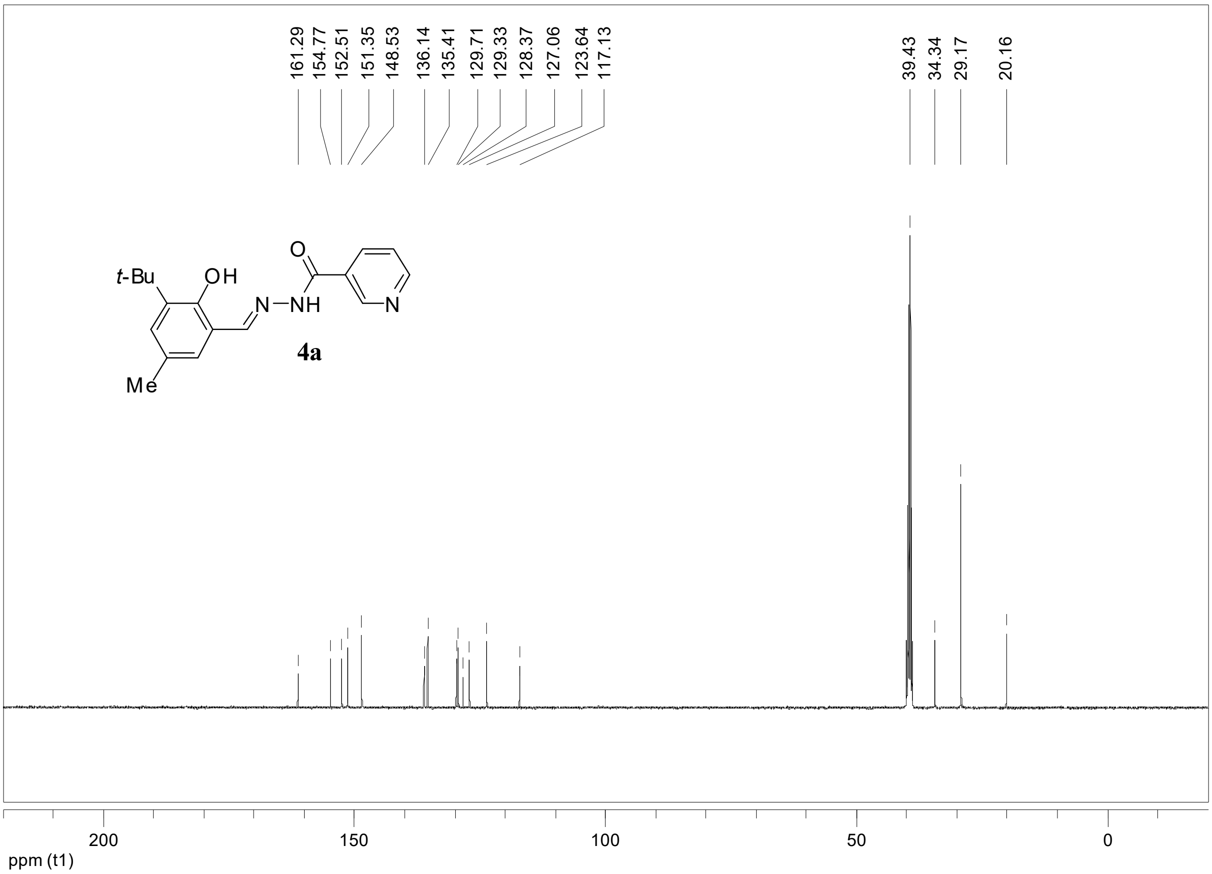

Figure S49. ^{13}C-NMR (100 MHz, DMSO-d_6) spectrum of compound **4a**

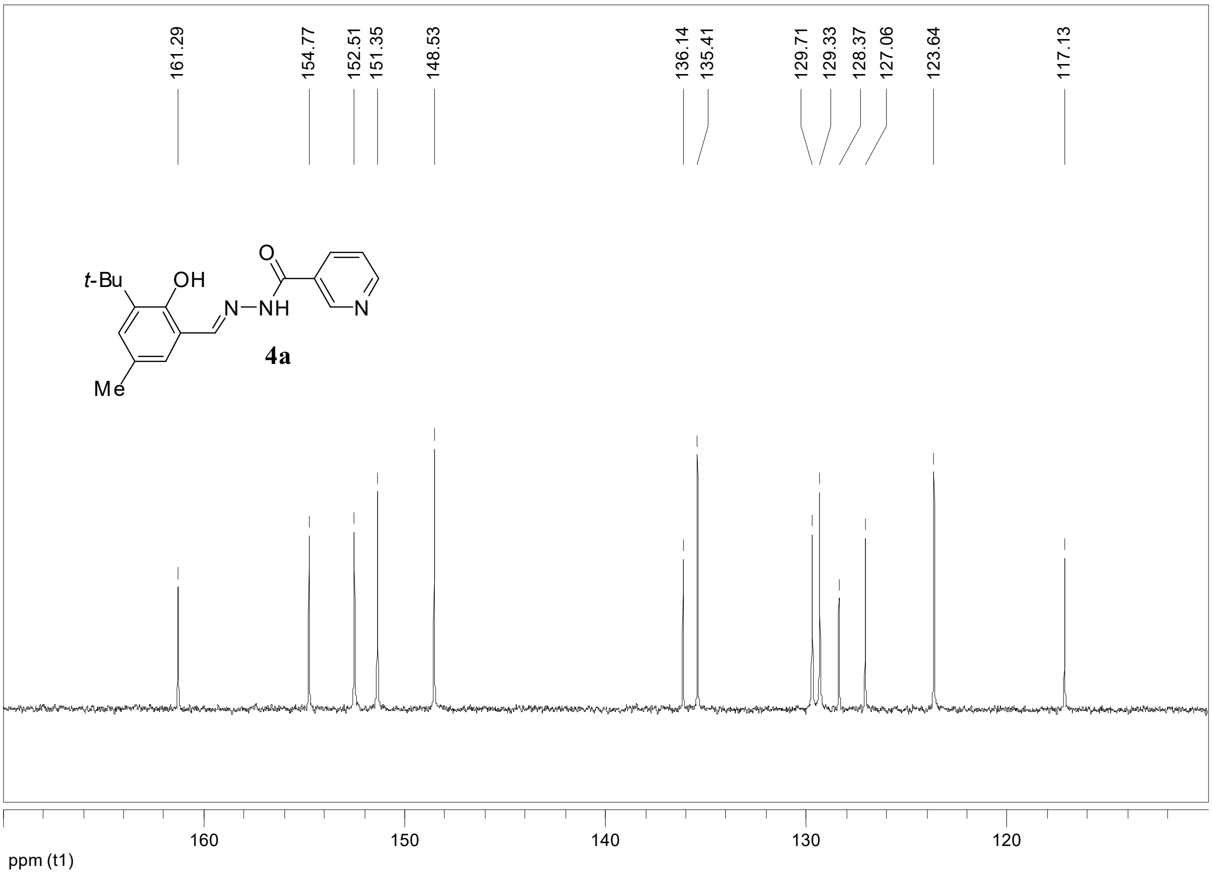

Figure S50. Expansion of ^{13}C-NMR (100 MHz, DMSO-d_6) spectrum of compound **4a**

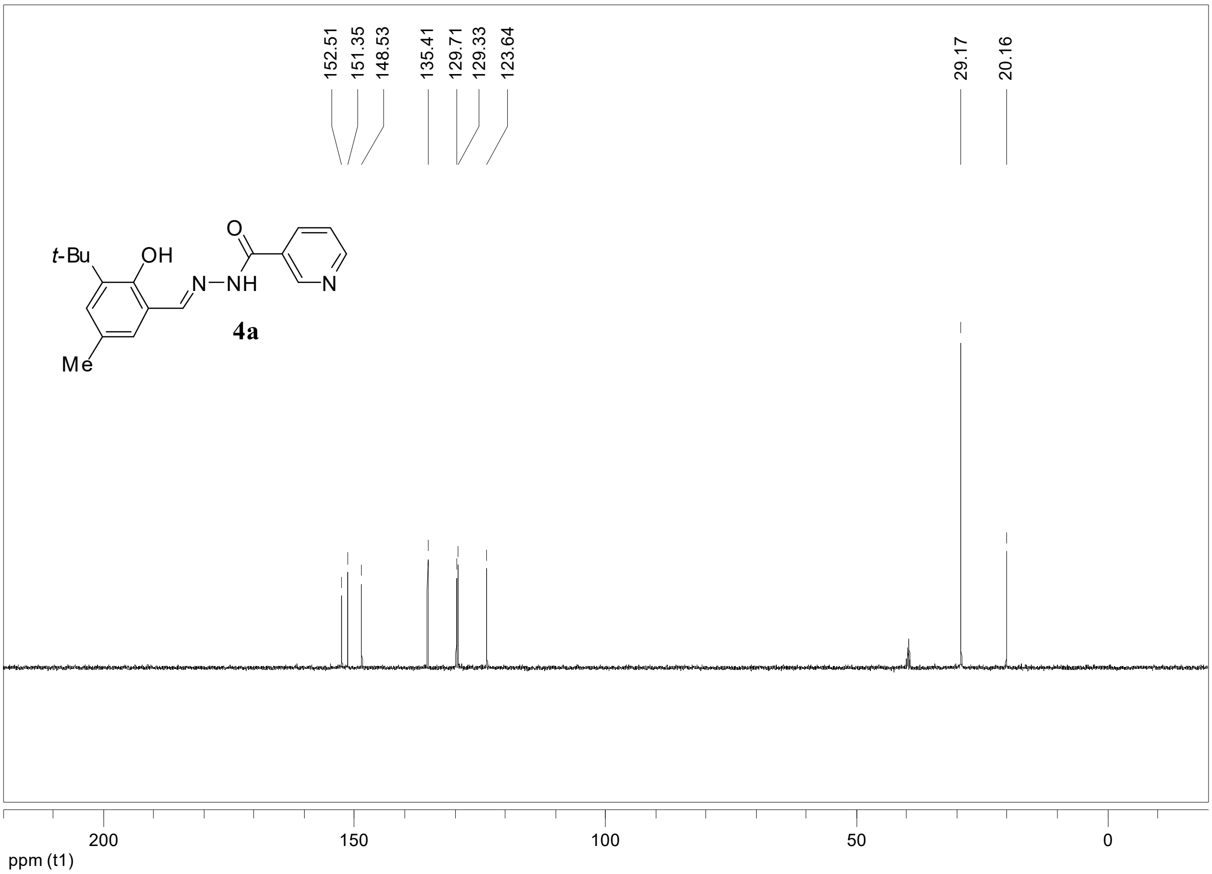

Figure S51. ^{13}C-NMR (100 MHz, DMSO-d_6) dept-135 experiment of compound **4a**

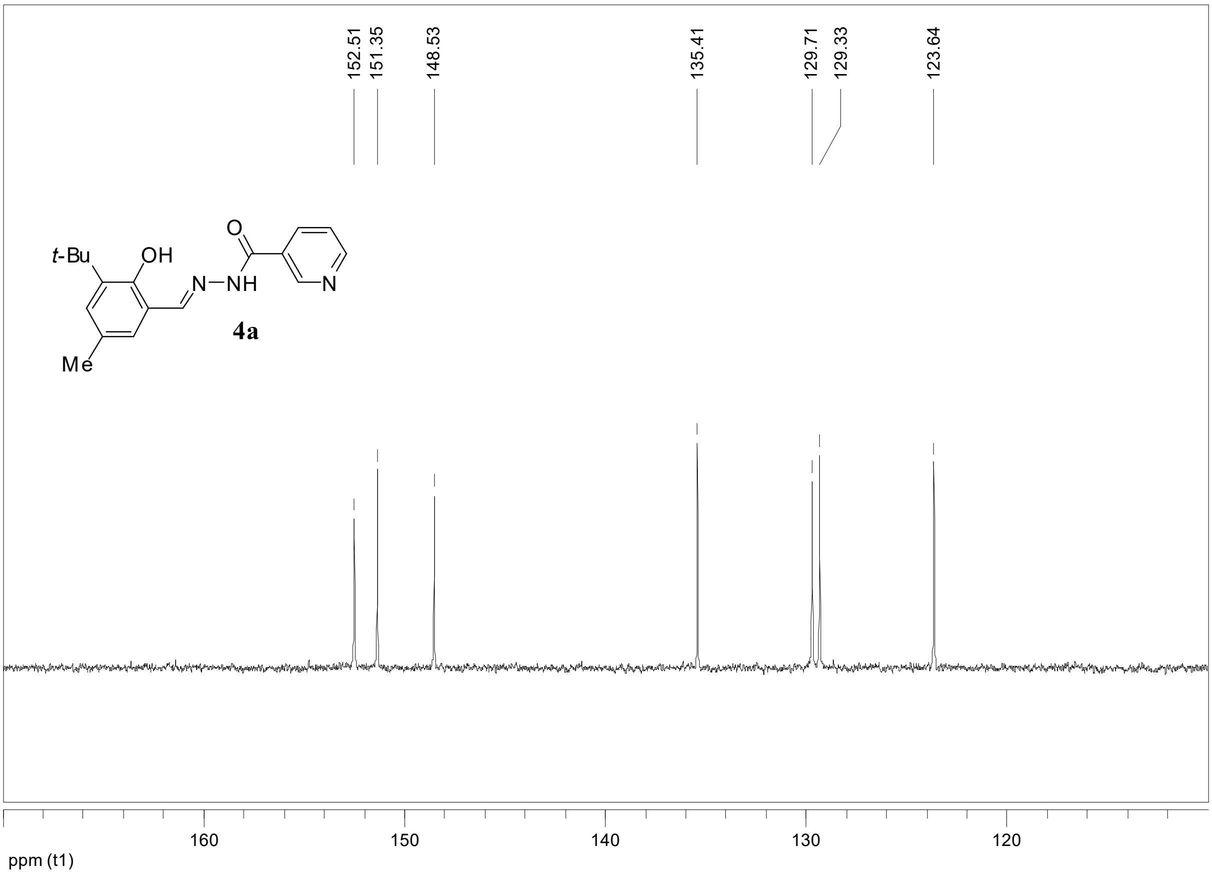

Figure S52. Expansion of ^{13}C-NMR (100 MHz, DMSO-d_6) dept-135 experiment of compound **4a**

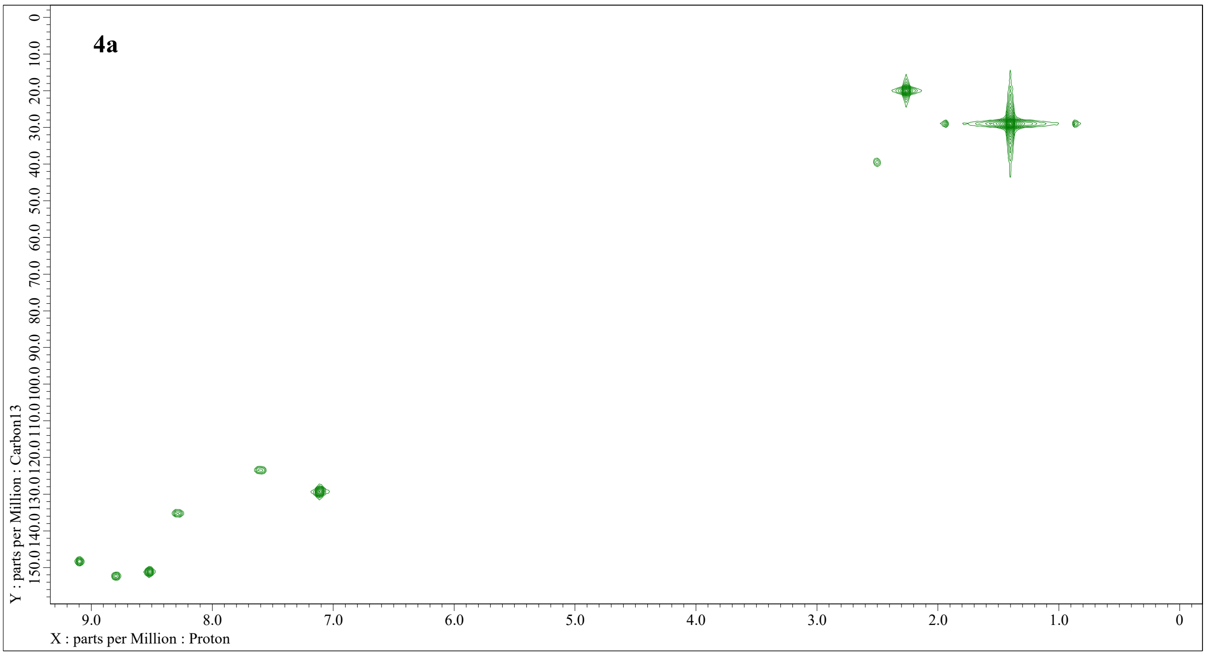

Figure S53. 2D-NMR (400 MHz, DMSO-d_6) HMQC experiment of *N*'-[(*E*)-(3-*tert*-butyl-2-hydroxy-5-methylphenyl)methylidene]-3-pyridohydrazide (**4a**)

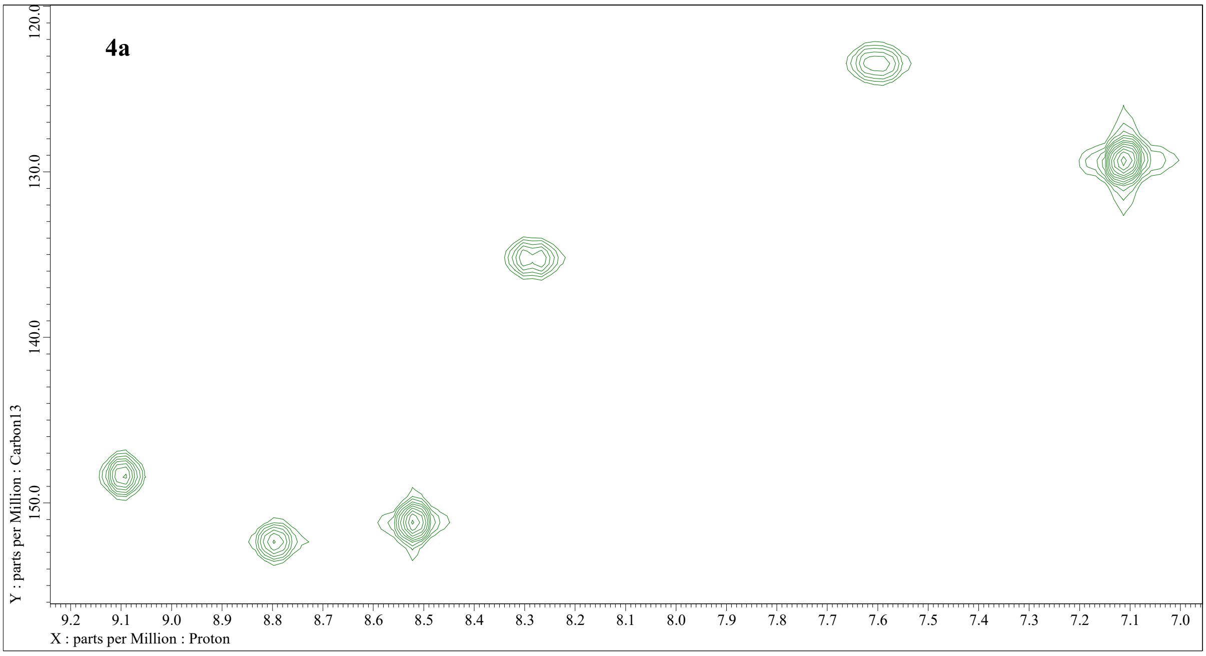

Figure S54. Expansion of 2D-NMR (400 MHz, DMSO-d_6) HMQC experiment of *N*'-[(*E*)-(3-*tert*-butyl-2-hydroxy-5-methylphenyl)methylidene]-3-pyridohydrazide (**4a**)

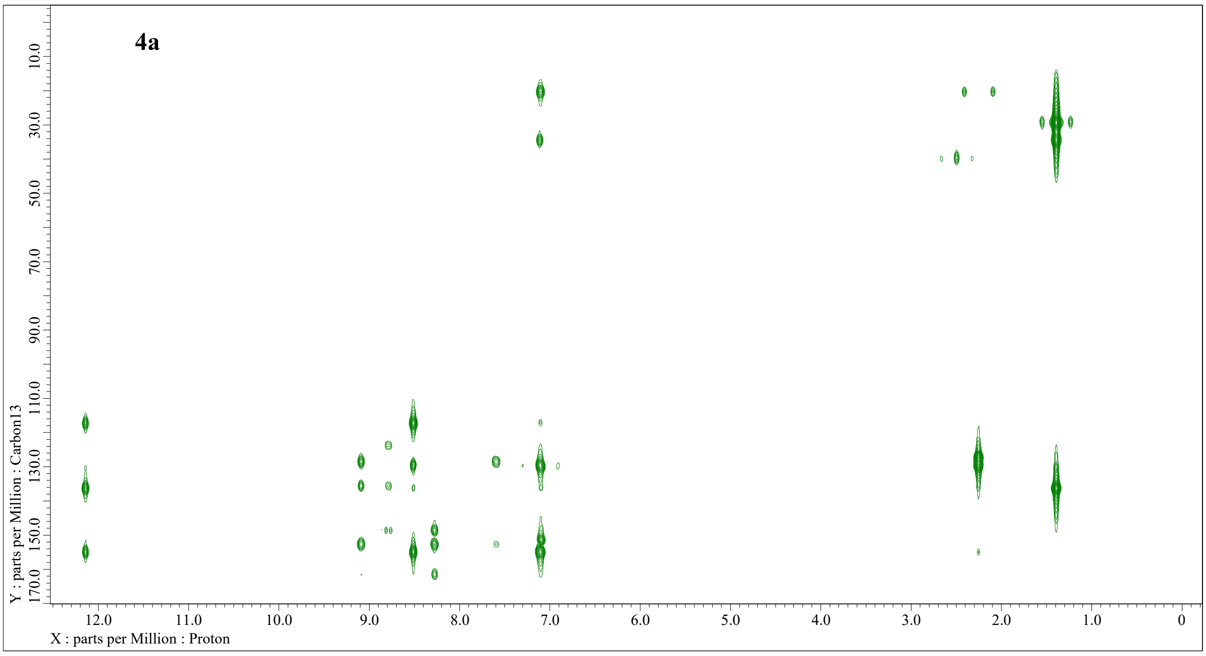

Figure S55. 2D-NMR (400 MHz, DMSO-d_6) HMBC experiment of *N*'-[(*E*)-(3-*tert*-butyl-2-hydroxy-5-methylphenyl)methylidene]-3-pyridohydrazide (**4a**)

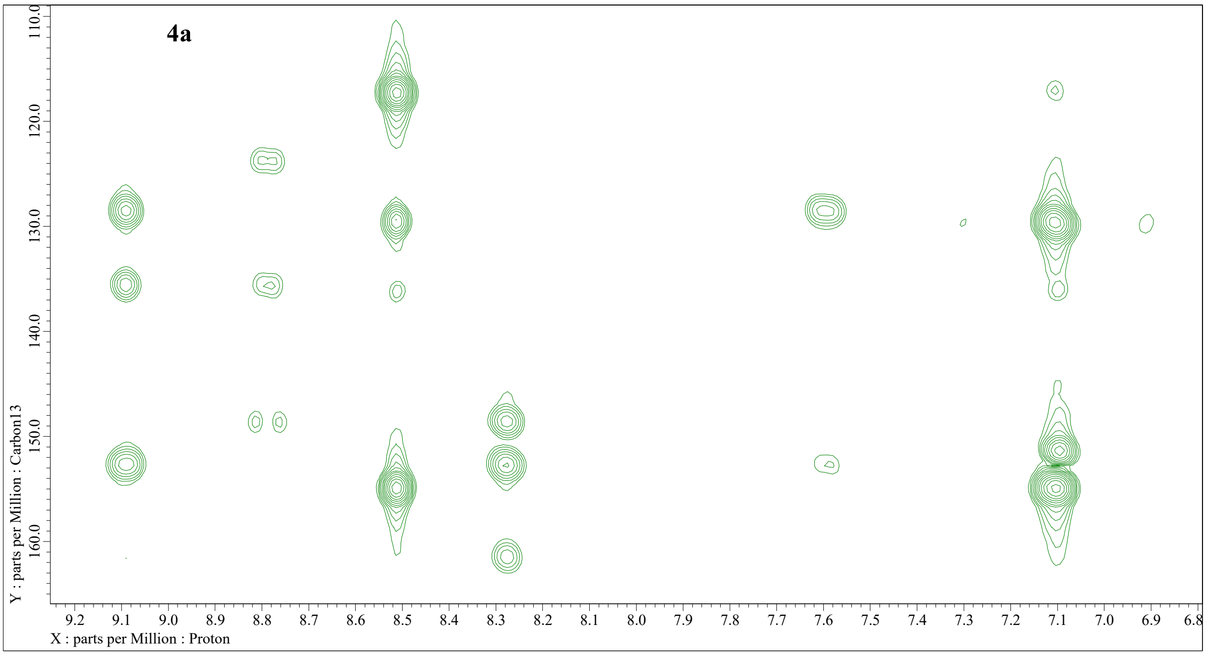

Figure S56. Expansion of 2D-NMR (400 MHz, DMSO-d_6) HMBC experiment of *N*'-[(*E*)-(3-*tert*-butyl-2-hydroxy-5-methylphenyl)methylidene]-3-pyridohydrazide (**4a**)

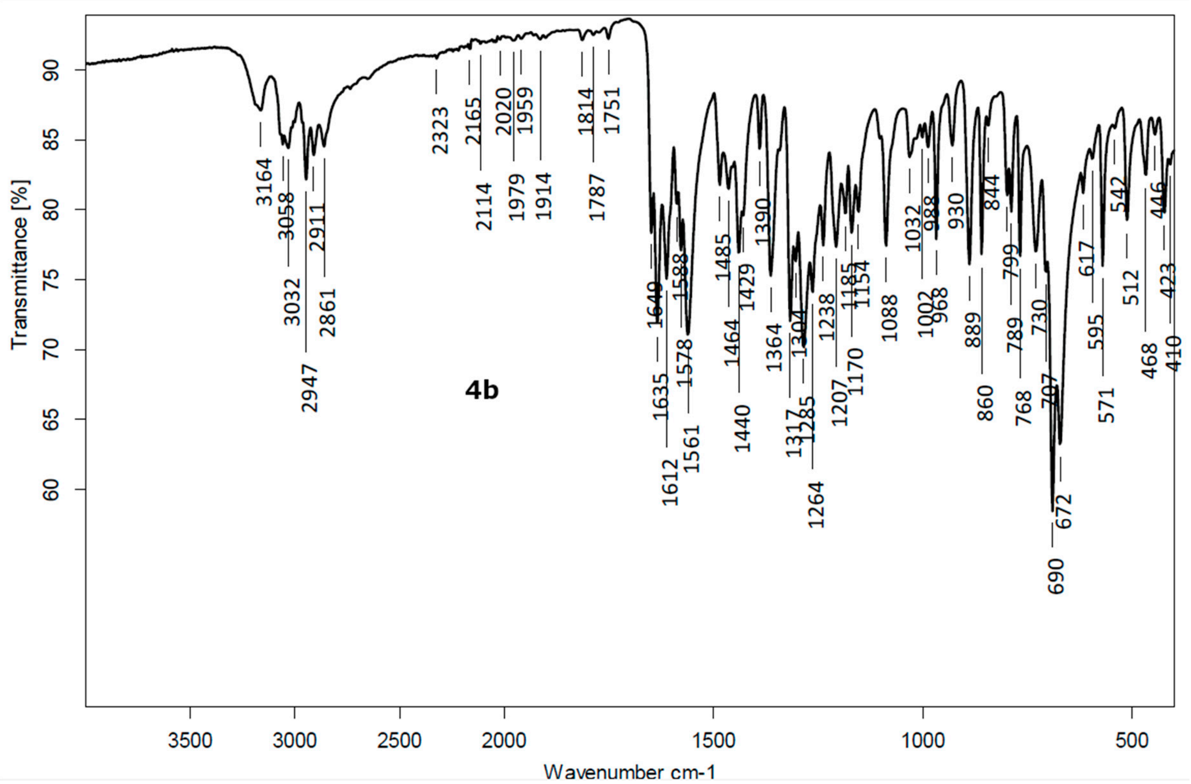

Figure S57. FT-IR (ATR) spectrum of *N*'-[(*E*)-(3-*tert*-butyl-2-hydroxy-5-methylphenyl)methylidene]benzohydrazide (**4b**)

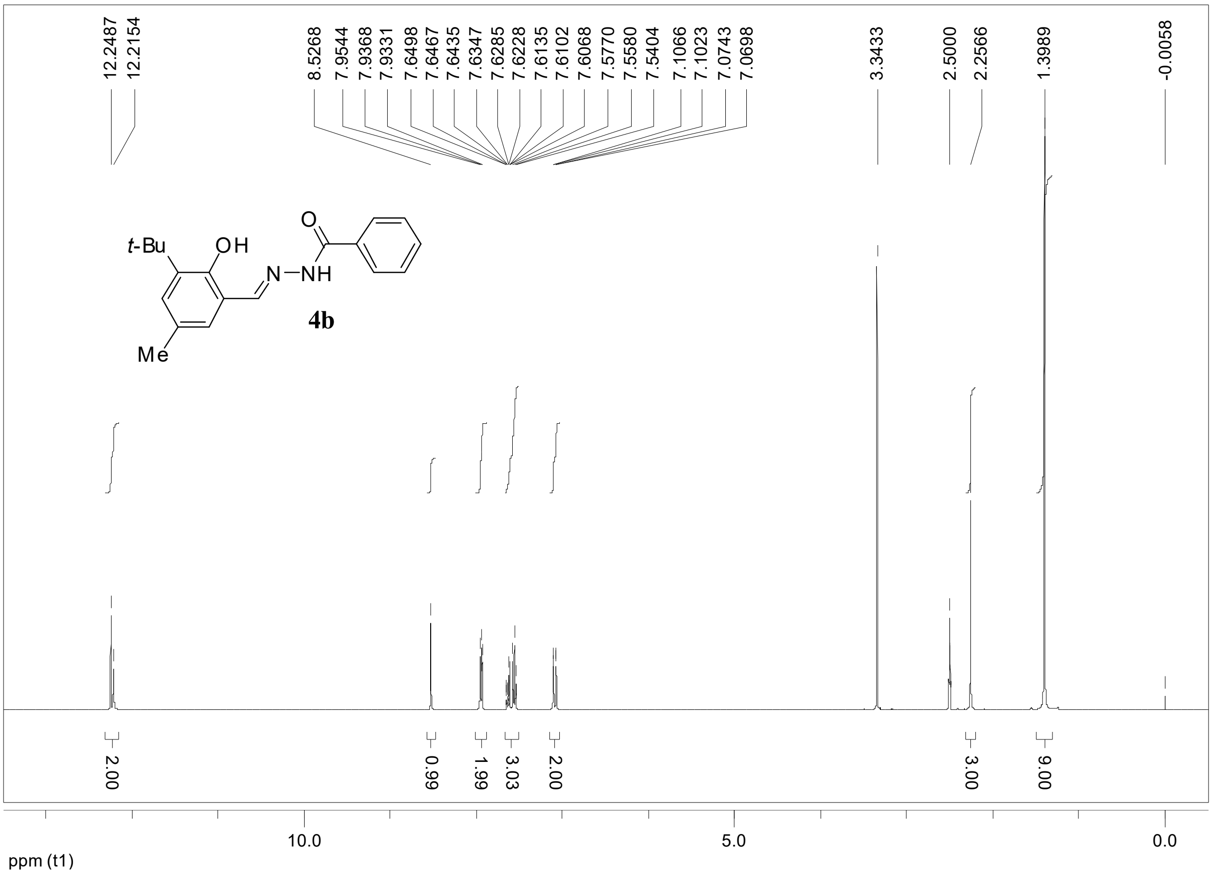

Figure S58. ^{1}H-NMR (400 MHz, DMSO-d_6) spectrum of compound **4b**

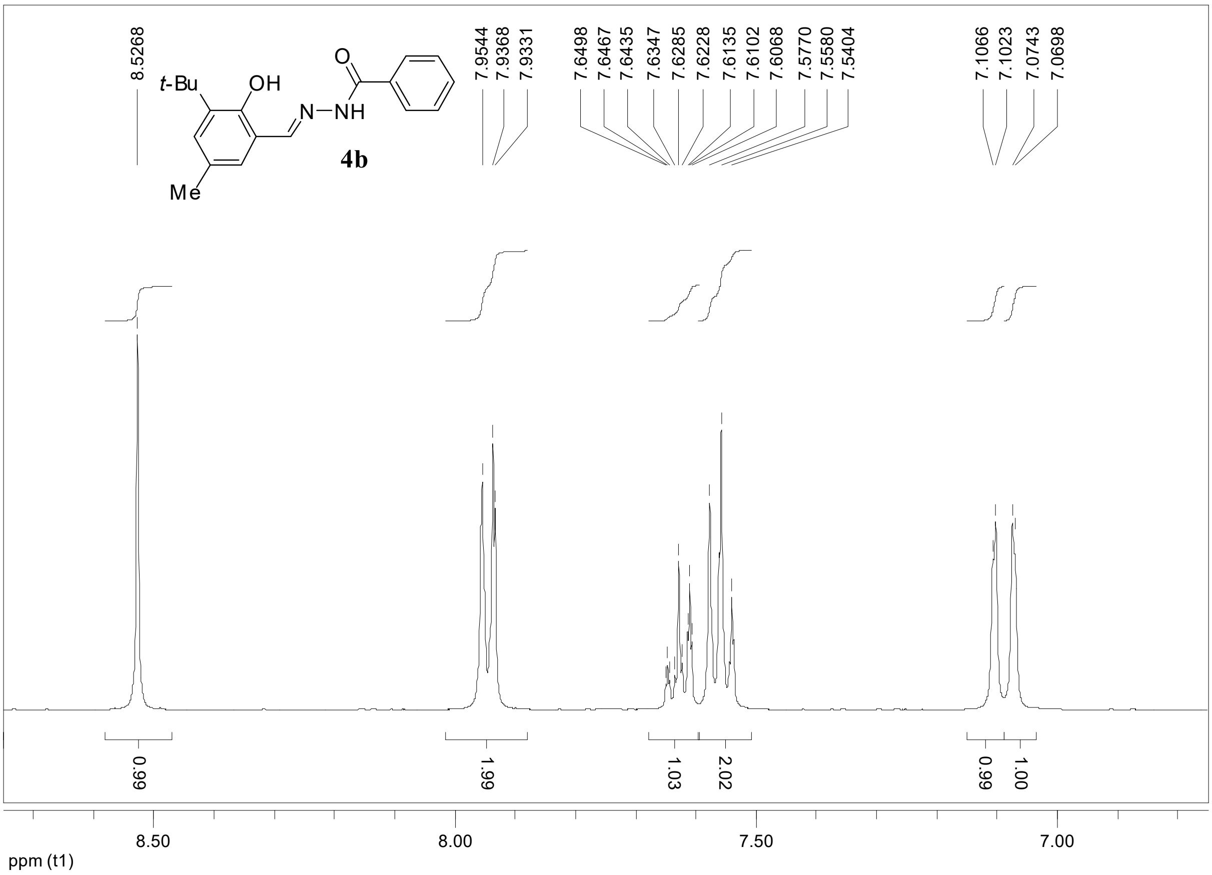

Figure S59. Expansion of ^{1}H-NMR (400 MHz, DMSO-d_6) spectrum of compound **4b**

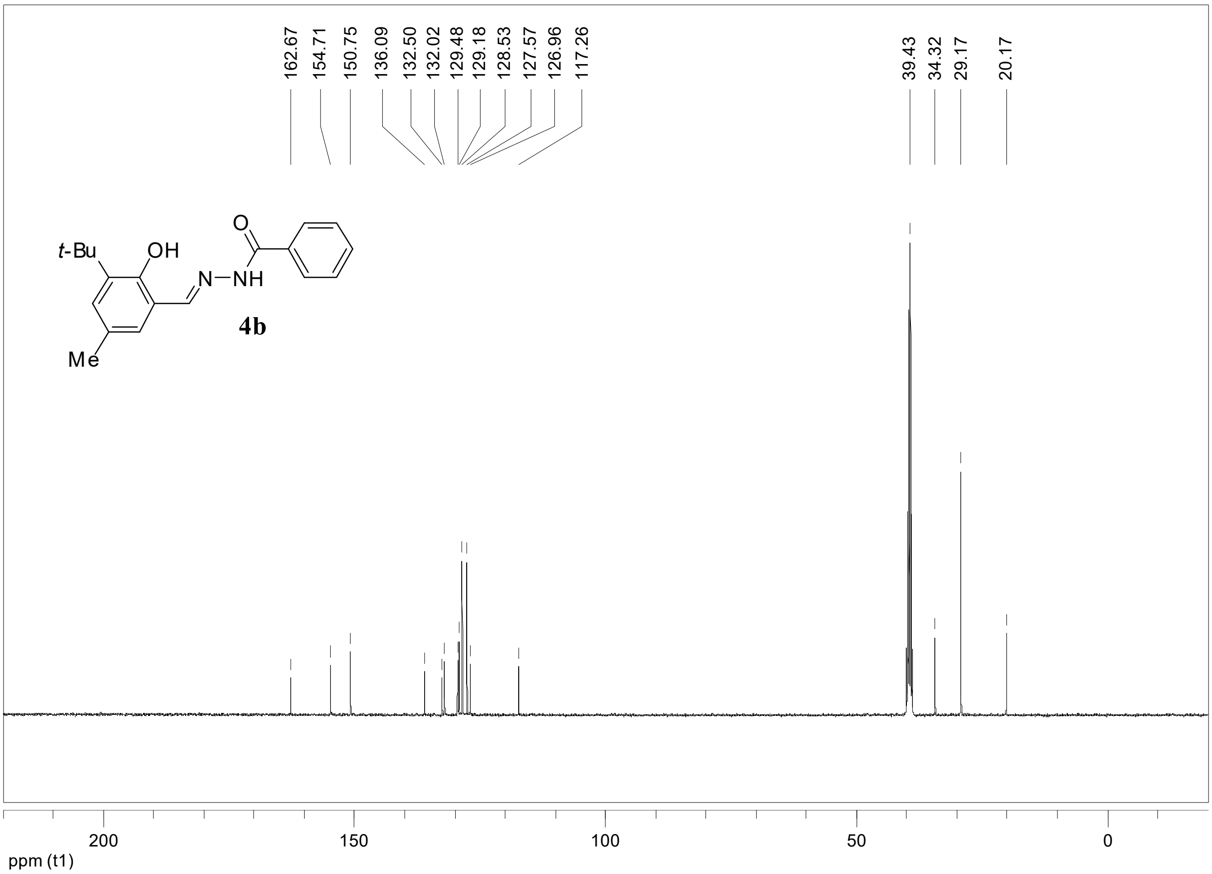

Figure S60. ^{13}C-NMR (100 MHz, DMSO-d_6) spectrum of compound **4b**

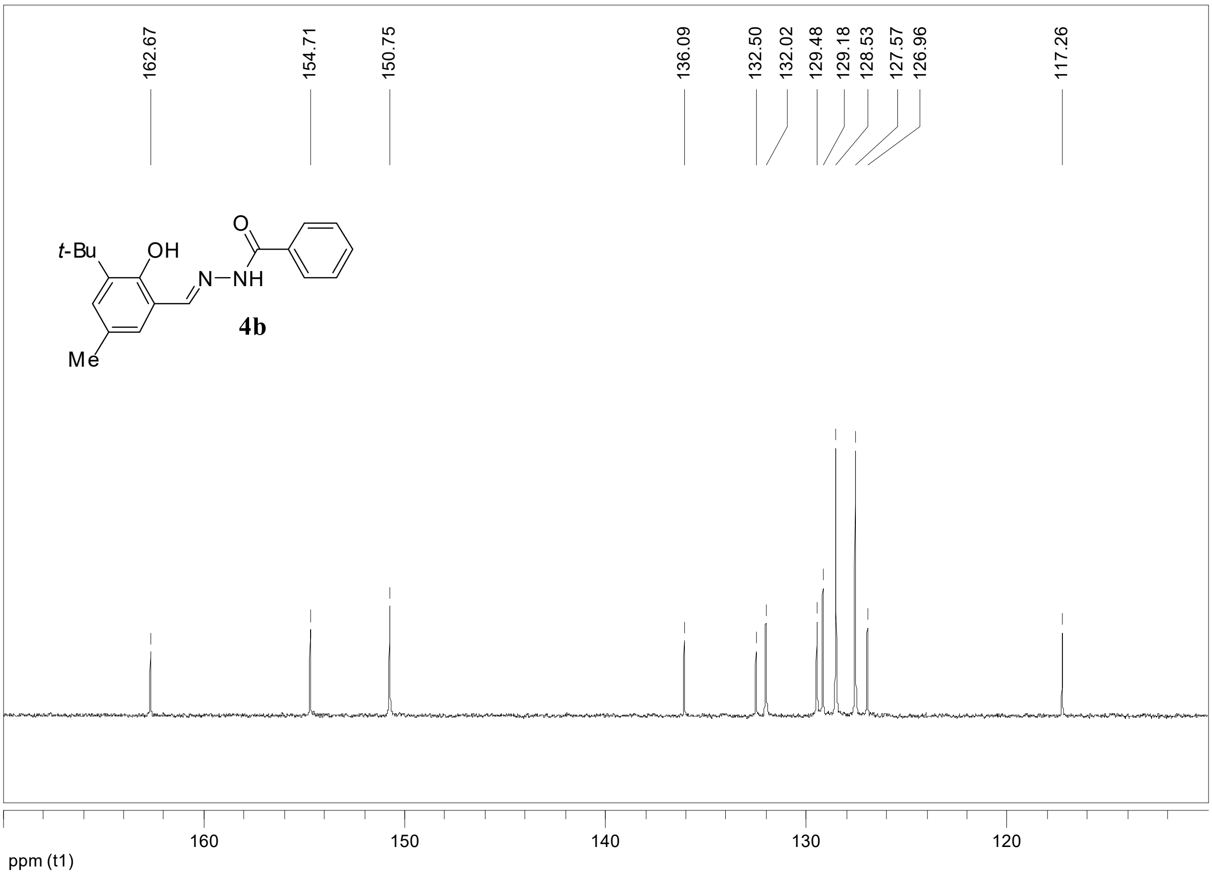

Figure S61. Expansion of ^{13}C-NMR (100 MHz, DMSO-d_6) spectrum of compound **4b**

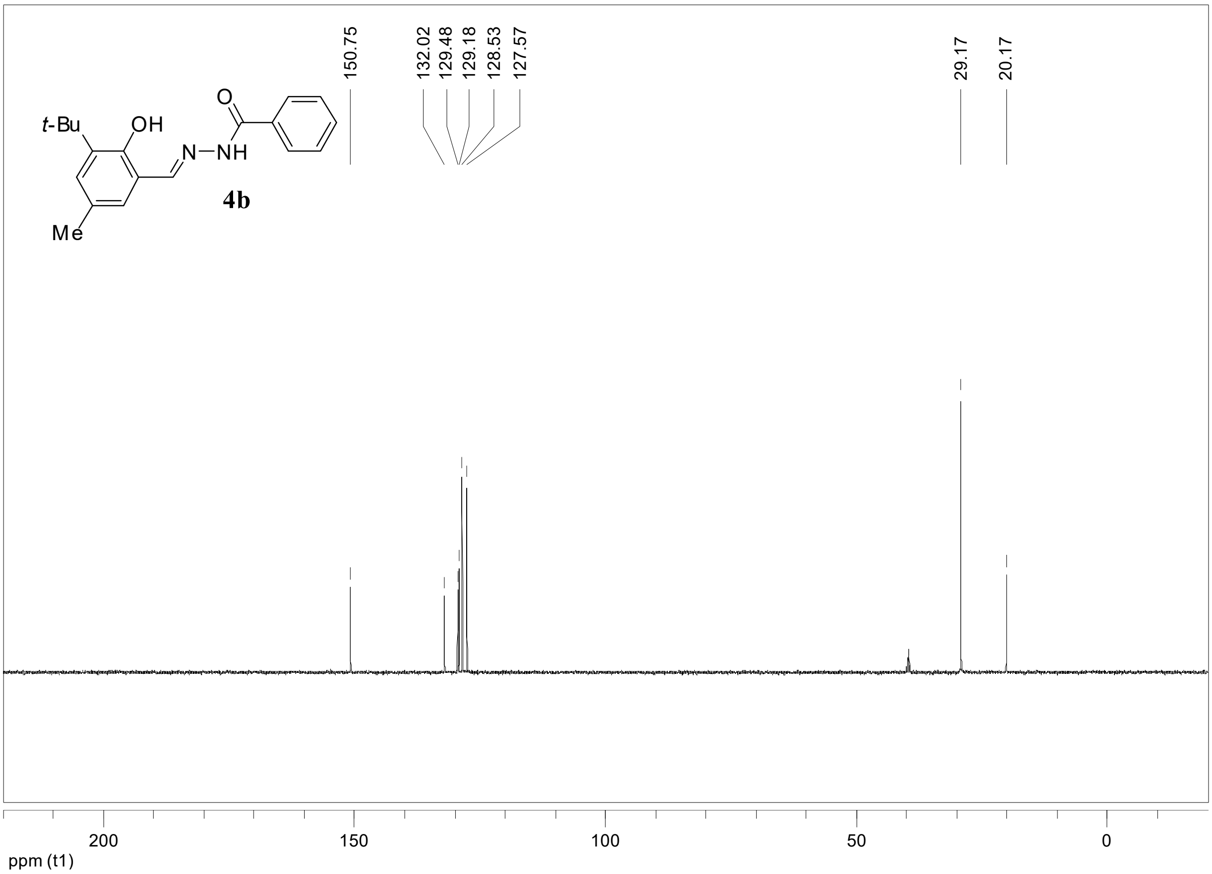

Figure S62. ^{13}C-NMR (100 MHz, DMSO-d_6) dept-135 experiment of compound **4b**

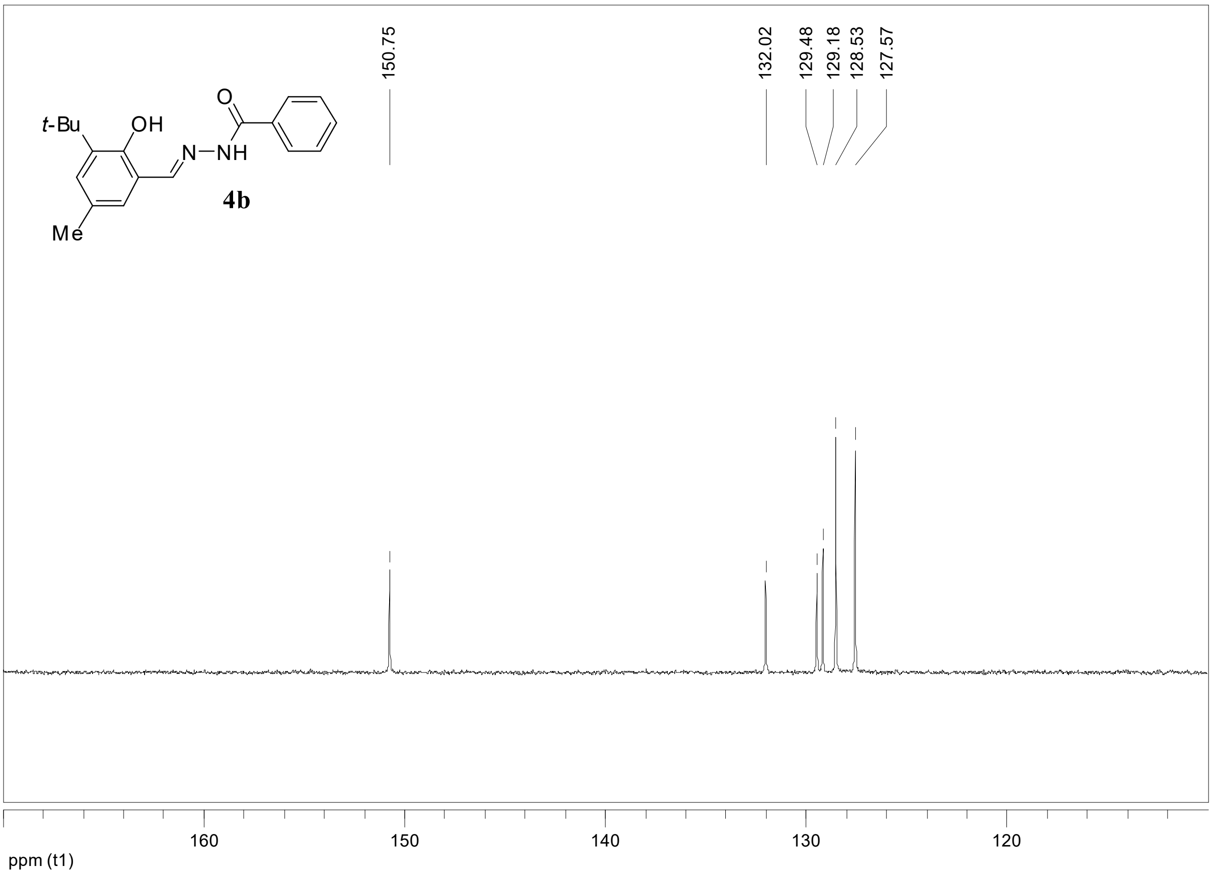

Figure S63. Expansion of ^{13}C-NMR (100 MHz, DMSO-d_6) dept-135 experiment of compound **4b**

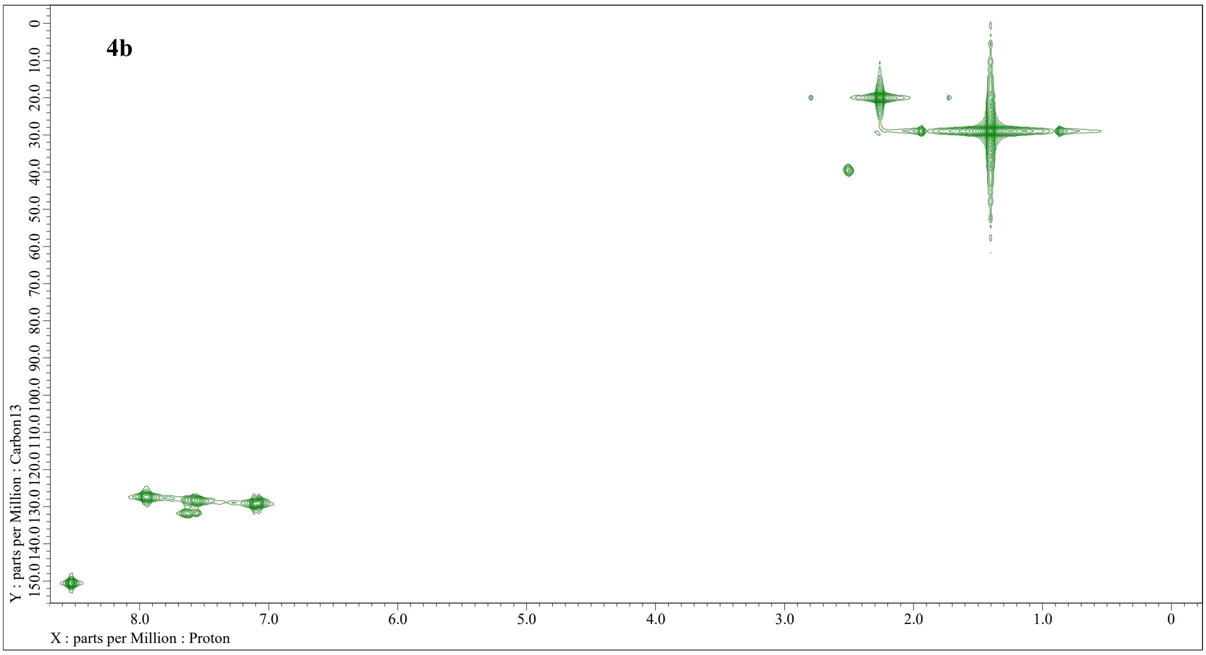

Figure S64. 2D-NMR (400 MHz, DMSO-d_6) HMQC experiment of *N*'-[(*E*)-(3-*tert*-butyl-2-hydroxy-5-methylphenyl)methylidene]benzohydrazide (**4b**)

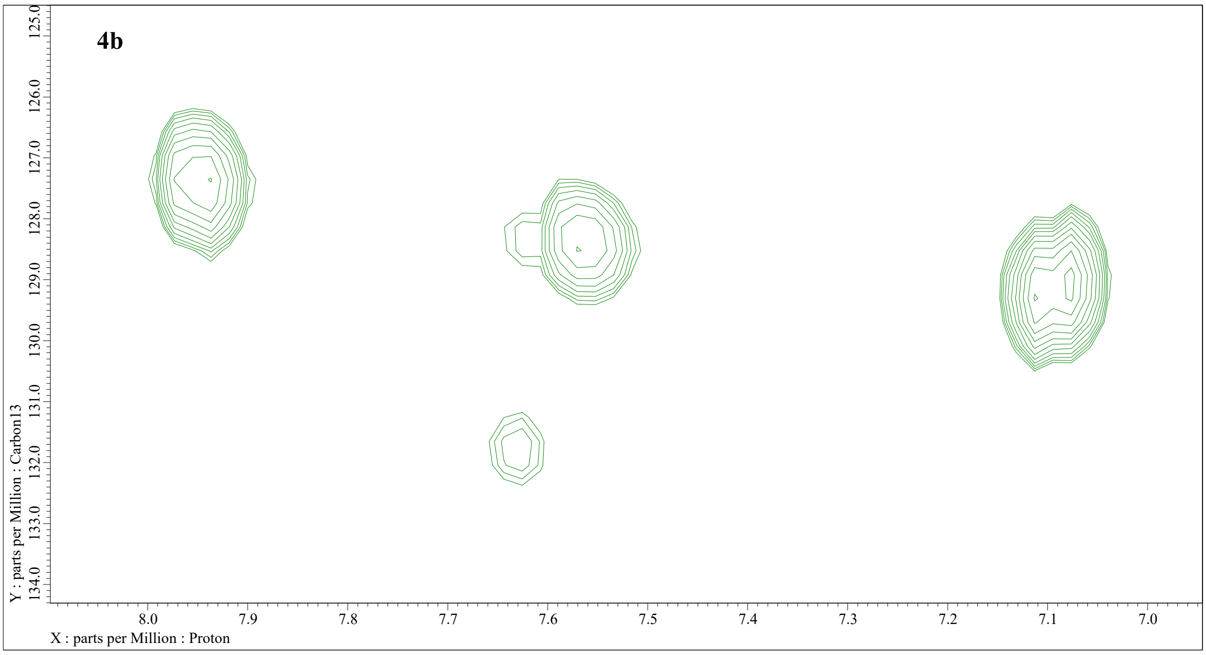

Figure S65. Expansion of 2D-NMR (400 MHz, DMSO-d_6) HMQC experiment of *N*'-[(*E*)-(3-*tert*-butyl-2-hydroxy-5-methylphenyl)methylidene]benzohydrazide (**4b**)

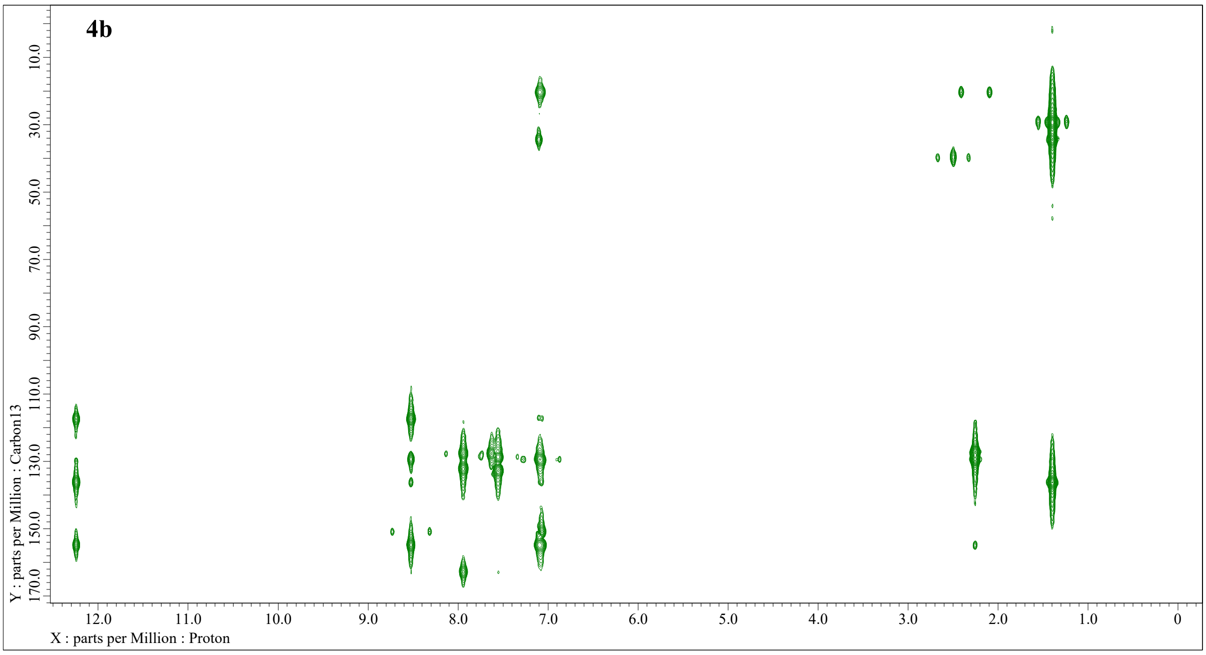

Figure S66. 2D-NMR (400 MHz, DMSO-d_6) HMBC experiment of *N*'-[(*E*)-(3-*tert*-butyl-2-hydroxy-5-methylphenyl)methylidene]benzohydrazide (**4b**)

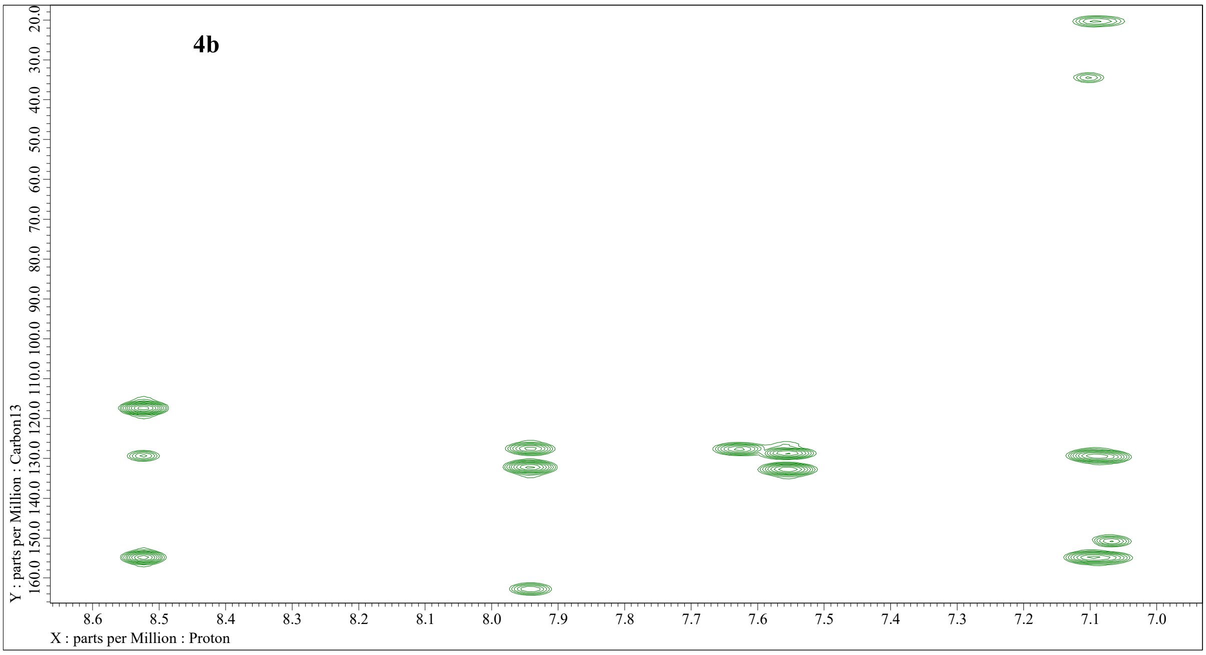

Figure S67. Expansion 2D-NMR (400 MHz, DMSO-d_6) HMBC experiment of *N*'-[(*E*)-(3-*tert*-butyl-2-hydroxy-5-methylphenyl)methylidene]benzohydrazide (**4b**)

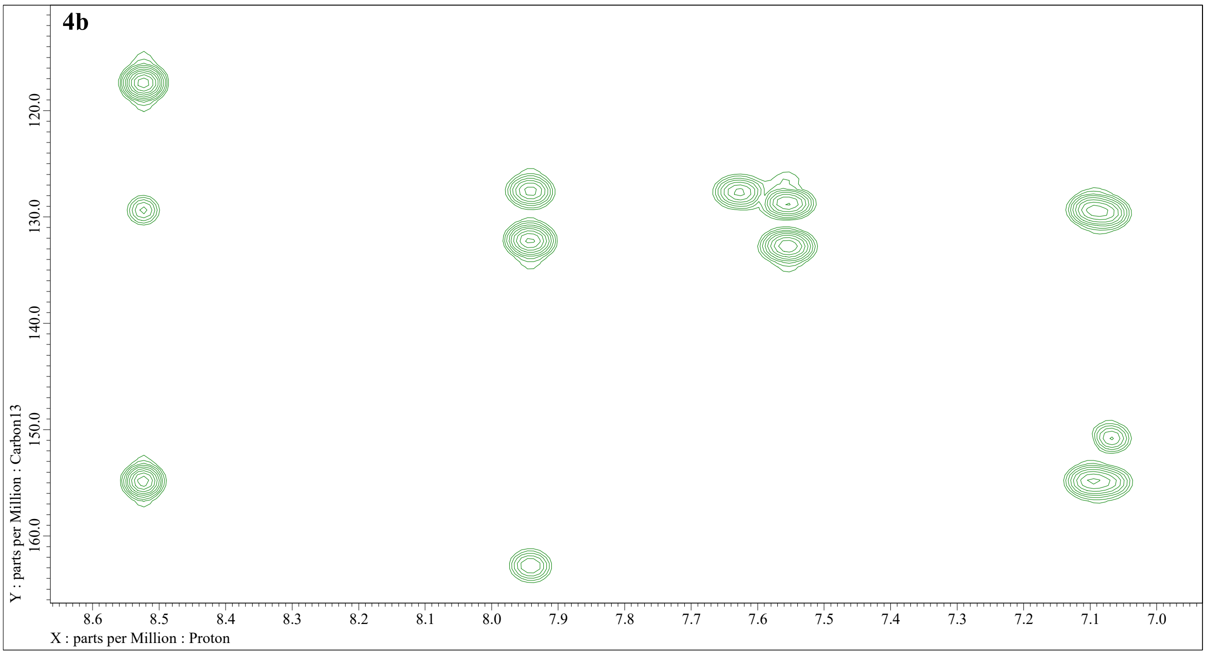

Figure S68. Expansion 2D-NMR (400 MHz, DMSO-d_6) HMBC experiment of *N'*-[(*E*)-(3-*tert*-butyl-2-hydroxy-5-methylphenyl)methylidene]benzohydrazide (**4b**)

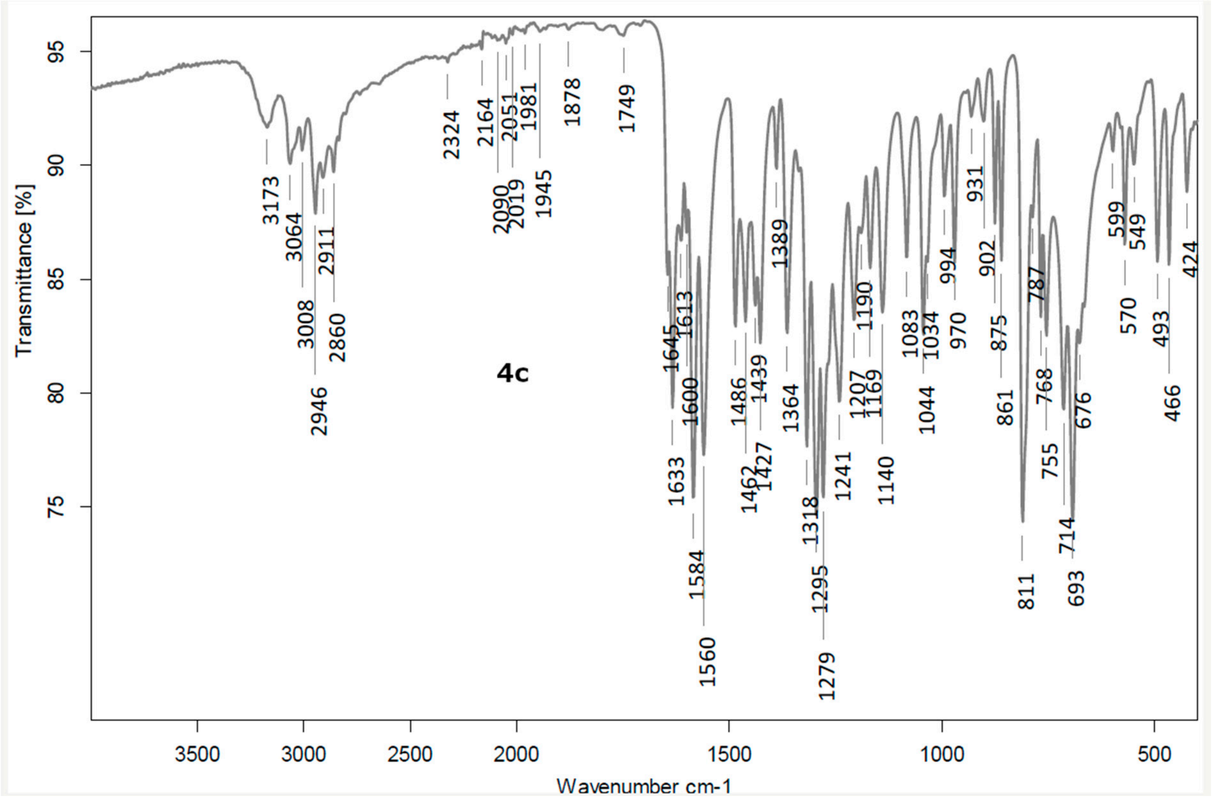

Figure S69. FT-IR (ATR) spectrum of 3-methoxy-*N'*-[(*E*)-(3-*tert*-butyl-2-hydroxy-5-methylphenyl)methylidene]benzohydrazide (**4c**)

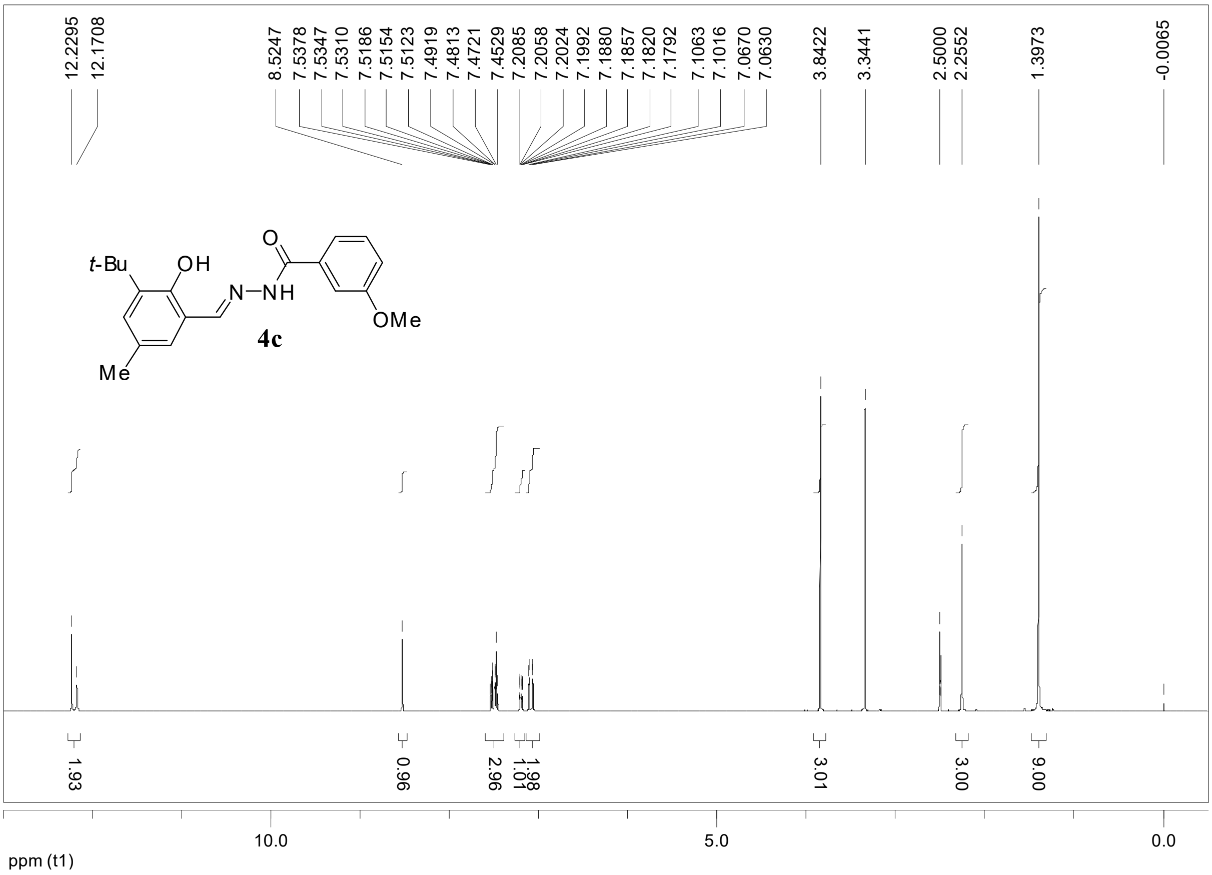

Figure S70. ^{1}H-NMR (400 MHz, DMSO-d_6) spectrum of compound **4c**

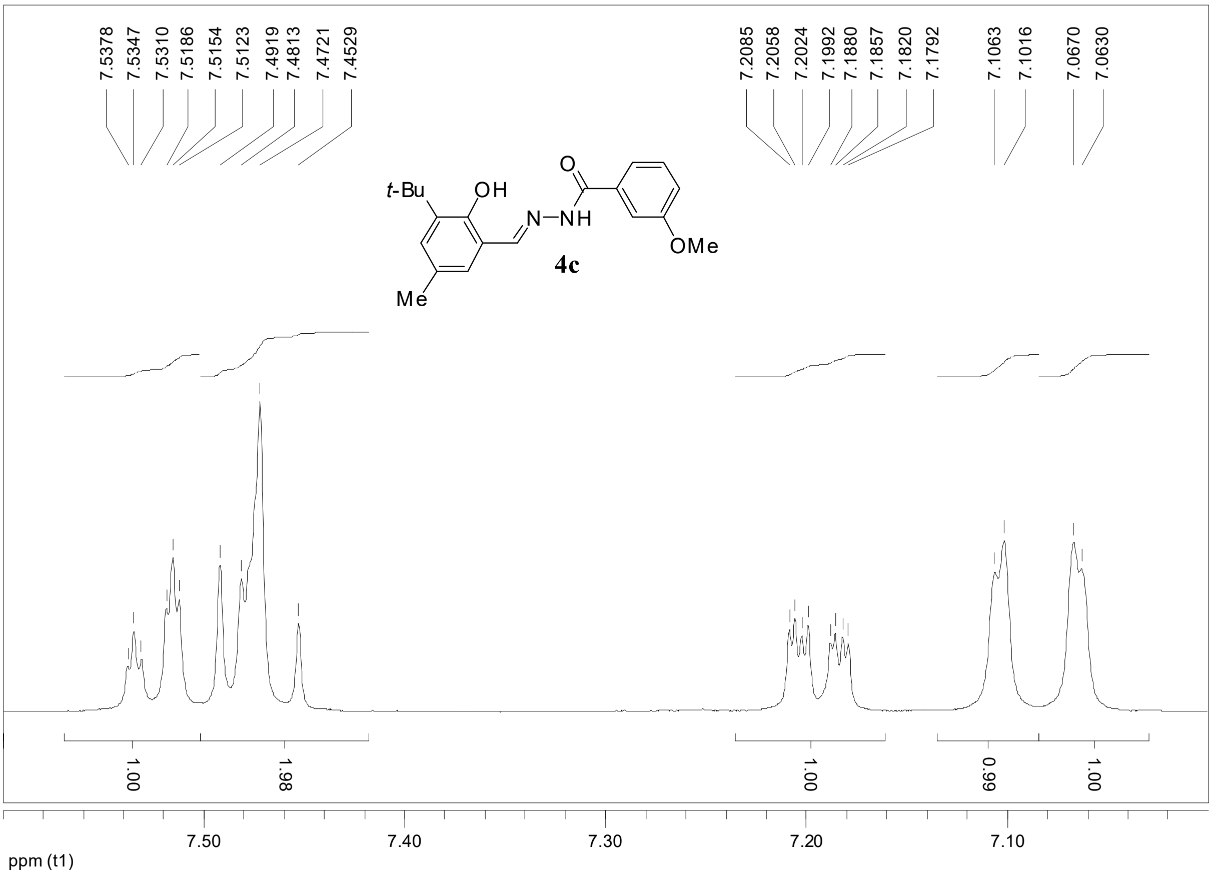

Figure S71. Expansion of ^{1}H-NMR (400 MHz, DMSO-d_6) spectrum of compound **4c**

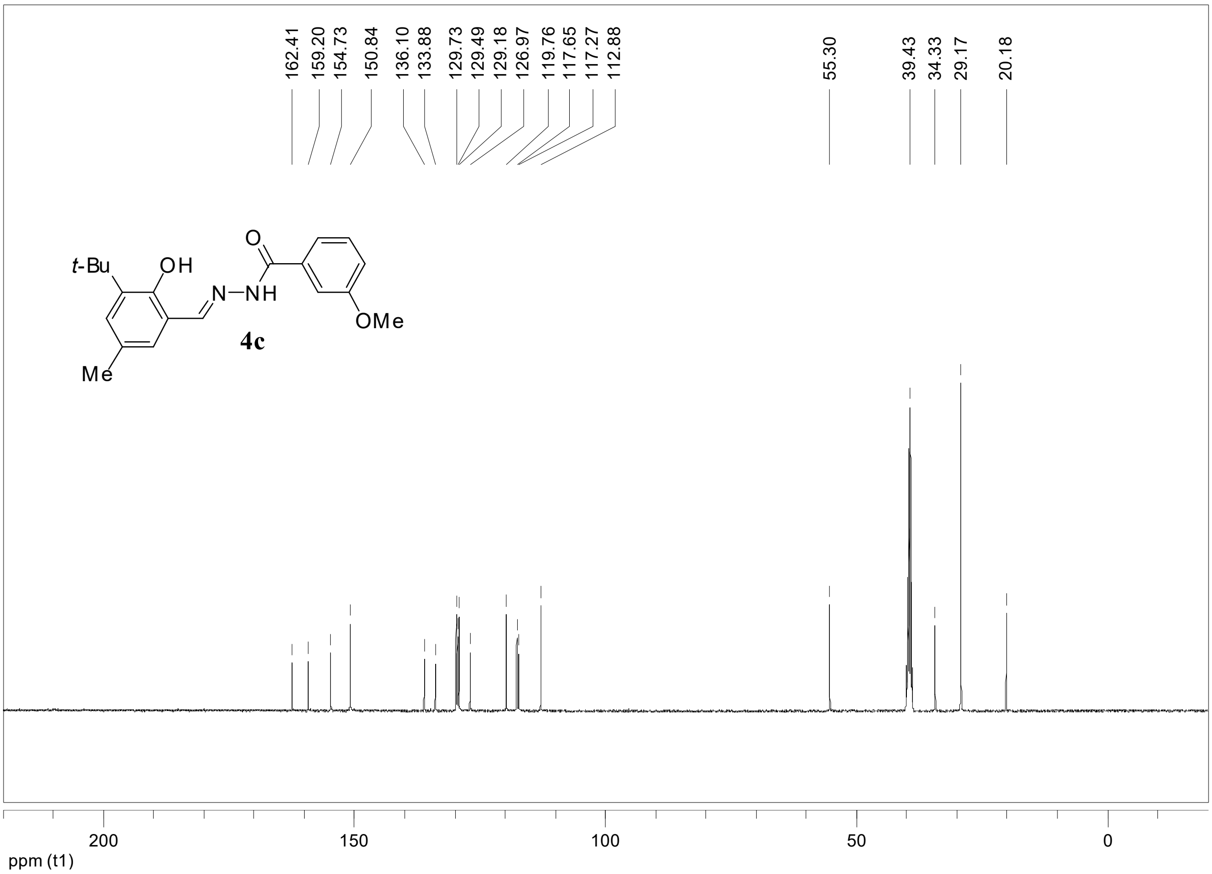

Figure S72. ^{13}C-NMR (100 MHz, DMSO-d_6) spectrum of compound **4c**

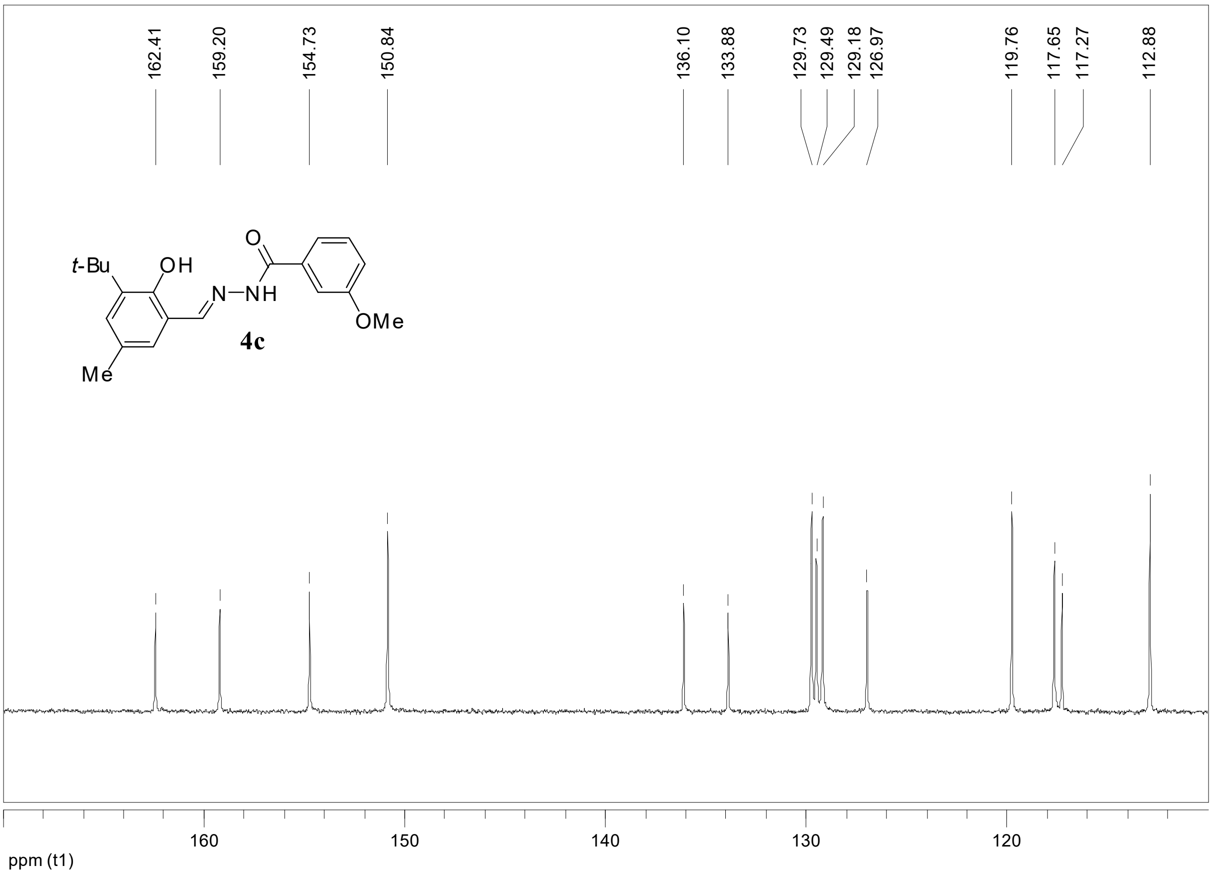

Figure S73. Expansion of ^{13}C-NMR (100 MHz, DMSO-d_6) spectrum of compound **4c**

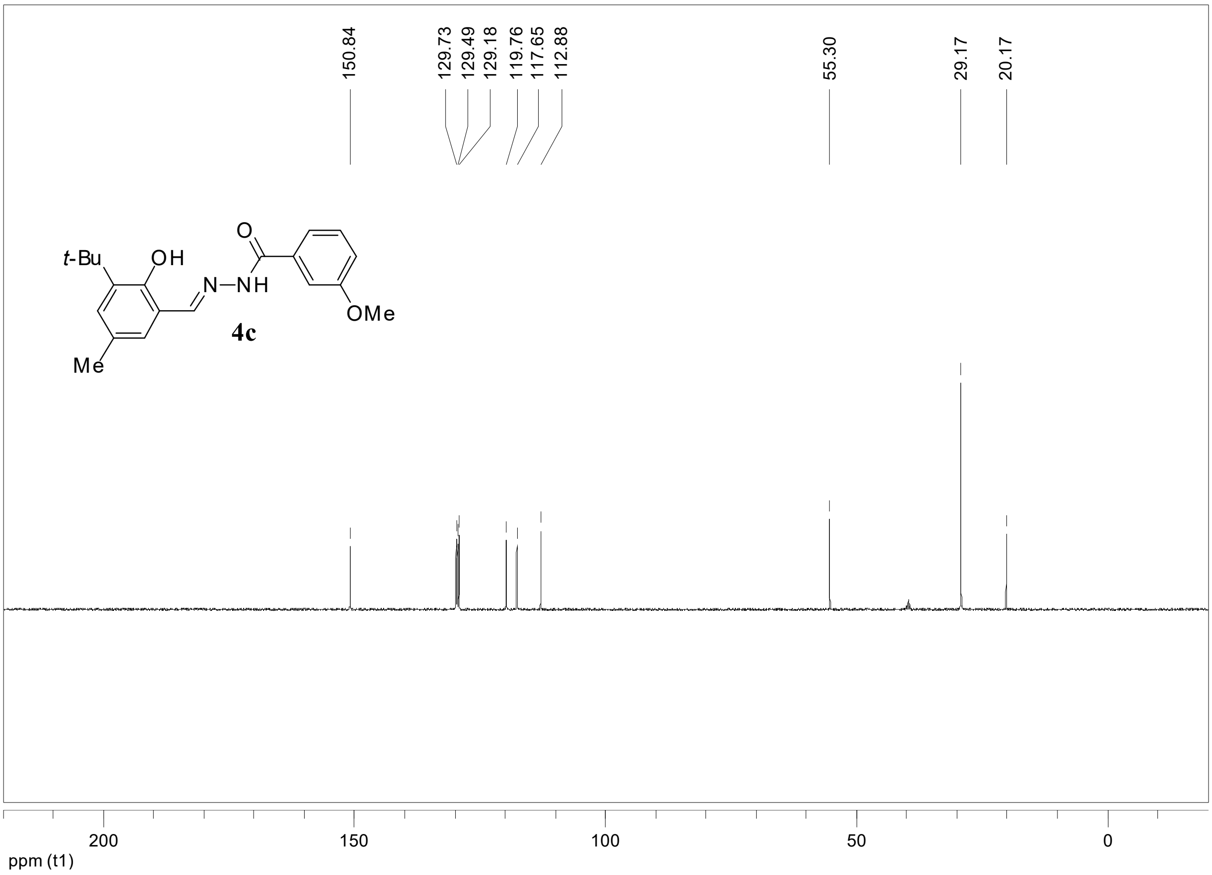

Figure S74. ^{13}C-NMR (100 MHz, DMSO-d_6) dept-135 experiment of compound **4c**

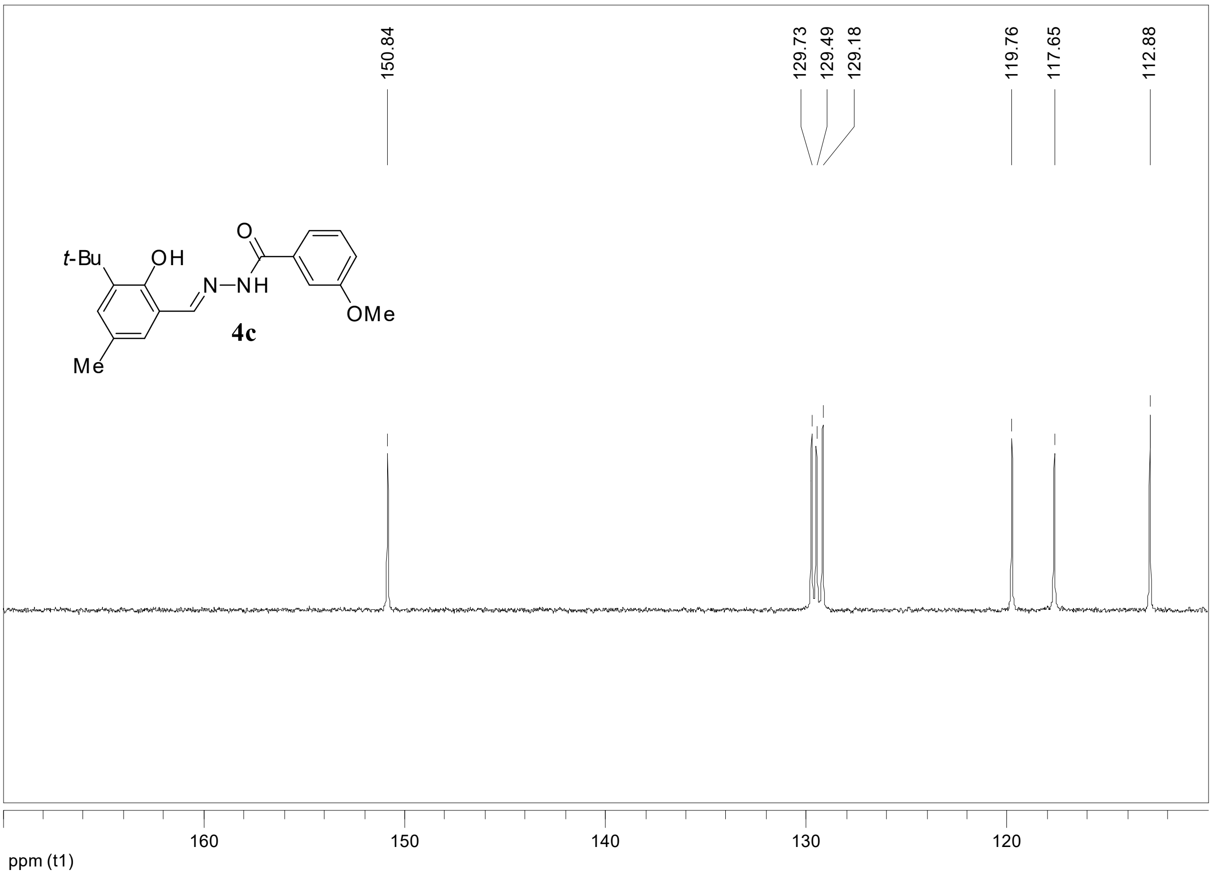

Figure S75. Expansion of ^{13}C-NMR (100 MHz, DMSO-d_6) dept-135 experiment of compound **4c**

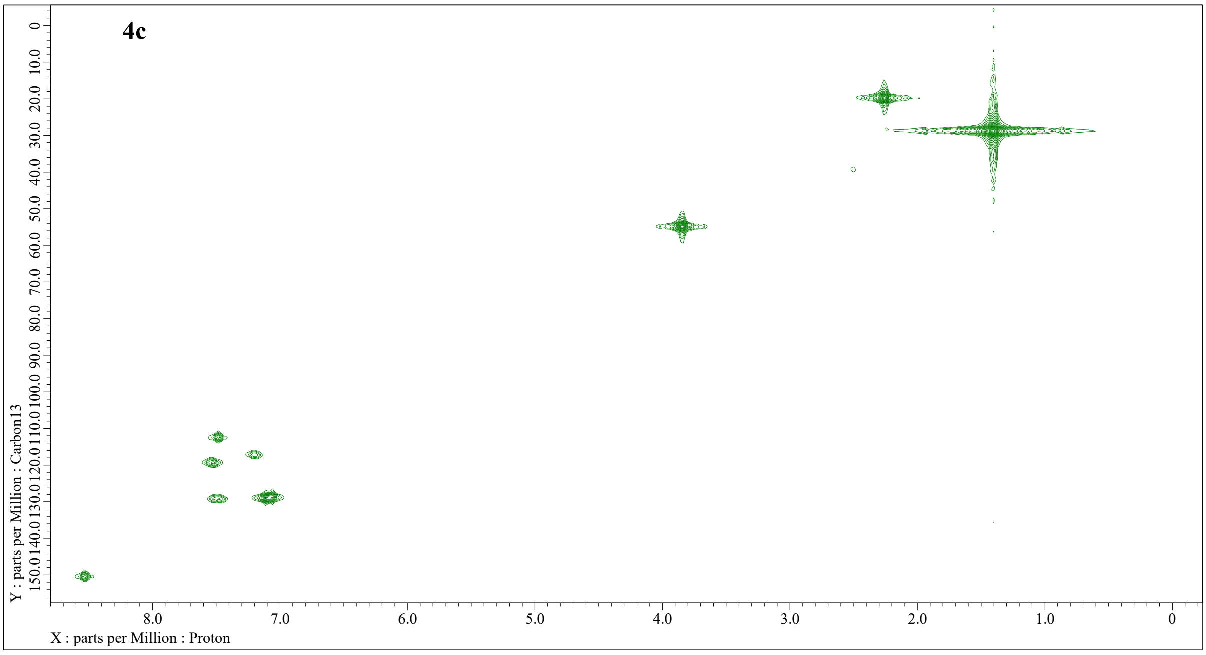

Figure S76. 2D-NMR (400 MHz, DMSO-d_6) HMQC experiment of 3-methoxy-*N*'-[(*E*)-(3-*tert*-butyl-2-hydroxy-5-methylphenyl)methylidene]benzohydrazide (**4c**)

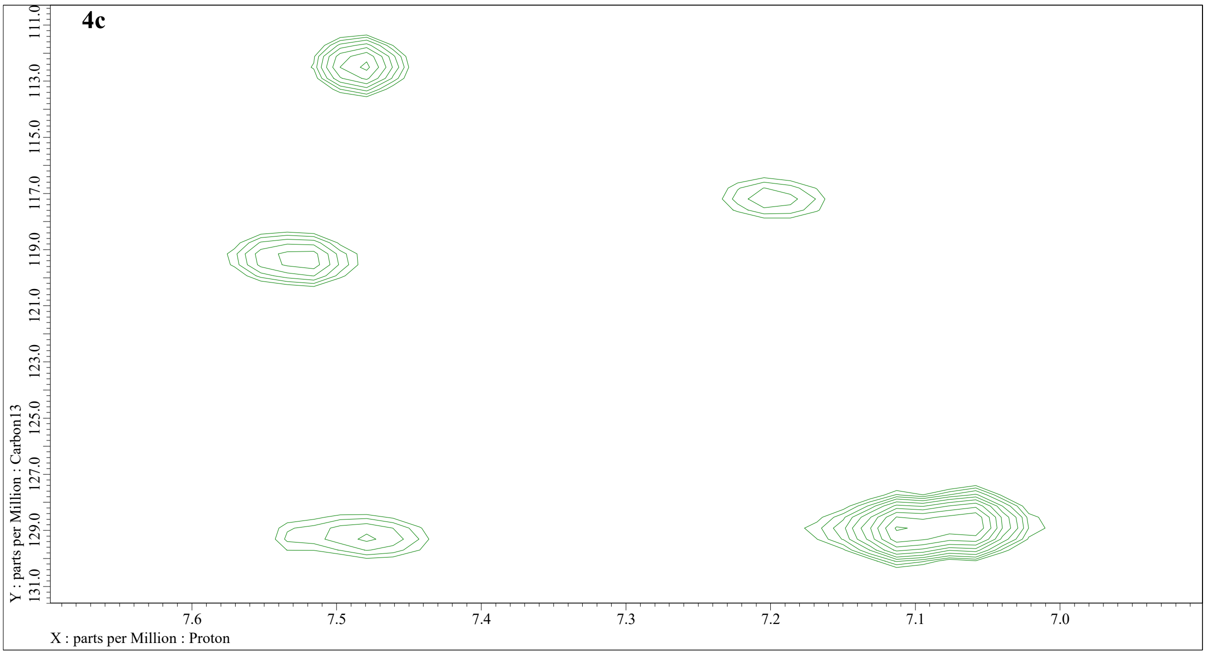

Figure S77. Expansion of 2D-NMR (400 MHz, DMSO-d_6) HMQC experiment of 3-methoxy-*N*'-[(*E*)-(3-*tert*-butyl-2-hydroxy-5-methylphenyl)methylidene]benzohydrazide (**4c**)

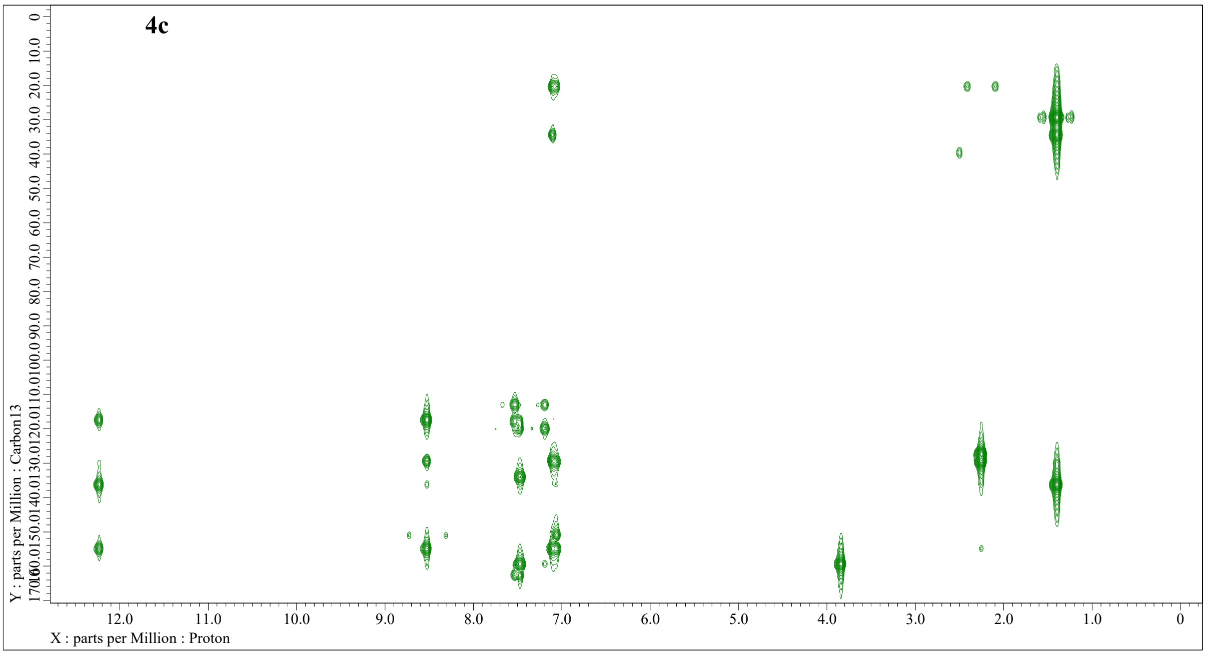

Figure S78. 2D-NMR (400 MHz, DMSO-d_6) HMBC experiment of 3-methoxy-*N*'-[(*E*)-(3-*tert*-butyl-2-hydroxy-5-methylphenyl)methylidene]benzohydrazide (**4c**)

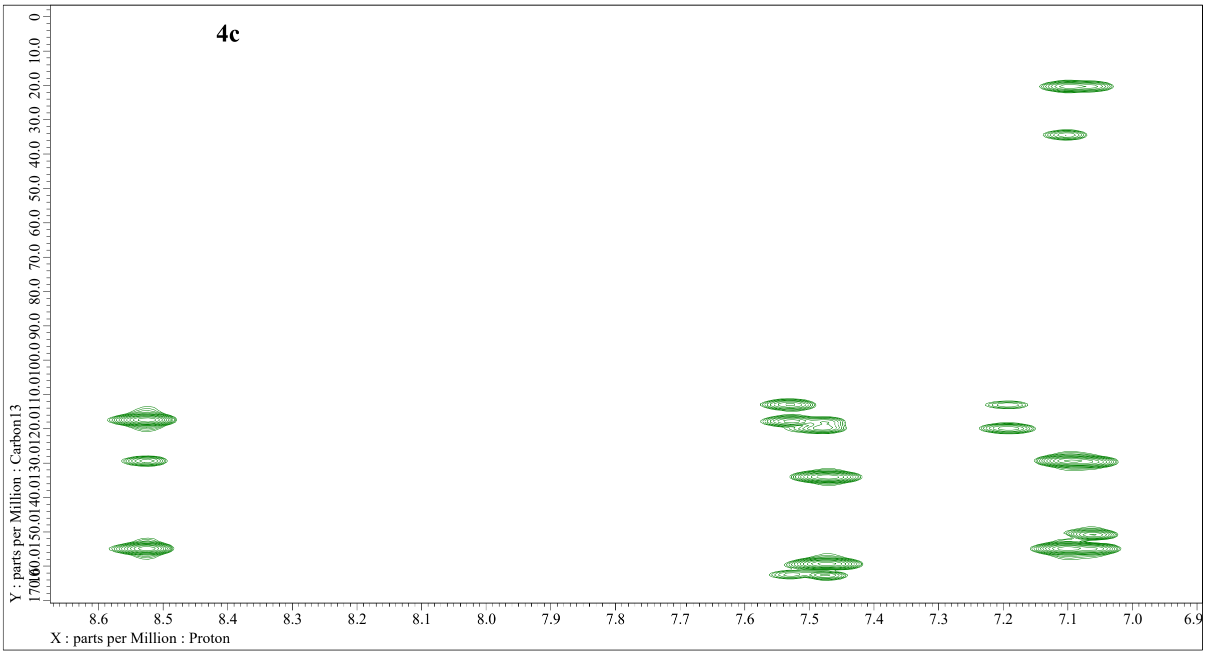

Figure S79. Expansion of 2D-NMR (400 MHz, DMSO-d_6) HMBC experiment of 3-methoxy-*N*'-[(*E*)-(3-*tert*-butyl-2-hydroxy-5-methylphenyl)methylidene]benzohydrazide (**4c**)

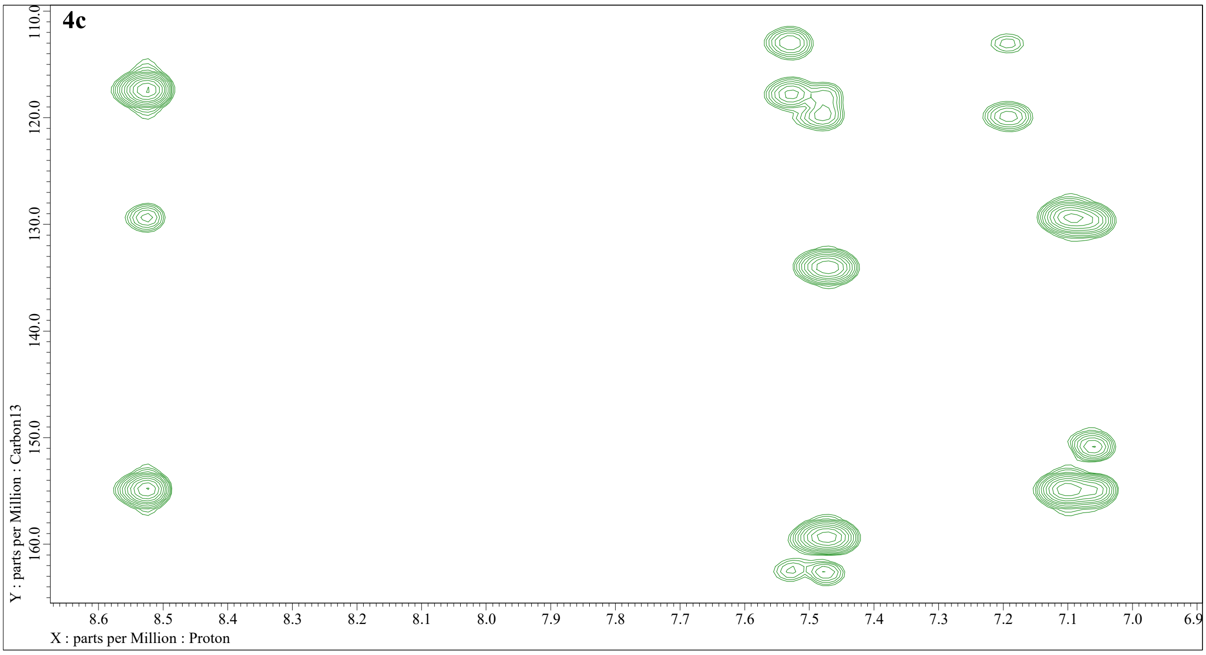

Figure S80. Expansion of 2D-NMR (400 MHz, DMSO-d_6) HMBC experiment of 3-methoxy-*N'*-[(*E*)-(3-*tert*-butyl-2-hydroxy-5-methylphenyl)methylidene]benzohydrazide (**4c**)

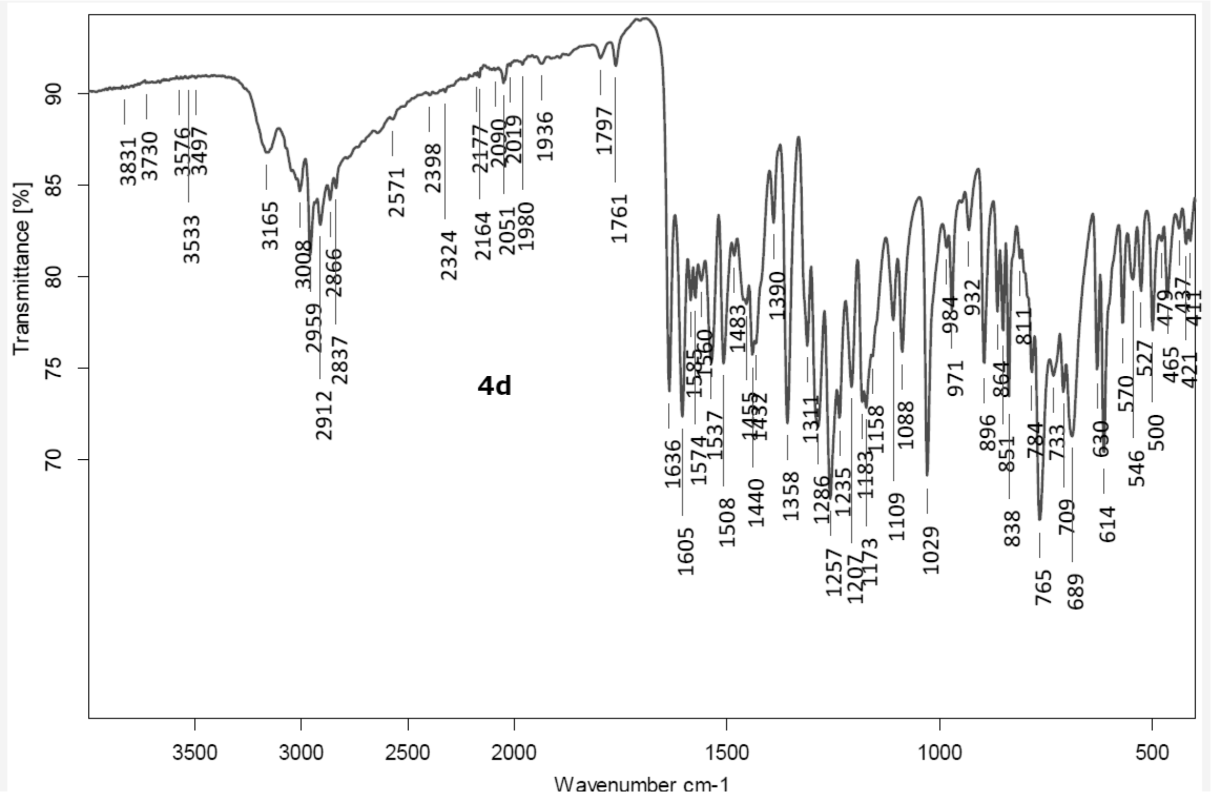

Figure S81. FT-IR (ATR) spectrum of 4-methoxy-*N'*-[(*E*)-(3-*tert*-butyl-2-hydroxy-5-methylphenyl)methylidene]benzohydrazide (**4d**)

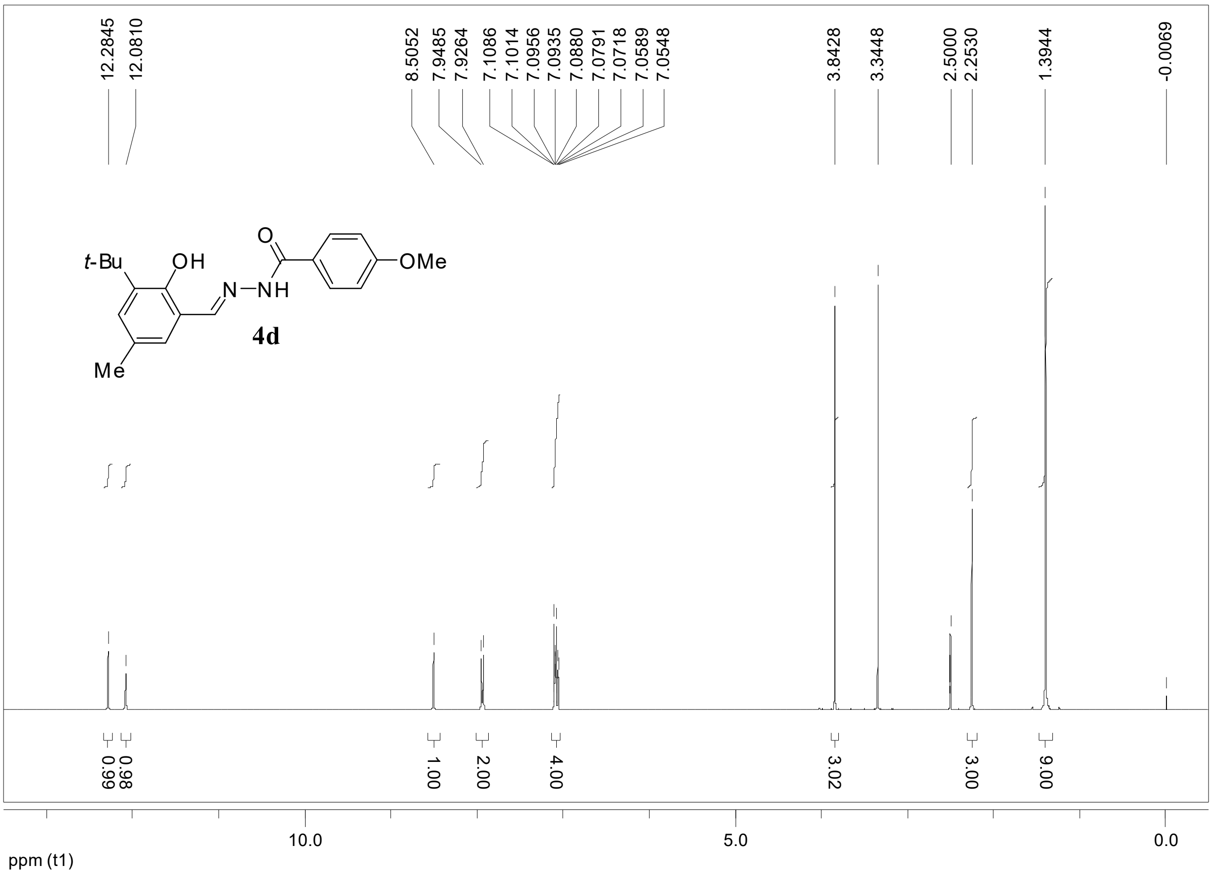

Figure S82. ^{1}H-NMR (400 MHz, DMSO-d_6) spectrum of compound **4d**

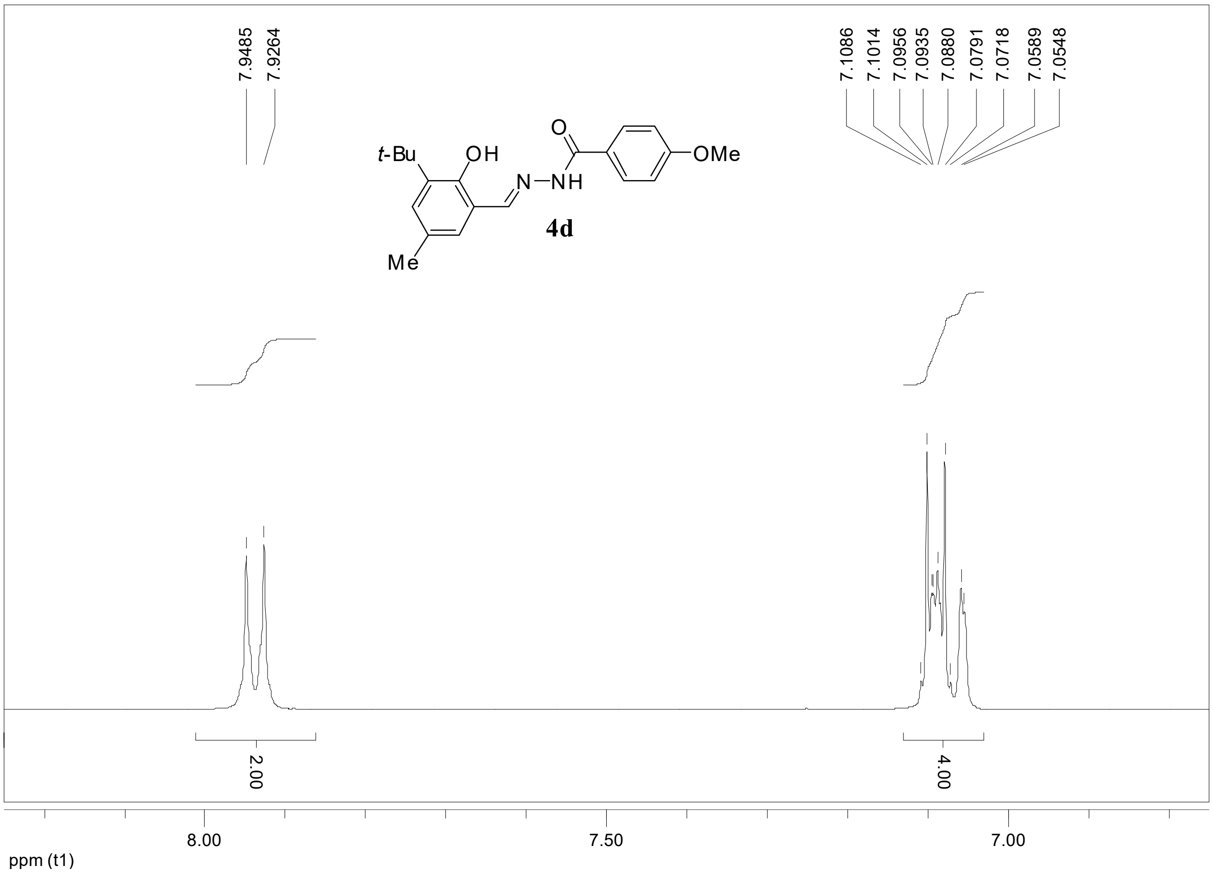

Figure S83. Expansion of ^{1}H-NMR (400 MHz, DMSO-d_6) spectrum of compound **4d**

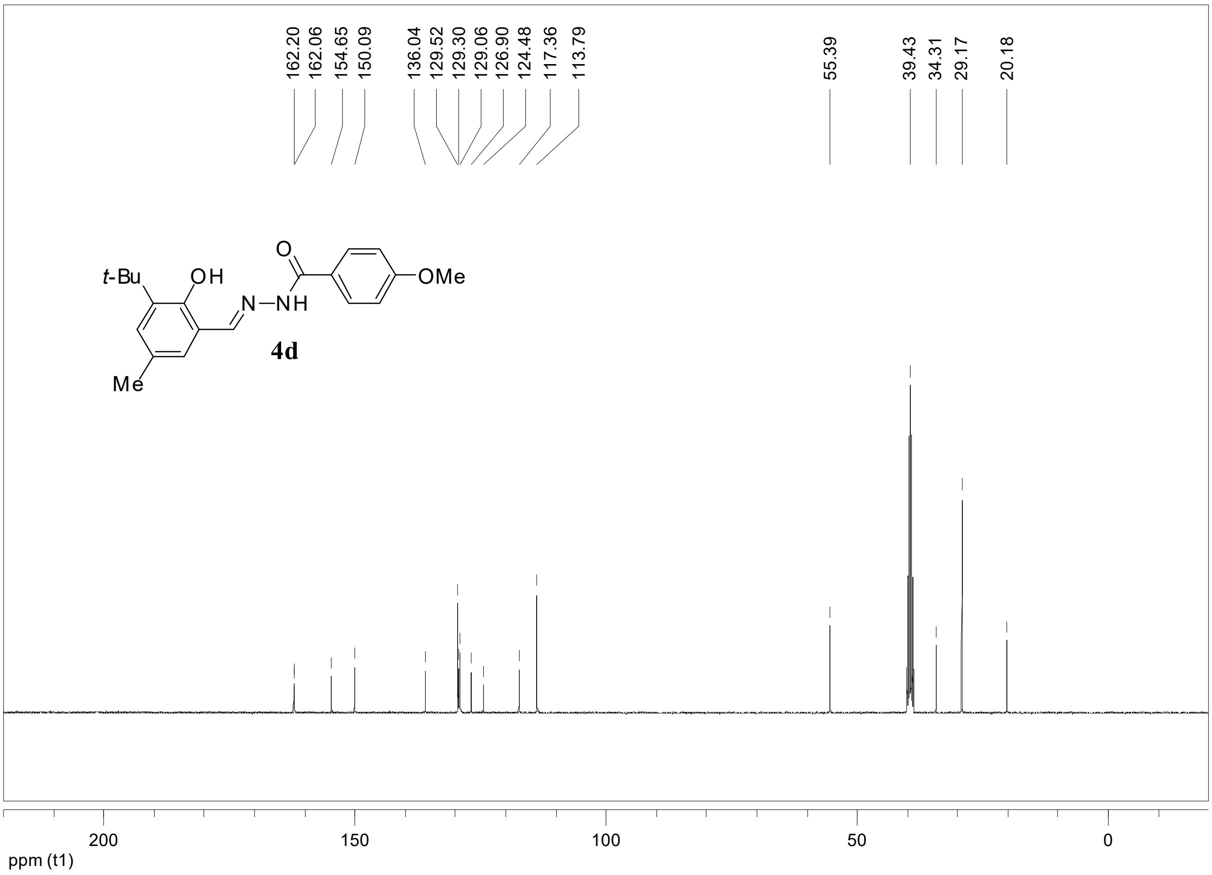

Figure S84. ^{13}C-NMR (100 MHz, DMSO-d_6) spectrum of compound **4d**

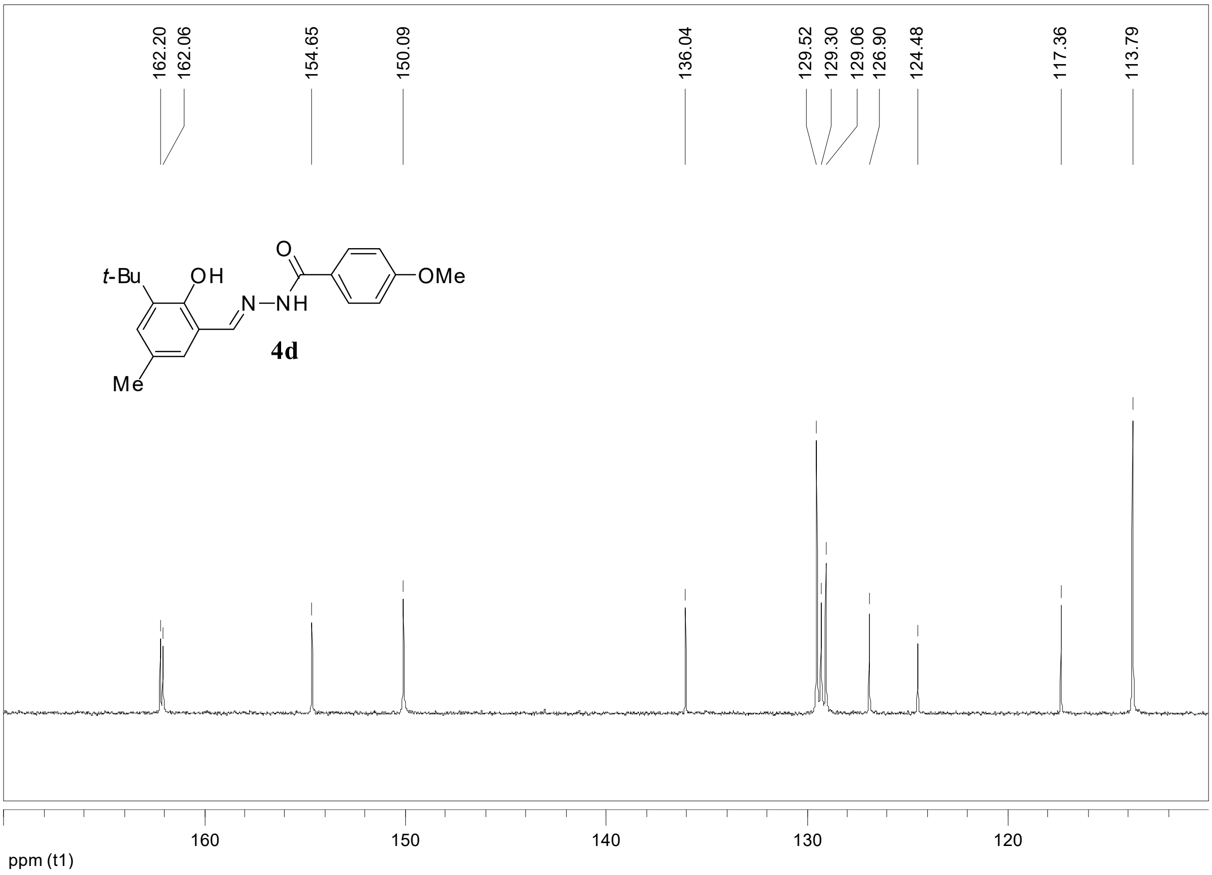

Figure S85. Expansion of ^{13}C-NMR (100 MHz, DMSO-d_6) spectrum of compound **4d**

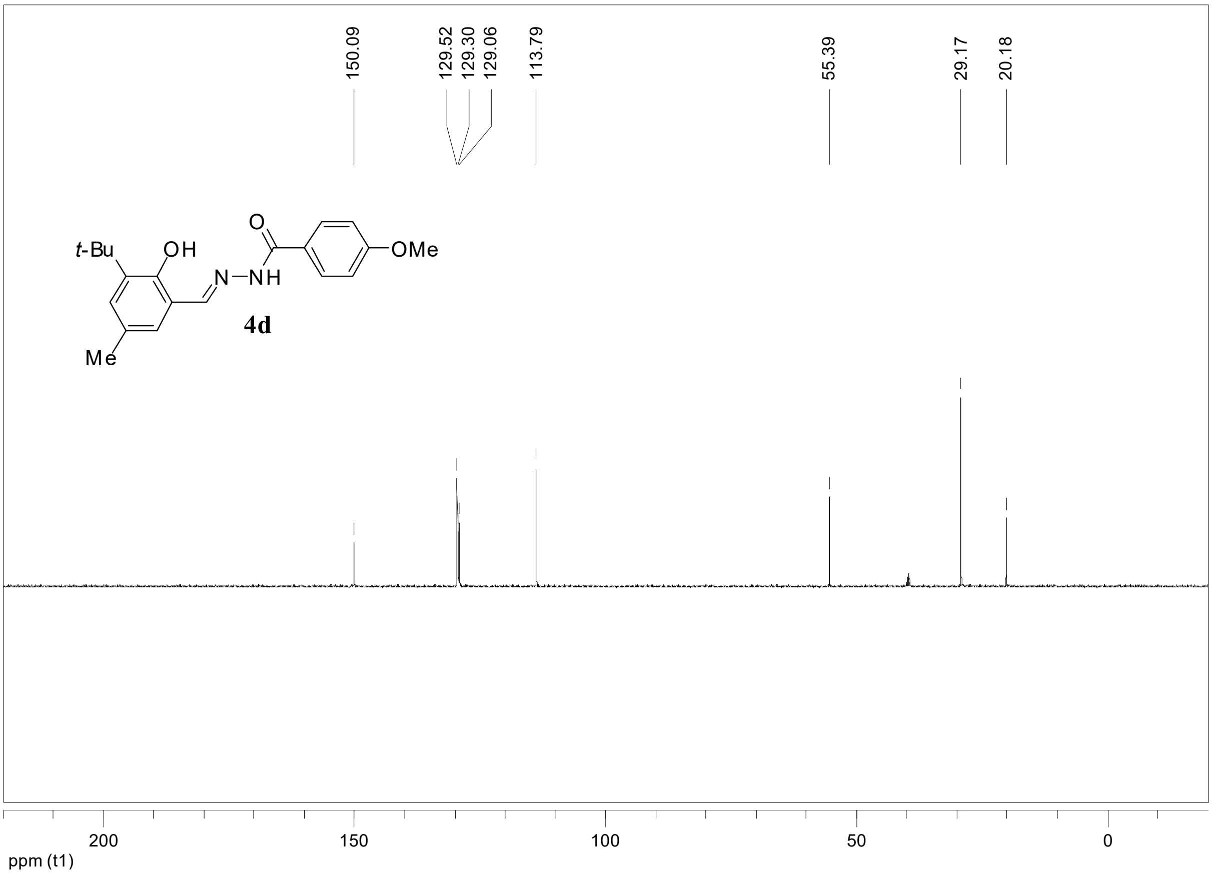

Figure S86. ^{13}C-NMR (100 MHz, DMSO-d_6) dept-135 experiment of compound **4d**

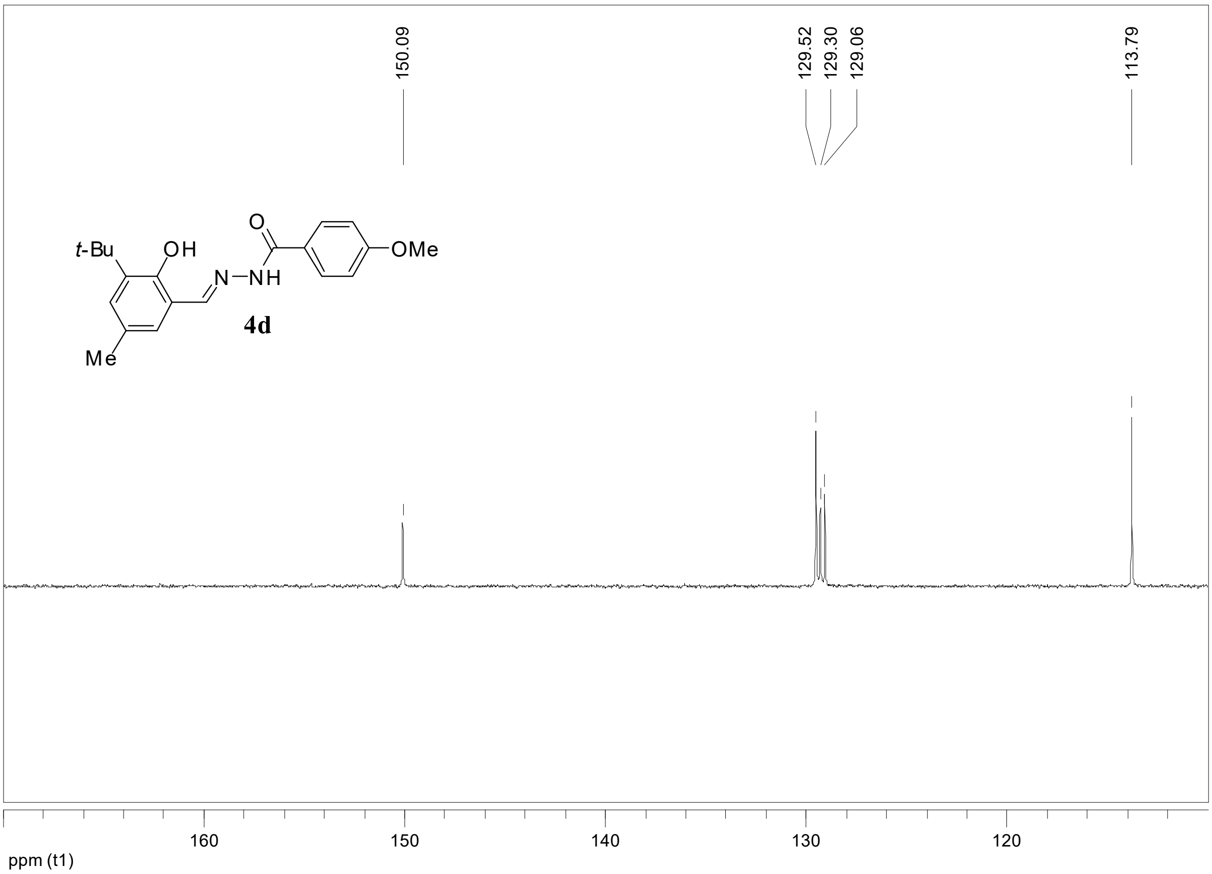

Figure S87. Expansion of ^{13}C-NMR (100 MHz, DMSO-d_6) dept-135 experiment of compound **4d**

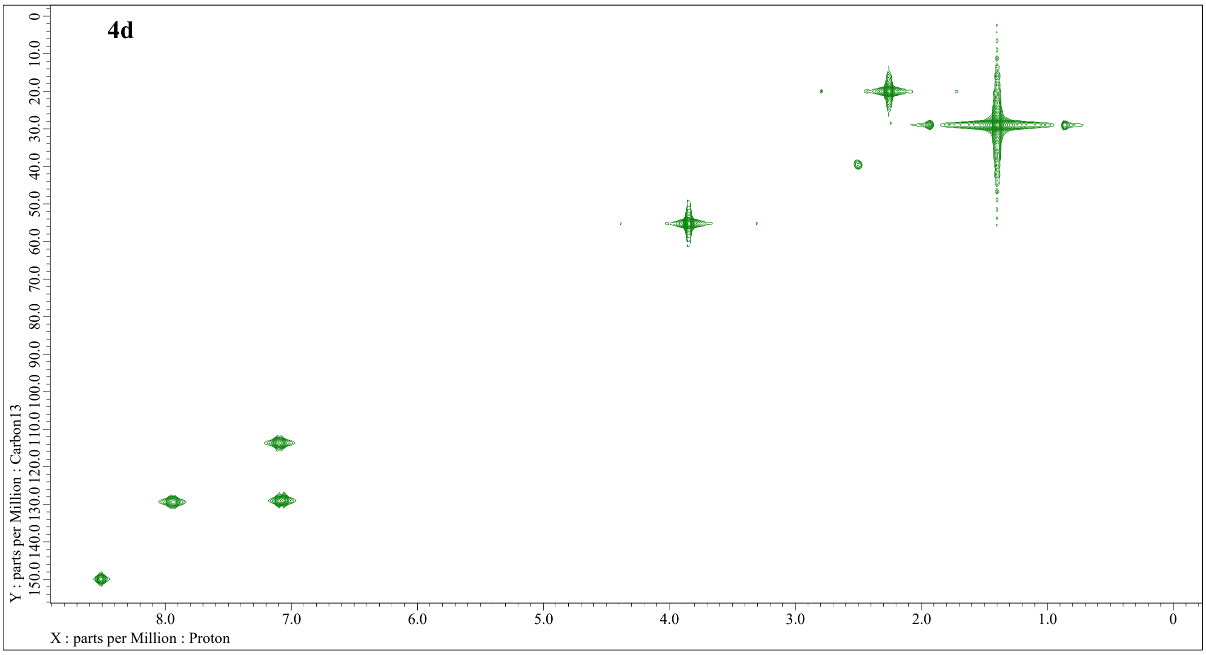

Figure S88. 2D-NMR (400 MHz, DMSO-d_6) HMQC experiment of 4-methoxy-*N*'-[(*E*)-(3-*tert*-butyl-2-hydroxy-5-methylphenyl)methylidene]benzohydrazide (**4d**)

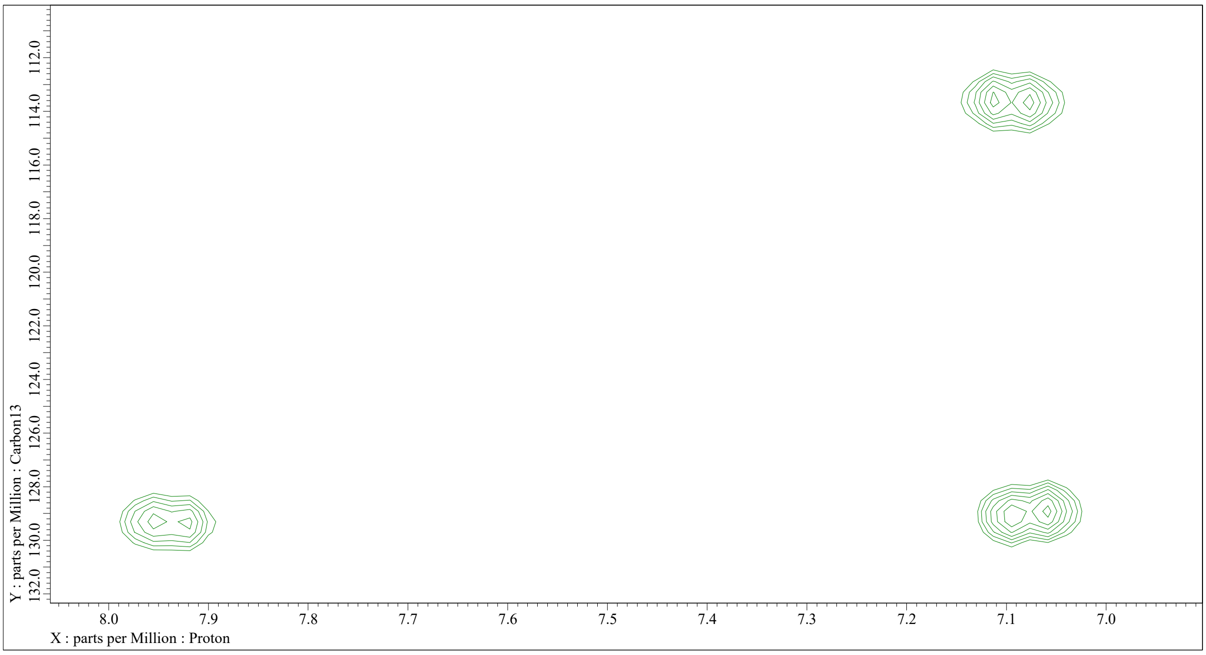

Figure S89. Expansion of 2D-NMR (400 MHz, DMSO-d_6) HMQC experiment of 4-methoxy-*N*'-[(*E*)-(3-*tert*-butyl-2-hydroxy-5-methylphenyl)methylidene]benzohydrazide (**4d**)

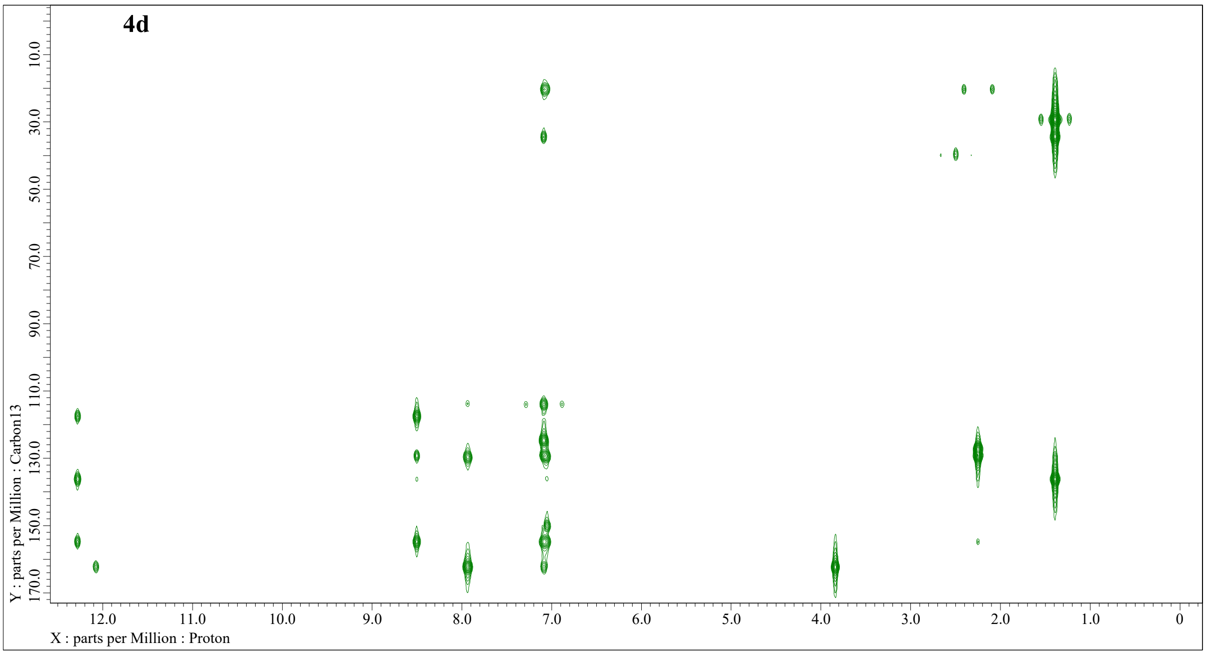

Figure S90. 2D-NMR (400 MHz, DMSO-d_6) HMBC experiment of 4-methoxy-*N*'-[(*E*)-(3-*tert*-butyl-2-hydroxy-5-methylphenyl)methylidene]benzohydrazide (**4d**)

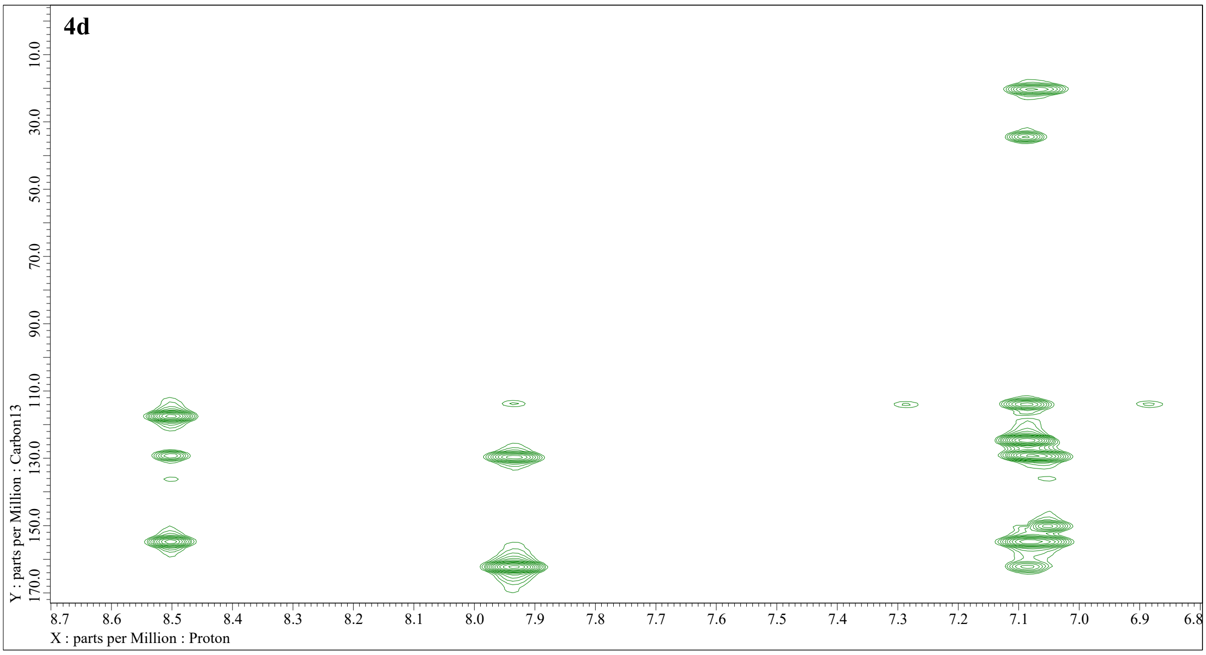

Figure S91. Expansion of 2D-NMR (400 MHz, DMSO-d_6) HMBC experiment of 4-methoxy-*N*'-[(*E*)-(3-*tert*-butyl-2-hydroxy-5-methylphenyl)methylidene]benzohydrazide (**4d**)

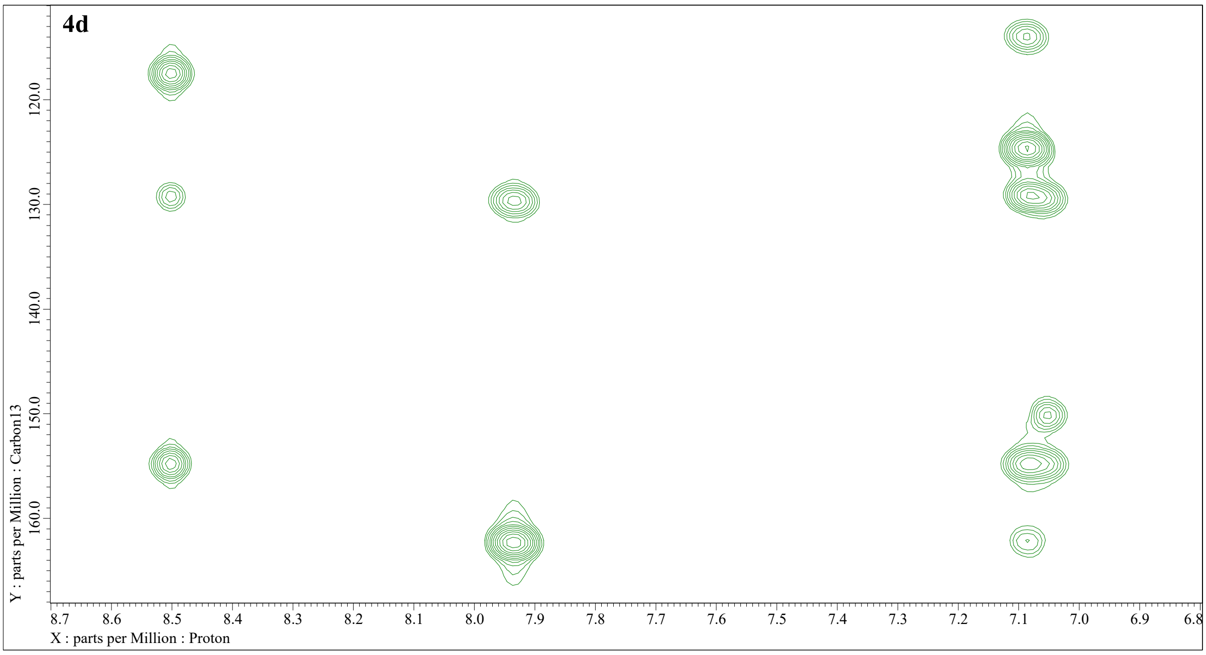

Figure S92. Expansion of 2D-NMR (400 MHz, DMSO-d_6) HMBC experiment of 4-methoxy-*N*'-[(*E*)-(3-*tert*-butyl-2-hydroxy-5-methylphenyl)methylidene]benzohydrazide (**4d**)

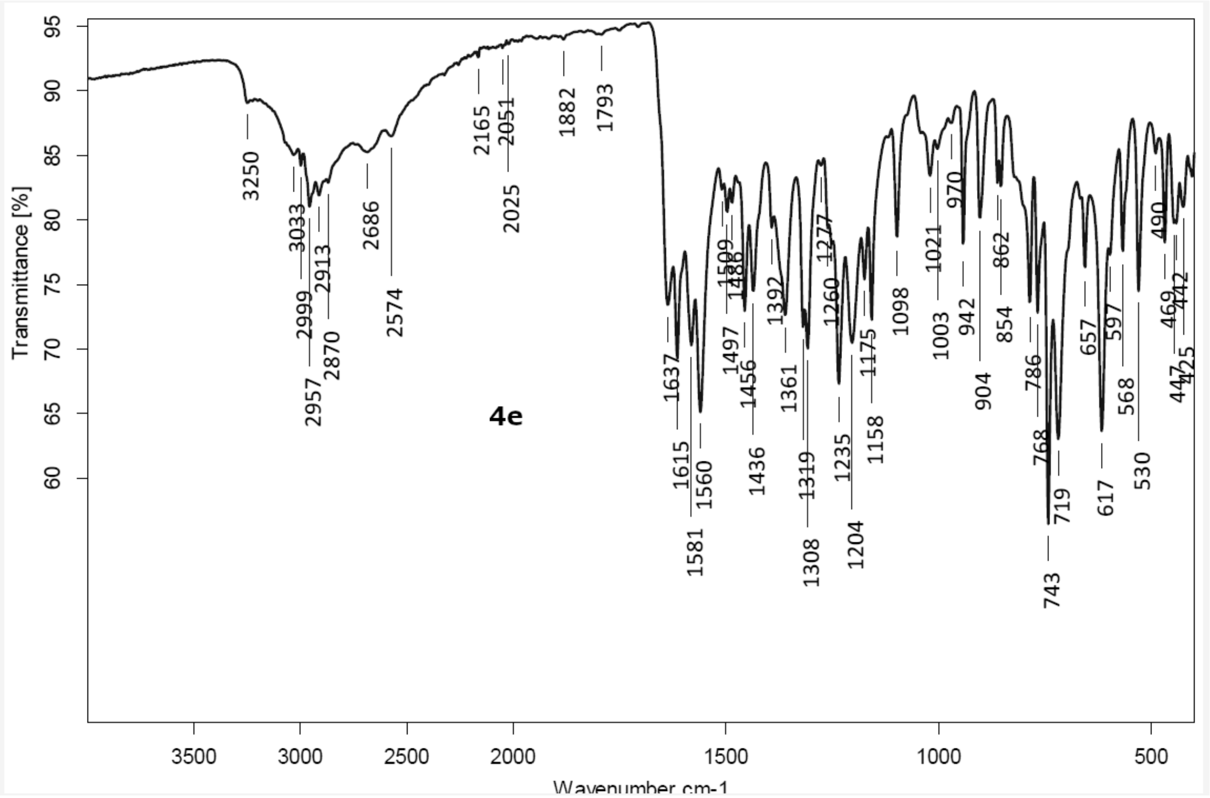

Figure S93. FT-IR (ATR) spectrum of 2-hydroxy-*N*'-[(*E*)-(3-*tert*-butyl-2-hydroxy-5-methylphenyl)methylidene]benzohydrazide (**4e**)

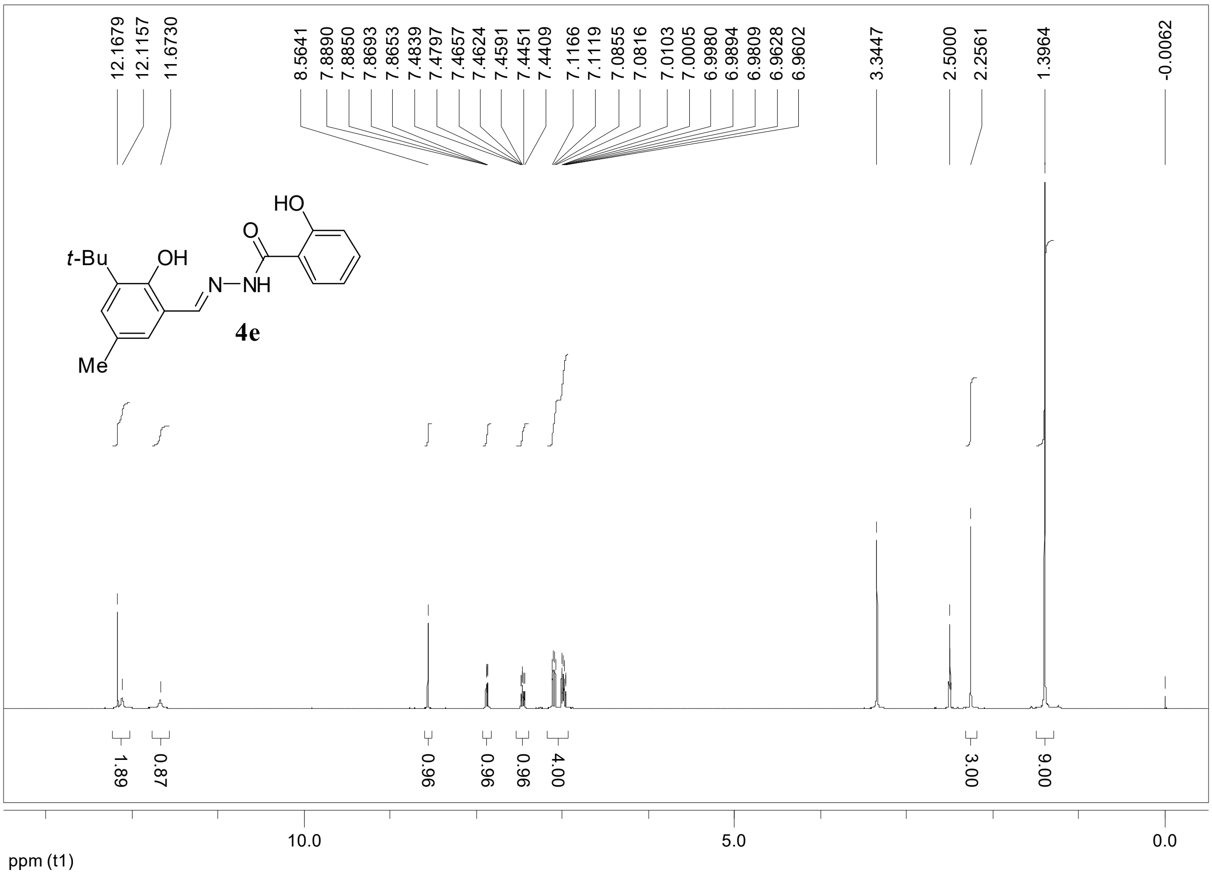

Figure S94. ^{1}H-NMR (400 MHz, DMSO-d_6) spectrum of compound **4e**

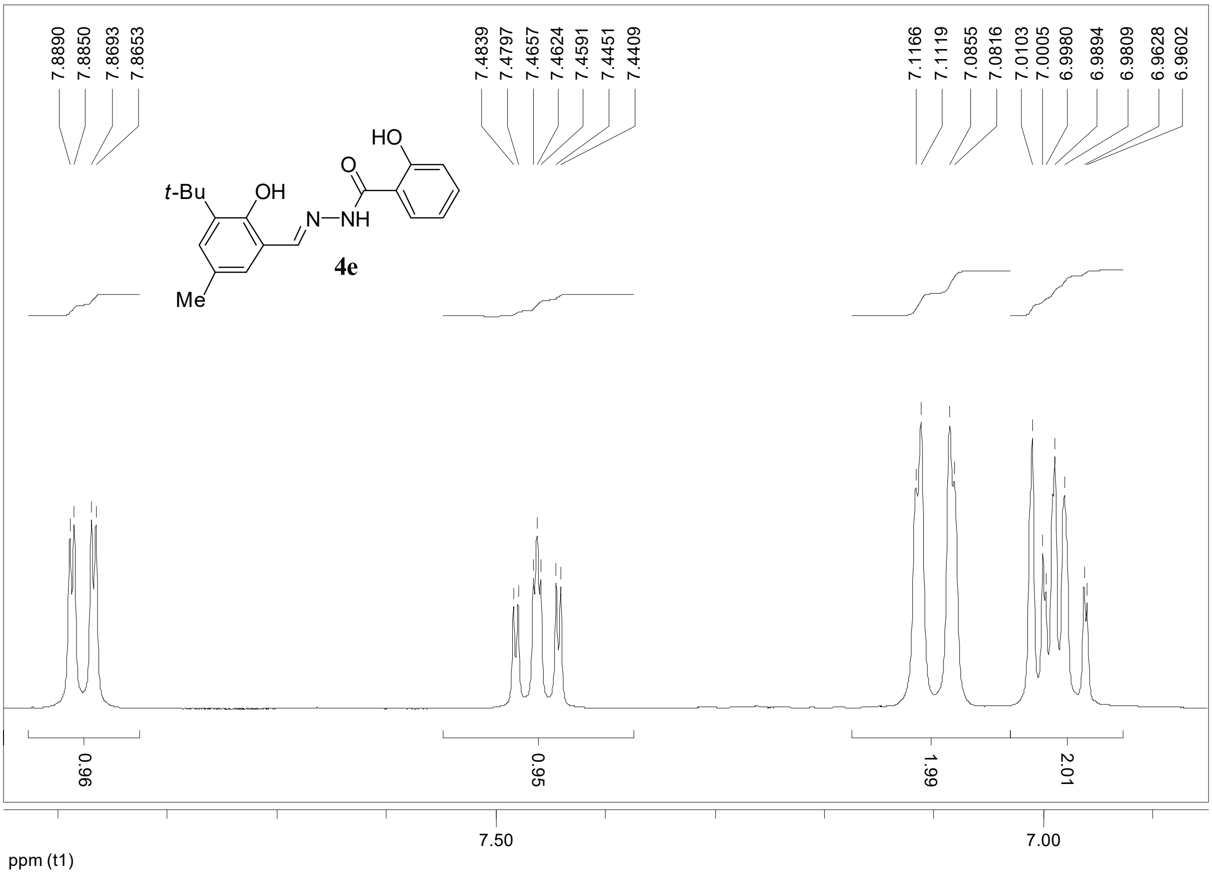

Figure S95. Expansion of ^{1}H-NMR (400 MHz, DMSO-d_6) spectrum of compound **4e**

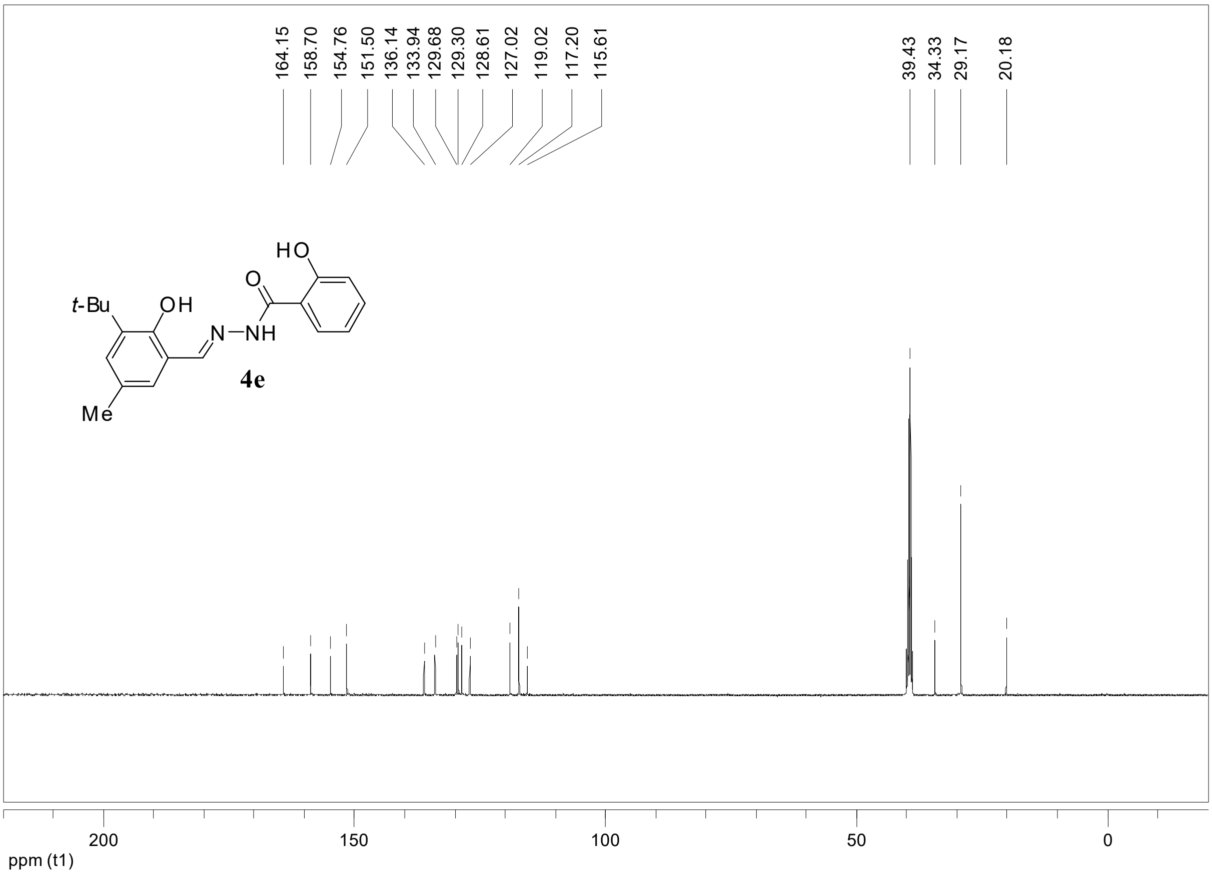

Figure S96. ^{13}C-NMR (100 MHz, DMSO-d_6) spectrum of compound **4e**

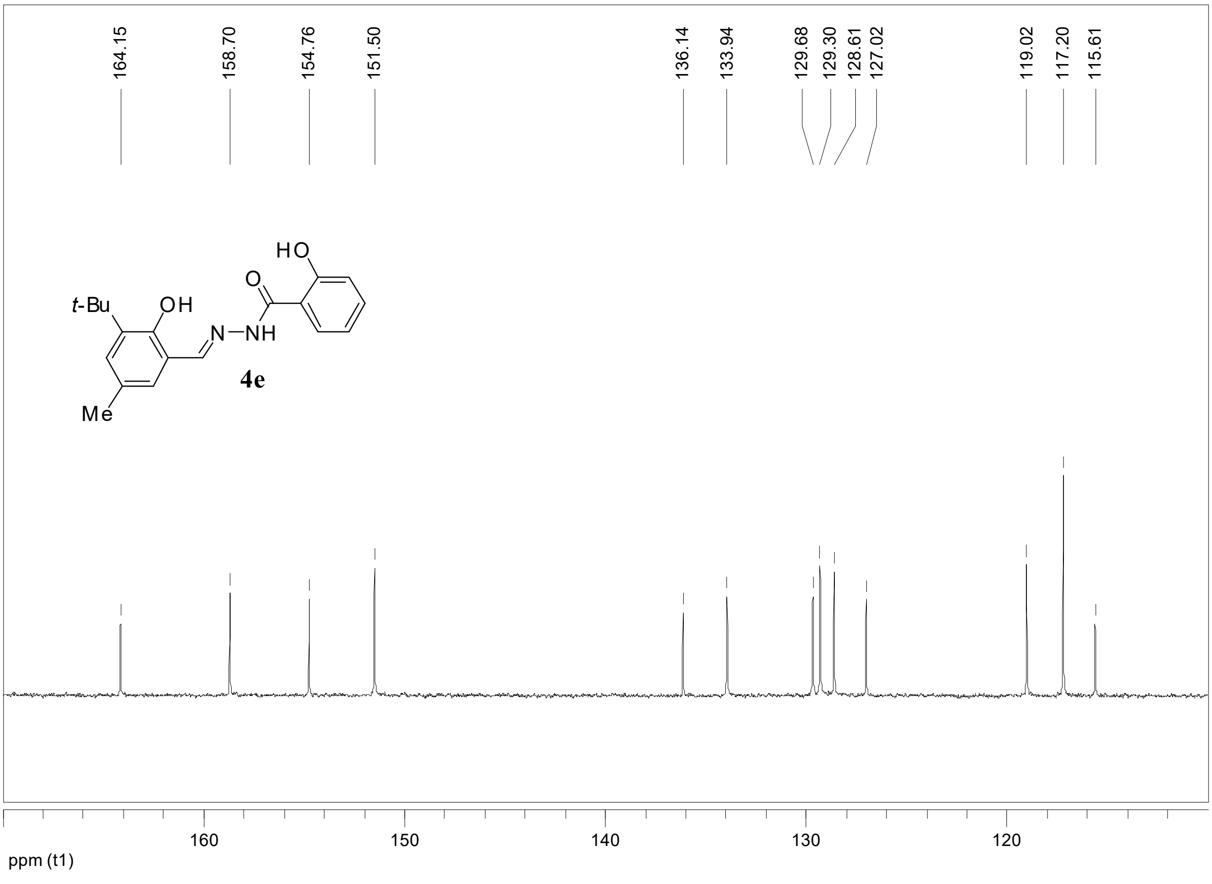

Figure S97. Expansion of ^{13}C-NMR (100 MHz, DMSO-d_6) spectrum of compound **4e**

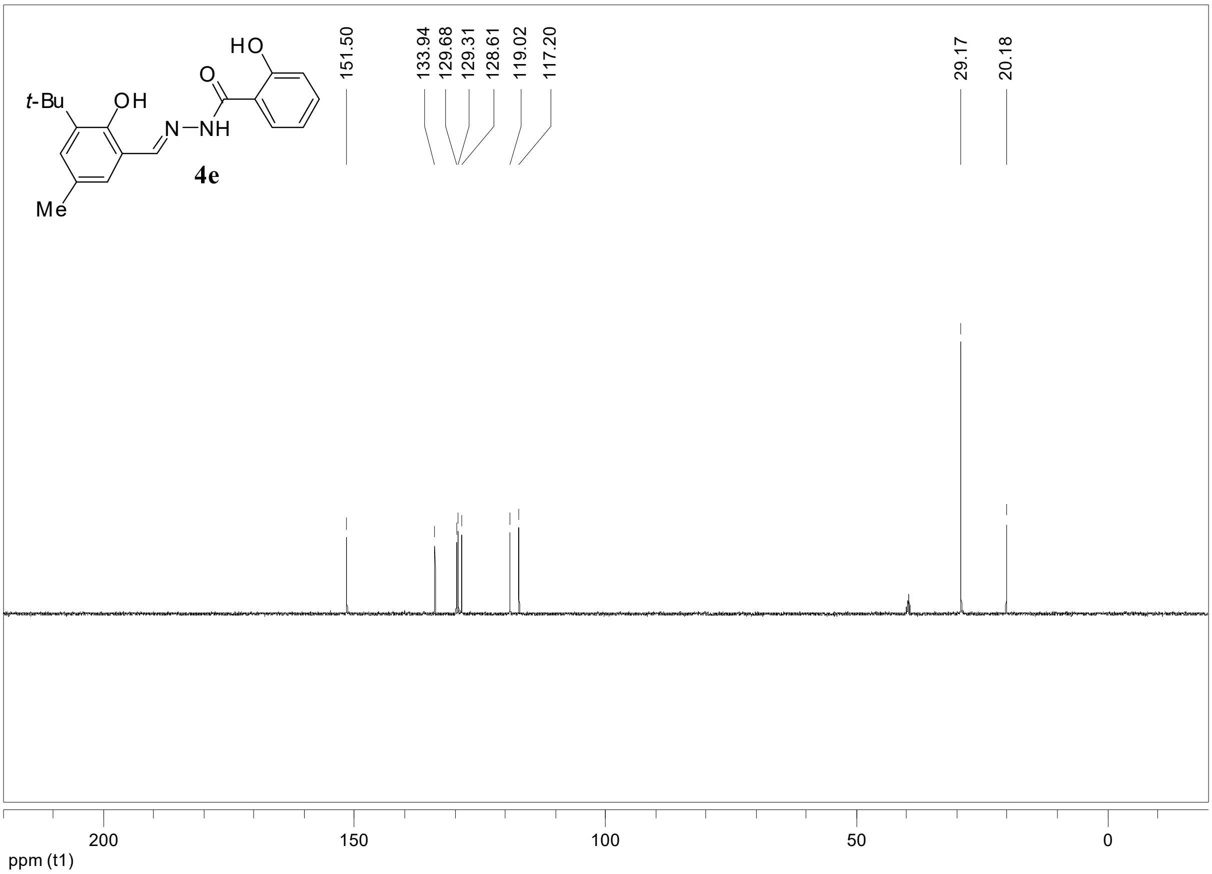

Figure S98. ^{13}C-NMR (100 MHz, DMSO-d_6) dept-135 experiment of compound **4e**

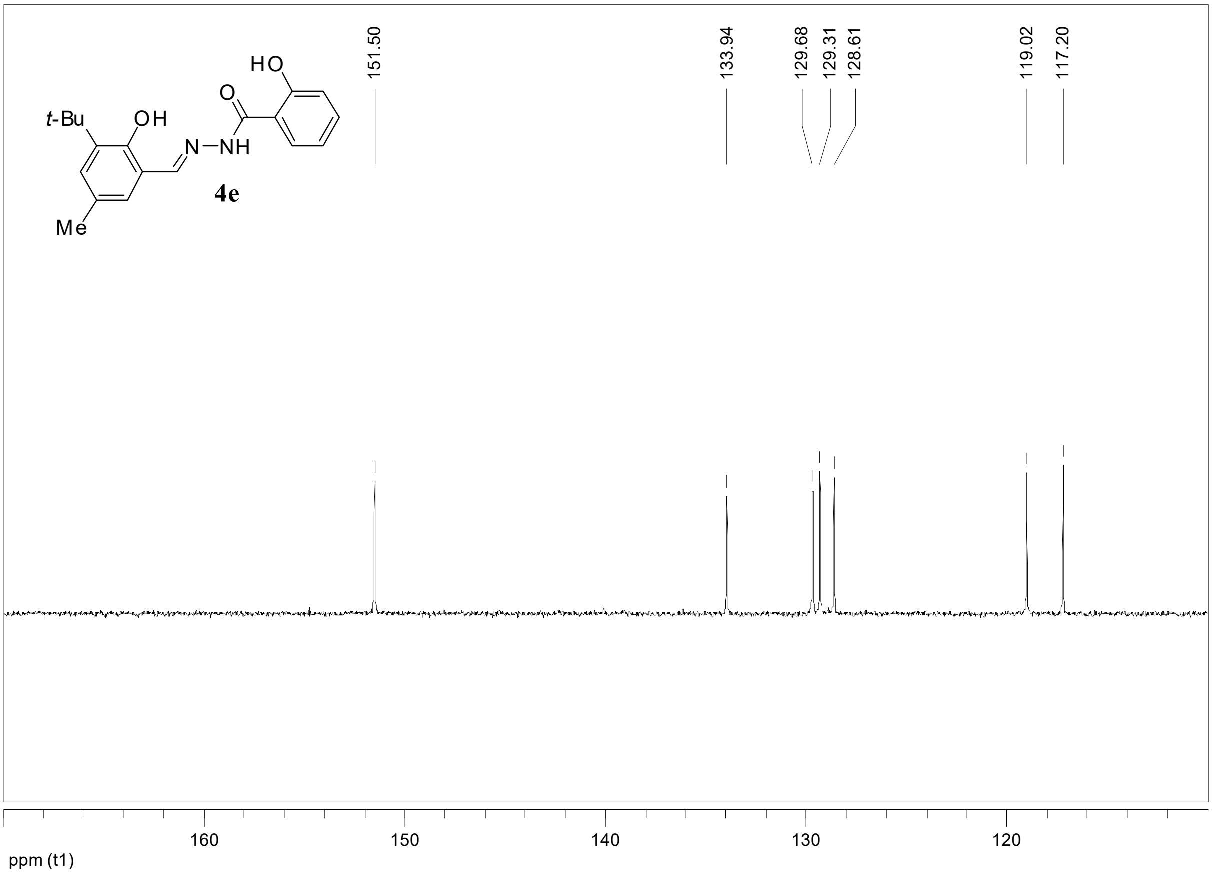

Figure S99. Expansion of ^{13}C-NMR (100 MHz, DMSO-d_6) dept-135 experiment of compound **4e**

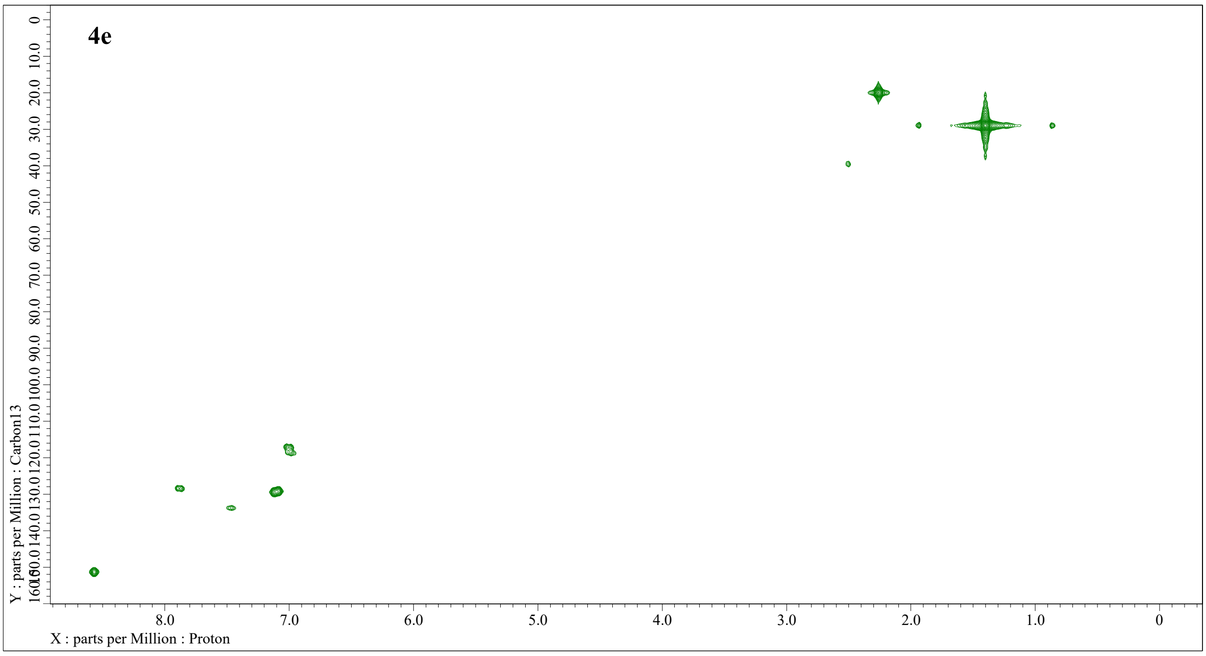

Figure S100. 2D-NMR (400 MHz, DMSO-d_6) HMQC experiment of 2-hydroxy-*N*'-[(*E*)-(3-*tert*-butyl-2-hydroxy-5-methylphenyl)methylidene]benzohydrazide (**4e**)

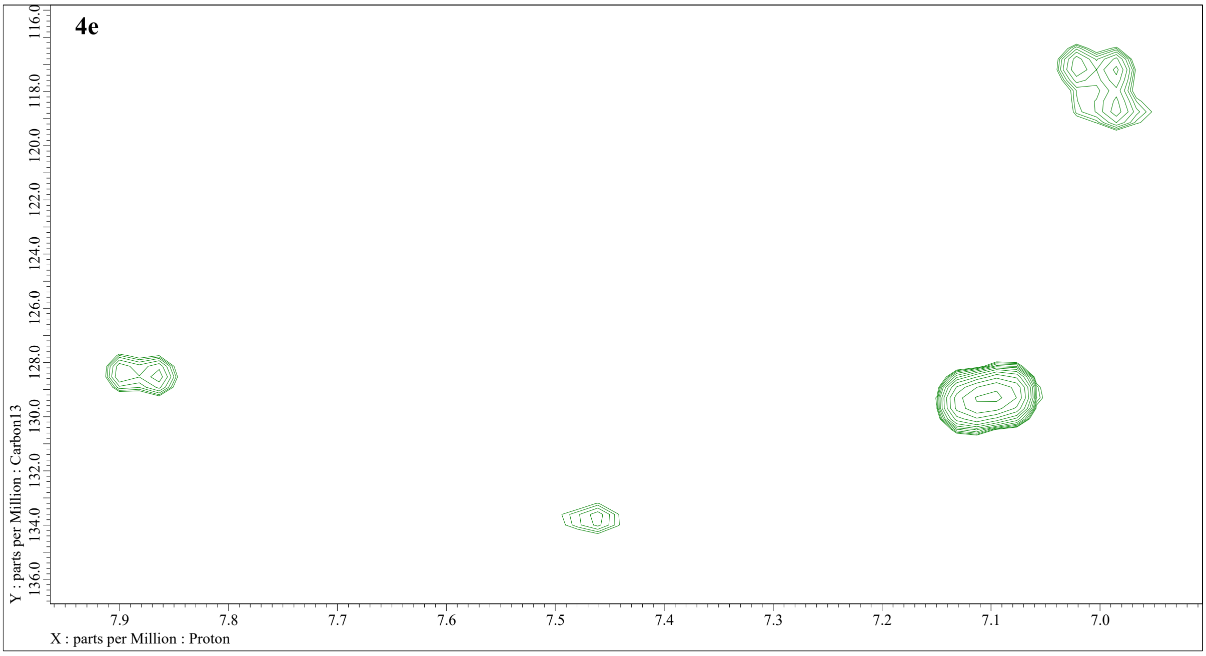

Figure S101. Expansion of 2D-NMR (400 MHz, DMSO-d_6) HMQC experiment of 2-hydroxy-*N*'-[(*E*)-(3-*tert*-butyl-2-hydroxy-5-methylphenyl)methylidene]benzohydrazide (**4e**)

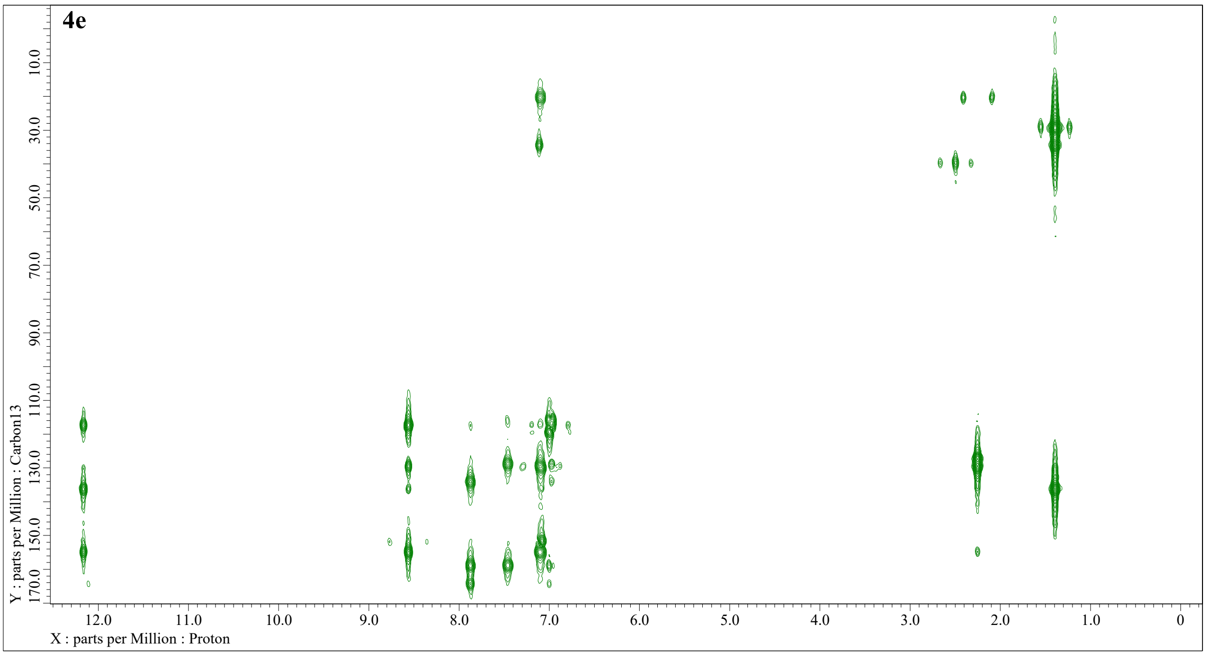

Figure S102. 2D-NMR (400 MHz, DMSO-d_6) HMBC experiment of 2-hydroxy-*N'*-[(*E*)-(3-*tert*-butyl-2-hydroxy-5-methylphenyl)methylidene]benzohydrazide (**4e**)

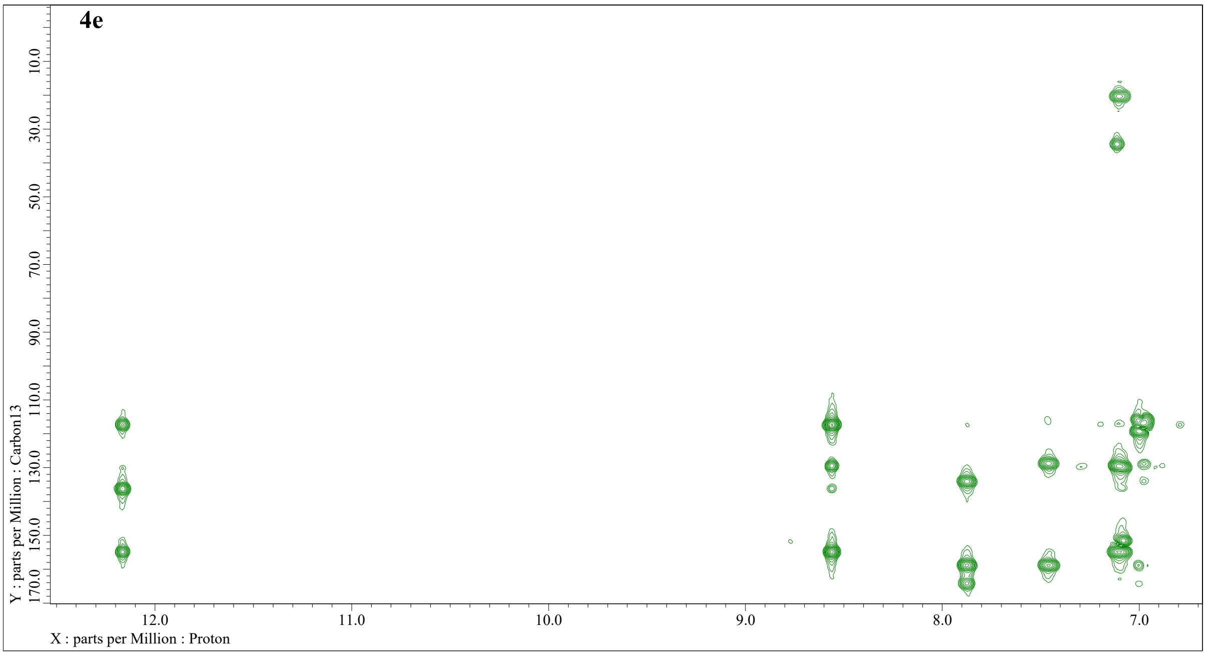

Figure S103. Expansion of 2D-NMR (400 MHz, DMSO-d_6) HMBC experiment of 2-hydroxy-*N'*-[(*E*)-(3-*tert*-butyl-2-hydroxy-5-methylphenyl)methylidene]benzohydrazide (**4e**)

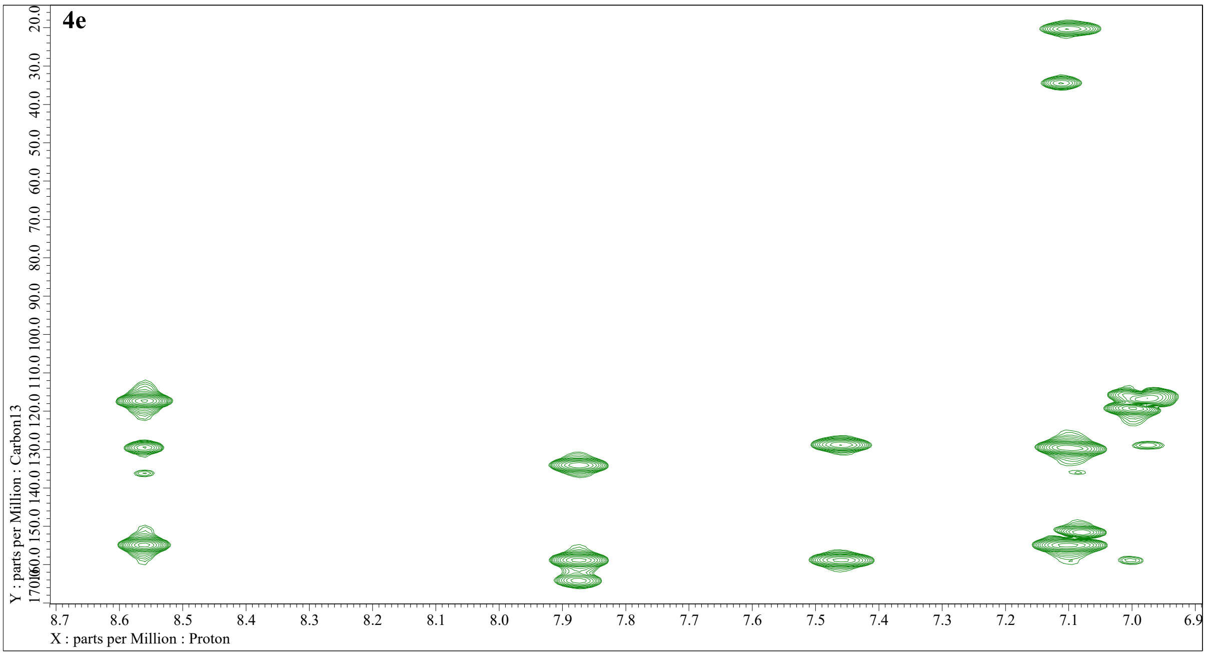

Figure S104. Expansion of 2D-NMR (400 MHz, DMSO-d_6) HMBC experiment of 2-hydroxy-*N*'-[(*E*)-(3-*tert*-butyl-2-hydroxy-5-methylphenyl)methylidene]benzohydrazide (**4e**)

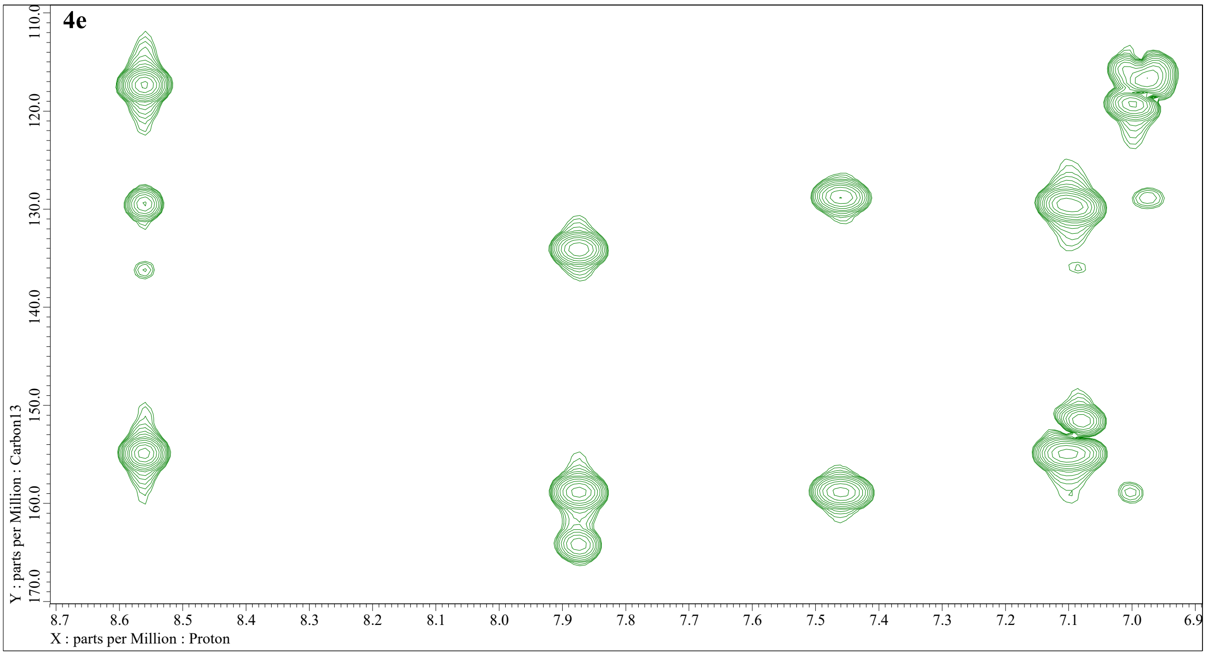

Figure S105. Expansion of 2D-NMR (400 MHz, DMSO-d_6) HMBC experiment of 2-hydroxy-*N*'-[(*E*)-(3-*tert*-butyl-2-hydroxy-5-methylphenyl)methylidene]benzohydrazide (**4e**)

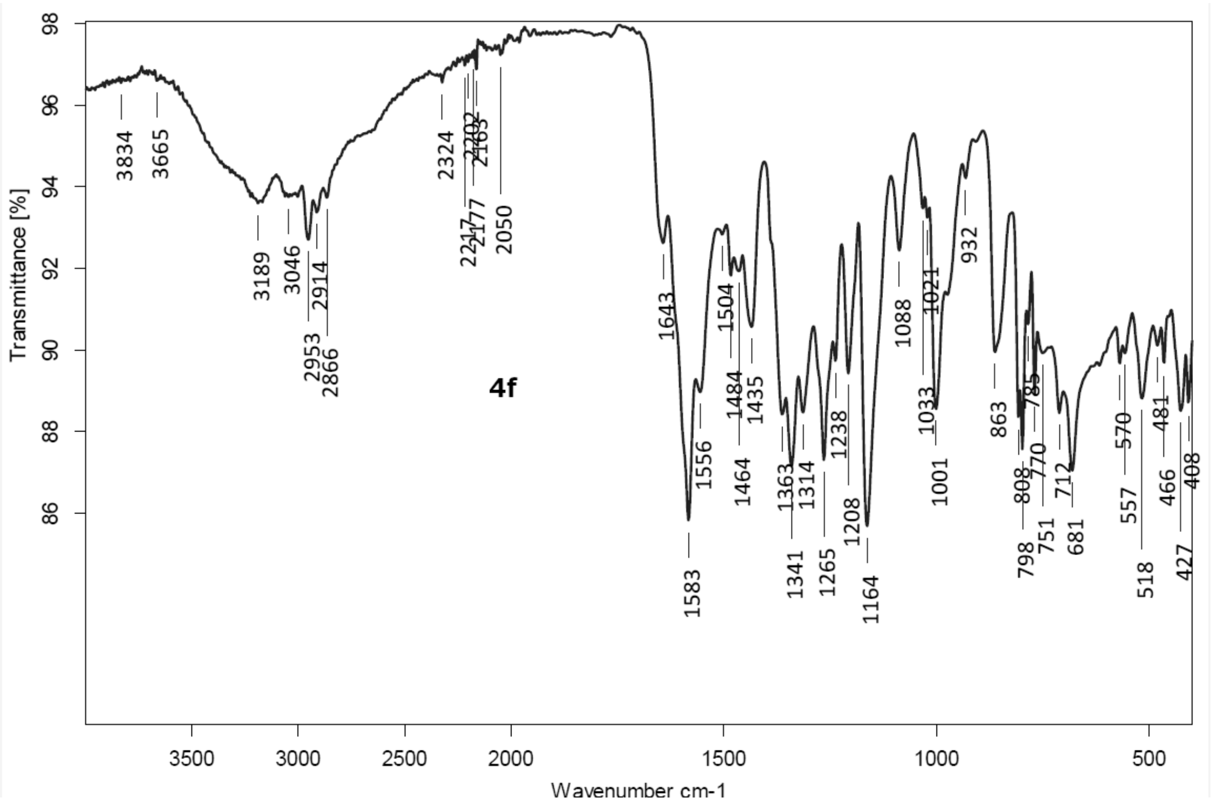

Figure S106. FT-IR (ATR) spectrum of 3,5-dihydroxy-*N*'-[(*E*)-(3-*tert*-butyl-2-hydroxy-5-methylphenyl)methylidene]benzohydrazide (**4f**)

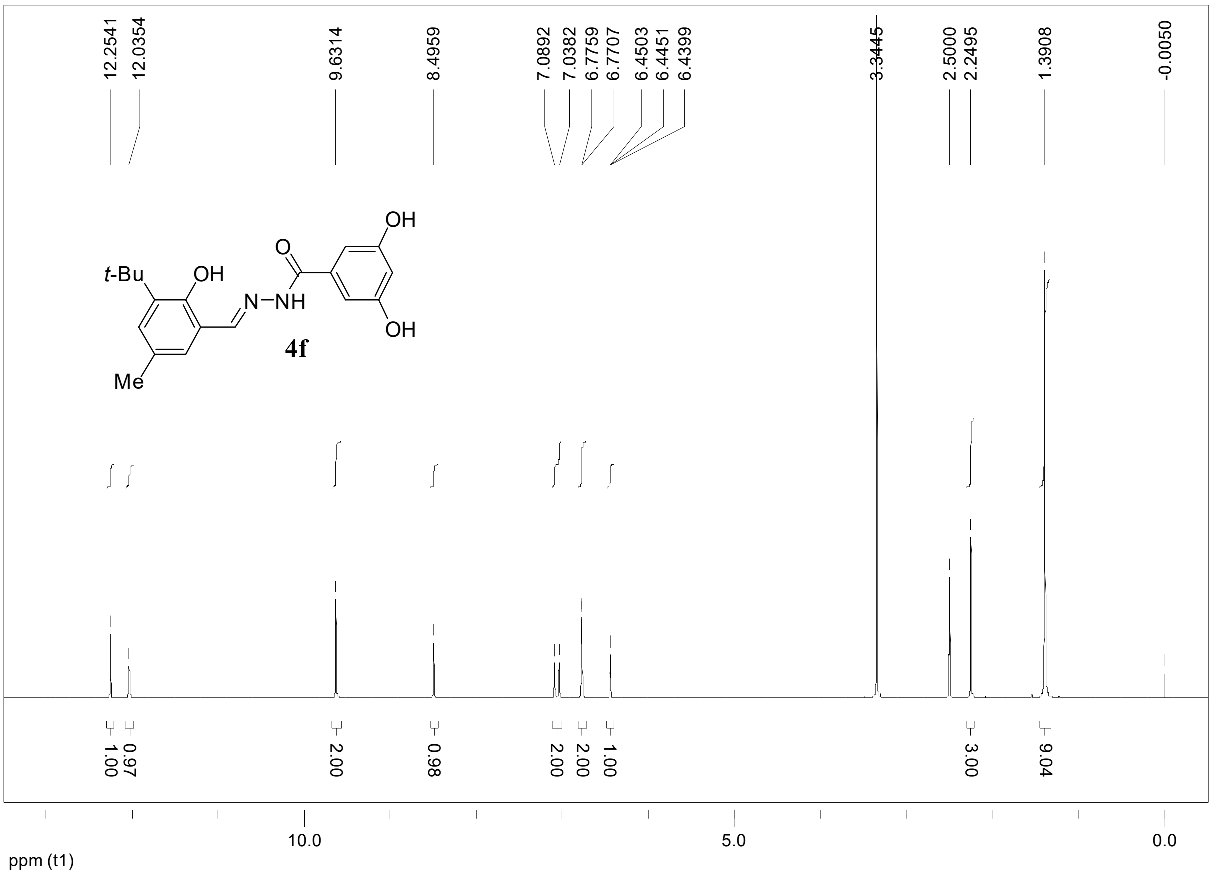

Figure S107. ^{1}H-NMR (400 MHz, DMSO-d_6) spectrum of compound **4f**

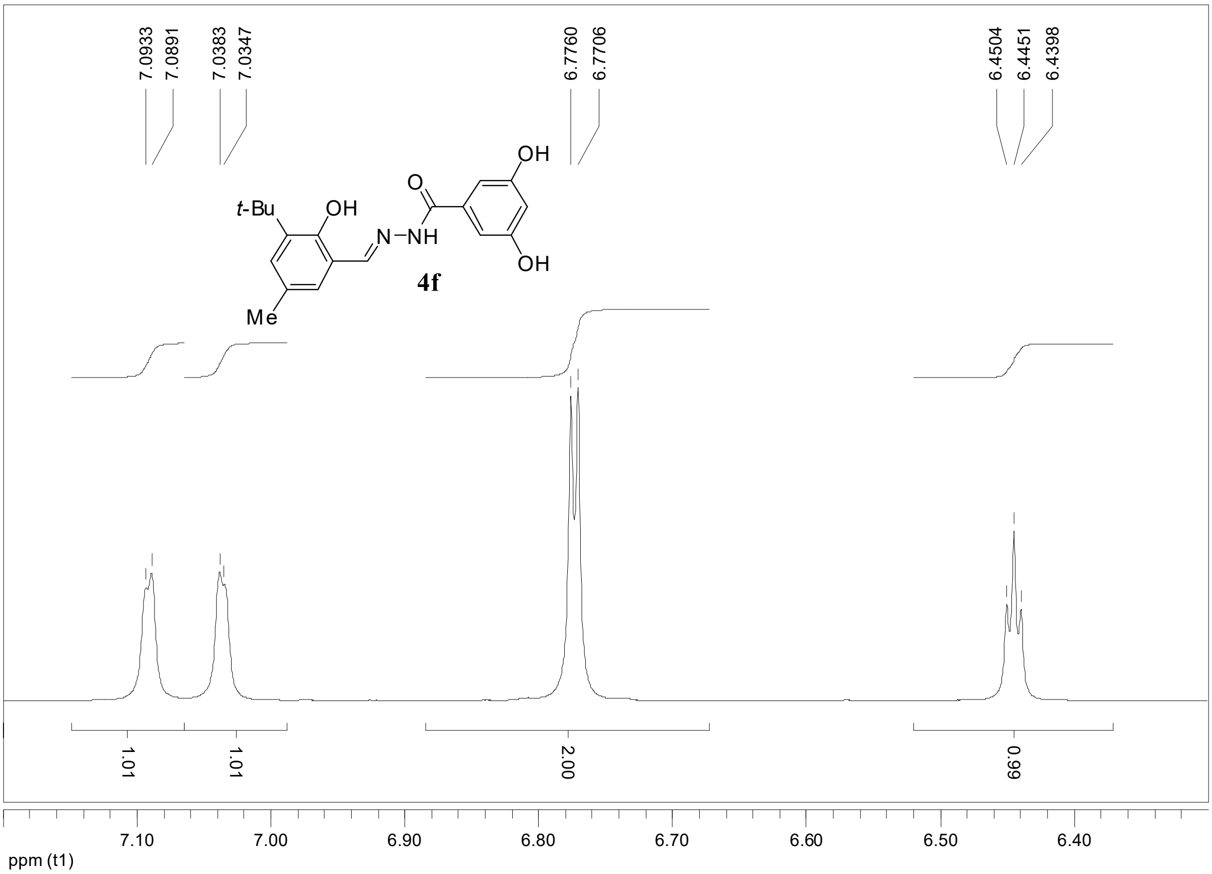

Figure S108. Expansion of ^{1}H-NMR (400 MHz, DMSO-d_6) spectrum of compound **4f**

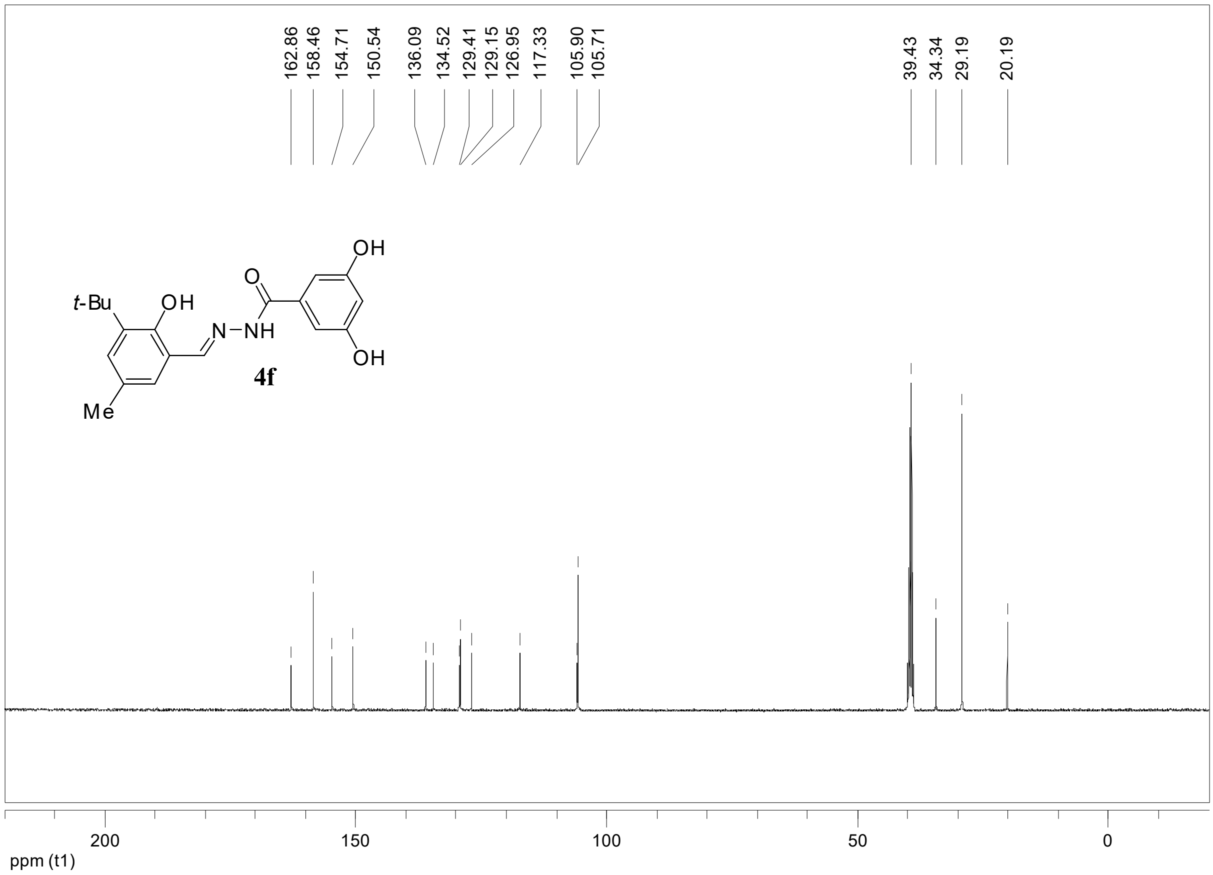

Figure S109. ^{13}C-NMR (100 MHz, DMSO-d_6) spectrum of compound **4f**

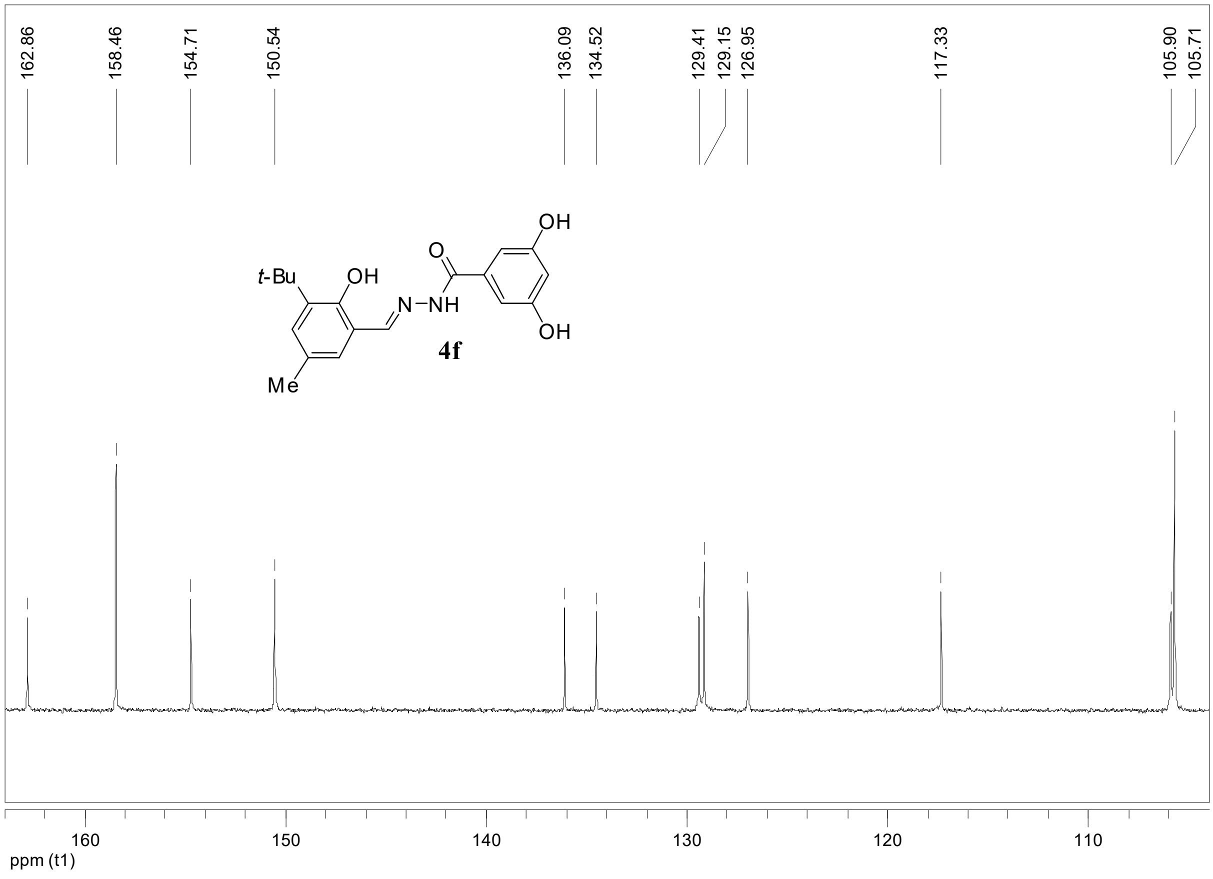

Figure S110. Expansion of ^{13}C-NMR (100 MHz, DMSO-d_6) spectrum of compound **4f**

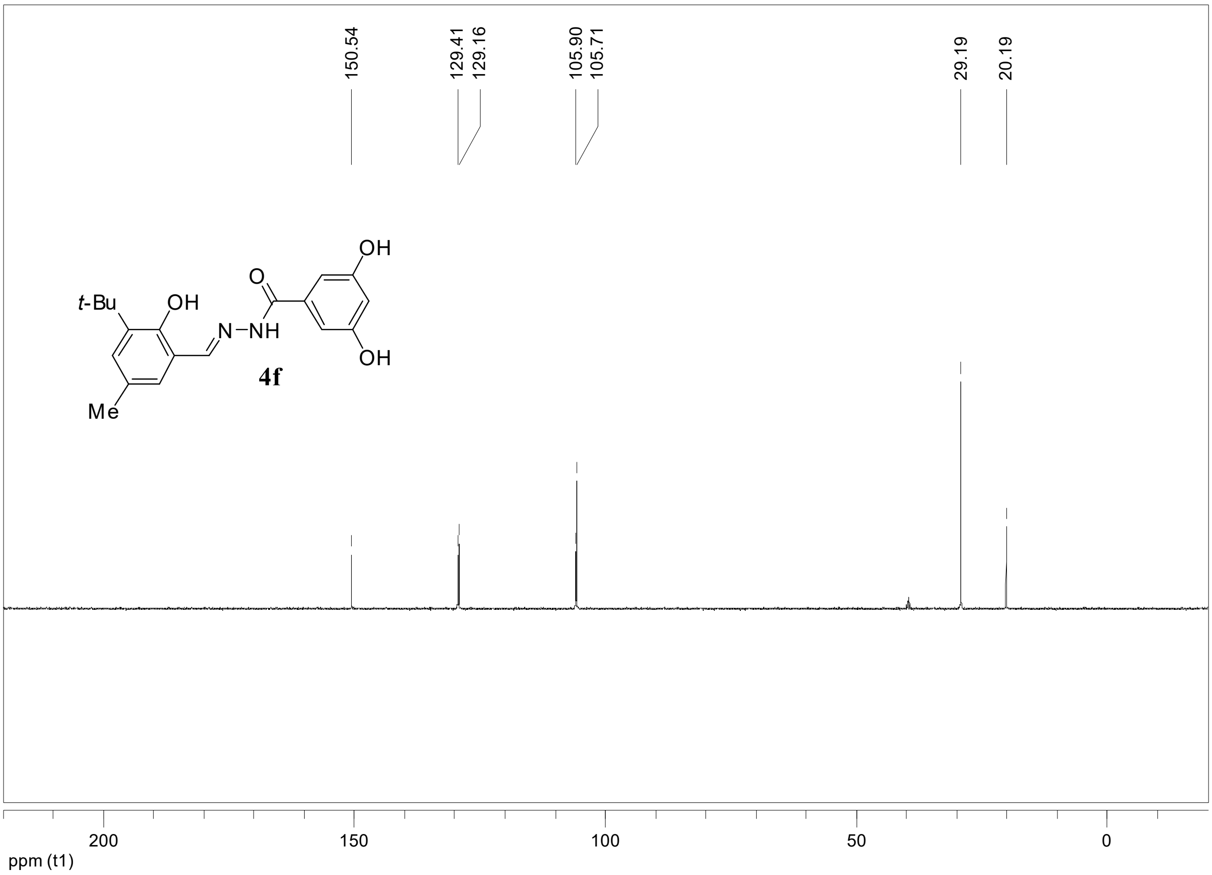

Figure S111. ^{13}C-NMR (100 MHz, DMSO-d_6) dept-135 experiment of compound **4f**

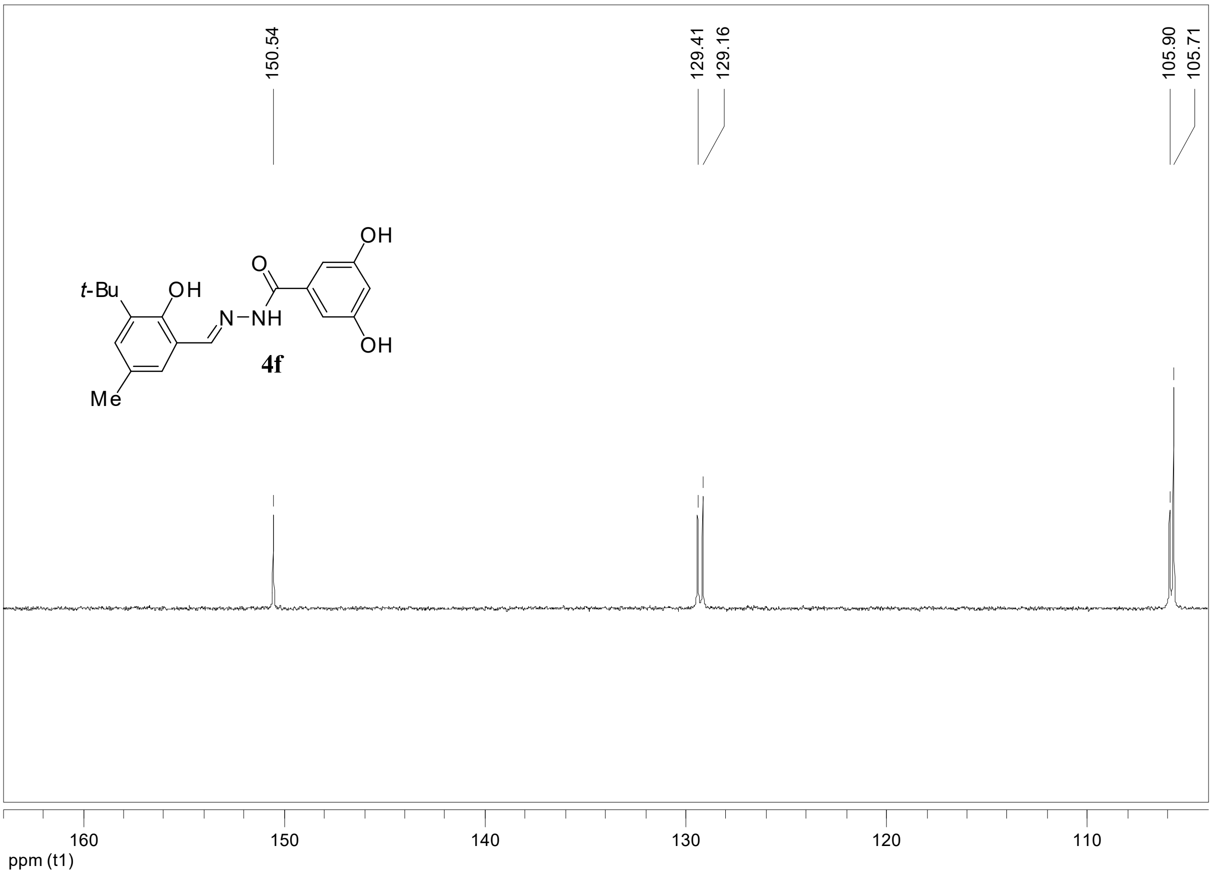

Figure S112. Expansion of ^{13}C-NMR (100 MHz, DMSO-d_6) dept-135 experiment of compound **4f**

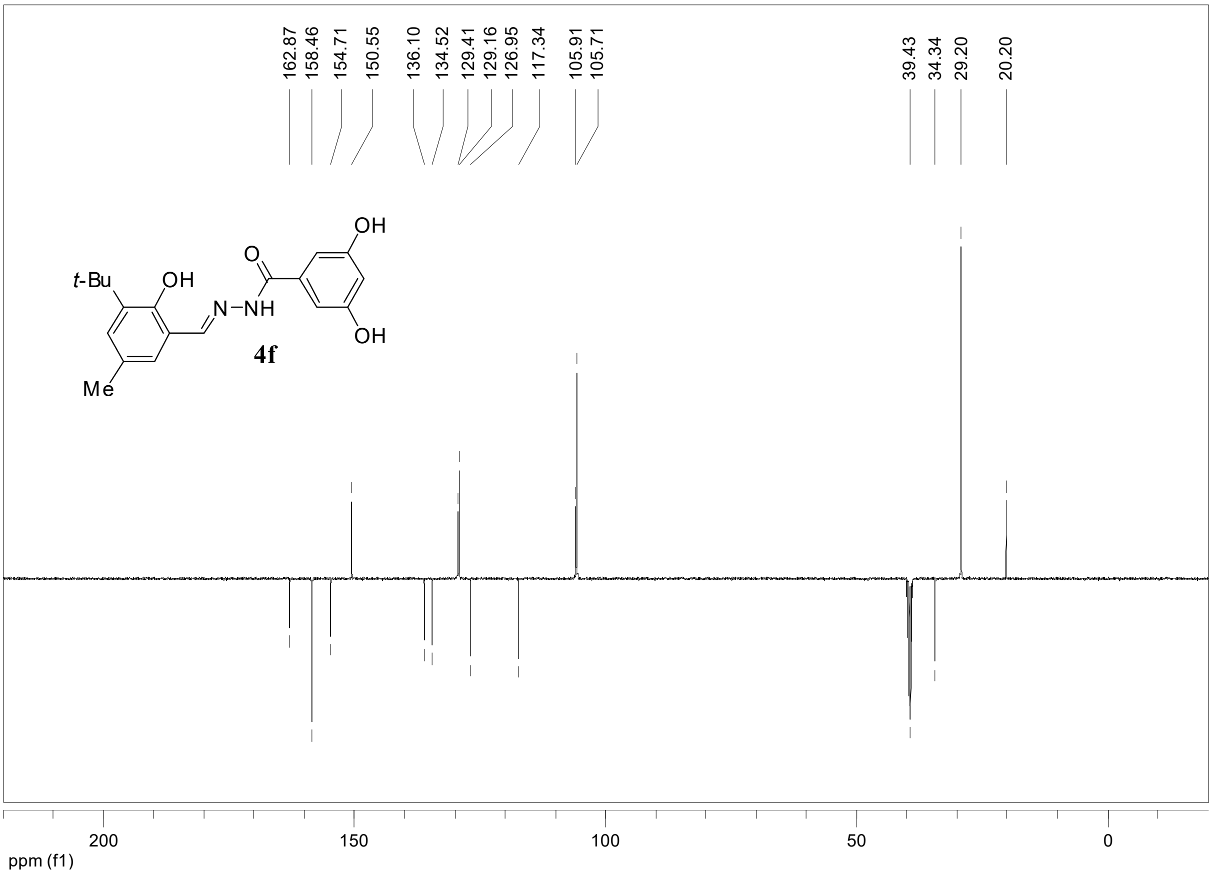

Figure S113. ^{13}C-NMR (100 MHz, DMSO-d_6) ATP experiment of compound **4f**

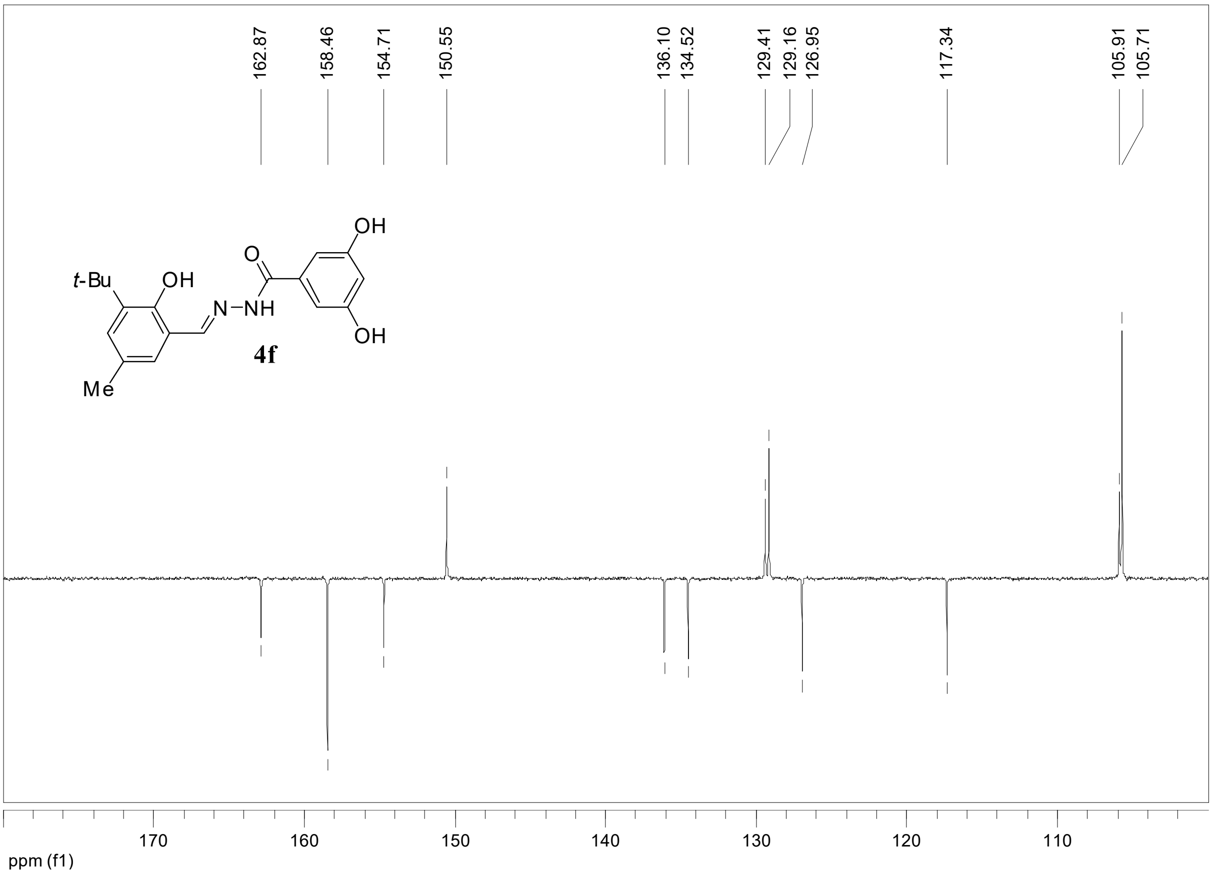

Figure S114. Expansion of ^{13}C-NMR (100 MHz, DMSO-d_6) ATP experiment of compound **4f**

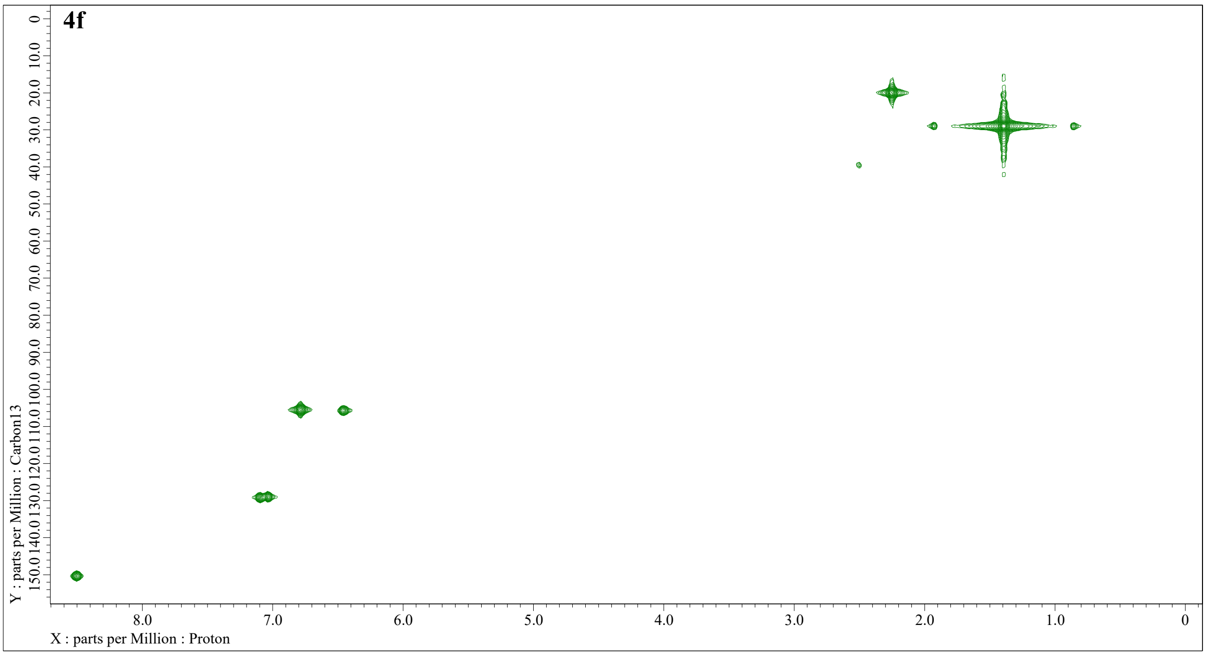

Figure S115. 2D-NMR (400 MHz, DMSO-d_6) HMQC experiment of 3,5-dihydroxy-*N'*-[(*E*)-(3-*tert*-butyl-2-hydroxy-5-methylphenyl)methylidene]benzohydrazide (**4f**)

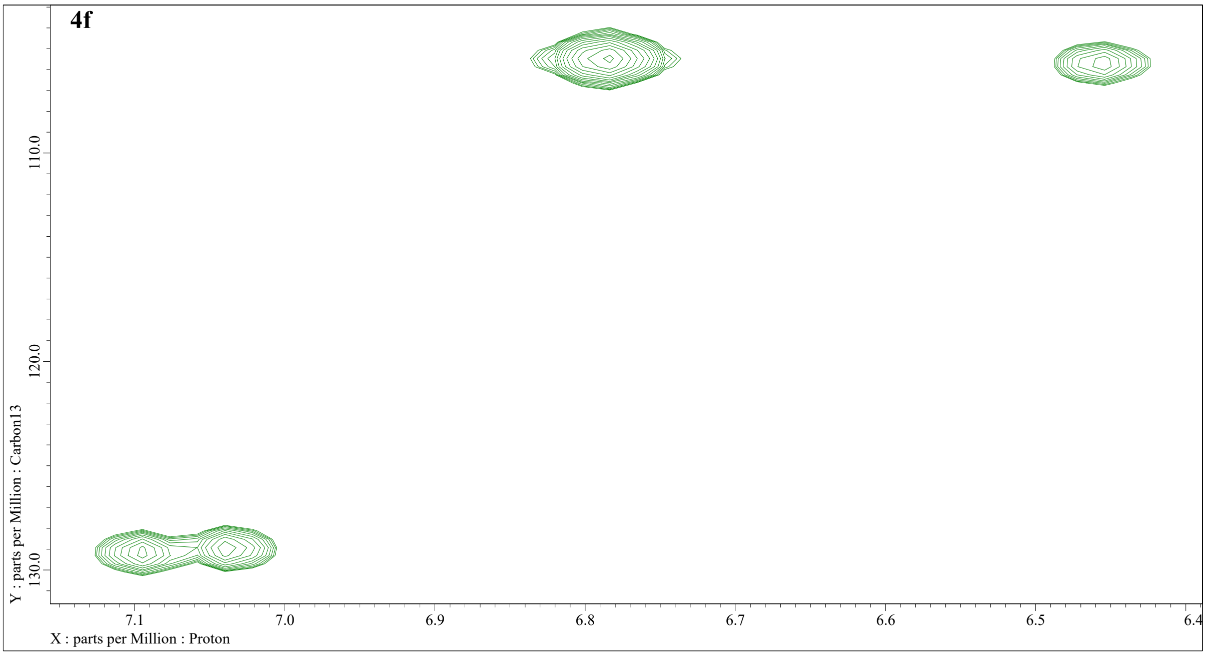

Figure S116. Expansion of 2D-NMR (400 MHz, DMSO-d_6) HMQC experiment of 3,5-dihydroxy-*N'*-[(*E*)-(3-*tert*-butyl-2-hydroxy-5-methylphenyl)methylidene]benzohydrazide (**4f**)

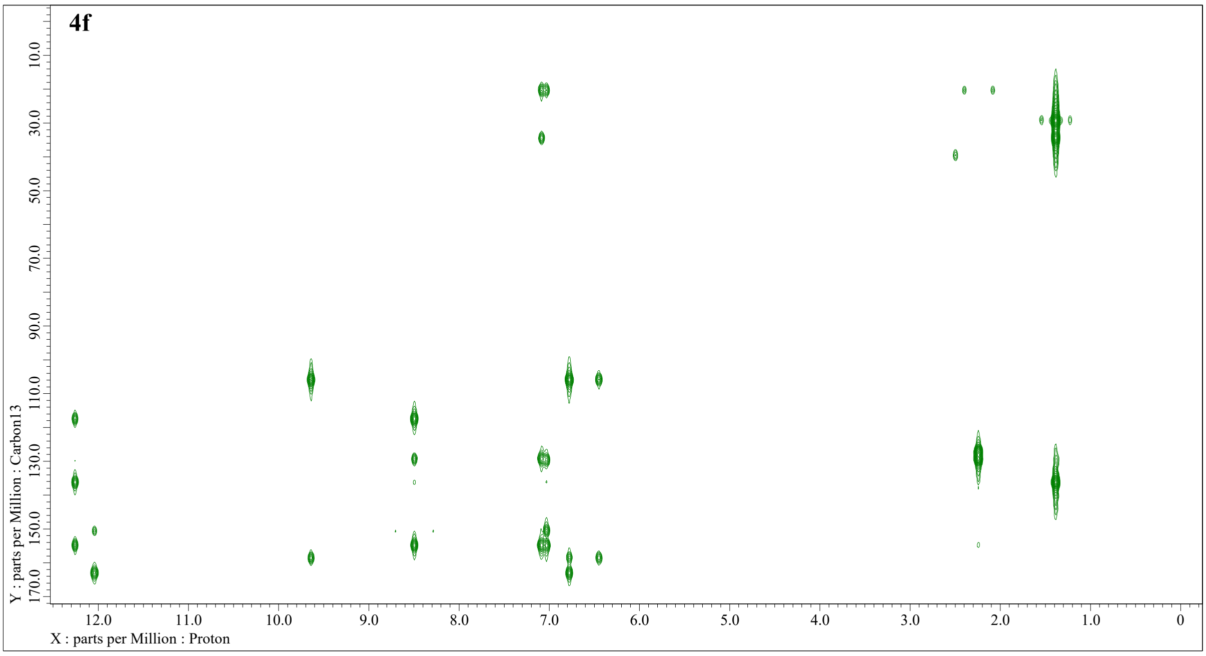

Figure S117. 2D-NMR (400 MHz, DMSO-d_6) HMBC experiment of 3,5-dihydroxy-*N*'-[(*E*)-(3-*tert*-butyl-2-hydroxy-5-methylphenyl)methylidene]benzohydrazide (**4f**)

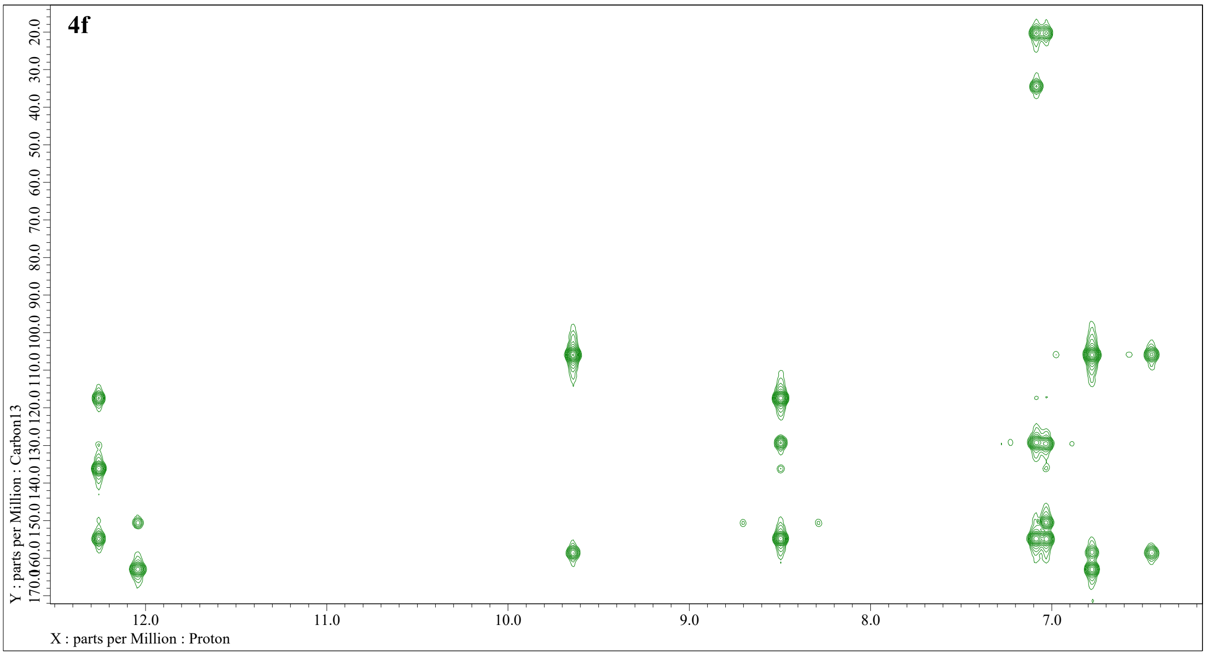

Figure S118. Expansion of 2D-NMR (400 MHz, DMSO-d_6) HMBC experiment of 3,5-dihydroxy-*N*'-[(*E*)-(3-*tert*-butyl-2-hydroxy-5-methylphenyl)methylidene]benzohydrazide (**4f**)

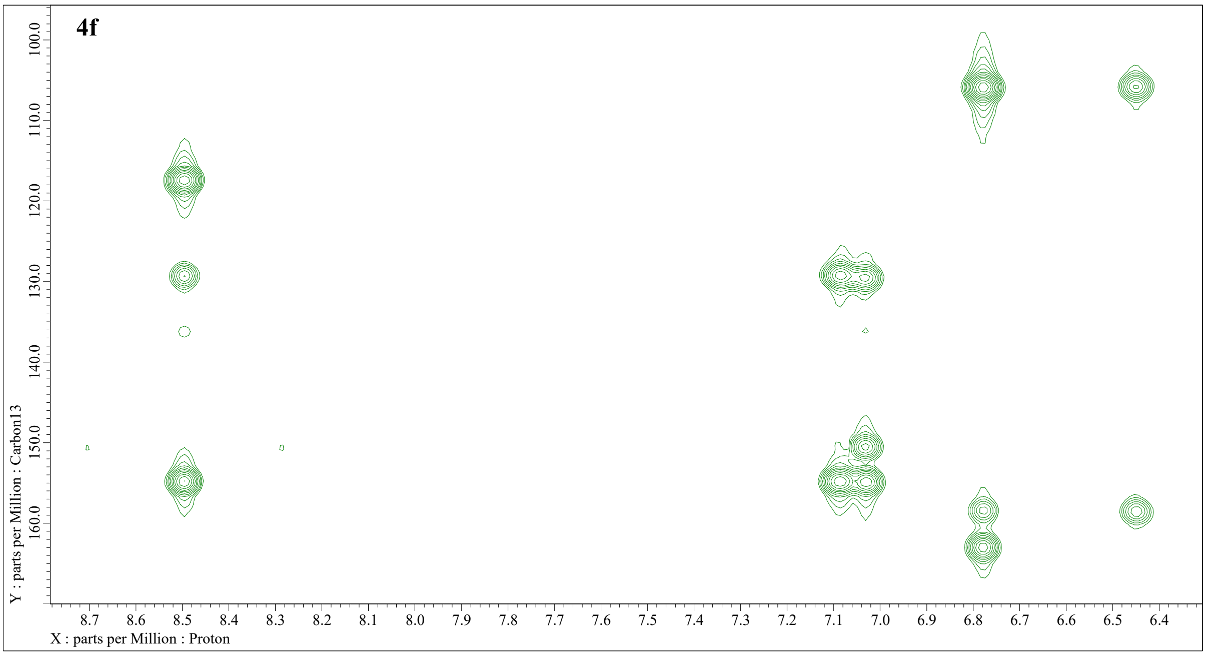

Figure S119. Expansion of 2D-NMR (400 MHz, DMSO-d_6) HMBC experiment of 3,5-dihydroxy-*N*'-[(*E*)-(3-*tert*-butyl-2-hydroxy-5-methylphenyl)methylidene]benzohydrazide (**4f**)

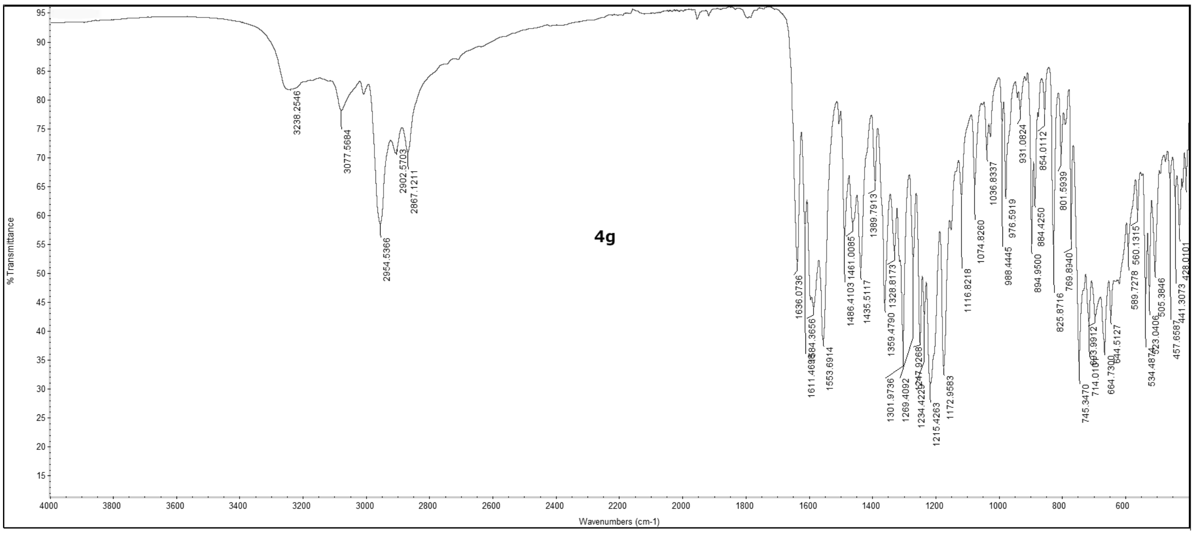

Figure S120. FT-IR (ATR) spectrum of 2-hydroxy-*N*'-[(*E*)-(3,5-di-*tert*-butyl-2-hydroxyphenyl)methylidene]benzohydrazide (**4g**)

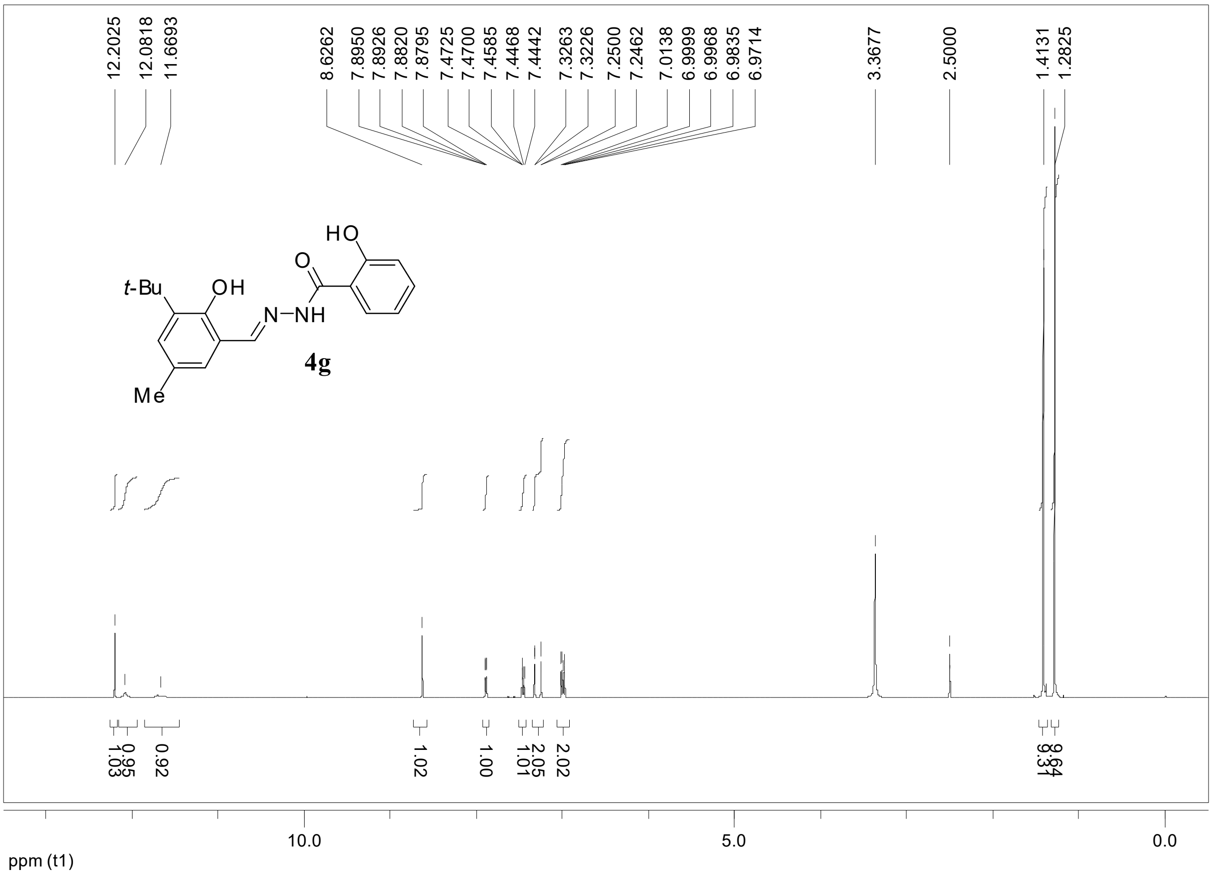

Figure S121. ^{1}H-NMR (600 MHz, DMSO-d_6) spectrum of compound **4g**

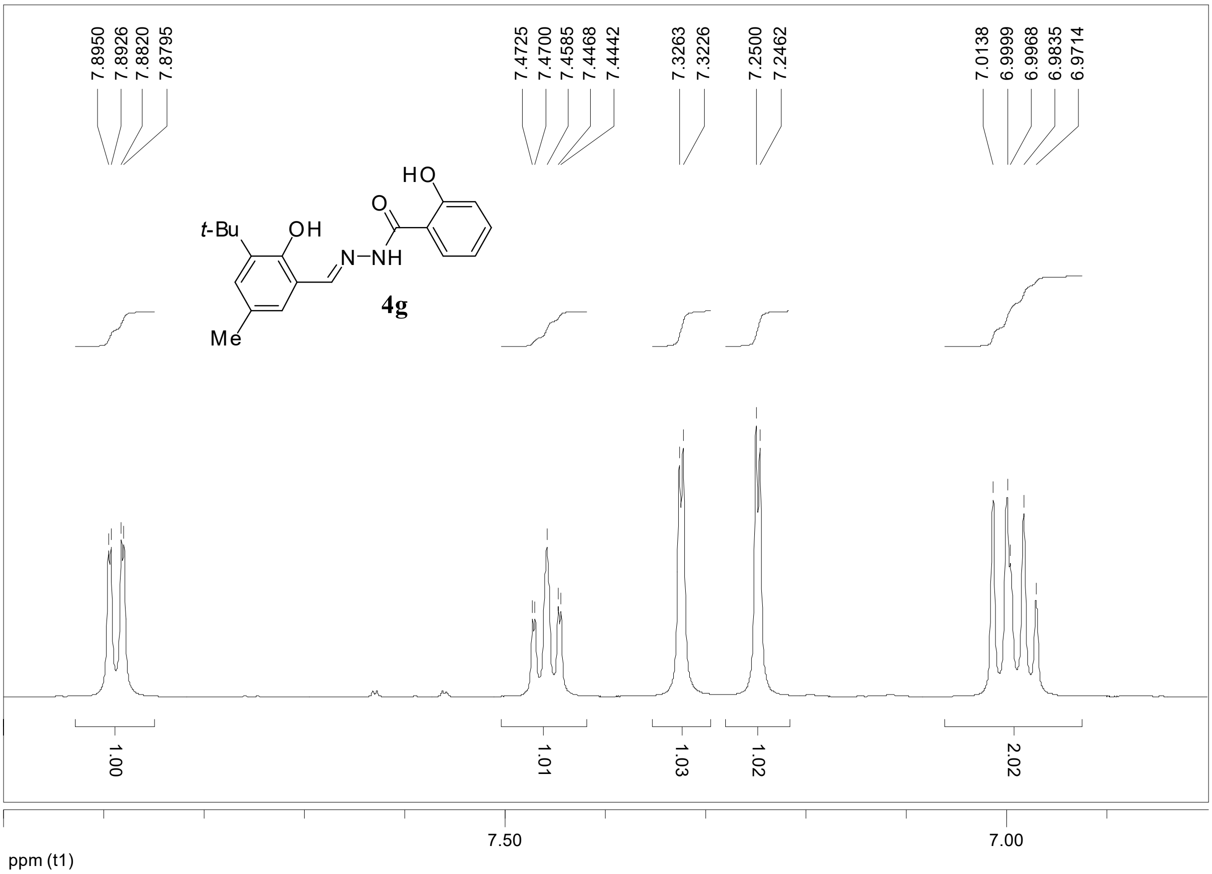

Figure S122. Expansion of ^{1}H-NMR (600 MHz, DMSO-d_6) spectrum of compound **4g**

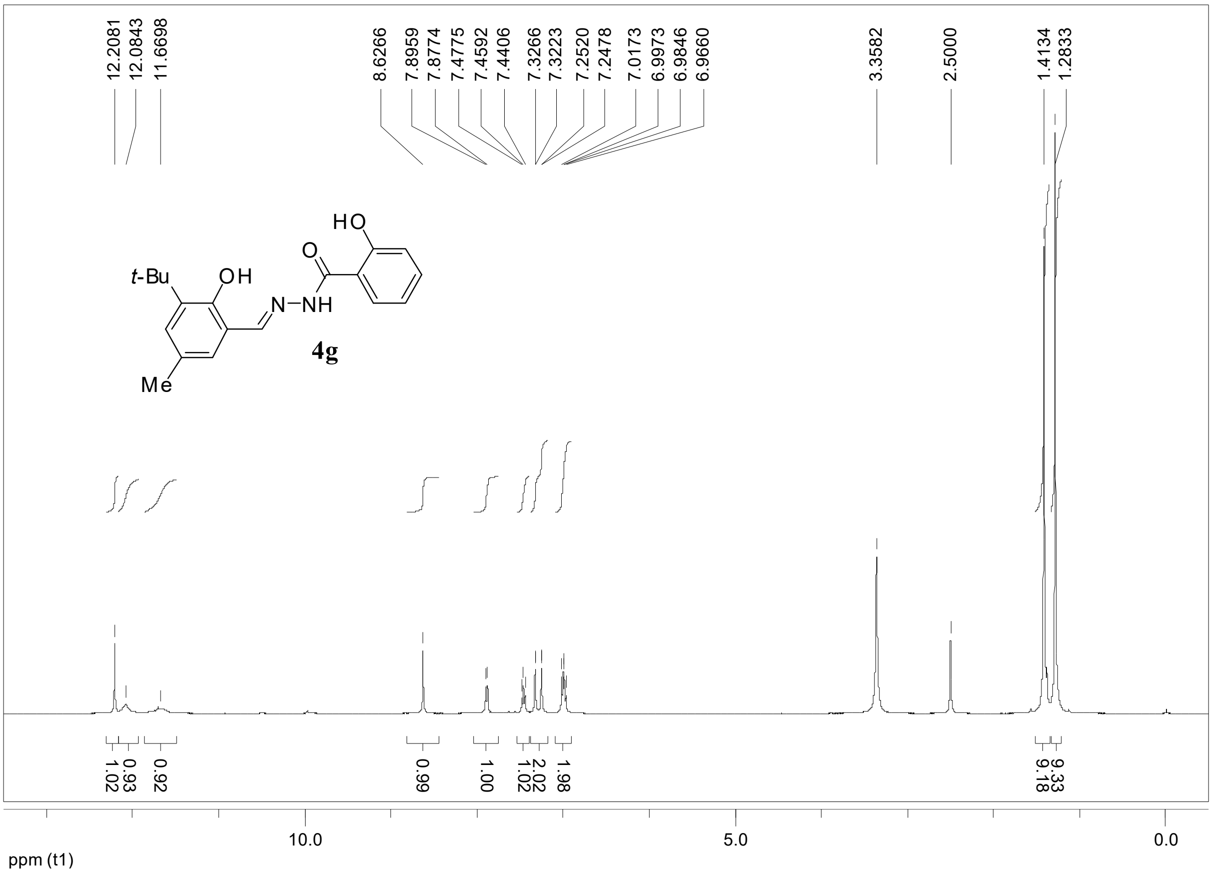

Figure S123. ^{1}H-NMR (400 MHz, DMSO-d_6) spectrum of compound **4g**

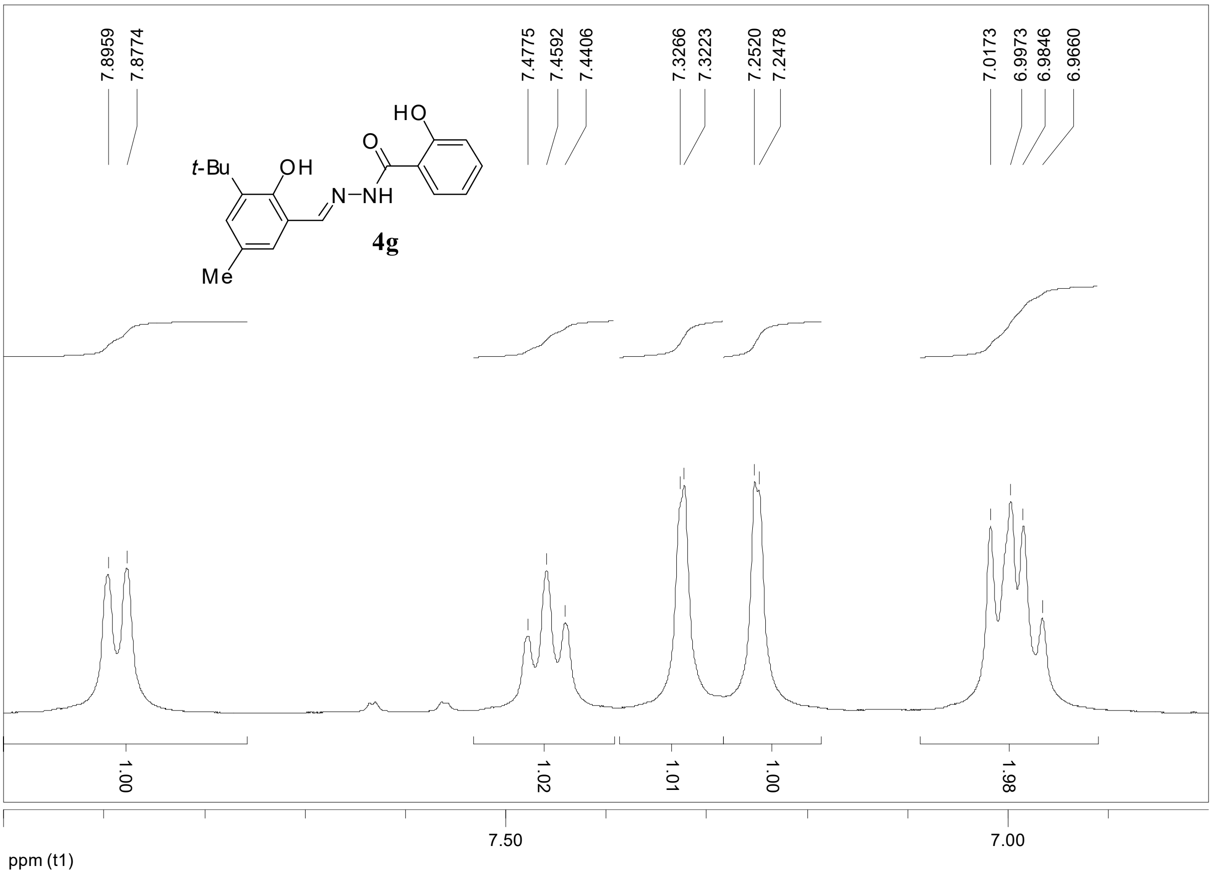

Figure S124. Expansion of ^{1}H-NMR (400 MHz, DMSO-d_6) spectrum of compound **4g**

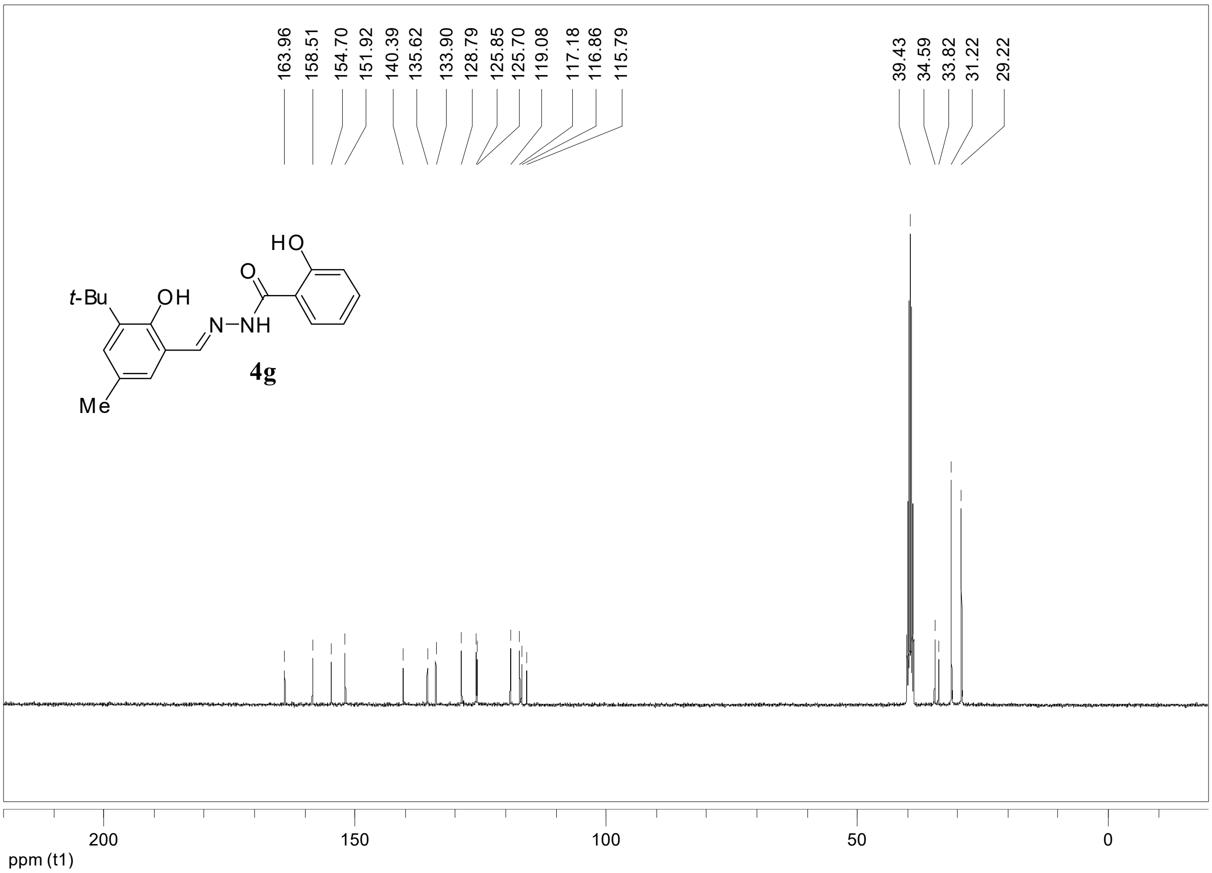

Figure S125. ^{13}C-NMR (100 MHz, DMSO-d_6) spectrum of compound **4g**

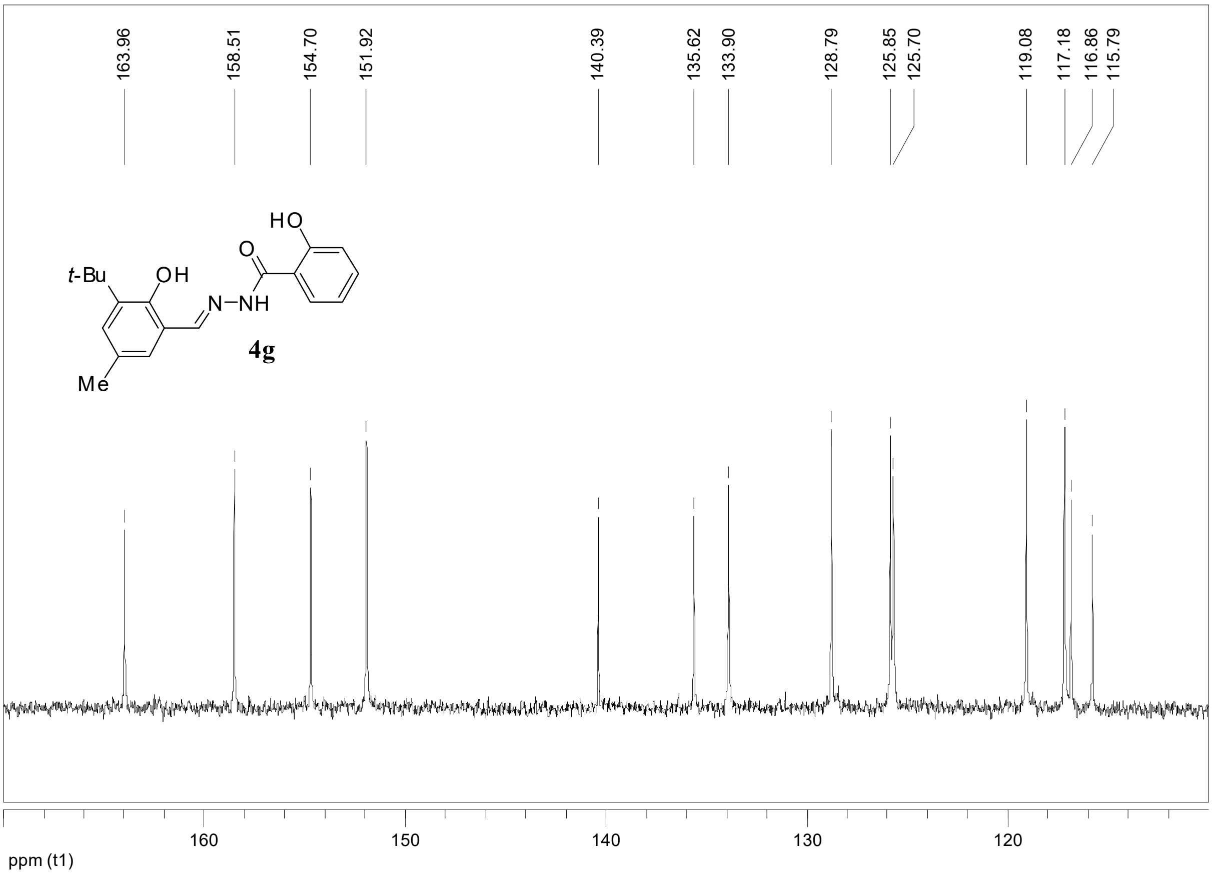

Figure S126. Expansion of ^{13}C-NMR (100 MHz, DMSO-d_6) spectrum of compound **4g**

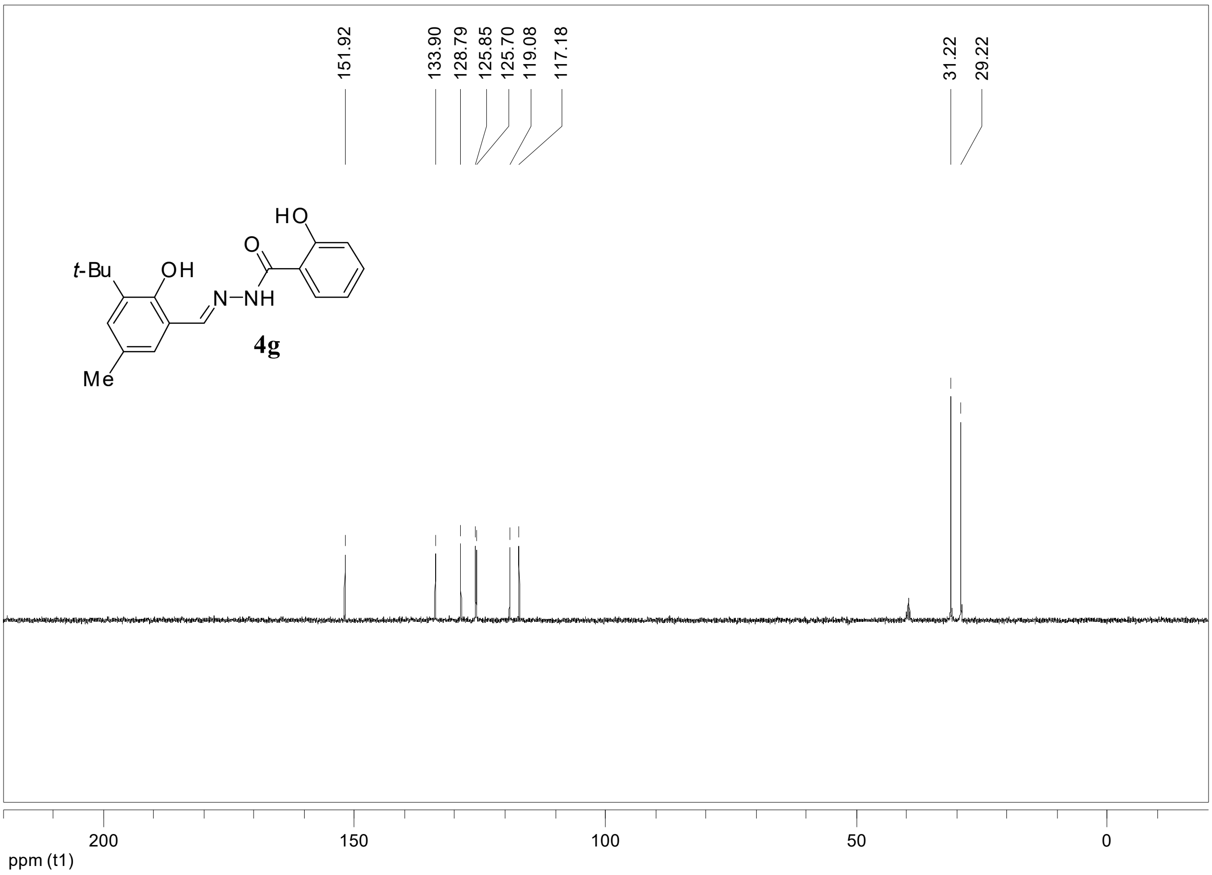

Figure S127. ^{13}C-NMR (100 MHz, DMSO-d_6) dept-135 experiment of compound **4g**

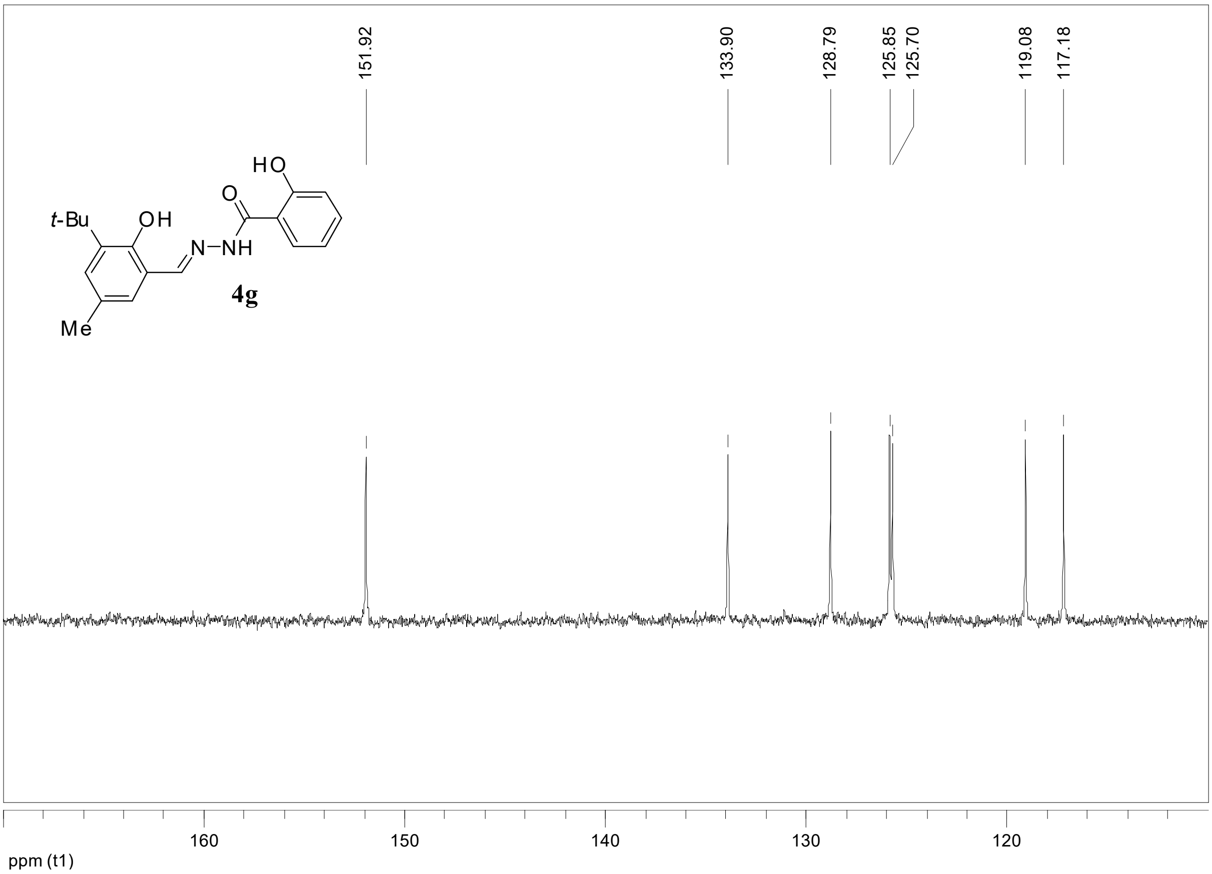

Figure S128. Expansion of ^{13}C-NMR (100 MHz, DMSO-d_6) dept-135 experiment of compound **4g**

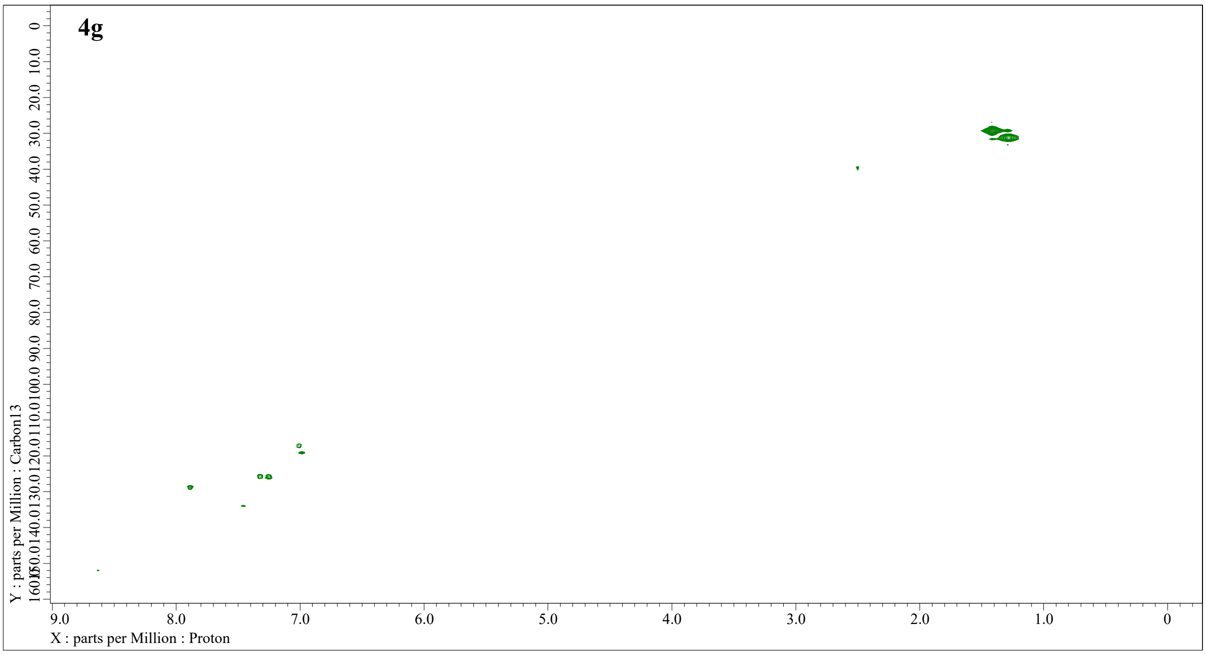

Figure S129. 2D-NMR (400 MHz, DMSO-d_6) HSQC experiment of 2-hydroxy-*N*'-[(*E*)-(3,5-di-*tert*-butyl-2-hydroxyphenyl)methylidene]benzohydrazide (**4g**)

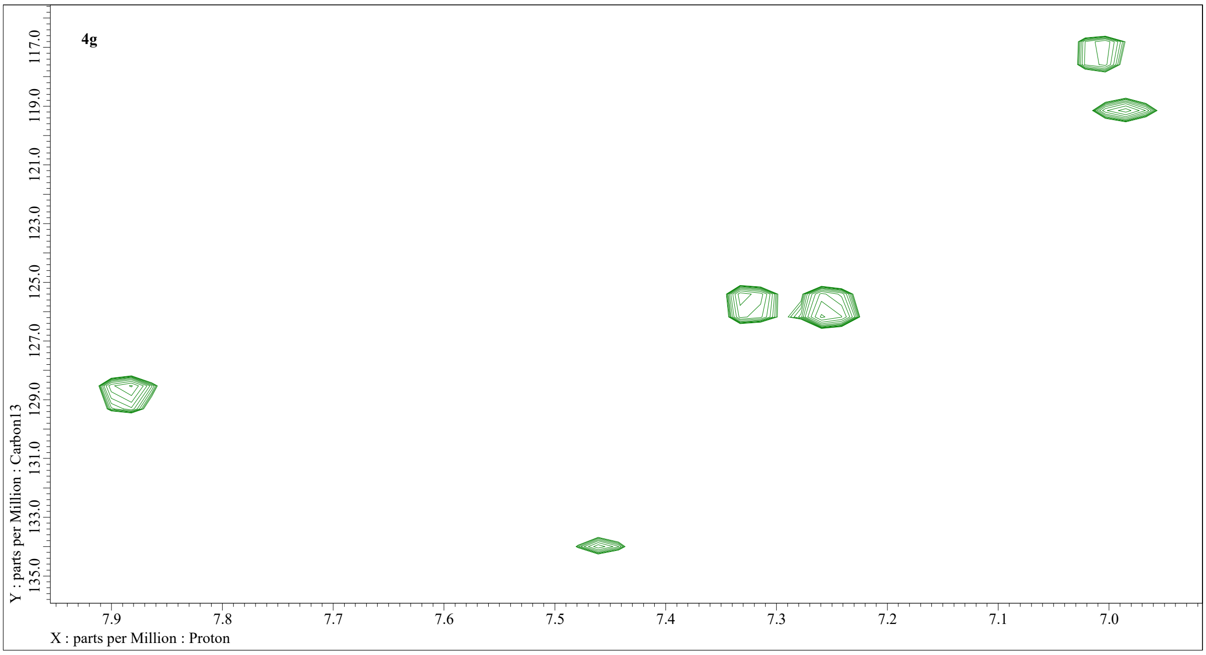

Figure S130. Expansion of 2D-NMR (400 MHz, DMSO-d_6) HSQC experiment of 2-hydroxy-*N*'-[(*E*)-(3,5-di-*tert*-butyl-2-hydroxyphenyl)methylidene]benzohydrazide (**4g**)

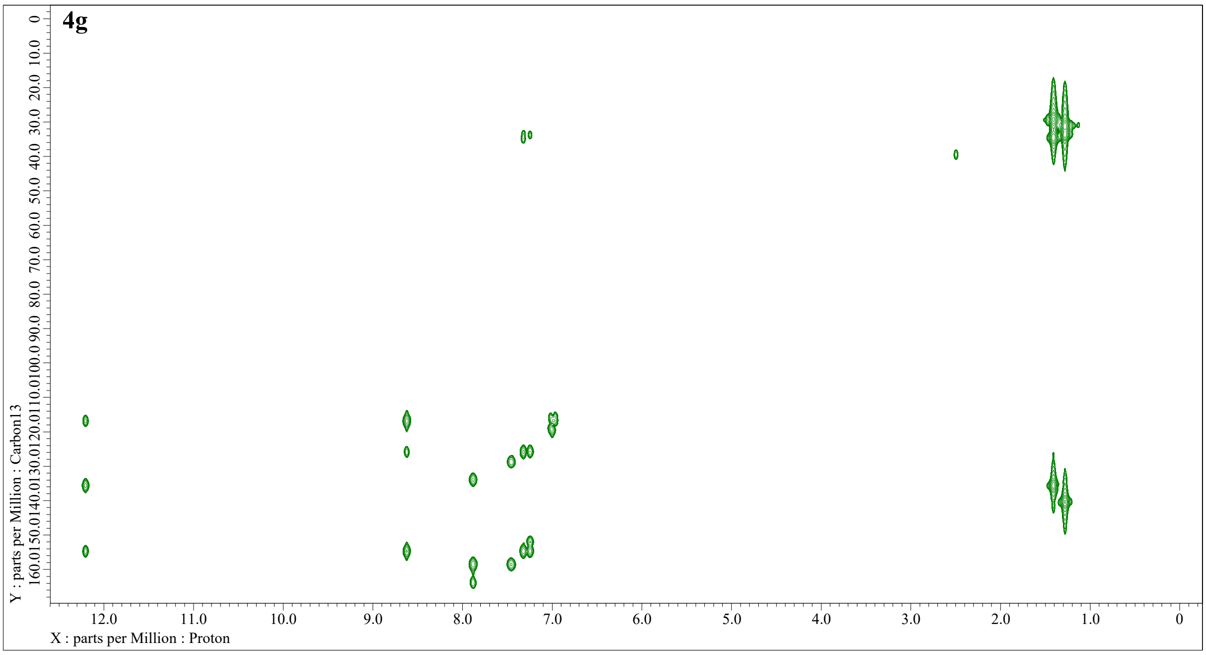

Figure S131. 2D-NMR (400 MHz, DMSO-d_6) HMBC experiment of 2-hydroxy-*N'*-[(*E*)-(3,5-di-*tert*-butyl-2-hydroxyphenyl)methylidene]benzohydrazide (**4g**)

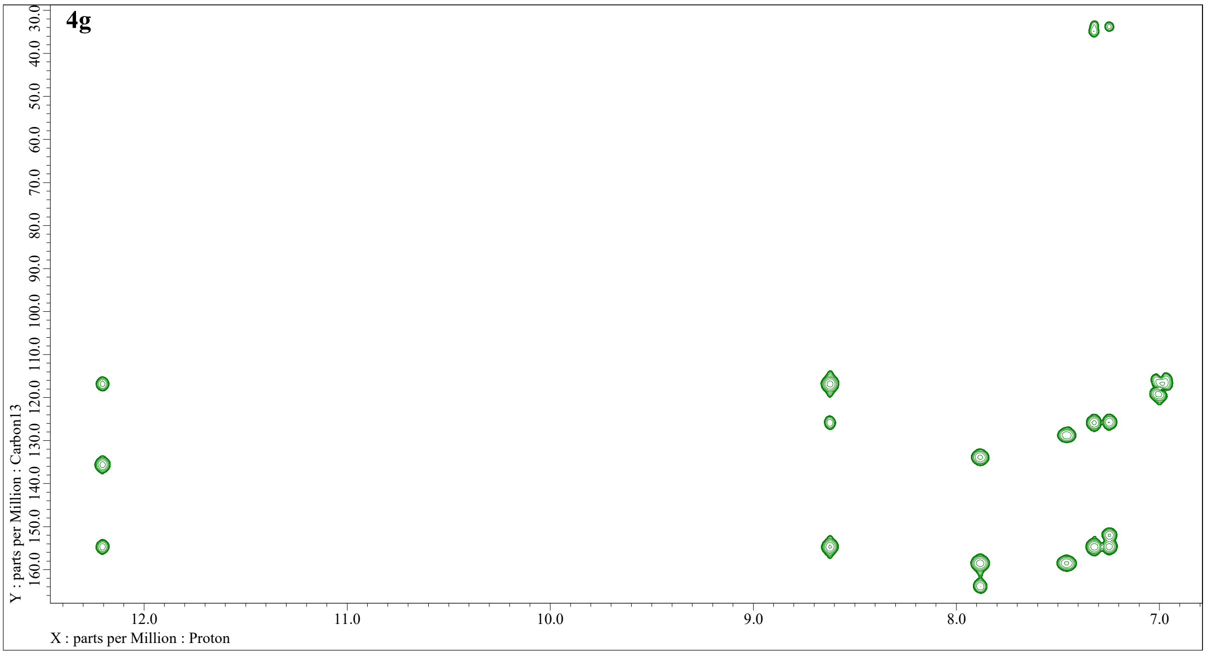

Figure S132. Expansion of 2D-NMR (400 MHz, DMSO-d_6) HMBC experiment of 2-hydroxy-*N'*-[(*E*)-(3,5-di-*tert*-butyl-2-hydroxyphenyl)methylidene]benzohydrazide (**4g**)

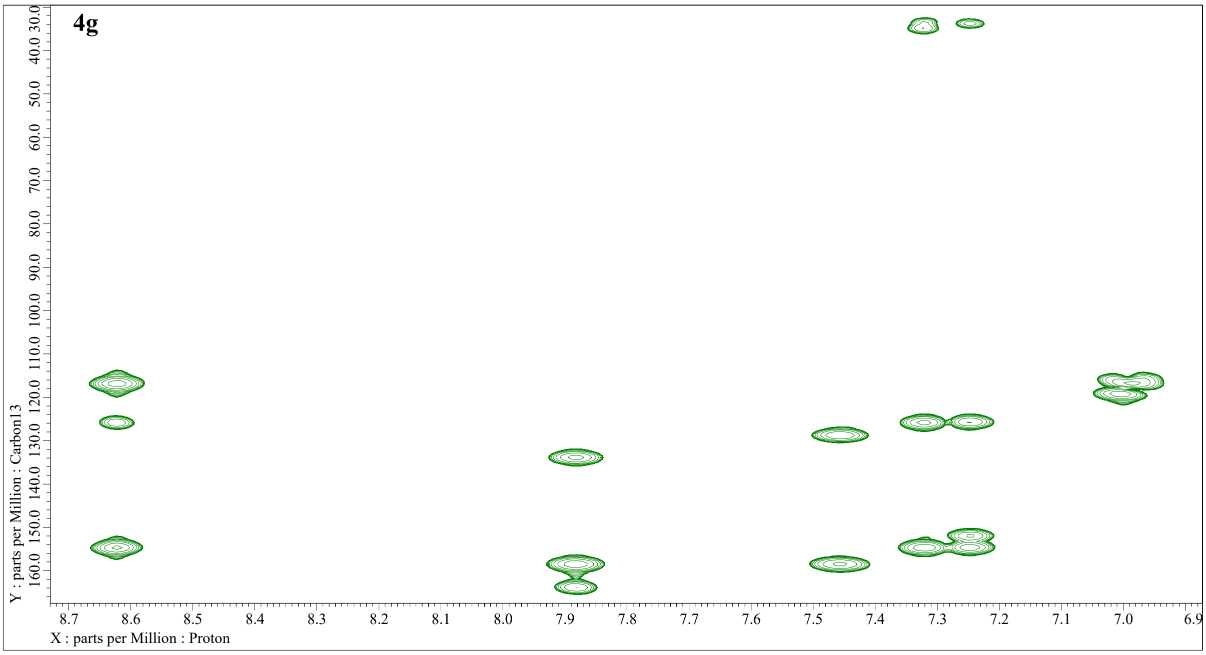

Figure S133. Expansion of 2D-NMR (400 MHz, DMSO-d_6) HMBC experiment of 2-hydroxy-*N*'-[(*E*)-(3,5-di-*tert*-butyl-2-hydroxyphenyl)methylidene]benzohydrazide (**4g**)

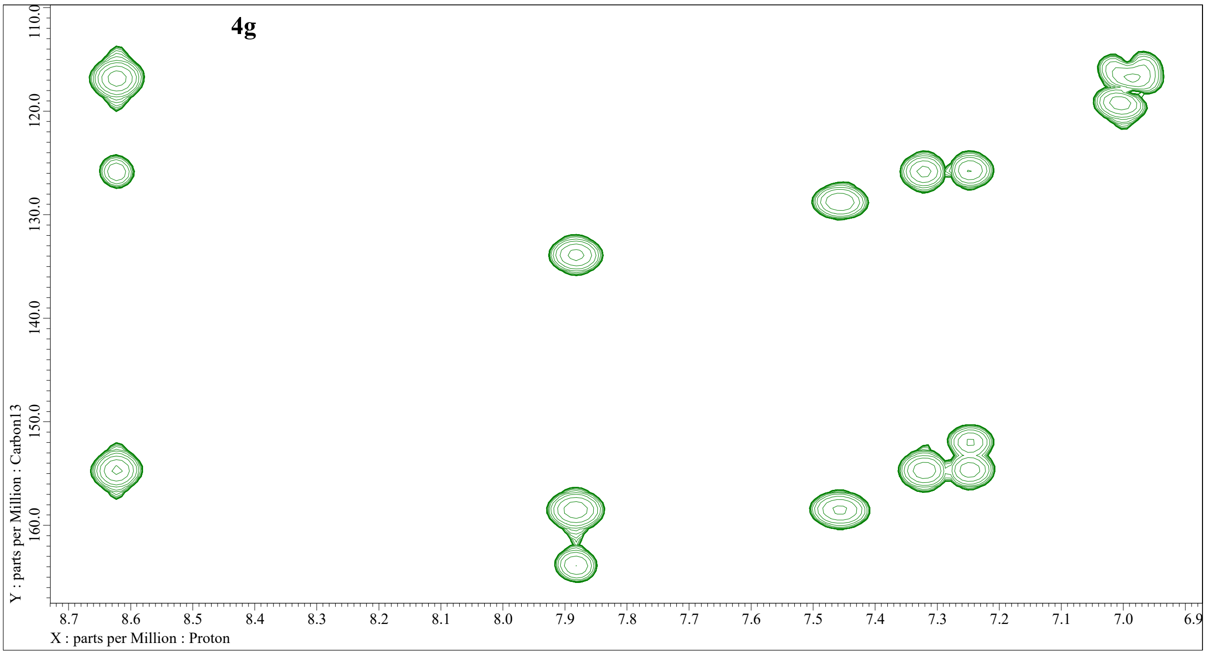

Figure S134. Expansion of 2D-NMR (400 MHz, DMSO-d_6) HMBC experiment of 2-hydroxy-*N*'-[(*E*)-(3,5-di-*tert*-butyl-2-hydroxyphenyl)methylidene]benzohydrazide (**4g**)

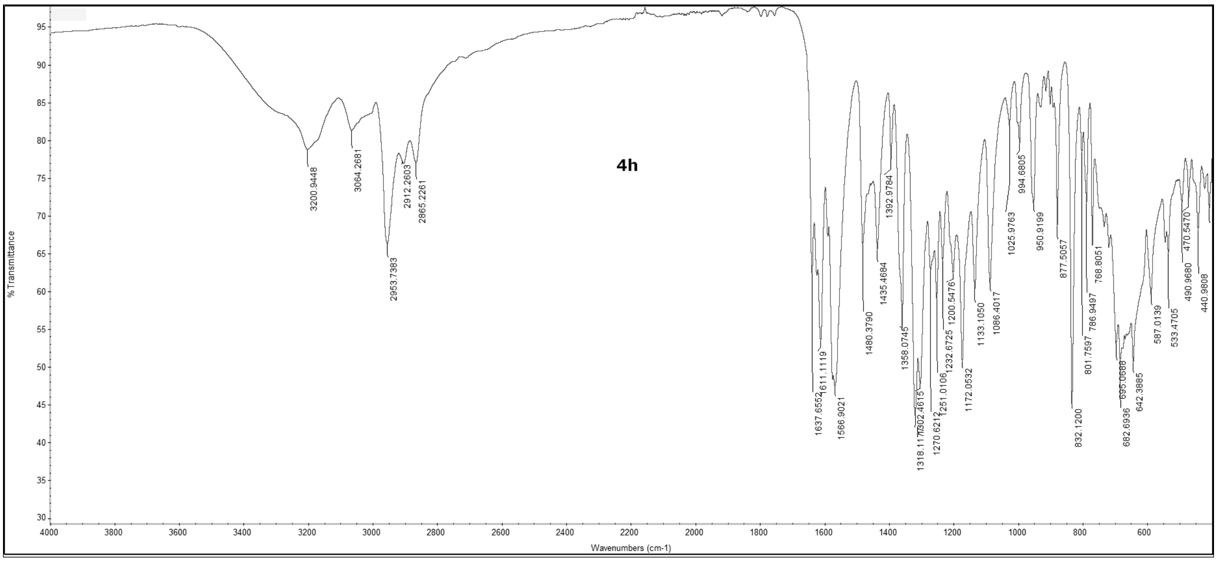

Figure S135. FT-IR (ATR) spectrum of 3-hydroxy-*N*'-[(*E*)-(3,5-di-*tert*-butyl-2-hydroxyphenyl)methylidene]benzohydrazide (**4h**)

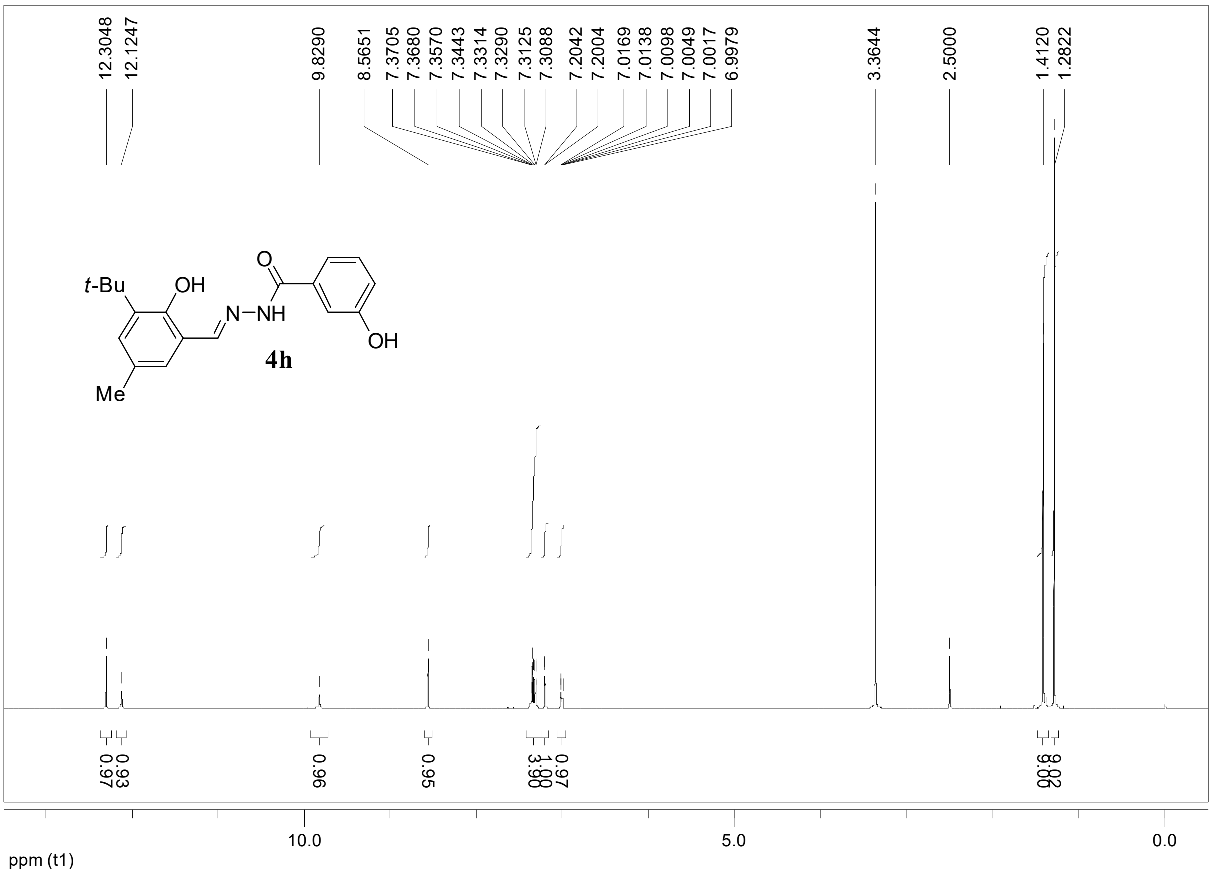

Figure S136. ^{1}H-NMR (600 MHz, DMSO-d_6) spectrum of compound **4h**

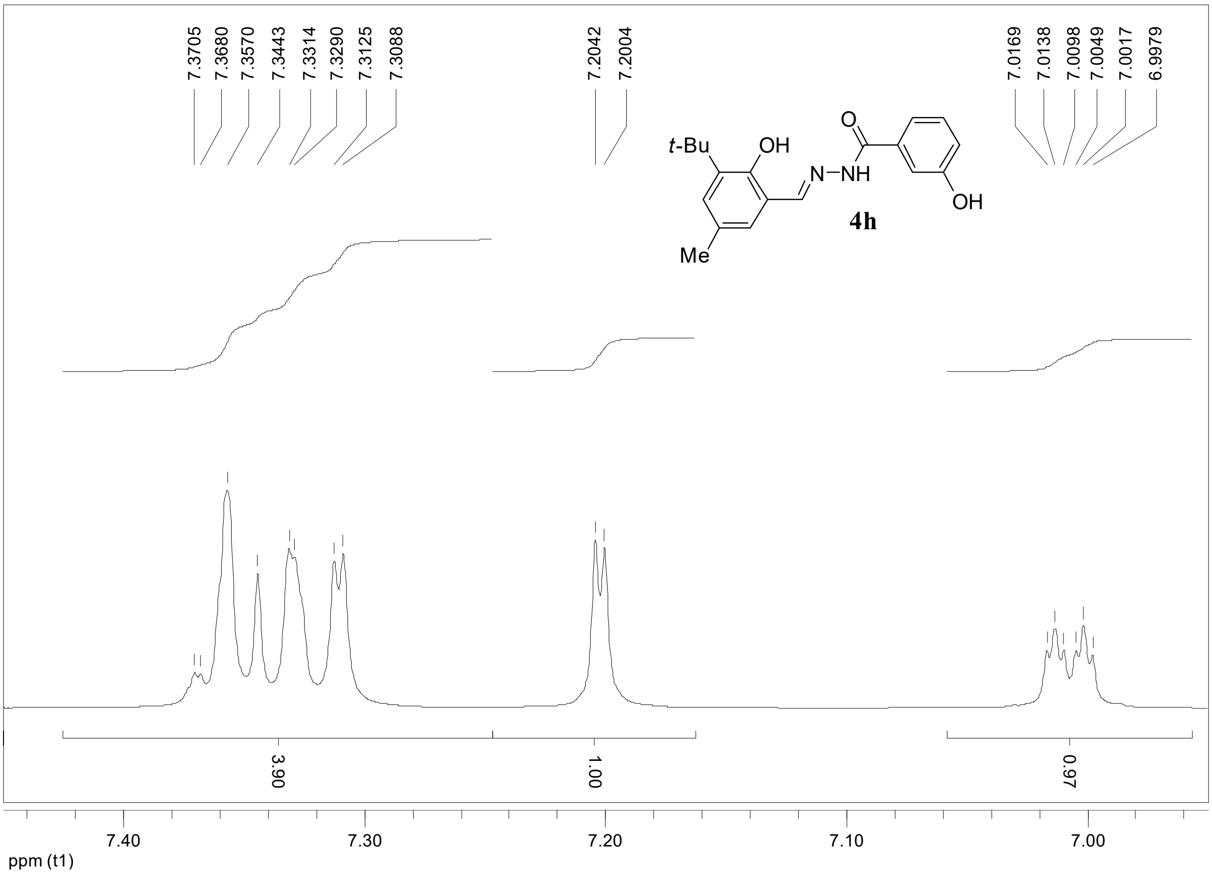

Figure S137. Expansion of ^{1}H-NMR (600 MHz, DMSO-d_6) spectrum of compound **4h**

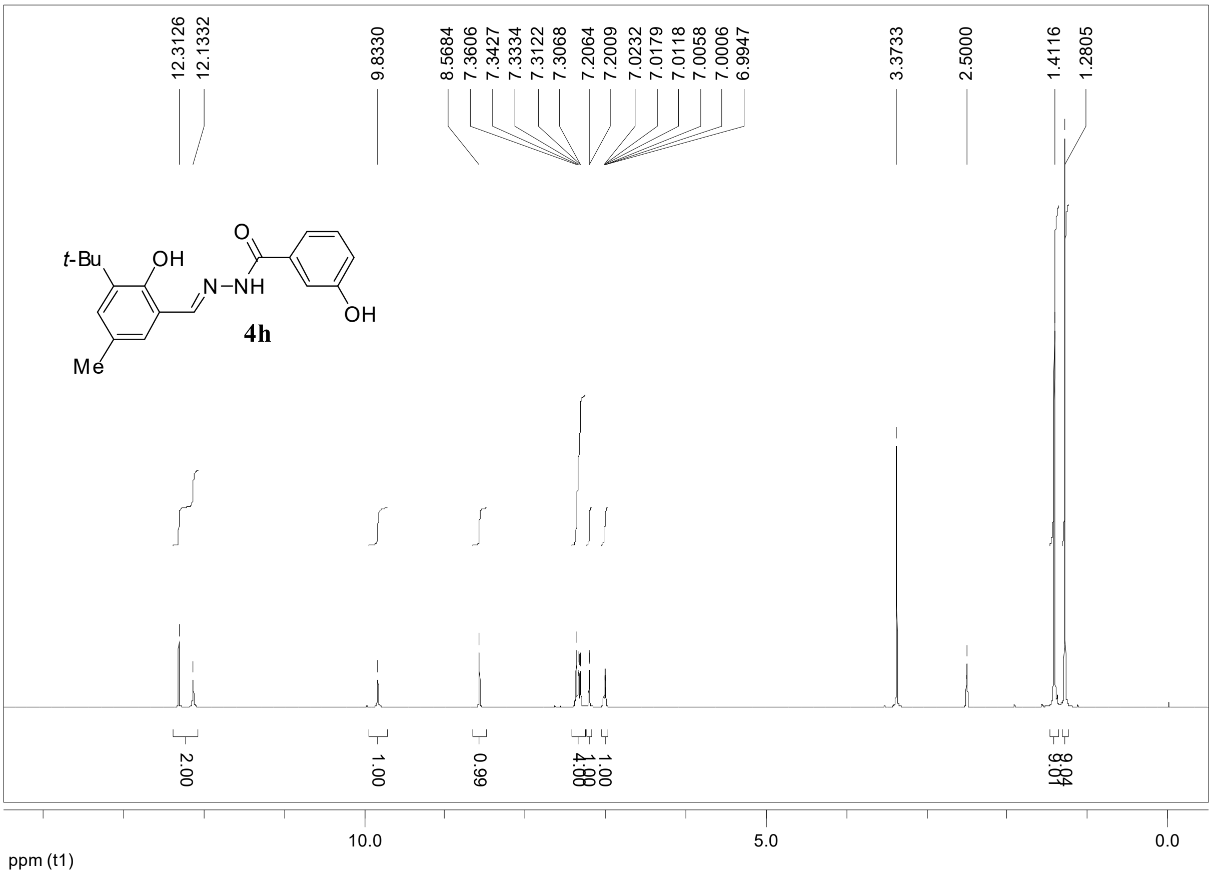

Figure S138. ^{1}H-NMR (400 MHz, DMSO-d_6) spectrum of compound **4h**

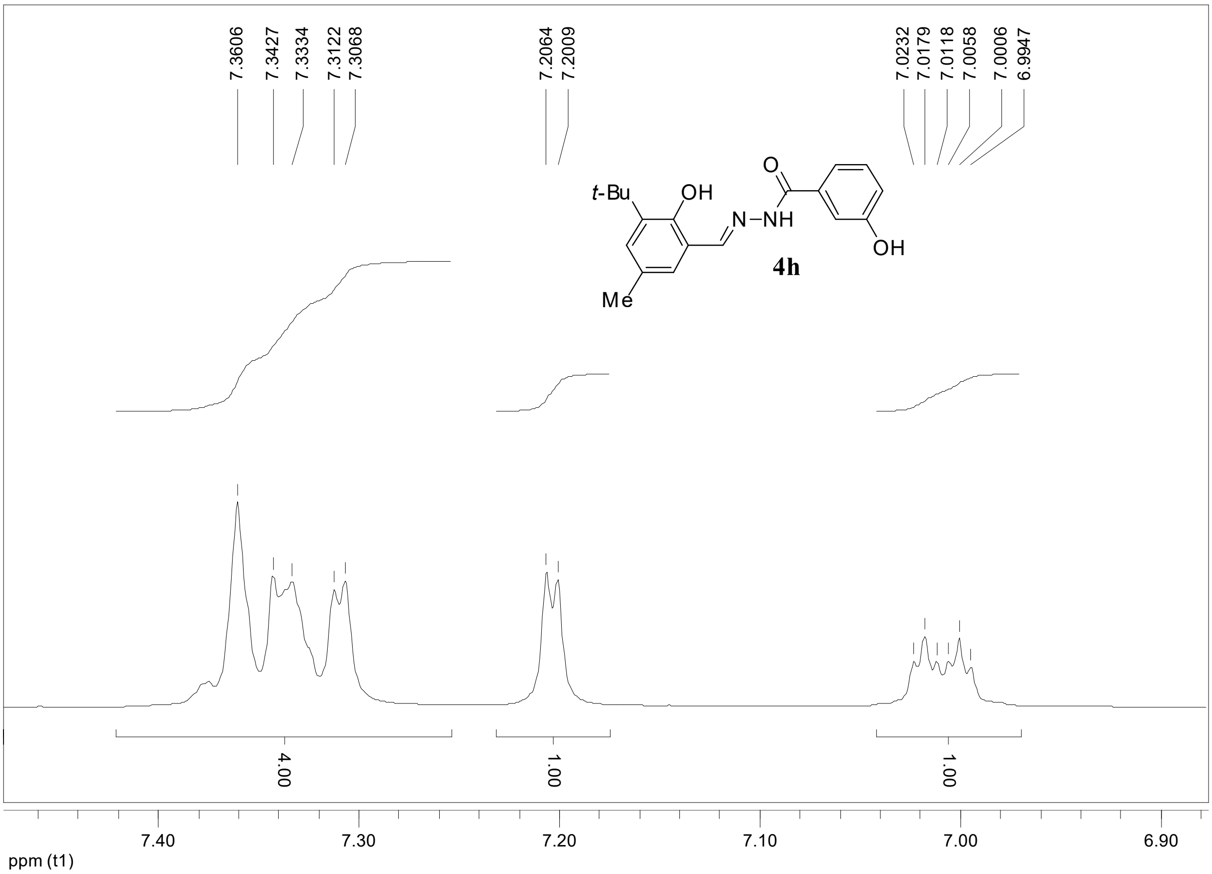

Figure S139. Expansion of ^{1}H-NMR (400 MHz, DMSO-d_6) spectrum of compound **4h**

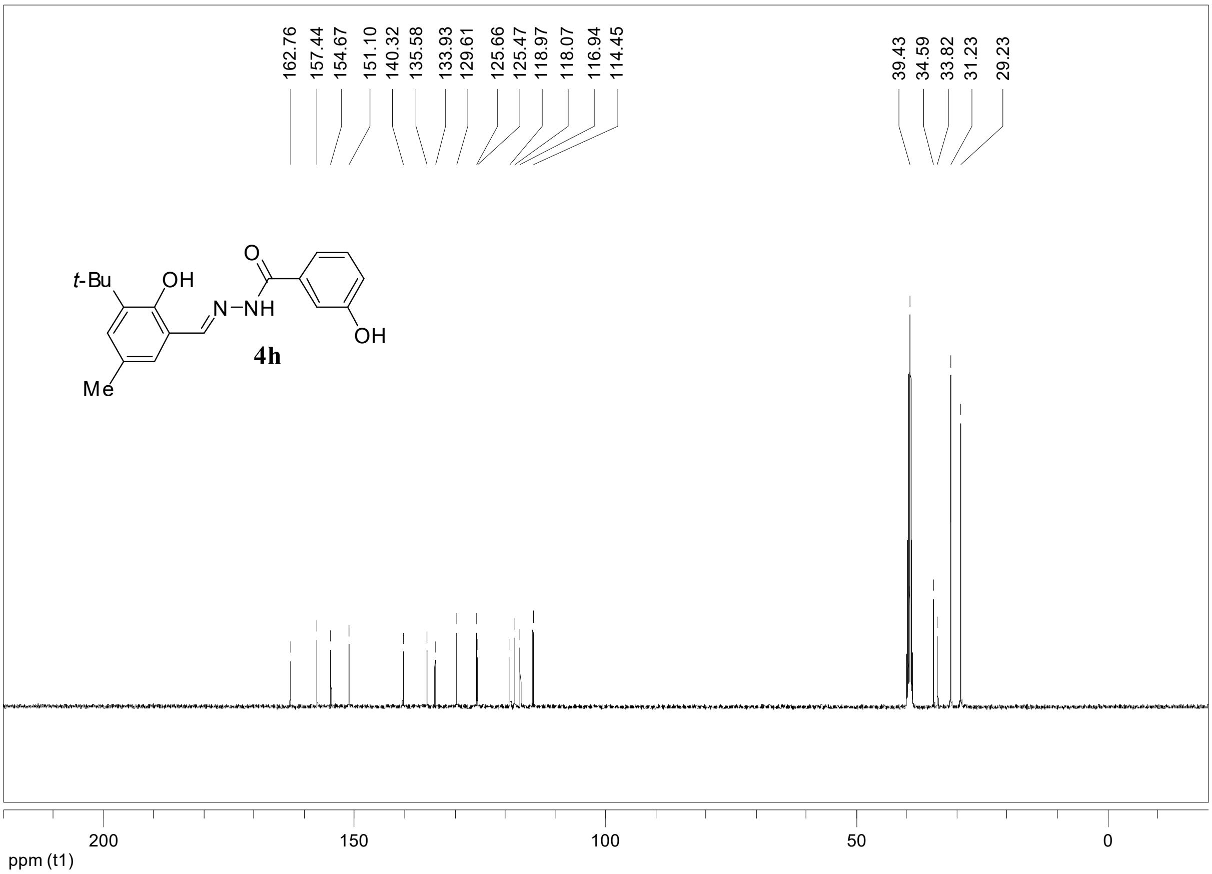

Figure S140. ^{13}C-NMR (100 MHz, DMSO-d_6) spectrum of compound **4h**

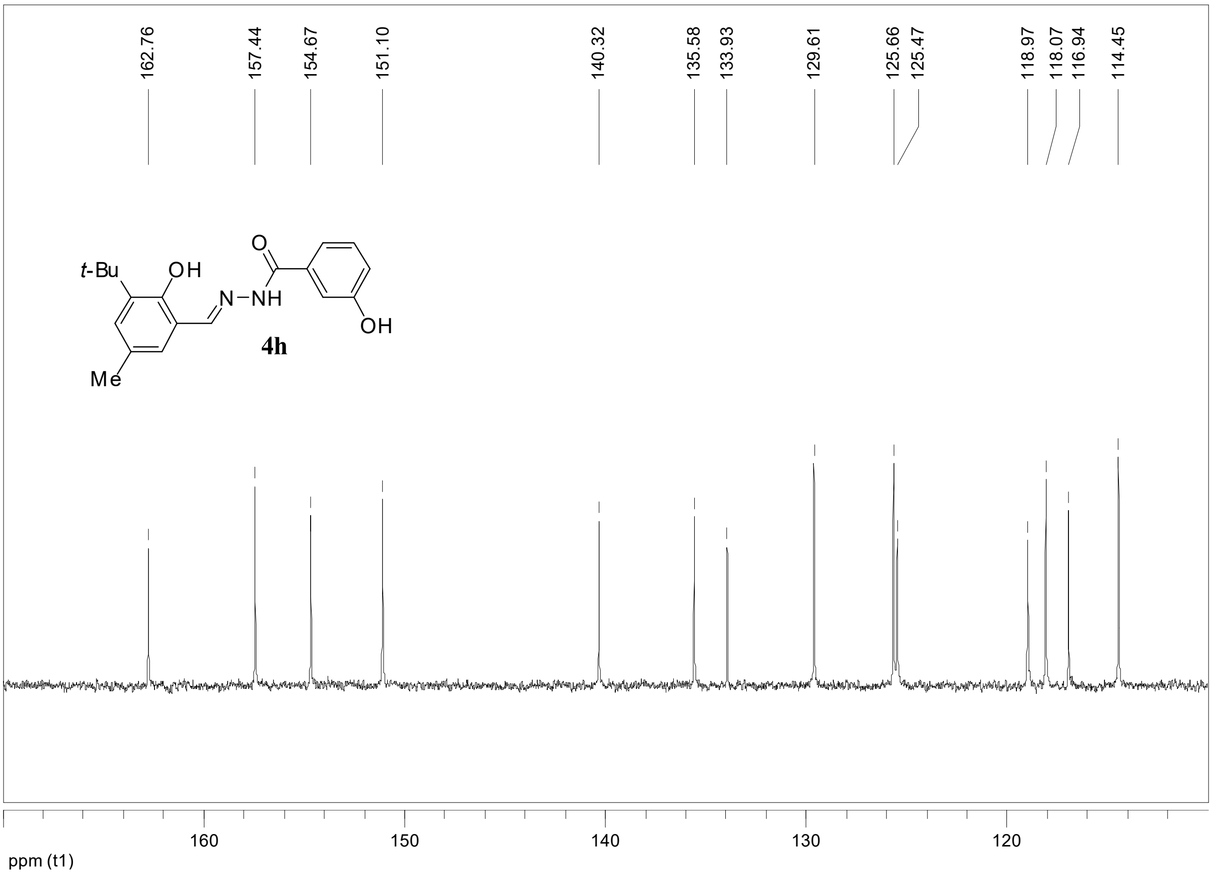

Figure S141. Expansion of ^{13}C-NMR (100 MHz, DMSO-d_6) spectrum of compound **4h**

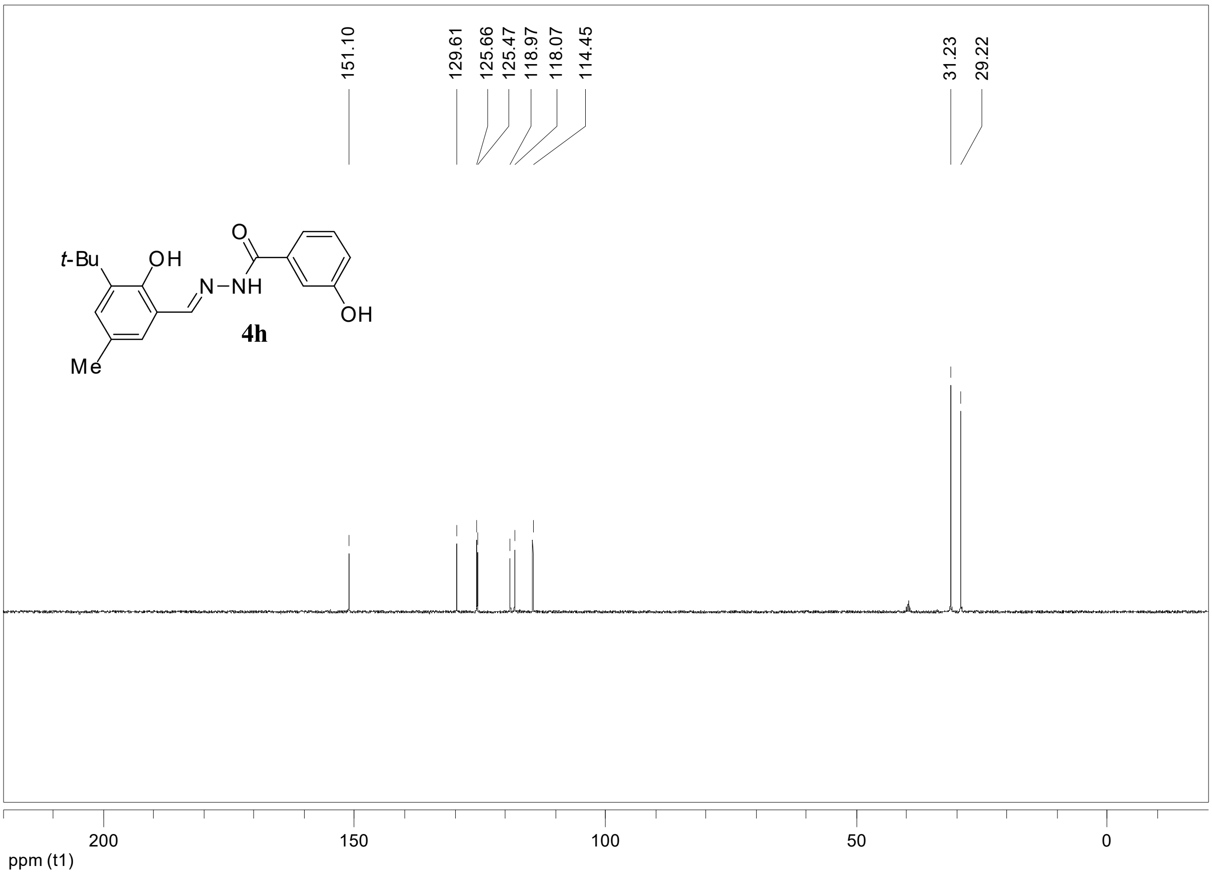

Figure S142. ^{13}C-NMR (100 MHz, DMSO-d_6) dept-135 experiment of compound **4h**

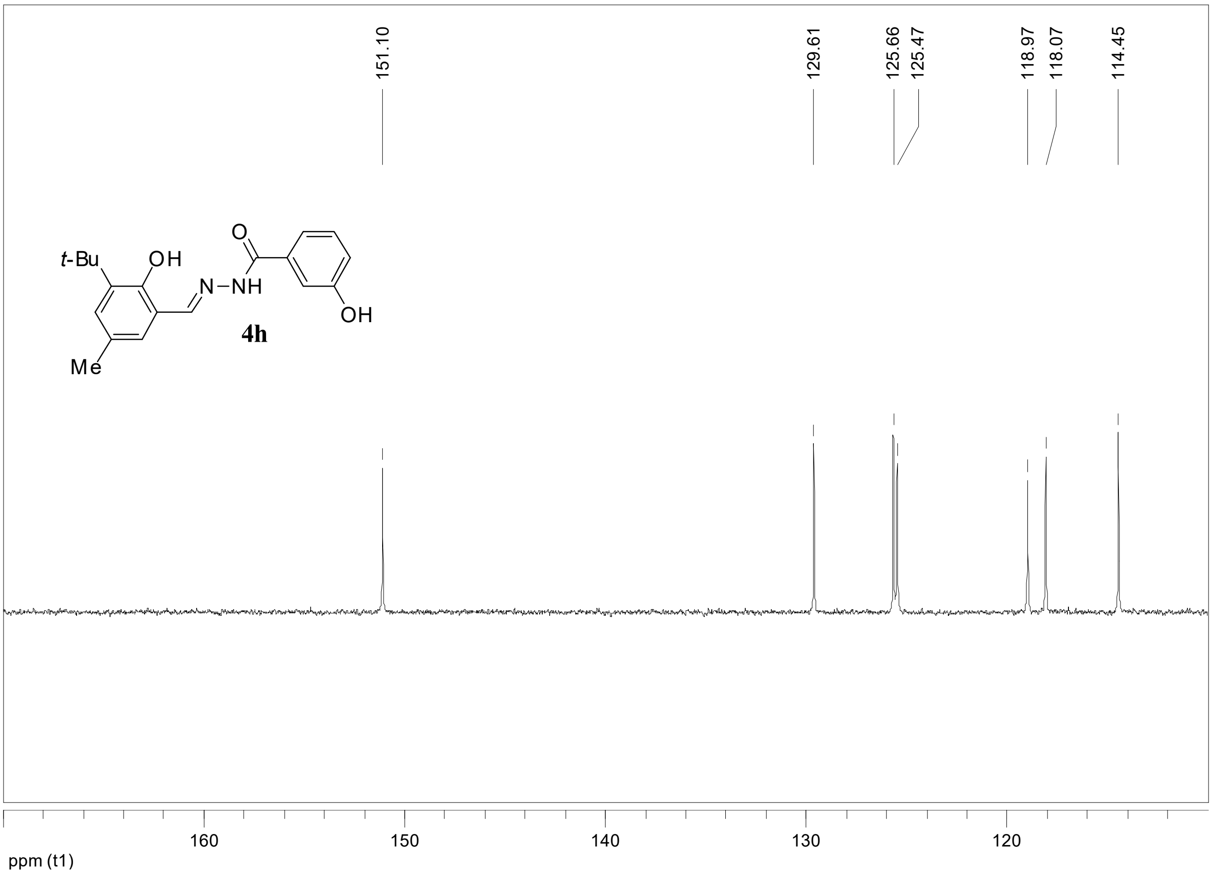

Figure S143. Expansion of ^{13}C-NMR (100 MHz, DMSO-d_6) dept-135 experiment of compound **4h**

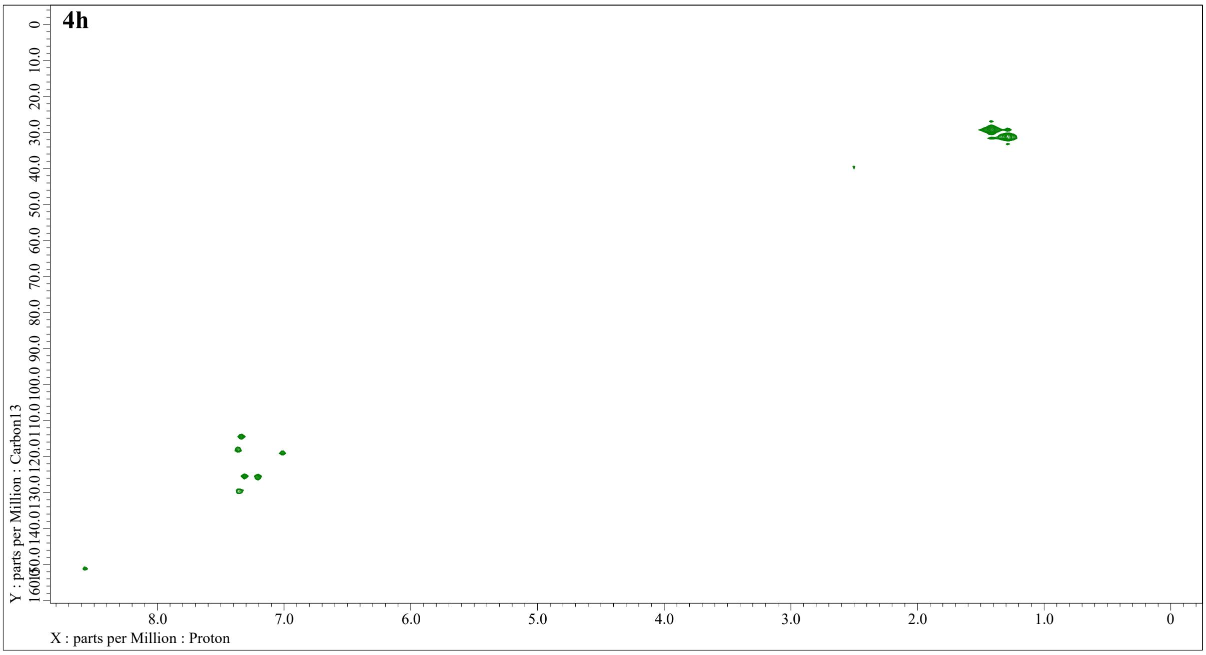

Figure S144. 2D-NMR (400 MHz, DMSO-d_6) HSQC experiment of 3-hydroxy-*N*'-[(*E*)-(3,5-di-*tert*-butyl-2-hydroxyphenyl)methylidene]benzohydrazide (**4h**)

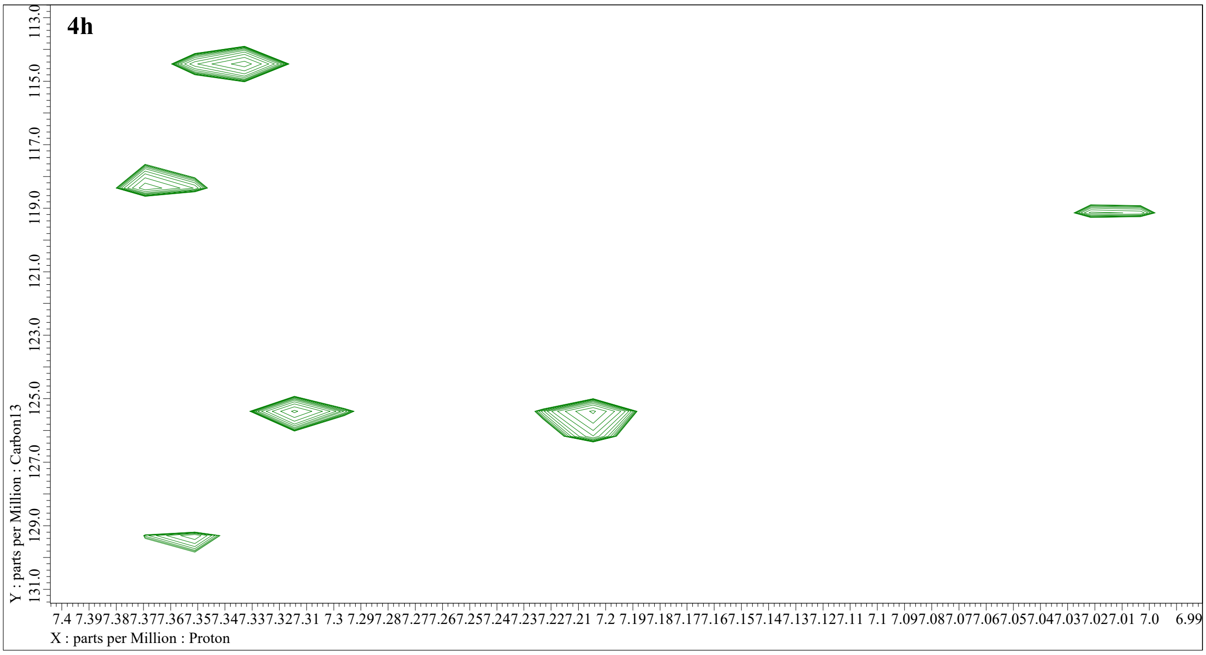

Figure S145. Expansion of 2D-NMR (400 MHz, DMSO-d_6) HSQC experiment of 3-hydroxy-*N*'-[(*E*)-(3,5-di-*tert*-butyl-2-hydroxyphenyl)methylidene]benzohydrazide (**4h**)

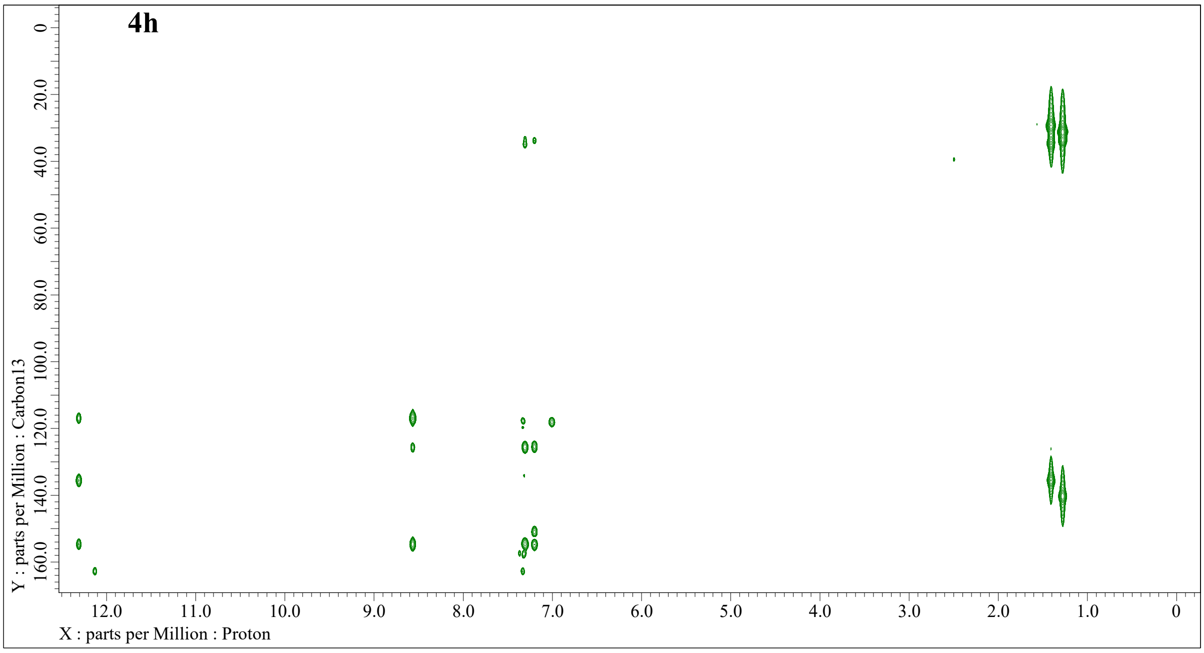

Figure S146. 2D-NMR (400 MHz, DMSO-d_6) HMBC experiment of 3-hydroxy-*N*'-[(*E*)-(3,5-di-*tert*-butyl-2-hydroxyphenyl)methylidene]benzohydrazide (**4h**)

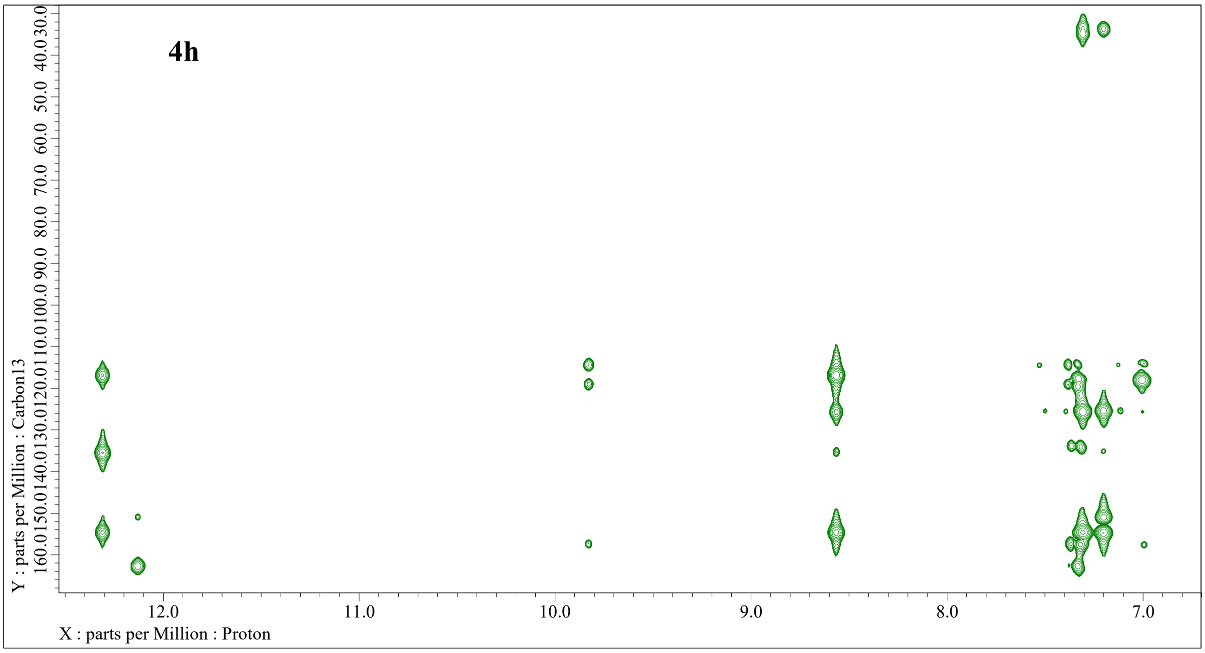

Figure S147. Expansion of 2D-NMR (400 MHz, DMSO-d_6) HMBC experiment of 3-hydroxy-*N*'-[(*E*)-(3,5-di-*tert*-butyl-2-hydroxyphenyl)methylidene]benzohydrazide (**4h**)

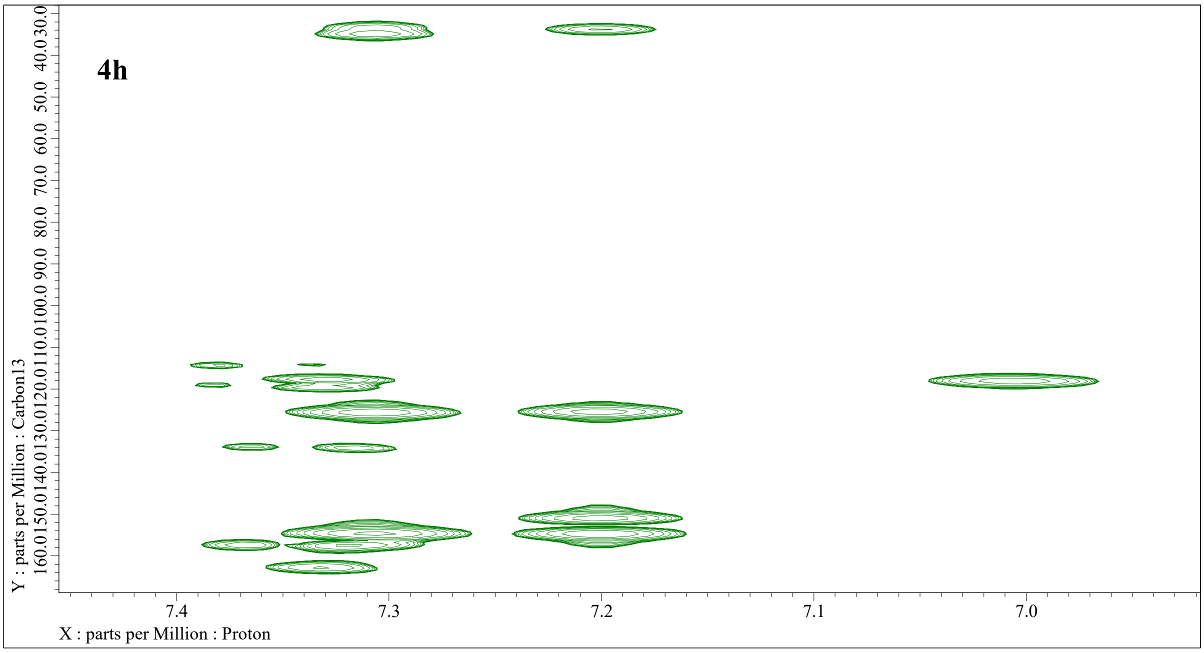

Figure S148. Expansion of 2D-NMR (400 MHz, DMSO-d_6) HMBC experiment of 3-hydroxy-*N'*-[(*E*)-(3,5-di-*tert*-butyl-2-hydroxyphenyl)methylidene]benzohydrazide (**4h**)

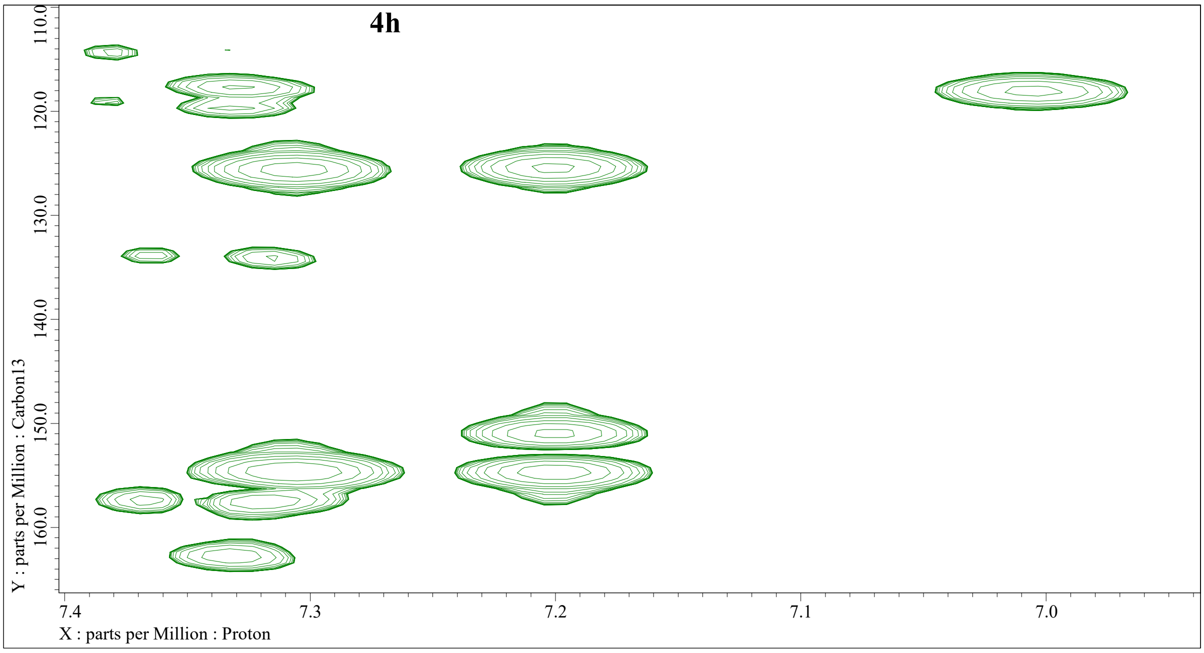

Figure S149. Expansion of 2D-NMR (400 MHz, DMSO-d_6) HMBC experiment of 3-hydroxy-*N'*-[(*E*)-(3,5-di-*tert*-butyl-2-hydroxyphenyl)methylidene]benzohydrazide (**4h**)

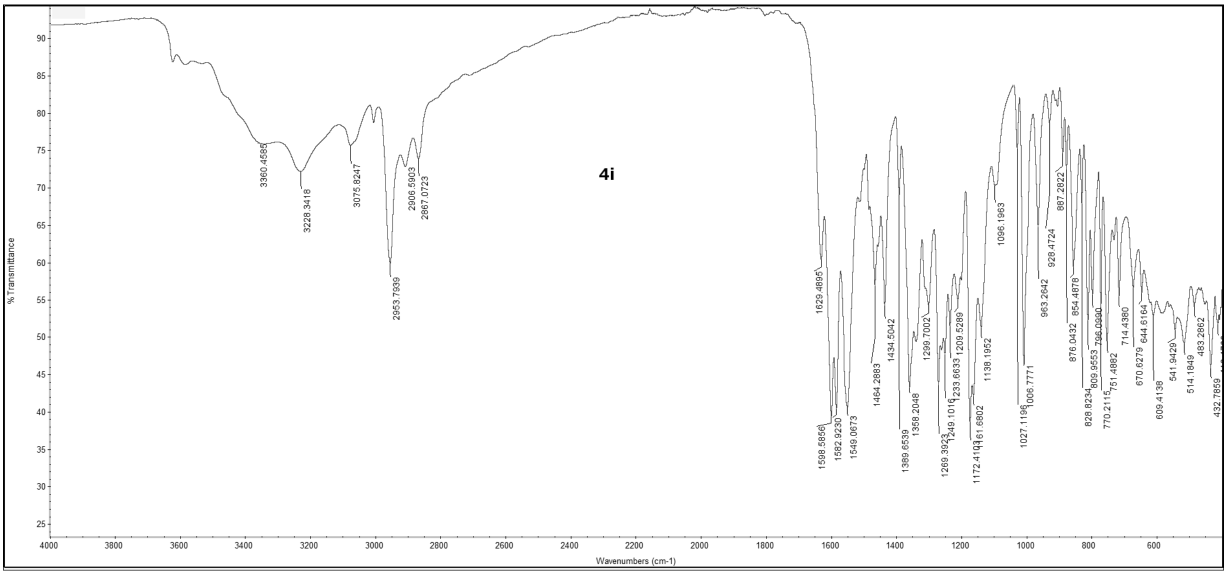

Figure S150. FT-IR (ATR) spectrum of 3,5-dihydroxy-*N*'-[(*E*)-(3,5-di-*tert*-butyl-2-hydroxyphenyl)methylidene]benzohydrazide (**4i**)

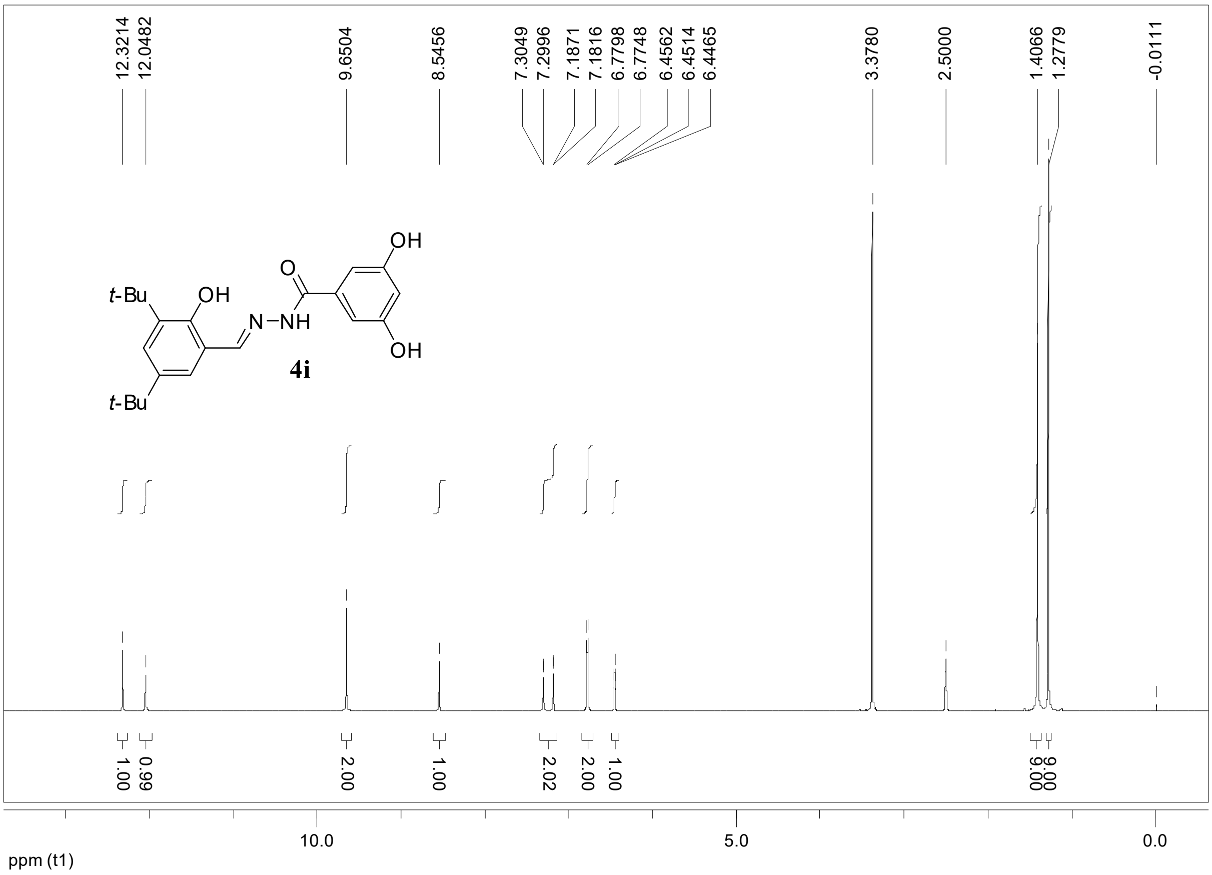

Figure S151. ^{1}H-NMR (400 MHz, DMSO-d_6) spectrum of compound **4i**

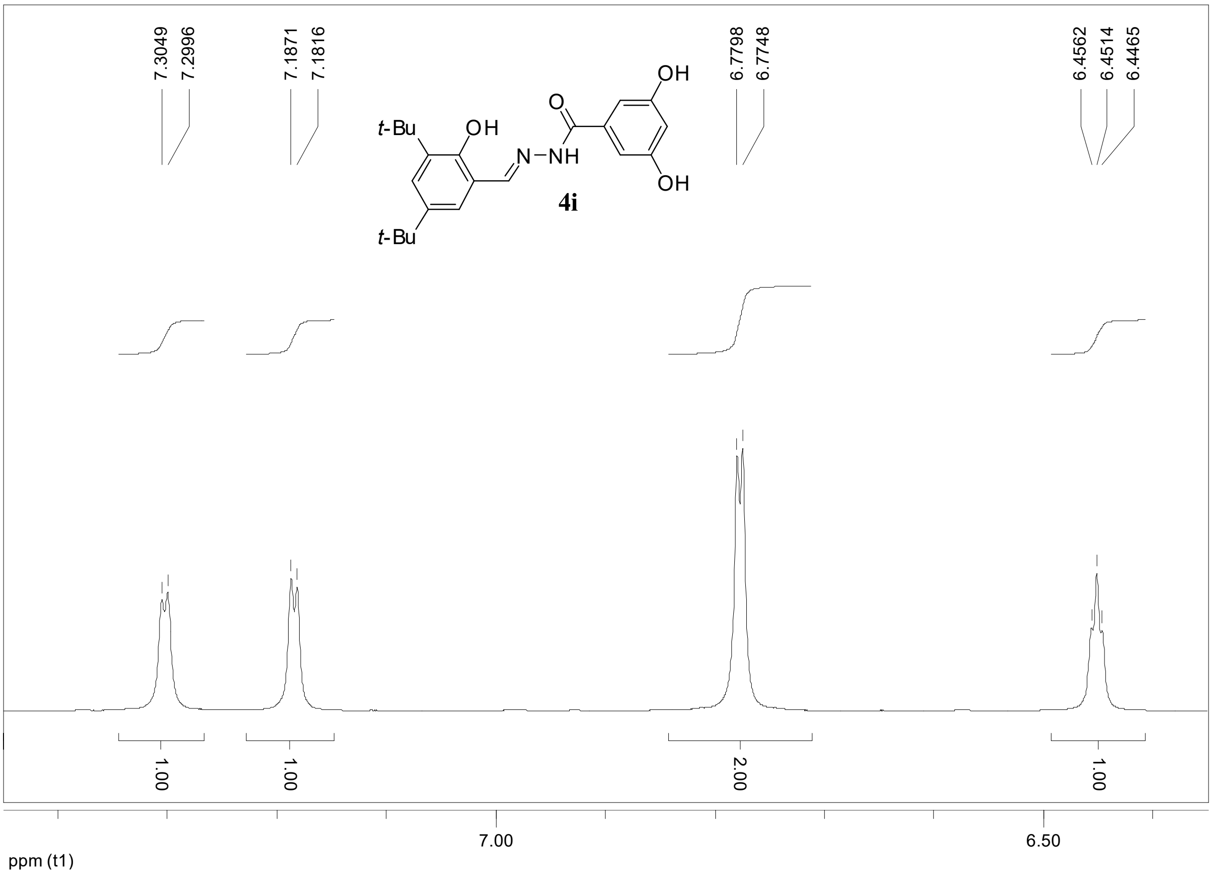

Figure S152. Expansion of ^{1}H-NMR (400 MHz, DMSO-d_6) spectrum of compound **4i**

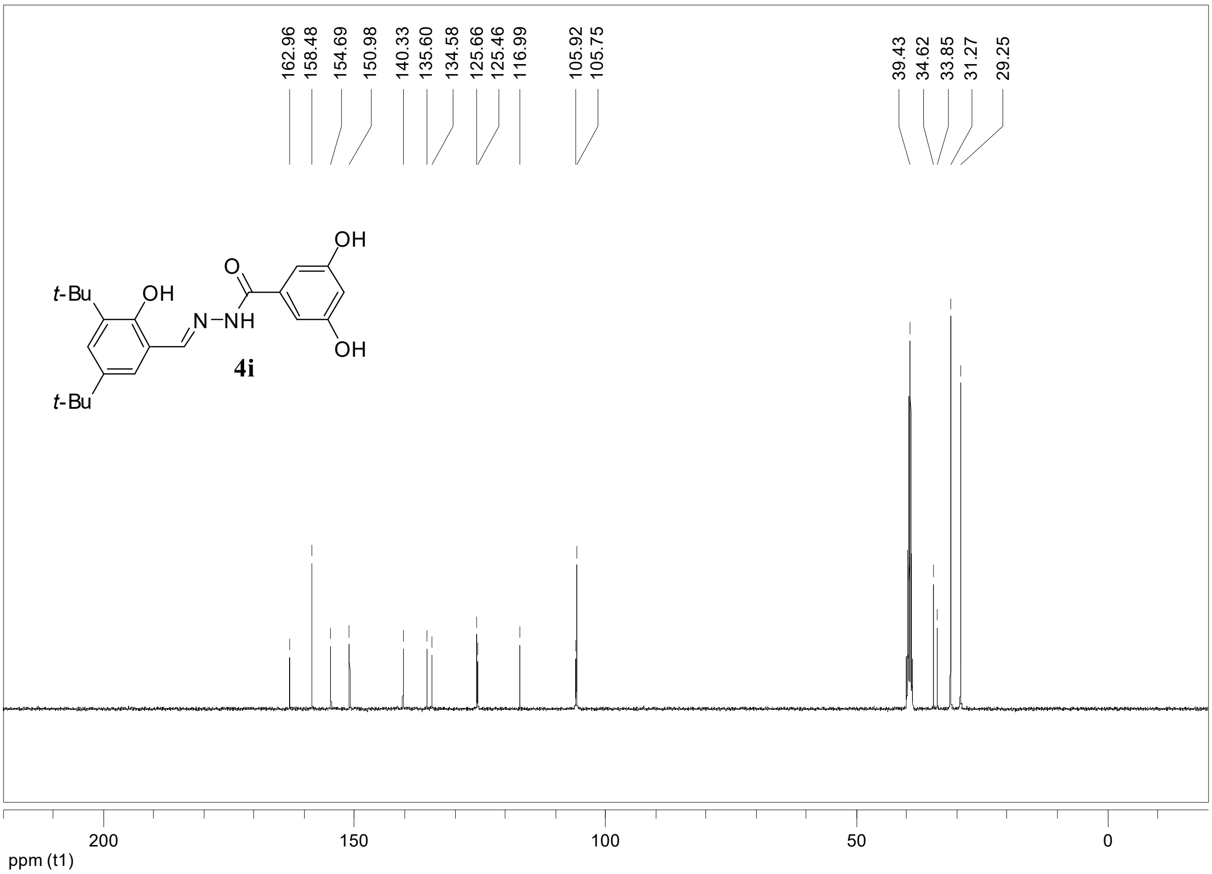

Figure S153. ^{13}C-NMR (100 MHz, DMSO-d_6) spectrum of compound **4i**

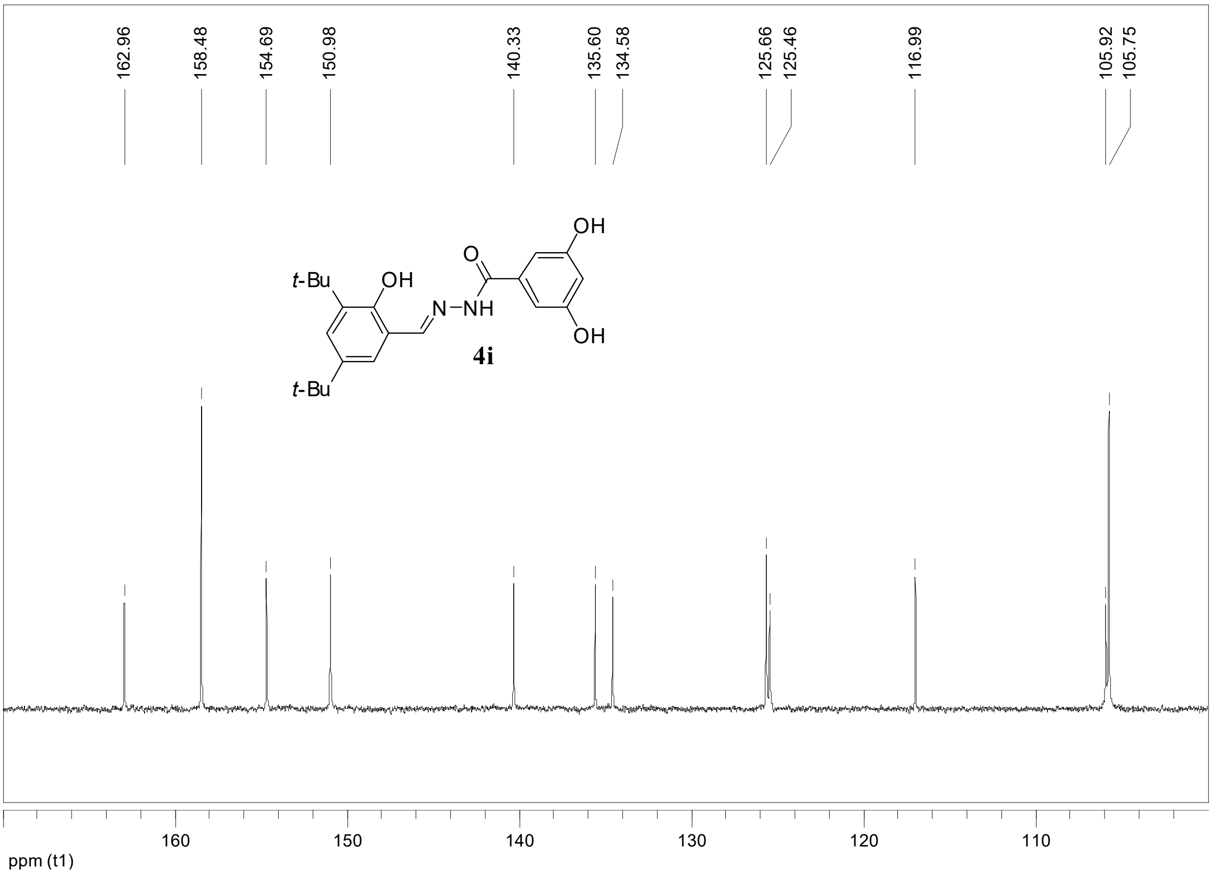

Figure S154. Expansion of ^{13}C-NMR (100 MHz, DMSO-d_6) spectrum of compound **4i**

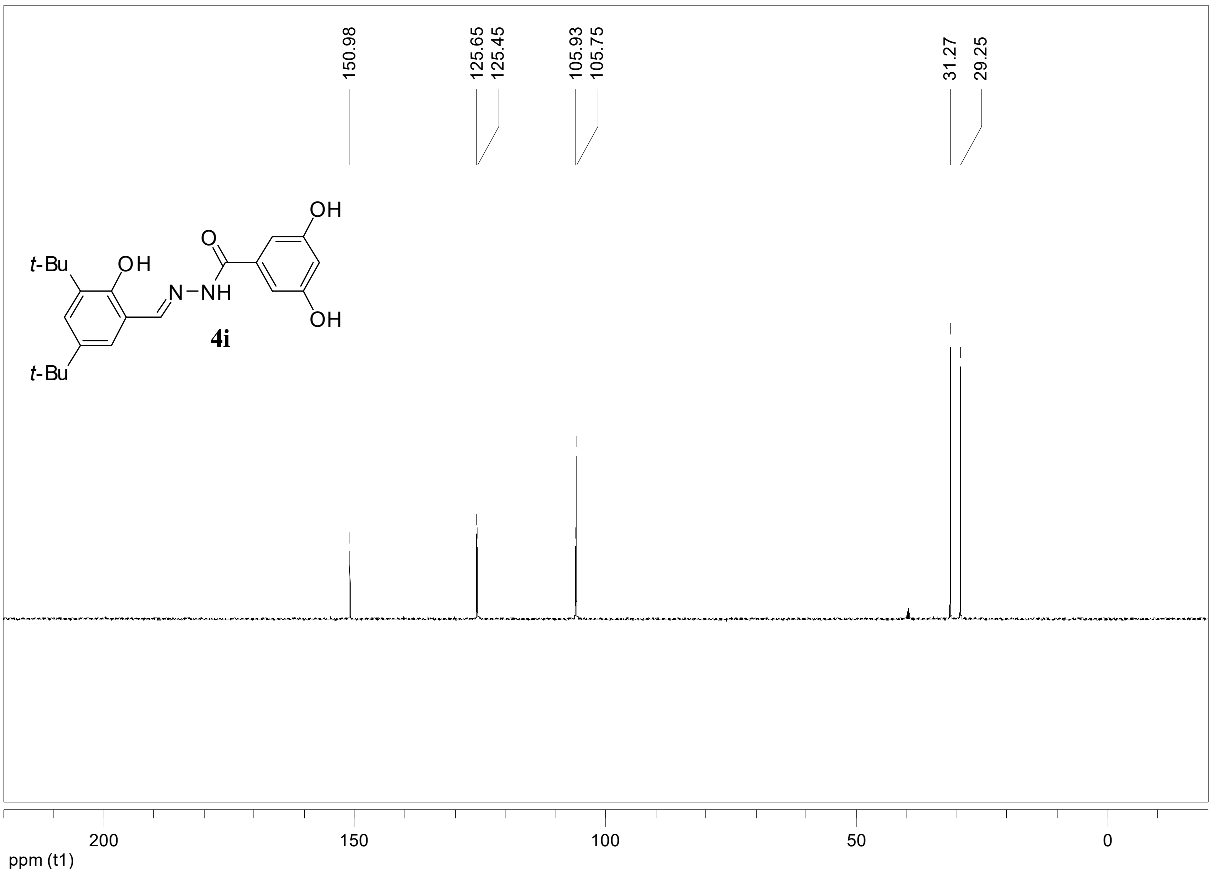

Figure S155. ^{13}C-NMR (100 MHz, DMSO-d_6) dept-135 experiment of compound **4i**

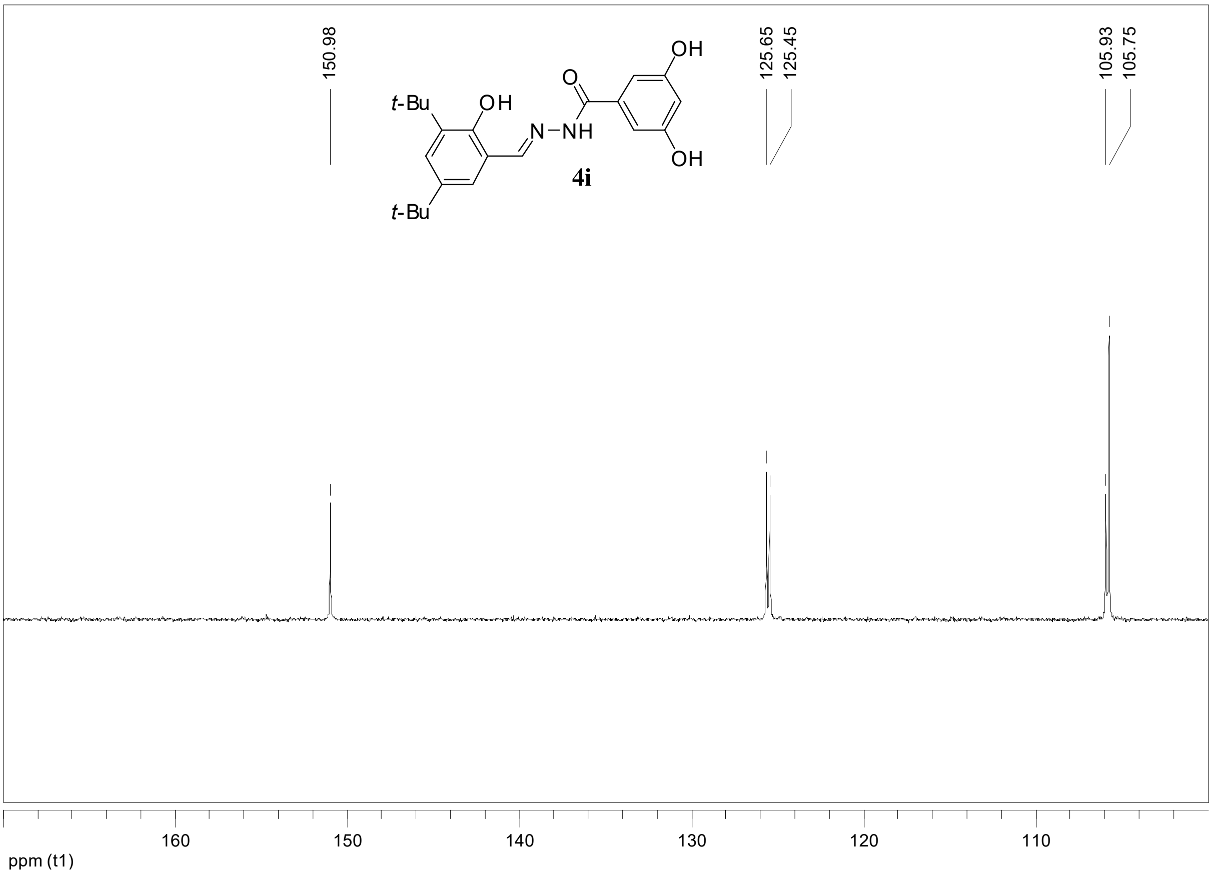

Figure S156. Expansion of ^{13}C-NMR (100 MHz, DMSO-d_6) dept-135 experiment of compound **4i**

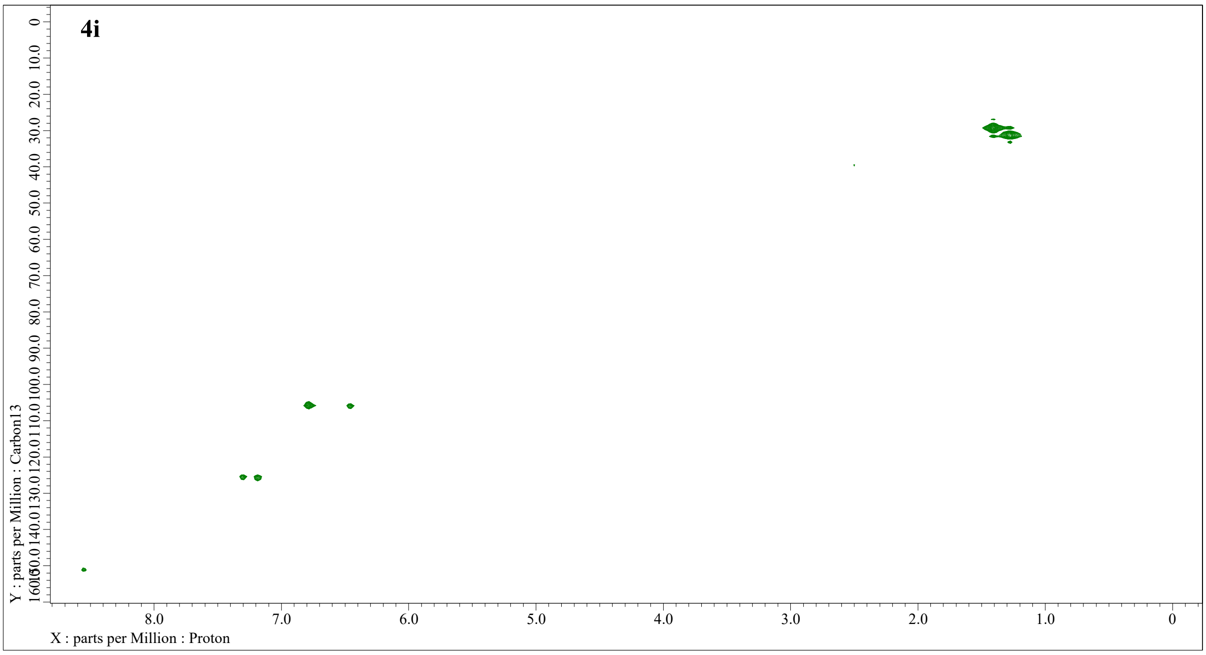

Figure S157. 2D-NMR (400 MHz, DMSO-d_6) HSQC experiment of 3,5-dihydroxy-*N*'-[(*E*)-(3,5-di-*tert*-butyl-2-hydroxyphenyl)methylidene]benzohydrazide (**4i**)

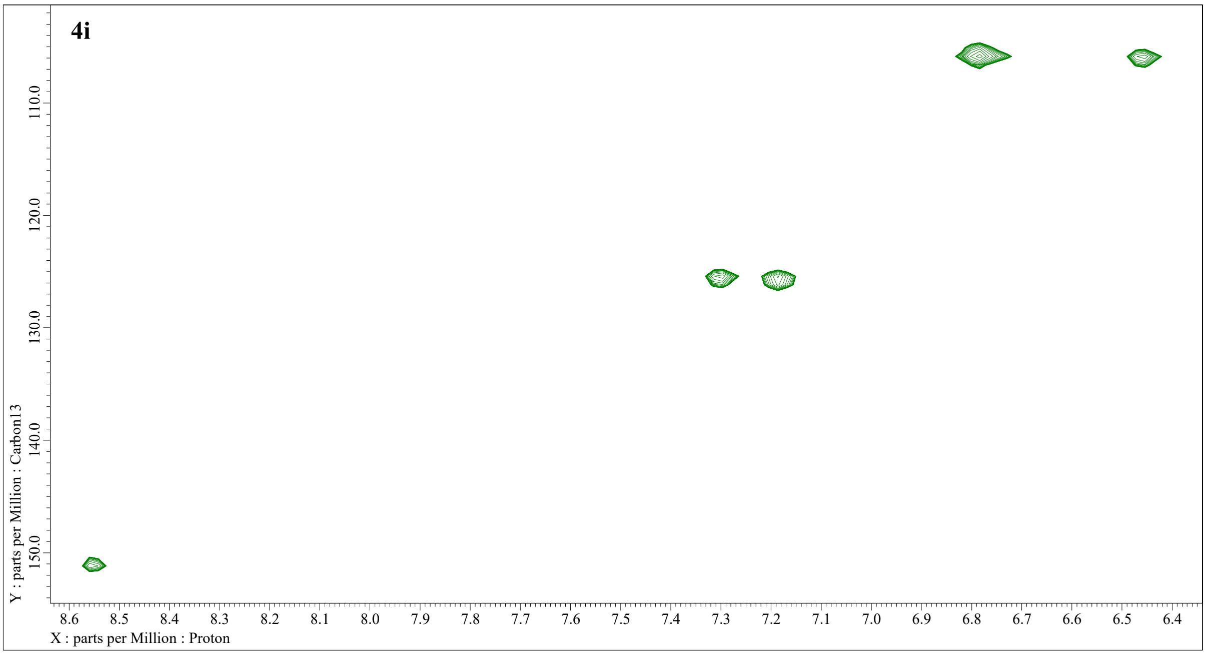

Figure S158. Expansion of 2D-NMR (400 MHz, DMSO-d_6) HSQC experiment of 3,5-dihydroxy-*N*'-[(*E*)-(3,5-di-*tert*-butyl-2-hydroxyphenyl)methylidene]benzohydrazide (**4i**)

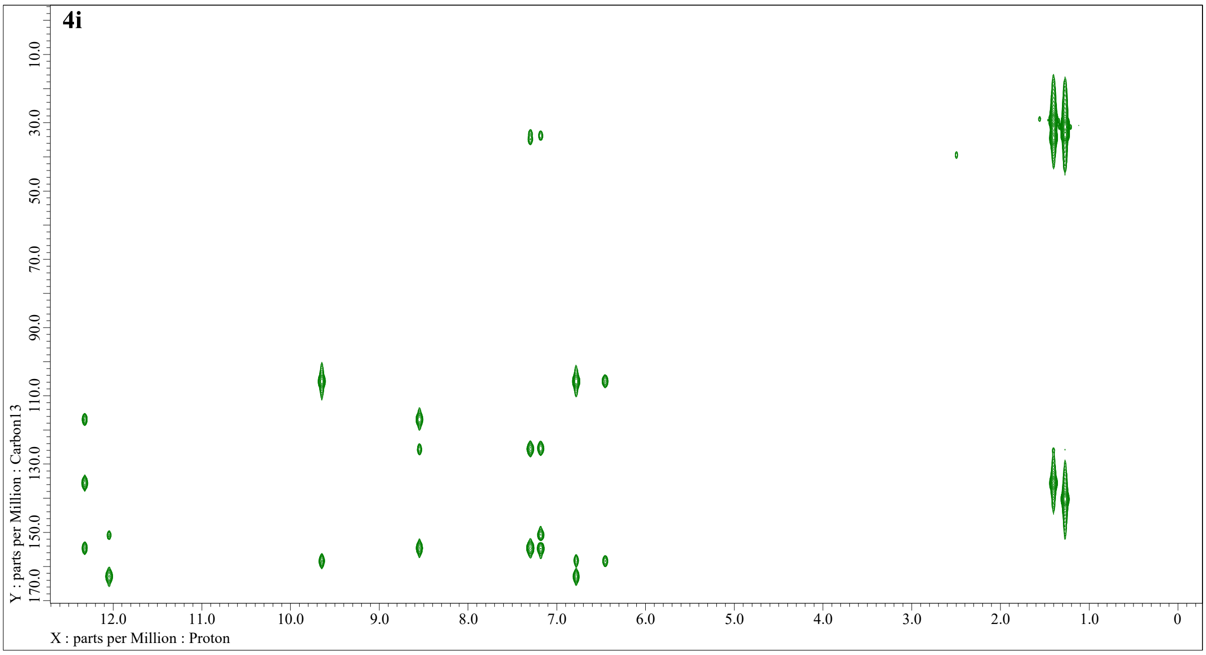

Figure S159. 2D-NMR (400 MHz, DMSO-d_6) HMBC experiment of 3,5-dihydroxy-*N*'-[(*E*)-(3,5-di-*tert*-butyl-2-hydroxyphenyl)methylidene]benzohydrazide (**4i**)

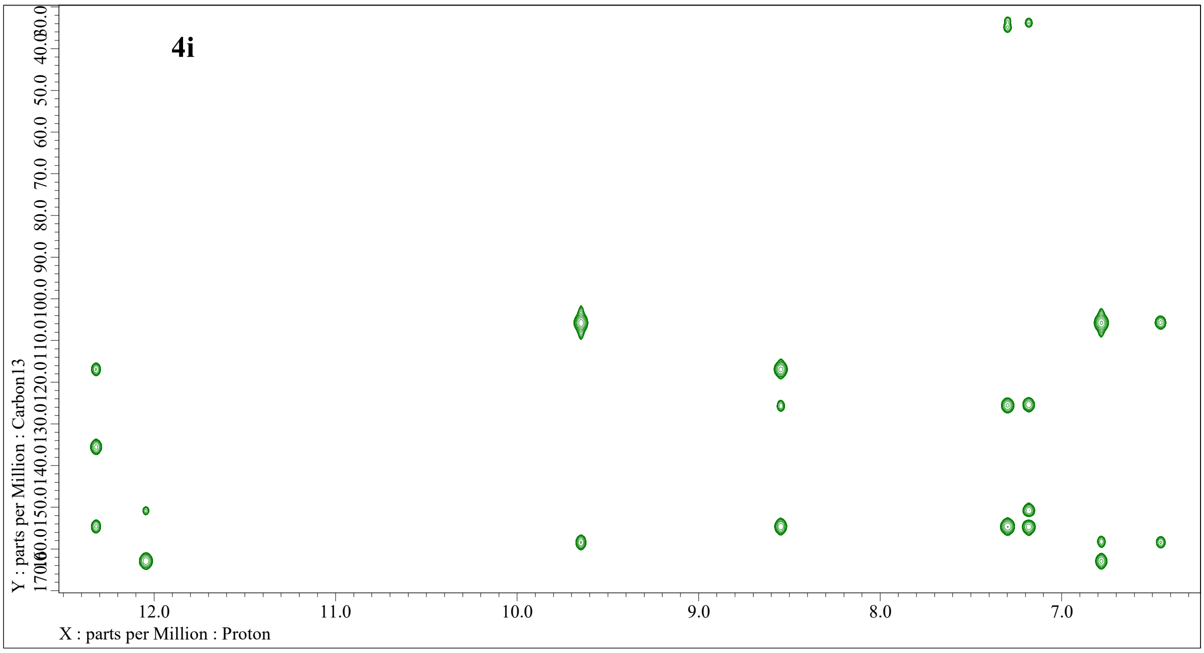

Figure S160. Expansion of 2D-NMR (400 MHz, DMSO-d_6) HMBC experiment of 3,5-dihydroxy-*N*'-[(*E*)-(3,5-di-*tert*-butyl-2-hydroxyphenyl)methylidene]benzohydrazide (**4i**)

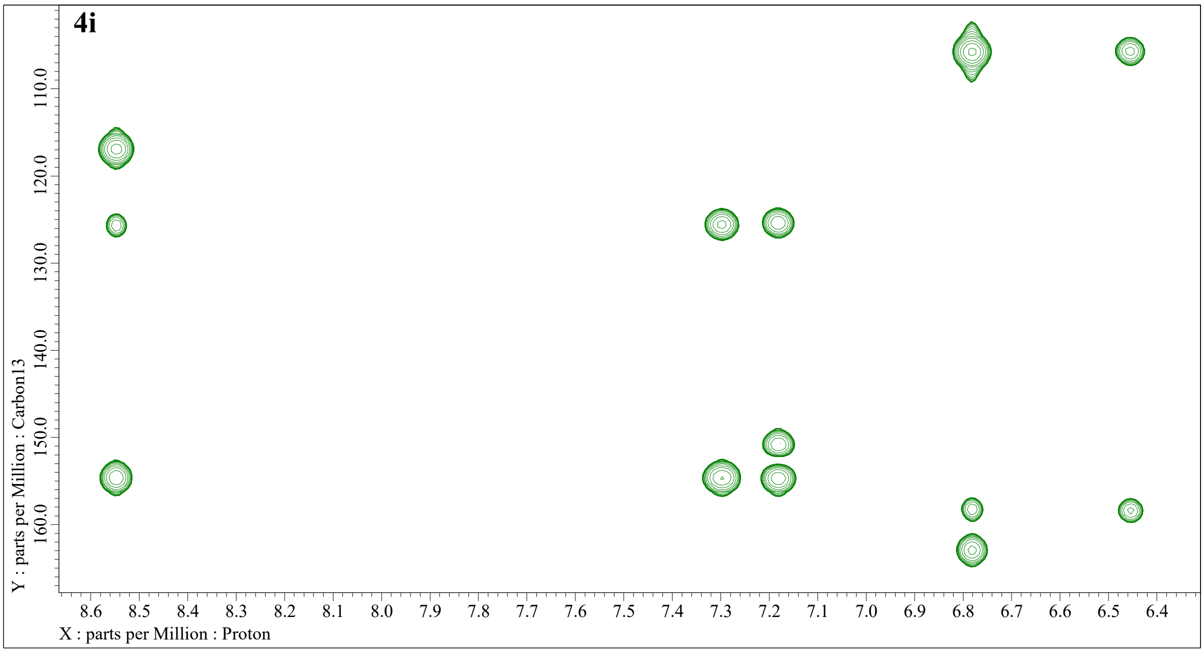

Figure S161. Expansion of 2D-NMR (400 MHz, DMSO-d_6) HMBC experiment of 3,5-dihydroxy-*N*'-[(*E*)-(3,5-di-*tert*-butyl-2-hydroxyphenyl)methylidene]benzohydrazide (**4i**)

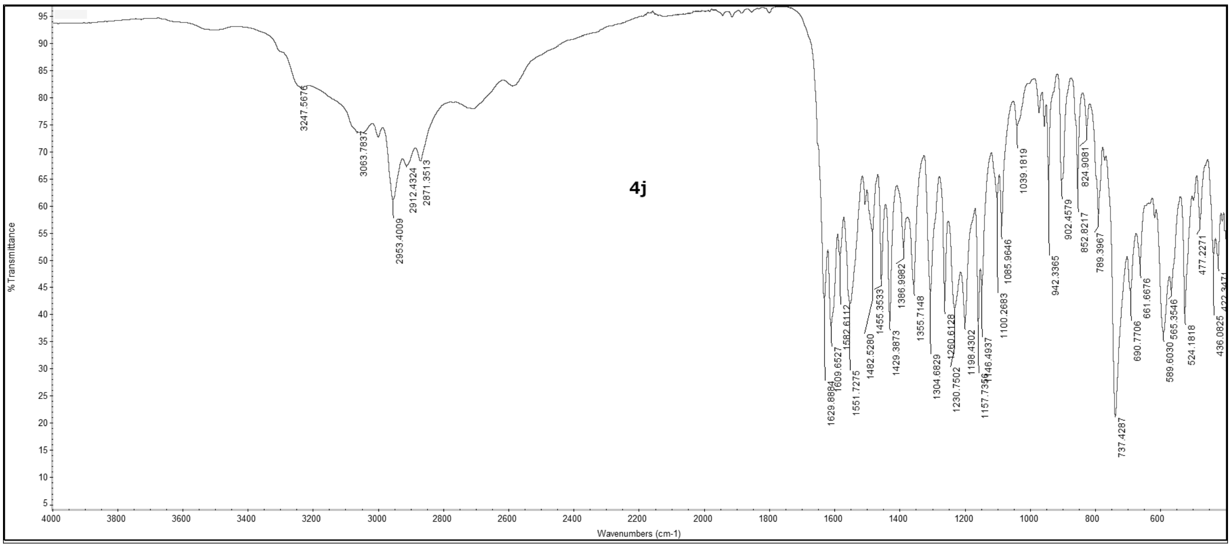

Figure S162. FT-IR (ATR) spectrum of 2-hydroxy-*N*'-[(*E*)-(3-*tert*-butyl-2-hydroxyphenyl)methylidene]benzohydrazide (**4j**)

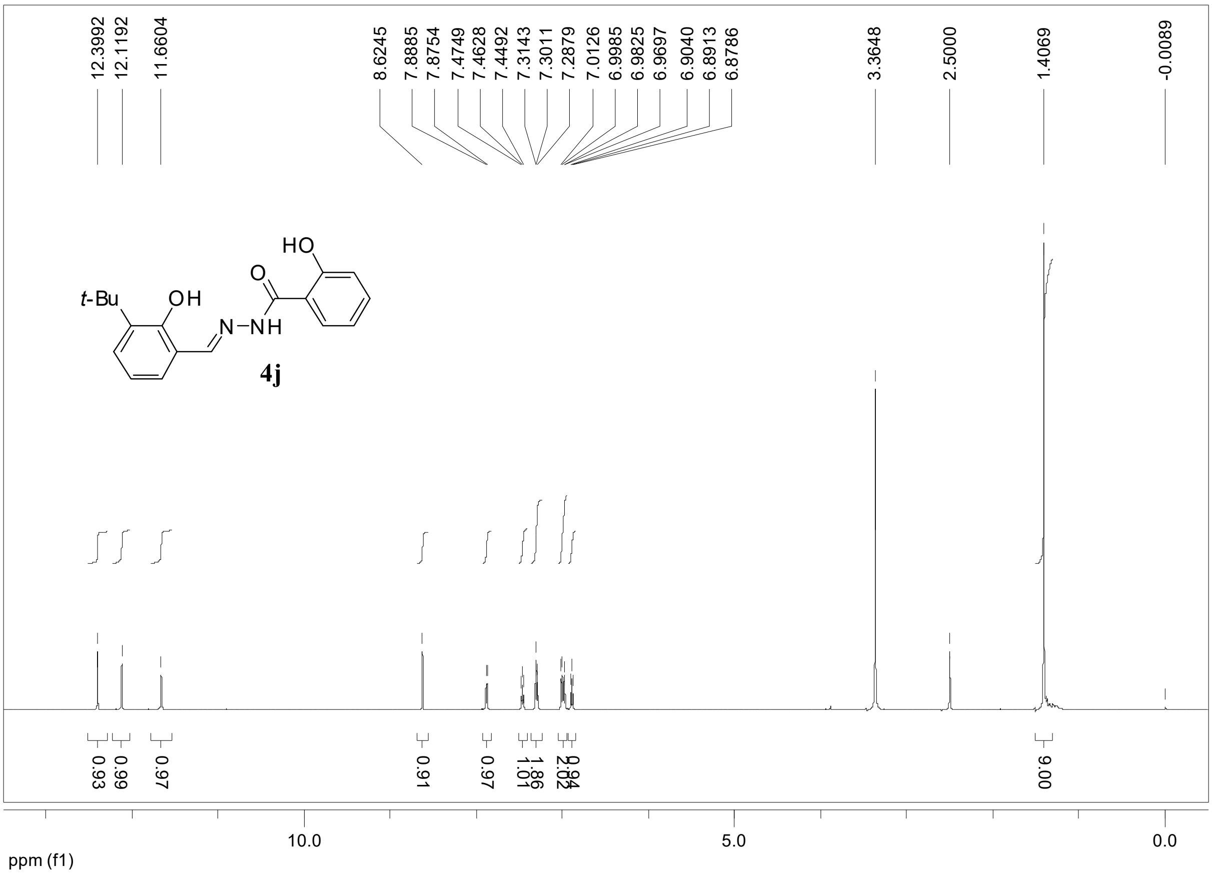

Figure S163. ^{1}H-NMR (600 MHz, DMSO-d_6) spectrum of compound **4j**

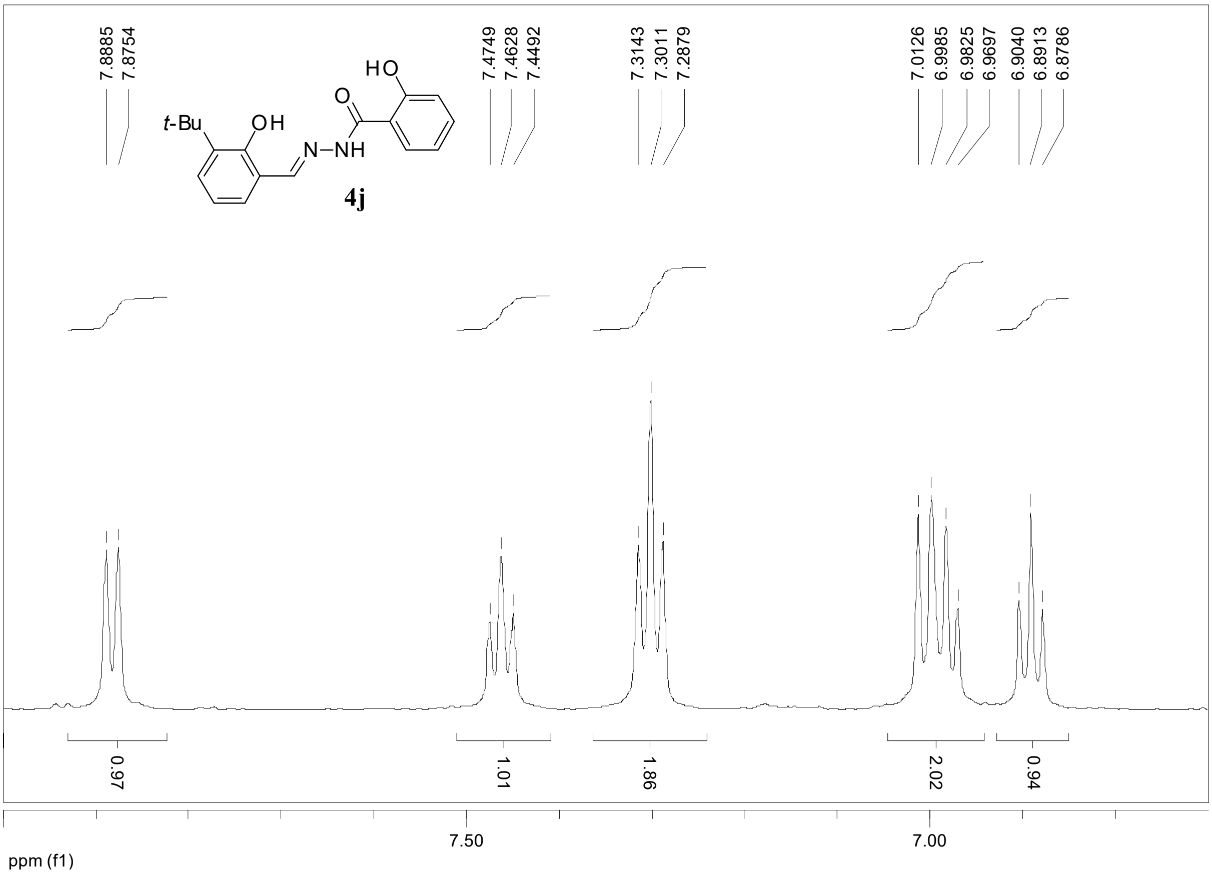

Figure S164. Expansion of ^{1}H-NMR (600 MHz, DMSO-d_6) spectrum of compound **4j**

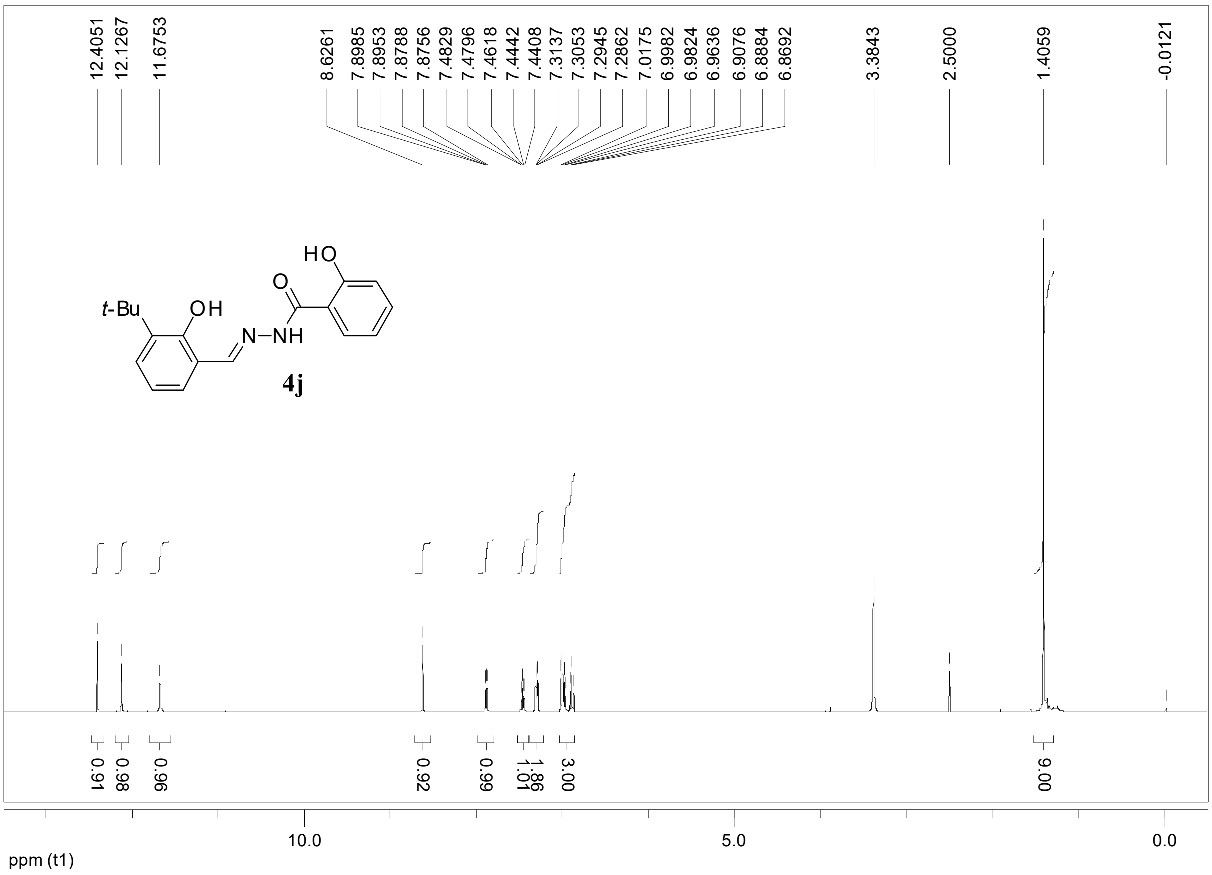

Figure S165. ^{1}H-NMR (400 MHz, DMSO-d_6) spectrum of compound **4j**

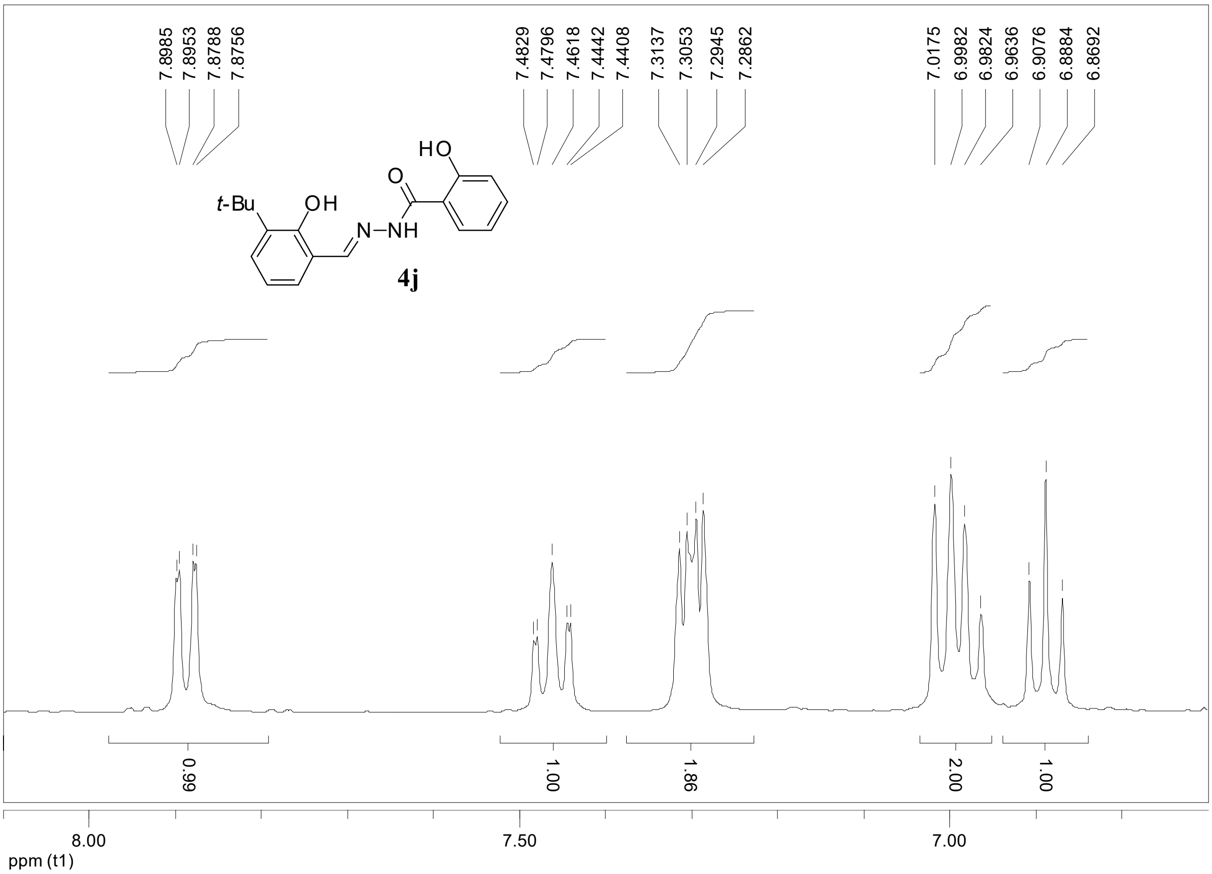

Figure S166. Expansion of ^{1}H-NMR (400 MHz, DMSO-d_6) spectrum of compound **4j**

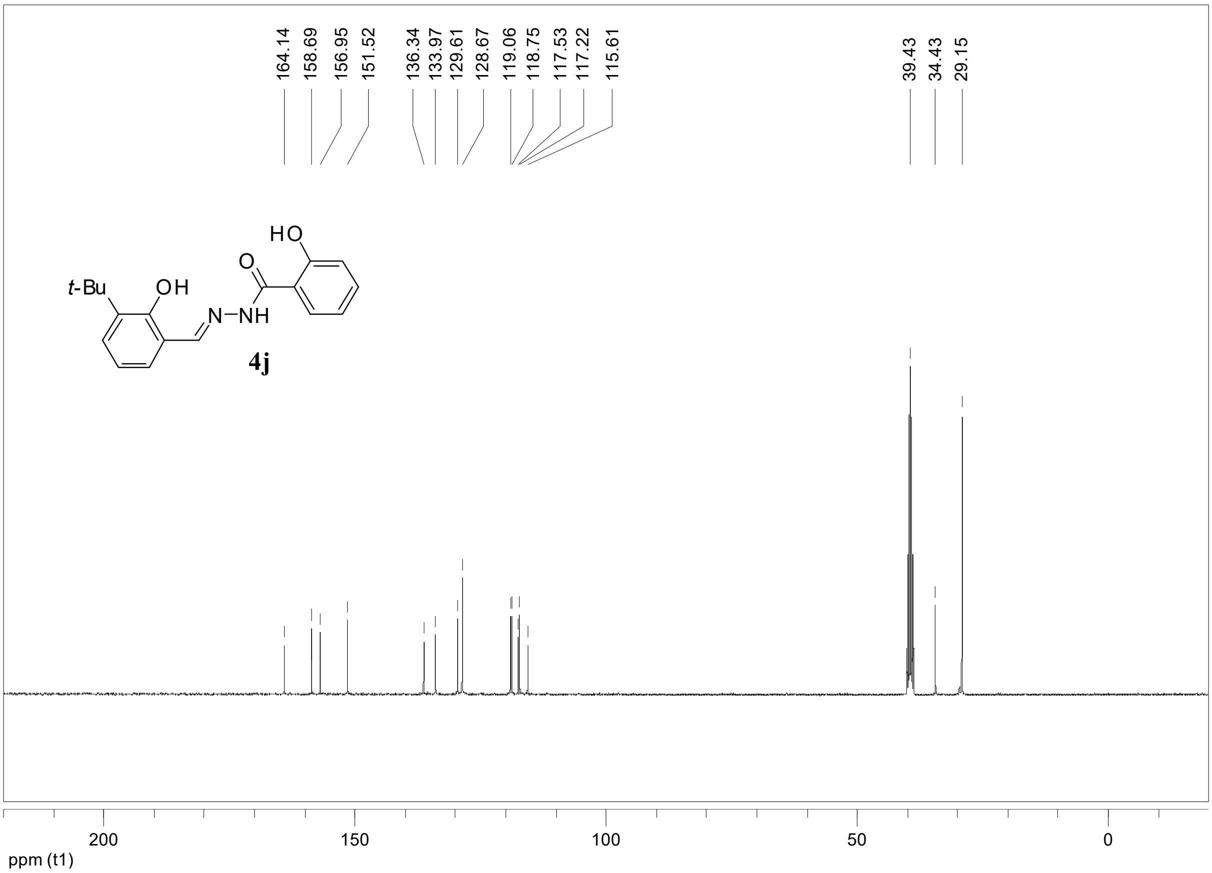

Figure S167. ^{13}C-NMR (100 MHz, DMSO-d_6) spectrum of compound **4j**

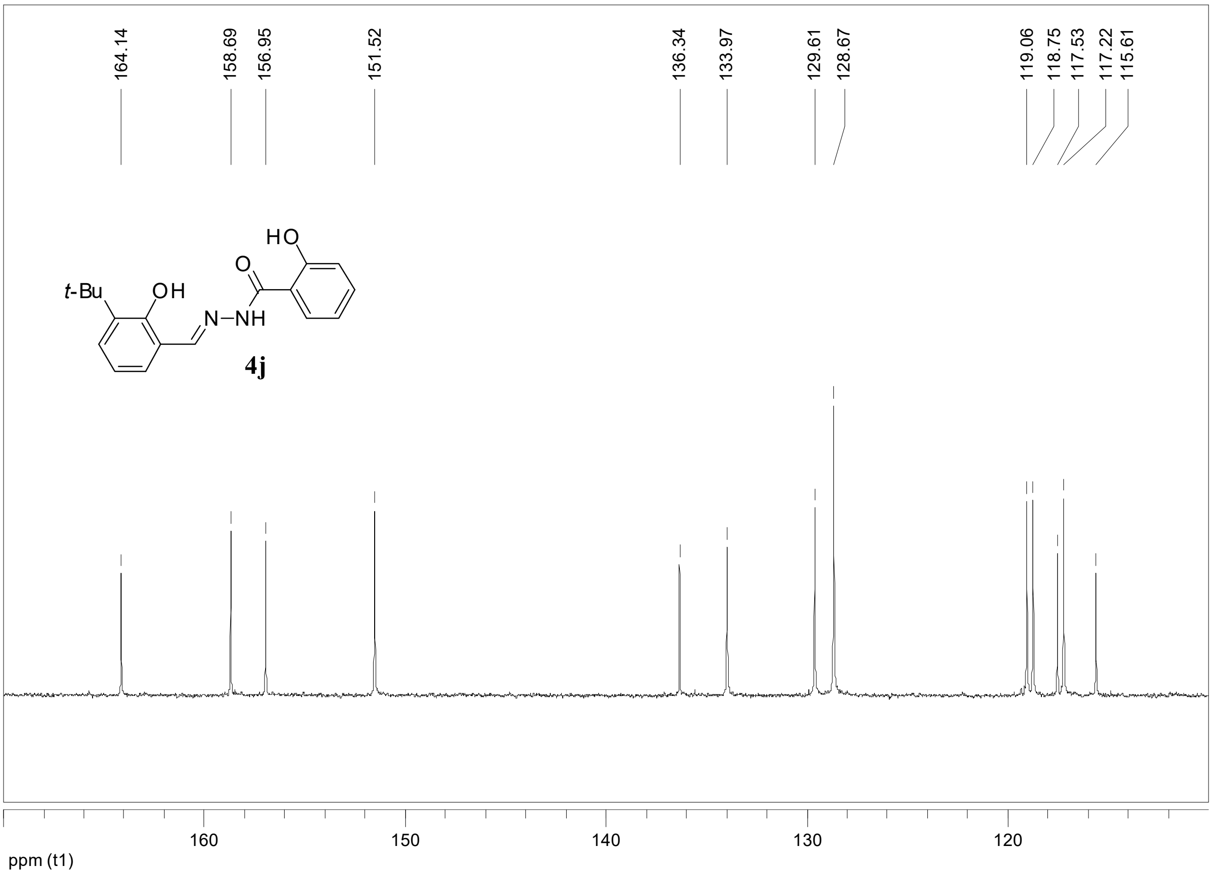

Figure S168. Expansion of ^{13}C-NMR (100 MHz, DMSO-d_6) spectrum of compound **4j**

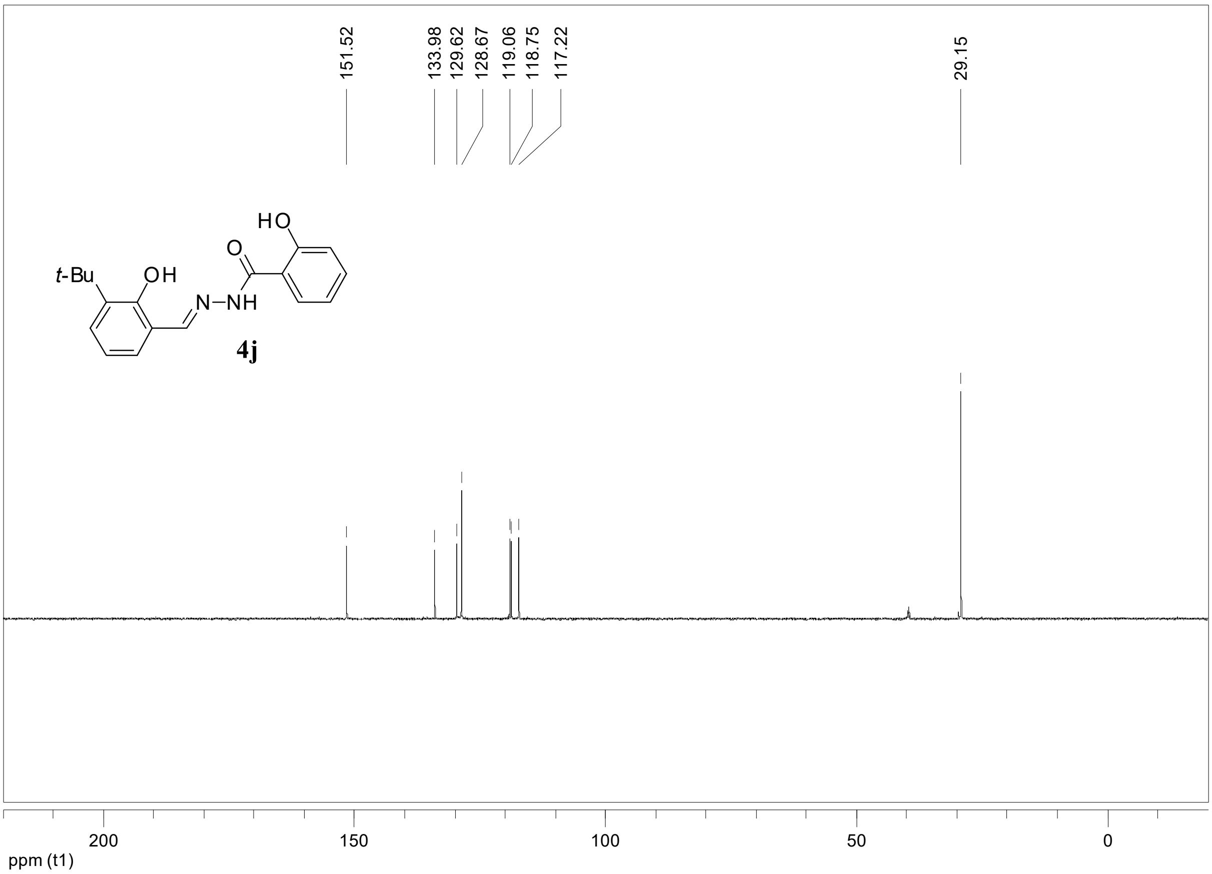

Figure S169. ^{13}C-NMR (100 MHz, DMSO-d_6) dept-135 experiment of compound **4j**

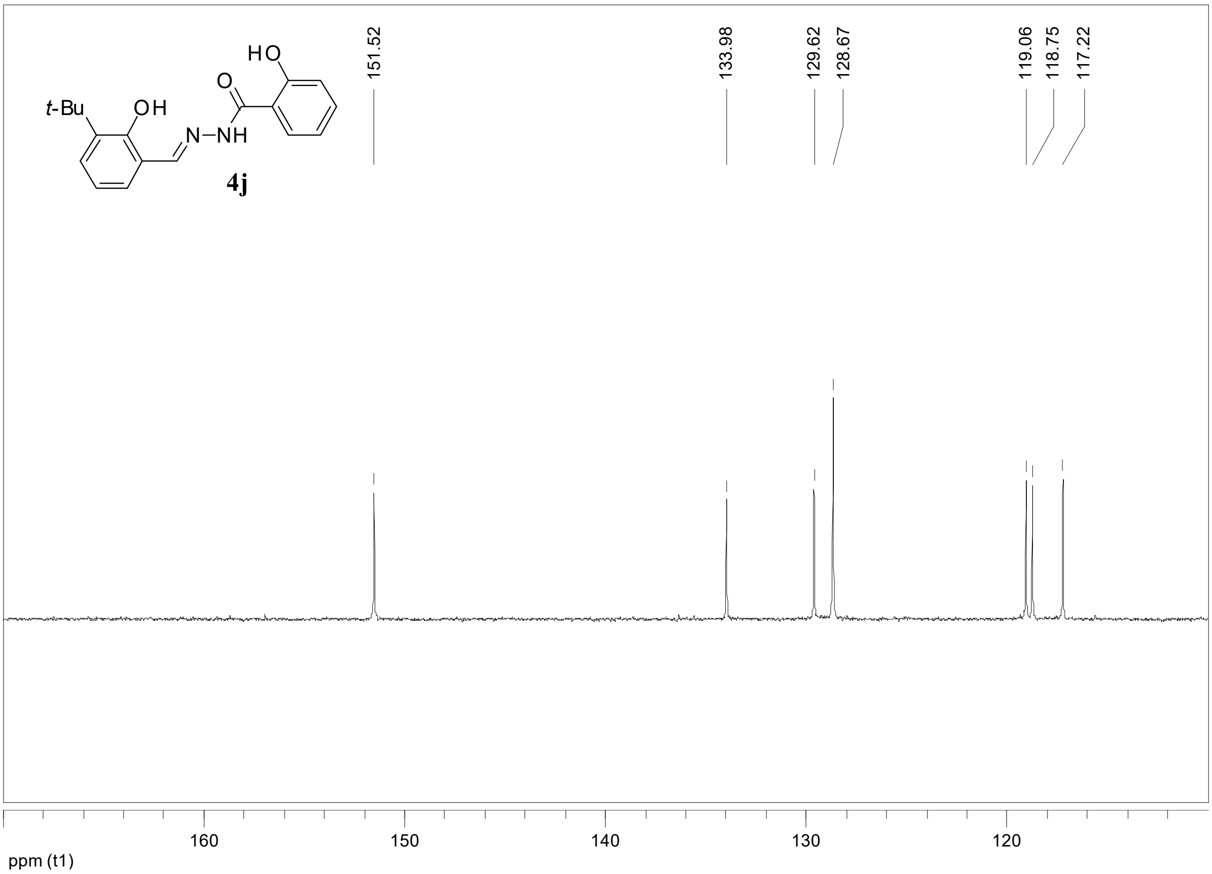

Figure S170. Expansion of ^{13}C-NMR (100 MHz, DMSO-d_6) dept-135 experiment of compound **4j**

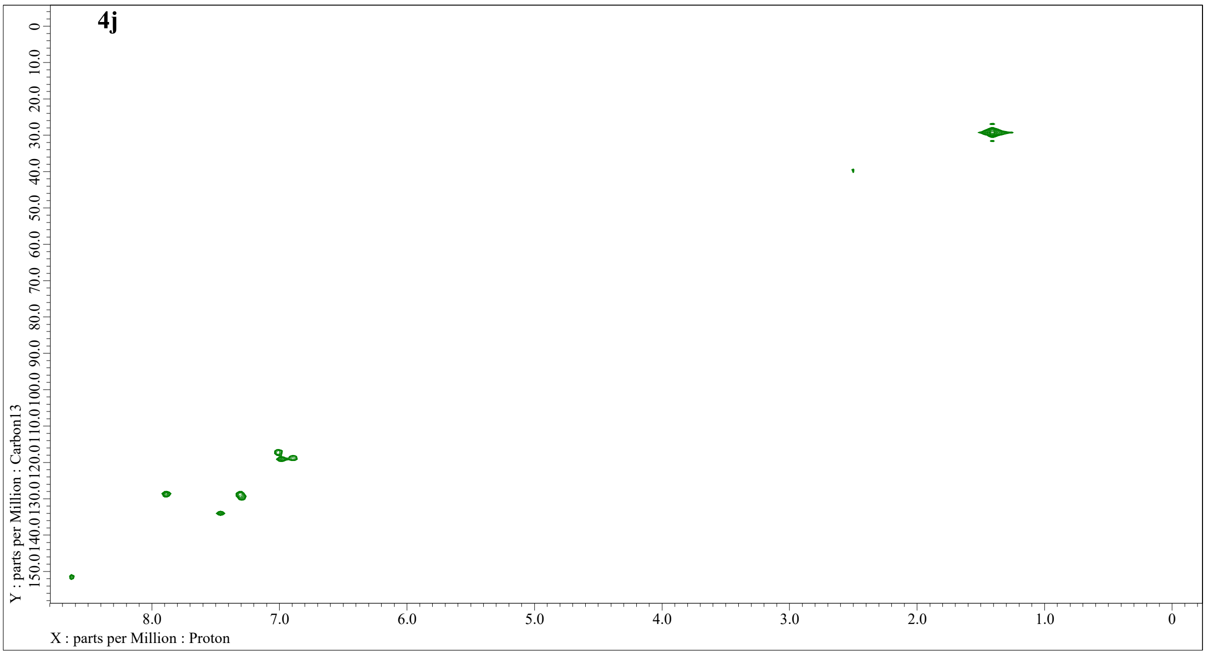

Figure S171. 2D-NMR (400 MHz, DMSO-d_6) HSQC experiment of 2-hydroxy-*N*'-[(*E*)-(3-*tert*-butyl-2-hydroxyphenyl)methylidene]benzohydrazide (**4j**)

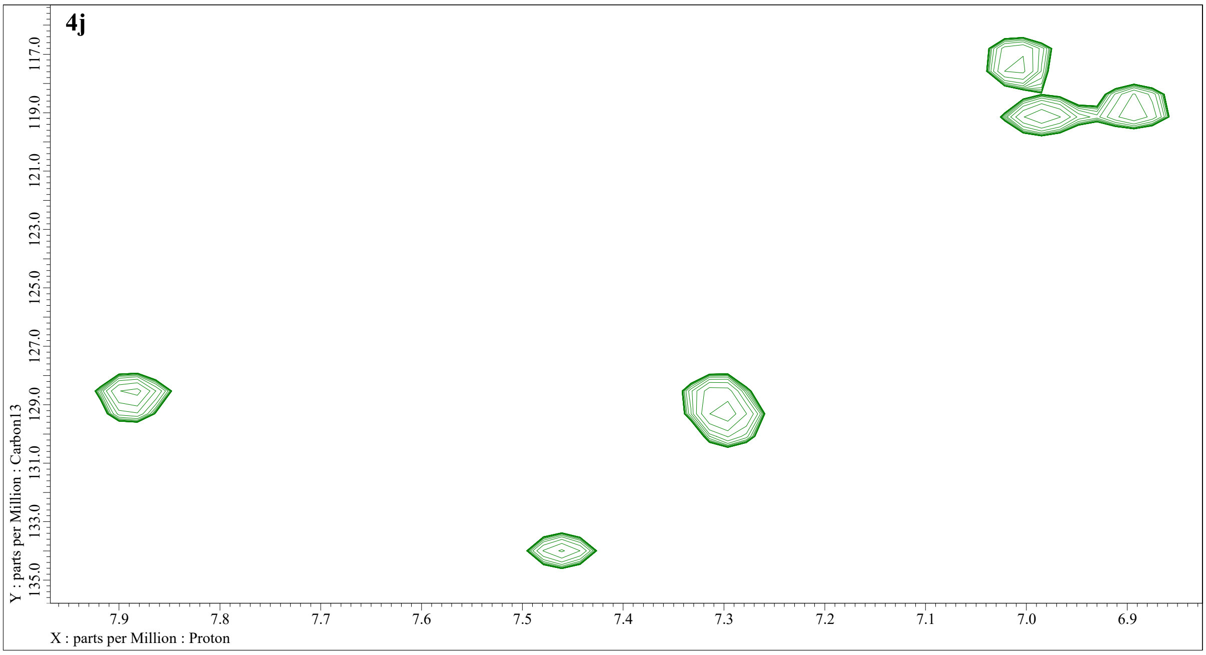

Figure S172. Expansion of 2D-NMR (400 MHz, DMSO-d_6) HSQC experiment of 2-hydroxy-*N*'-[(*E*)-(3-*tert*-butyl-2-hydroxyphenyl)methylidene]benzohydrazide (**4j**)

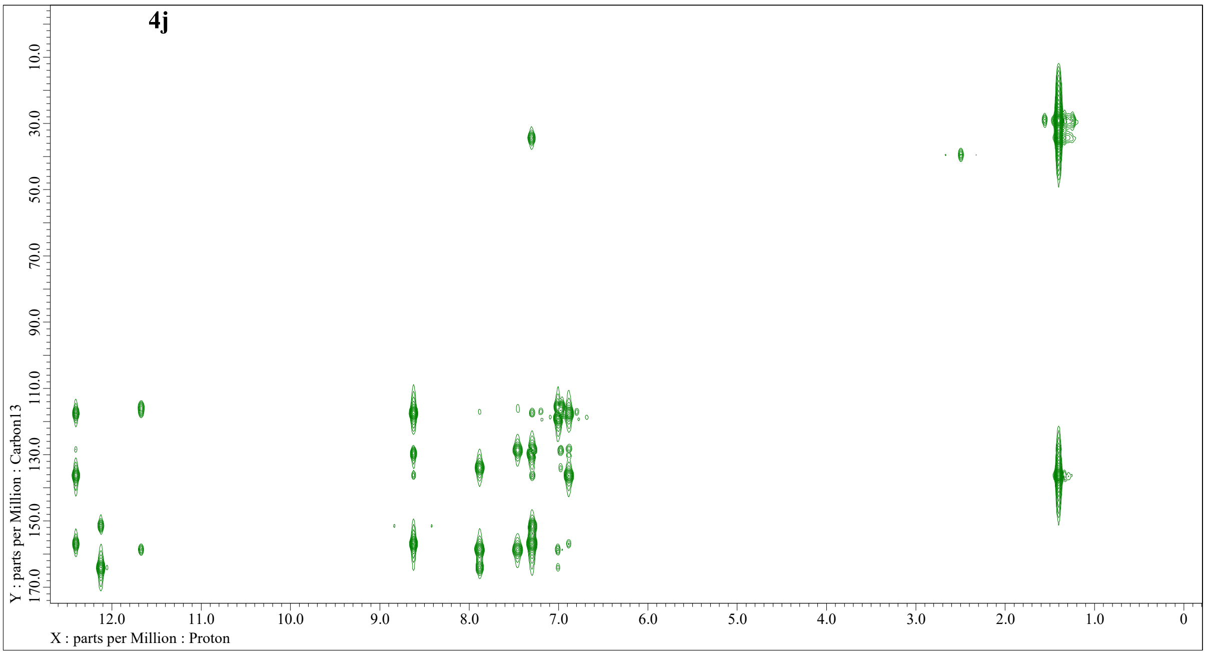

Figure S173. 2D-NMR (400 MHz, DMSO-d_6) HMBC experiment of 2-hydroxy-*N'*-[(*E*)-(3-*tert*-butyl-2-hydroxyphenyl)methylidene]benzohydrazide (**4j**)

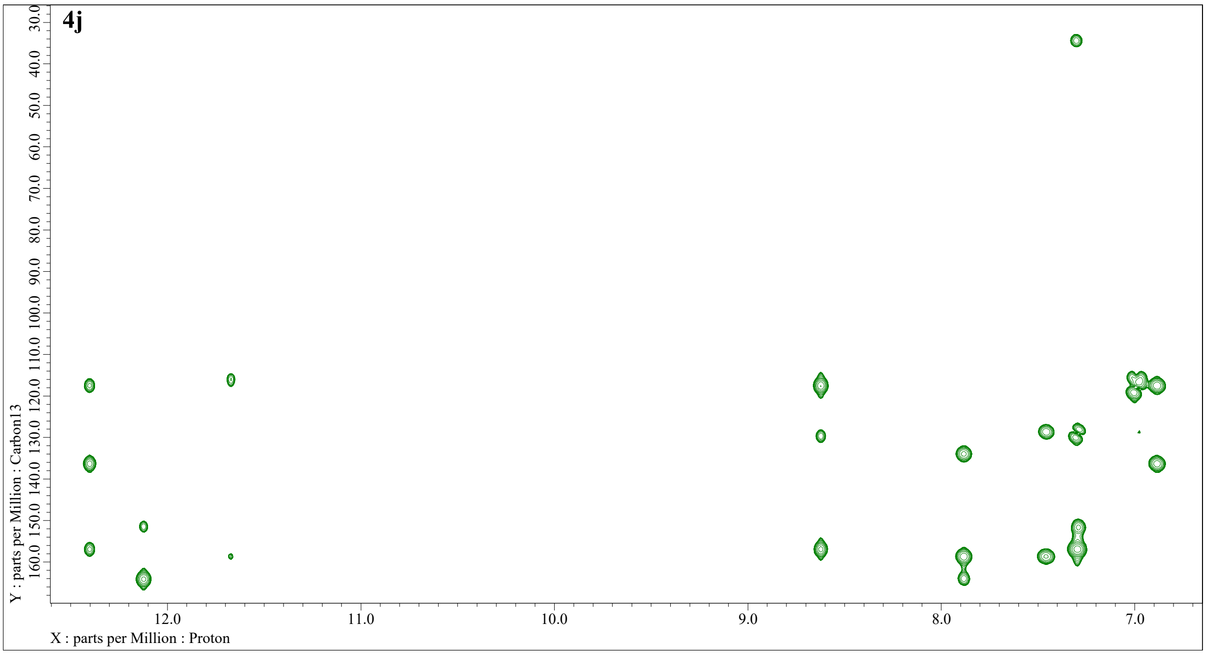

Figure S174. Expansion of 2D-NMR (400 MHz, DMSO-d_6) HMBC experiment of 2-hydroxy-*N'*-[(*E*)-(3-*tert*-butyl-2-hydroxyphenyl)methylidene]benzohydrazide (**4j**)

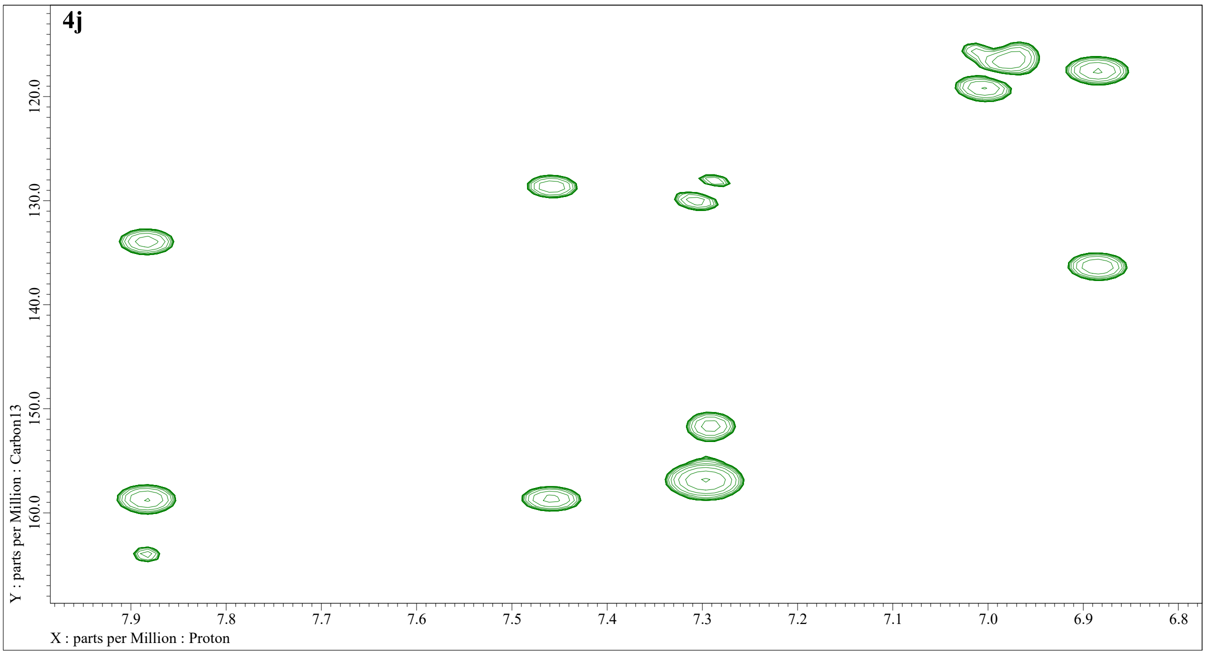

Figure S175. Expansion of 2D-NMR (400 MHz, DMSO-d_6) HMBC experiment of 2-hydroxy-*N'*-[(*E*)-(3-*tert*-butyl-2-hydroxyphenyl)methylidene]benzohydrazide (**4j**)

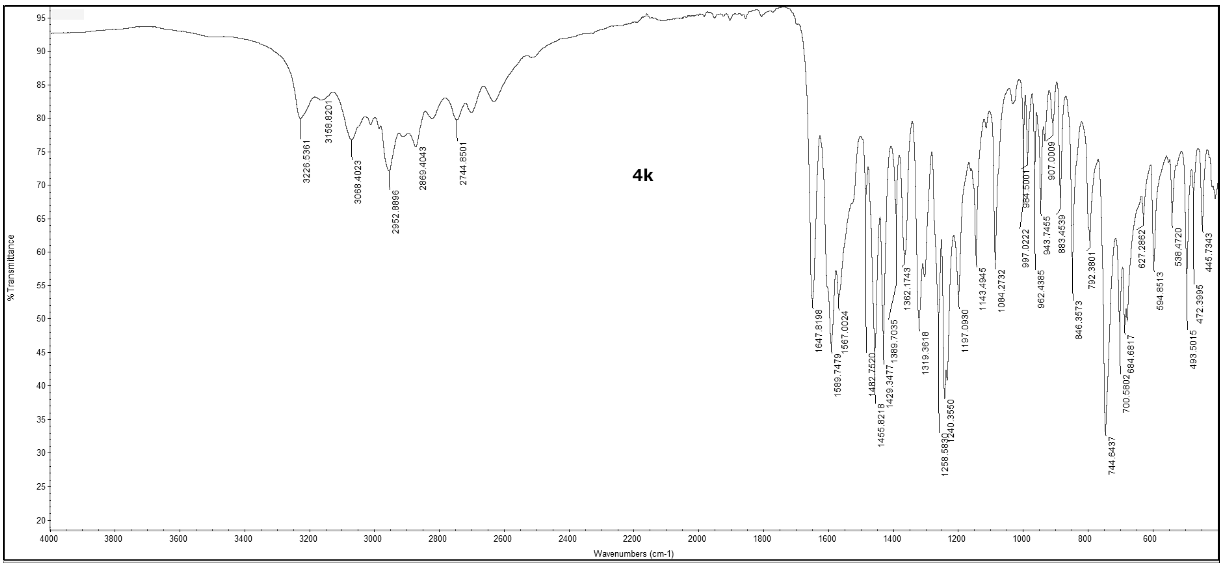

Figure S176. FT-IR (ATR) spectrum of 3-hydroxy-*N'*-[(*E*)-(3-*tert*-butyl-2-hydroxyphenyl)methylidene]benzohydrazide (**4k**)

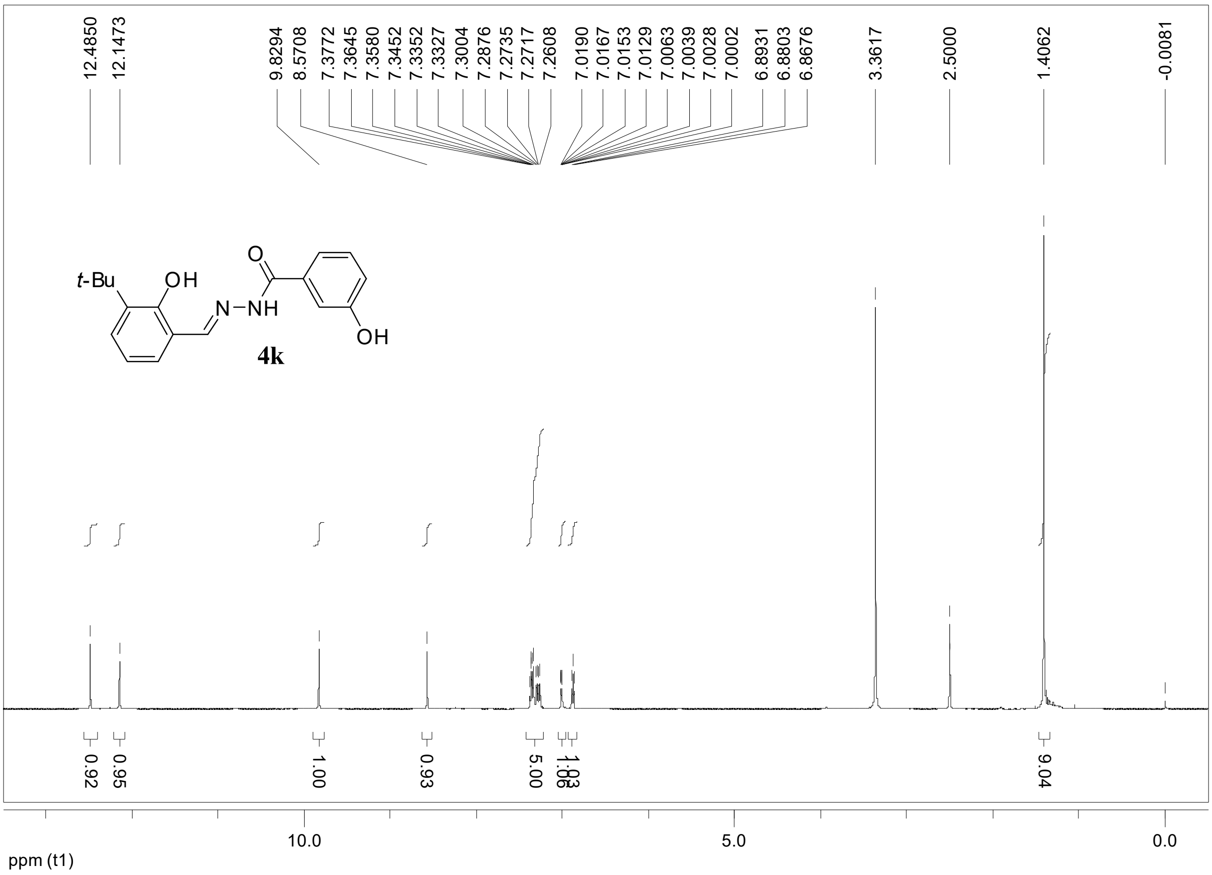

Figure S177. ^{1}H-NMR (600 MHz, DMSO-d_6) spectrum of compound **4k**

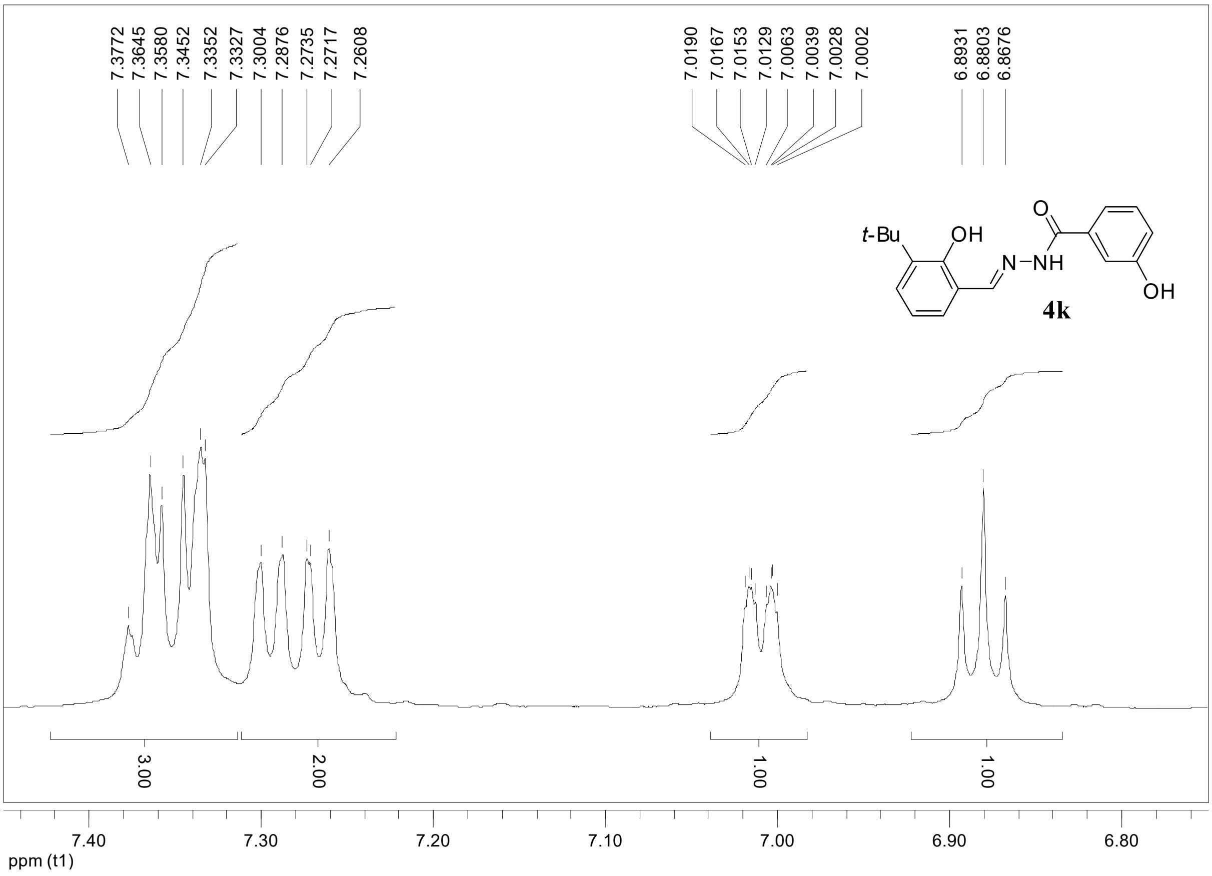

Figure S178. Expansion of ^{1}H-NMR (600 MHz, DMSO-d_6) spectrum of compound **4k**

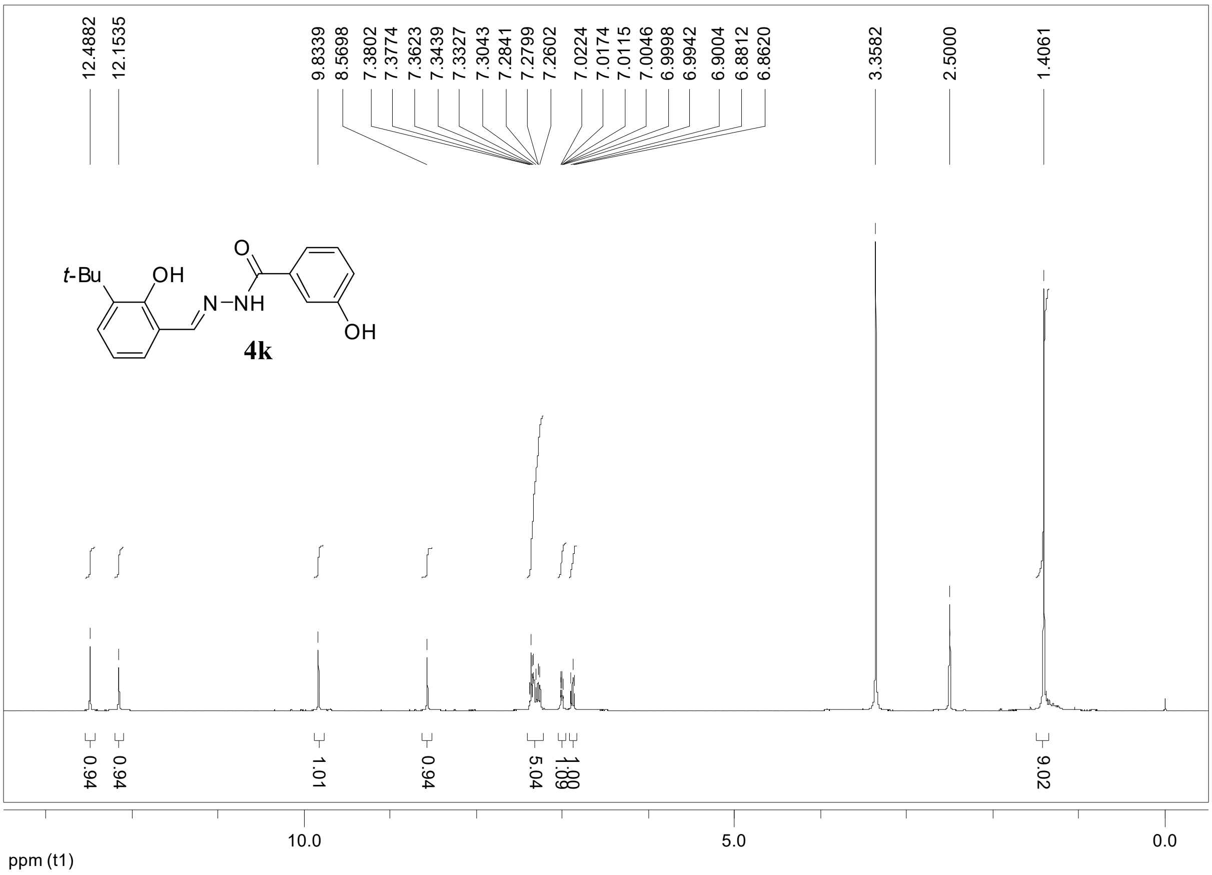

Figure S179. ^{1}H-NMR (400 MHz, DMSO-d_6) spectrum of compound **4k**

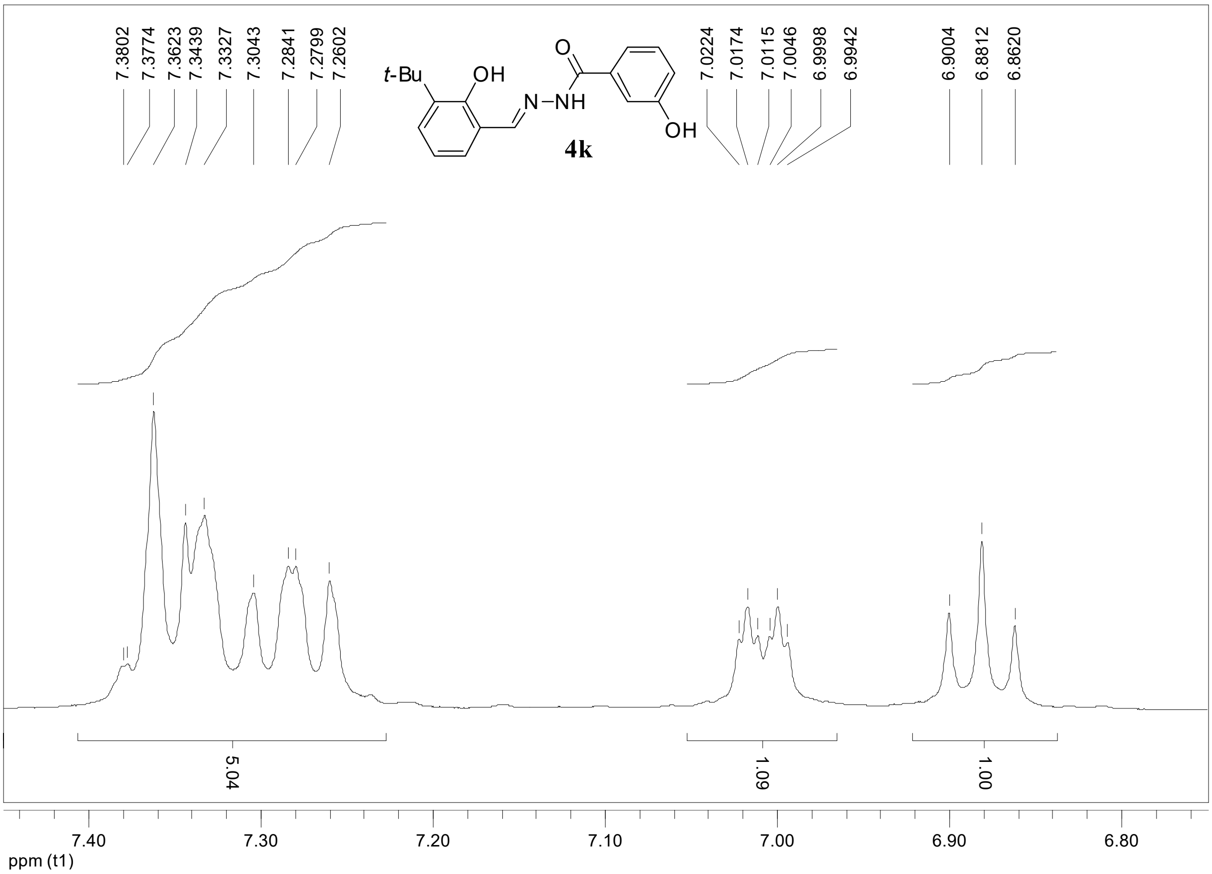

Figure S180. Expansion of ^{1}H-NMR (400 MHz, DMSO-d_6) spectrum of compound **4k**

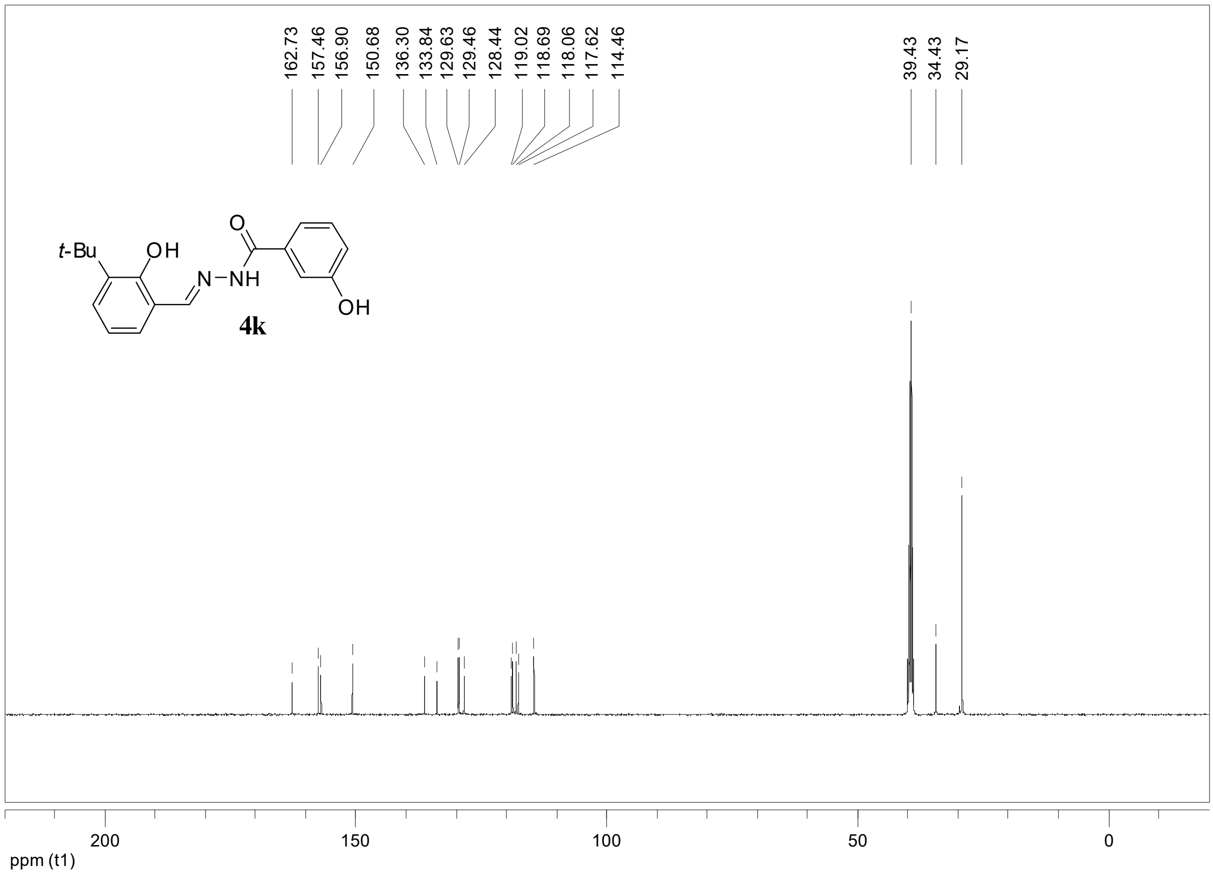

Figure S181. ^{13}C-NMR (100 MHz, DMSO-d_6) spectrum of compound **4k**

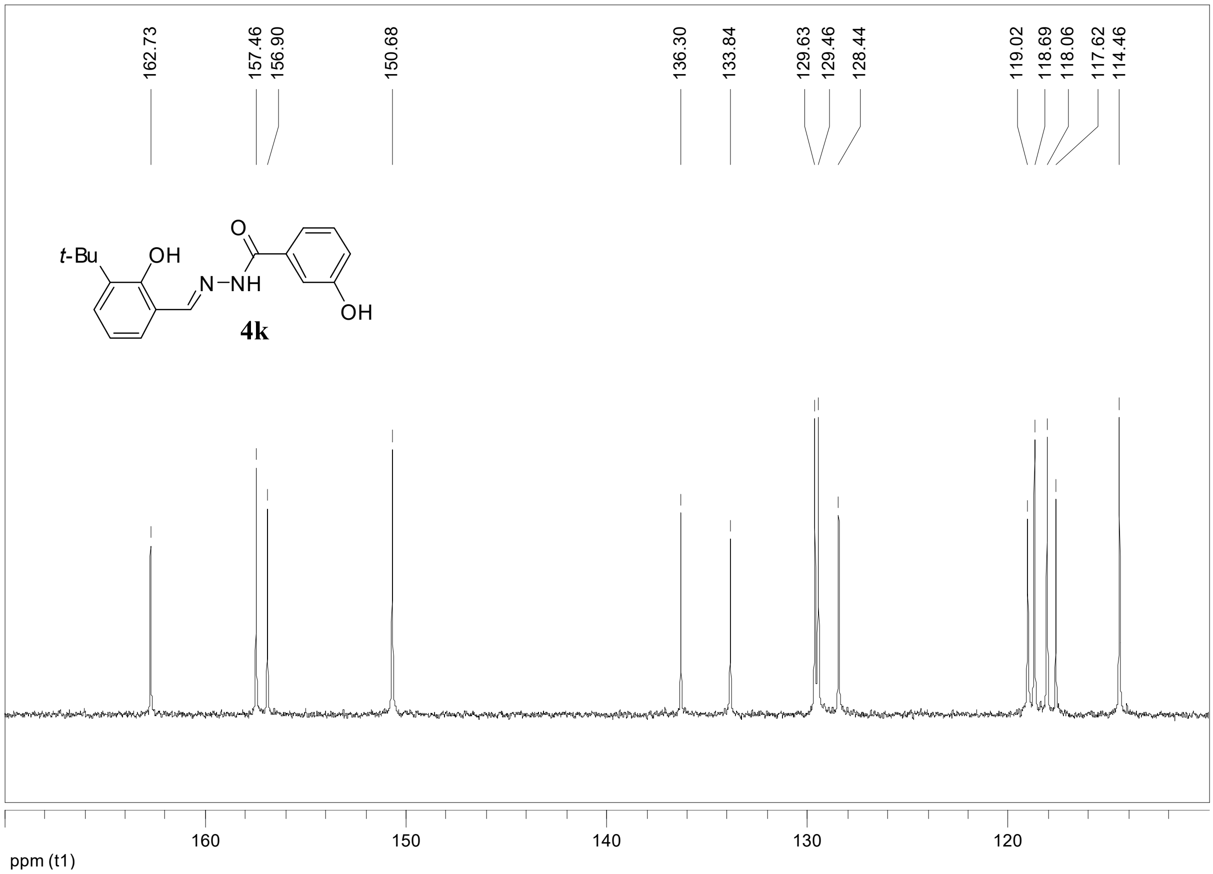

Figure S182. Expansion of ^{13}C-NMR (100 MHz, DMSO-d_6) spectrum of compound **4k**

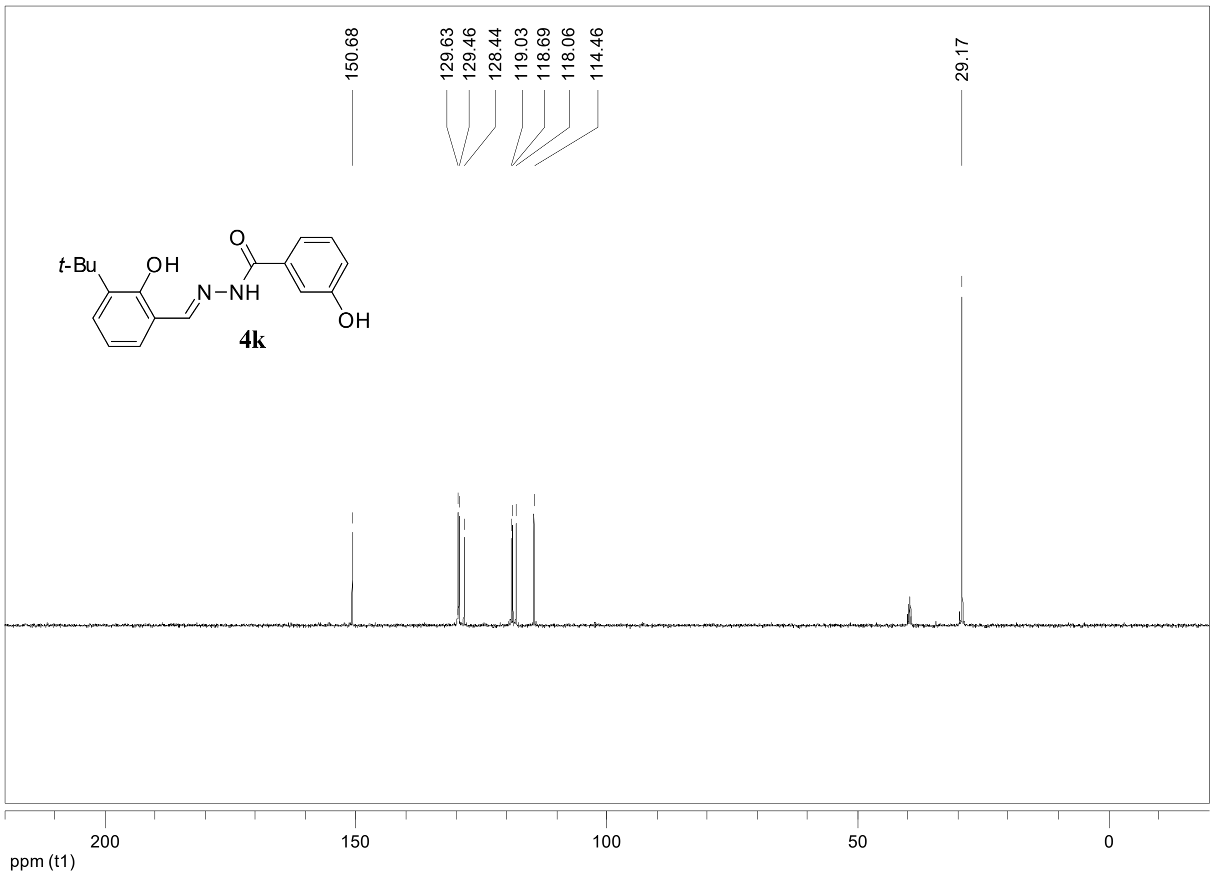

Figure S183. ^{13}C-NMR (100 MHz, DMSO-d_6) dept-135 experiment of compound **4k**

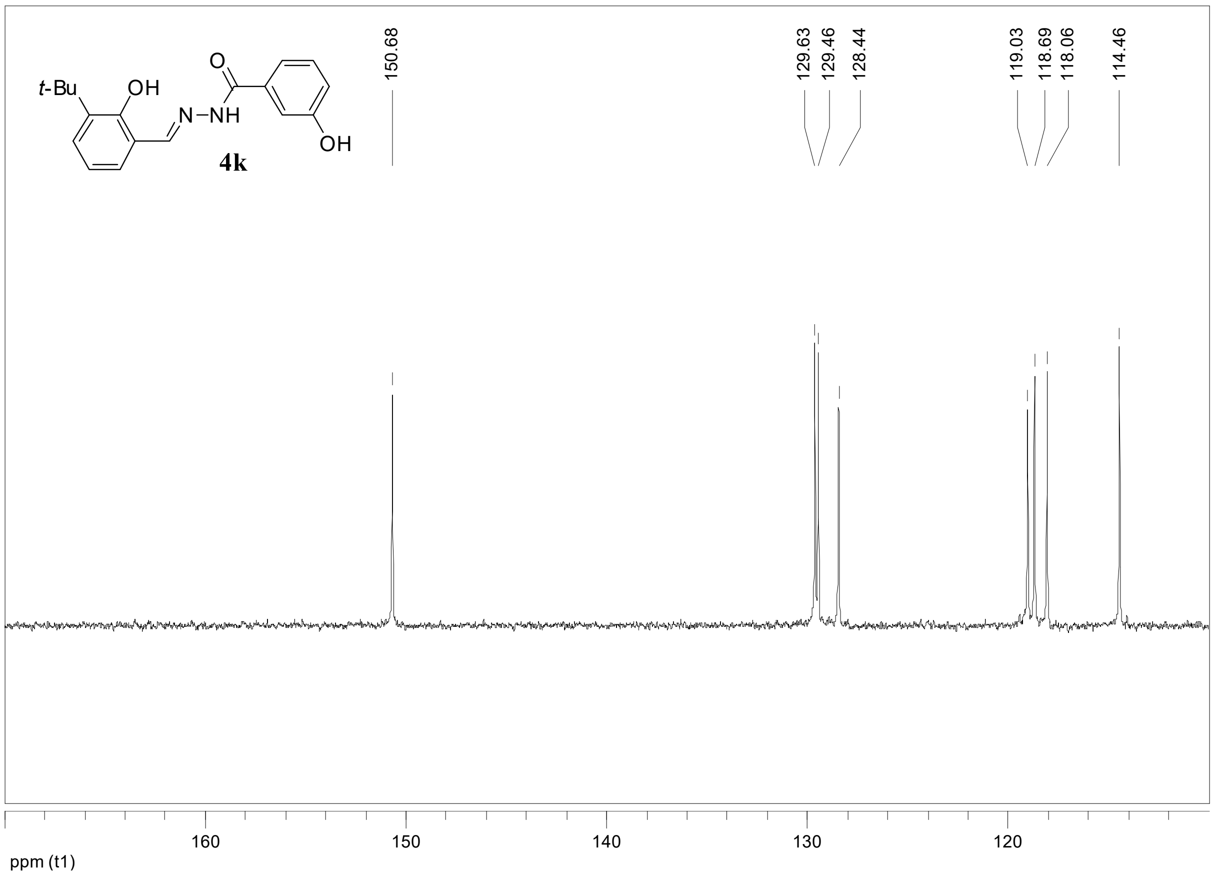

Figure S184. Expansion of ^{13}C-NMR (100 MHz, DMSO-d_6) dept-135 experiment of compound **4k**

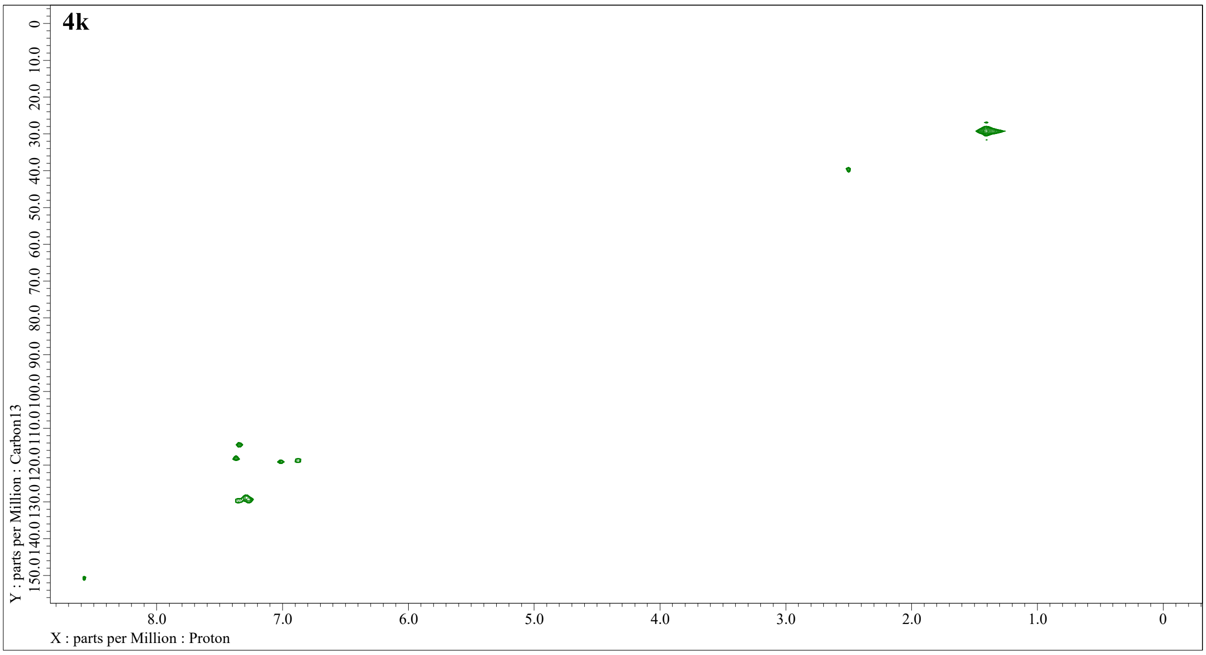

Figure S185. 2D-NMR (400 MHz, DMSO-d_6) HSQC experiment of 3-hydroxy-*N*'-[(*E*)-(3-*tert*-butyl-2-hydroxyphenyl)methylidene]benzohydrazide (**4k**)

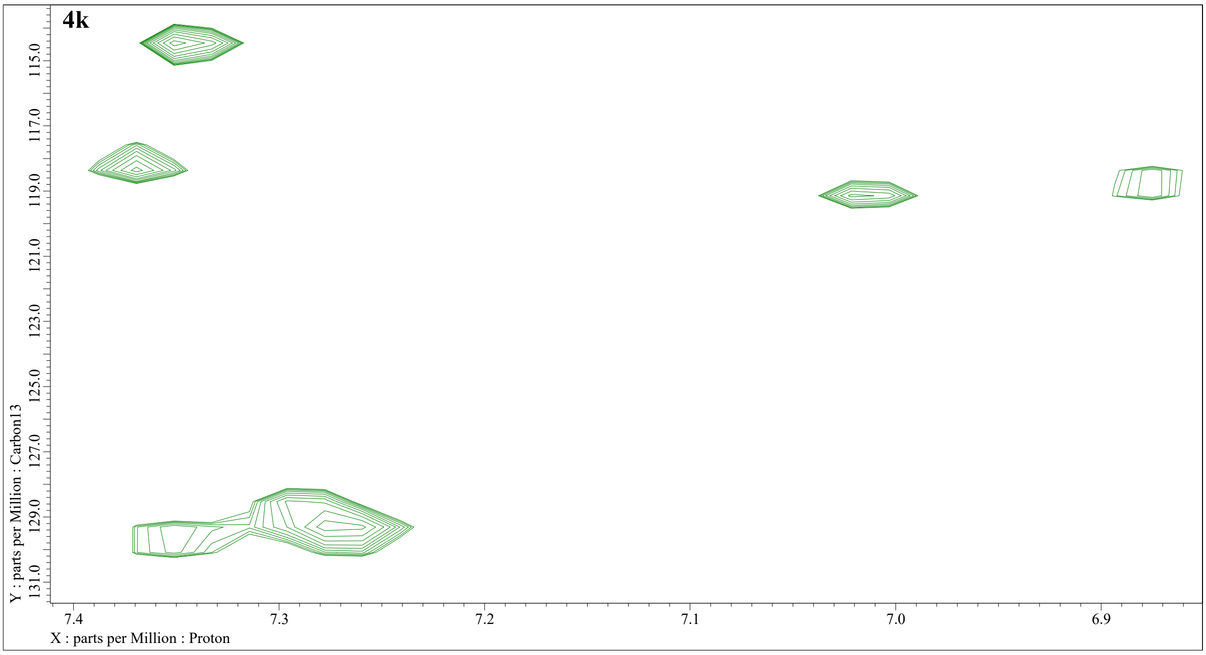

Figure S186. Expansion of 2D-NMR (400 MHz, DMSO-d_6) HSQC experiment of 3-hydroxy-*N*'-[(*E*)-(3-*tert*-butyl-2-hydroxyphenyl)methylidene]benzohydrazide (**4k**)

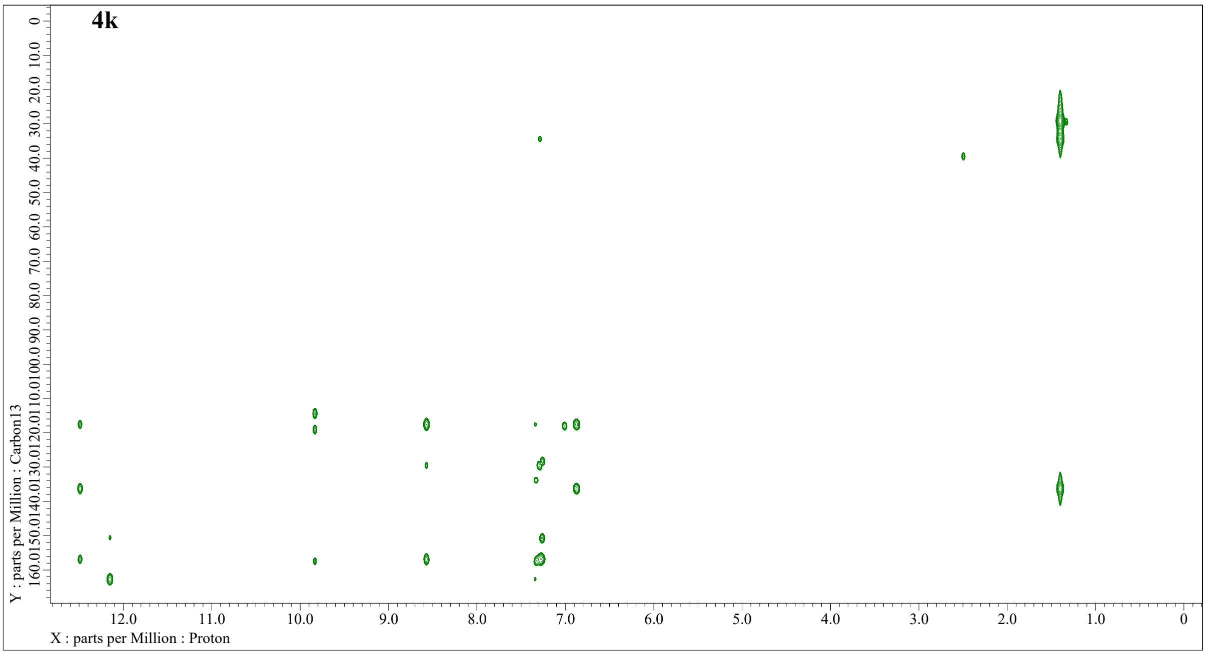

Figure S187. 2D-NMR (400 MHz, DMSO-d_6) HMBC experiment of 3-hydroxy-*N*'-[(*E*)-(3-*tert*-butyl-2-hydroxyphenyl)methylidene]benzohydrazide (**4k**)

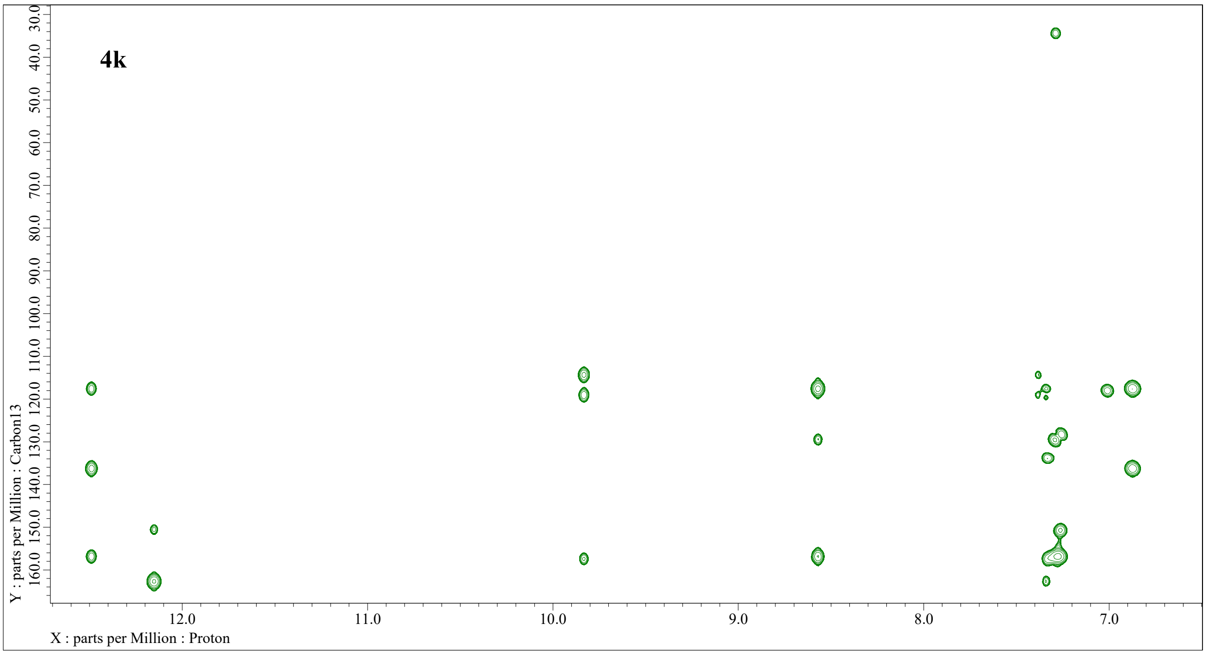

Figure S188. Expansion of 2D-NMR (400 MHz, DMSO-d_6) HMBC experiment of 3-hydroxy-*N*'-[(*E*)-(3-*tert*-butyl-2-hydroxyphenyl)methylidene]benzohydrazide (**4k**)

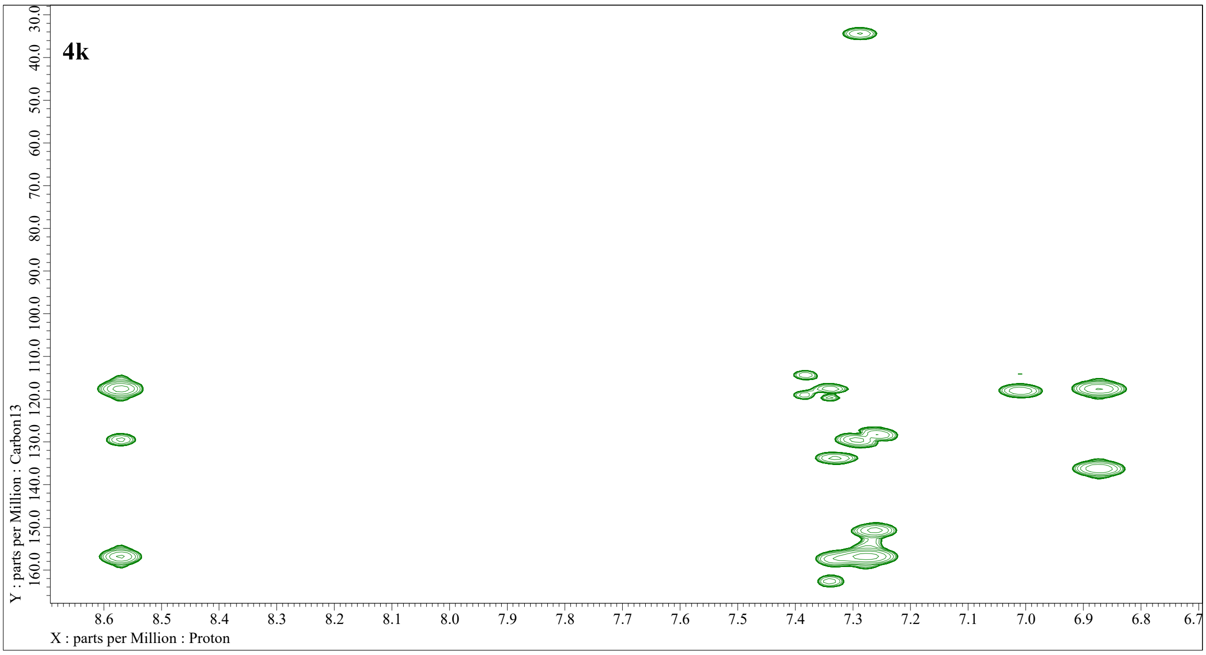

Figure S189. Expansion of 2D-NMR (400 MHz, DMSO-d_6) HMBC experiment of 3-hydroxy-*N'*-[(*E*)-(3-*tert*-butyl-2-hydroxyphenyl)methylidene]benzohydrazide (**4k**)

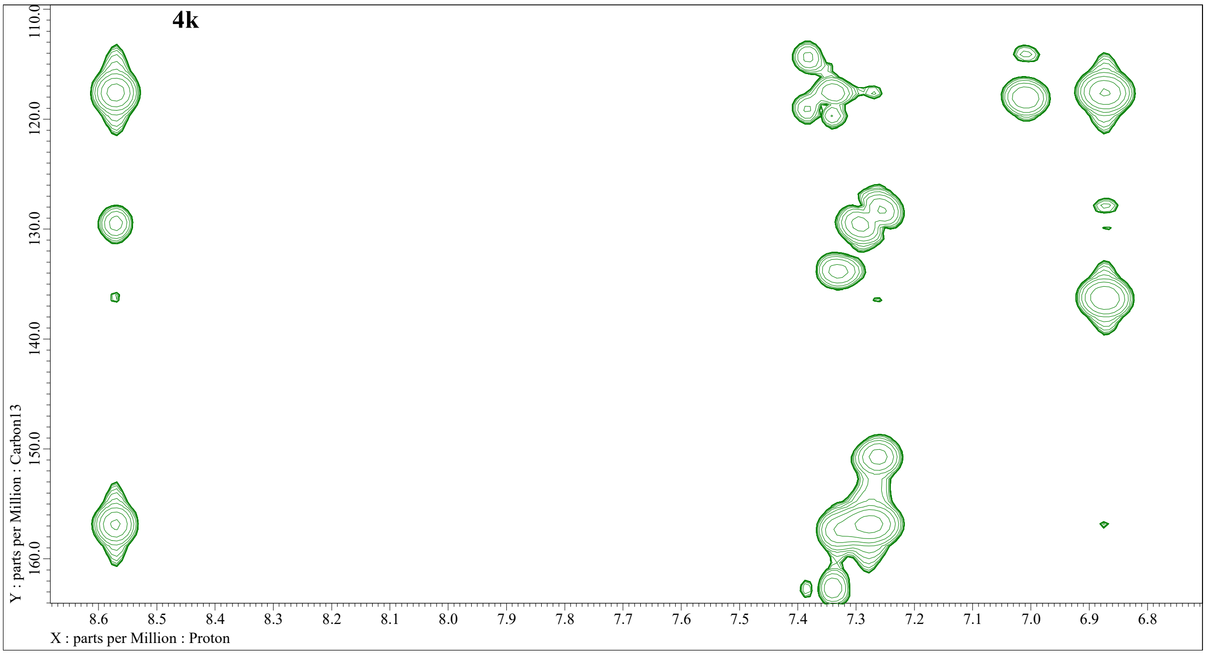

Figure S190. Expansion of 2D-NMR (400 MHz, DMSO-d_6) HMBC experiment of 3-hydroxy-*N'*-[(*E*)-(3-*tert*-butyl-2-hydroxyphenyl)methylidene]benzohydrazide (**4k**)

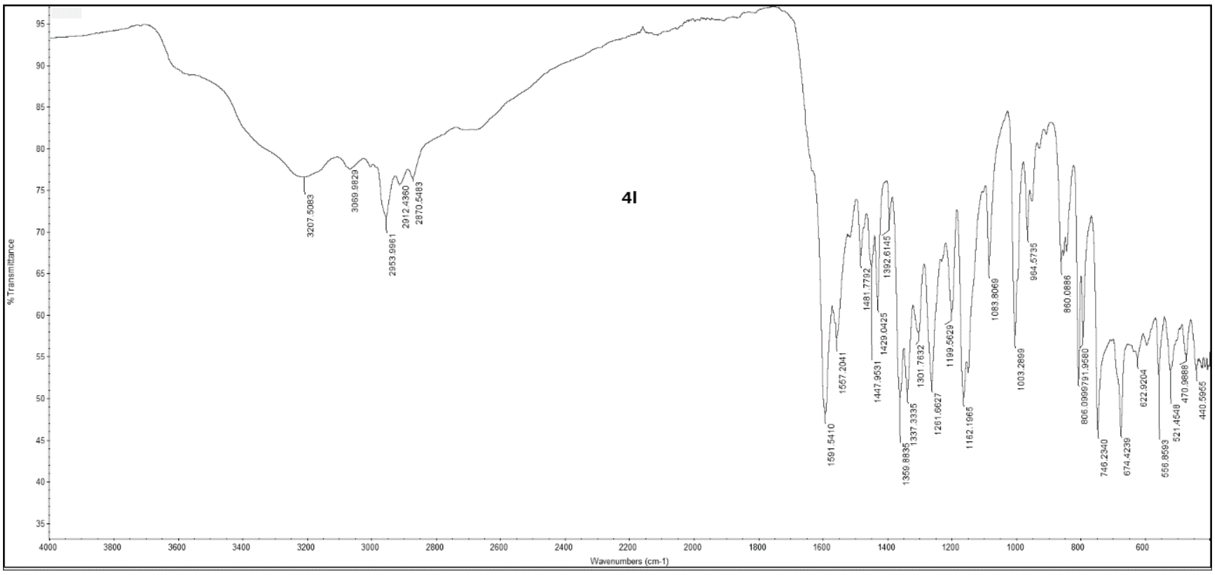

Figure S191. FT-IR (ATR) spectrum of 3,5-dihydroxy-*N*'-[(*E*)-(3-*tert*-butyl-2-hydroxyphenyl)methylidene]benzohydrazide (**4l**)

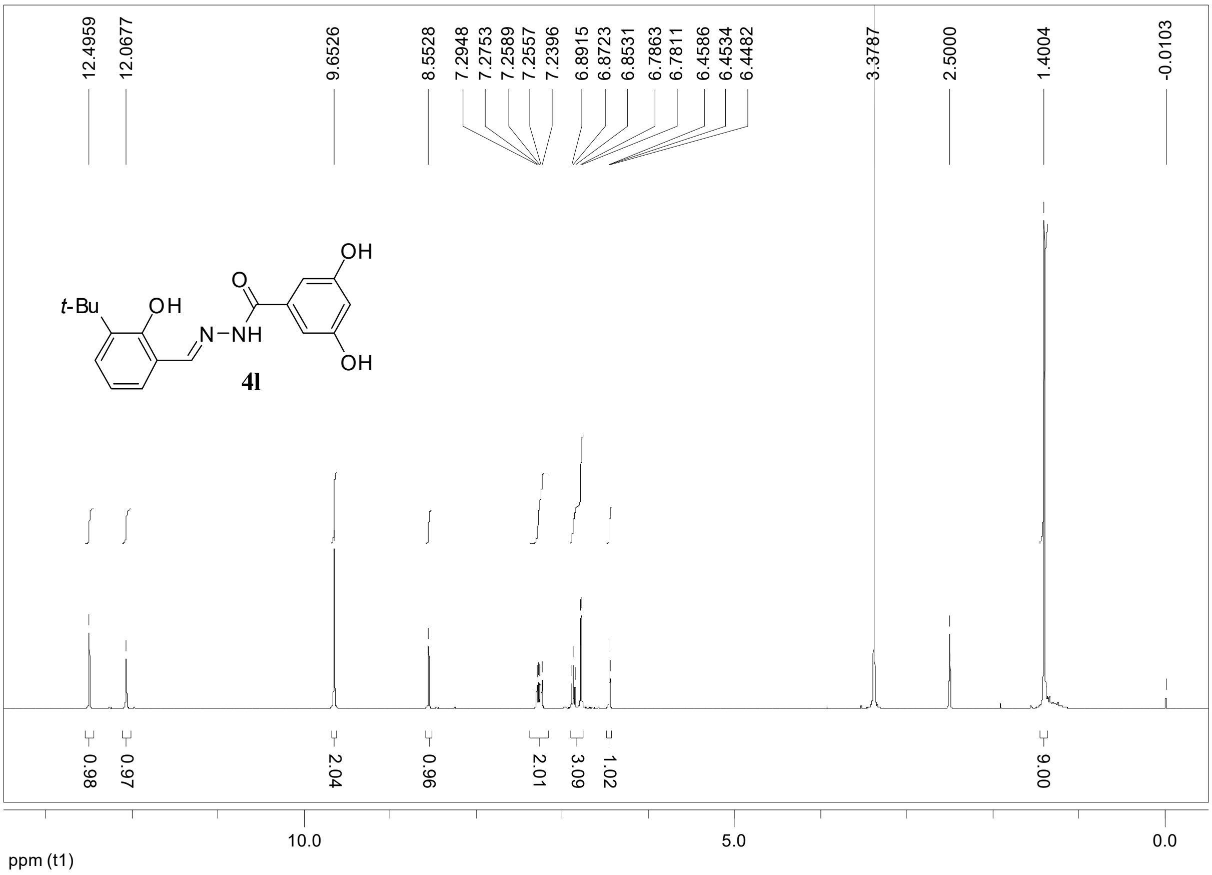

Figure S192. ^{1}H-NMR (400 MHz, DMSO-d_6) spectrum of compound **4l**

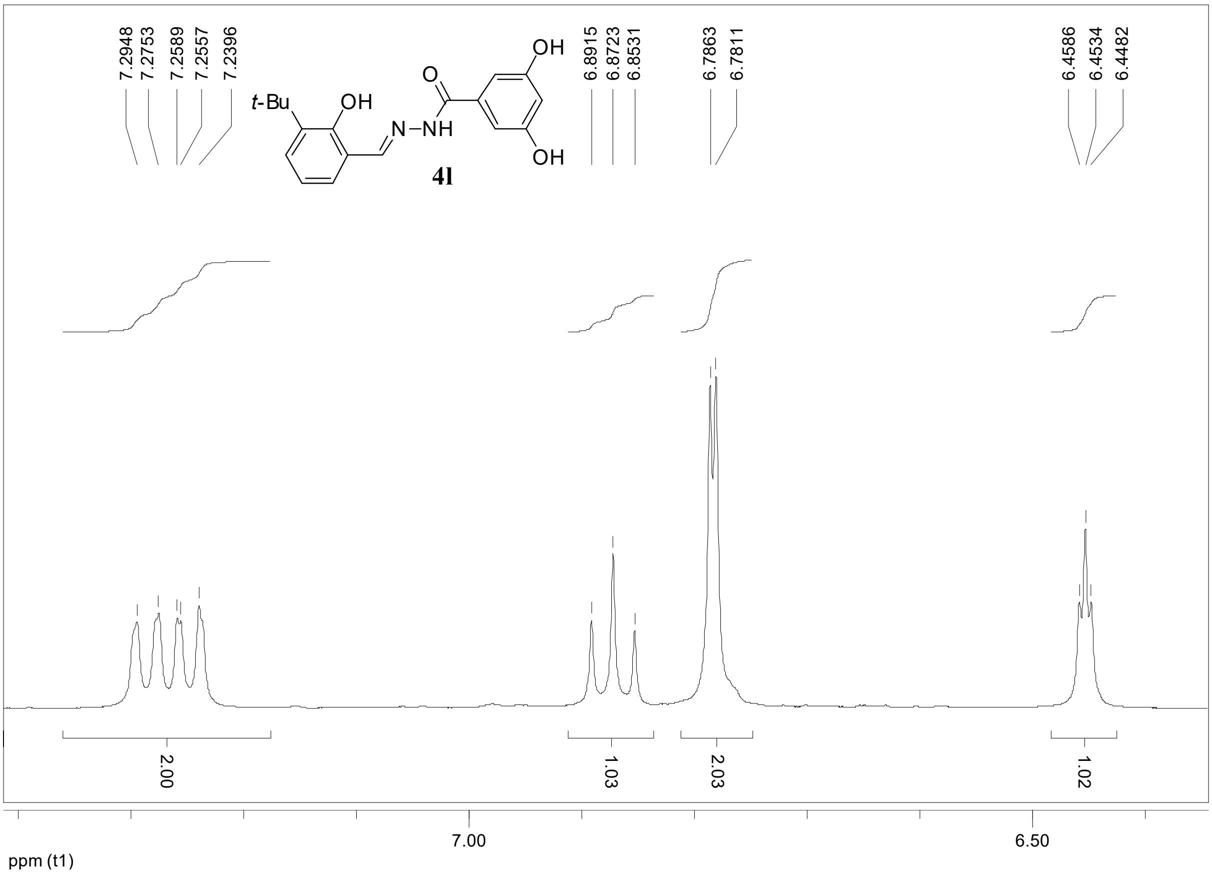

Figure S193. Expansion of ^{1}H-NMR (400 MHz, DMSO-d_6) spectrum of compound **4l**

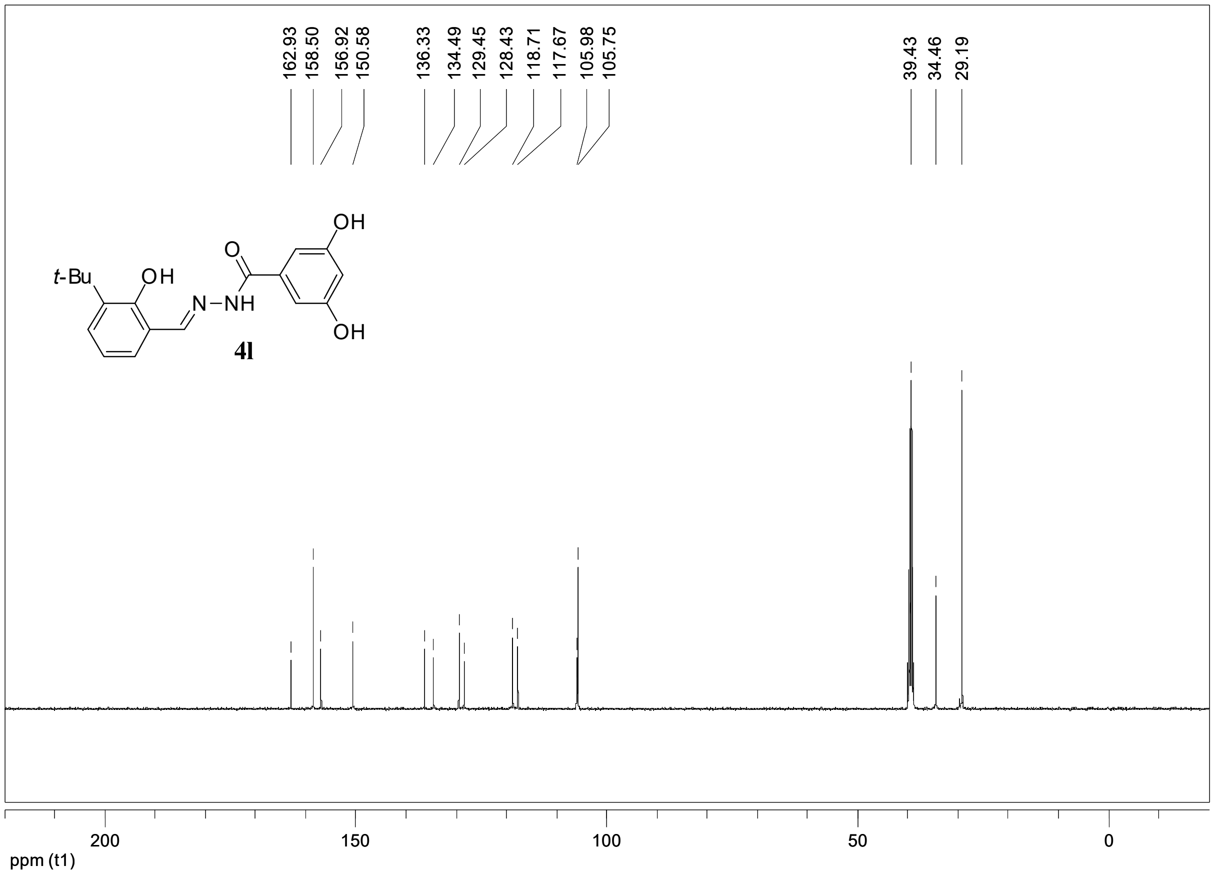

Figure S194. ^{13}C-NMR (100 MHz, DMSO-d_6) spectrum of compound **4l**

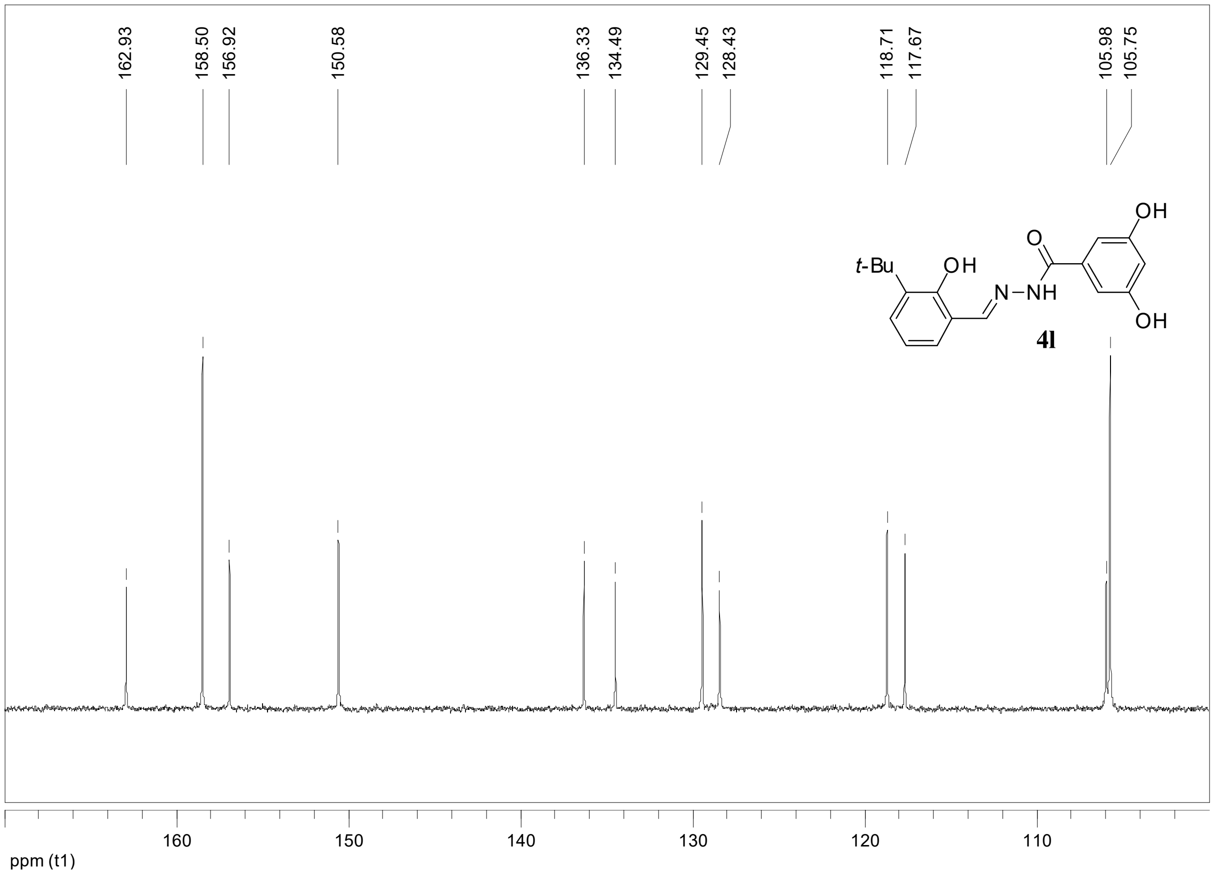

Figure S195. Expansion of ^{13}C-NMR (100 MHz, DMSO-d_6) spectrum of compound **4l**

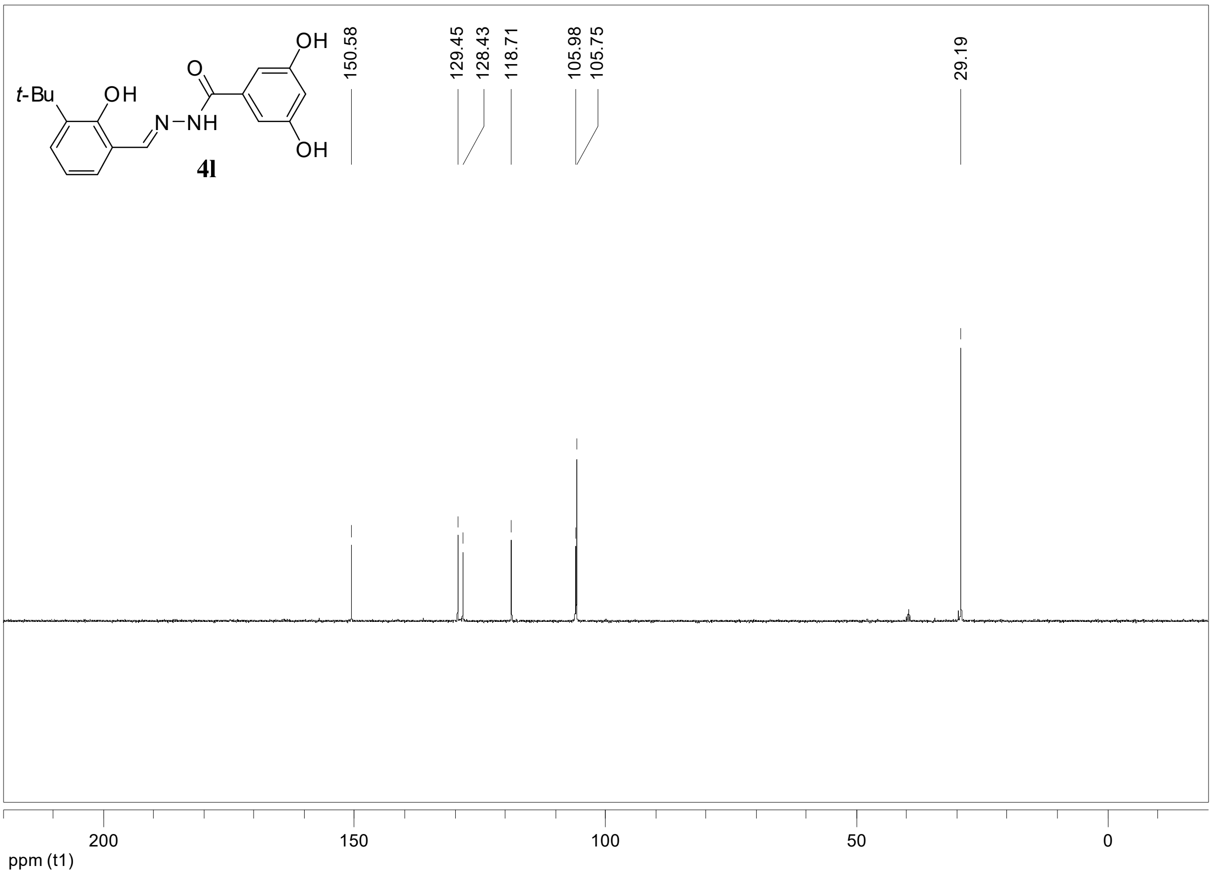

Figure S196. ^{13}C-NMR (100 MHz, DMSO-d_6) dept-135 experiment of compound **4l**

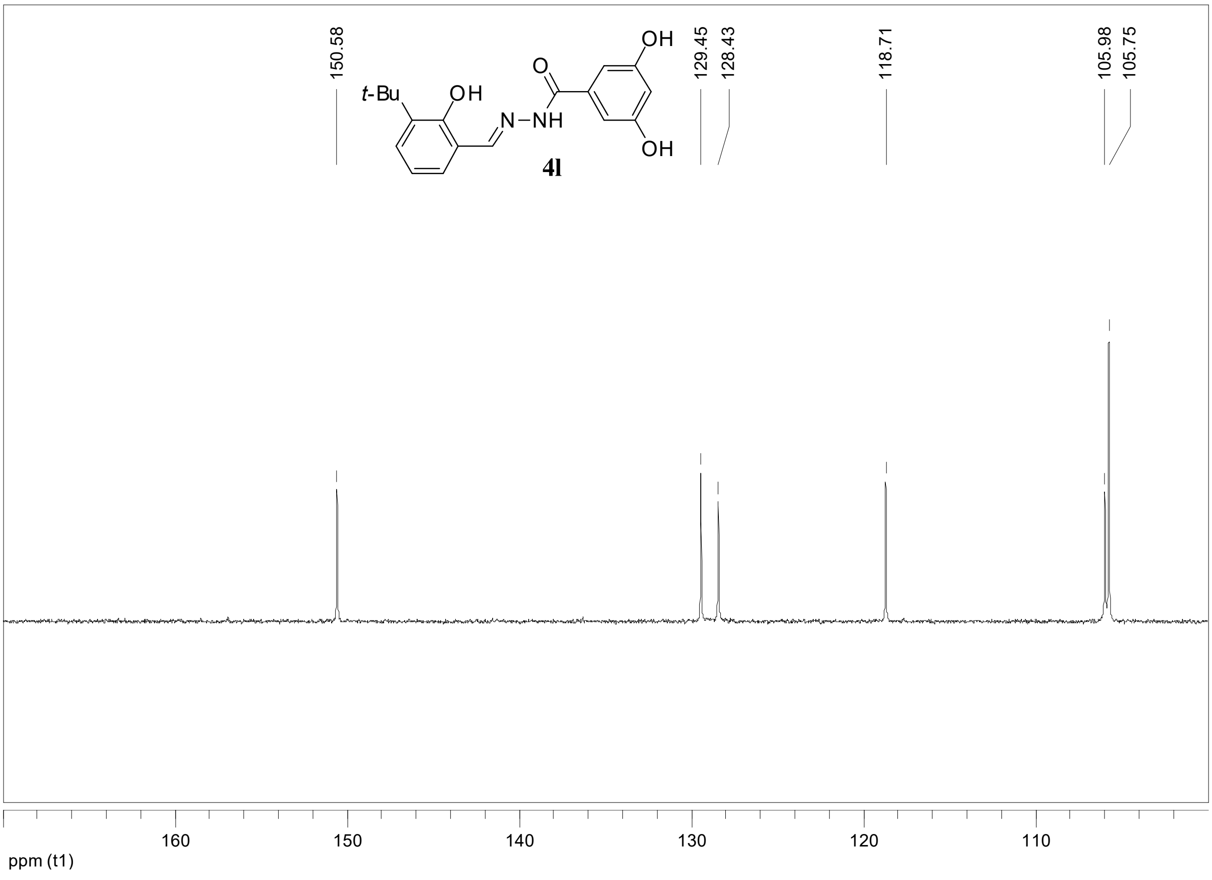

Figure S197. Expansion of ^{13}C-NMR (100 MHz, DMSO-d_6) dept-135 experiment of compound **4l**

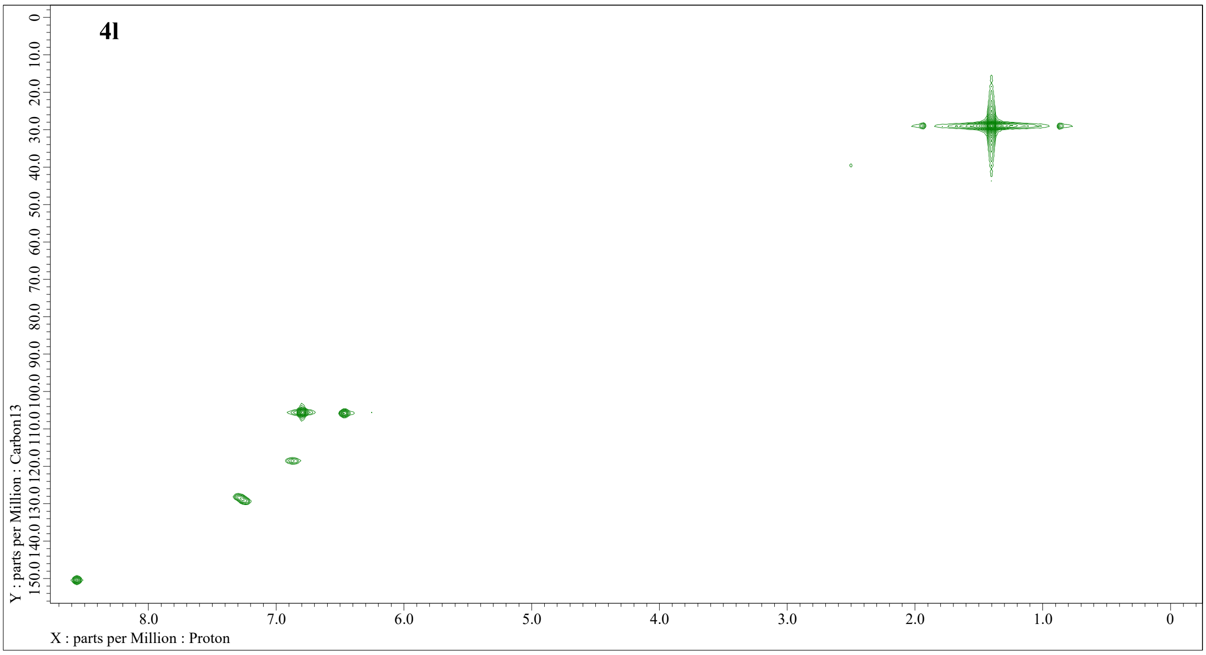

Figure S198. 2D-NMR (400 MHz, DMSO-d_6) HMQC experiment of 3,5-dihydroxy-*N*'-[(*E*)-(3-*tert*-butyl-2-hydroxyphenyl)methylidene]benzohydrazide (**4l**)

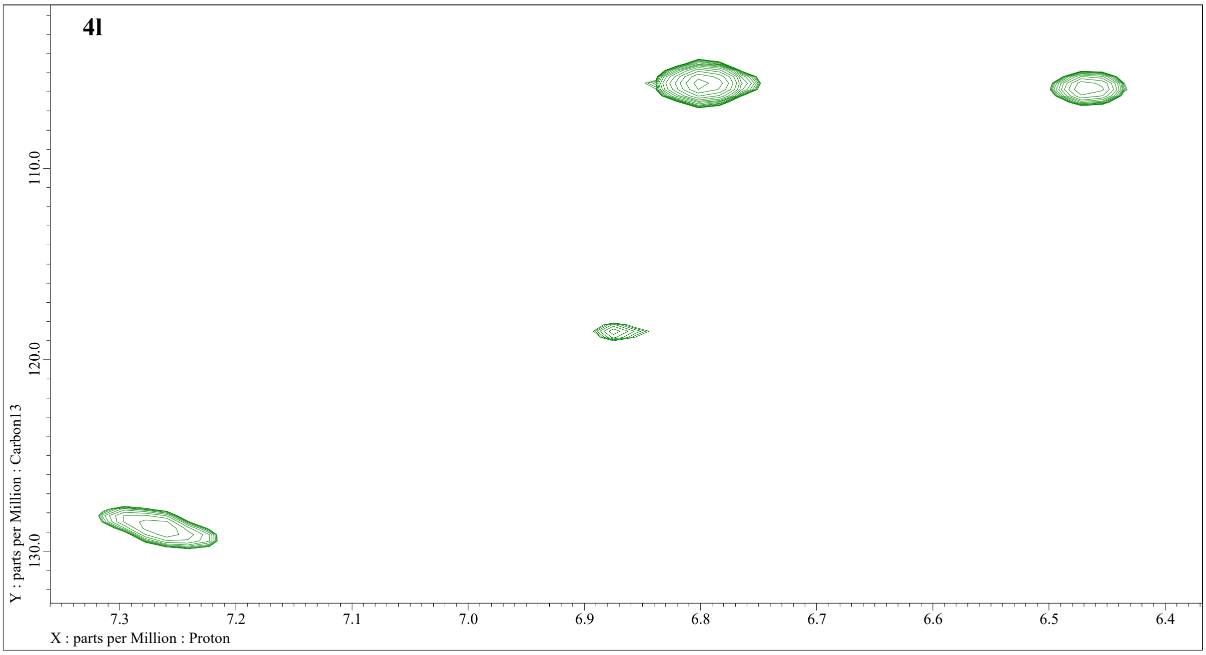

Figure S199. Expansion of 2D-NMR (400 MHz, DMSO-d_6) HMQC experiment of 3,5-dihydroxy-*N*'-[(*E*)-(3-*tert*-butyl-2-hydroxyphenyl)methylidene]benzohydrazide (**4l**)

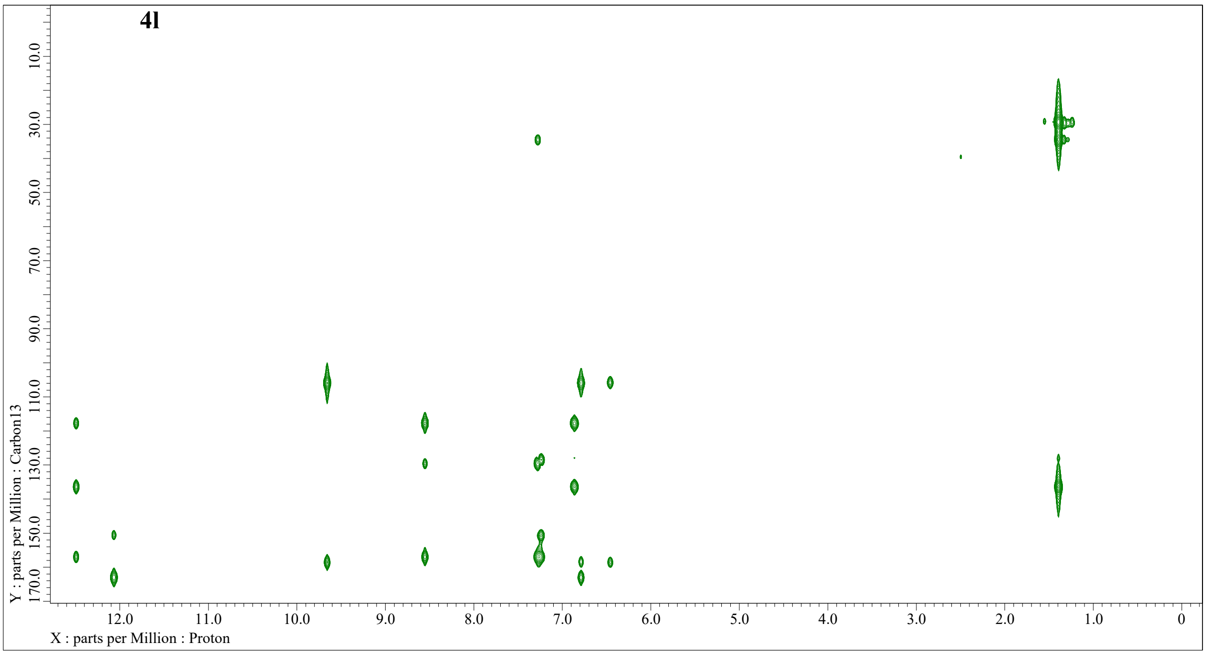

Figure S200. 2D-NMR (400 MHz, DMSO-d_6) HMBC experiment of 3,5-dihydroxy-*N*'-[(*E*)-(3-*tert*-butyl-2-hydroxyphenyl)methylidene]benzohydrazide (**4l**)

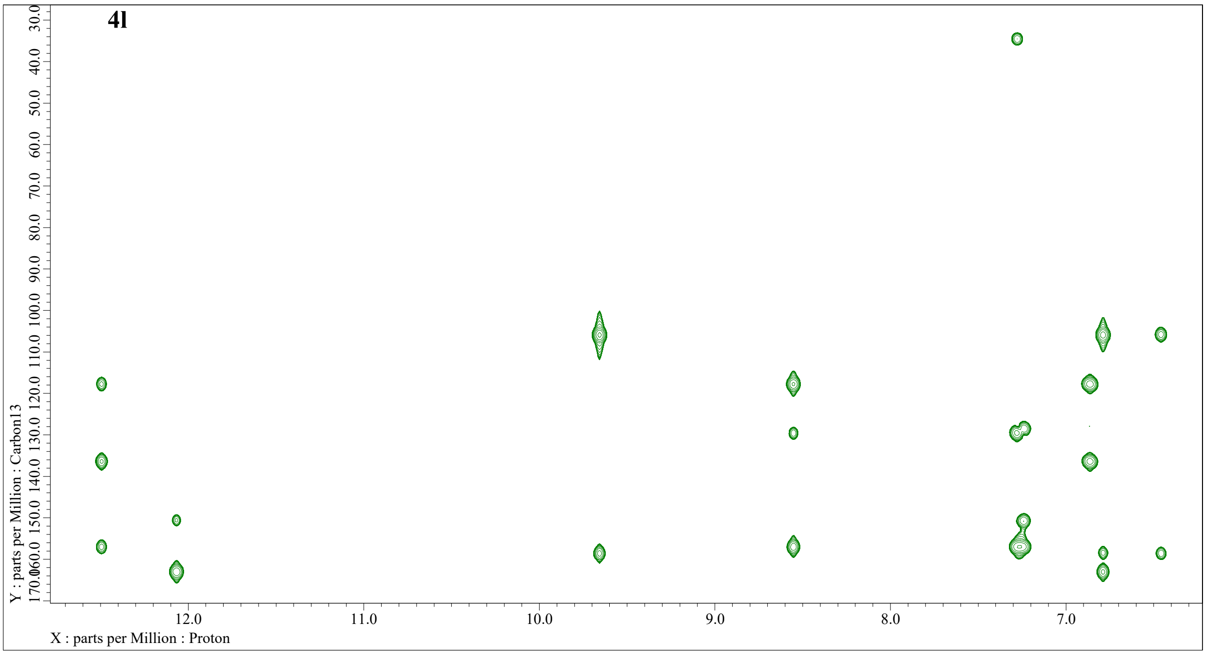

Figure S201. Expansion of 2D-NMR (400 MHz, DMSO-d_6) HMBC experiment of 3,5-dihydroxy-*N*'-[(*E*)-(3-*tert*-butyl-2-hydroxyphenyl)methylidene]benzohydrazide (**4l**)

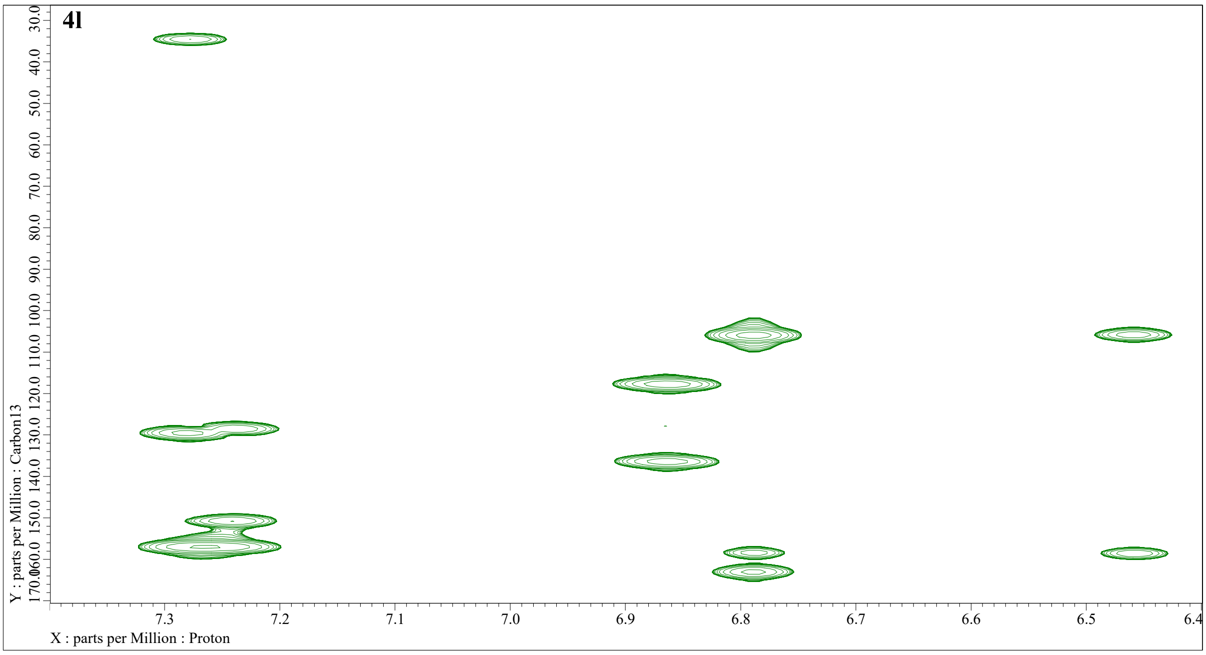

Figure S202. Expansion of 2D-NMR (400 MHz, DMSO-d_6) HMBC experiment of 3,5-dihydroxy-*N*'-[(*E*)-(3-*tert*-butyl-2-hydroxyphenyl)methylidene]benzohydrazide (**4l**)

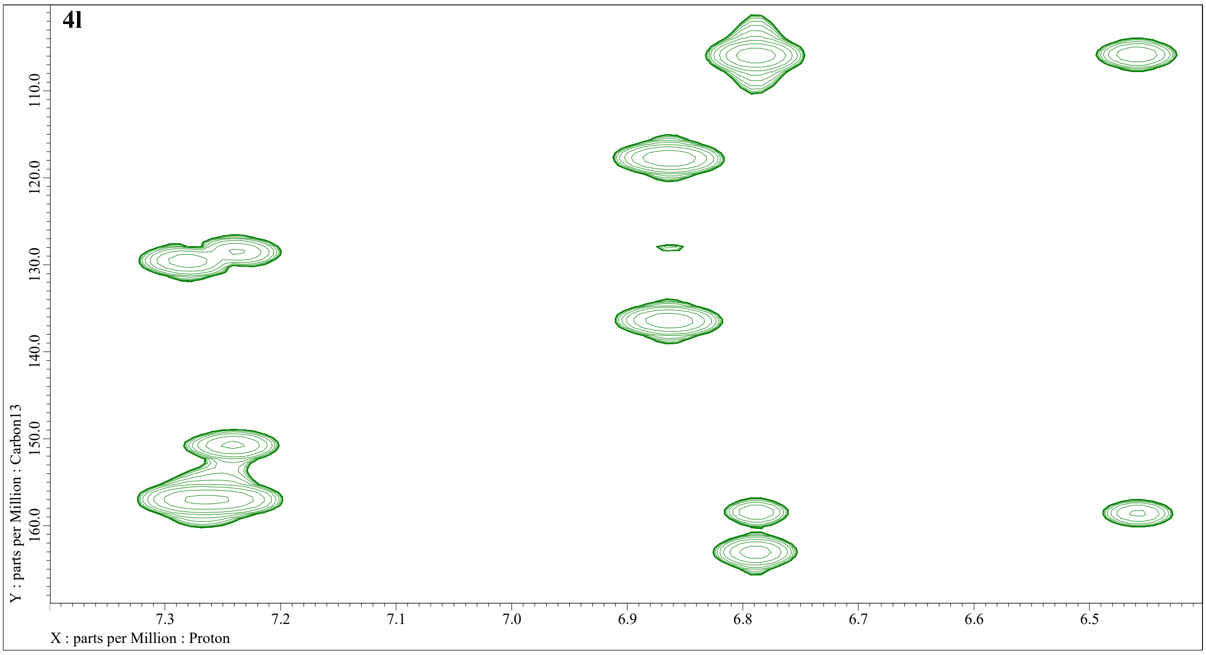

Figure S203. Expansion of 2D-NMR (400 MHz, DMSO-d_6) HMBC experiment of 3,5-dihydroxy-*N*'-[(*E*)-(3-*tert*-butyl-2-hydroxyphenyl)methylidene]benzohydrazide (**4l**)

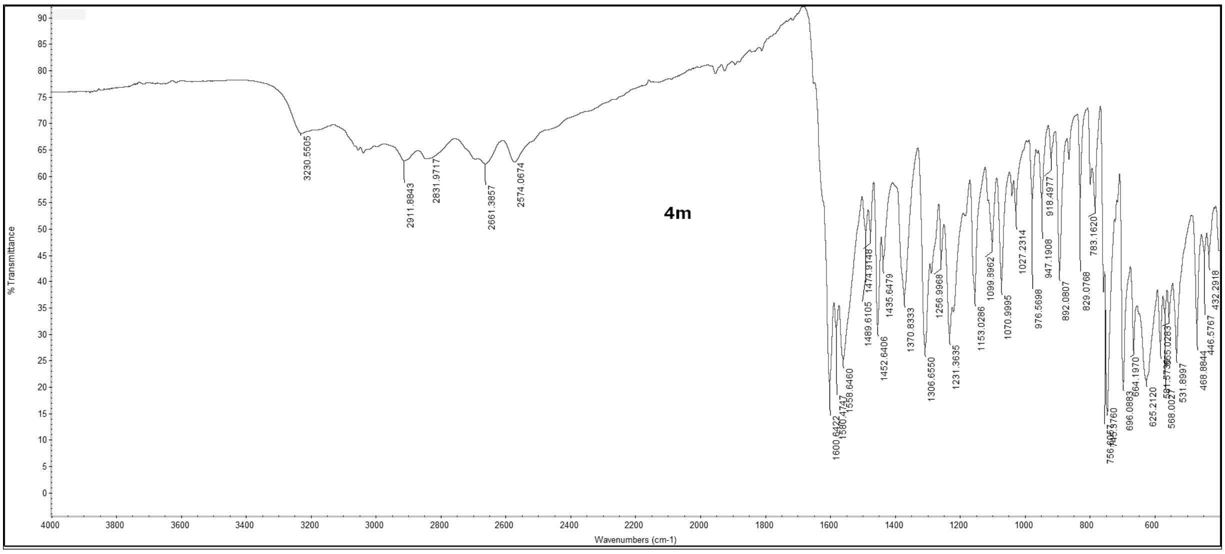

Figure S204. FT-IR (ATR) spectrum of 2-hydroxy-*N*'-[(*E*)-(2-hydroxy-3-phenyl-phenyl)methylidene]benzohydrazide (**4m**)

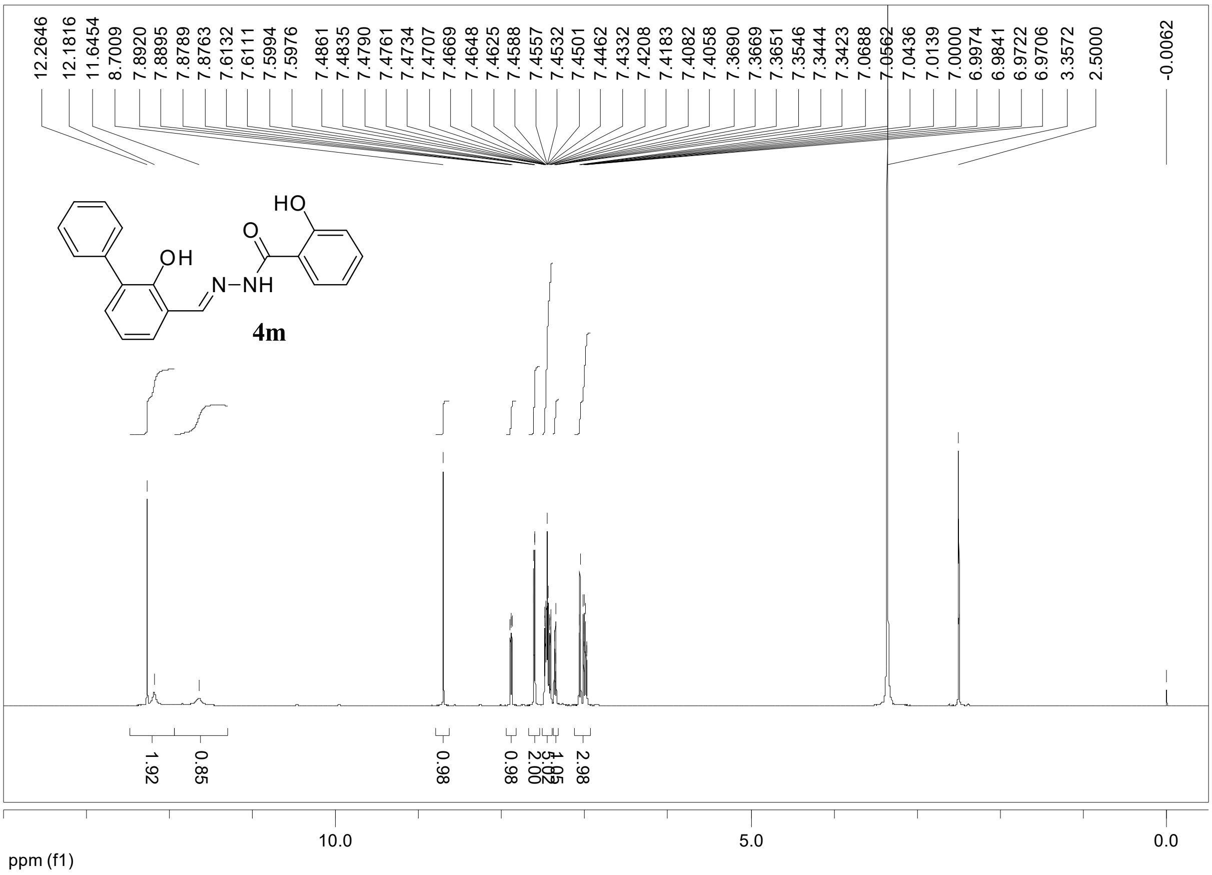

Figure S205. ^{1}H-NMR (600 MHz, DMSO-d_6) spectrum of compound **4m**

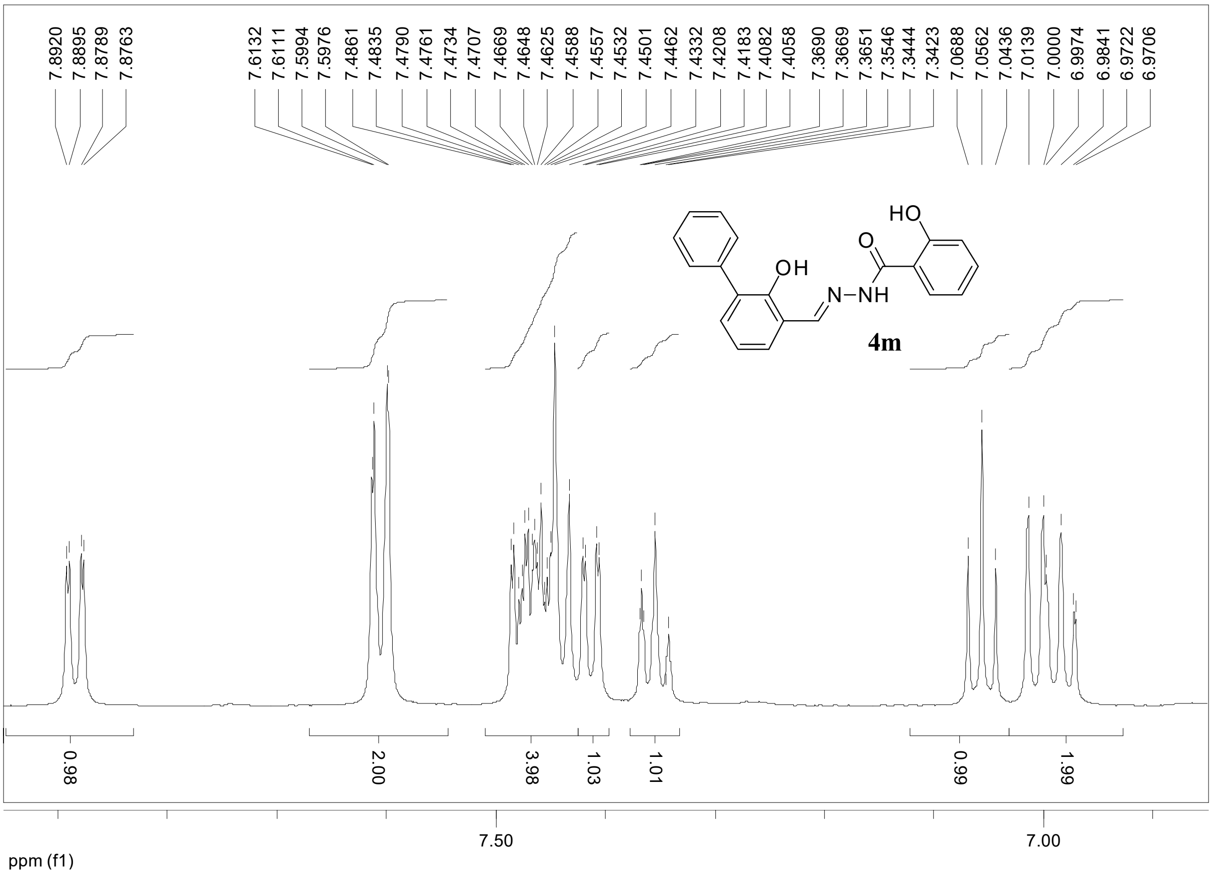

Figure S206. Expansion of ^{1}H-NMR (600 MHz, DMSO-d_6) spectrum of compound **4m**

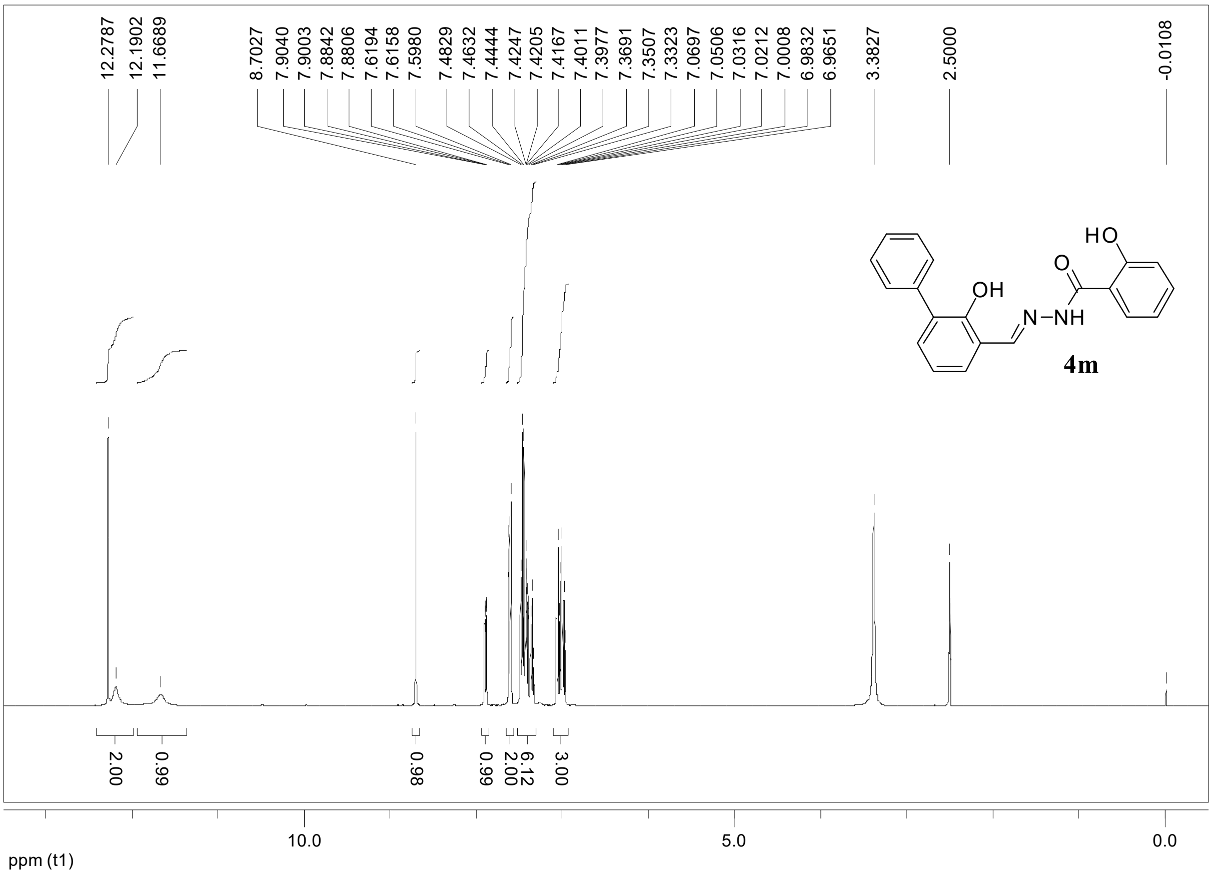

Figure S207. ^{1}H-NMR (400 MHz, DMSO-d_6) spectrum of compound **4m**

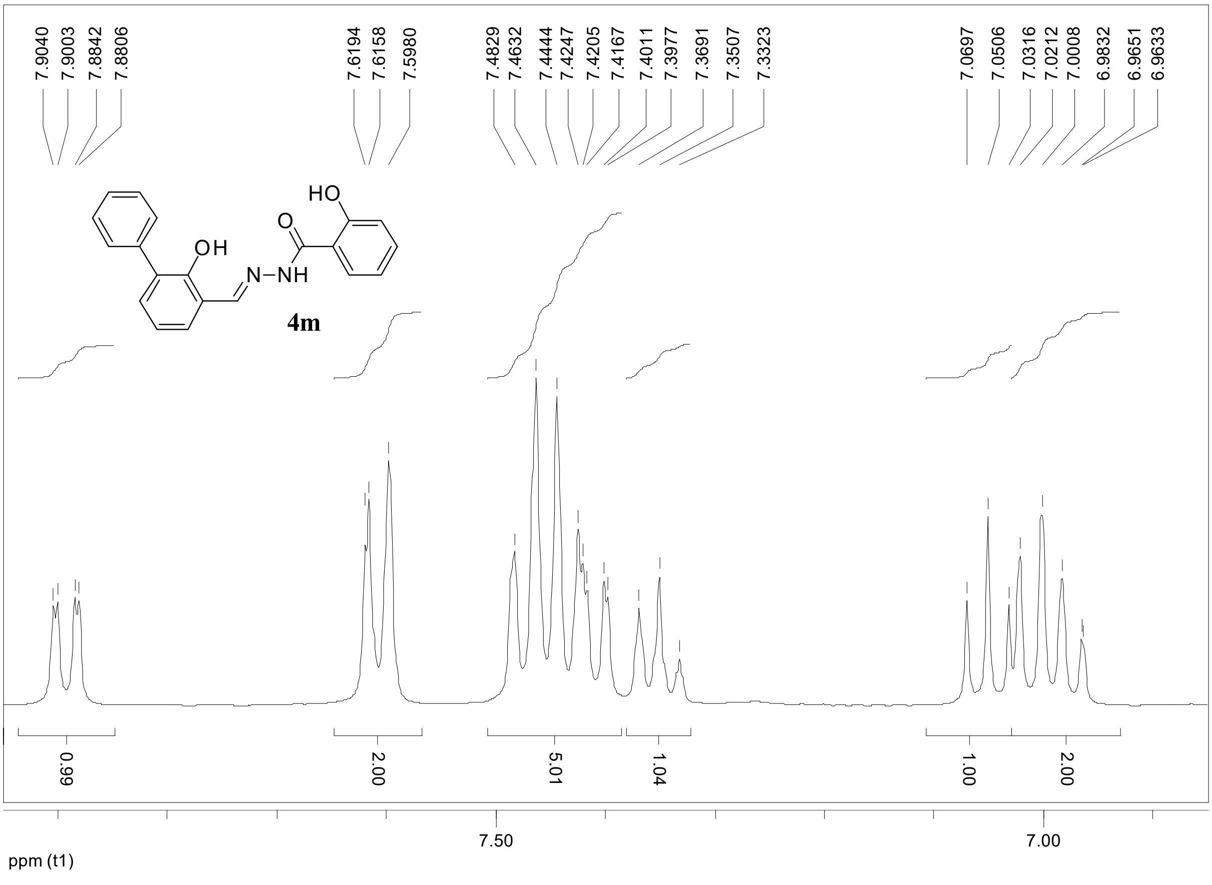

Figure S208. Expansion of ^{1}H-NMR (400 MHz, DMSO-d_6) spectrum of compound **4m**

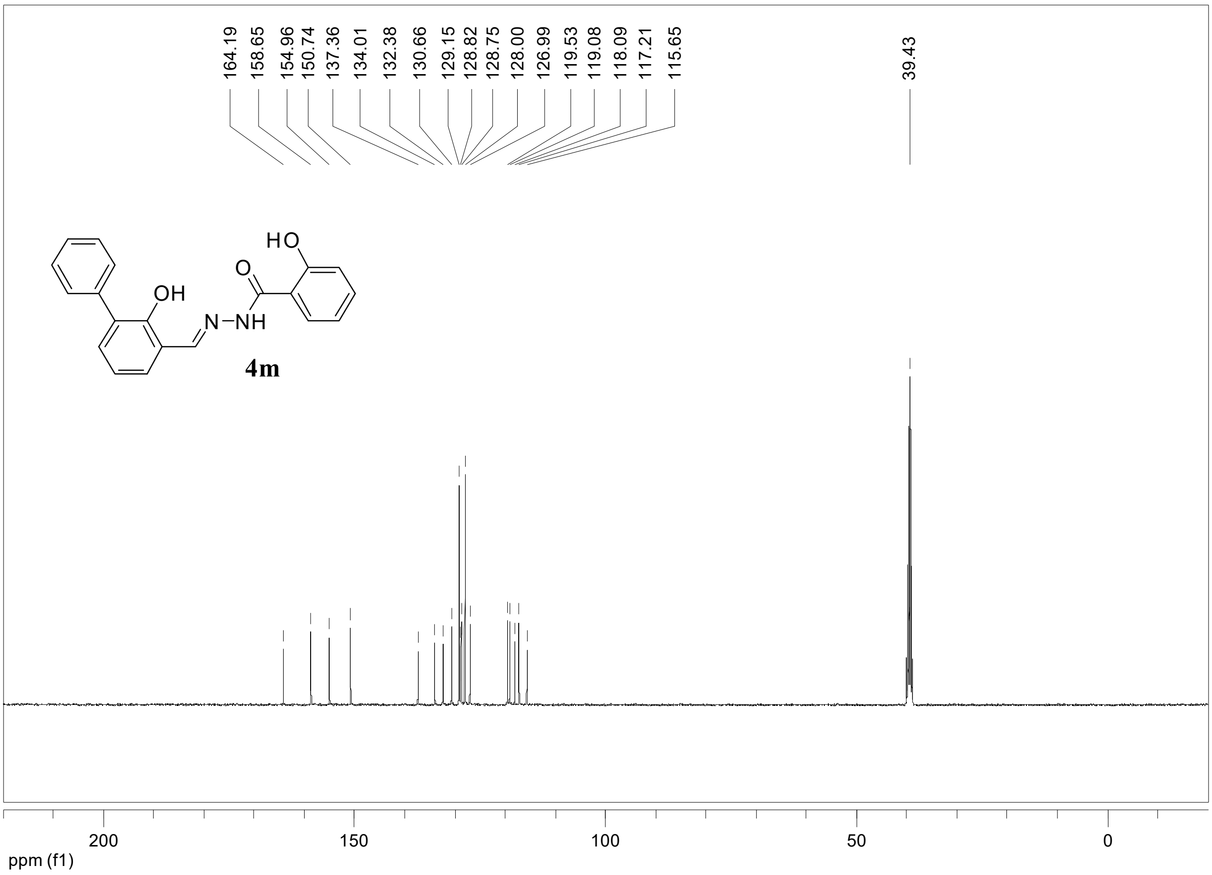

Figure S209. ^{13}C-NMR (100 MHz, DMSO-d_6) spectrum of compound **4m**

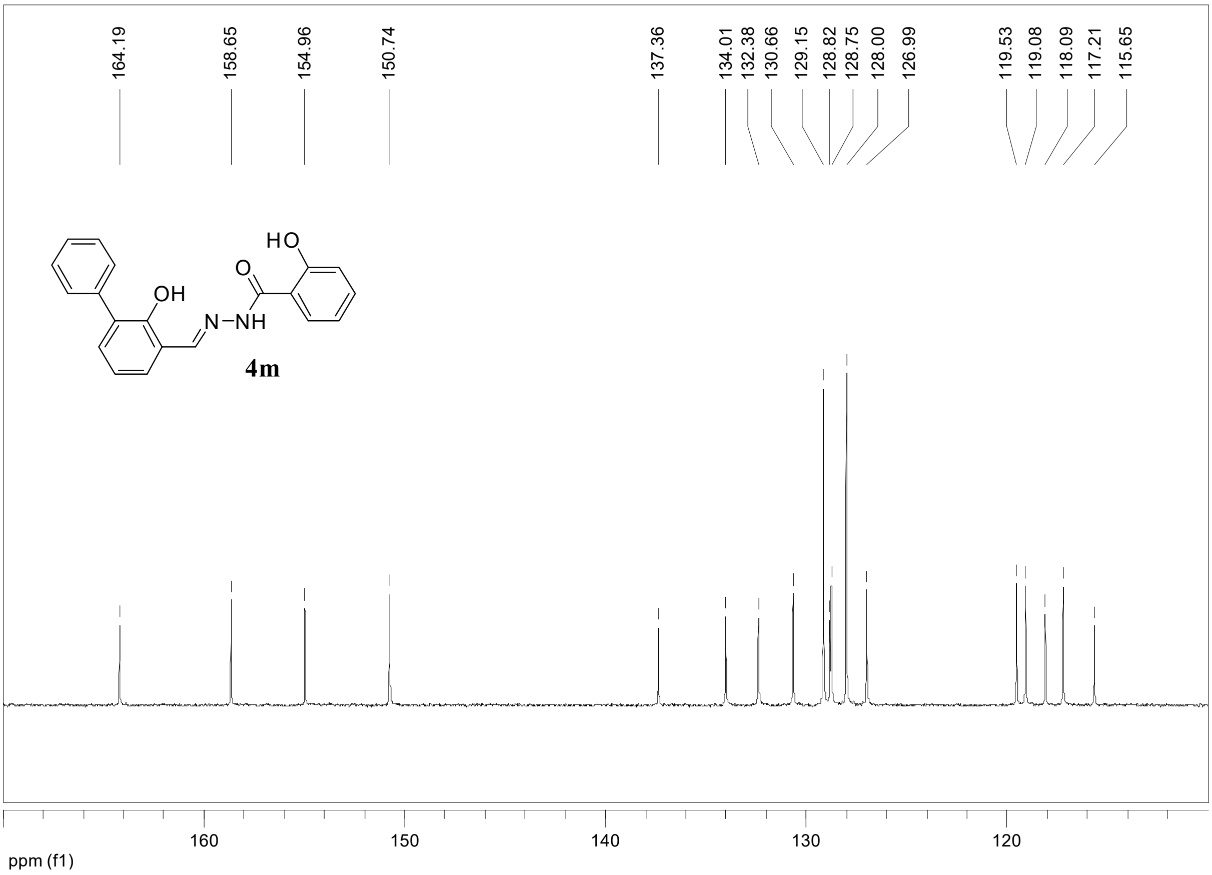

Figure S210. Expansion of ^{13}C-NMR (100 MHz, DMSO-d_6) spectrum of compound **4m**

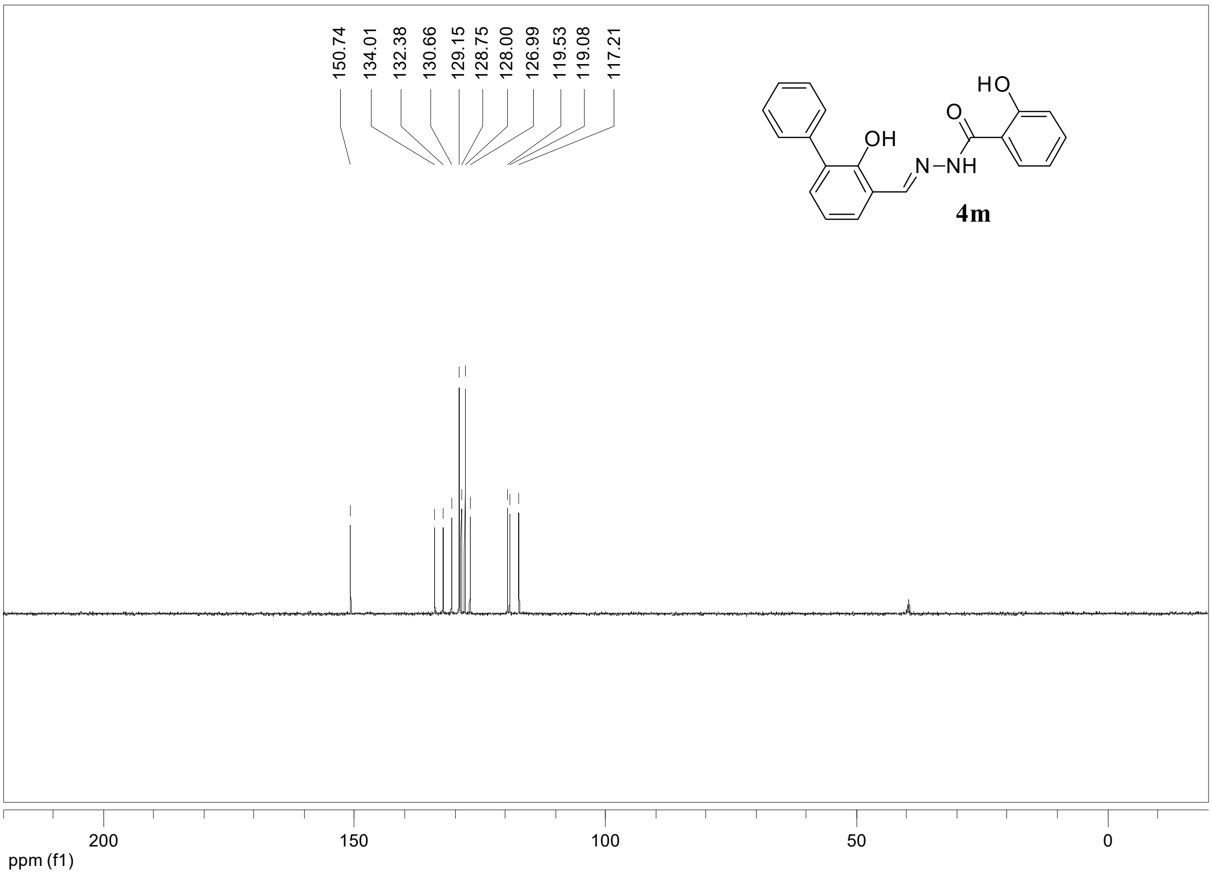

Figure S211. ^{13}C-NMR (100 MHz, DMSO-d_6) dept-135 experiment of compound **4m**

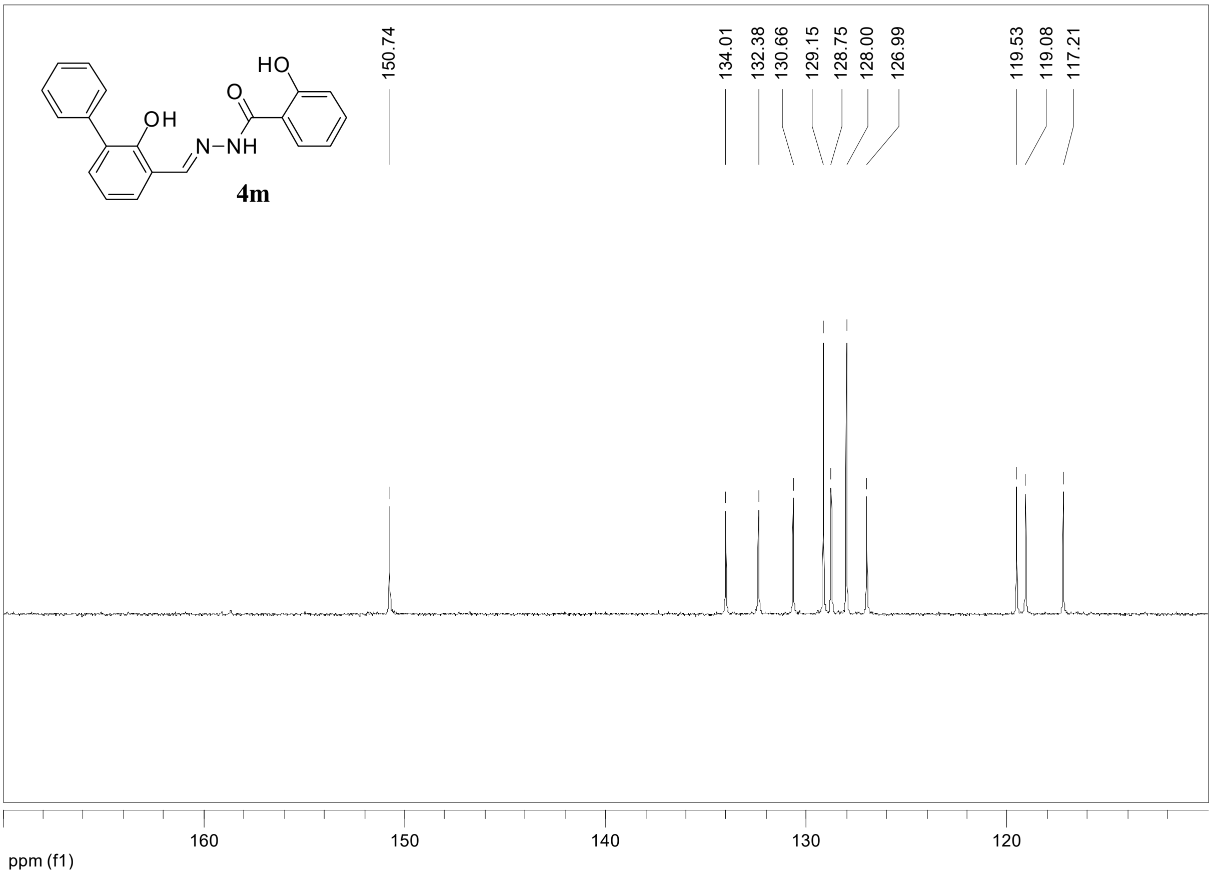

Figure S212. Expansion of ^{13}C-NMR (100 MHz, DMSO-d_6) dept-135 experiment of compound **4m**

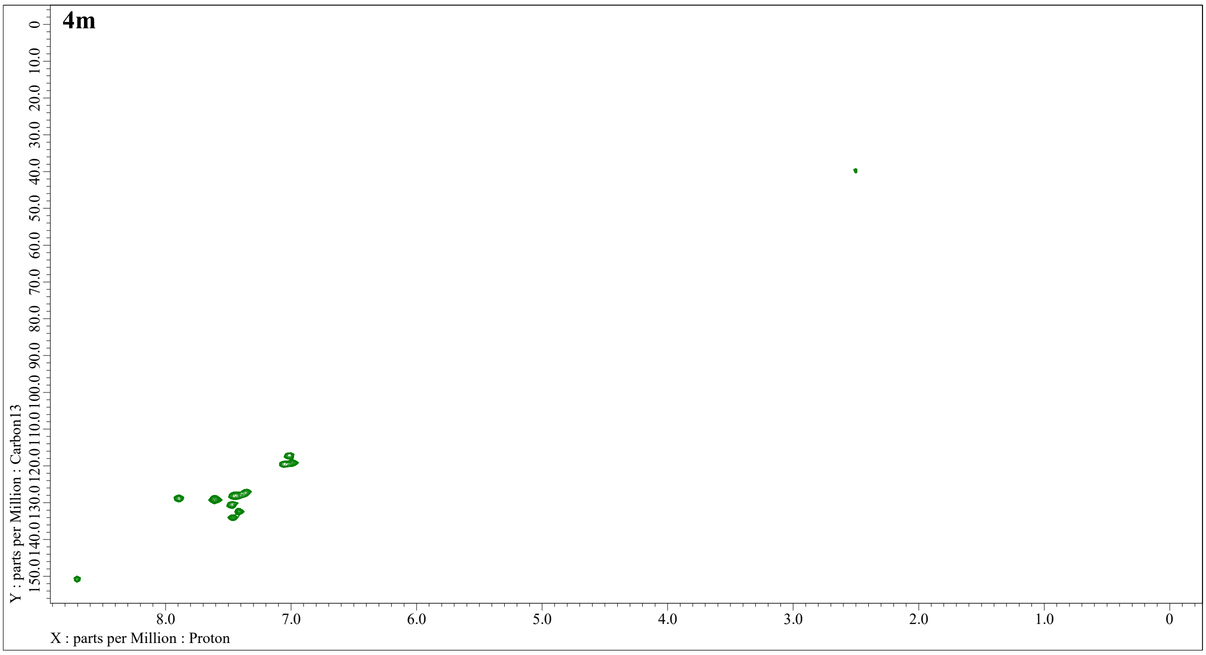

Figure S213. 2D-NMR (400 MHz, DMSO-d_6) HSQC experiment of 2-hydroxy-*N*'-[(*E*)-(2-hydroxy-3-phenyl-phenyl)methylidene]benzohydrazide (**4m**)

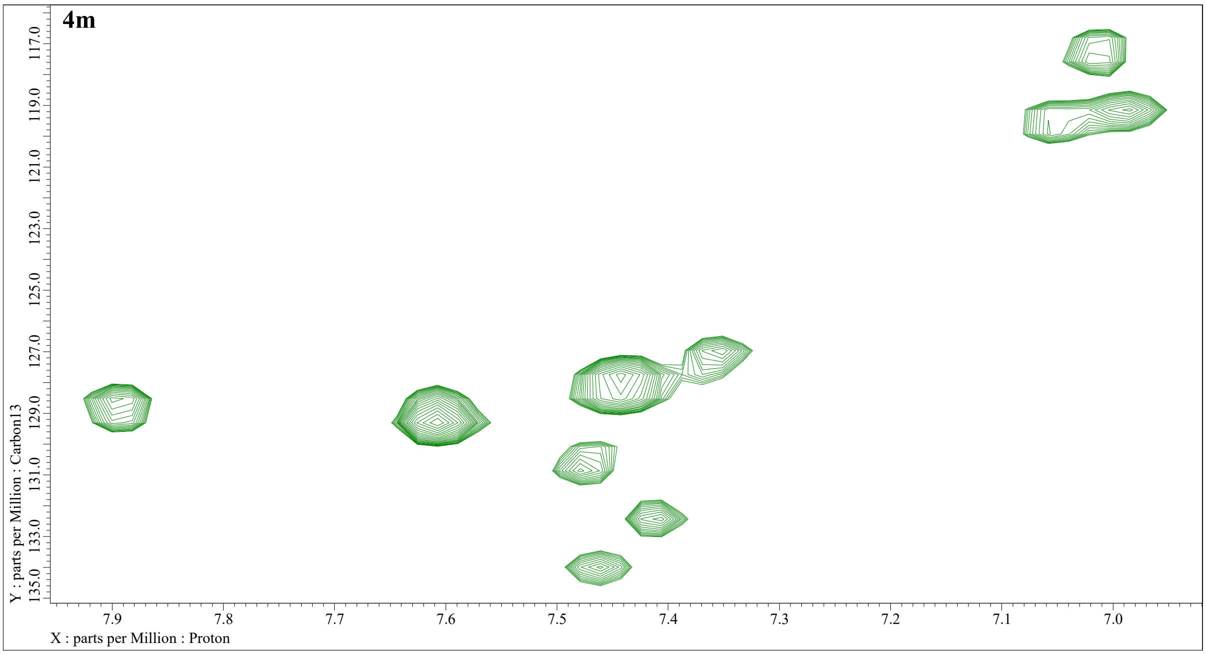

Figure S214. Expansion of 2D-NMR (400 MHz, DMSO-d_6) HSQC experiment of 2-hydroxy-*N*'-[(*E*)-(2-hydroxy-3-phenyl-phenyl)methylidene]benzohydrazide (**4m**)

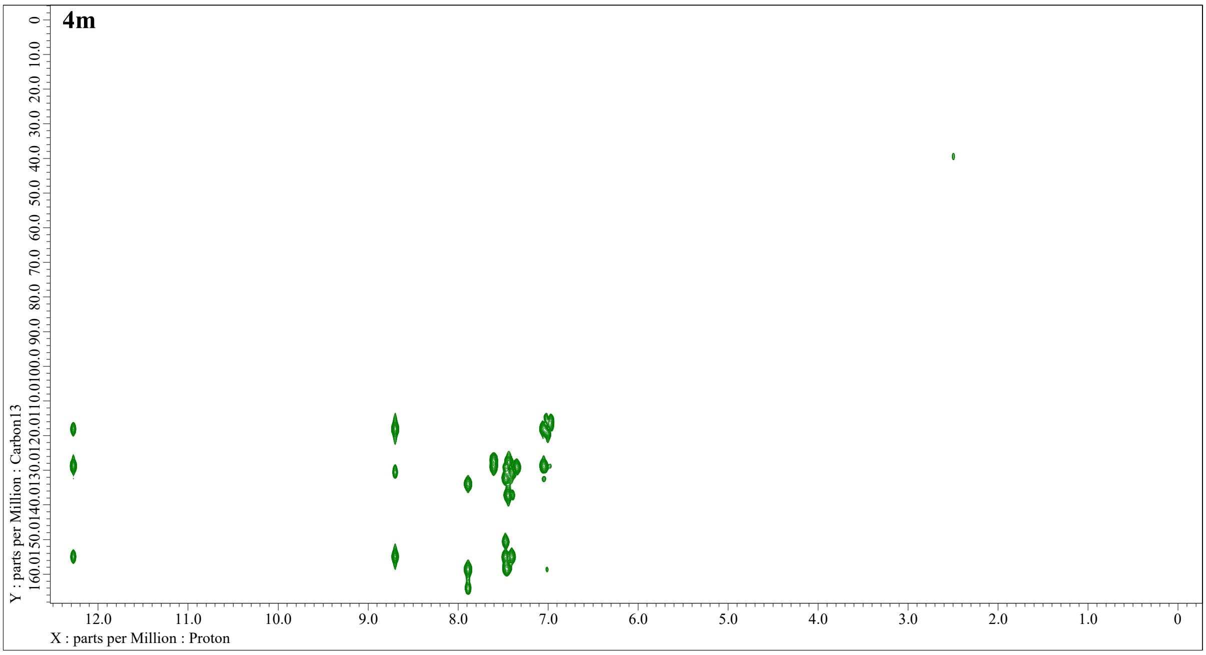

Figure S215. 2D-NMR (400 MHz, DMSO-d_6) HMBC experiment of 2-hydroxy-*N*'-[(*E*)-(2-hydroxy-3-phenyl-phenyl)methylidene]benzohydrazide (**4m**)

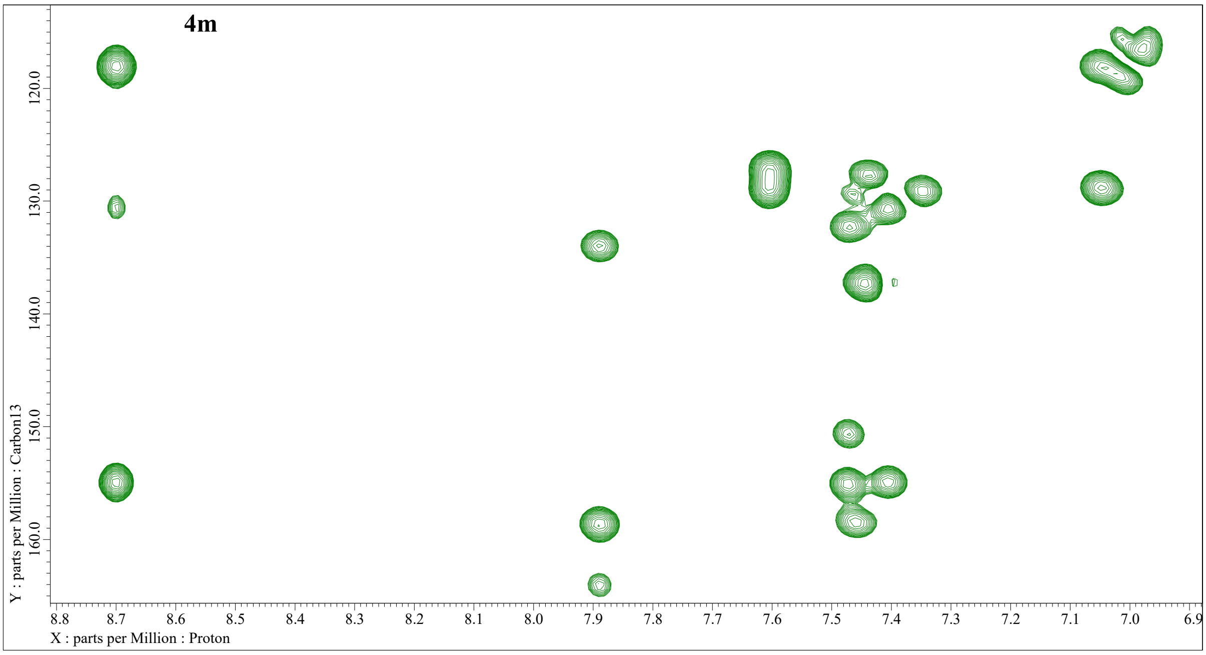

Figure S216. Expansion of 2D-NMR (400 MHz, DMSO-d_6) HMBC experiment of 2-hydroxy-*N*'-[(*E*)-(2-hydroxy-3-phenyl-phenyl)methylidene]benzohydrazide (**4m**)

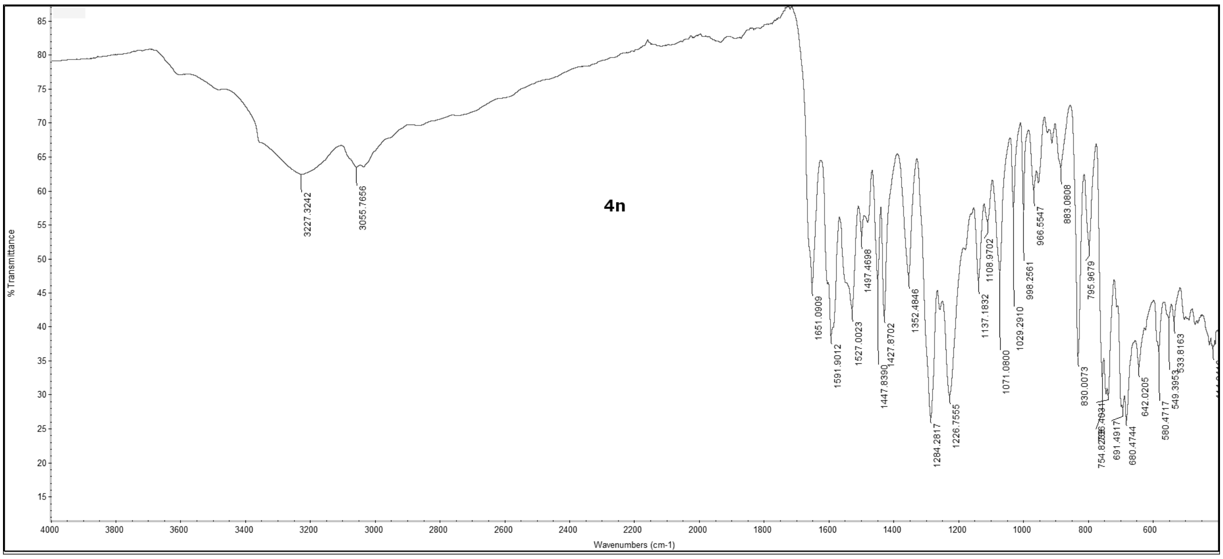

Figure S217. FT-IR (ATR) spectrum of 3-hydroxy-*N*'-[(*E*)-(2-hydroxy-3-phenyl-phenyl)methylidene]benzohydrazide (**4n**)

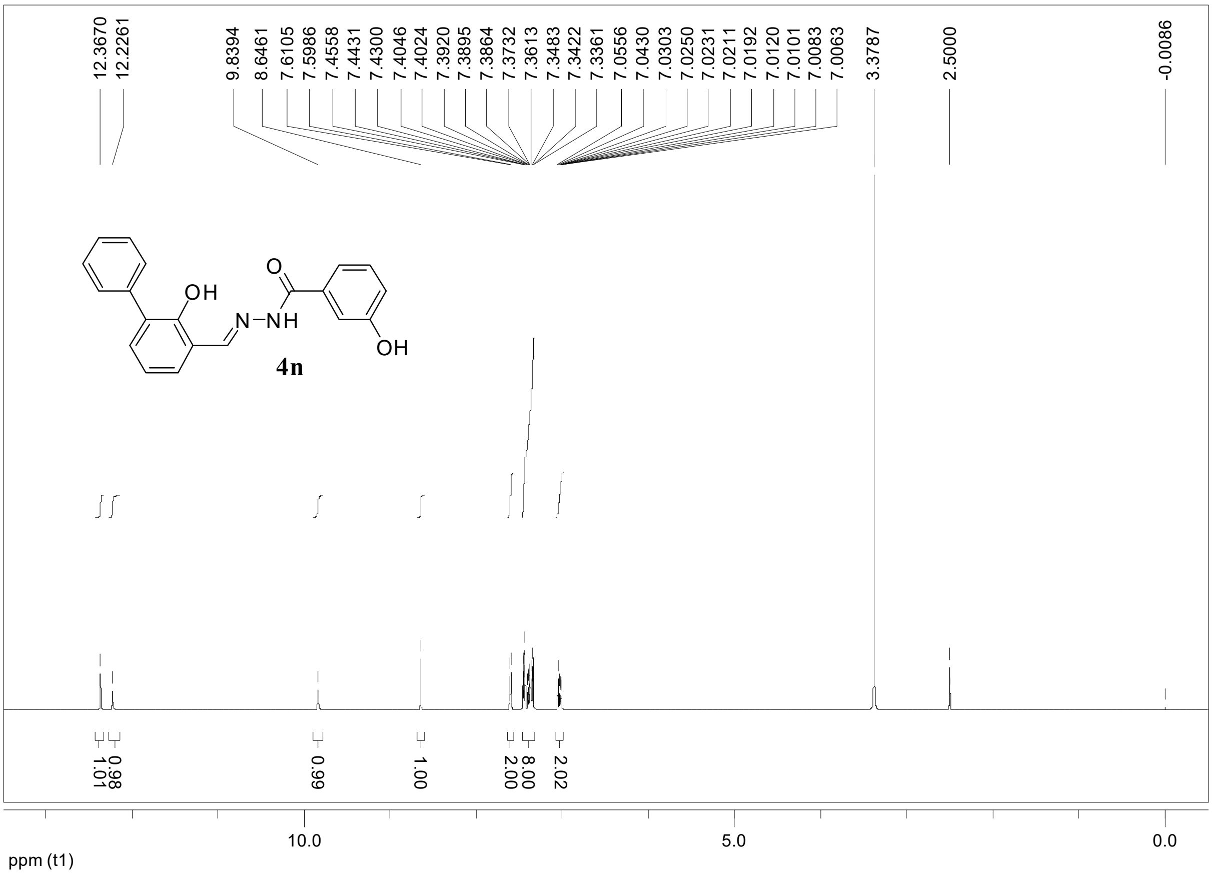

Figure S218. ^{1}H-NMR (600 MHz, DMSO-d_6) spectrum of compound **4n**

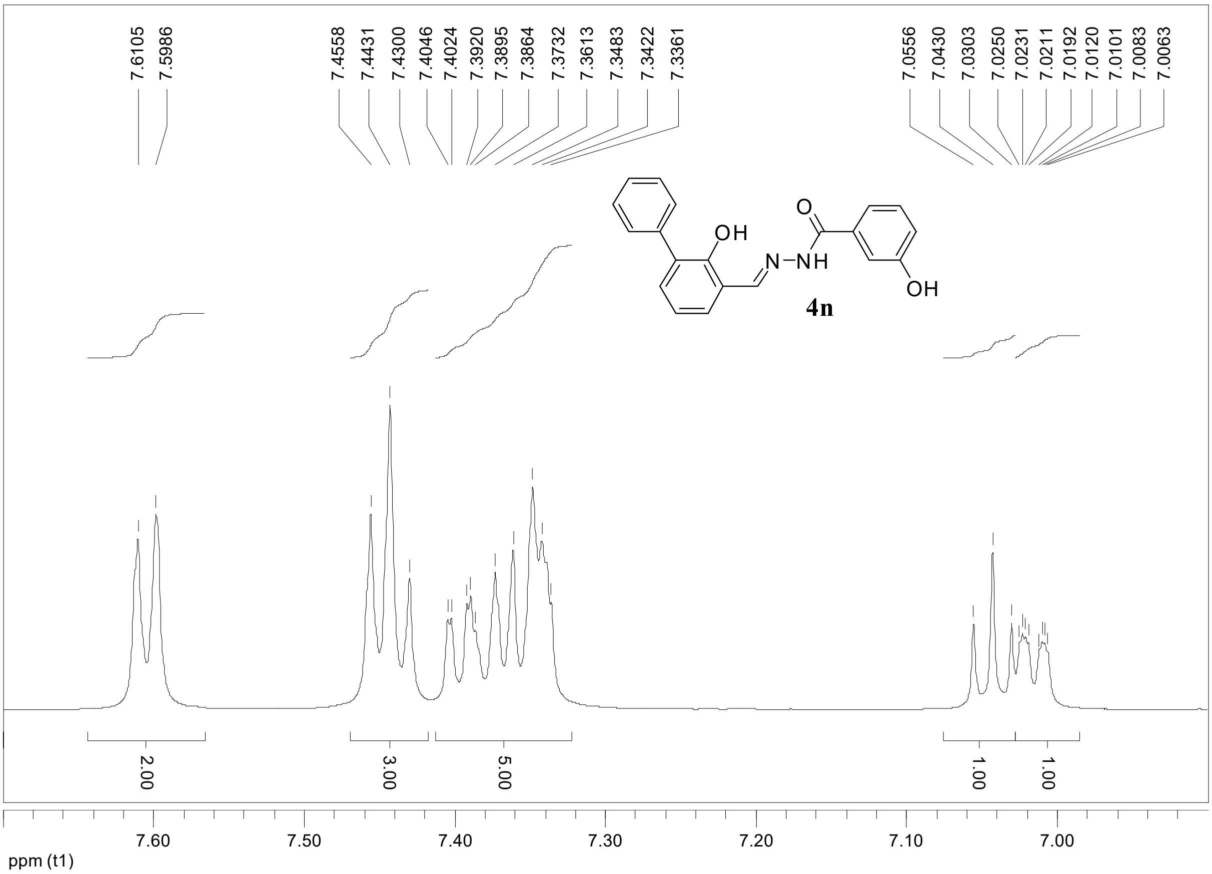

Figure S219. Expansion of ^{1}H-NMR (600 MHz, DMSO-d_6) spectrum of compound **4n**

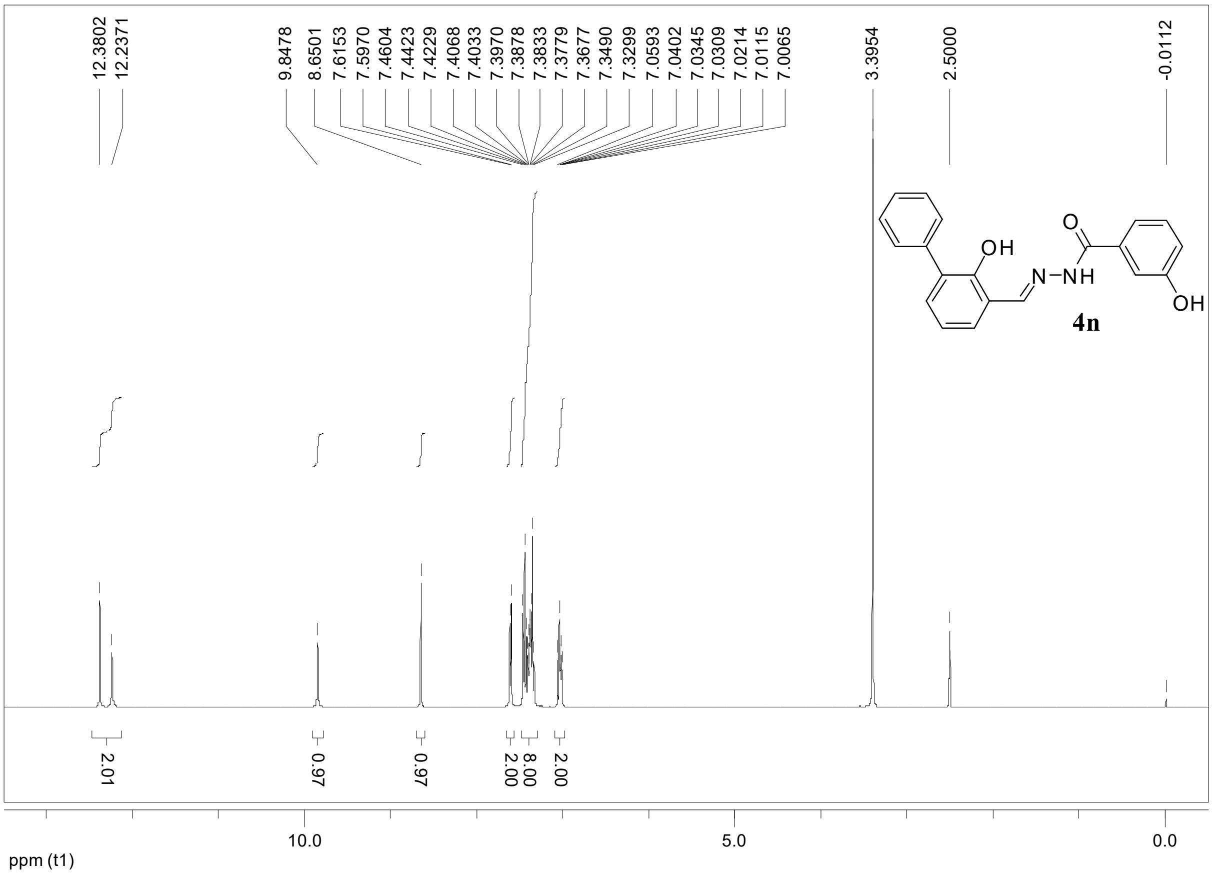

Figure S220. ^{1}H-NMR (400 MHz, DMSO-d_6) spectrum of compound **4n**

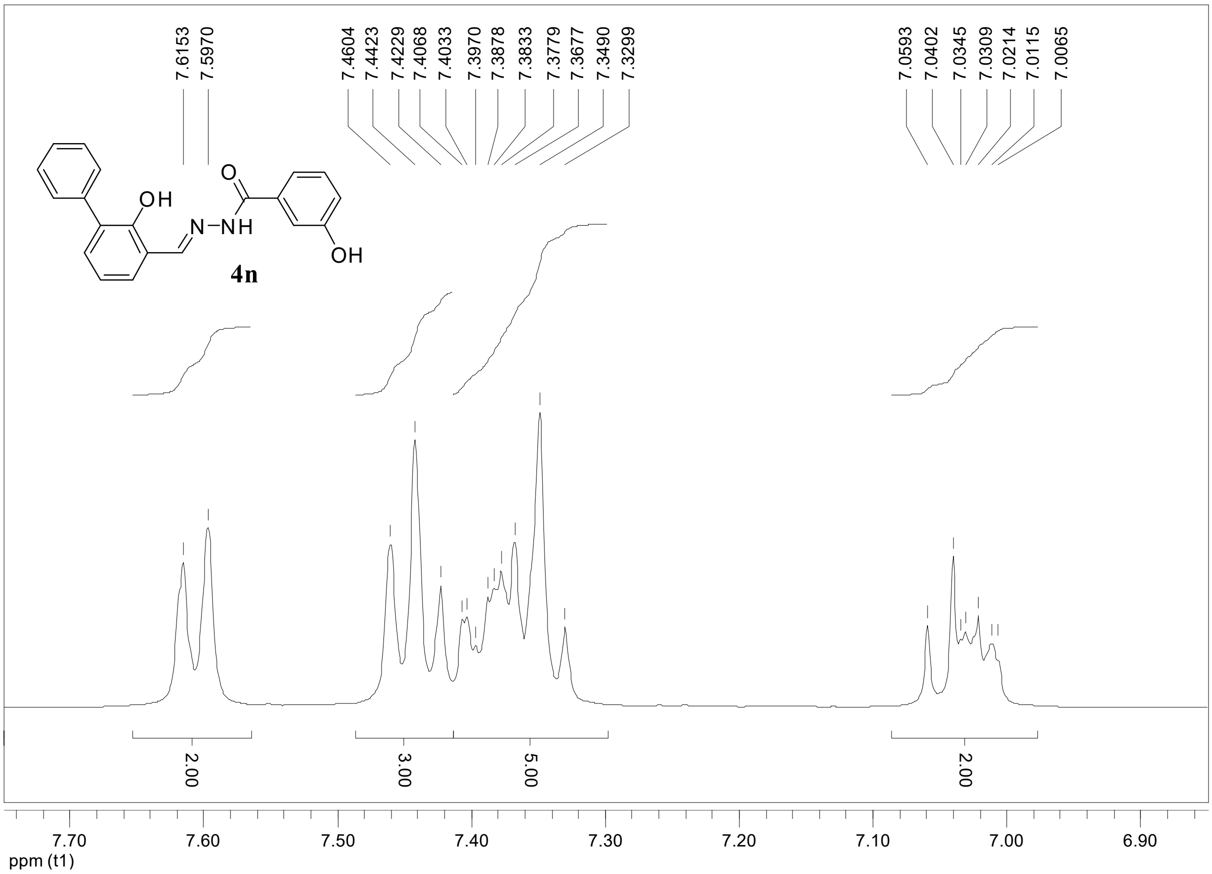

Figure S221. Expansion of ^{1}H-NMR (400 MHz, DMSO-d_6) spectrum of compound **4n**

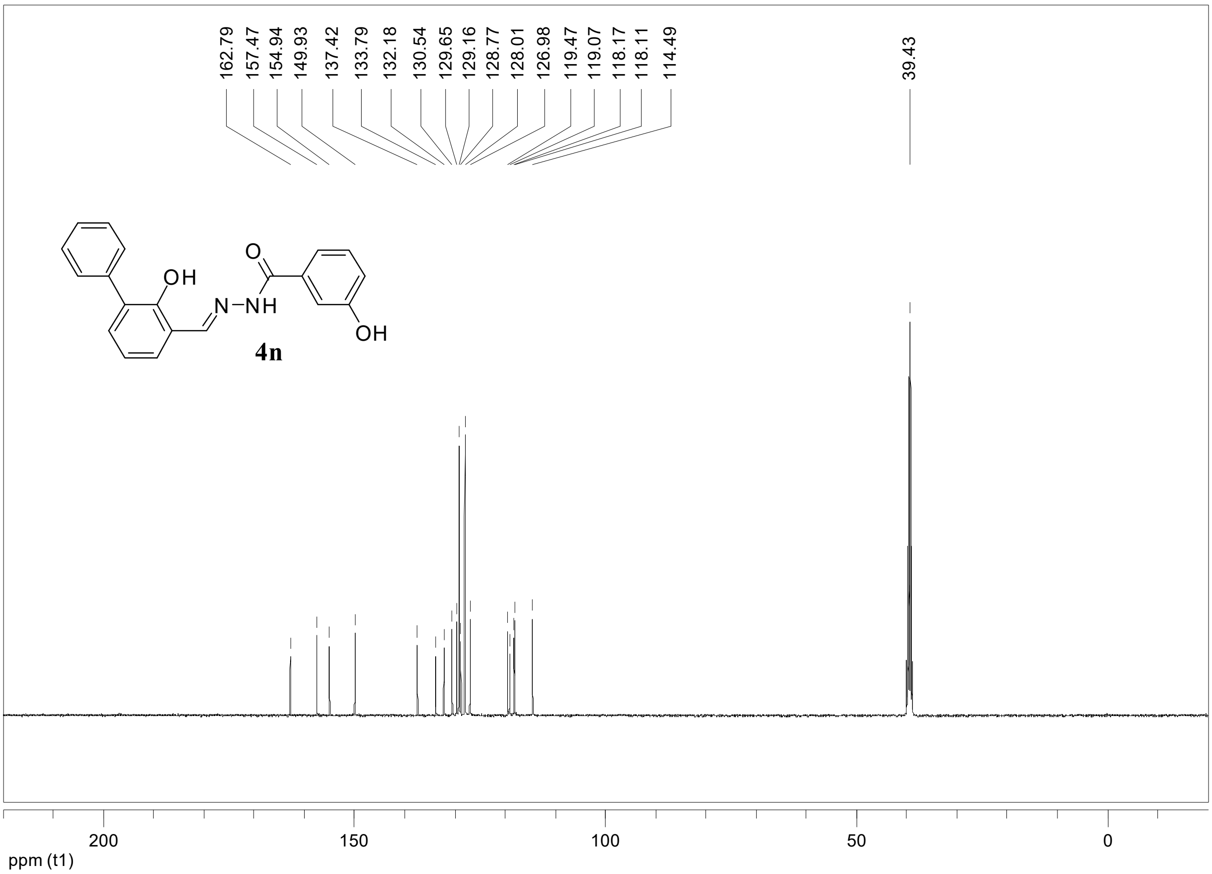

Figure S222. ^{13}C-NMR (100 MHz, DMSO-d_6) spectrum of compound **4n**

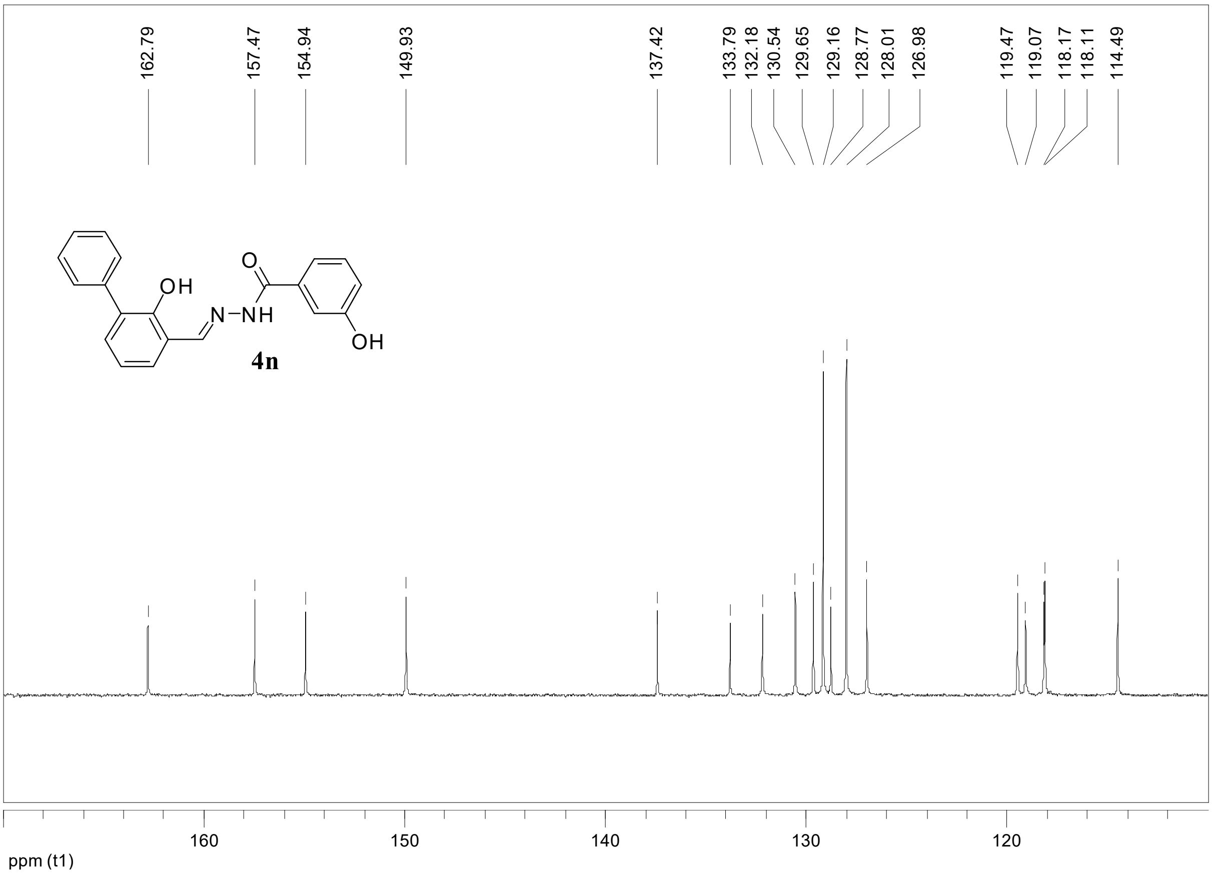

Figure S223. Expansion of ^{13}C-NMR (100 MHz, DMSO-d_6) spectrum of compound **4n**

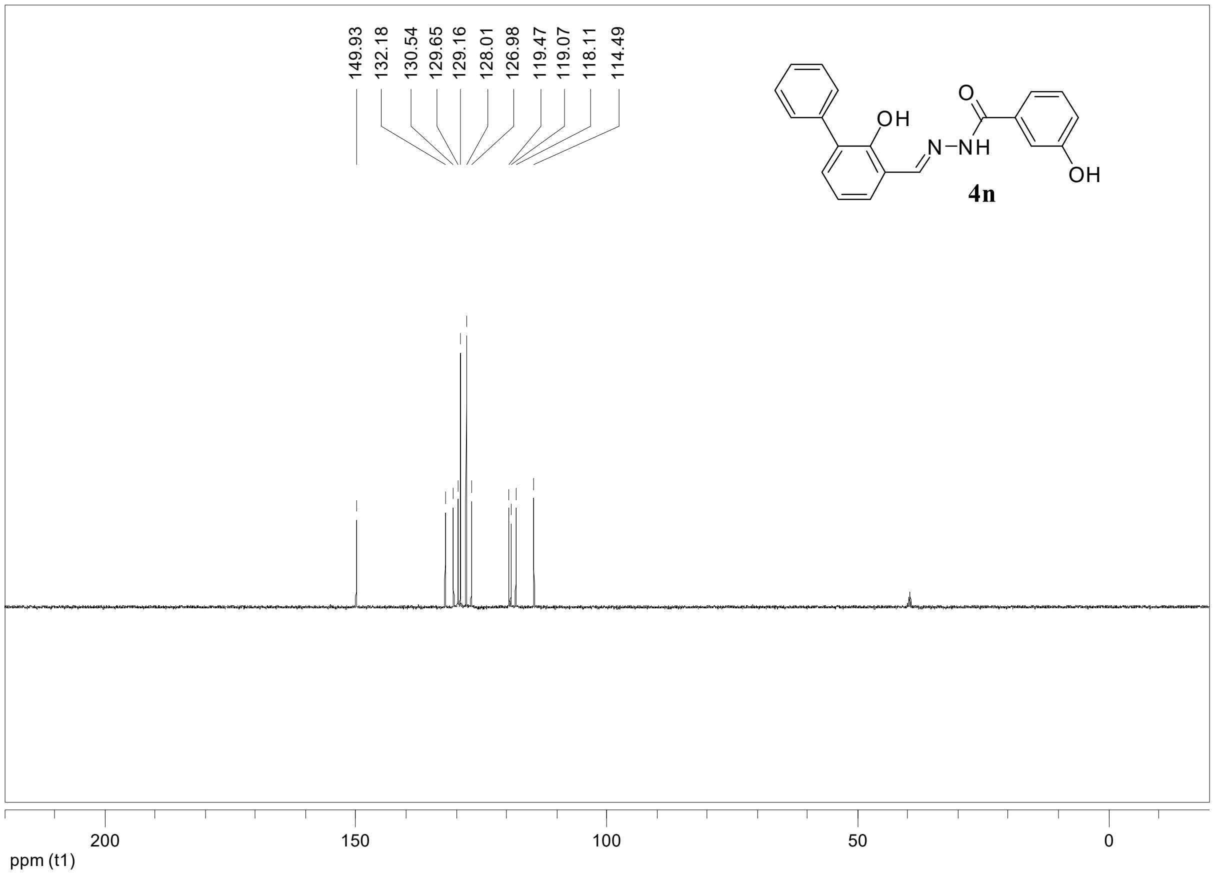

Figure S224. ^{13}C-NMR (100 MHz, DMSO-d_6) dept-135 experiment of compound **4n**

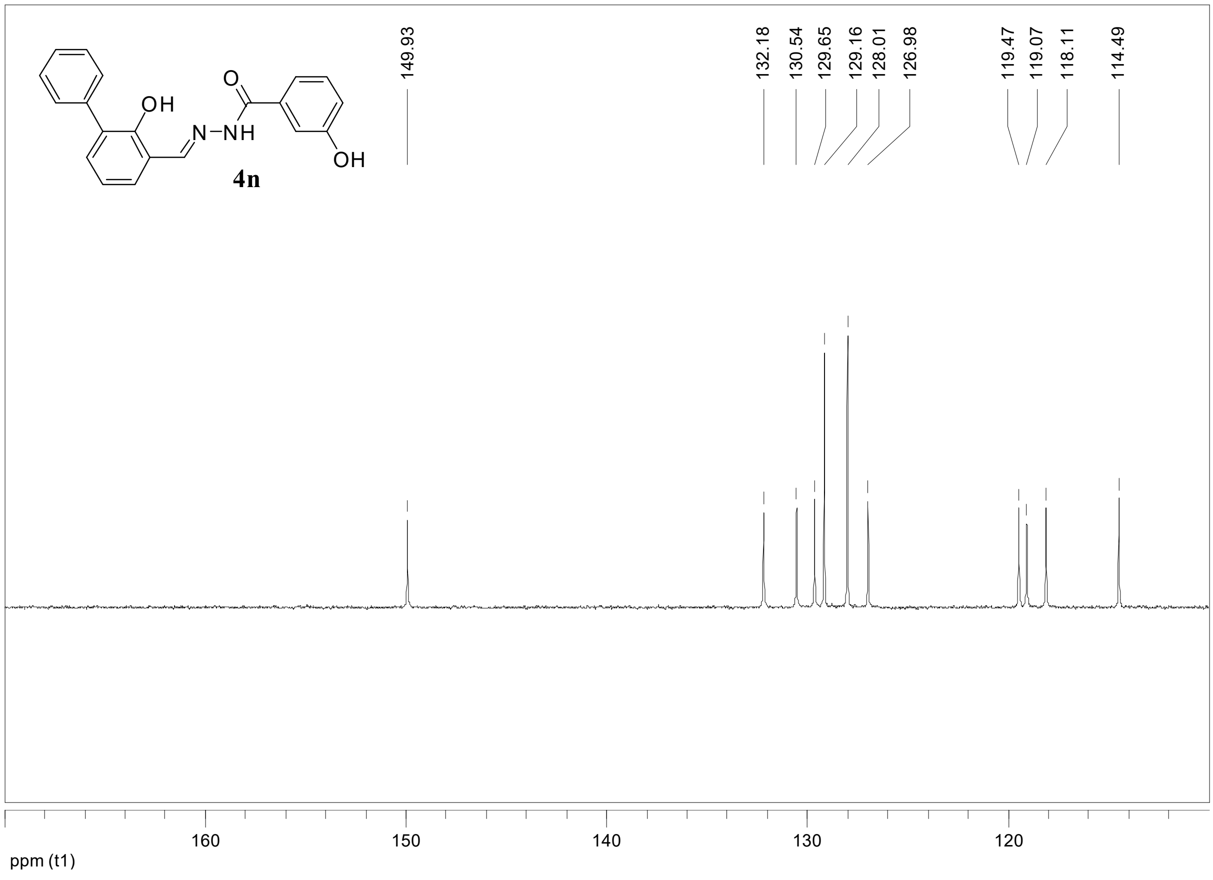

Figure S225. Expansion of ^{13}C-NMR (100 MHz, DMSO-d_6) dept-135 experiment of compound **4n**

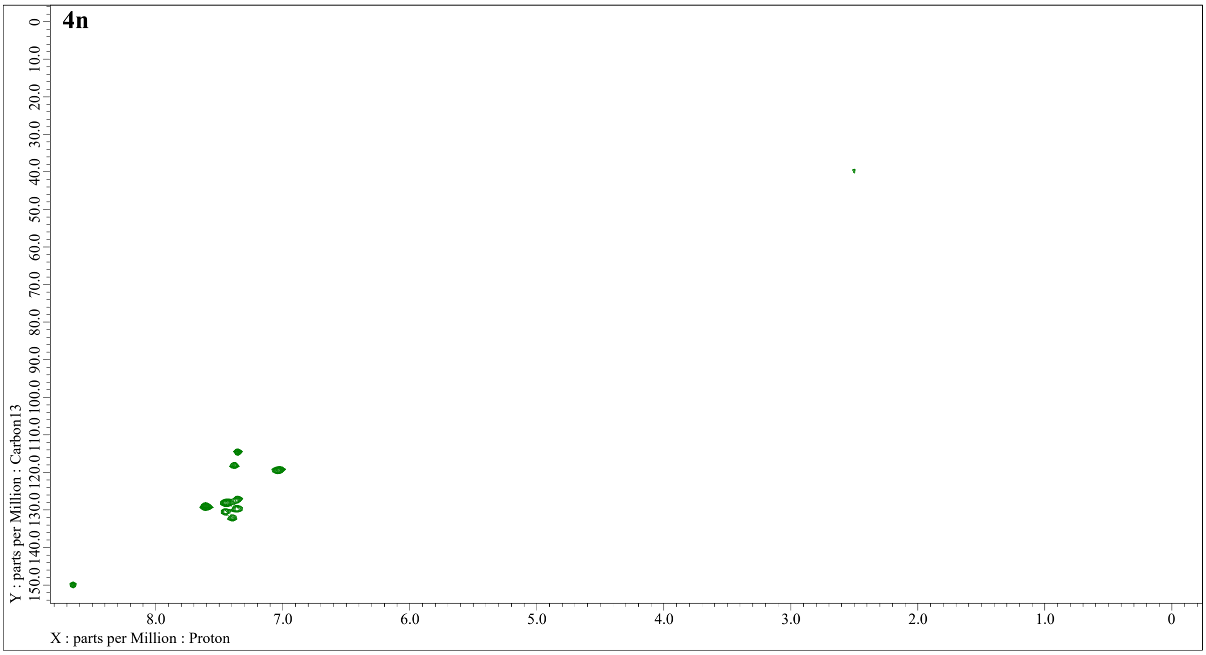

Figure S226. 2D-NMR (400 MHz, DMSO-d_6) HSQC experiment of 3-hydroxy-*N'*-[(*E*)-(2-hydroxy-3-phenyl-phenyl)methylidene]benzohydrazide (**4n**)

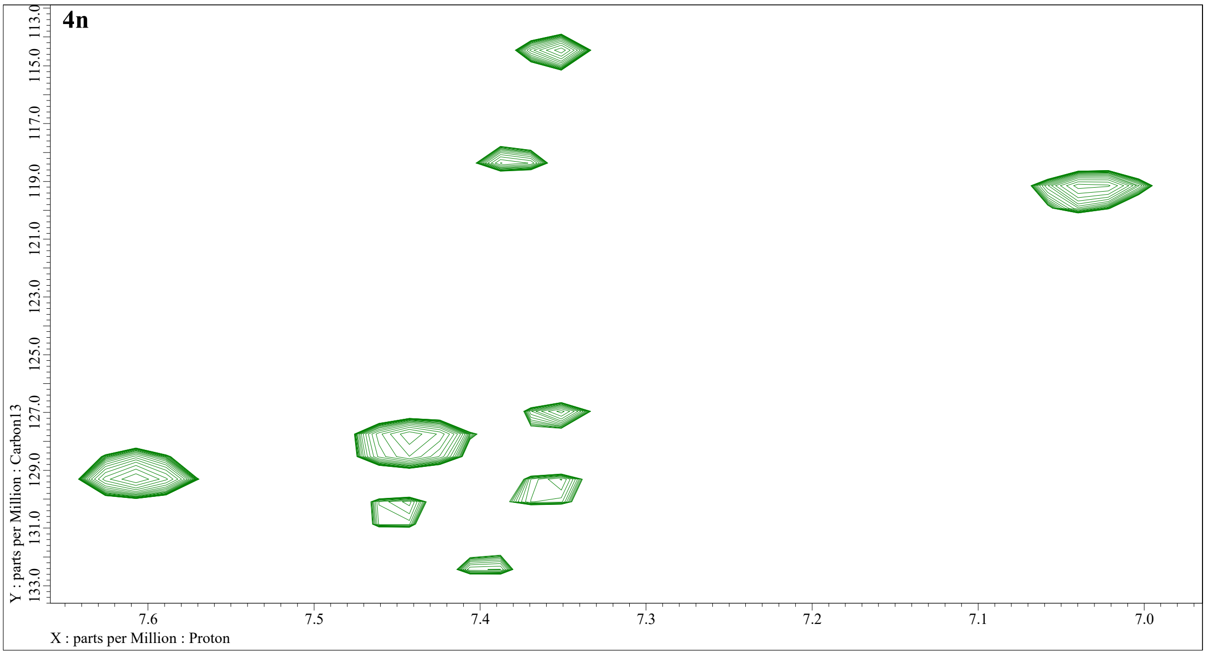

Figure S227. Expansion of 2D-NMR (400 MHz, DMSO-d_6) HSQC experiment of 3-hydroxy-*N'*-[(*E*)-(2-hydroxy-3-phenyl-phenyl)methylidene]benzohydrazide (**4n**)

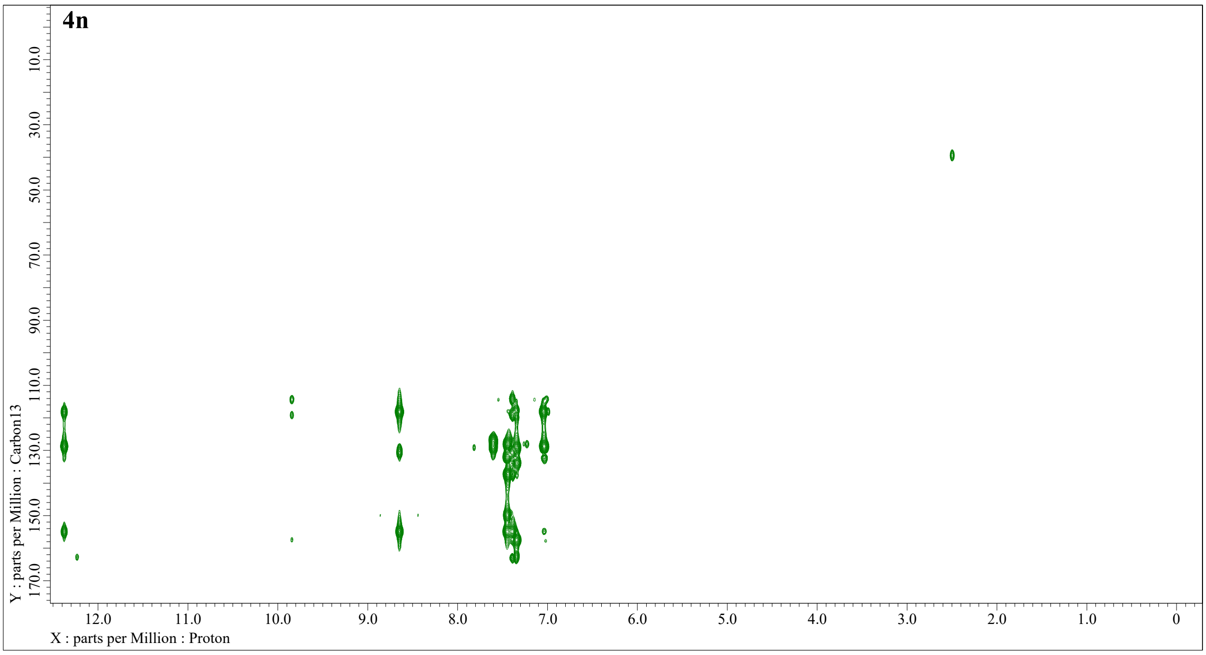

Figure S228. 2D-NMR (400 MHz, DMSO-d_6) HMBC experiment of 3-hydroxy-*N*'-[(*E*)-(2-hydroxy-3-phenyl-phenyl)methylidene]benzohydrazide (**4n**)

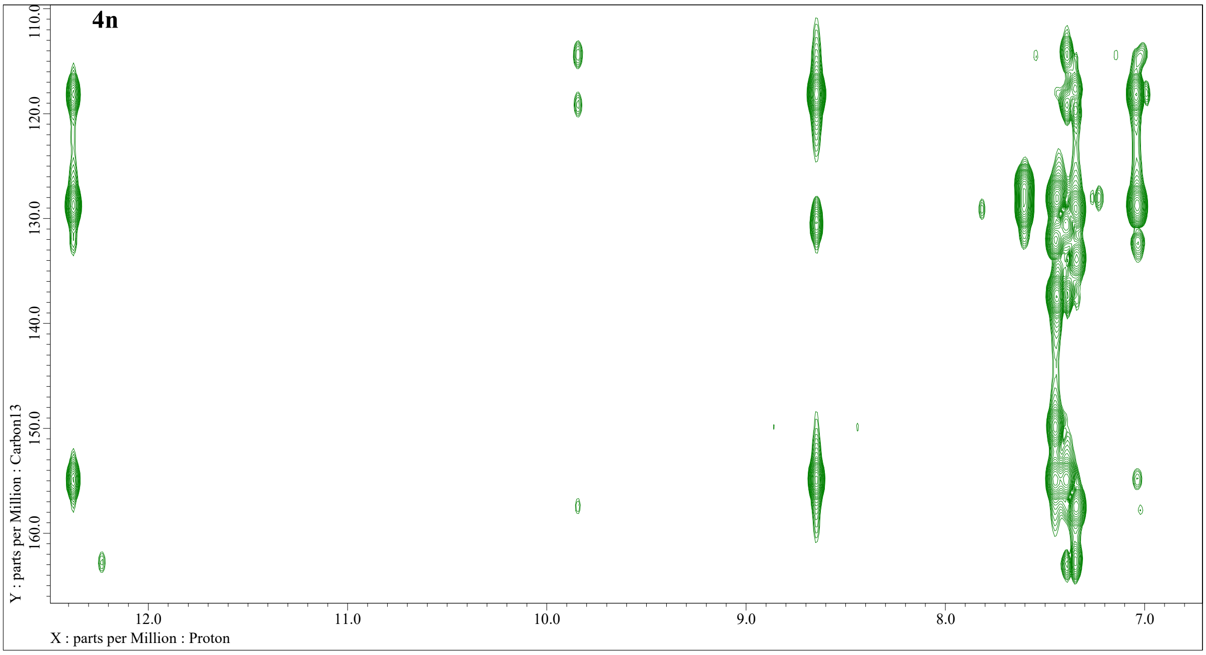

Figure S229. Expansion of 2D-NMR (400 MHz, DMSO-d_6) HMBC experiment of 3-hydroxy-*N*'-[(*E*)-(2-hydroxy-3-phenyl-phenyl)methylidene]benzohydrazide (**4n**)

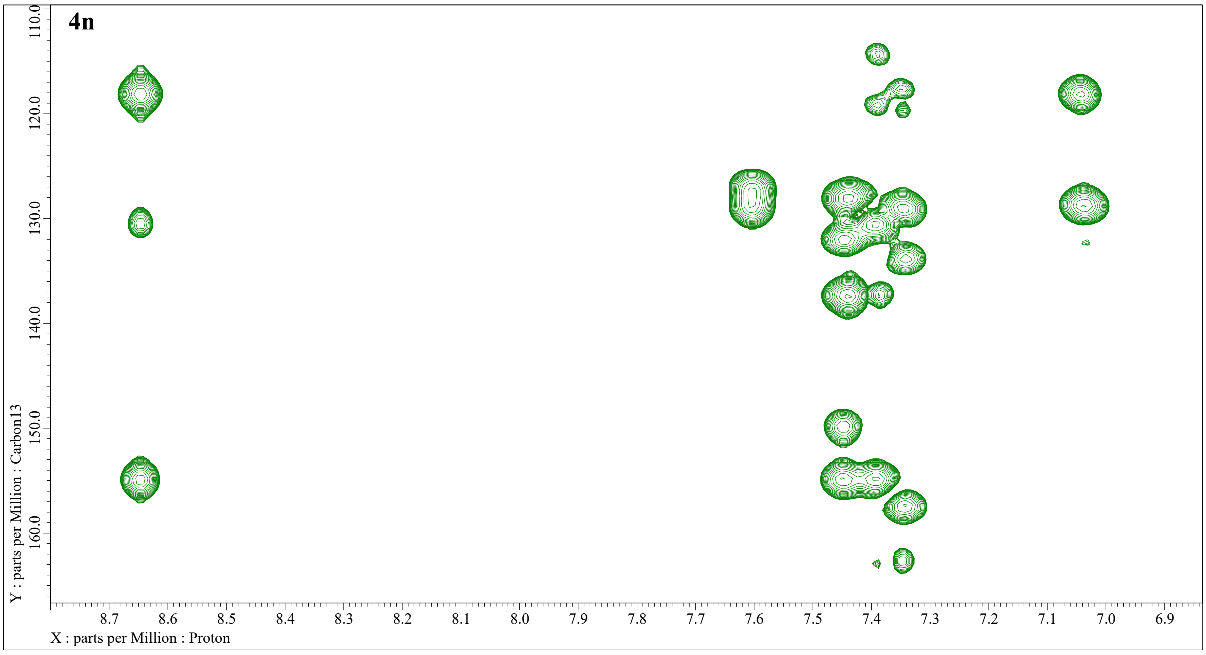

Figure S230. Expansion of 2D-NMR (400 MHz, DMSO-d_6) HMBC experiment of 3-hydroxy-*N*'-[(*E*)-(2-hydroxy-3-phenyl-phenyl)methylidene]benzohydrazide (**4n**)

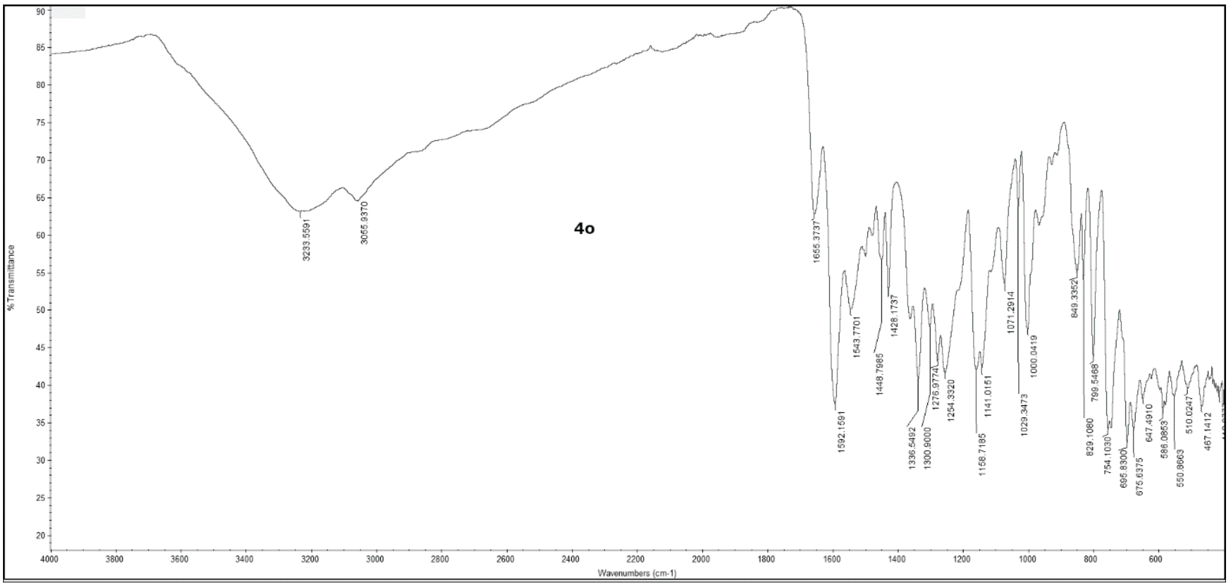

Figure S231. FT-IR (ATR) spectrum of compound **4o**

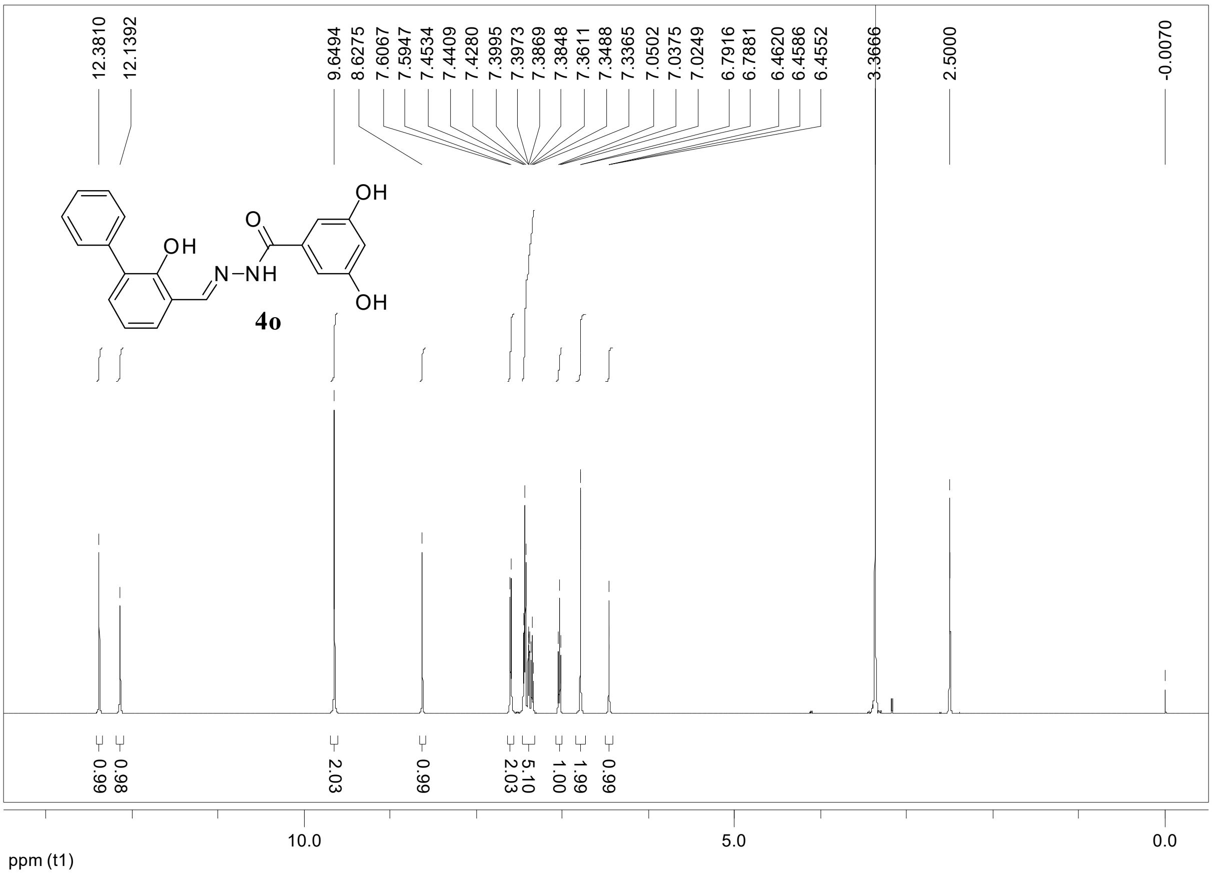

Figure S232. ^{1}H-NMR (600 MHz, DMSO-d_6) spectrum of compound **4o**

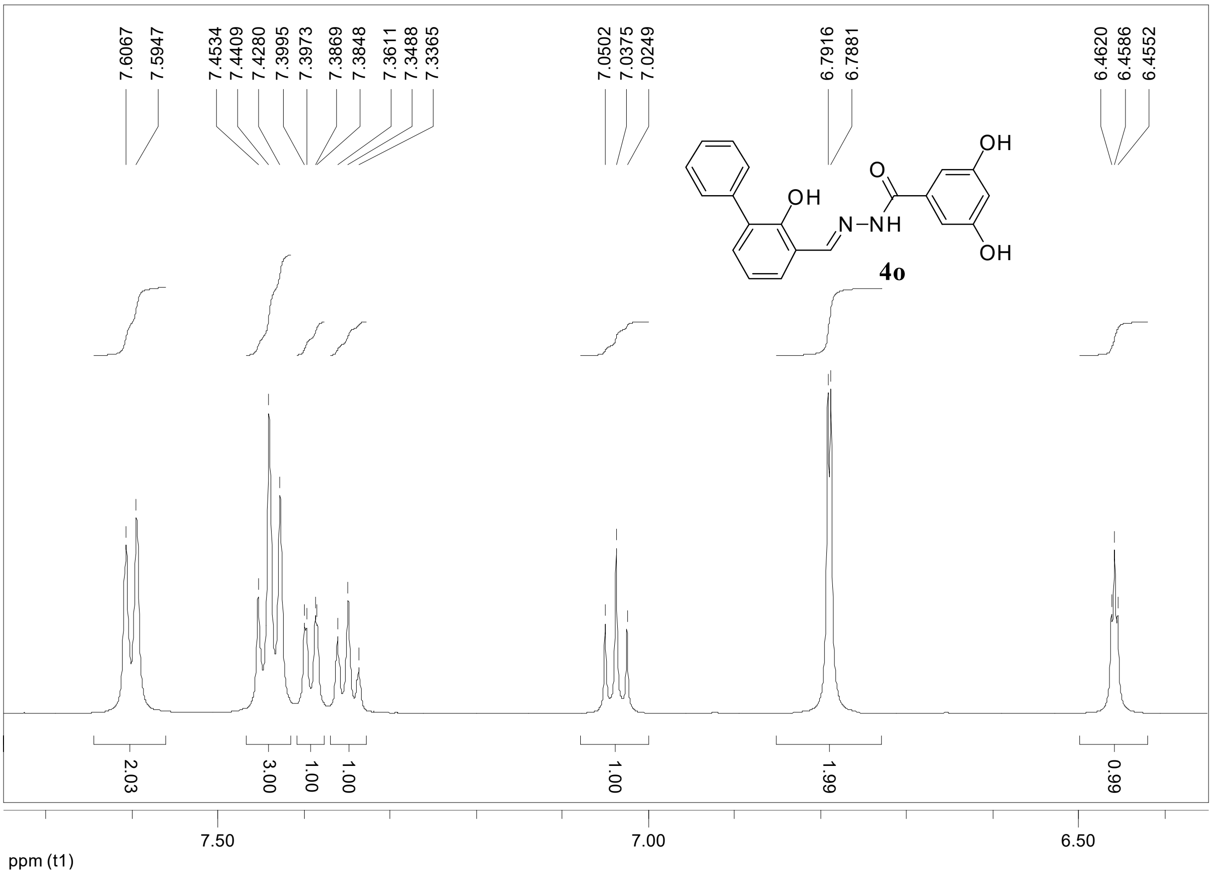

Figure S233. Expansion of ^{1}H-NMR (600 MHz, DMSO-d_6) spectrum of compound **4o**

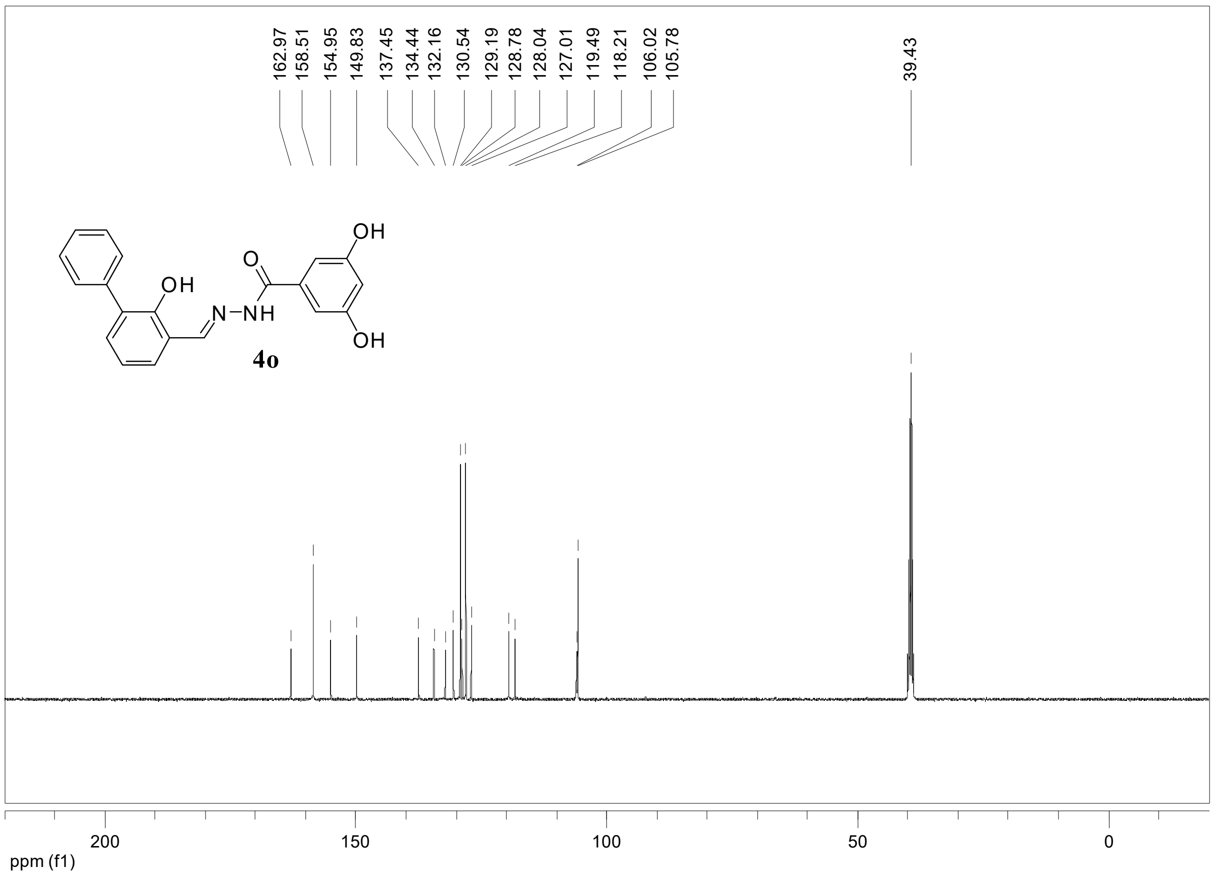

Figure S234. ^{13}C-NMR (100 MHz, DMSO-d_6) spectrum of compound **4o**

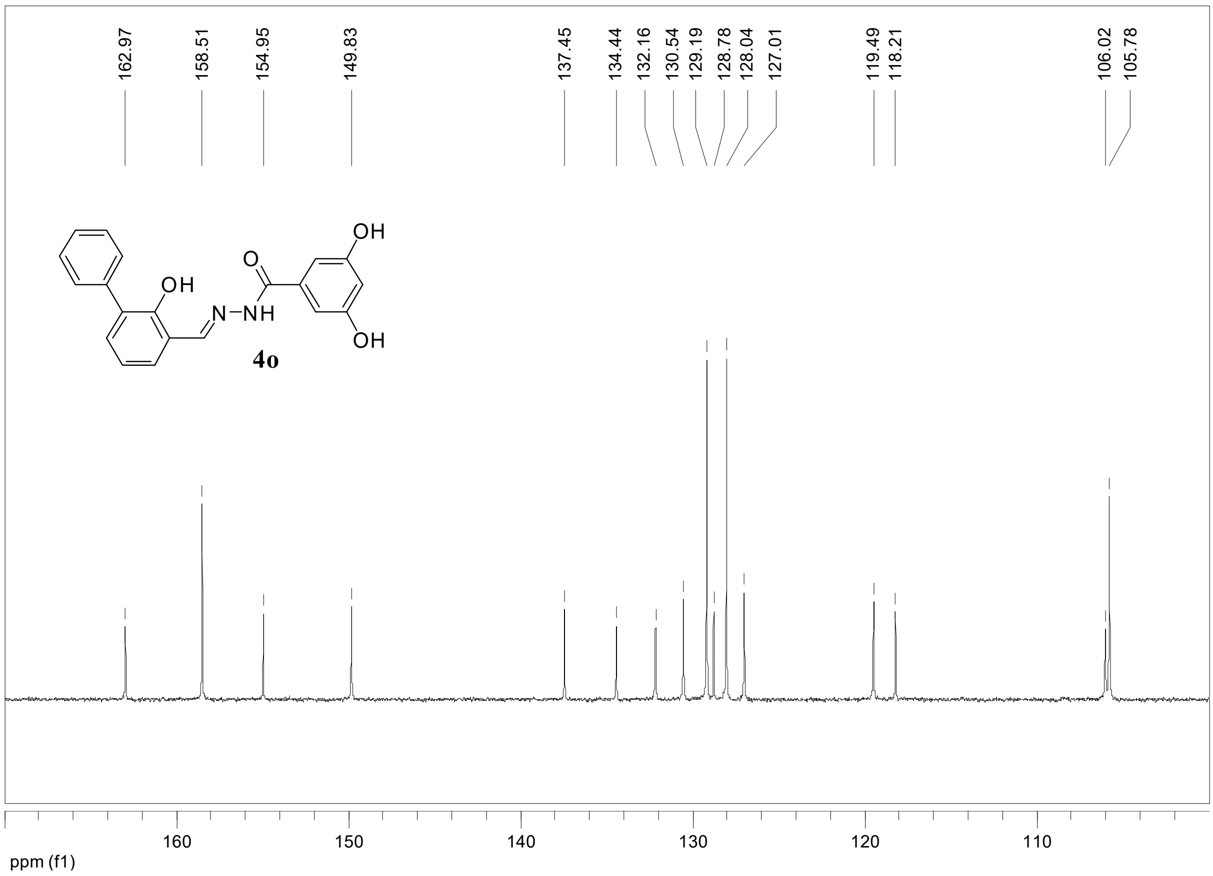

Figure S235. Expansion of ^{13}C-NMR (100 MHz, DMSO-d_6) spectrum of compound **4o**

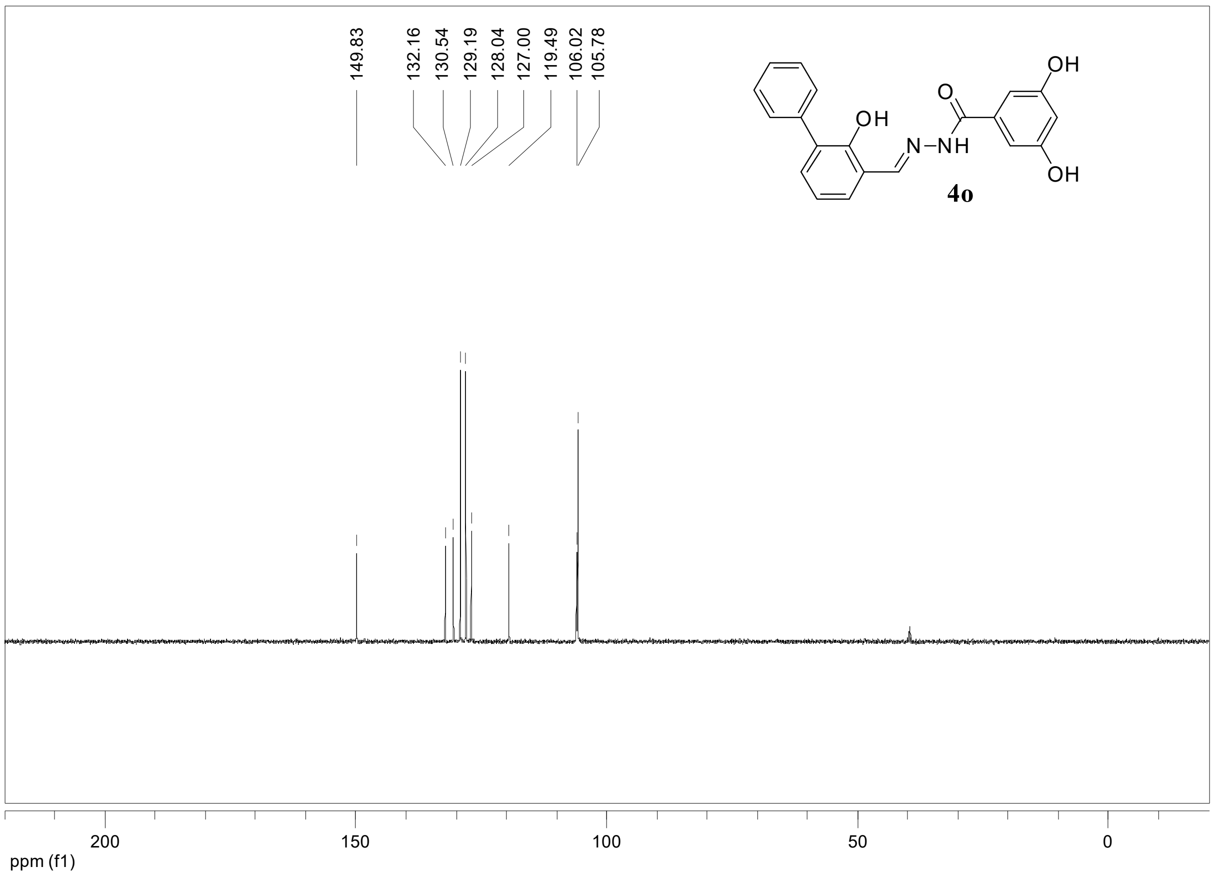

Figure S236. ^{13}C-NMR (100 MHz, DMSO-d_6) dept-135 experiment of compound **4o**

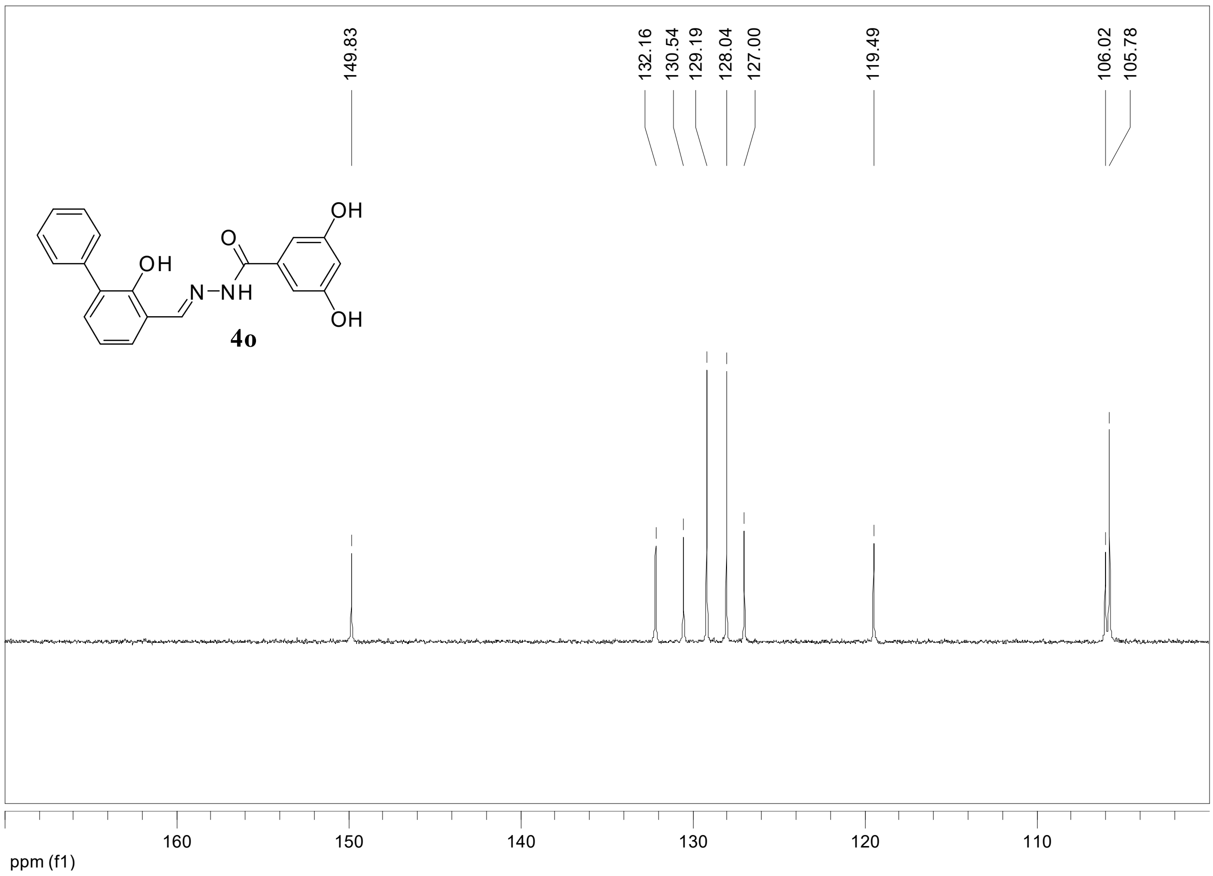

Figure S237. Expansion of ^{13}C-NMR (100 MHz, DMSO-d_6) dept-135 experiment of compound **4o**

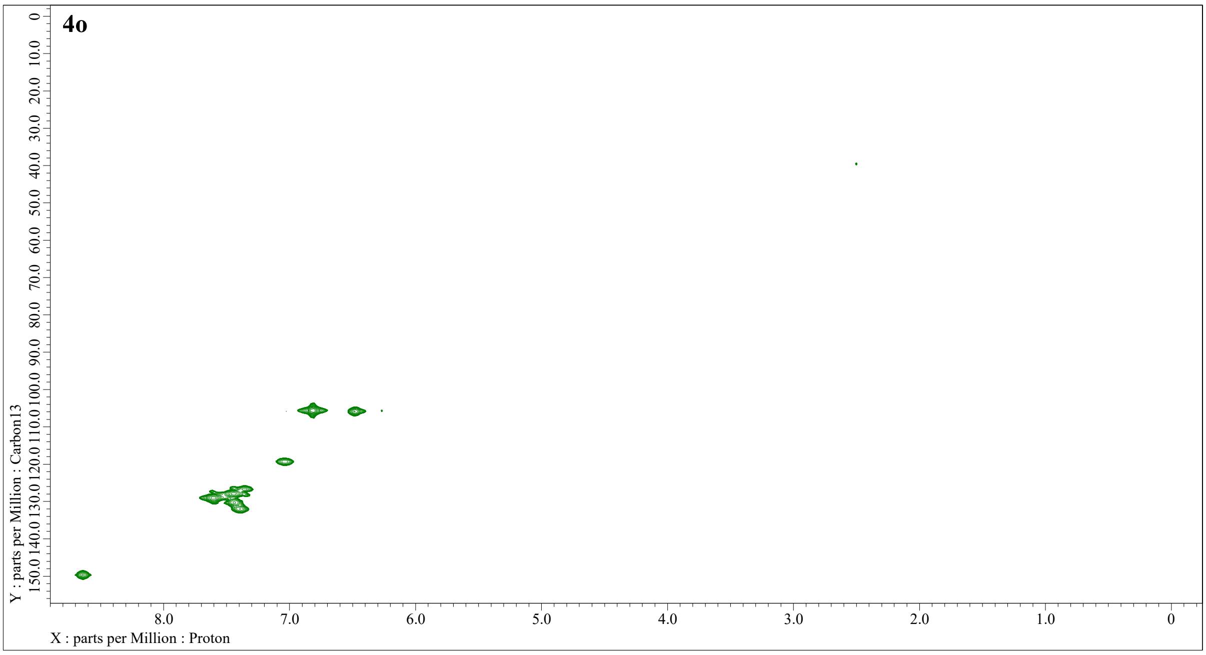

Figure S238. 2D-NMR (400 MHz, DMSO-d_6) HMQC experiment of compound **4o**

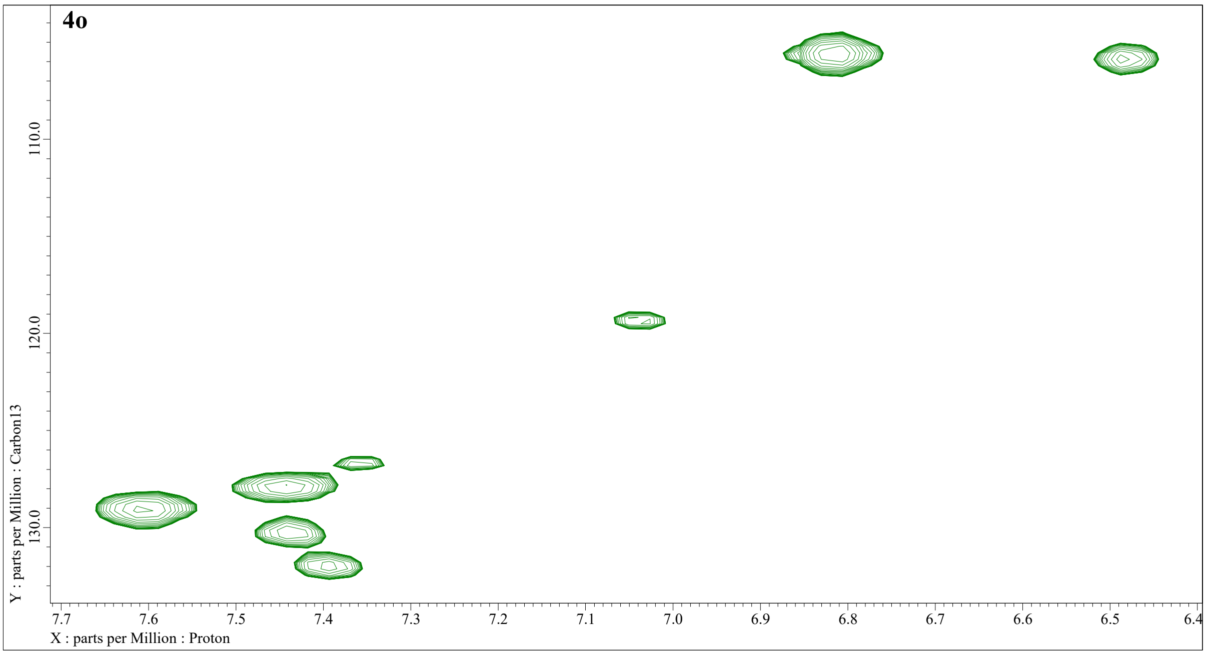

Figure S239. Expansion of 2D-NMR (400 MHz, DMSO-d_6) HMQC experiment of compound **4o**

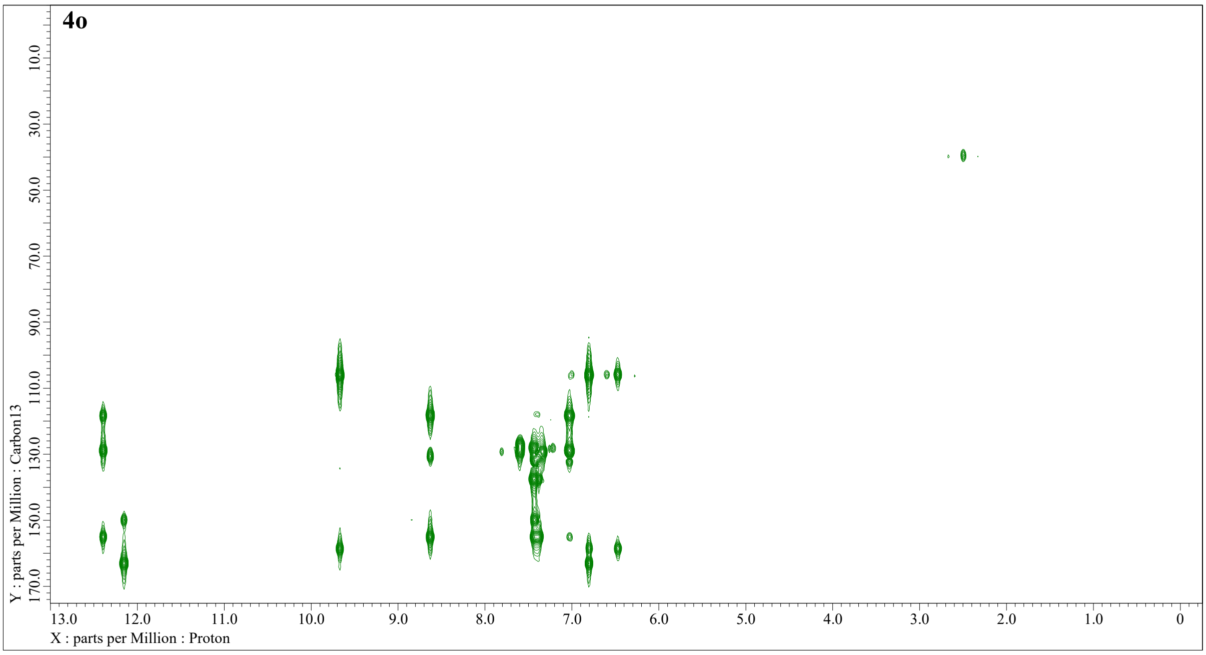

Figure S240. 2D-NMR (400 MHz, DMSO-d_6) HMBC experiment of compound **4o**

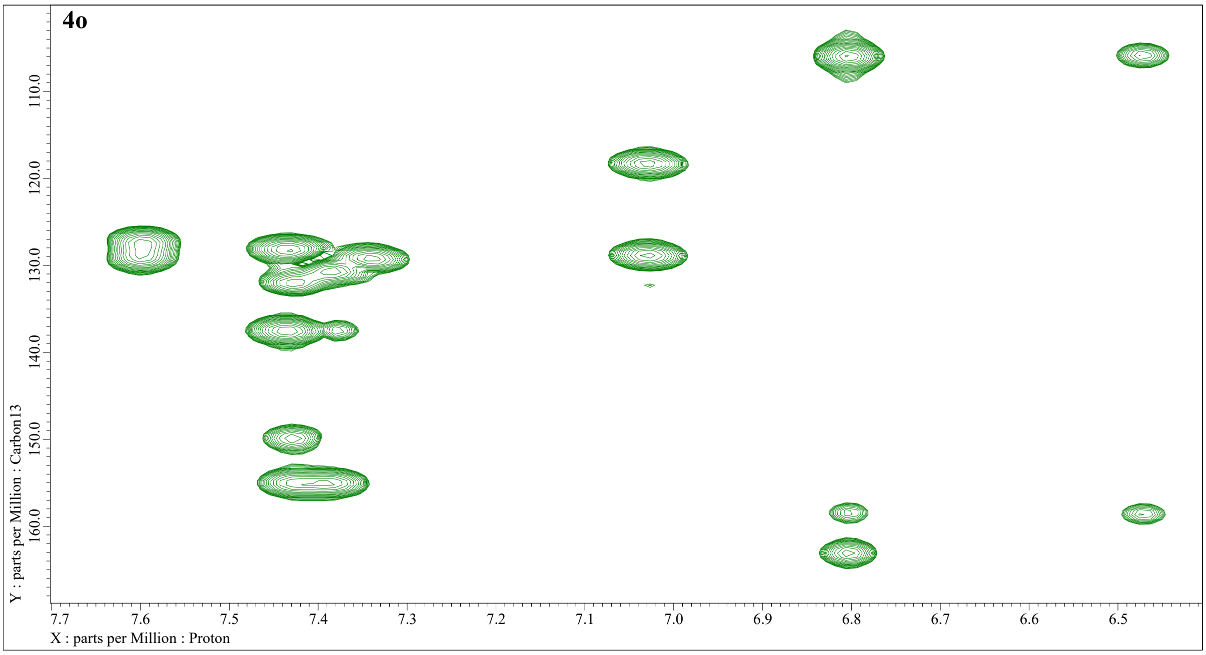

Figure S241. Expansion of 2D-NMR (400 MHz, DMSO-d_6) HMBC experiment of compound **4o**

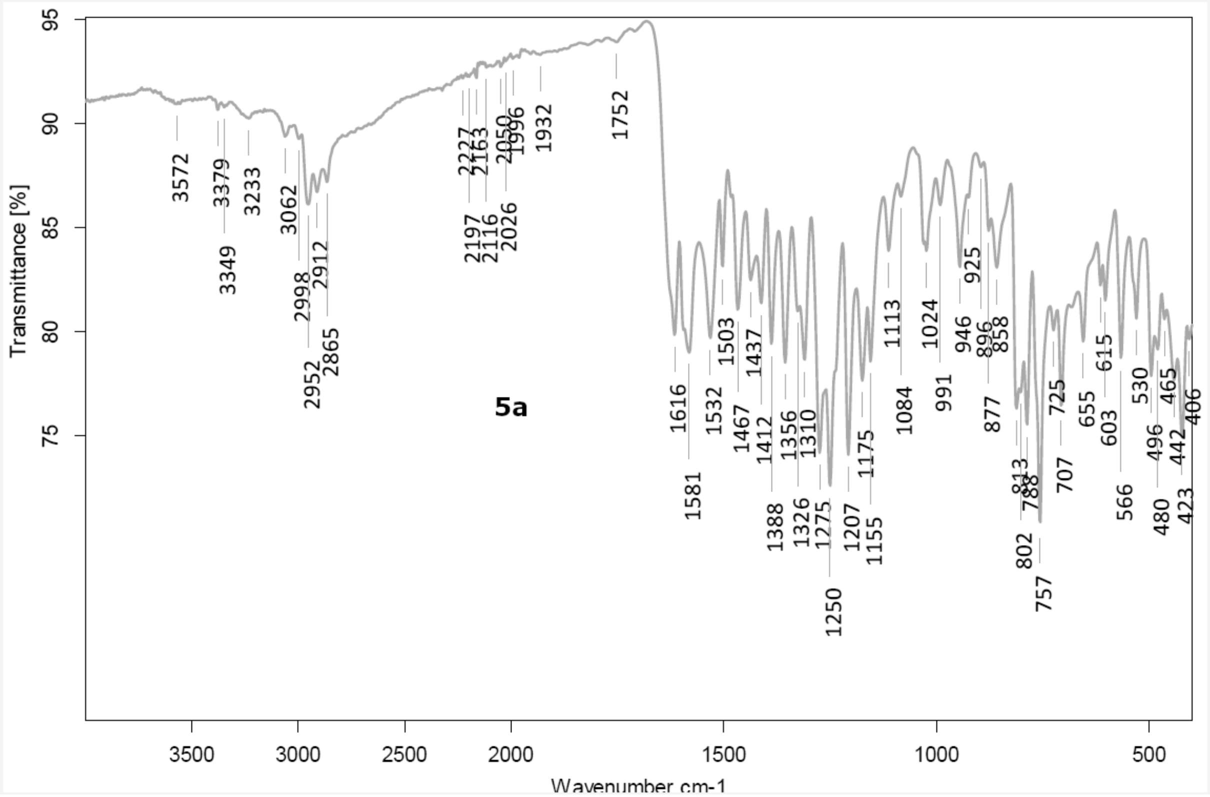

Figure S242. FT-IR (ATR) spectrum of *N'*-[(*E*)-(3-*tert*-butyl-2-hydroxy-5-methylphenyl)methylidene]-2-(1-hydroksynaphtho)hydrazide (**5a**)

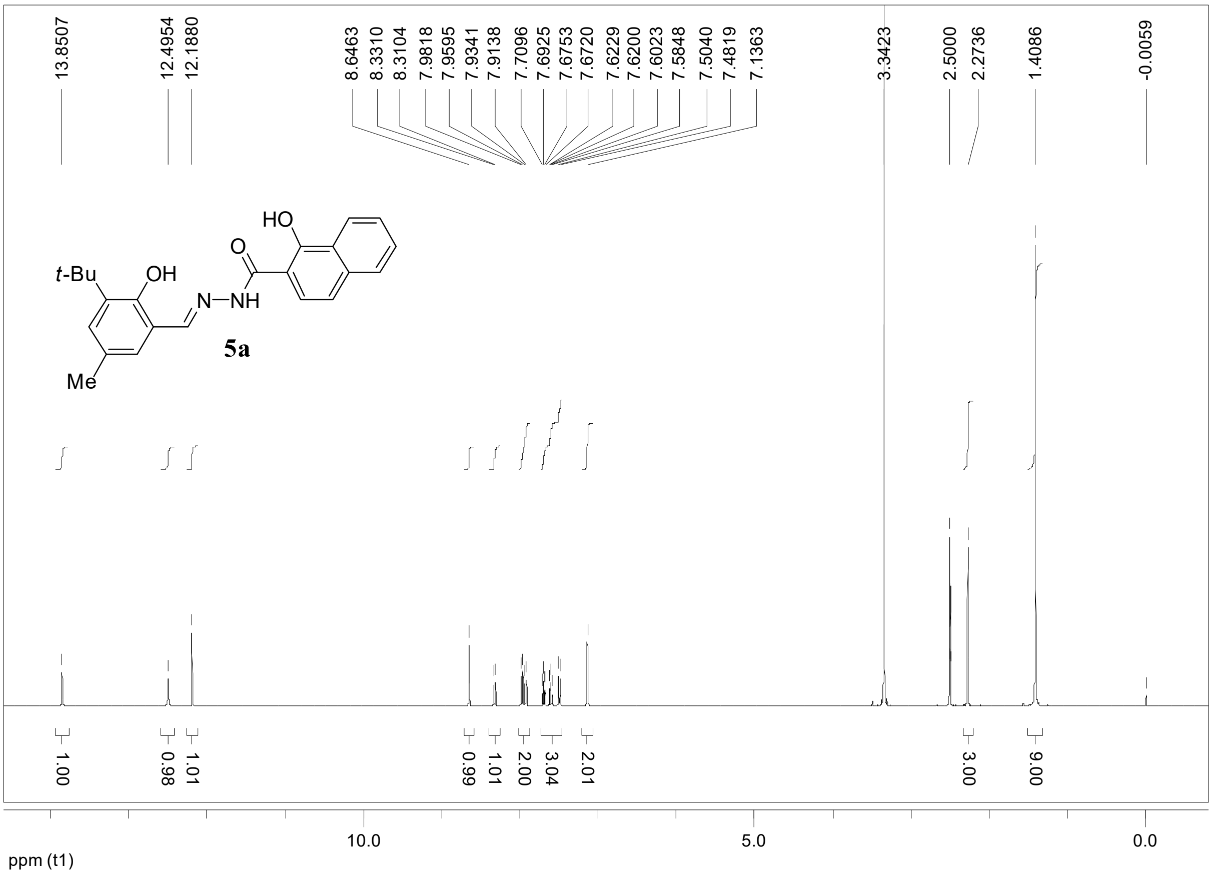

Figure S243. ^{1}H-NMR (400 MHz, DMSO-d_6) spectrum of compound **5a**

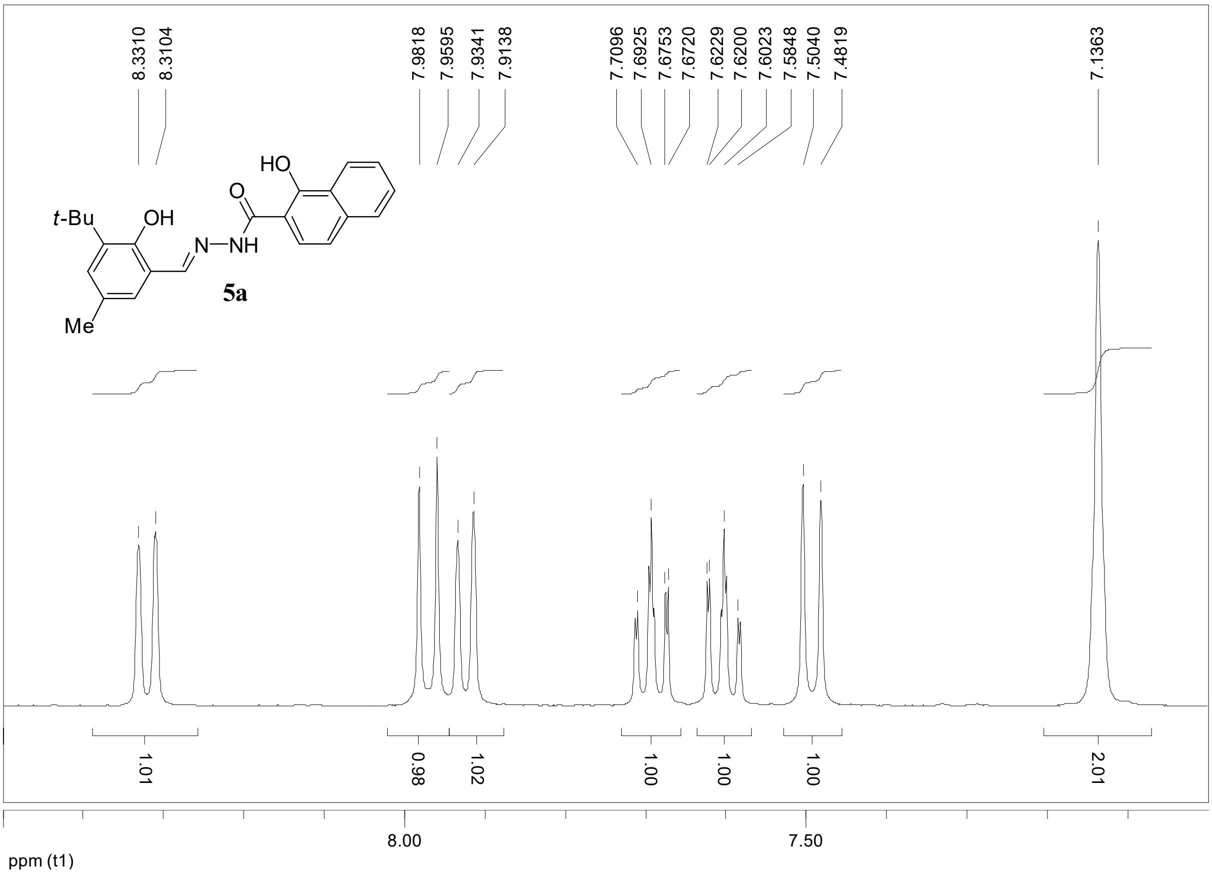

Figure S244. Expansion of ^{1}H-NMR (400 MHz, DMSO-d_6) spectrum of compound **5a**

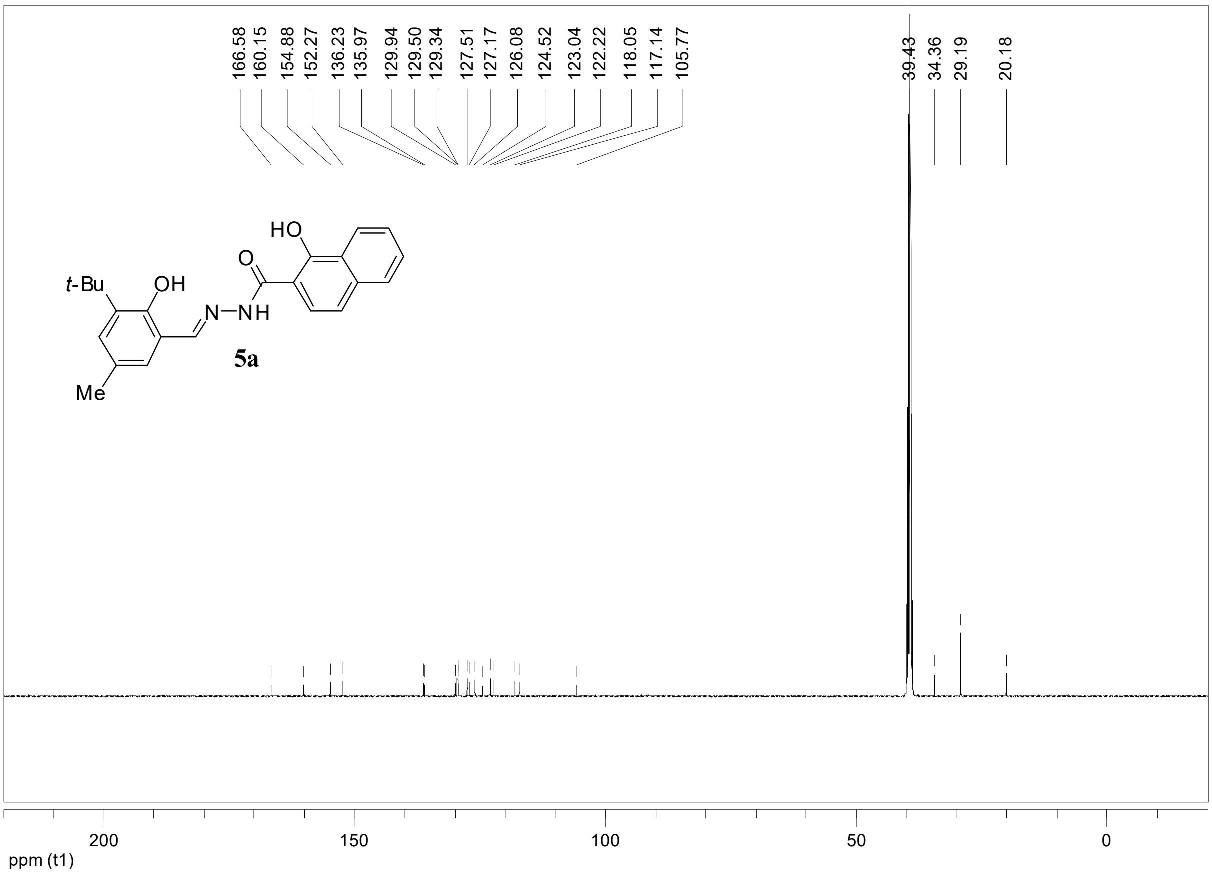

Figure S245. ^{13}C-NMR (100 MHz, DMSO-d_6) spectrum of compound **5a**

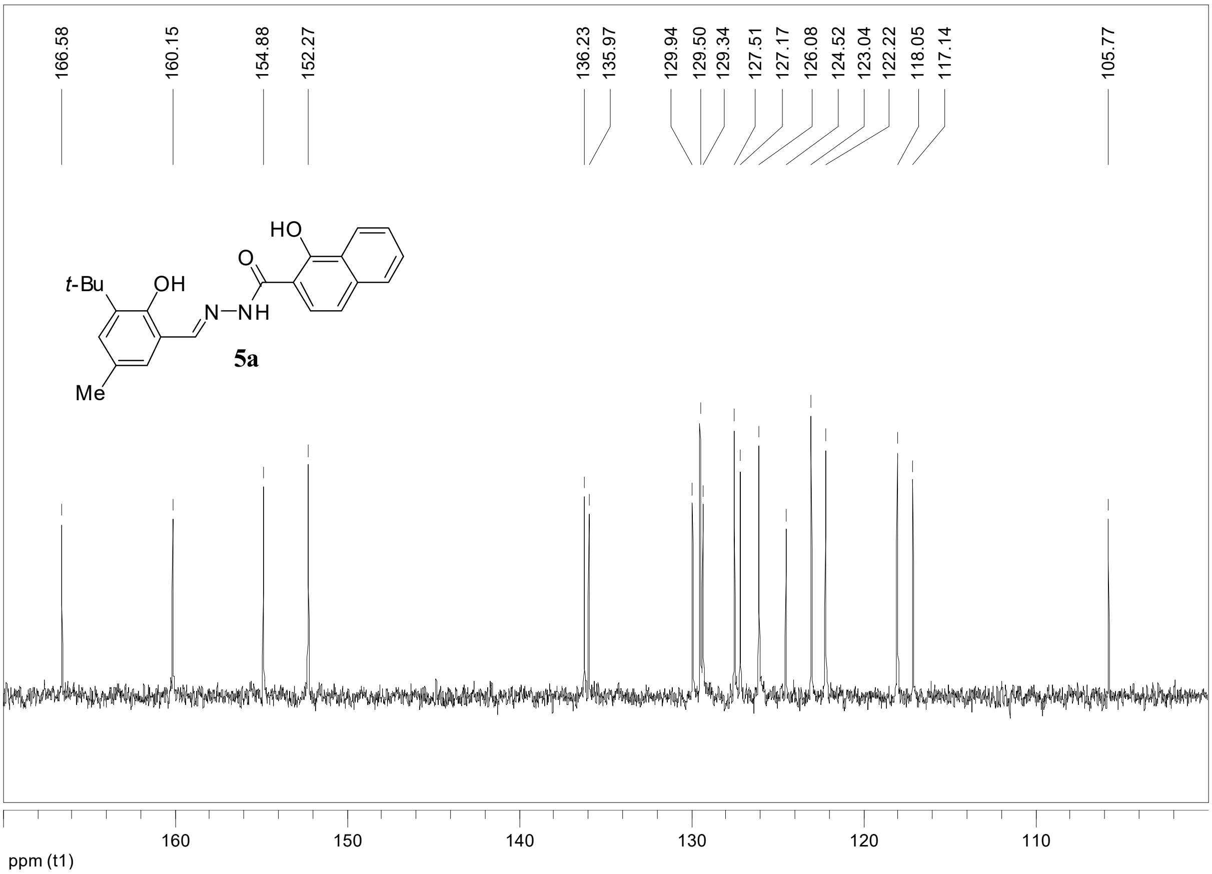

Figure S246. Expansion of ^{13}C-NMR (100 MHz, DMSO-d_6) spectrum of compound **5a**

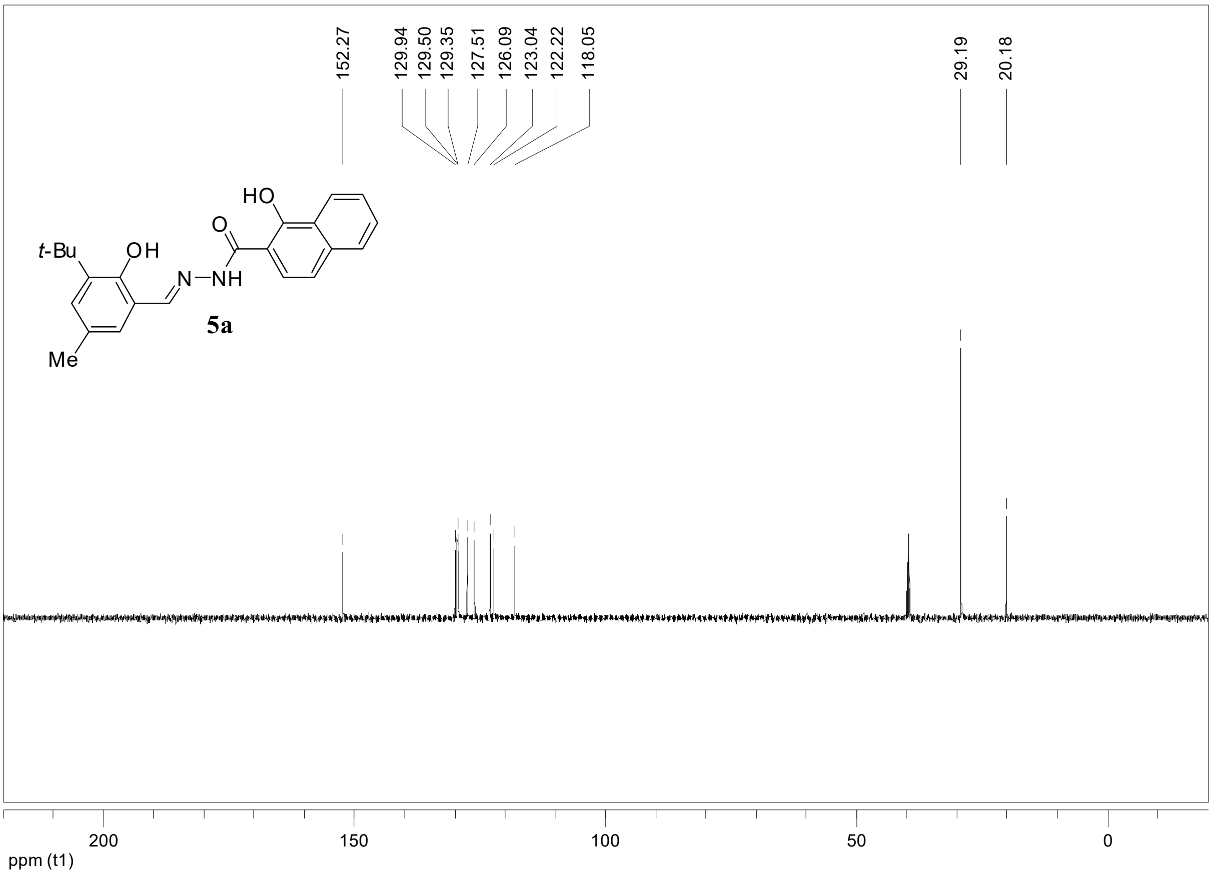

Figure S247. ^{13}C-NMR (100 MHz, DMSO-d_6) dept-135 experiment of compound **5a**

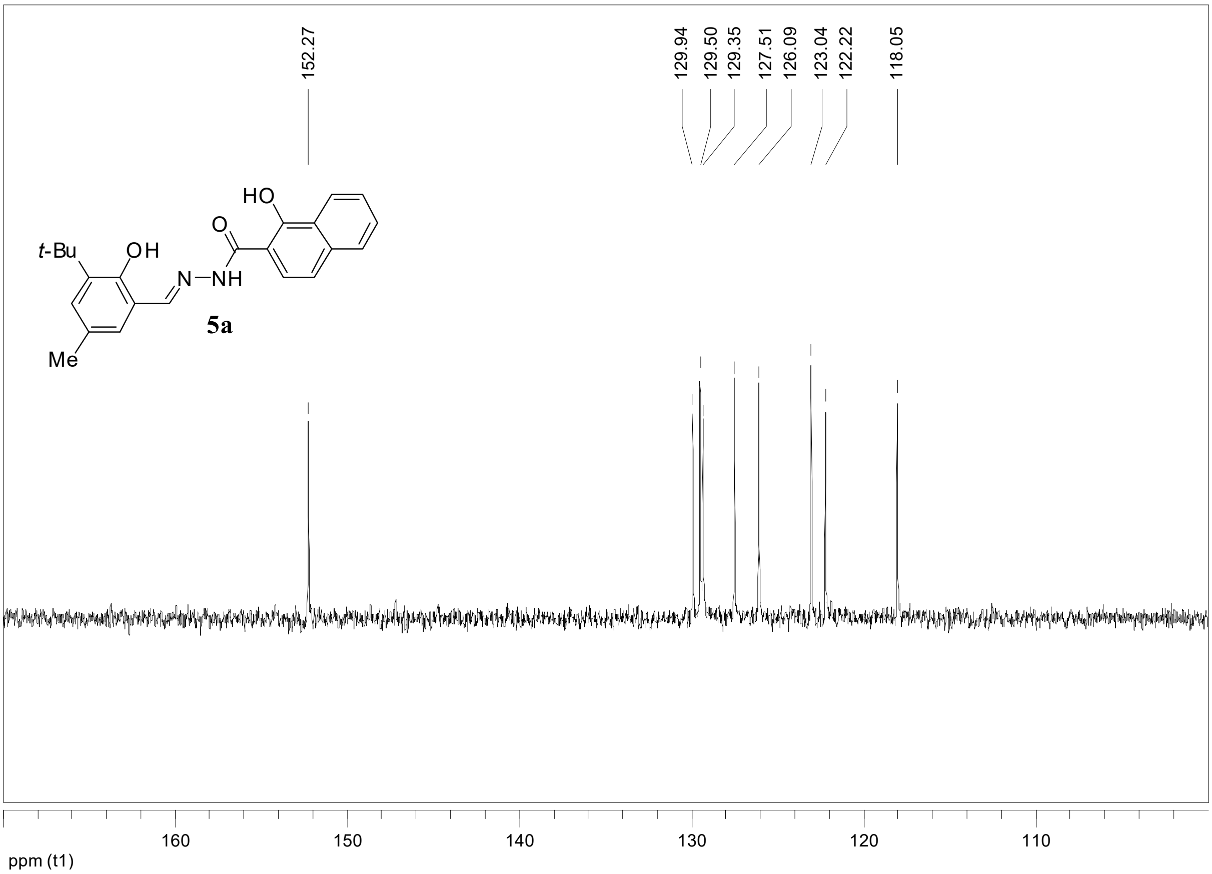

Figure S248. Expansion of ^{13}C-NMR (100 MHz, DMSO-d_6) dept-135 experiment of compound **5a**

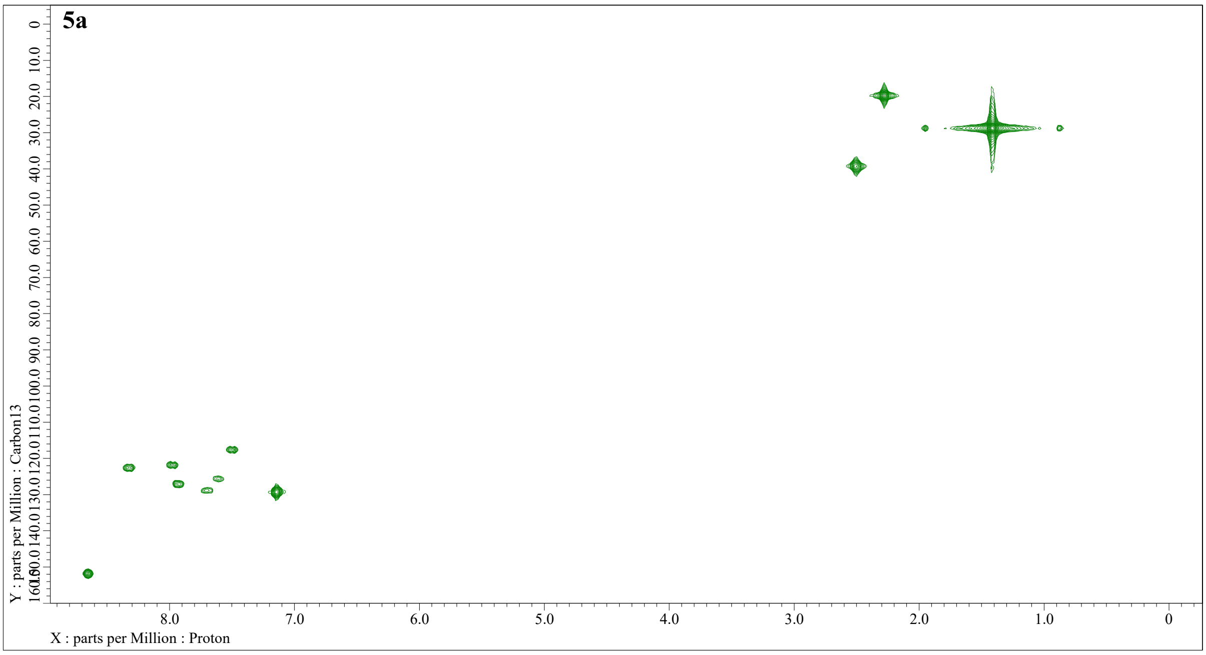

Figure S249. 2D-NMR (400 MHz, DMSO-d_6) HMQC experiment of *N'*-[(*E*)-(3-*tert*-butyl-2-hydroxy-5-methylphenyl)methylidene]-2-(1-hydroksynaphtho)hydrazide (**5a**)

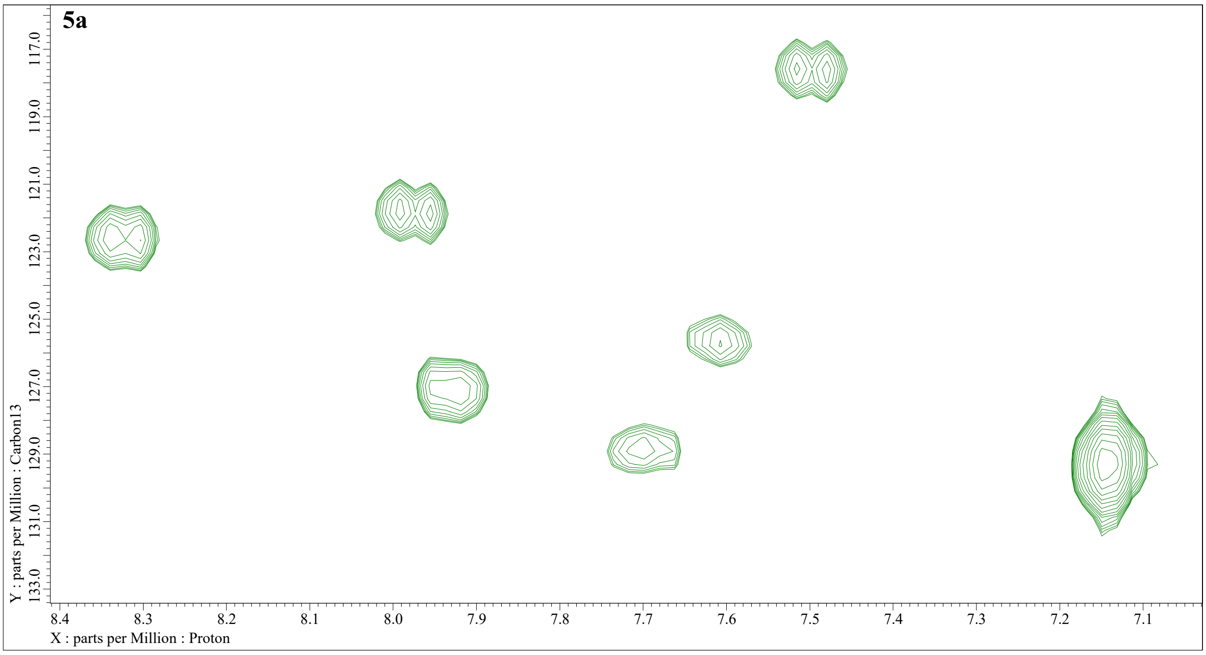

Figure S250. Expansion of 2D-NMR (400 MHz, DMSO-d_6) HMQC experiment of *N'*-[(*E*)-(3-*tert*-butyl-2-hydroxy-5-methylphenyl)methylidene]-2-(1-hydroksynaphtho)hydrazide (**5a**)

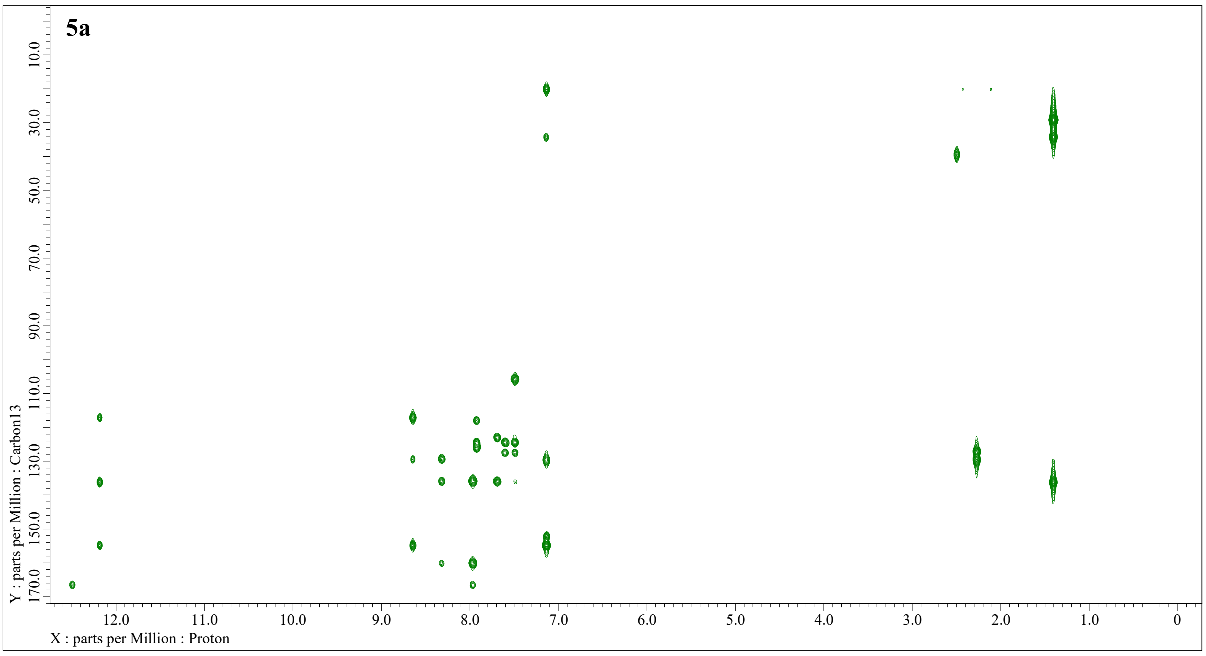

Figure S251. 2D-NMR (400 MHz, DMSO-d_6) HMBC experiment of *N*'-[(*E*)-(3-*tert*-butyl-2-hydroxy-5-methylphenyl)methylidene]-2-(1-hydroksynaphtho)hydrazide (**5a**)

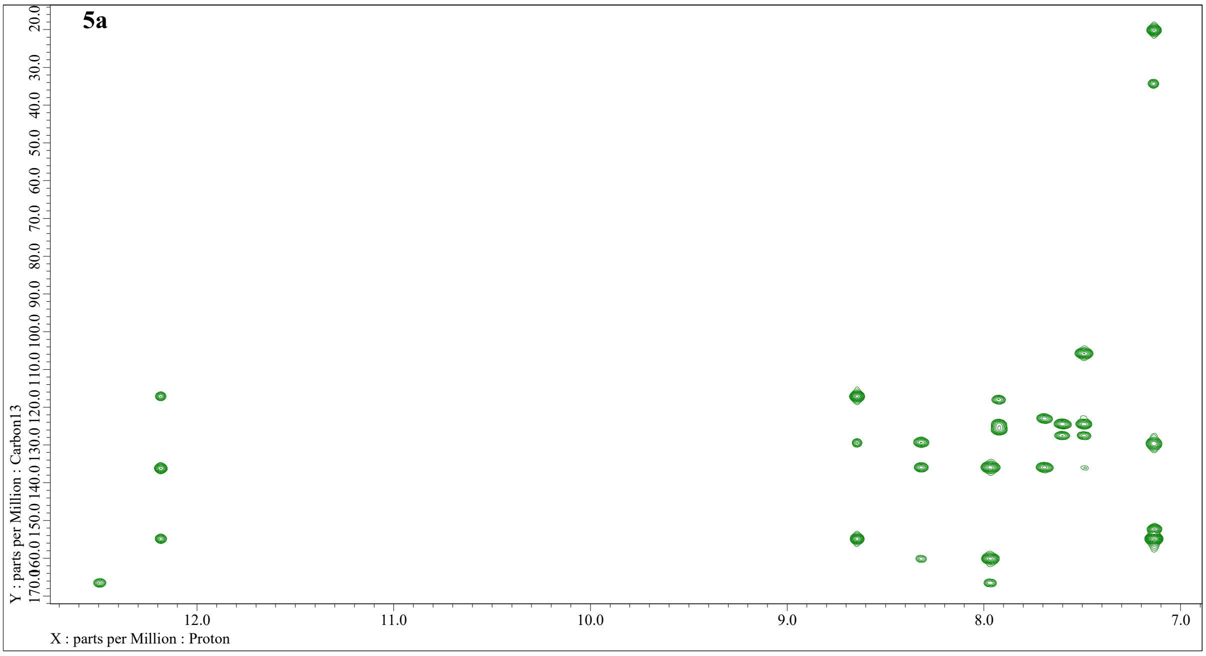

Figure S252. Expansion of 2D-NMR (400 MHz, DMSO-d_6) HMBC experiment of *N*'-[(*E*)-(3-*tert*-butyl-2-hydroxy-5-methylphenyl)methylidene]-2-(1-hydroksynaphtho)hydrazide (**5a**)

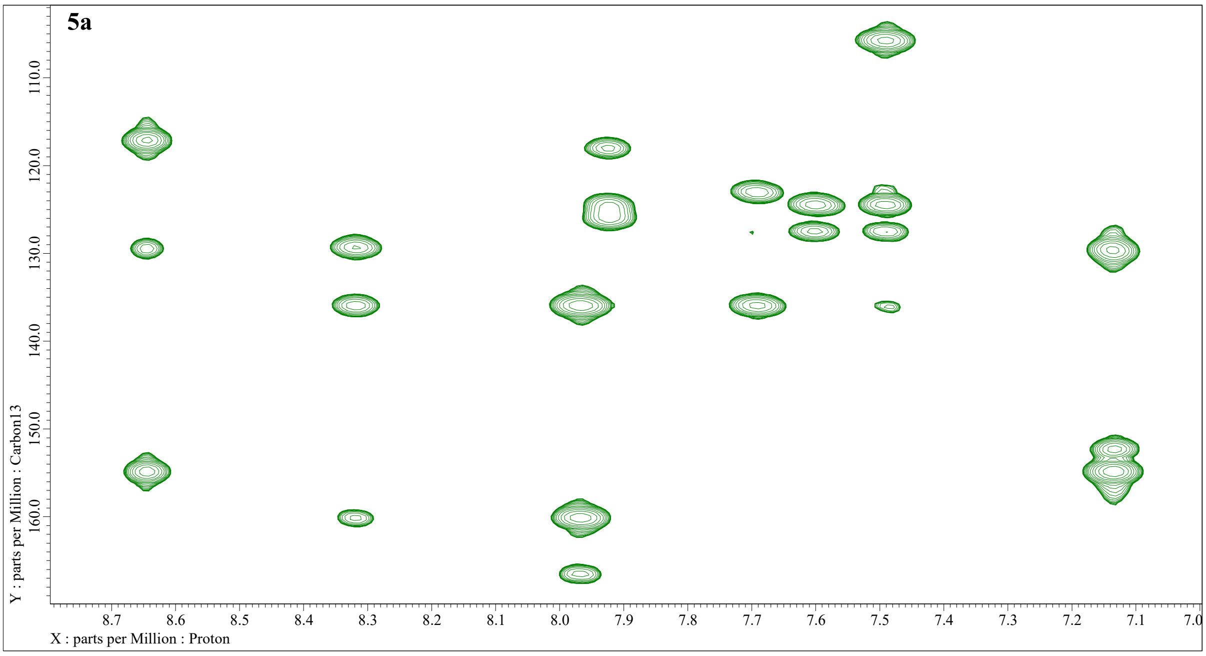

Figure S253. Expansion of 2D-NMR (400 MHz, DMSO-d_6) HMBC experiment of *N*'-[(*E*)-(3-*tert*-butyl-2-hydroxy-5-methylphenyl)methylidene]-2-(1-hydroksynaphtho)hydrazide (**5a**)

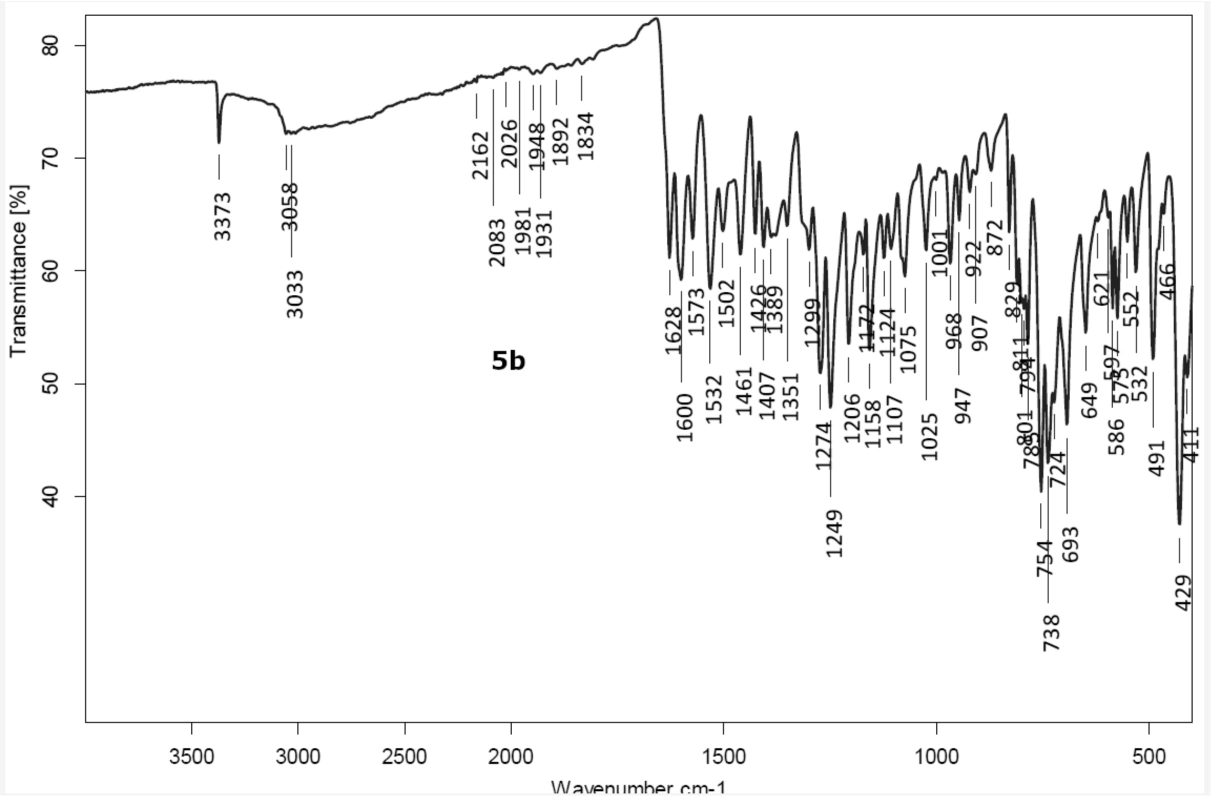

Figure S254. FT-IR (ATR) spectrum of *N*'-[(*E*)-(2-hydroxy-3-phenylphenyl)methylidene]-2-(1-hydroksynaphtho)hydrazide (**5b**)

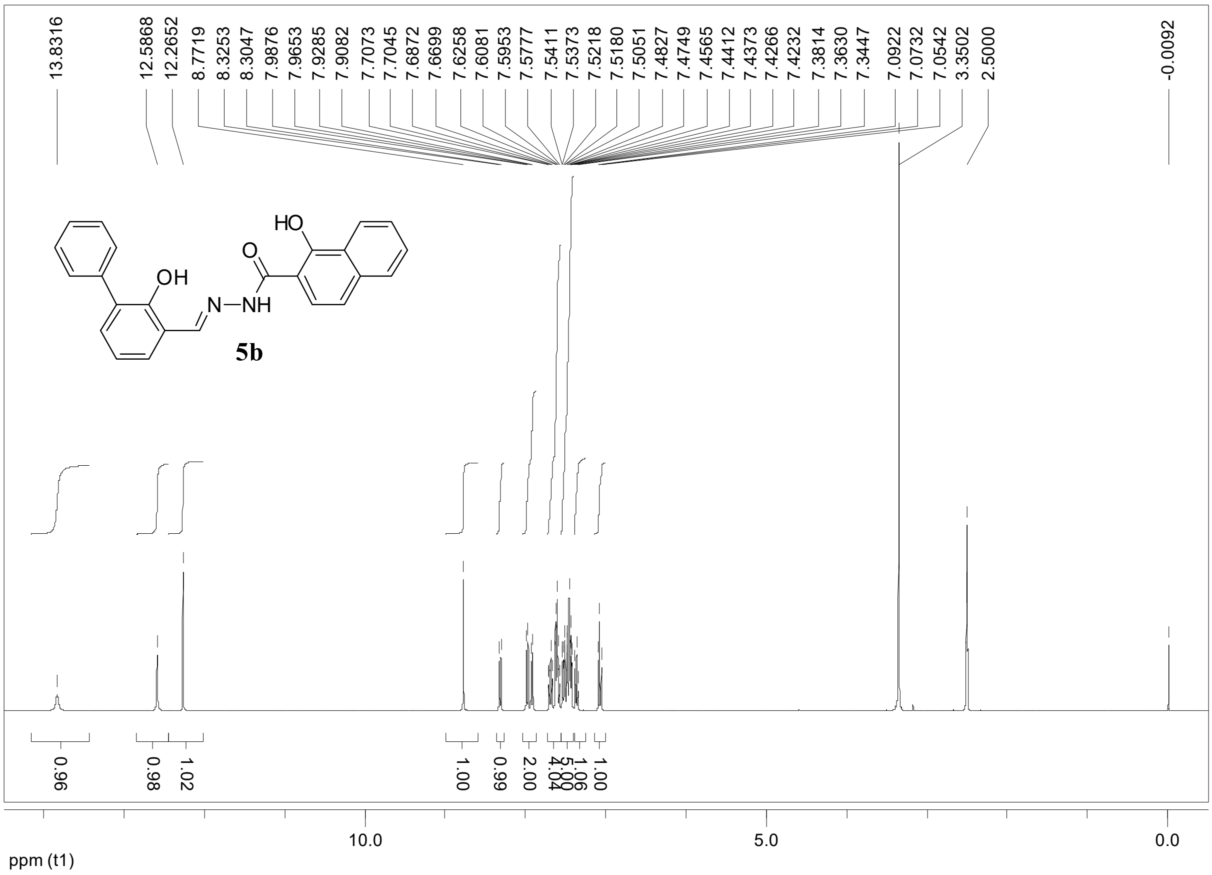

Figure S255. ^{1}H-NMR (400 MHz, DMSO-d_6) spectrum of compound **5b**

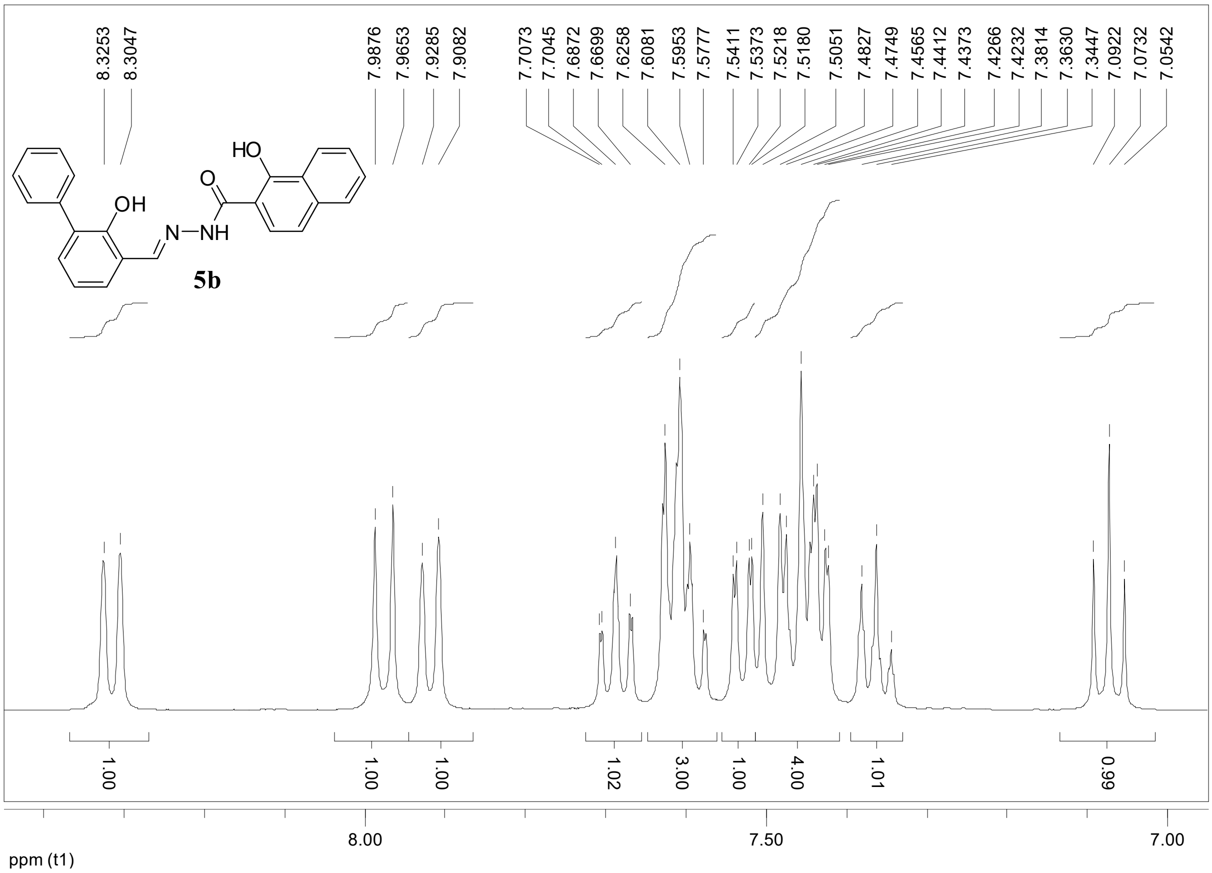

Figure S256. Expansion of ^{1}H-NMR (400 MHz, DMSO-d_6) spectrum of compound **5b**

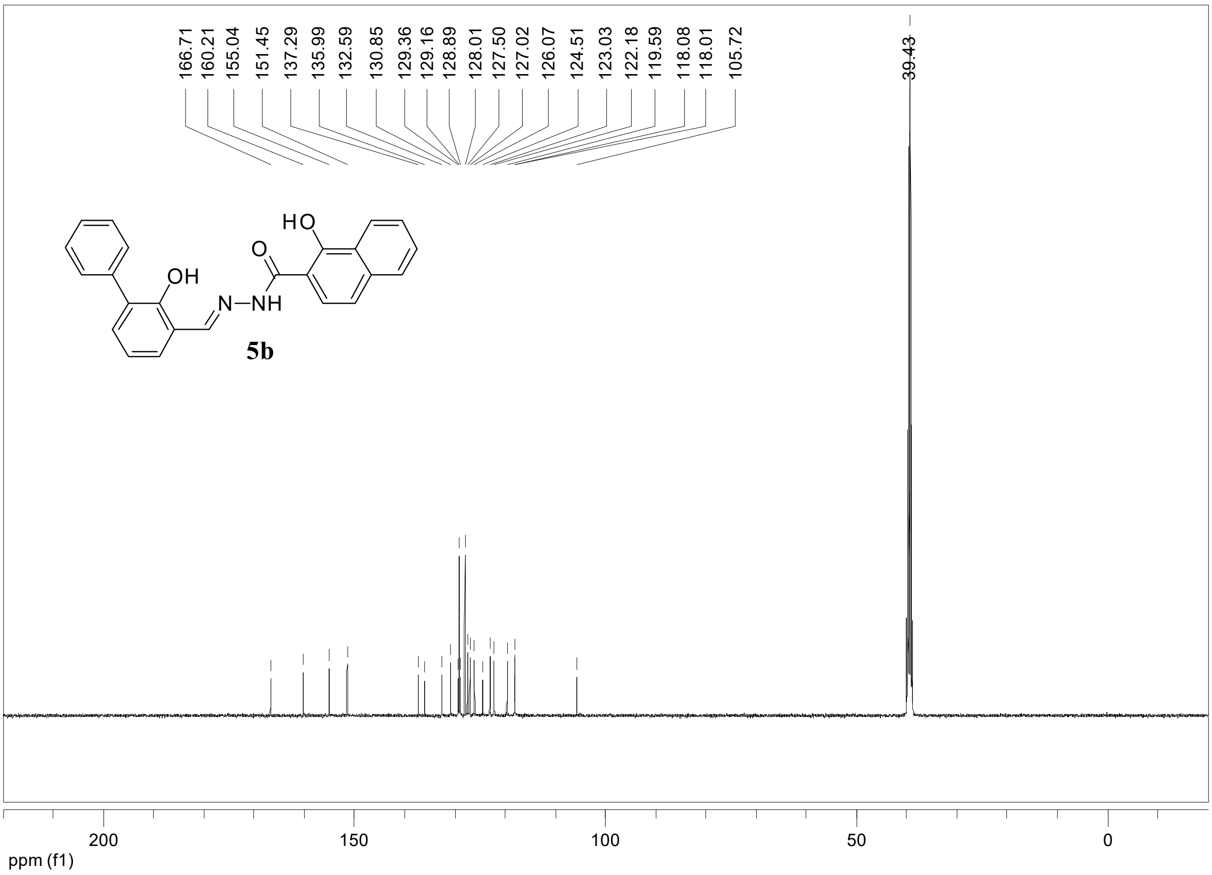

Figure S257. ^{13}C-NMR (100 MHz, DMSO-d_6) spectrum of compound **5b**

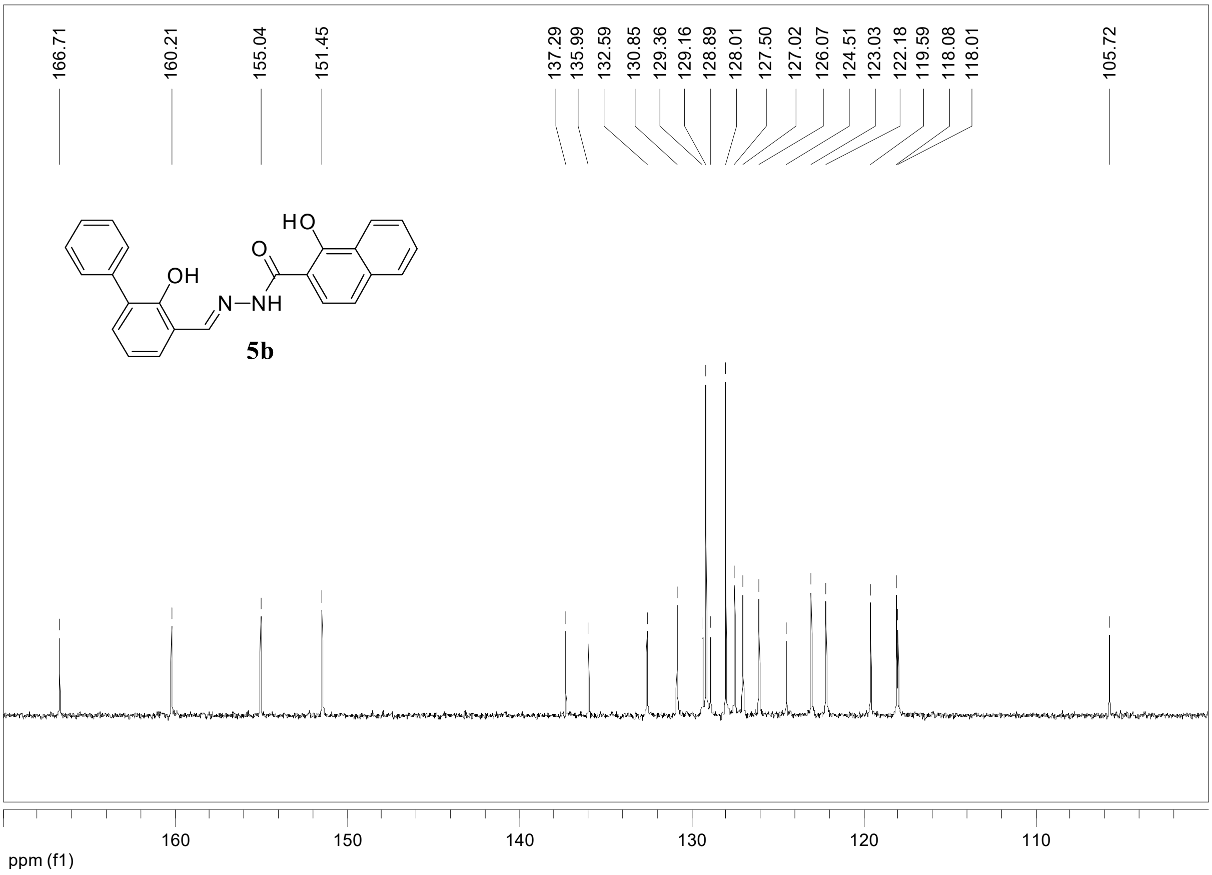

Figure S258. Expansion of ^{13}C-NMR (100 MHz, DMSO-d_6) spectrum of compound **5b**

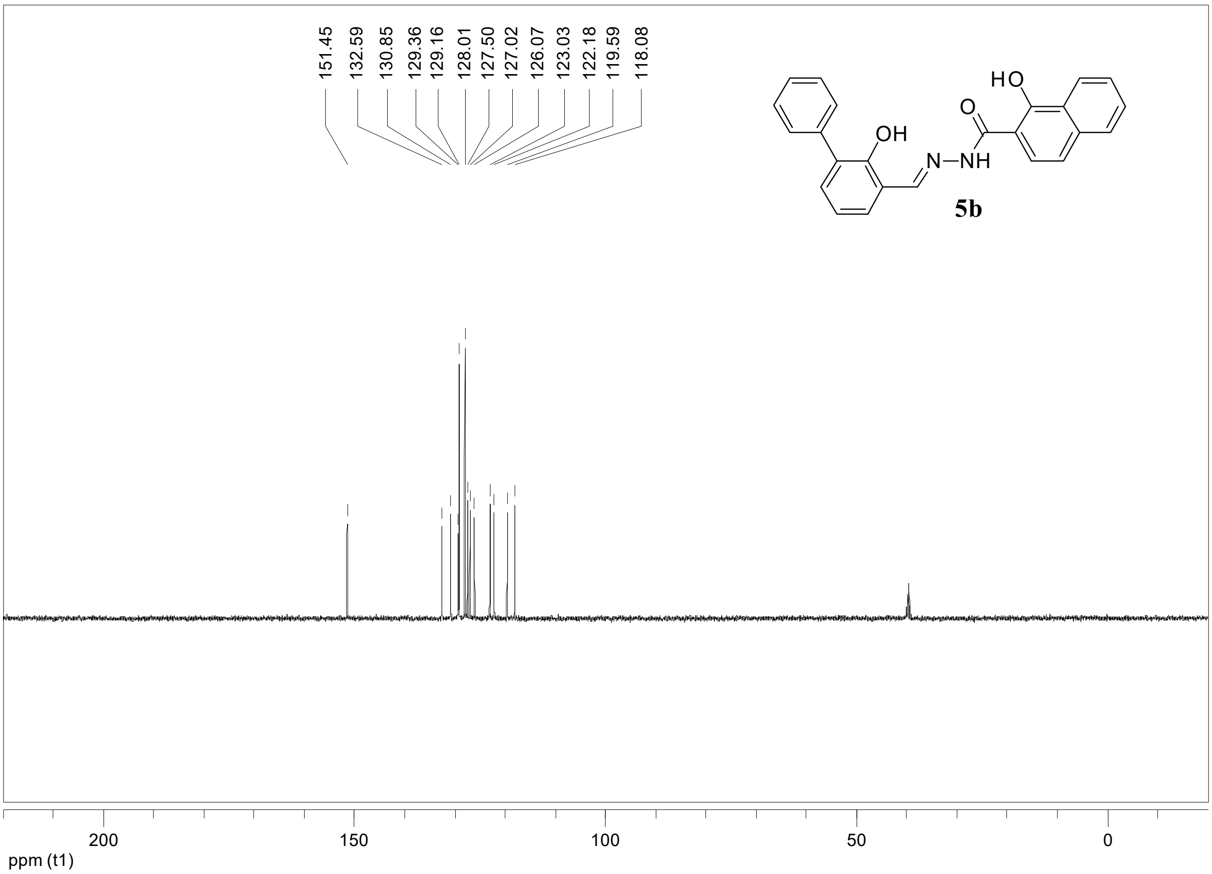

Figure S259. ^{13}C-NMR (100 MHz, DMSO-d_6) dept-135 experiment of compound **5b**

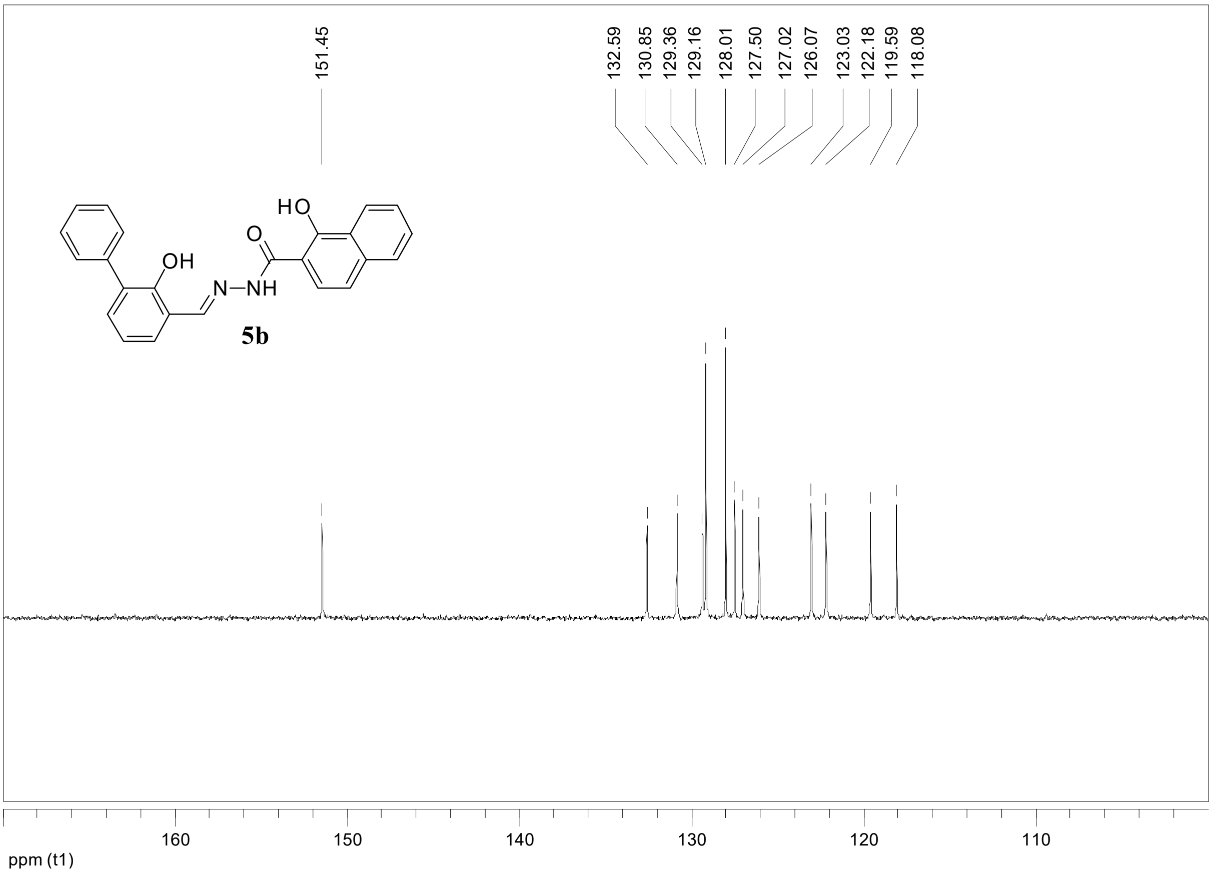

Figure S260. Expansion of ^{13}C-NMR (100 MHz, DMSO-d_6) dept-135 experiment of compound **5b**

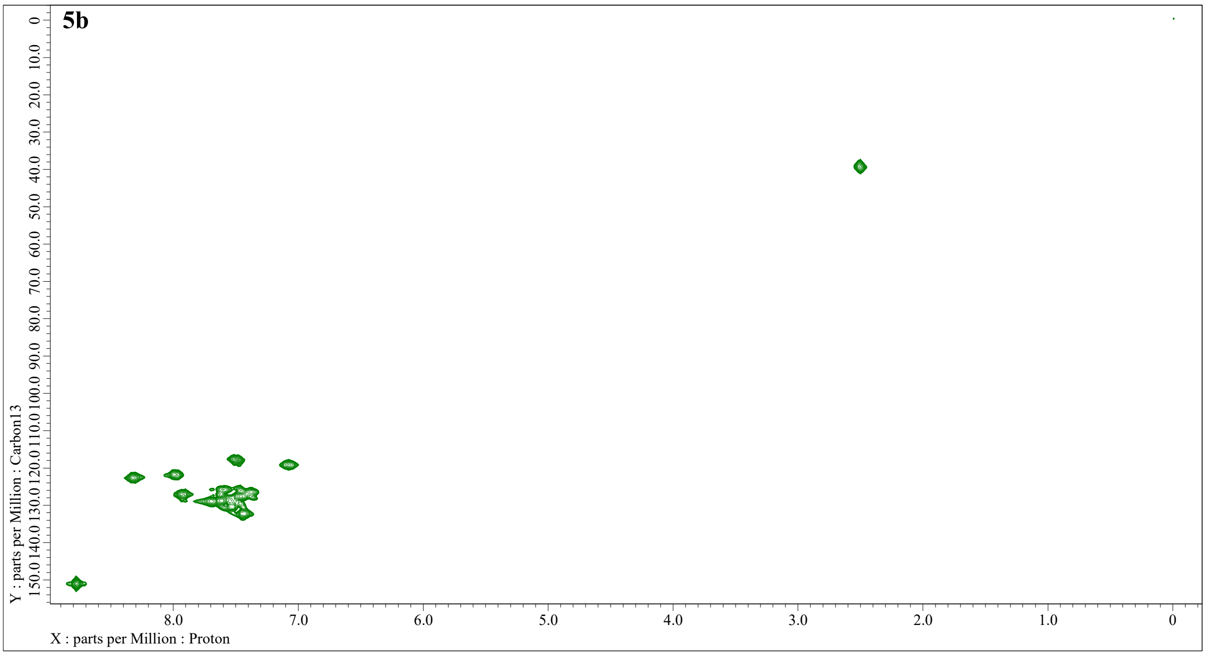

Figure S261. 2D-NMR (400 MHz, DMSO-d_6) HMQC experiment of *N*'-[(*E*)-(2-hydroxy-3-phenylphenyl)methylidene]-2-(1-hydroksynaphtho)hydrazide (**5b**)

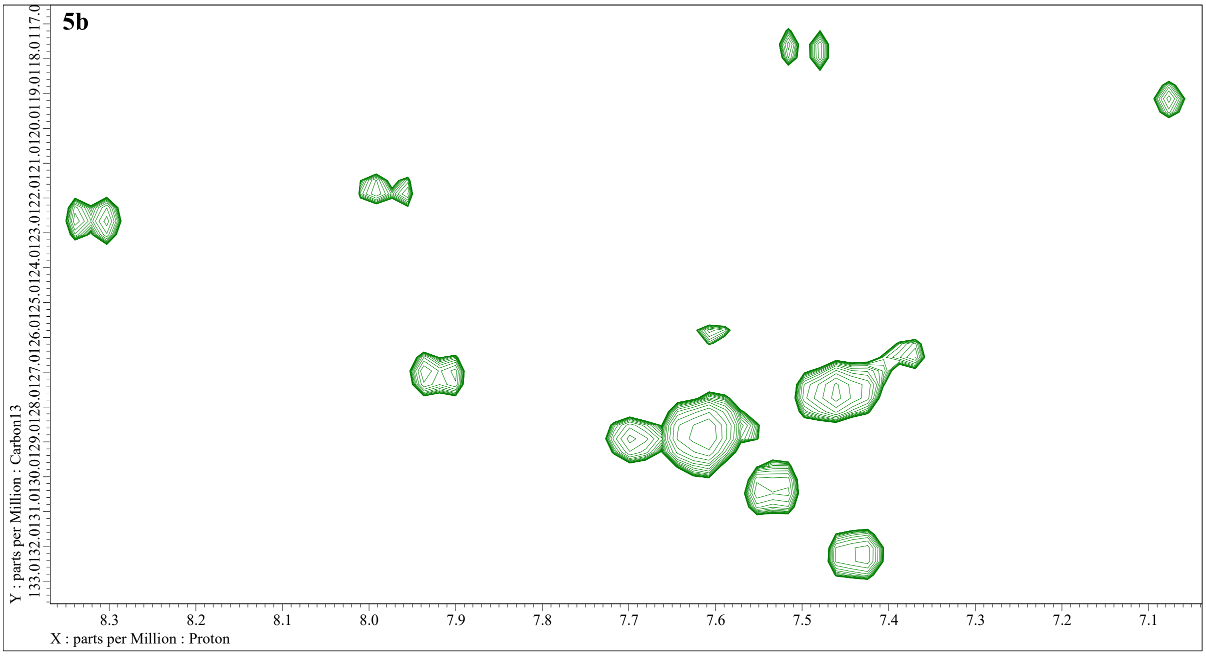

Figure S262. Expansion of 2D-NMR (400 MHz, DMSO-d_6) HMQC experiment of *N*'-[(*E*)-(2-hydroxy-3-phenylphenyl)methylidene]-2-(1-hydroksynaphtho)hydrazide (**5b**)

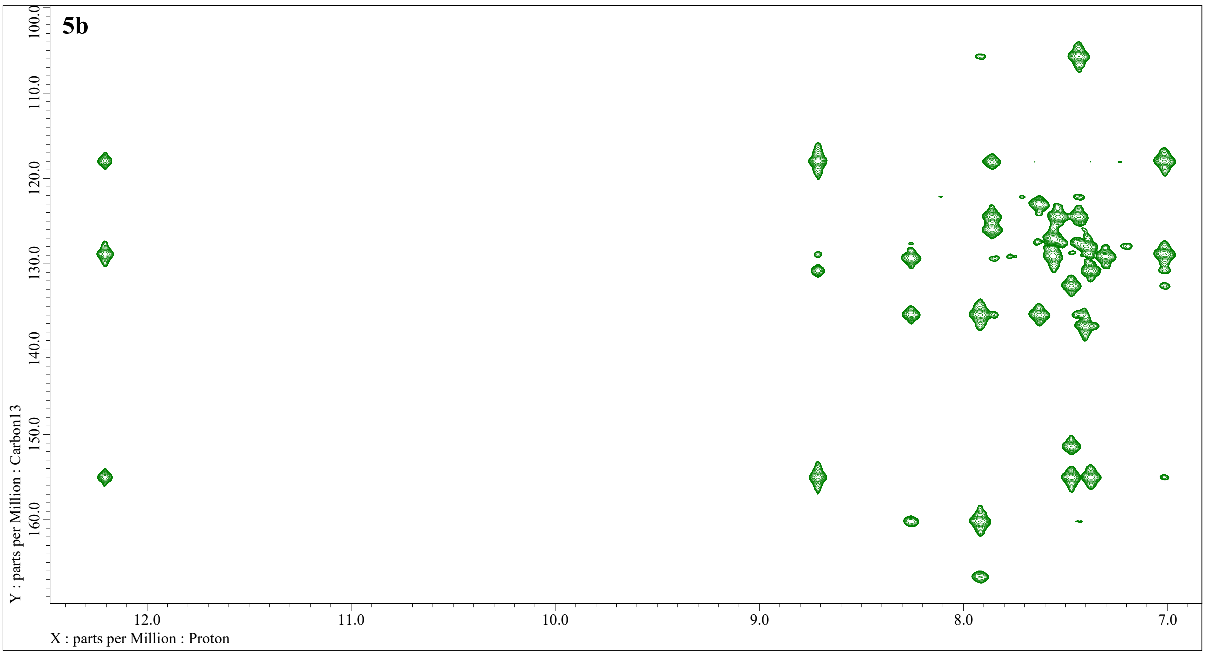

Figure S263. 2D-NMR (400 MHz, DMSO-d_6) HMBC experiment of *N'*-[(*E*)-(2-hydroxy-3-phenylphenyl)methylidene]-2-(1-hydroksynaphtho)hydrazide (**5b**)

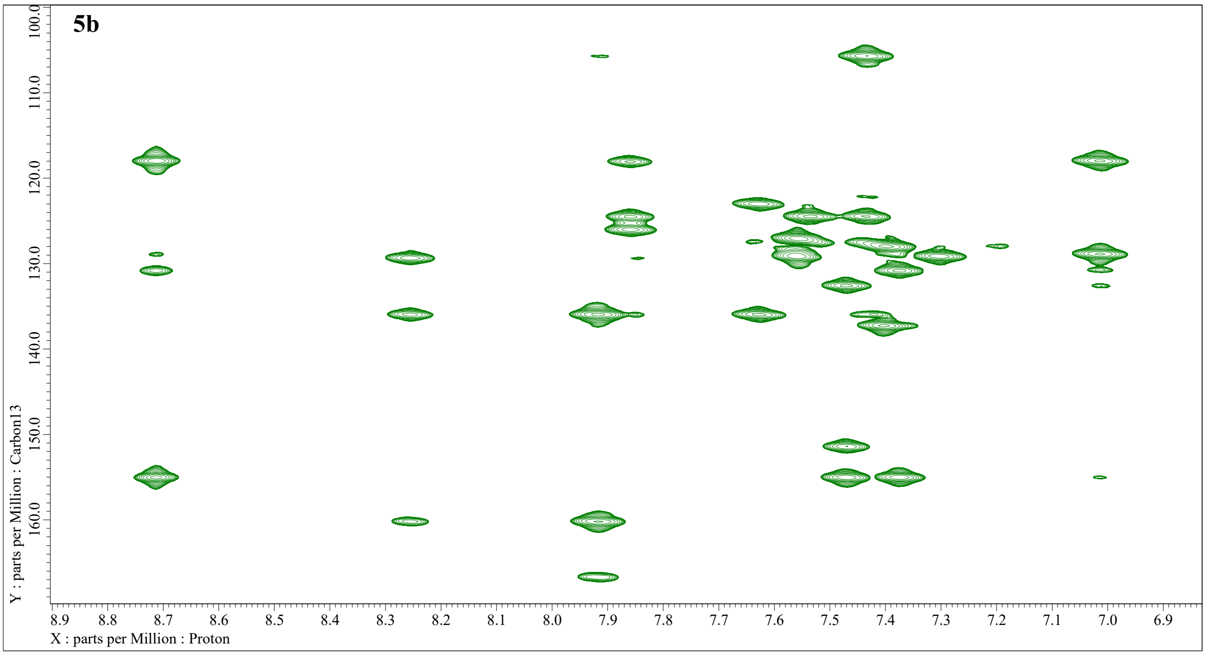

Figure S264. Expansion of 2D-NMR (400 MHz, DMSO-d_6) HMBC experiment of *N'*-[(*E*)-(2-hydroxy-3-phenylphenyl)methylidene]-2-(1-hydroksynaphtho)hydrazide (**5b**)

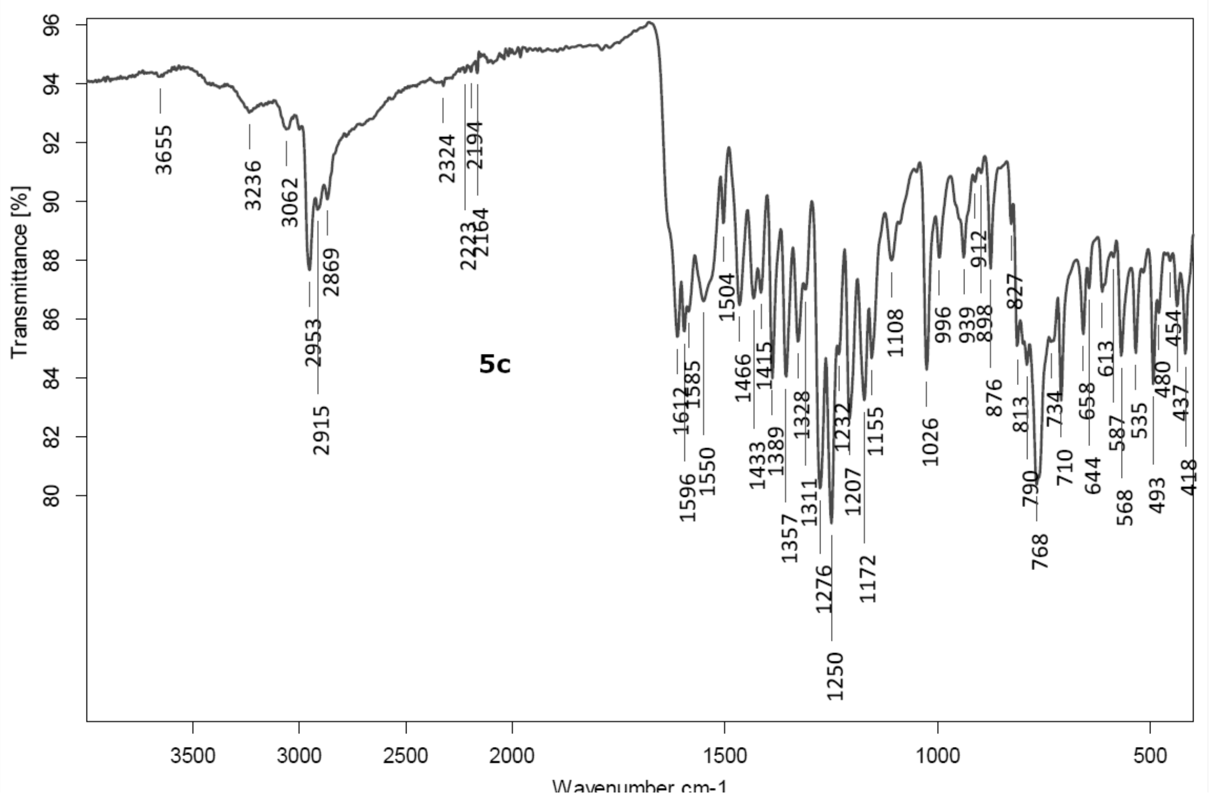

Figure S265. FT-IR (ATR) spectrum of *N'*-[(*E*)-(3,5-di-*tert*-butyl-2-hydroxy-phenyl)methylidene]-2-(1-hydroksynaphtho)hydrazide monomethanolate (**5c**)

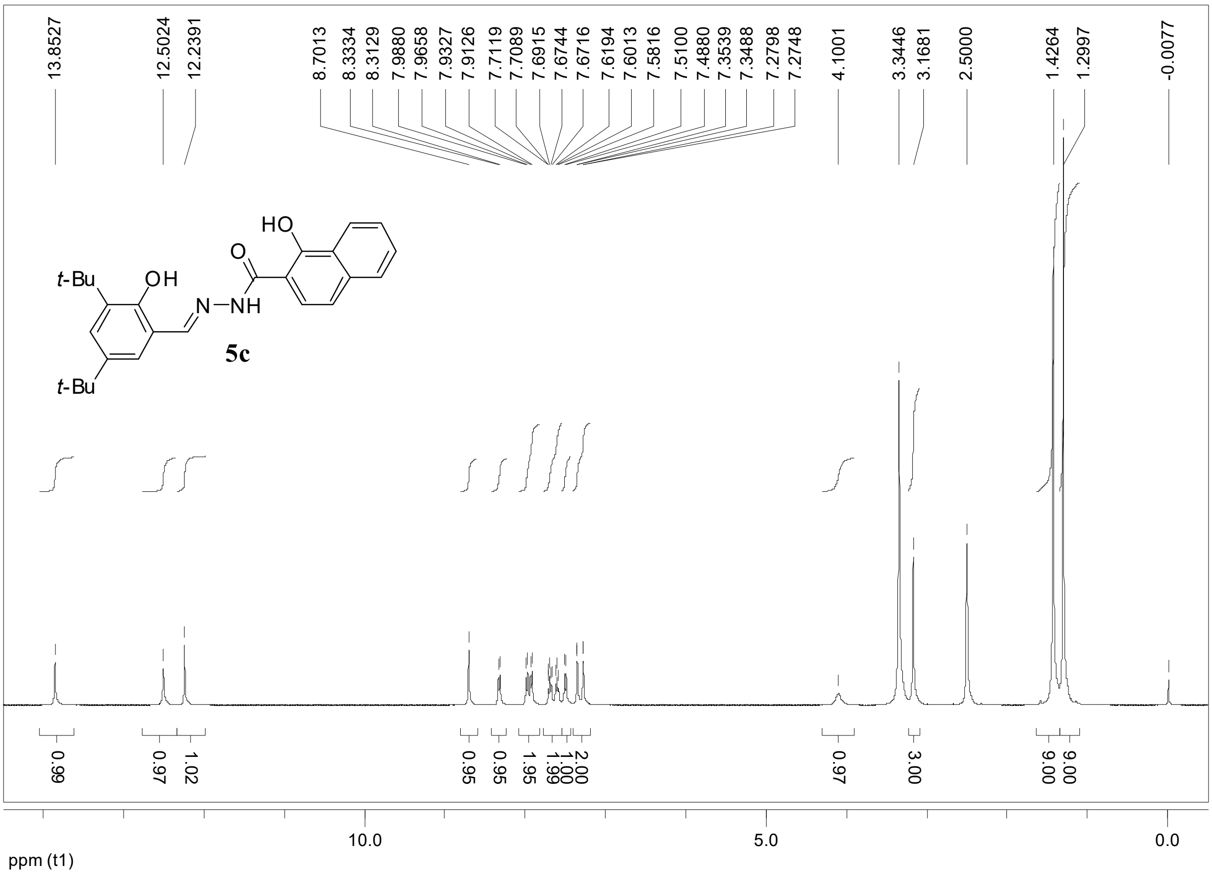

Figure S266. ^{1}H-NMR (400 MHz, DMSO-d_6) spectrum of compound **5c**

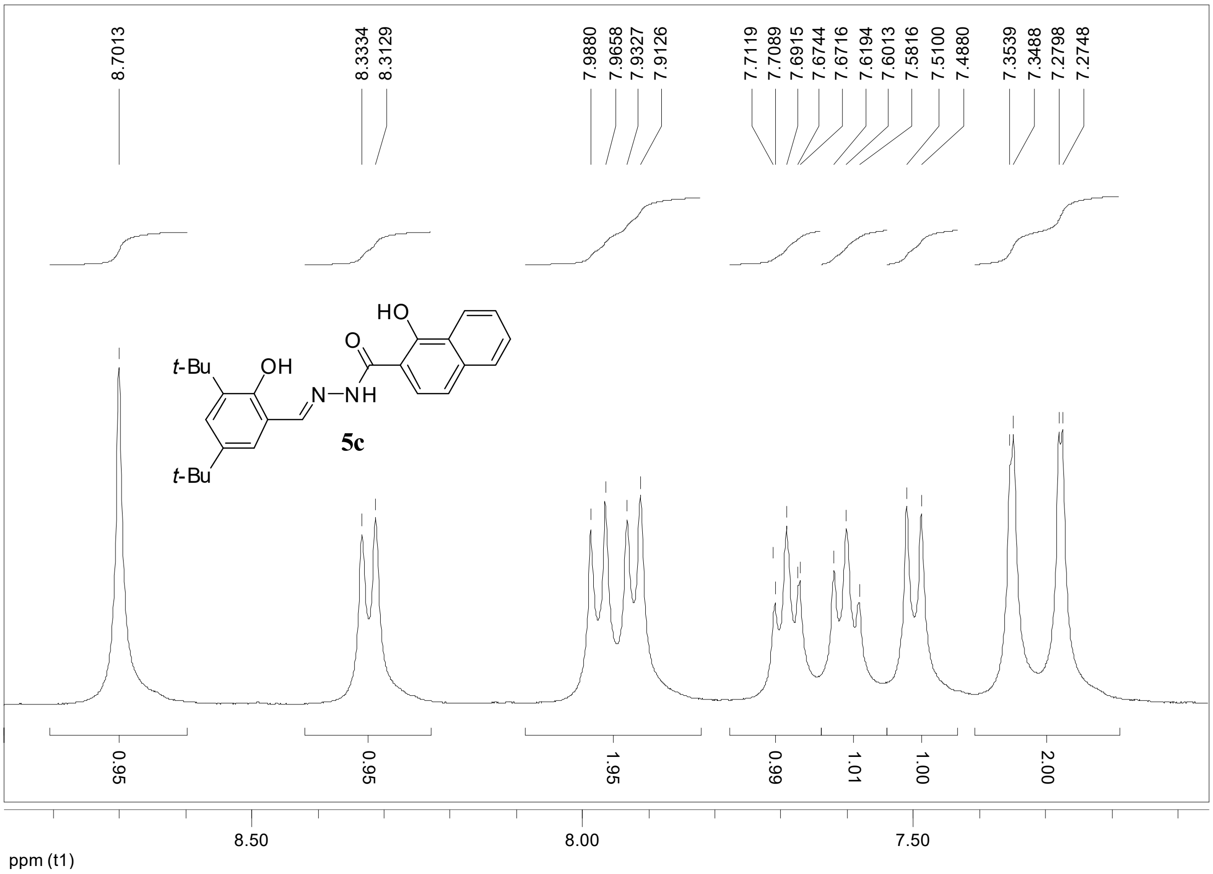

Figure S267. Expansion of ^{1}H-NMR (400 MHz, DMSO-d_6) spectrum of compound **5c**

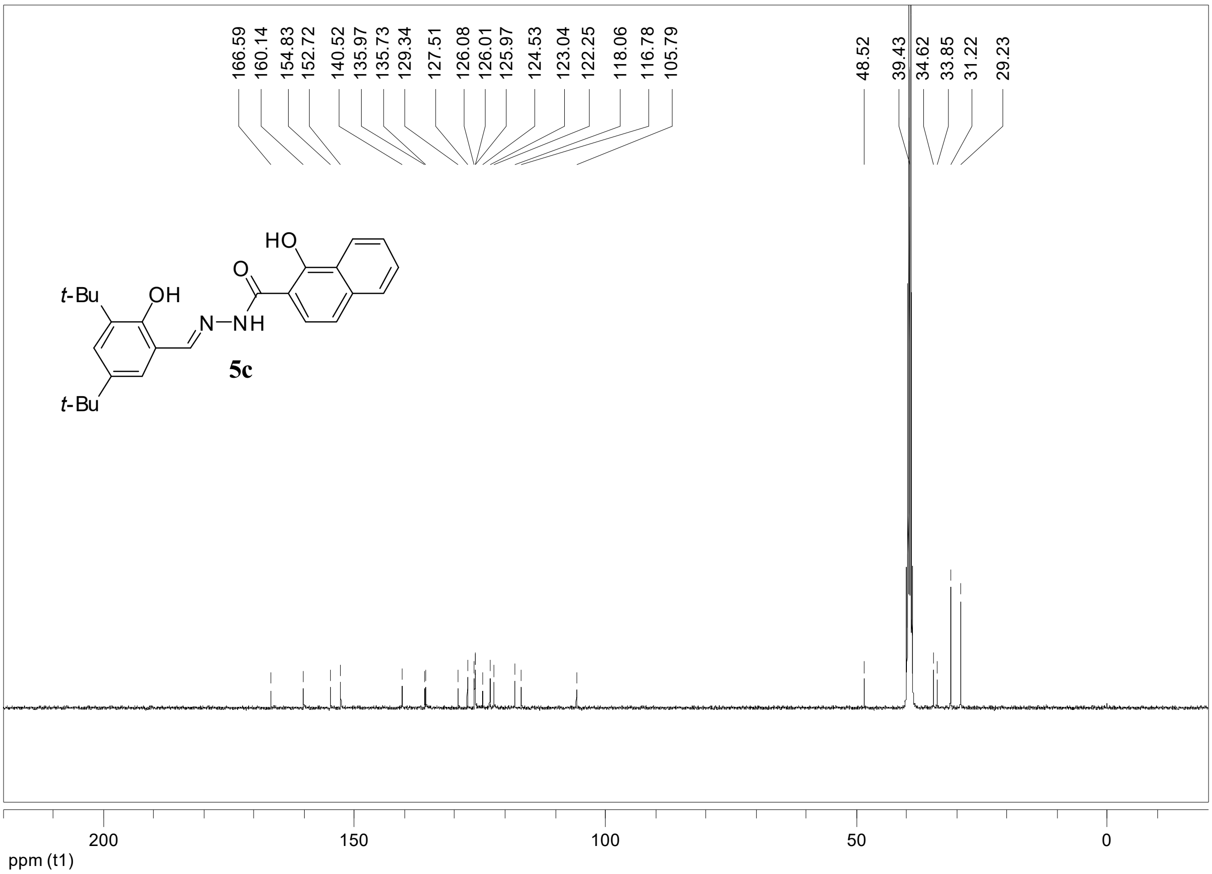

Figure S268. ^{13}C-NMR (100 MHz, DMSO-d_6) spectrum of compound **5c**

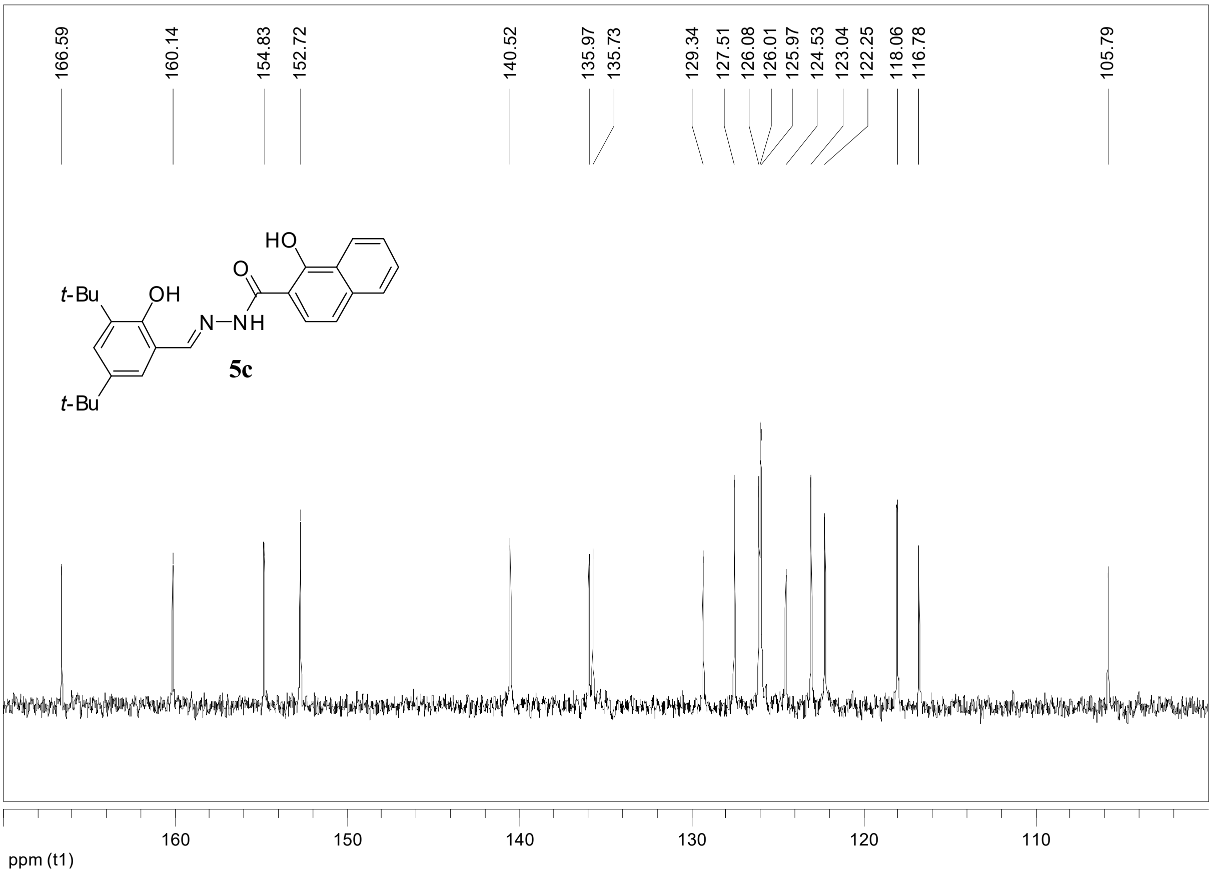

Figure S269. Expansion of ^{13}C-NMR (100 MHz, DMSO-d_6) spectrum of compound **5c**

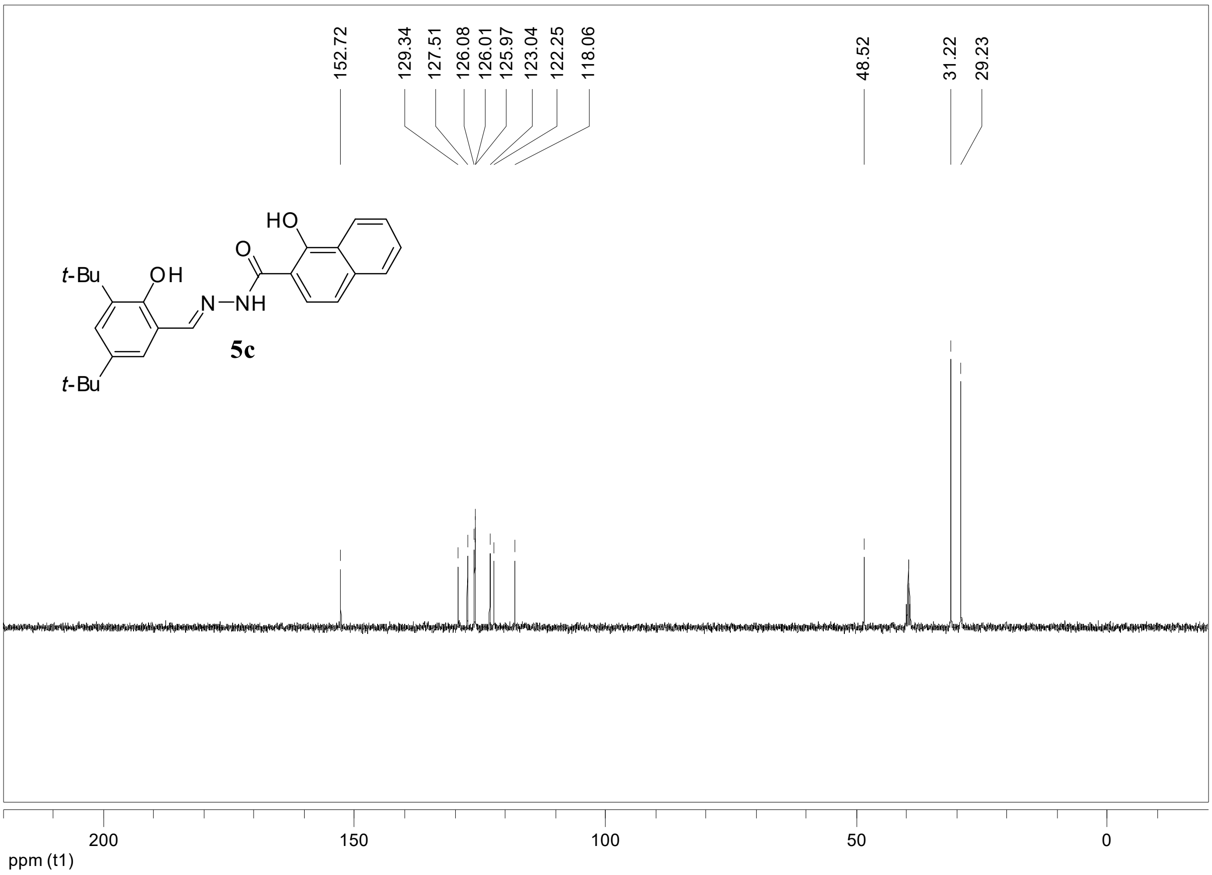

Figure S270. ^{13}C-NMR (100 MHz, DMSO-d_6) dept-135 experiment of compound **5c**

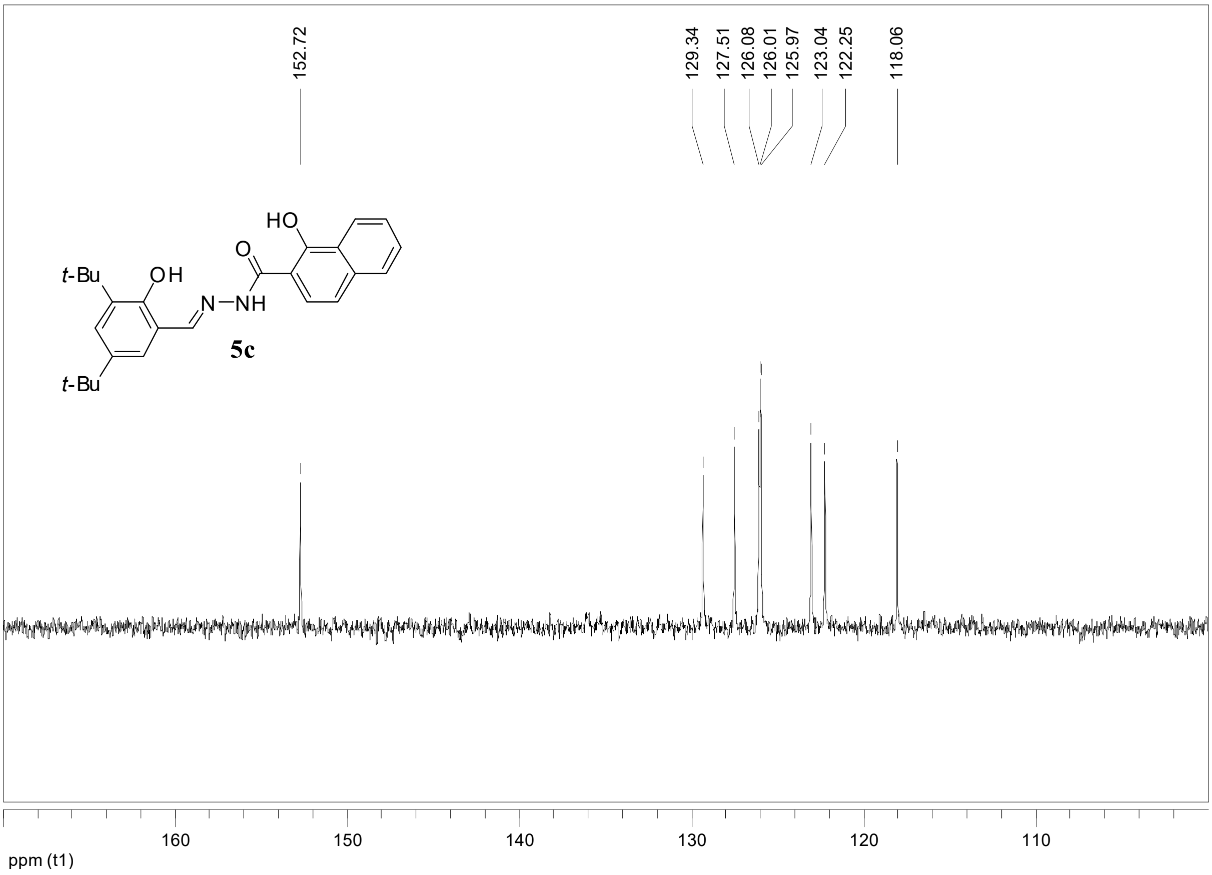

Figure S271. Expansion of ^{13}C-NMR (100 MHz, DMSO-d_6) dept-135 experiment of compound **5c**

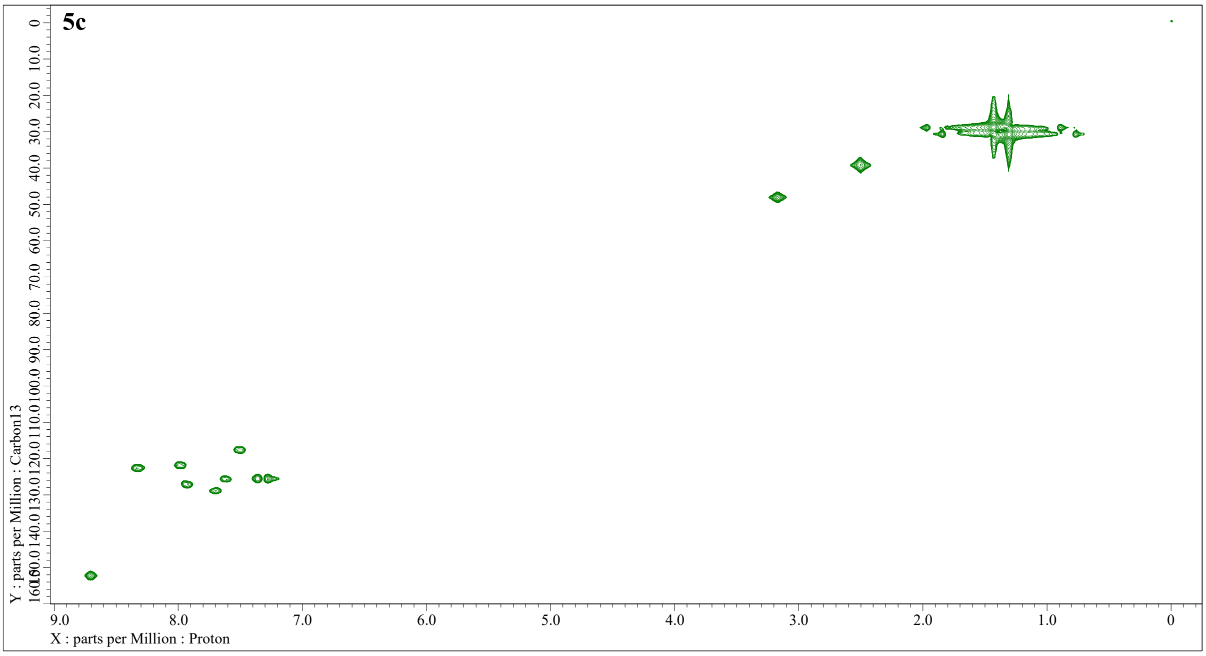

Figure S272. 2D-NMR (400 MHz, DMSO-d_6) HMQC experiment of *N*'-[(*E*)-(3,5-di-*tert*-butyl-2-hydroxy-phenyl)methylidene]-2-(1-hydroksynaphtho)hydrazide monomethanolate (**5c**)

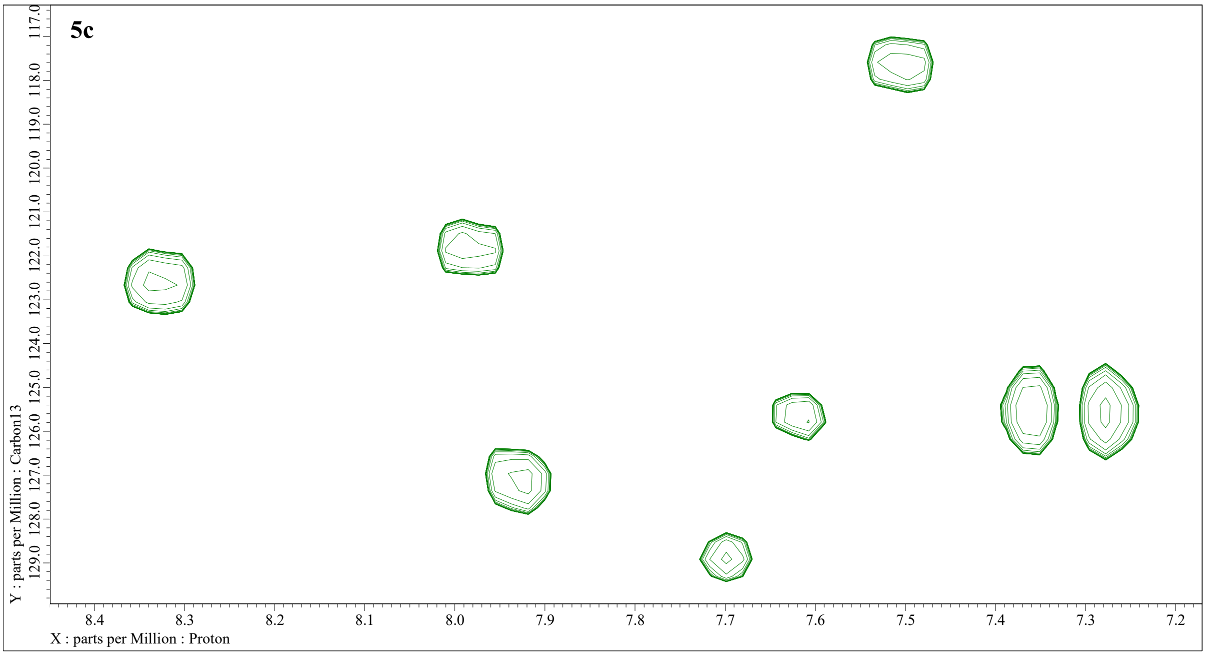

Figure S273. Expansion of 2D-NMR (400 MHz, DMSO-d_6) HMQC experiment of *N*'-[(*E*)-(3,5-di-*tert*-butyl-2-hydroxy-phenyl)methylidene]-2-(1-hydroksynaphtho)hydrazide monomethanolate (**5c**)

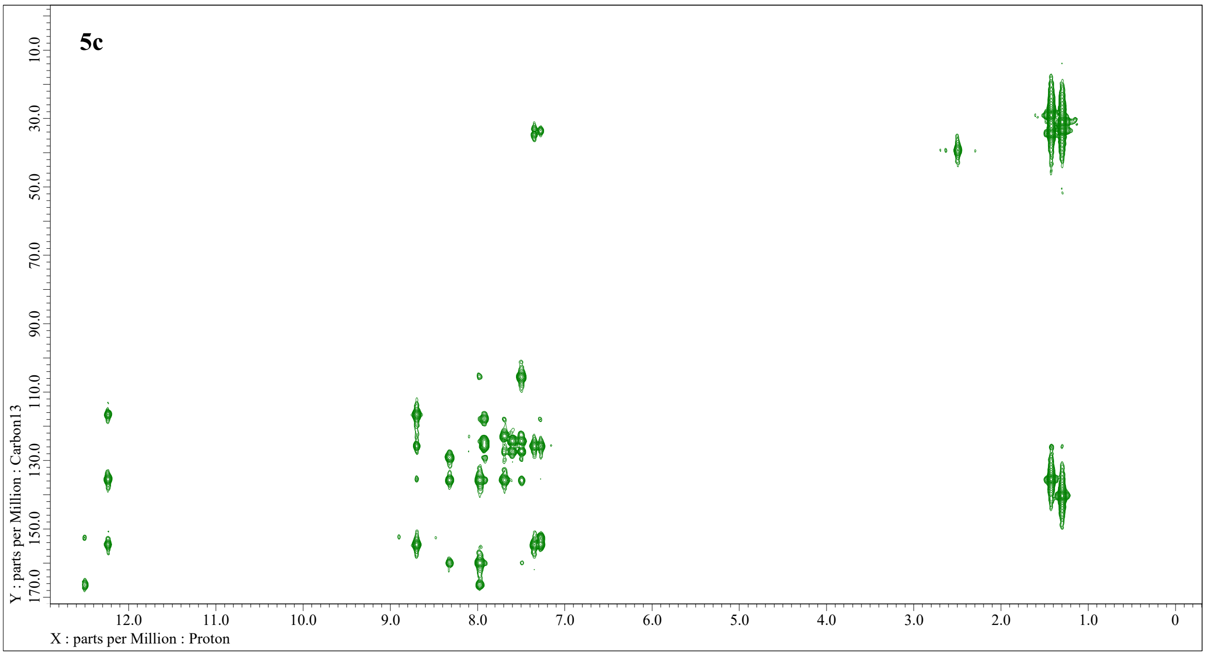

Figure S274. 2D-NMR (400 MHz, DMSO-d_6) HMBC experiment of *N*'-[(*E*)-(3,5-di-*tert*-butyl-2-hydroxy-phenyl)methylidene]-2-(1-hydroksynaphtho)hydrazide monomethanolate (**5c**)

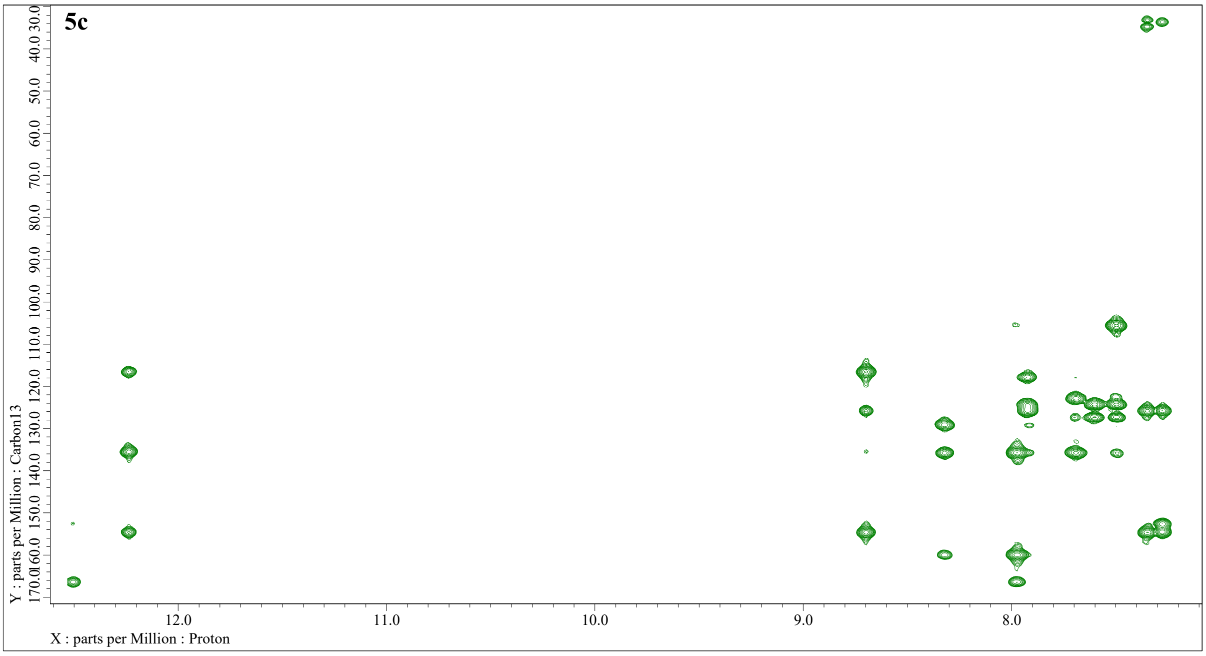

Figure S275. Expansion of 2D-NMR (400 MHz, DMSO-d_6) HMBC experiment of *N*'-[(*E*)-(3,5-di-*tert*-butyl-2-hydroxy-phenyl)methylidene]-2-(1-hydroksynaphtho)hydrazide monomethanolate (**5c**)

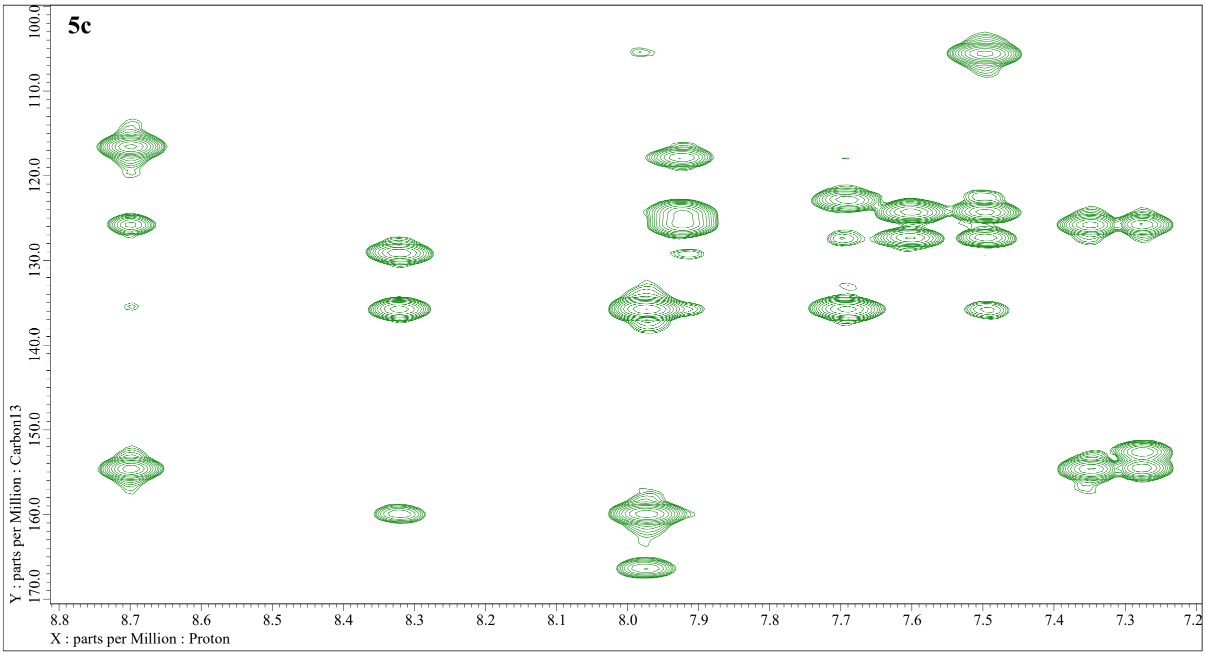

Figure S276. Expansion of 2D-NMR (400 MHz, DMSO-d_6) HMBC experiment of *N'*-[(*E*)-(3,5-di-*tert*-butyl-2-hydroxy-phenyl)methylidene]-2-(1-hydroksynaphtho)hydrazide monomethanolate (**5c**)

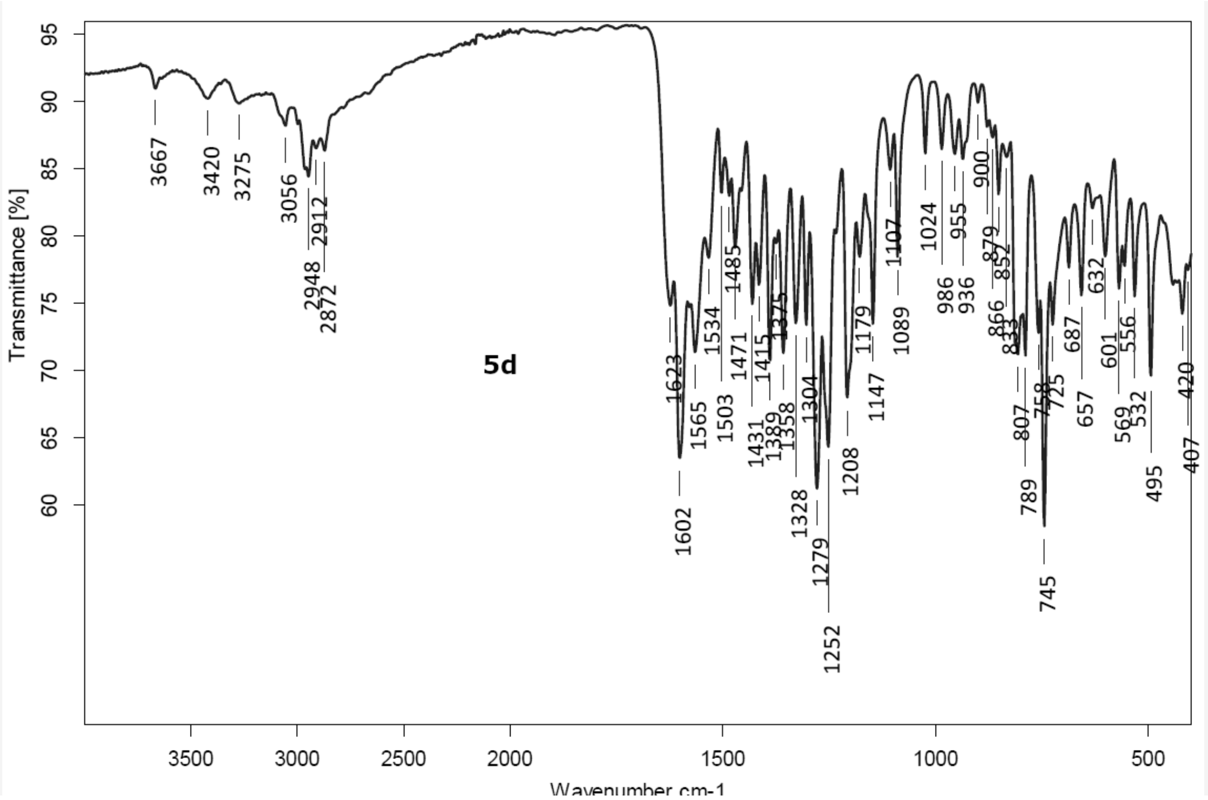

Figure S277. FT-IR (ATR) spectrum of *N'*-[(*E*)-(3-*tert*-butyl-2-hydroxy-phenyl)methylidene]-2-(1-hydroksynaphtho)hydrazide (**5d**)

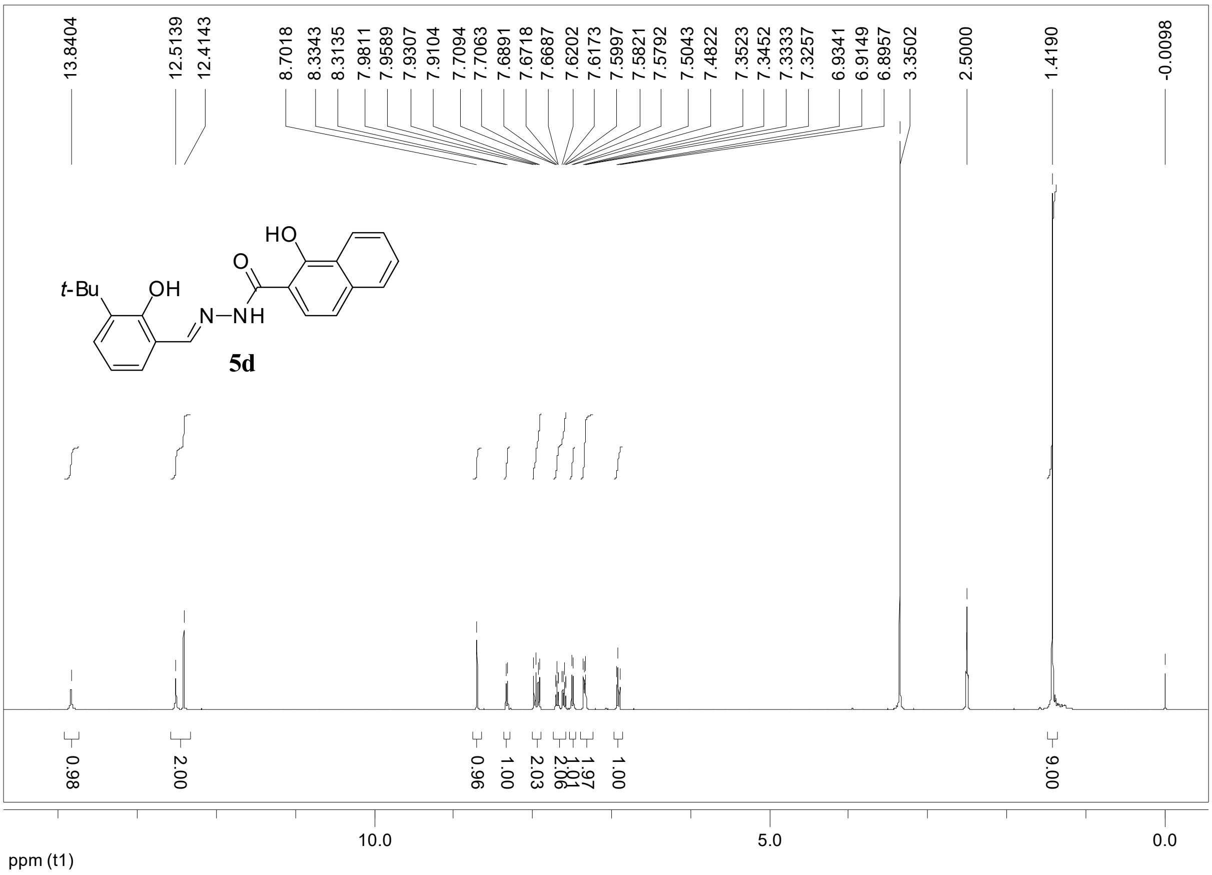

Figure S278. ^{1}H-NMR (400 MHz, DMSO-d_6) spectrum of compound **5d**

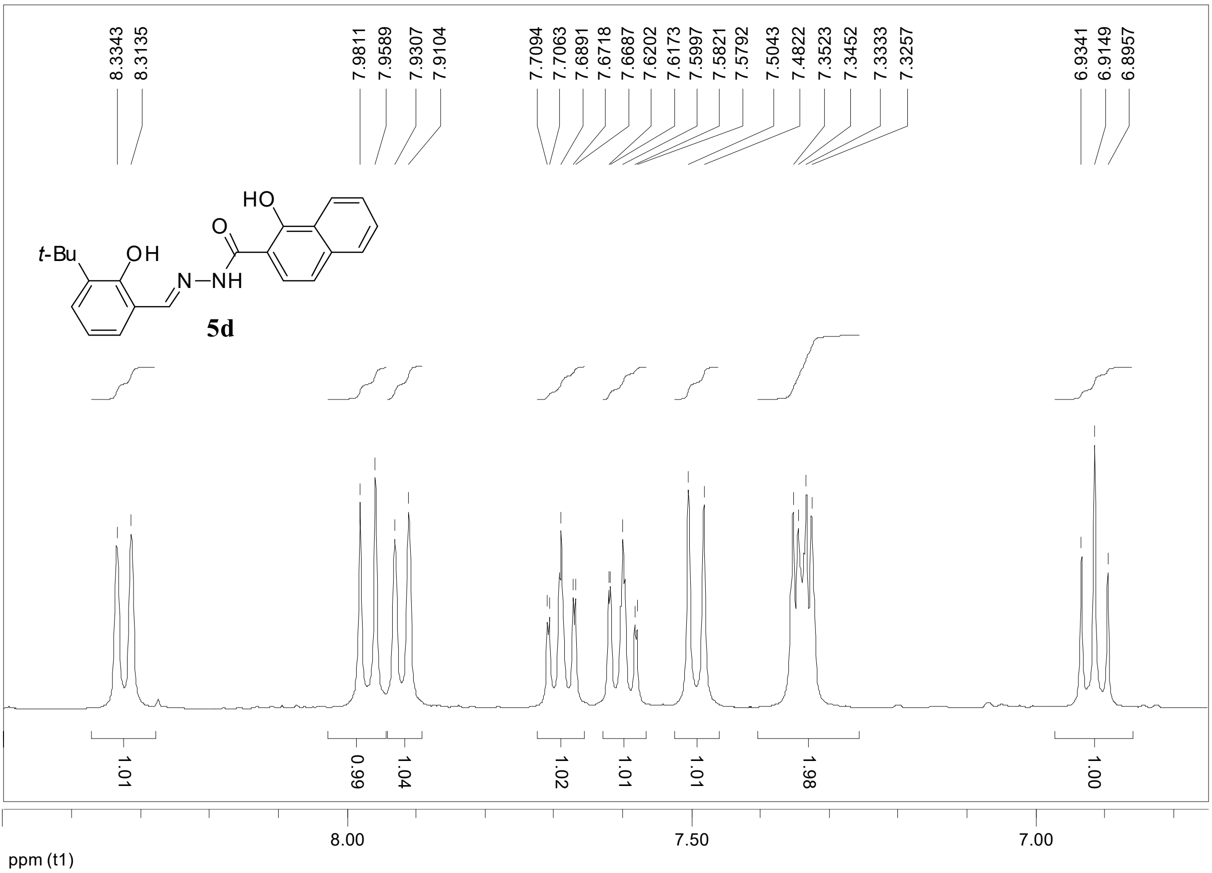

Figure S279. Expansion of ^{1}H-NMR (400 MHz, DMSO-d_6) spectrum of compound **5d**

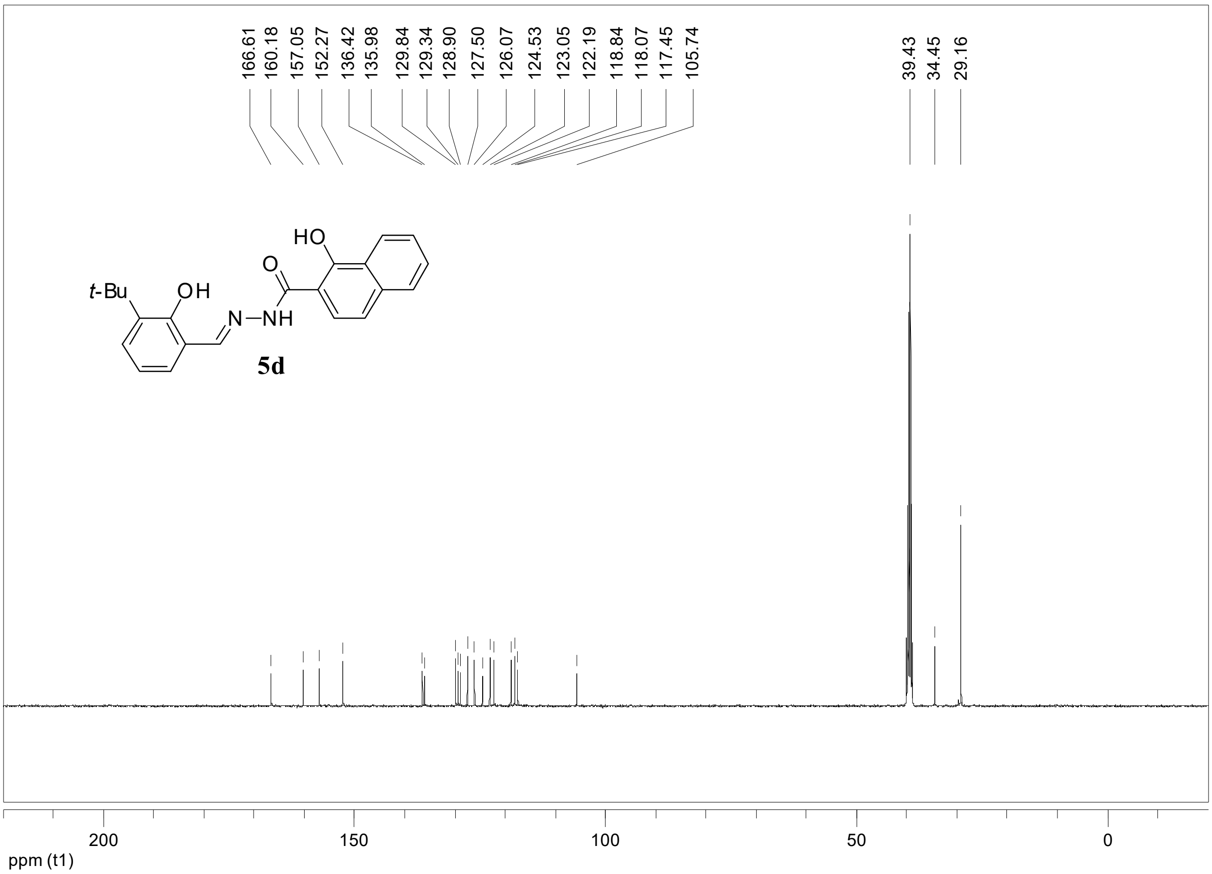

Figure S280. ^{13}C-NMR (100 MHz, DMSO-d_6) spectrum of compound **5d**

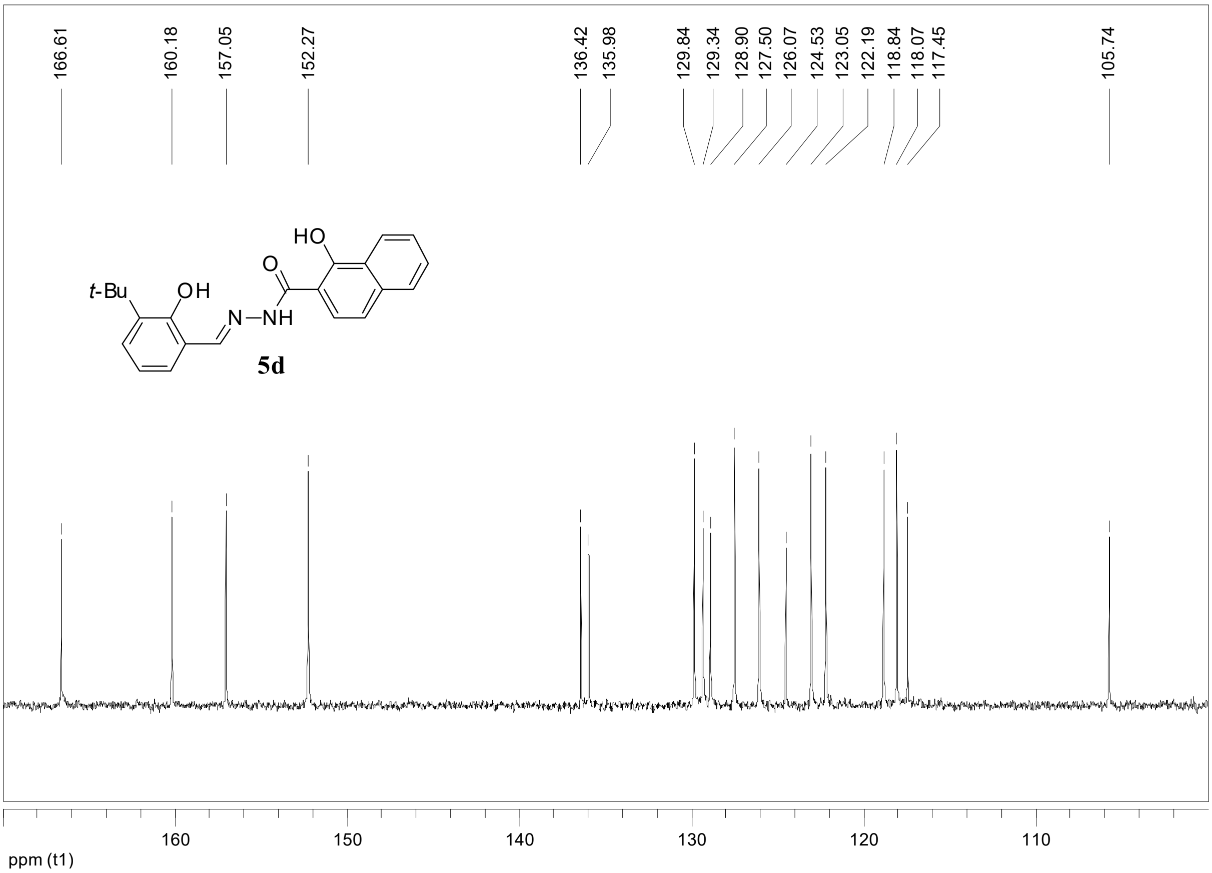

Figure S281. Expansion of ^{13}C-NMR (100 MHz, DMSO-d_6) spectrum of compound **5d**

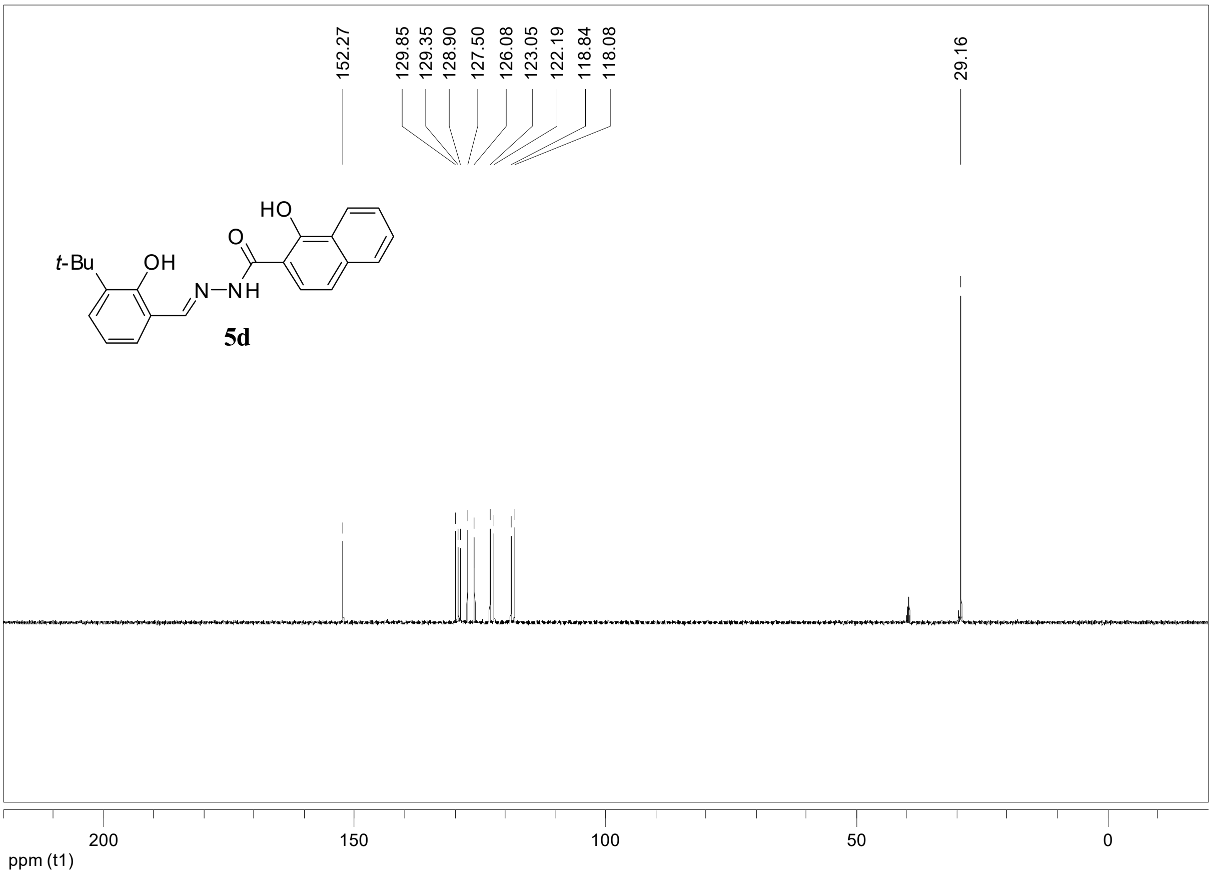

Figure S282. ^{13}C-NMR (100 MHz, DMSO-d_6) dept-135 experiment of compound **5d**

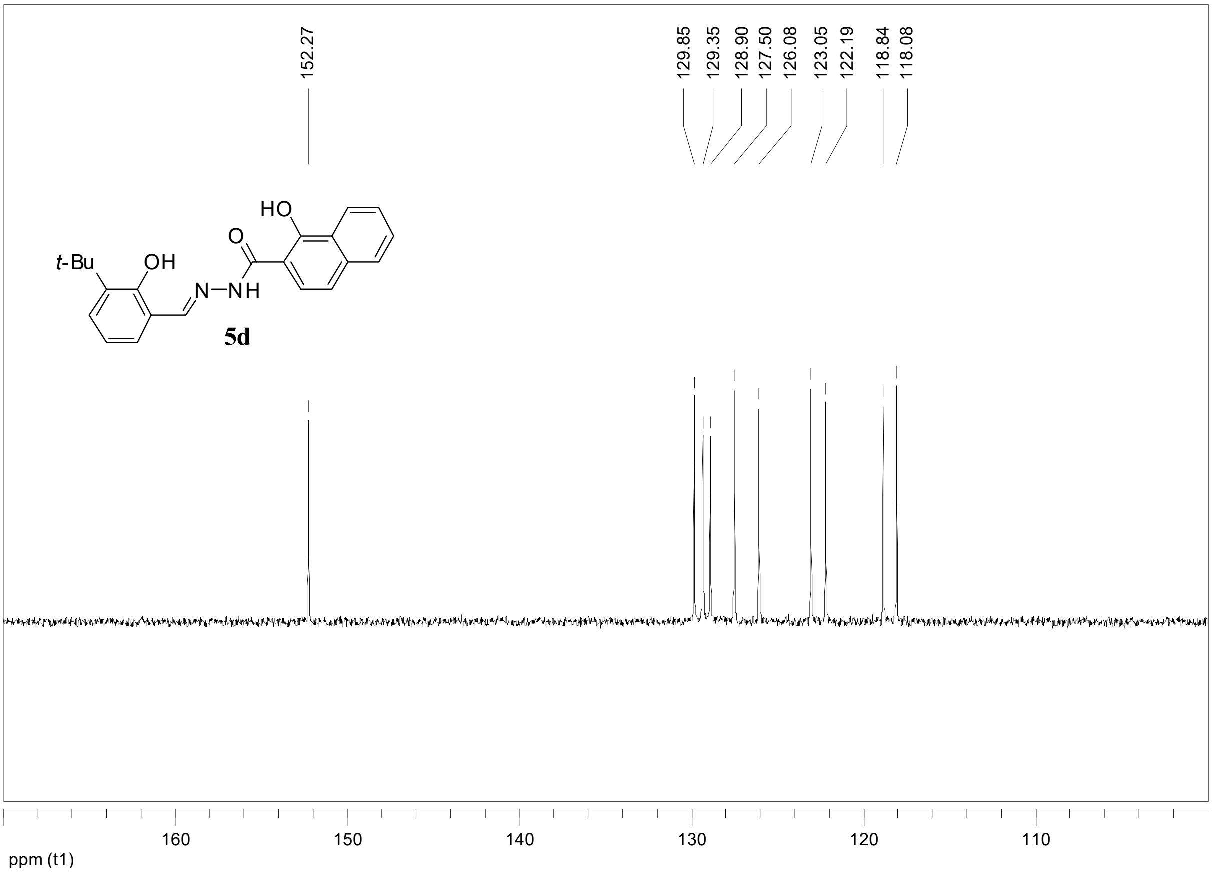

Figure S283. Expansion of ^{13}C-NMR (100 MHz, DMSO-d_6) dept-135 experiment of compound **5d**

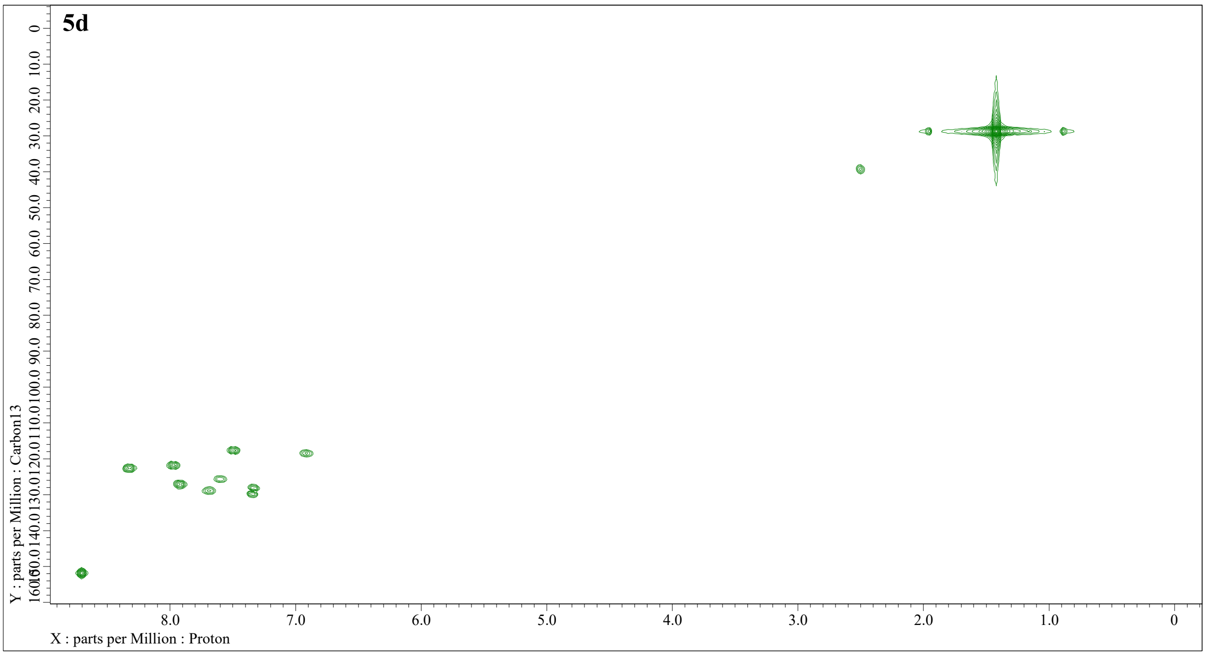

Figure S284. 2D-NMR (400 MHz, DMSO-d_6) HMQC experiment of *N*'-[(*E*)-(3-*tert*-butyl-2-hydroxy-phenyl)methylidene]-2-(1-hydroksynaphtho)hydrazide (**5d**)

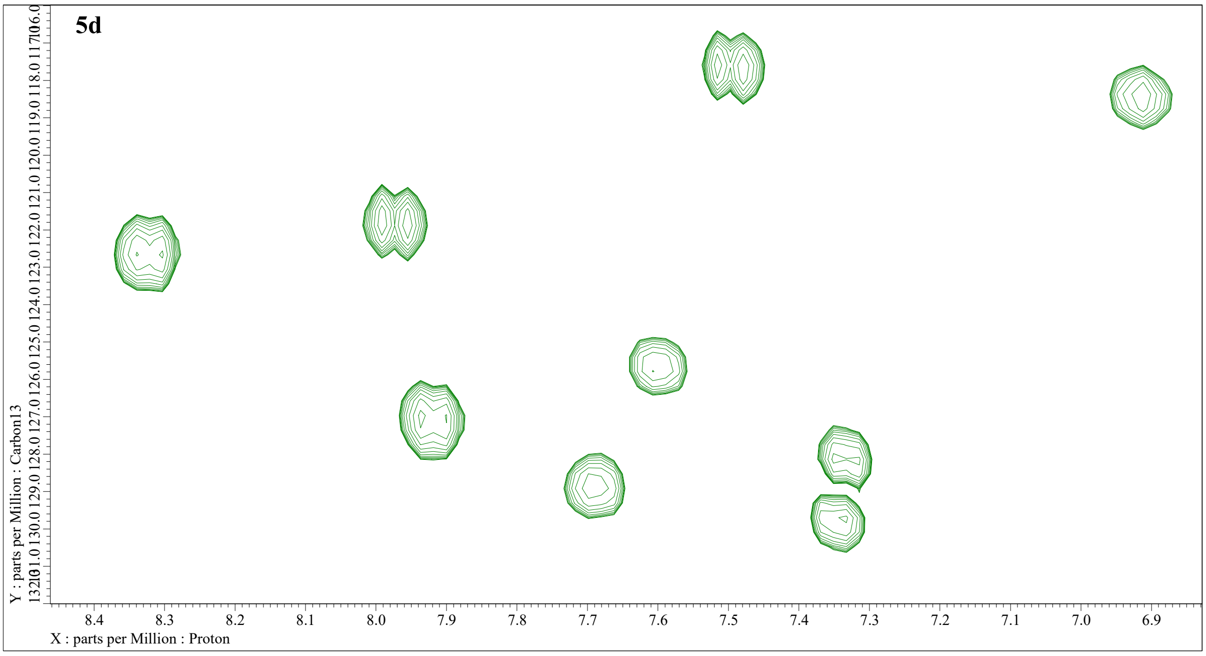

Figure S285. Expansion of 2D-NMR (400 MHz, DMSO-d_6) HMQC experiment of *N*'-[(*E*)-(3-*tert*-butyl-2-hydroxy-phenyl)methylidene]-2-(1-hydroksynaphtho)hydrazide (**5d**)

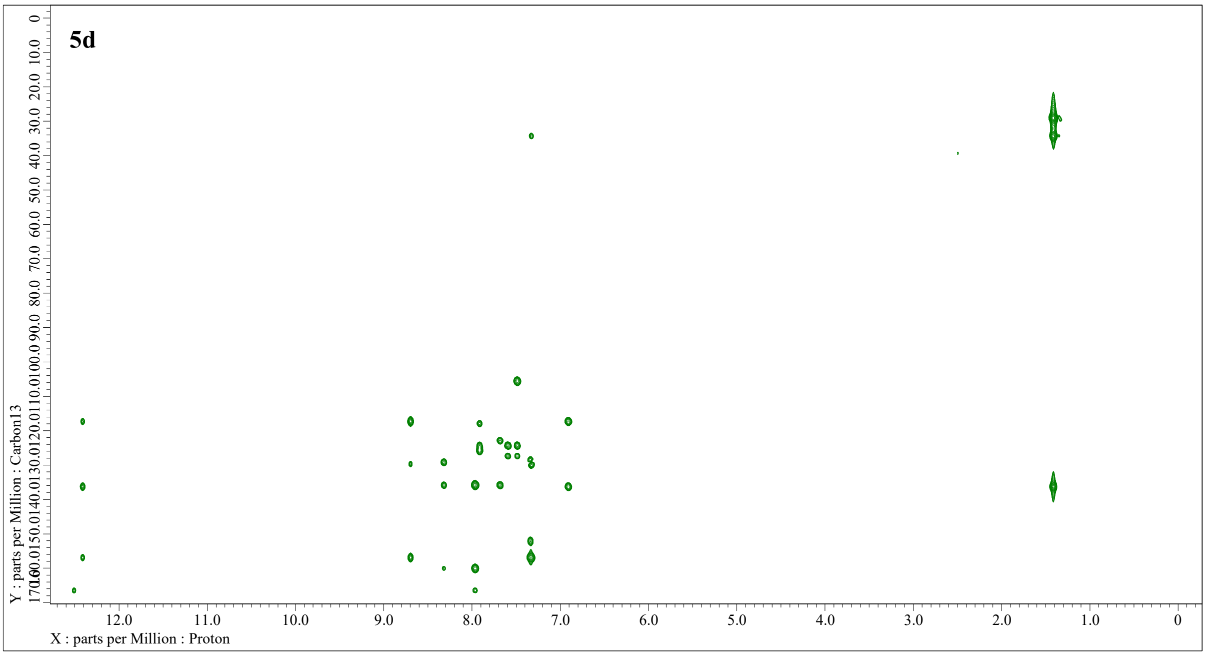

Figure S286. 2D-NMR (400 MHz, DMSO-d_6) HMBC experiment of *N'*-[(*E*)-(3-*tert*-butyl-2-hydroxy-phenyl)methylidene]-2-(1-hydroksynaphtho)hydrazide (**5d**)

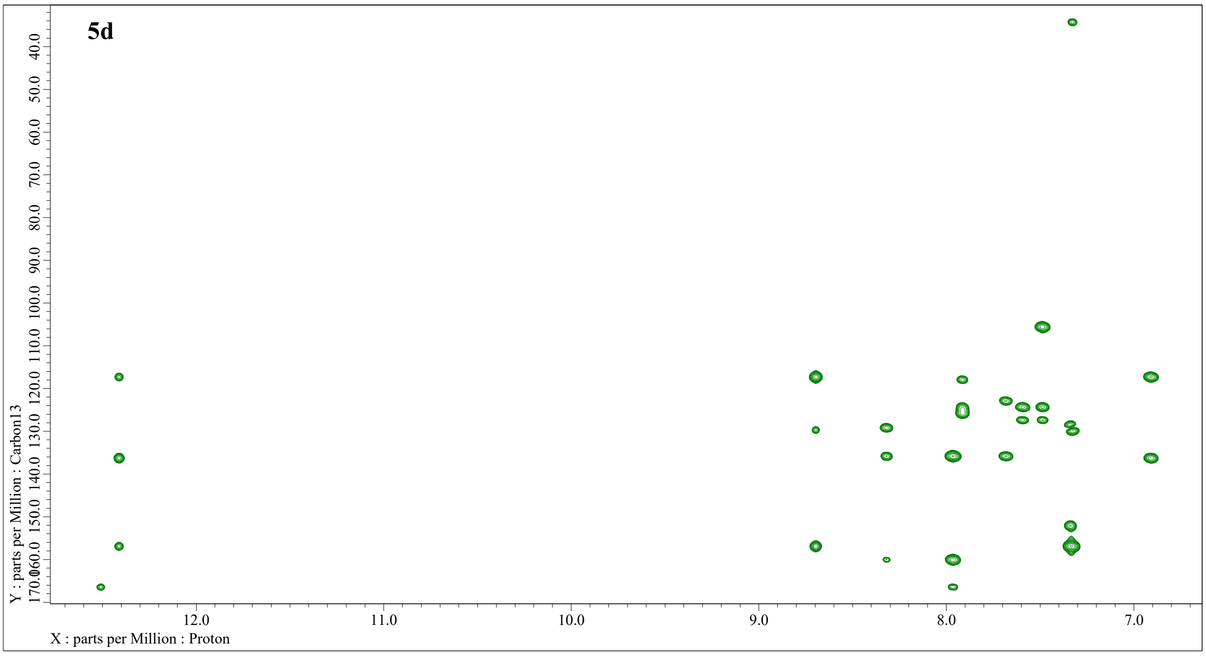

Figure S287. Expansion of 2D-NMR (400 MHz, DMSO-d_6) HMBC experiment of *N'*-[(*E*)-(3-*tert*-butyl-2-hydroxy-phenyl)methylidene]-2-(1-hydroksynaphtho)hydrazide (**5d**)

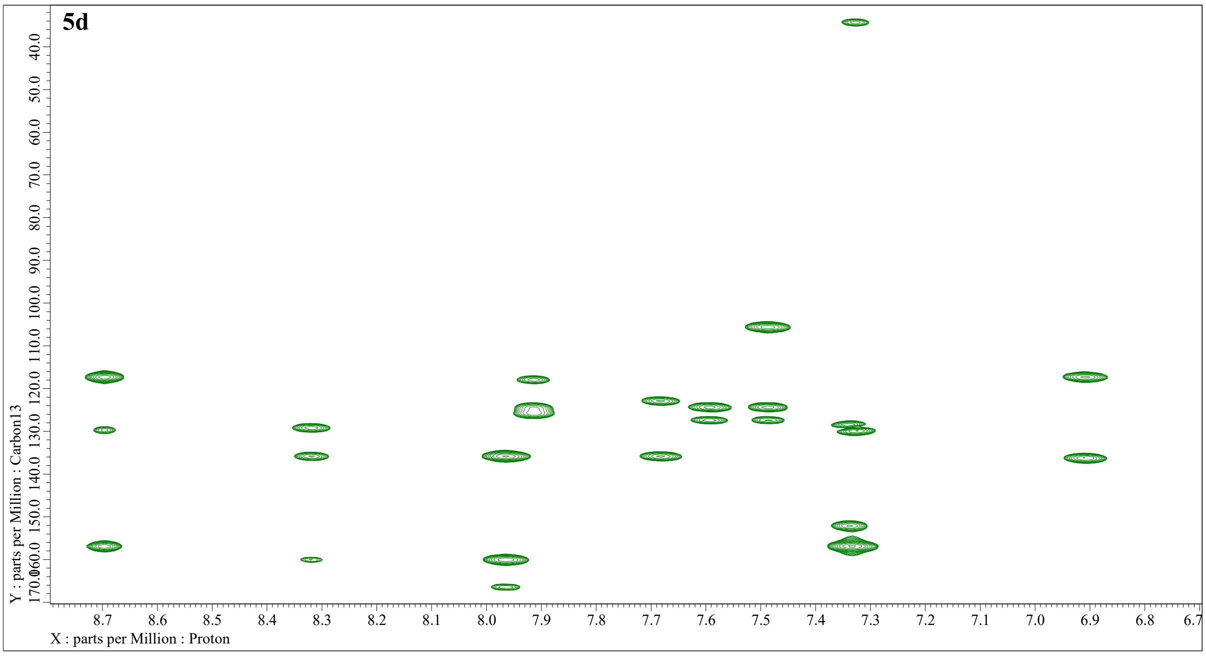

Figure S288. Expansion of 2D-NMR (400 MHz, DMSO-d_6) HMBC experiment of *N*'-[(*E*)-(3-*tert*-butyl-2-hydroxy-phenyl)methylidene]-2-(1-hydroksynaphtho)hydrazide (**5d**)

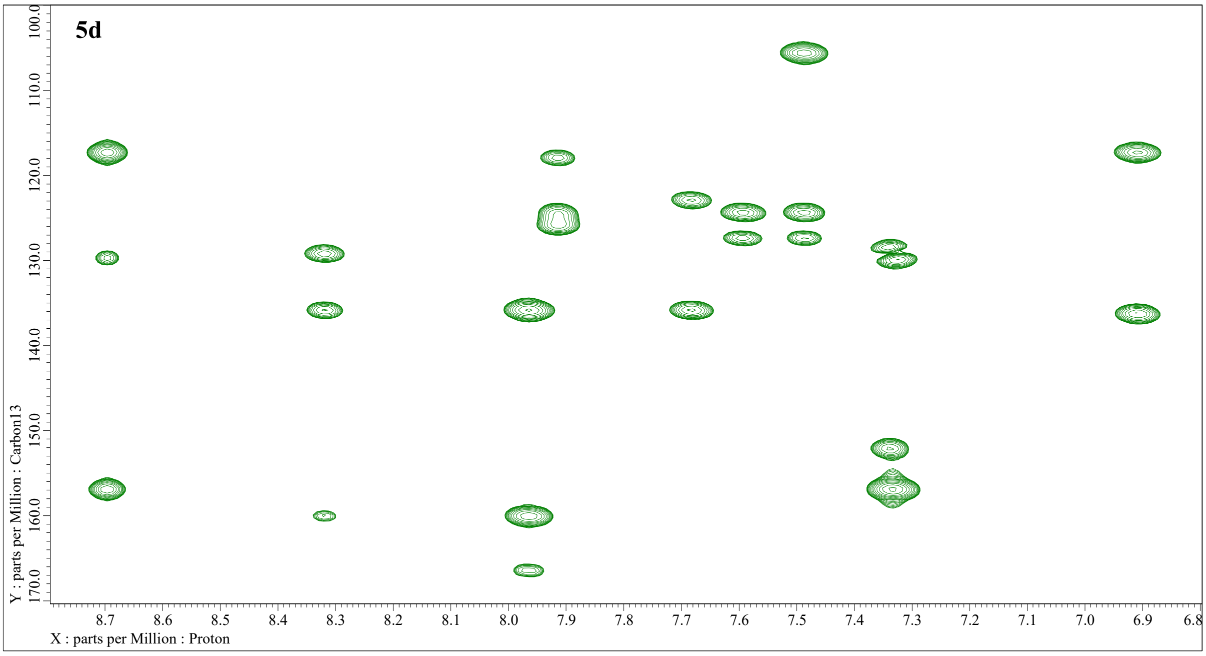

Figure S289. Expansion of 2D-NMR (400 MHz, DMSO-d_6) HMBC experiment of *N*'-[(*E*)-(3-*tert*-butyl-2-hydroxy-phenyl)methylidene]-2-(1-hydroksynaphtho)hydrazide (**5d**)

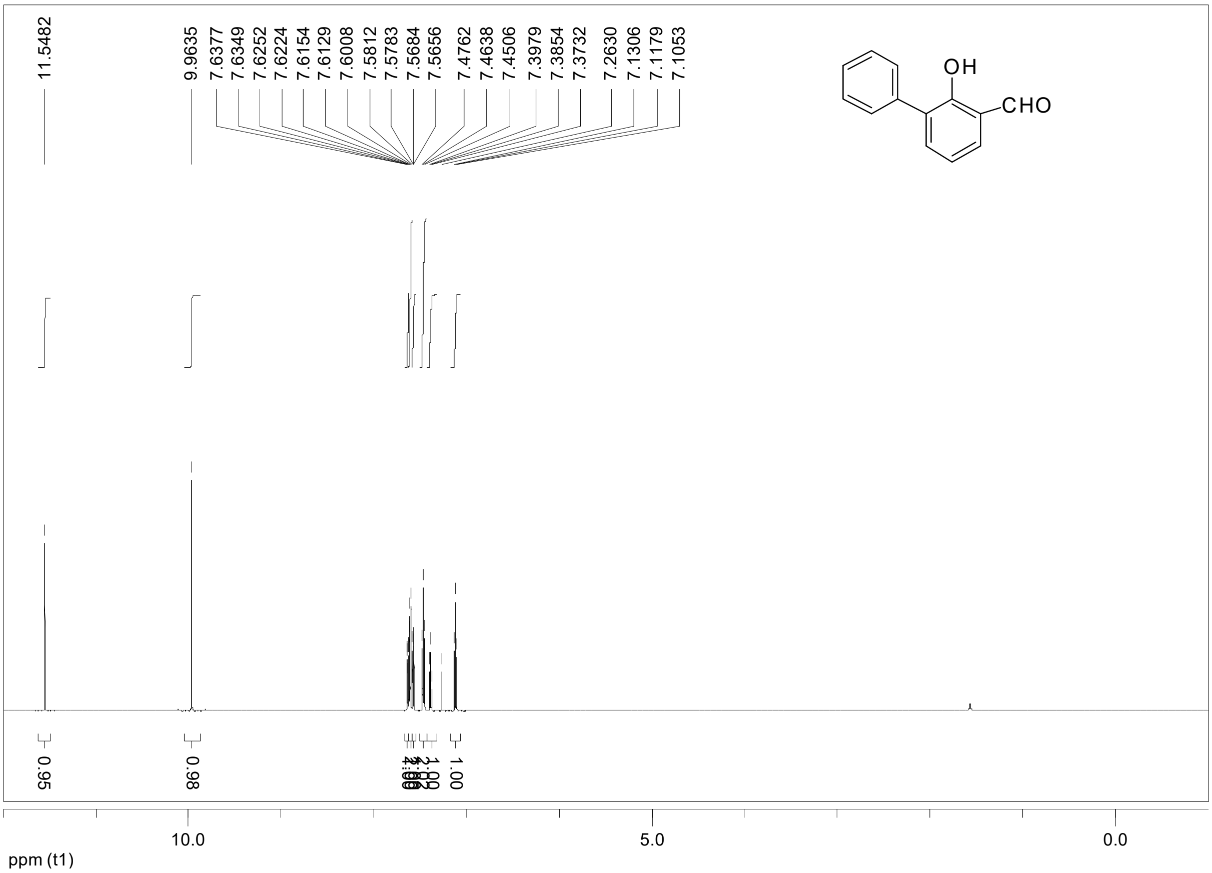

Figure S290. ^{1}H NMR (600 MHz, $CDCl_3$) spectrum of 2-hydroxy-3-phenylbenzaldehyde (**6**)

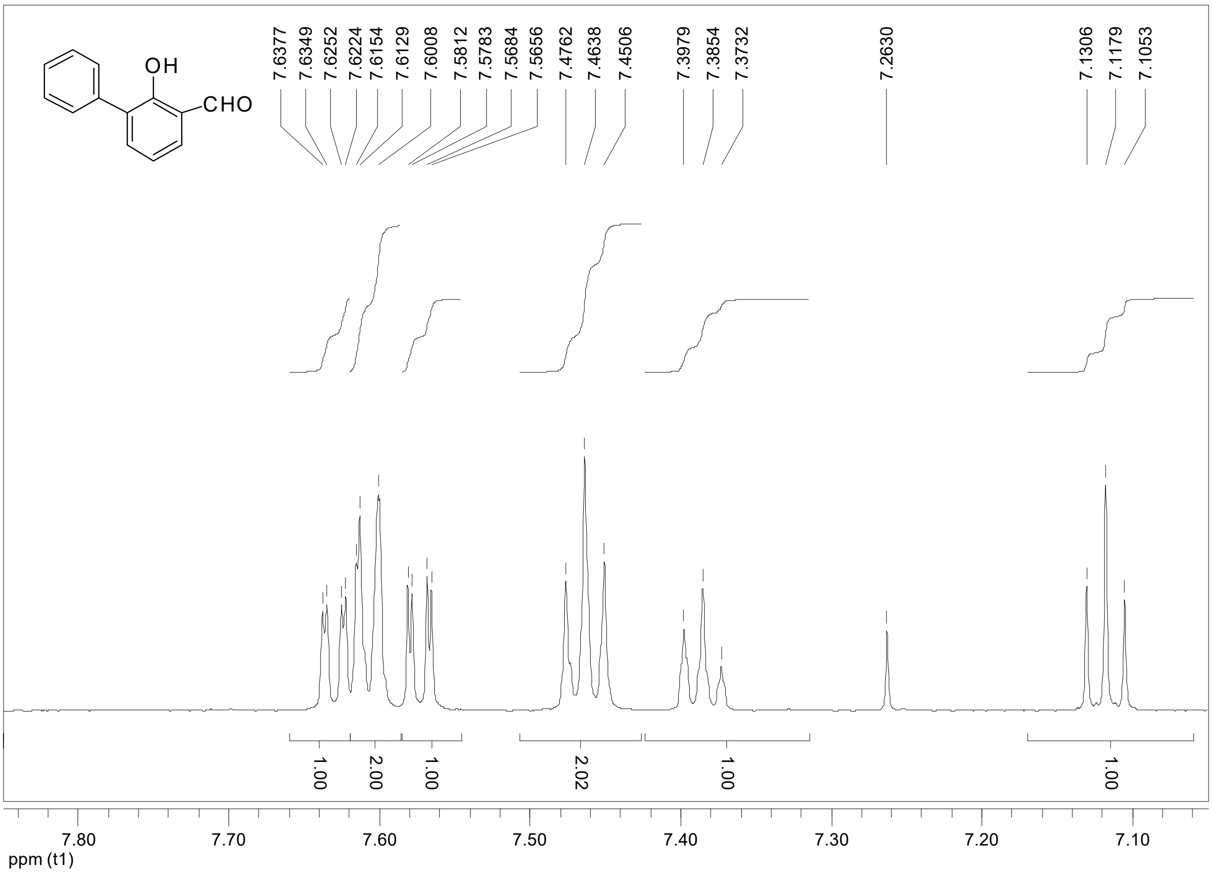

Figure S291. Expansion of ^{1}H NMR (600 MHz, $CDCl_3$) spectrum of 2-hydroxy-3-phenylbenzaldehyde (**6**)

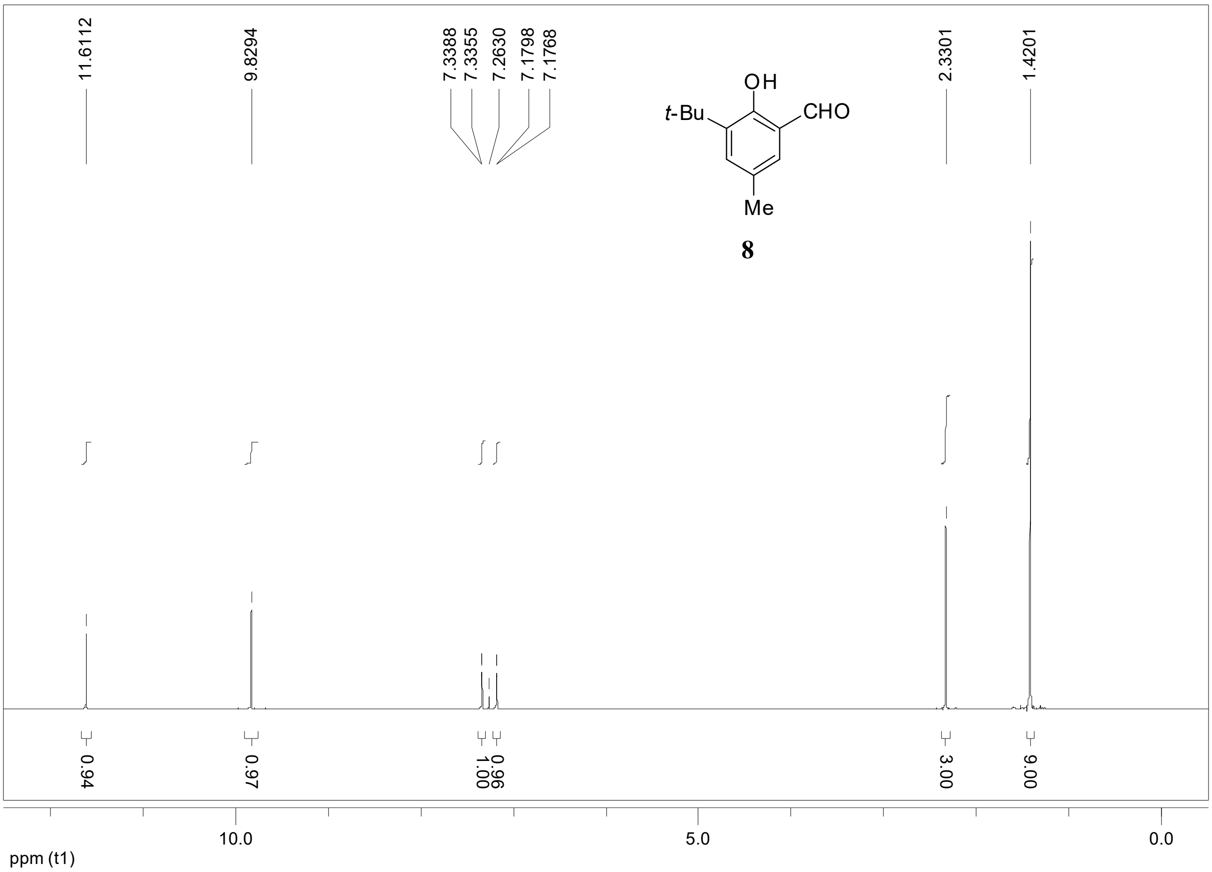

Figure S292. ¹H-NMR (600 MHz, $CDCl_3$) spectrum of 5-methyl-salicylic aldehyde (**8**)

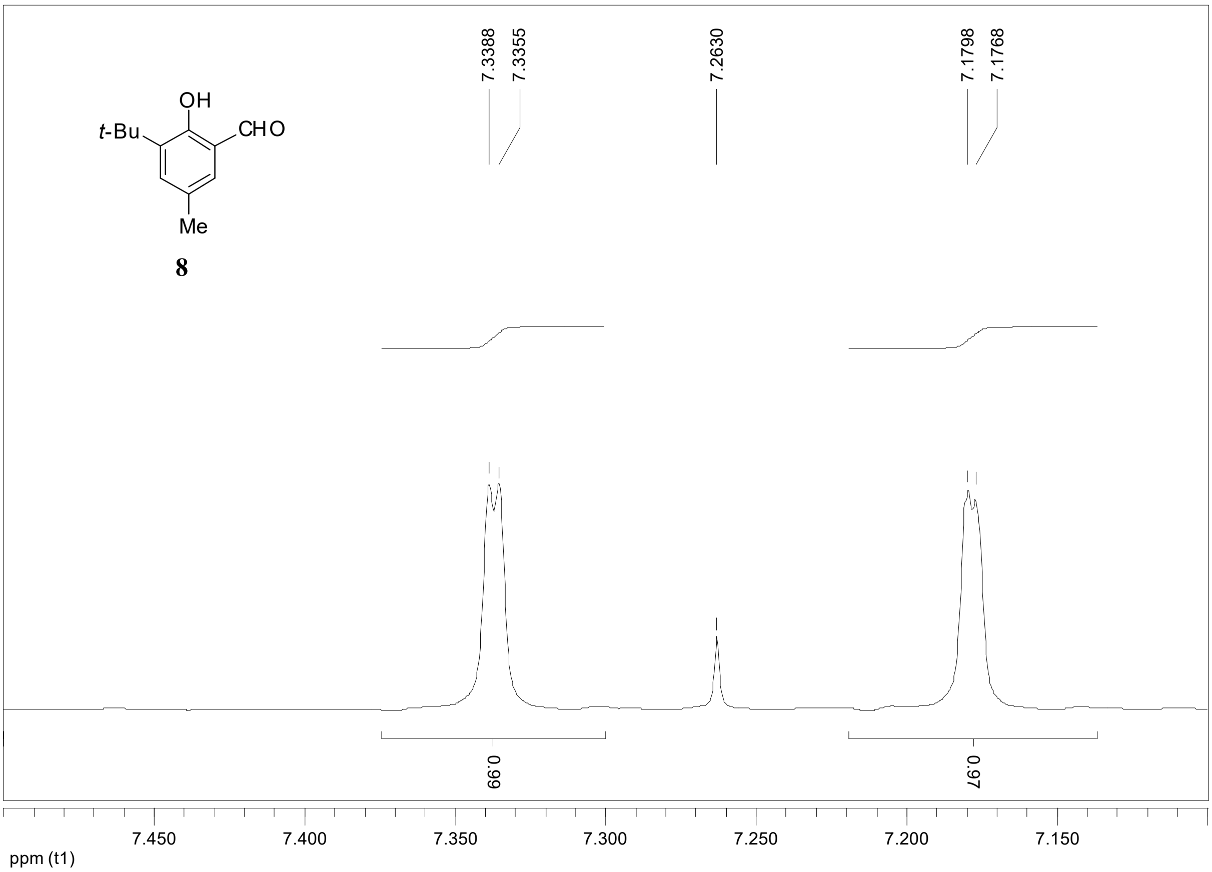

Figure S293. Expansion of ¹H-NMR (600 MHz, $CDCl_3$) spectrum of 3-*tert*-butyl-5-methyl-salicylic aldehyde (**8**)

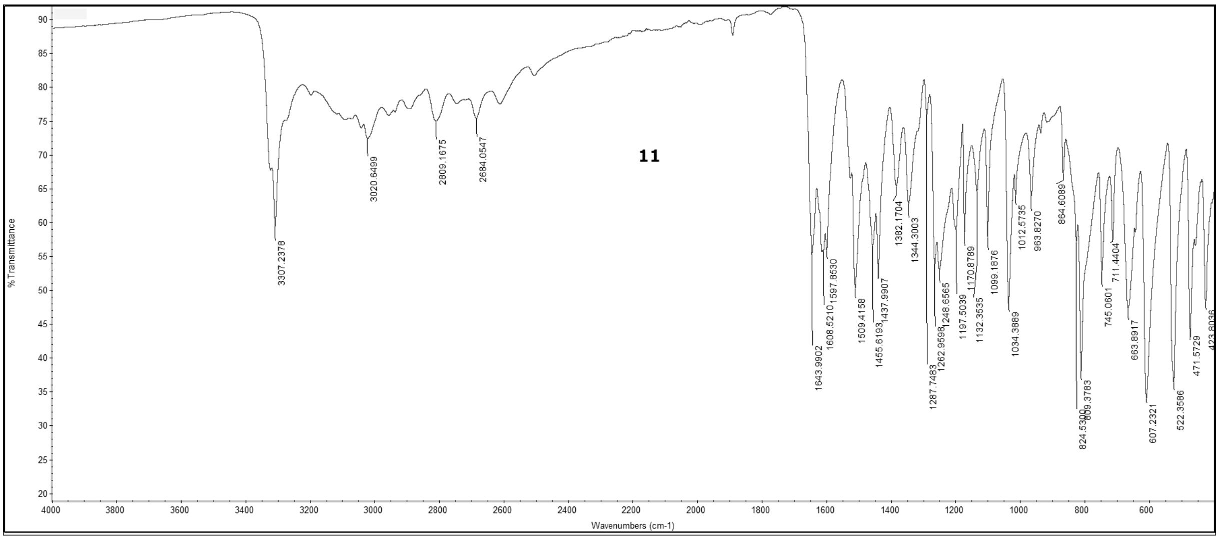

Figure S294. FT-IR (ATR) spectrum of 2-(4-hydroxyphenyl)acetic acid hydrazide (**11**)

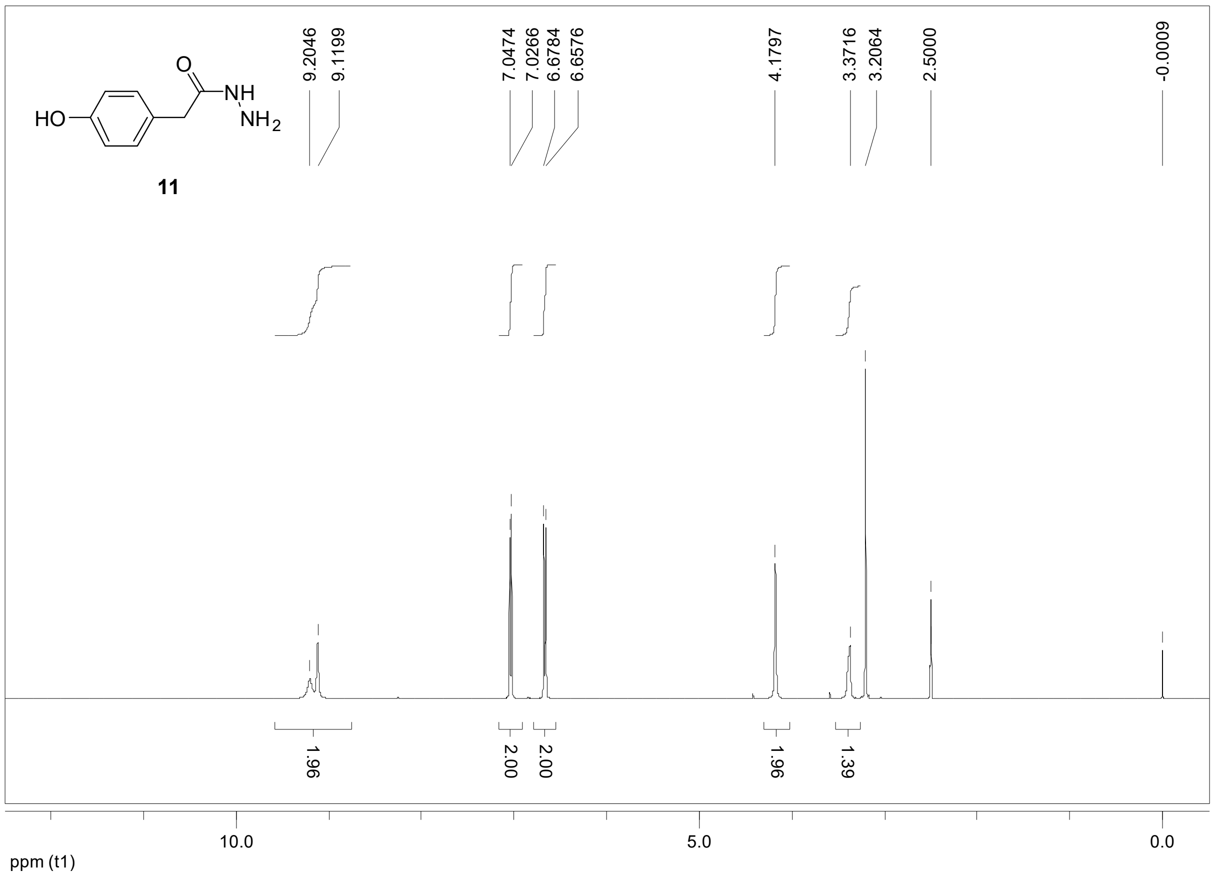

Figure S295. ^{1}H-NMR (400 MHz, DMSO-d_6) spectrum of 2-(4-hydroxyphenyl)acetic acid hydrazide (**11**)

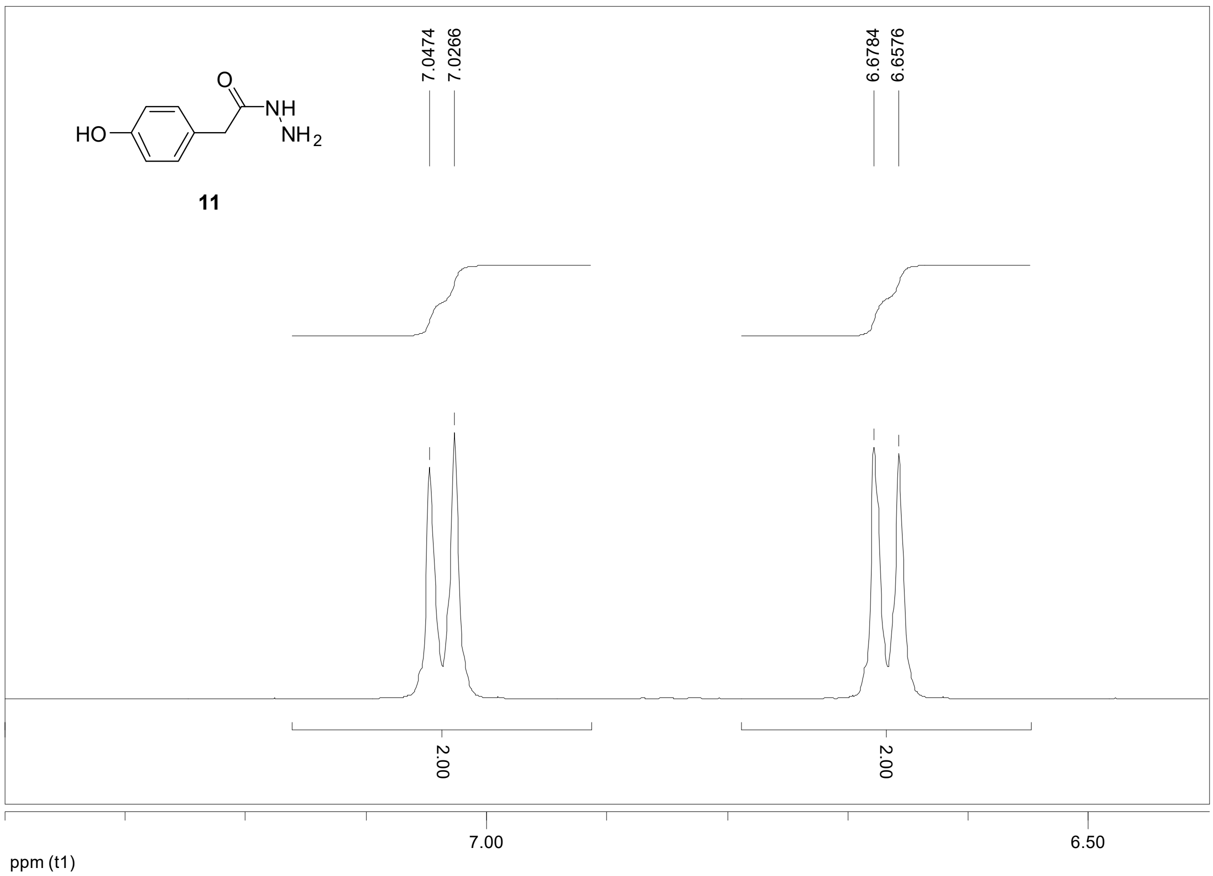

Figure S296. Expansion of ^{1}H-NMR (400 MHz, DMSO-d_6) spectrum of hydroxyphenylacetic acid hydrazide **11**

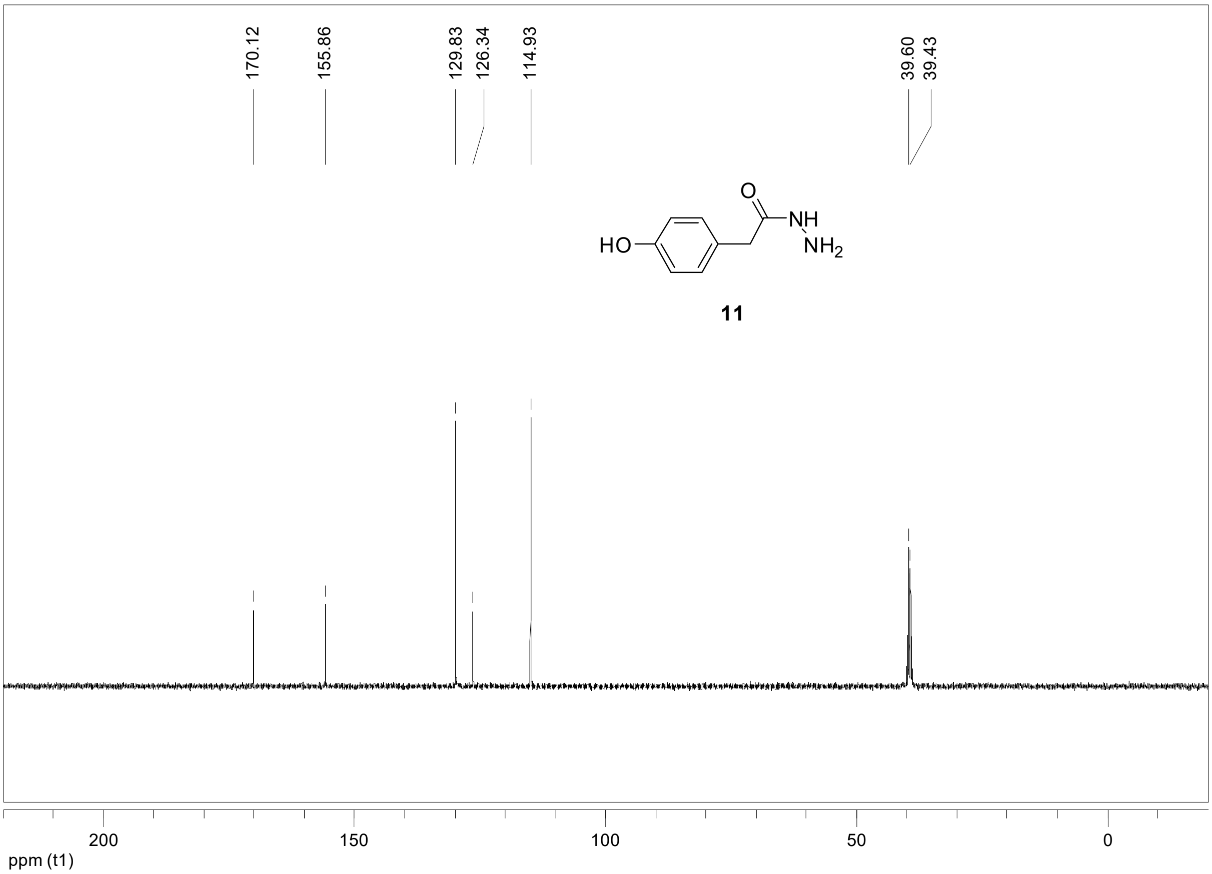

Figure S297. ^{13}C-NMR (100 MHz, DMSO-d_6) spectrum of 2-(4-hydroxyphenyl)acetic acid hydrazide (**11**)

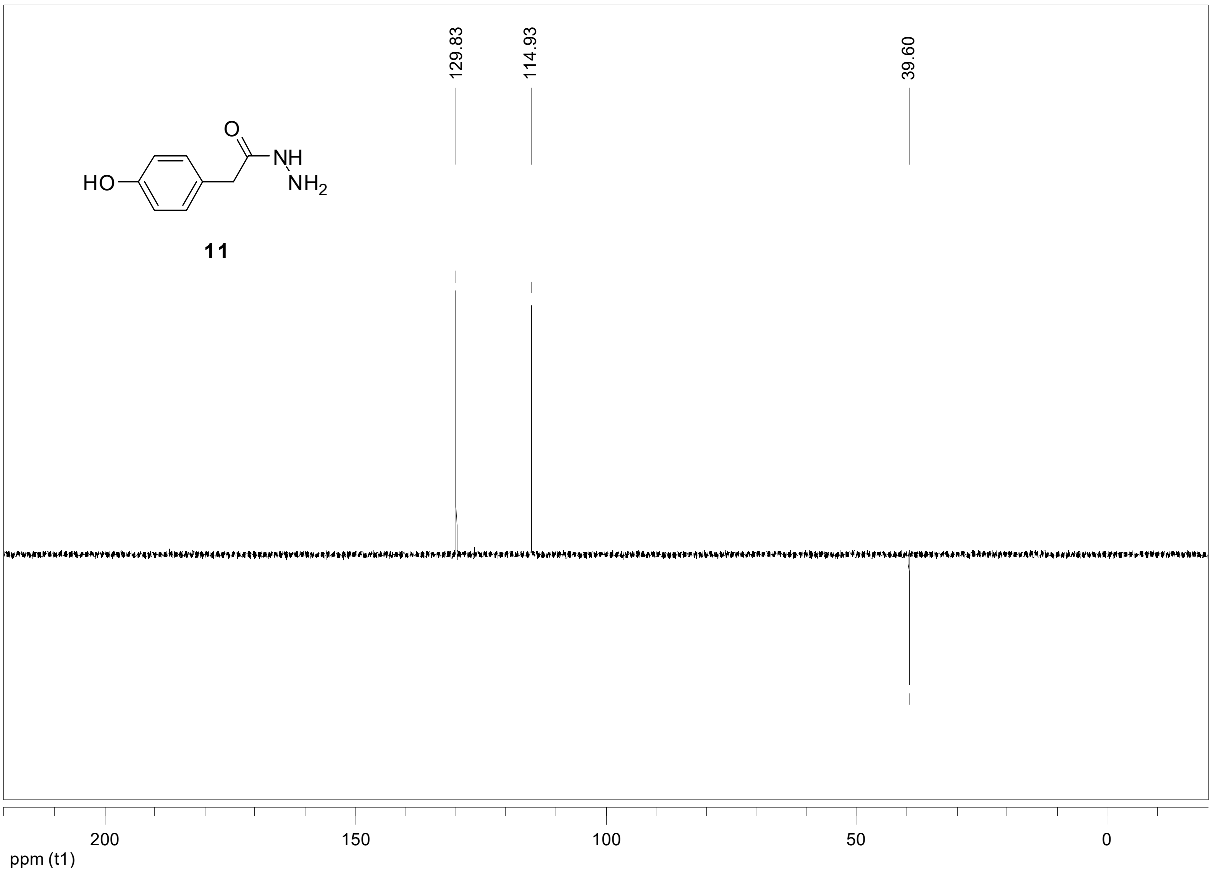

Figure S298. ^{13}C-NMR (100 MHz, DMSO-d_6) dept-135 experiment of 4-hydroxyphenylacetic acid hydrazide **11**

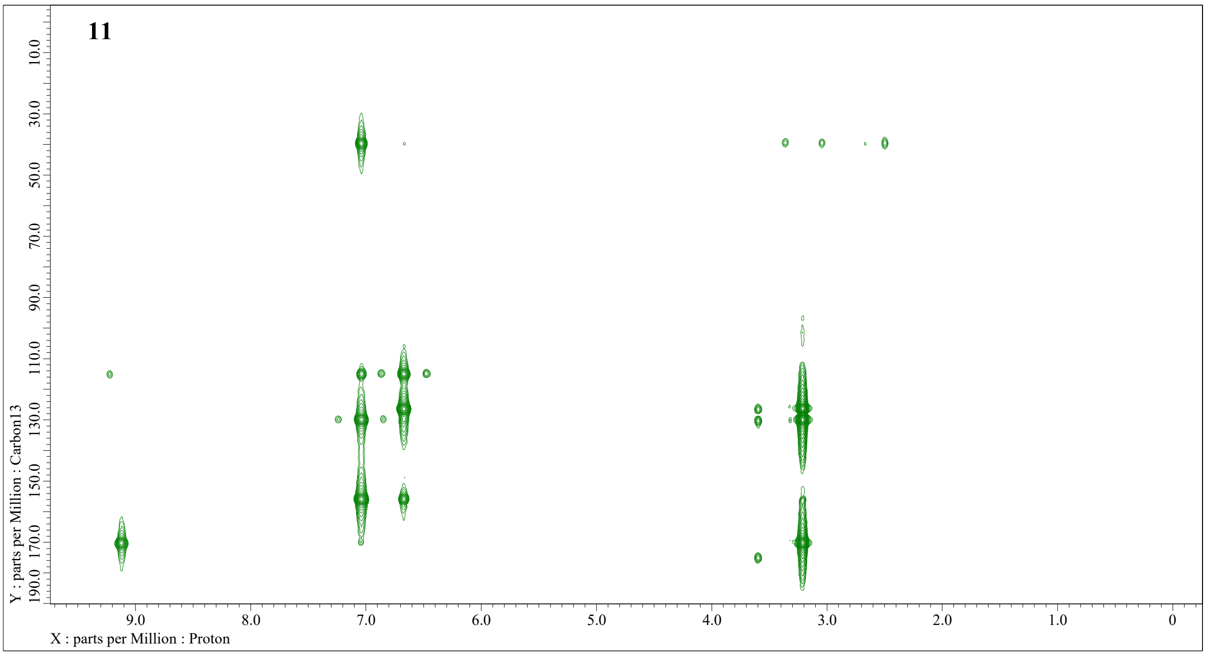

Figure S299. 2D-NMR (400 MHz, DMSO-d_6) HMBC experiment of 2-(4-hydroxyphenyl)acetic acid hydrazide (**11**)

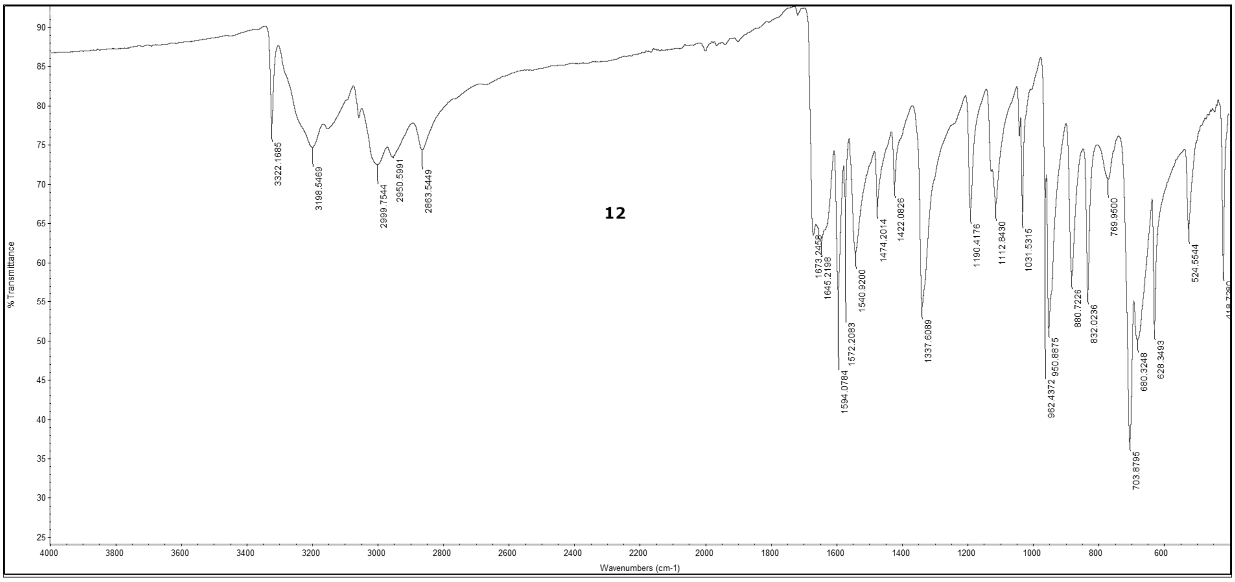

Figure S300. FT-IR (ATR) spectrum of nicotinic acid hydrazide (**12**)

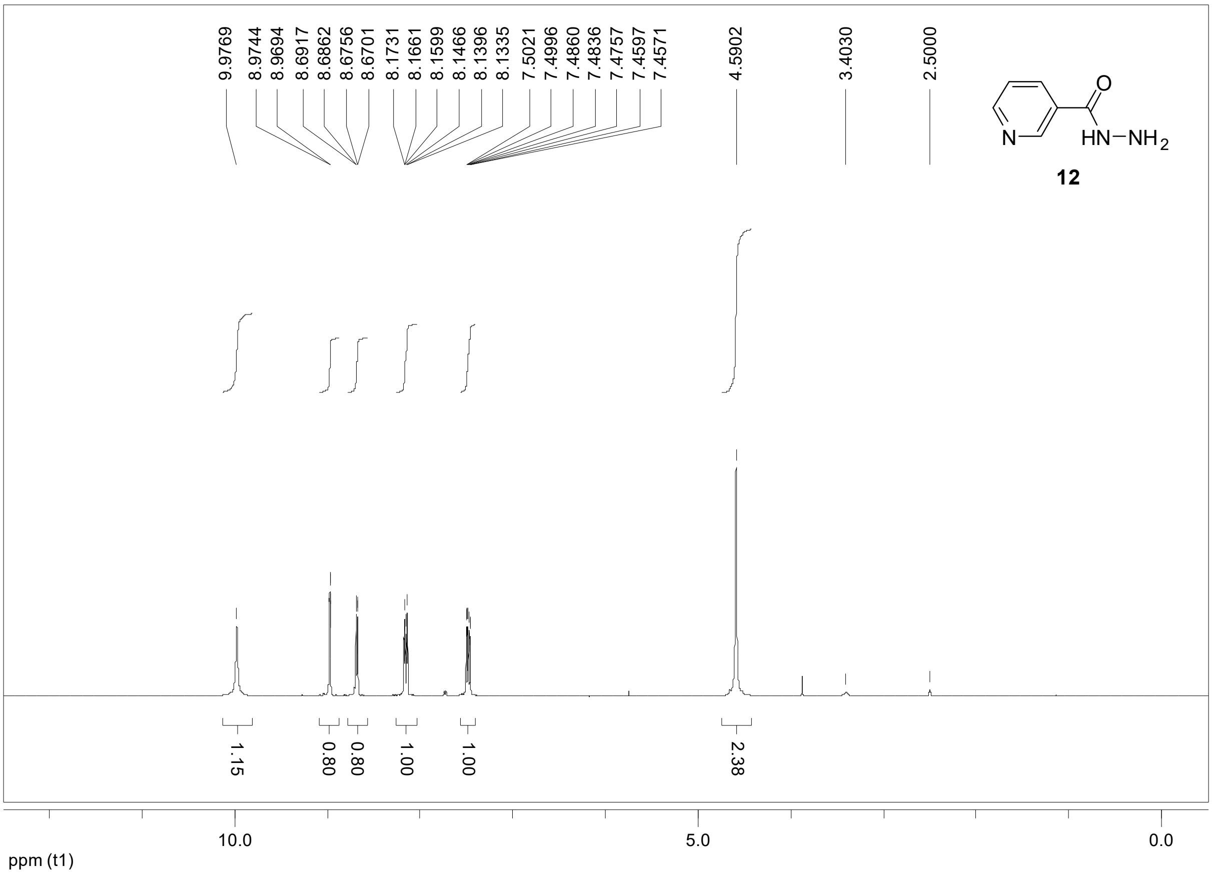

Figure S301. ^{1}H-NMR (300 MHz, DMSO-d_6) spectrum of nicotinic acid hydrazide (**12**)

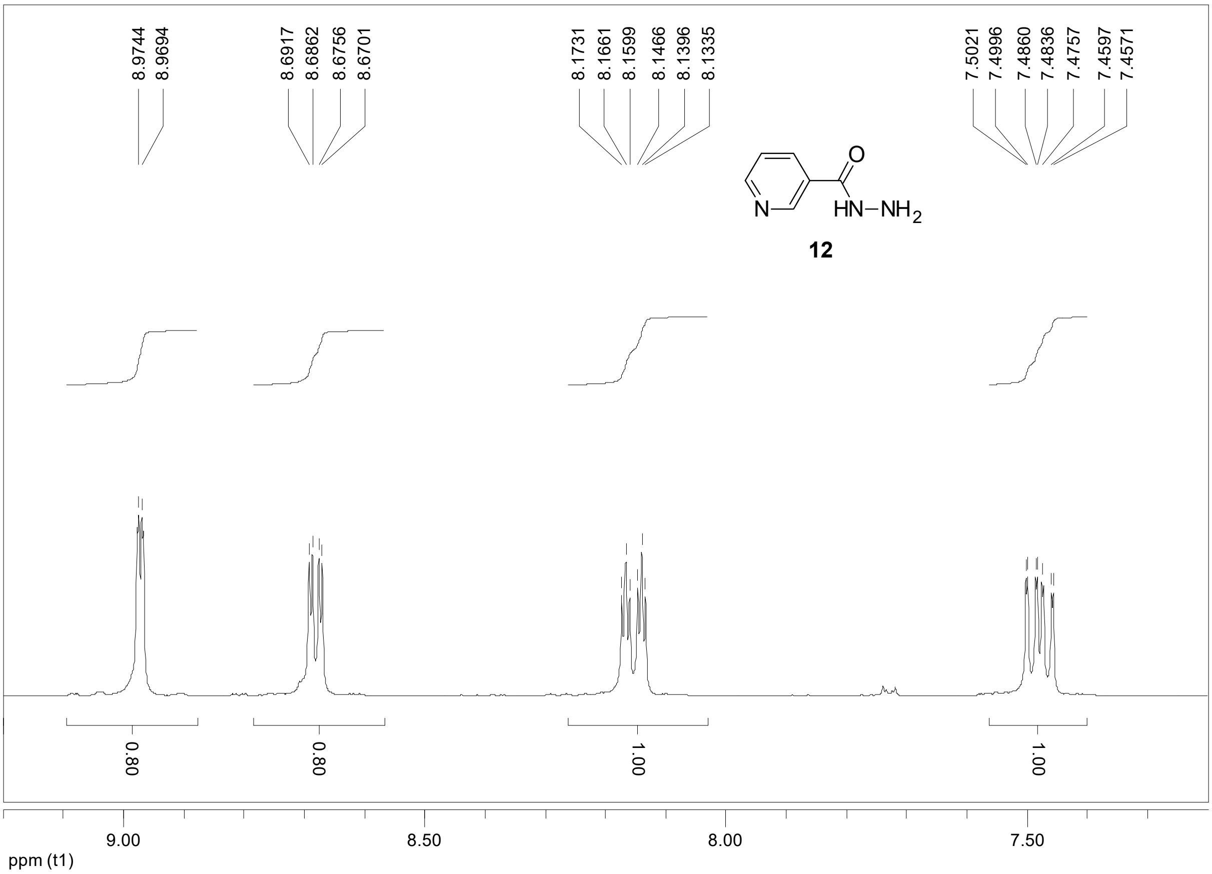

Figure S302. Expansion of ^{1}H-NMR (300 MHz, DMSO-d_6) spectrum of nicotinic acid hydrazide (**12**)

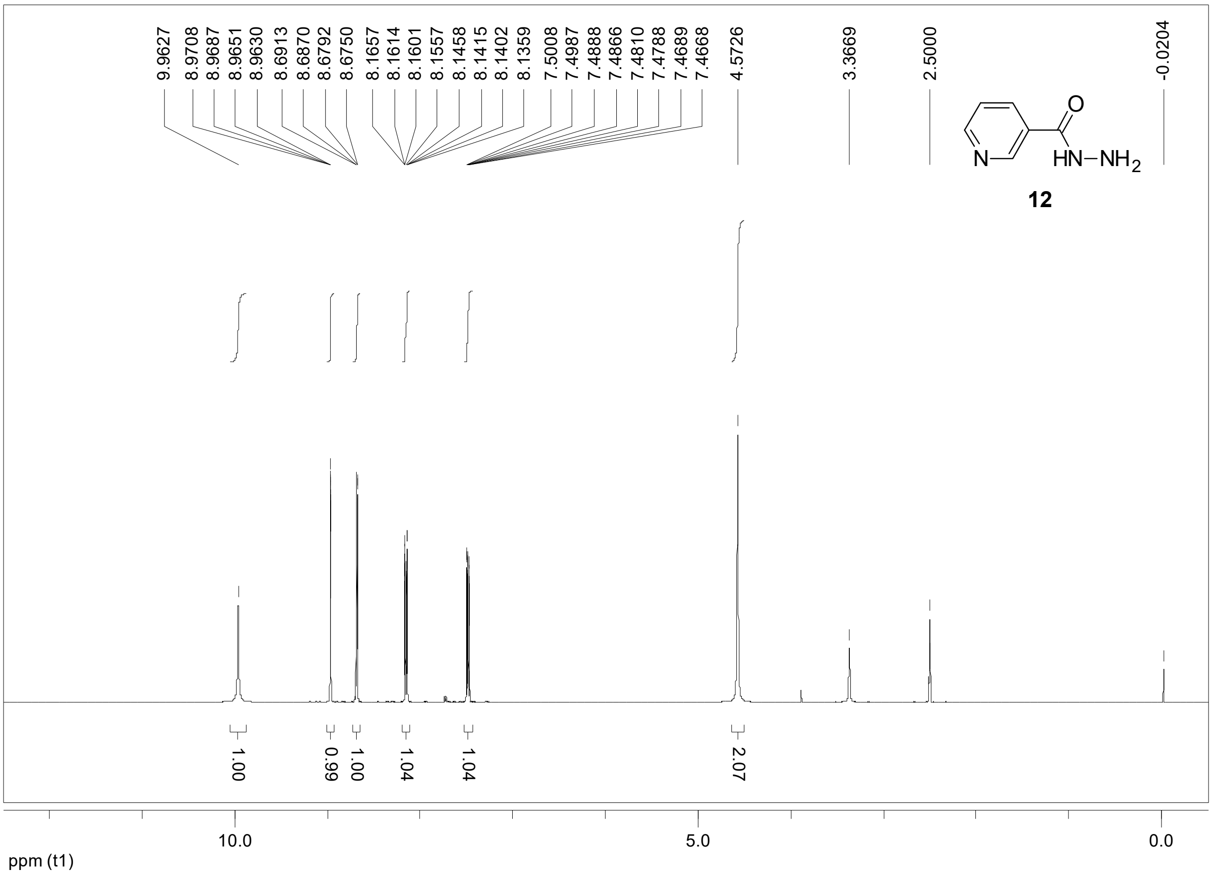

Figure S303. ^{1}H-NMR (400 MHz, DMSO-d_6) spectrum of nicotinic acid hydrazide (**12**)

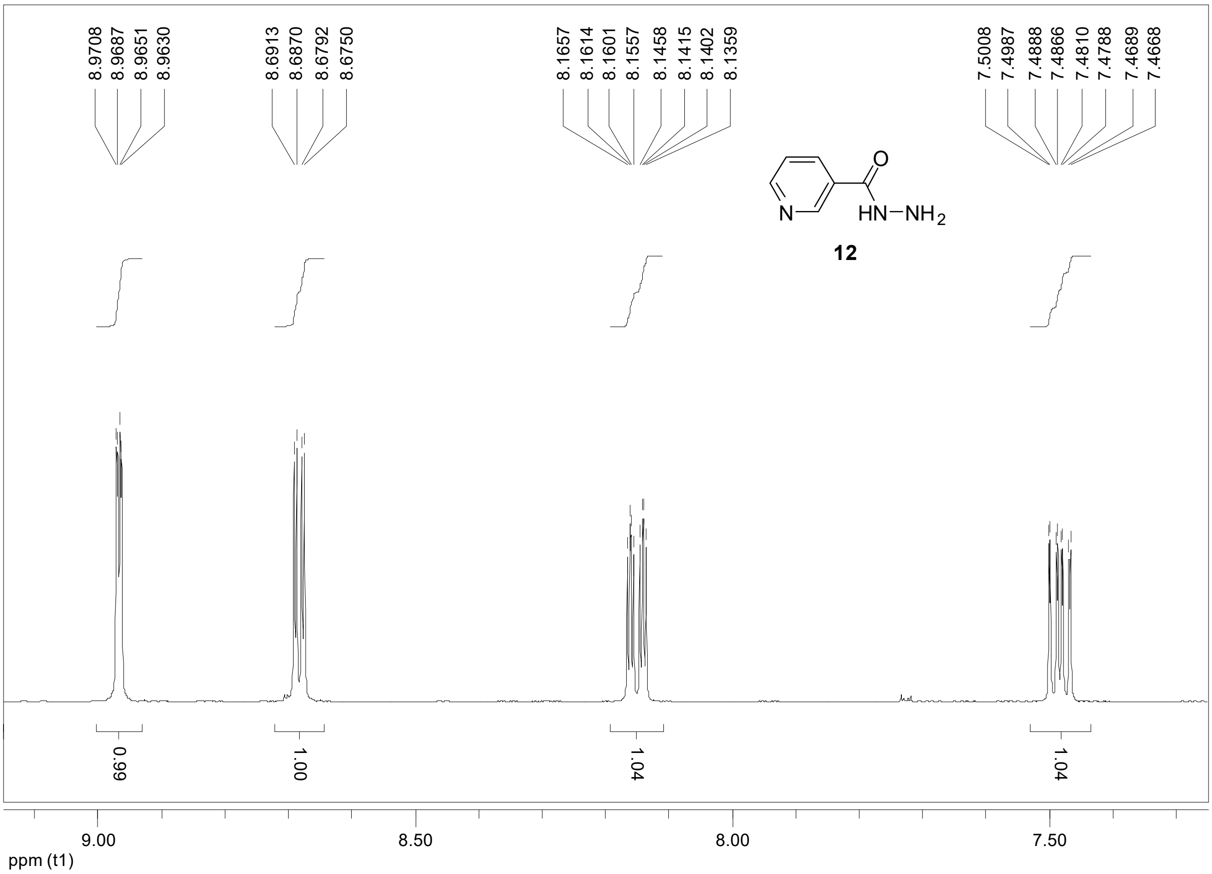

Figure S304. Expansion of ^{1}H-NMR (400 MHz, DMSO-d_6) spectrum of nicotinic acid hydrazide (**12**)

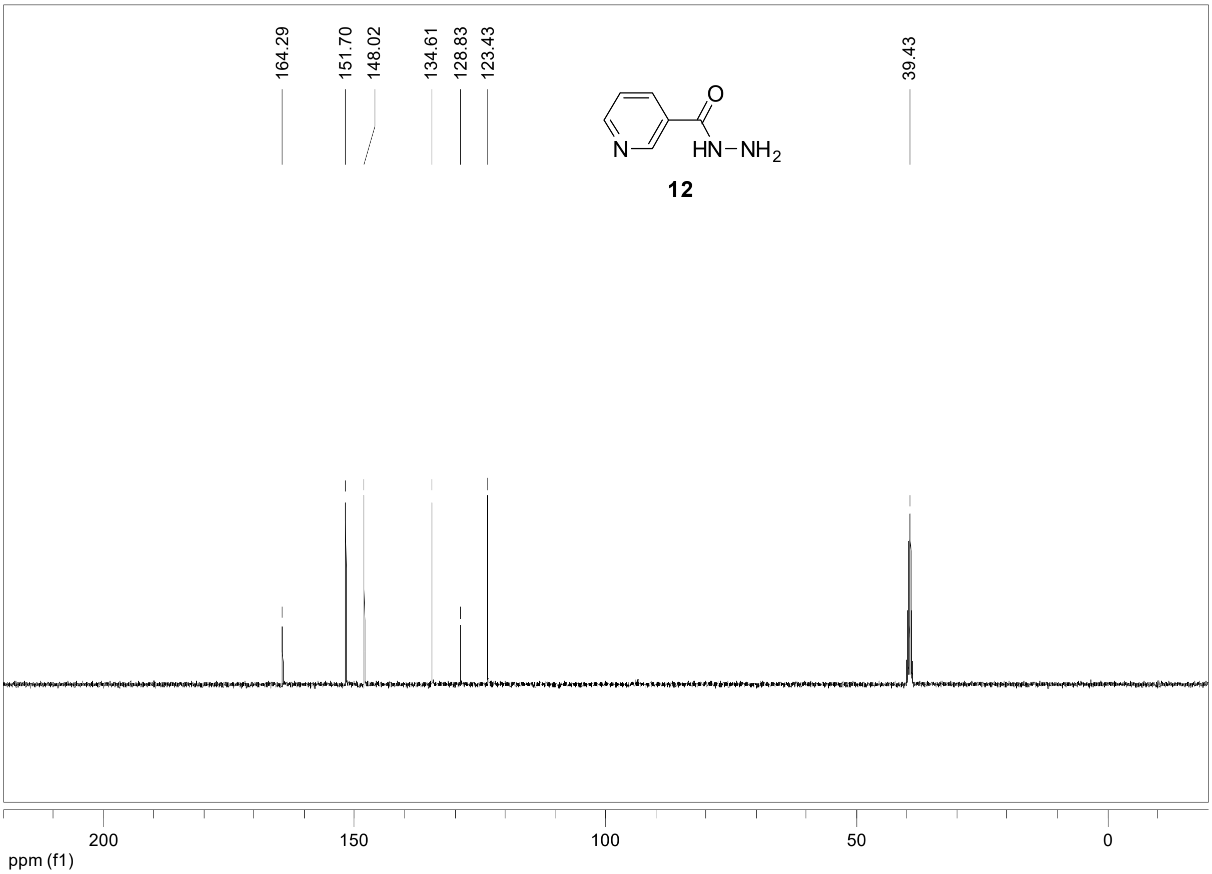

Figure S305. ^{13}C-NMR (100 MHz, DMSO-d_6) spectrum of nicotinic acid hydrazide (**12**)

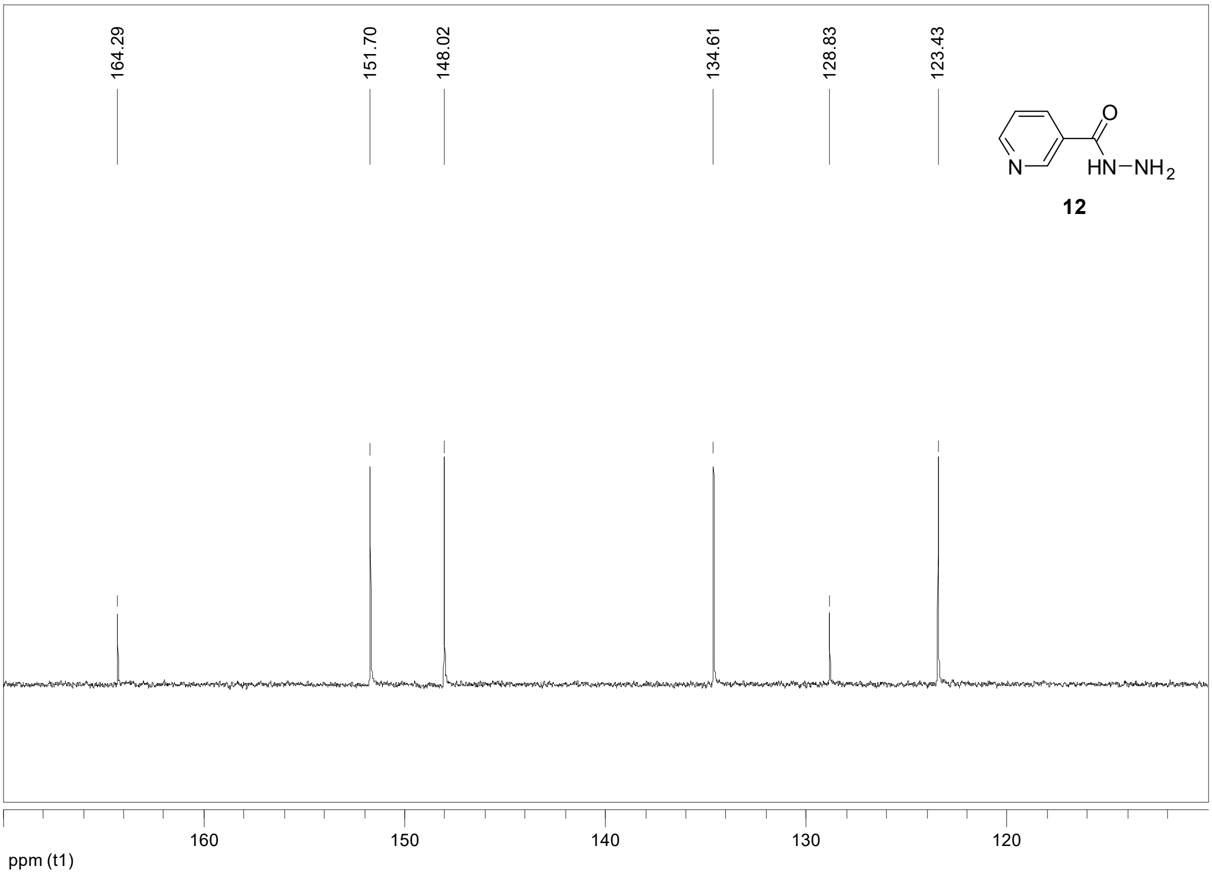

Figure S306. Expansion of ^{13}C-NMR (100 MHz, DMSO-d_6) spectrum of nicotinic acid hydrazide (**12**)

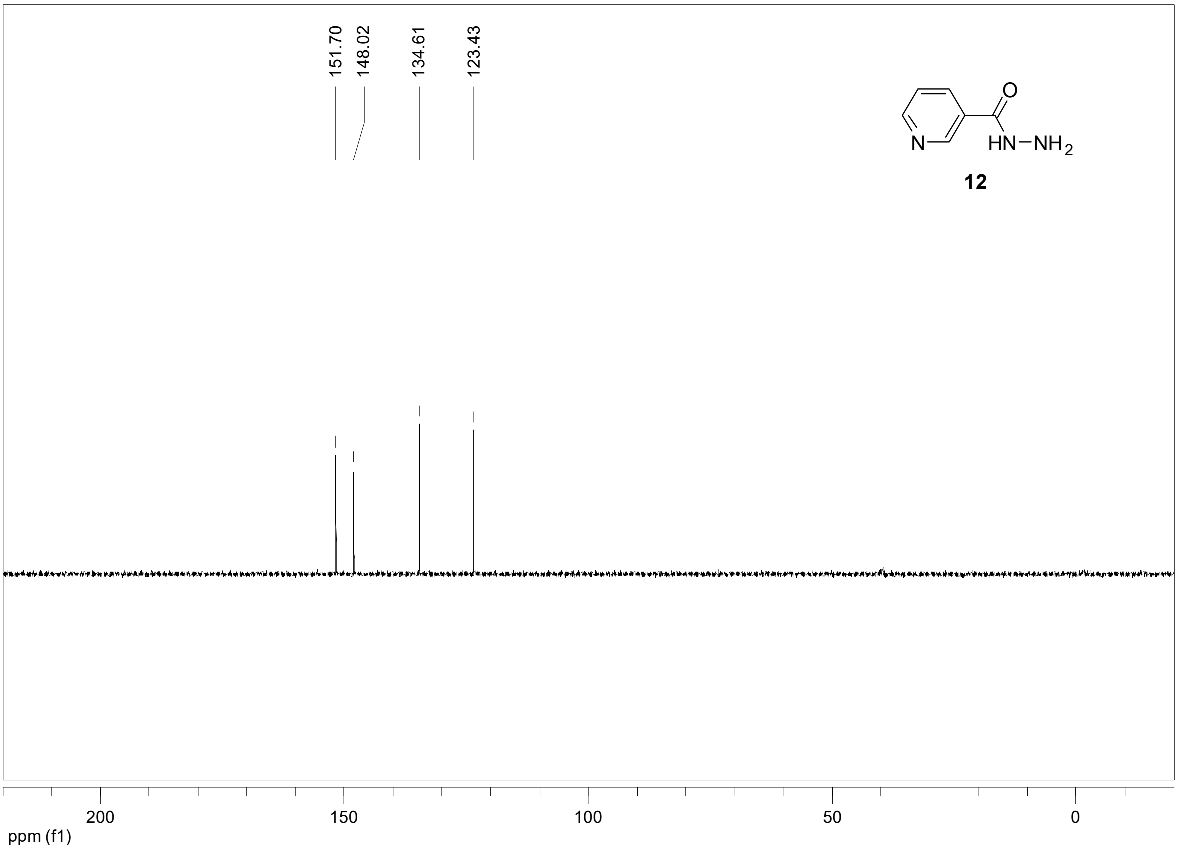

Figure S307. ^{13}C-NMR (100 MHz, DMSO-d_6) dept-135 experiment of nicotinic acid hydrazide (**12**)

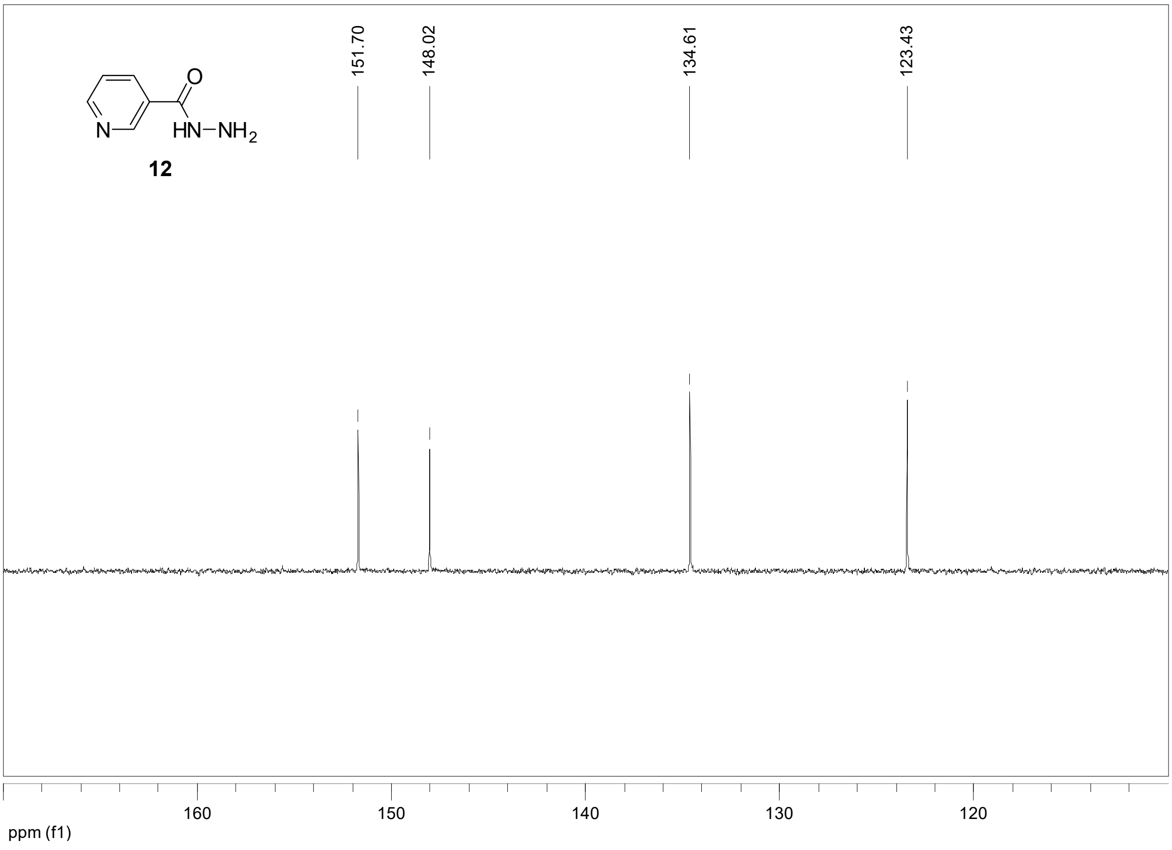

Figure S308. Expansion of ^{13}C-NMR (100 MHz, DMSO-d_6) dept-135 experiment of nicotinic acid hydrazide (**12**)

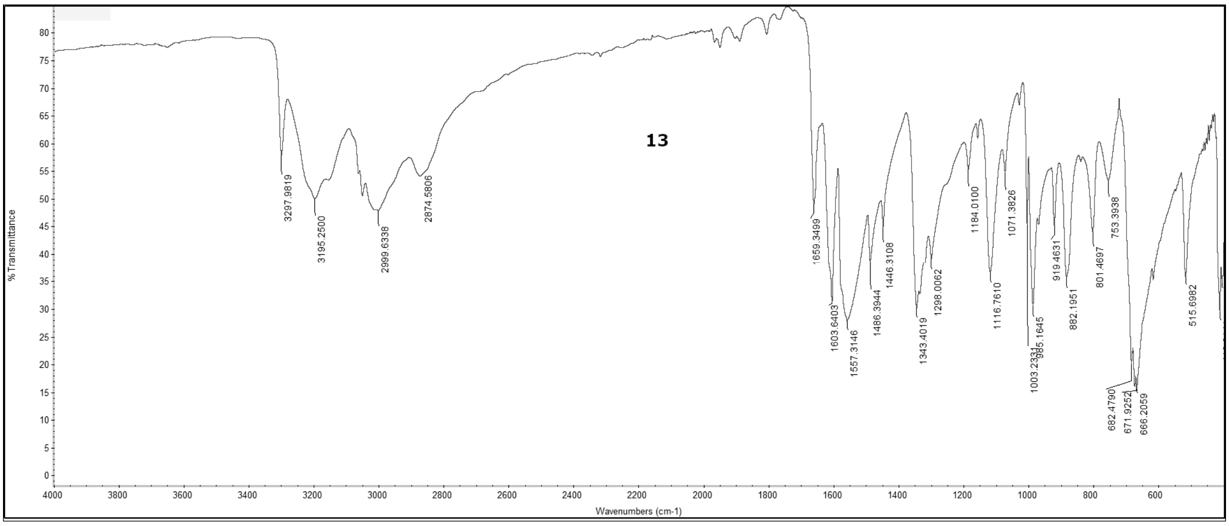

Figure S309. FT-IR (ATR) spectrum of benzoic acid hydrazide (**13**)

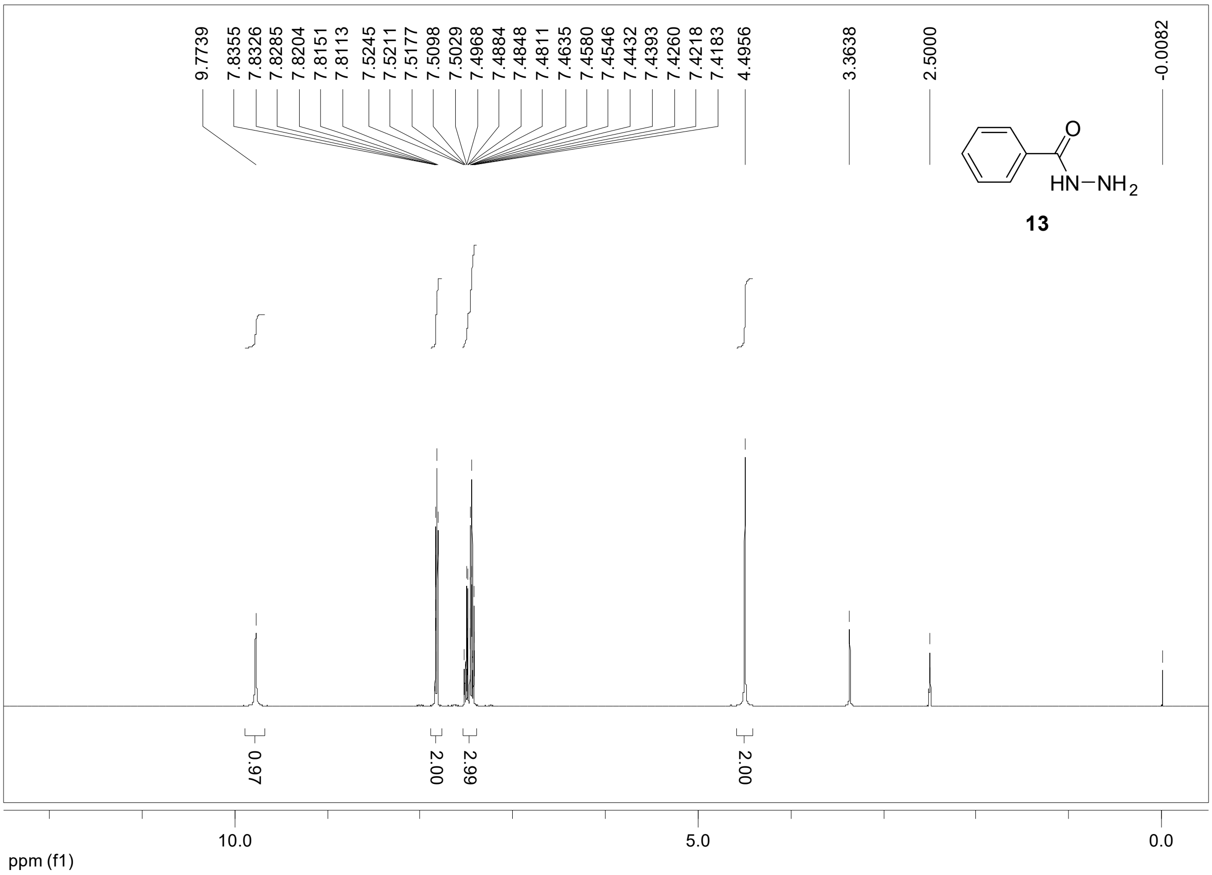

Figure S310. ^{1}H-NMR (400 MHz, DMSO-d_6) spectrum of benzoic acid hydrazide (**13**)

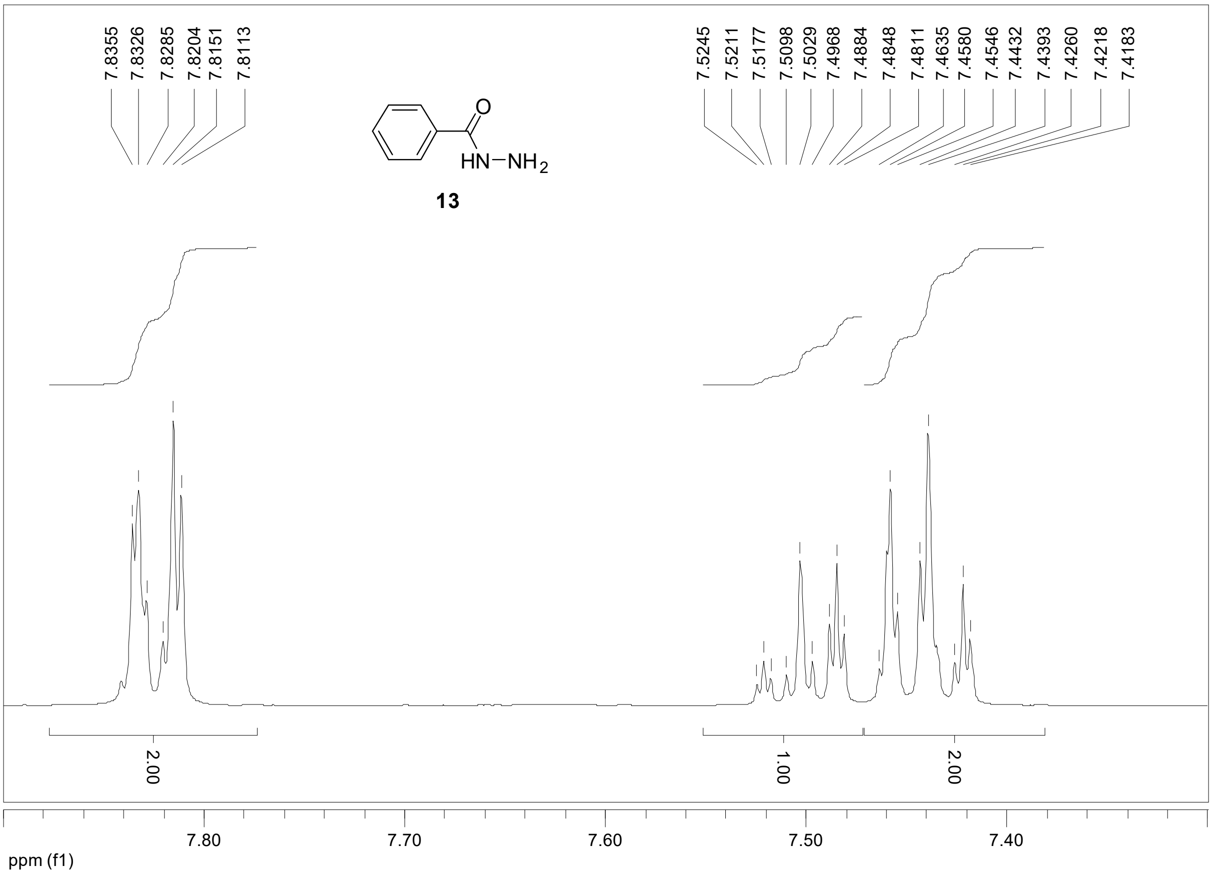

Figure S311. Expansion of ^{1}H-NMR (400 MHz, DMSO-d_6) spectrum of benzoic acid hydrazide (**13**)

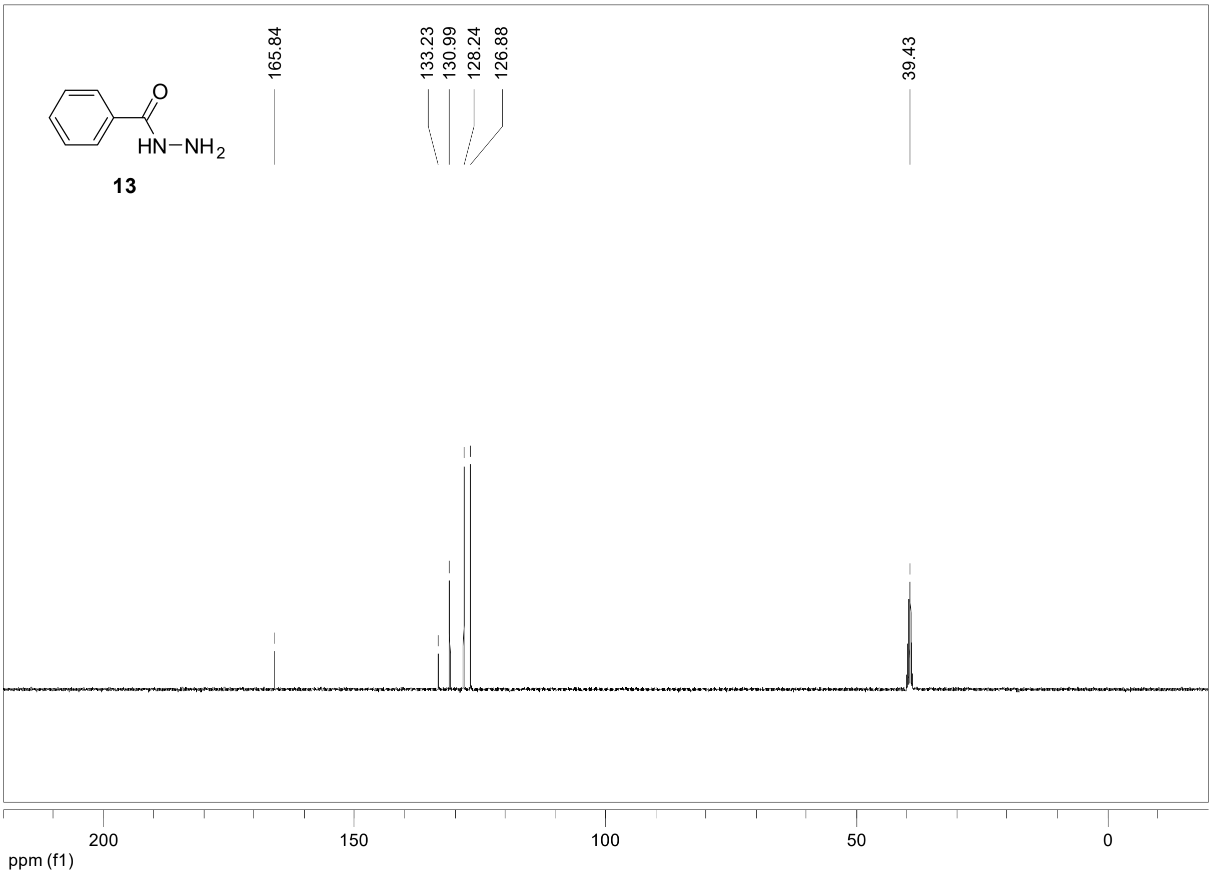

Figure S312. ^{13}C-NMR (100 MHz, DMSO-d_6) spectrum of benzoic acid hydrazide (**13**)

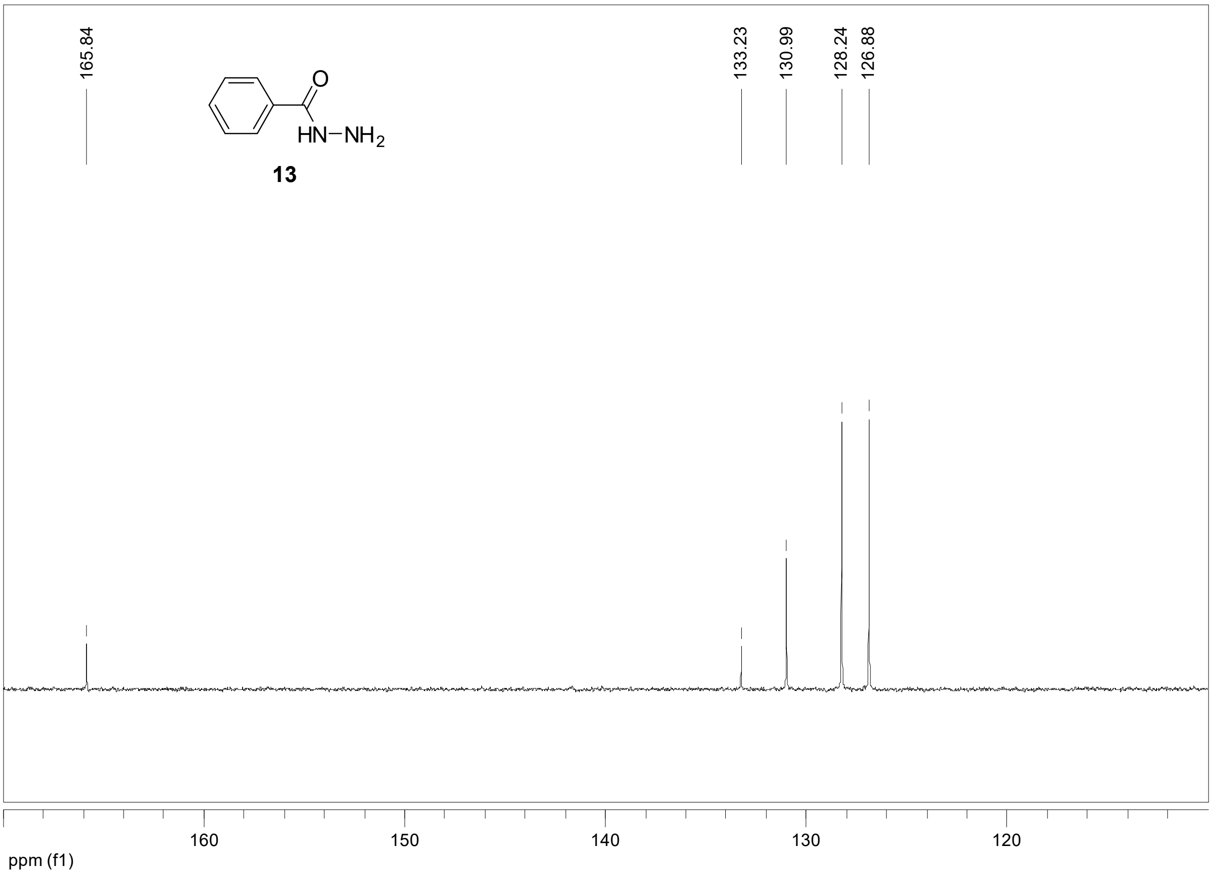

Figure S313. Expansion of ^{13}C-NMR (100 MHz, DMSO-d_6) spectrum of benzoic acid hydrazide (**13**)

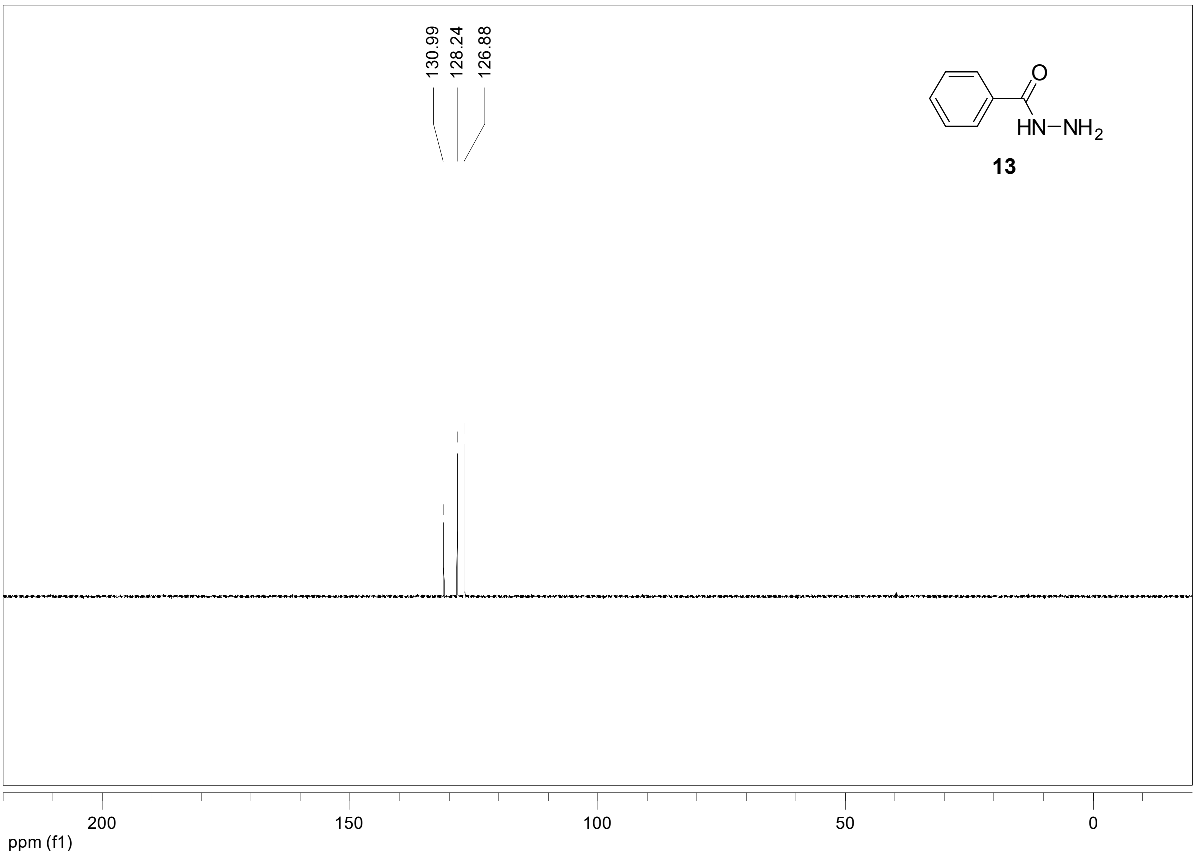

Figure S314. ^{13}C-NMR (100 MHz, DMSO-d_6) dept-135experiment of benzoic acid hydrazide (**13**)

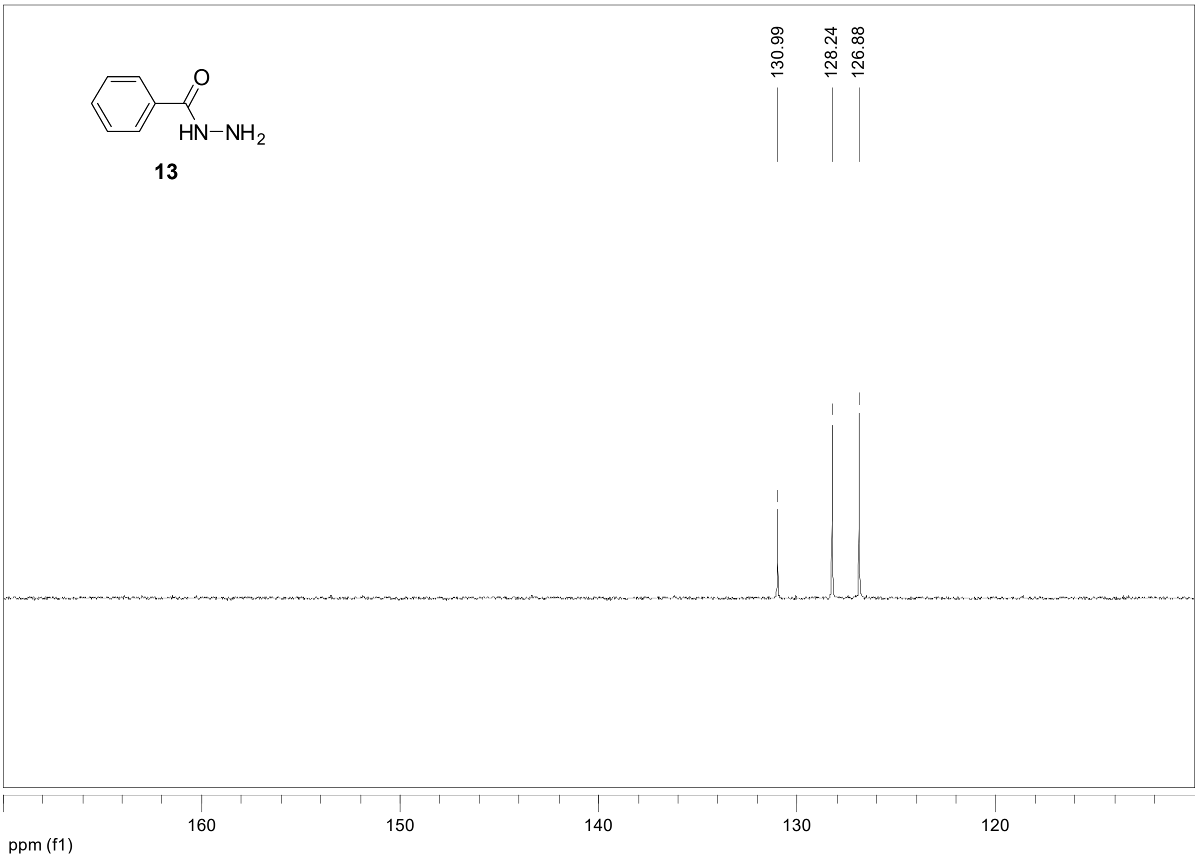

Figure S315. Expansion of ^{13}C-NMR (100 MHz, DMSO-d_6) dept-135 experiment of hydrazide (**13**)

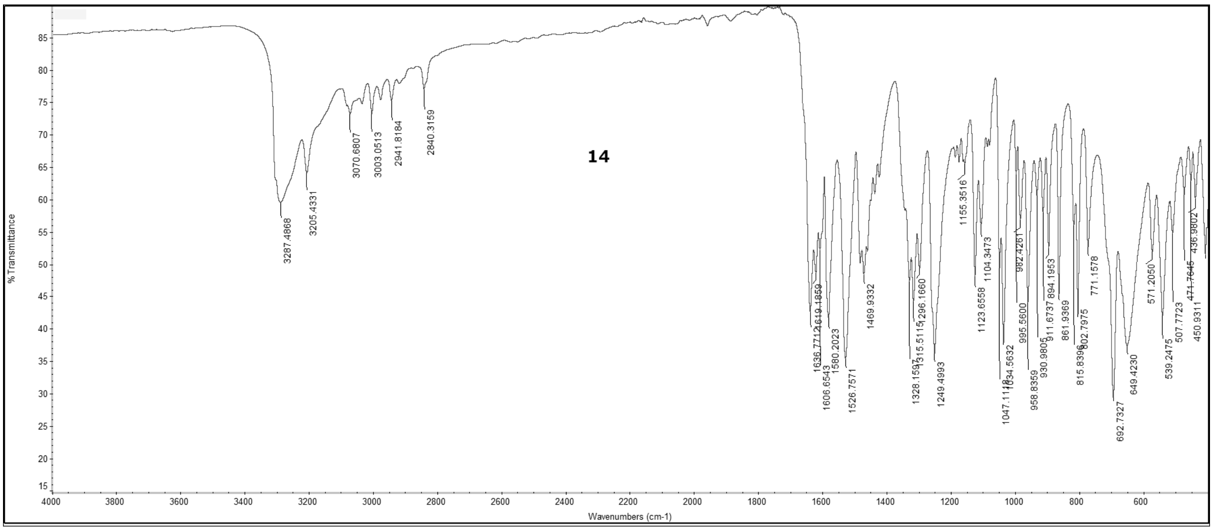

Figure S316. FT-IR (ATR) spectrum of 3-methoxybenzoic acid hydrazide (**14**)

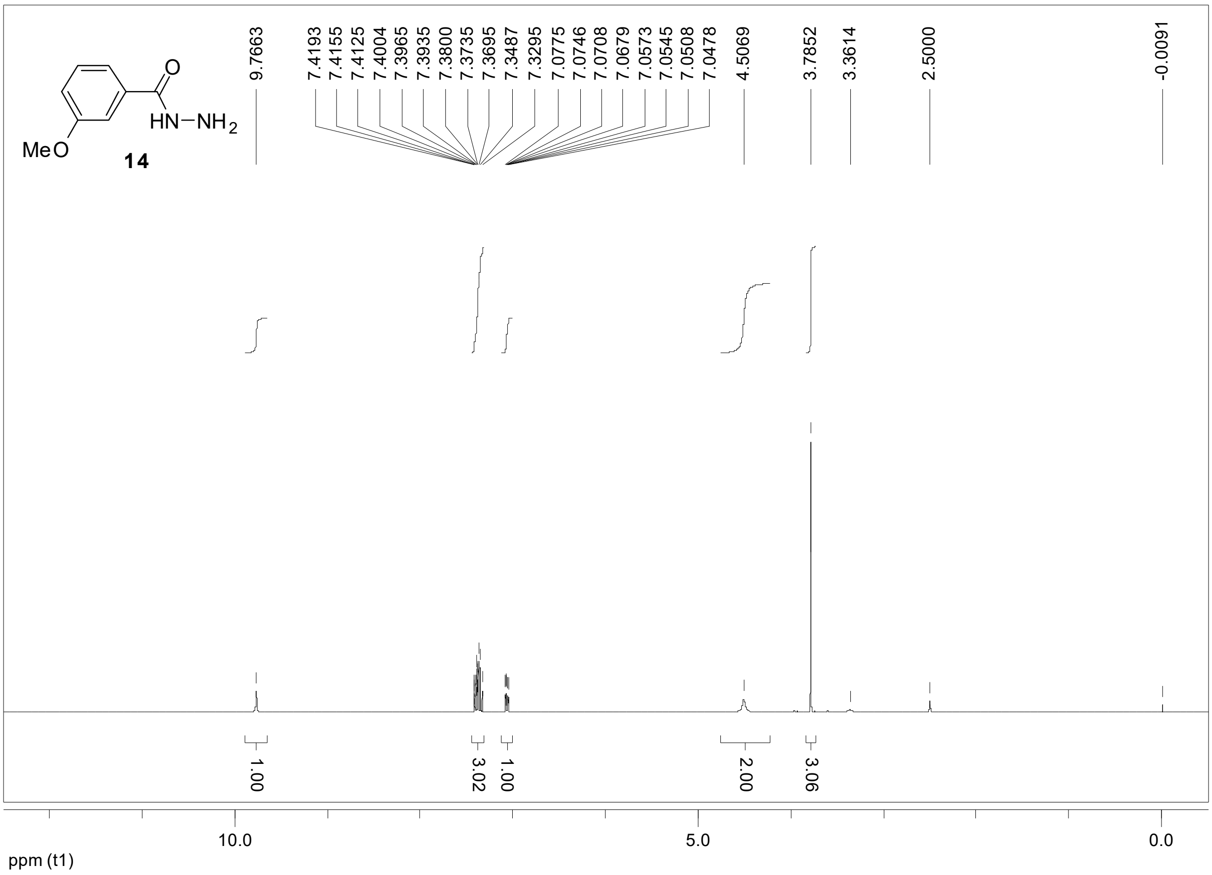

Figure S317. ^{1}H-NMR (400 MHz, DMSO-d_6) spectrum of 3-methoxybenzoic acid hydrazide (**14**)

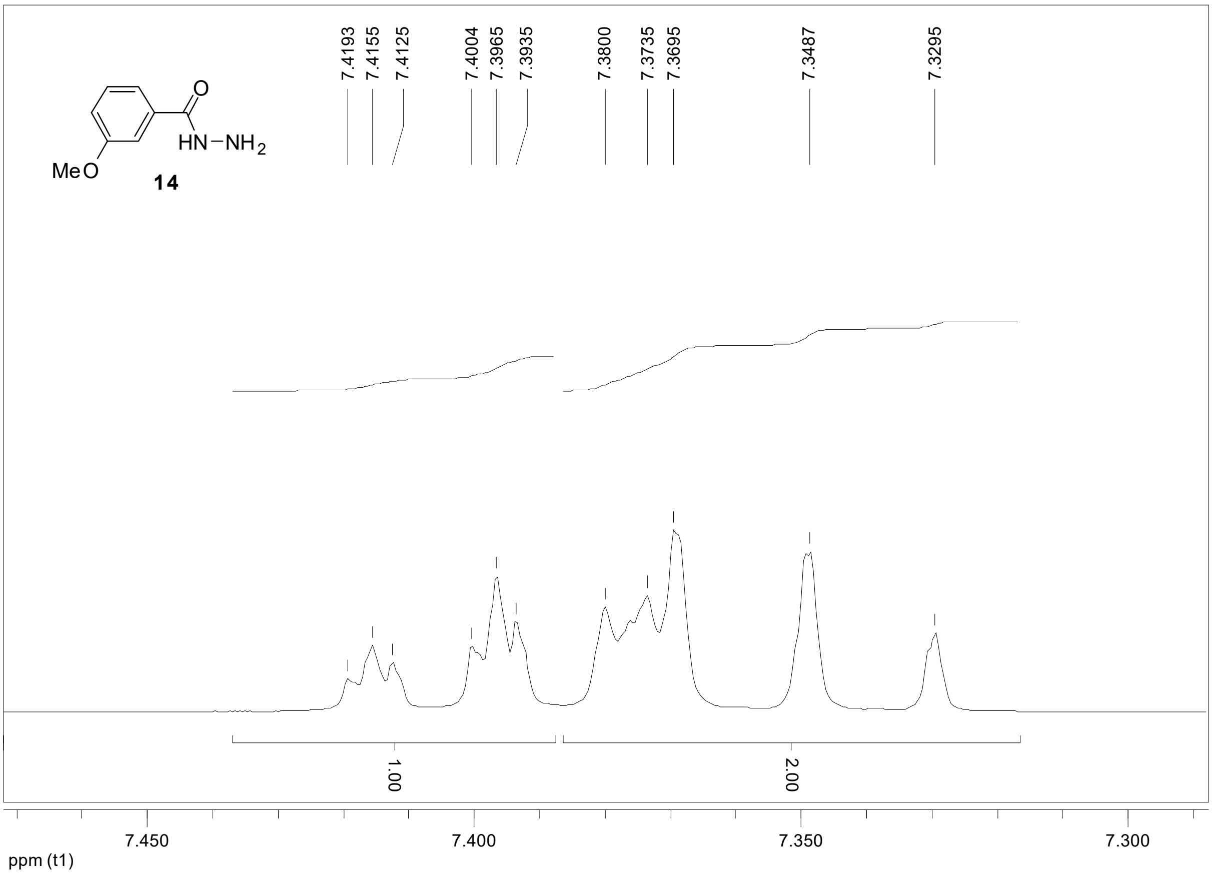

Figure S318. Expansion of ^{1}H-NMR (400 MHz, DMSO-d_6) spectrum of 3-methoxybenzoic acid hydrazide (**14**)

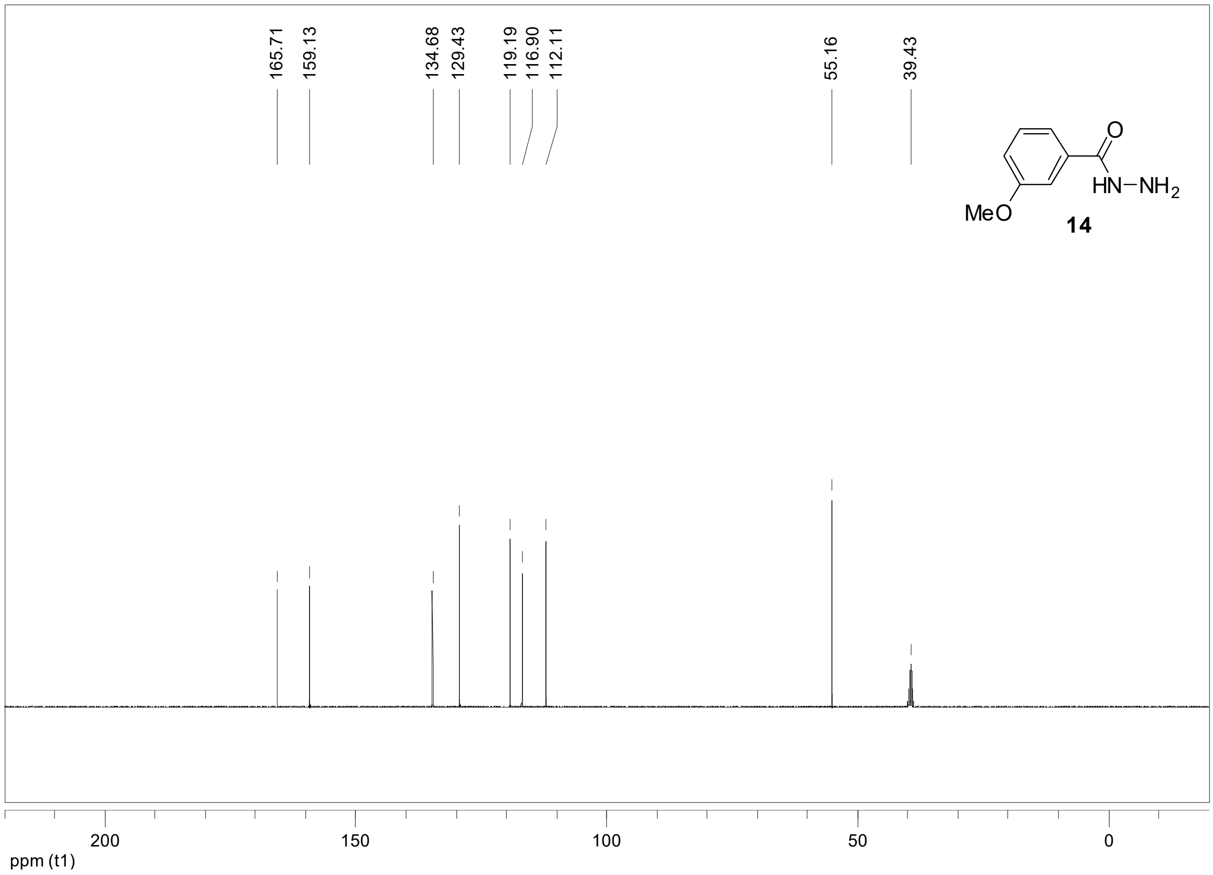

Figure S319. ^{13}C-NMR (100 MHz, DMSO-d_6) spectrum of 3-methoxybenzoic acid hydrazide (**14**)

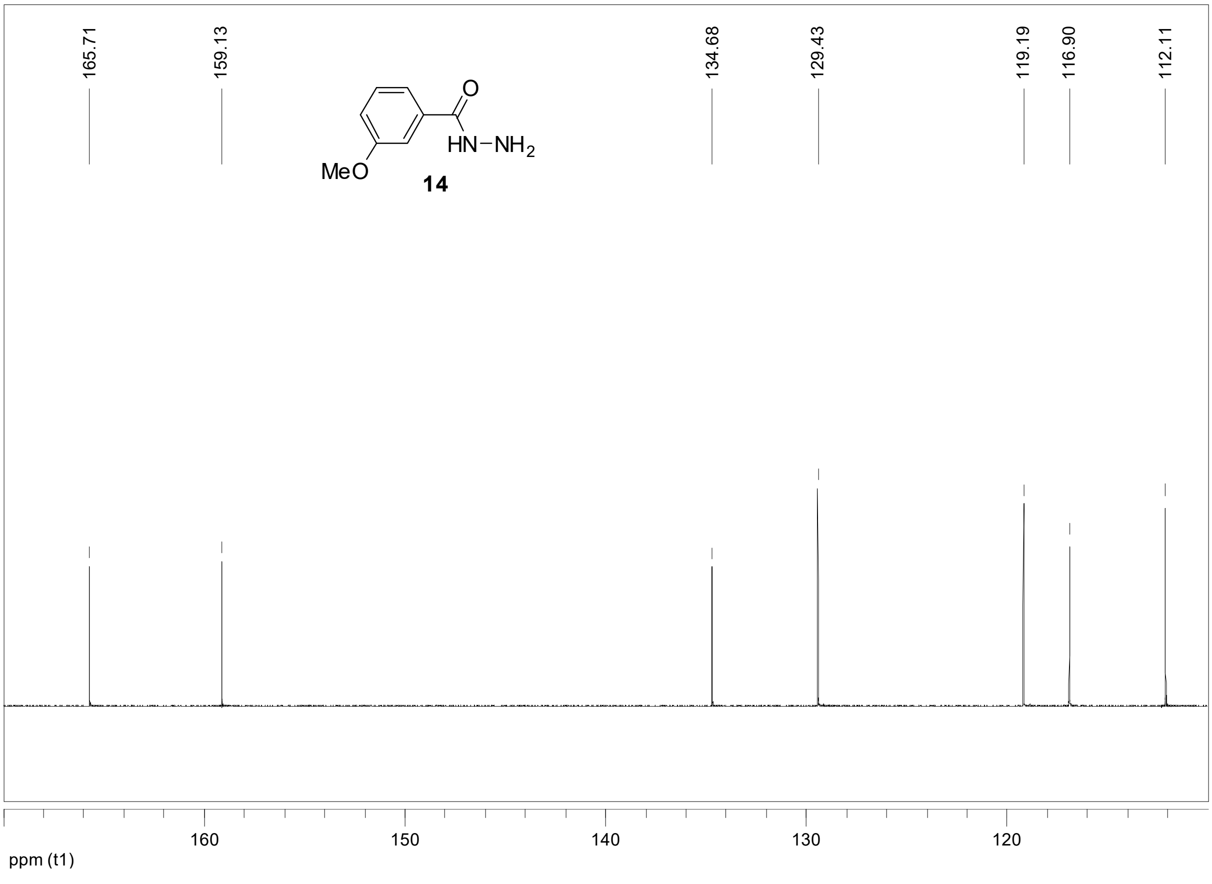

Figure S320. Expansion of ^{13}C-NMR (100 MHz, DMSO-d_6) spectrum of 3-methoxybenzoic acid hydrazide (**14**)

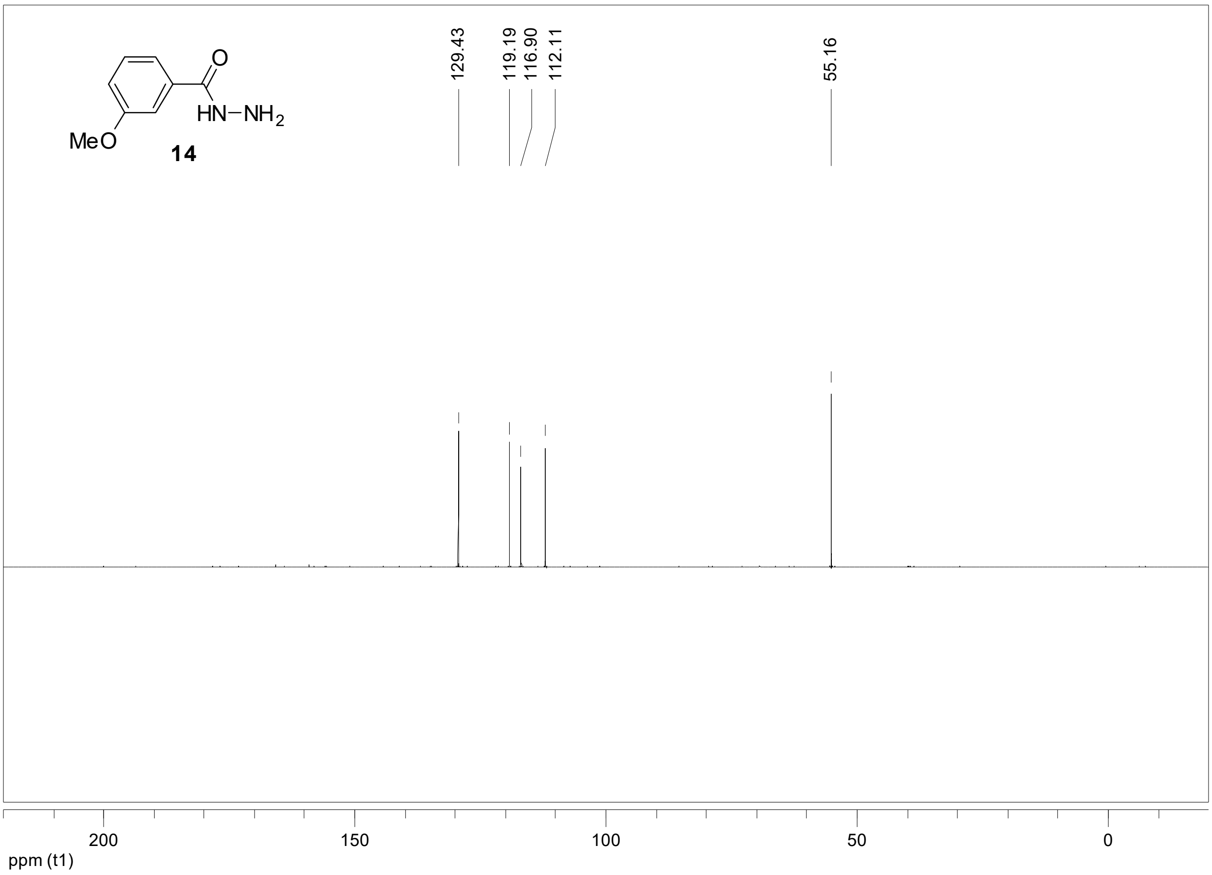

Figure S321. ^{13}C-NMR (100 MHz, DMSO-d_6) dept-135 experiment of 3-methoxybenzoic acid hydrazide (**14**)

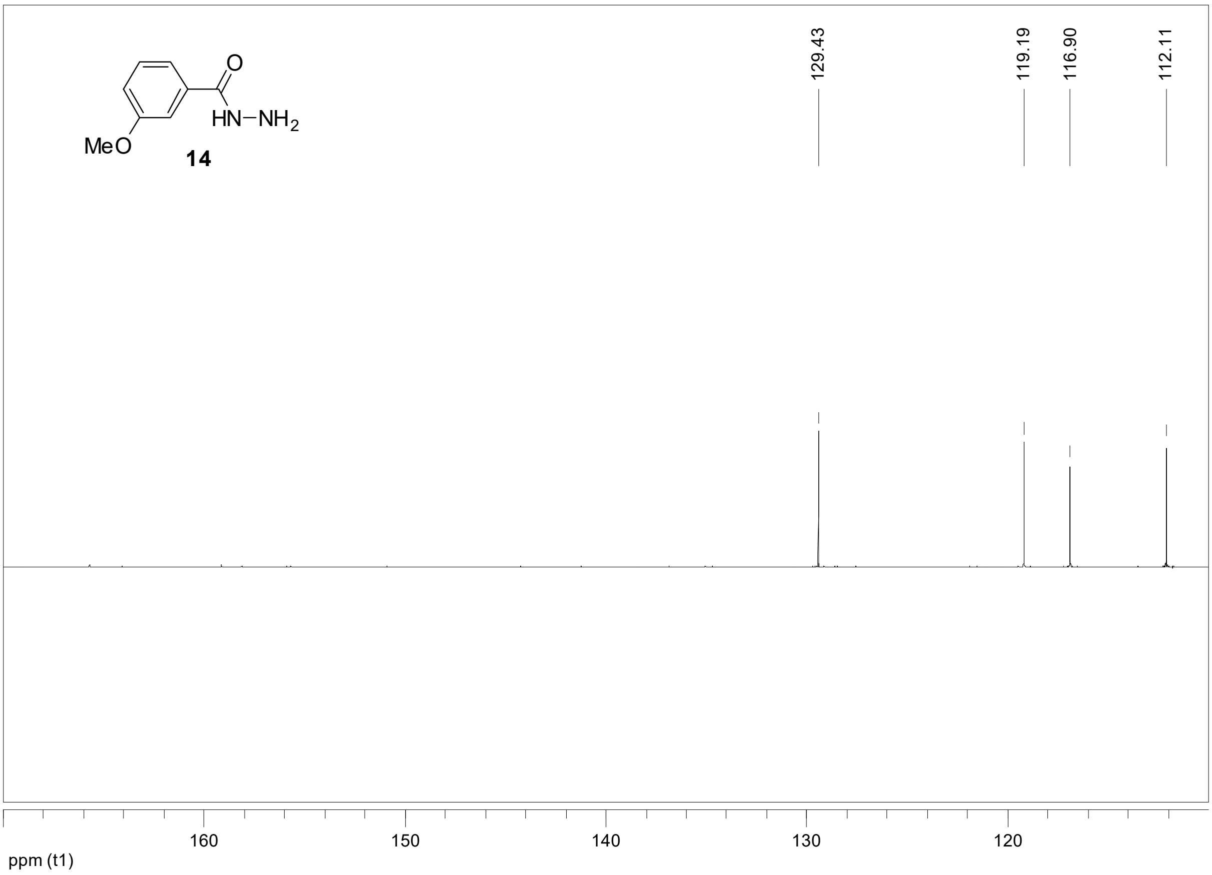

Figure S322. Expansion of ^{13}C-NMR (100 MHz, DMSO-d_6) dept-135 experiment of hydrazide **14**

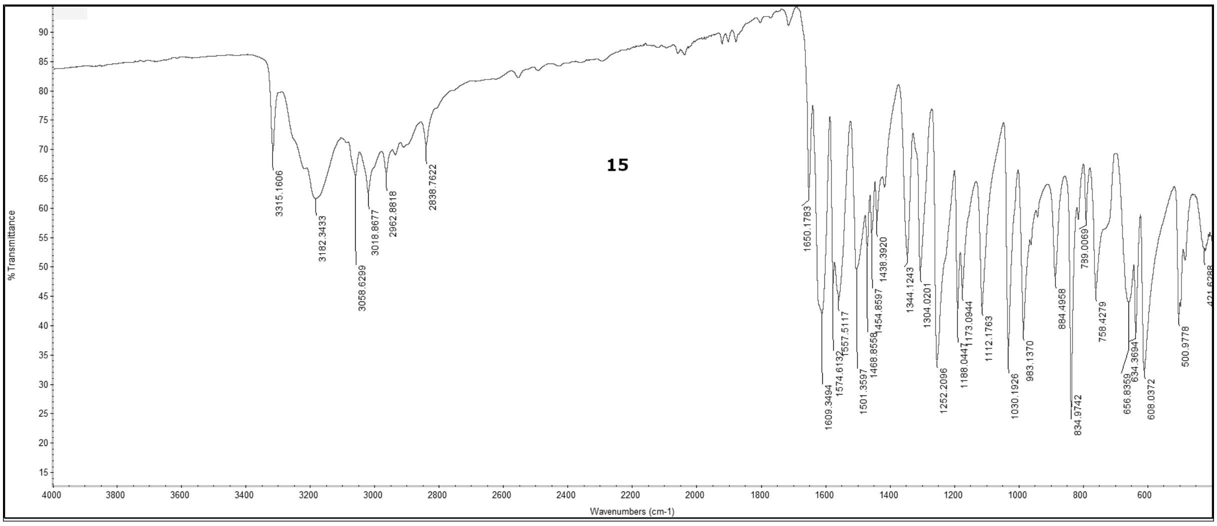

Figure S323. FT-IR (ATR) spectrum of 4-methoxybenzoic acid hydrazide (**15**)

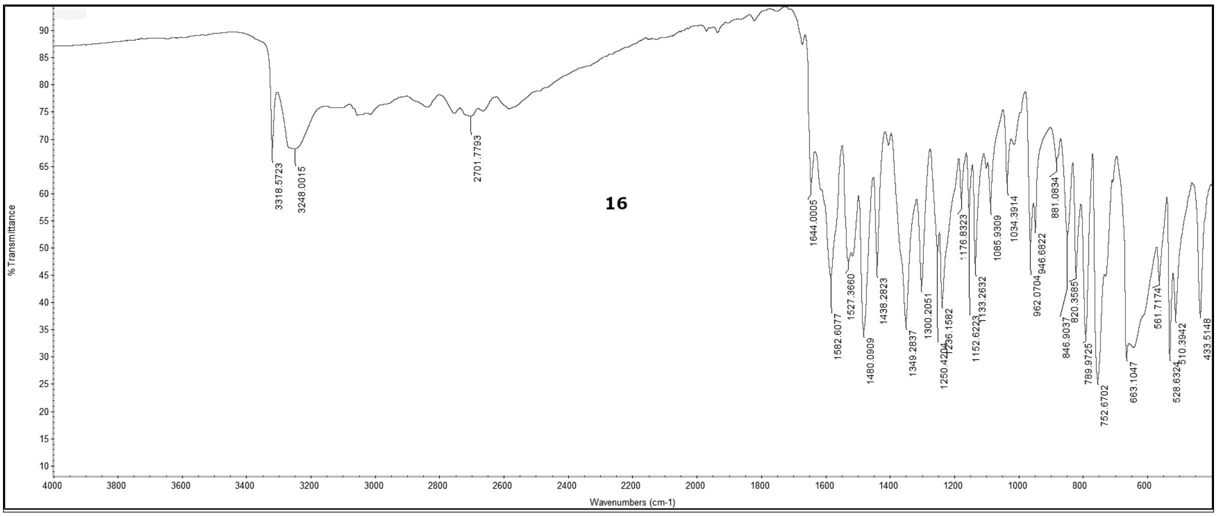

Figure S324. FT-IR (ATR) spectrum of salicylic acid hydrazide (**16**)

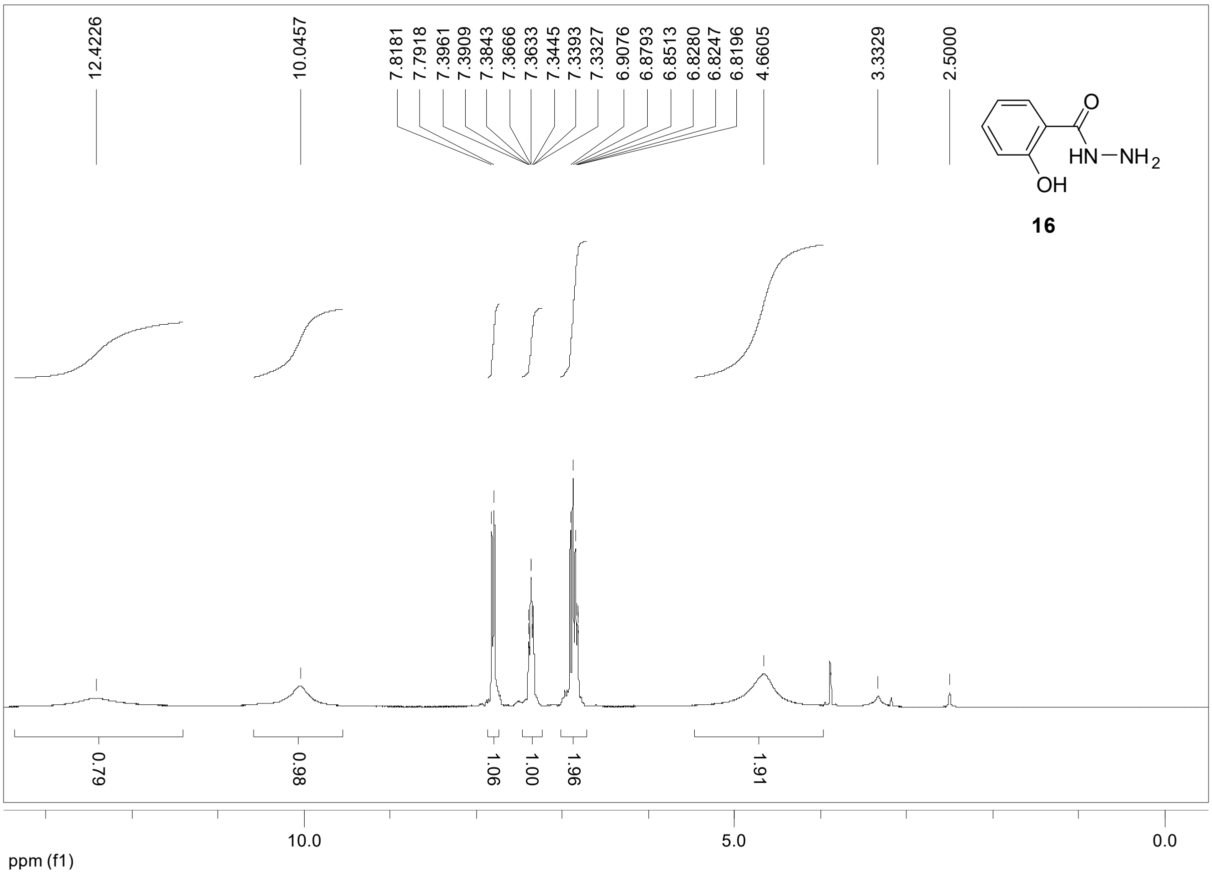

Figure S325. ^{1}H-NMR (300 MHz, DMSO-d_6) spectrum of salicylic acid hydrazide (**16**)

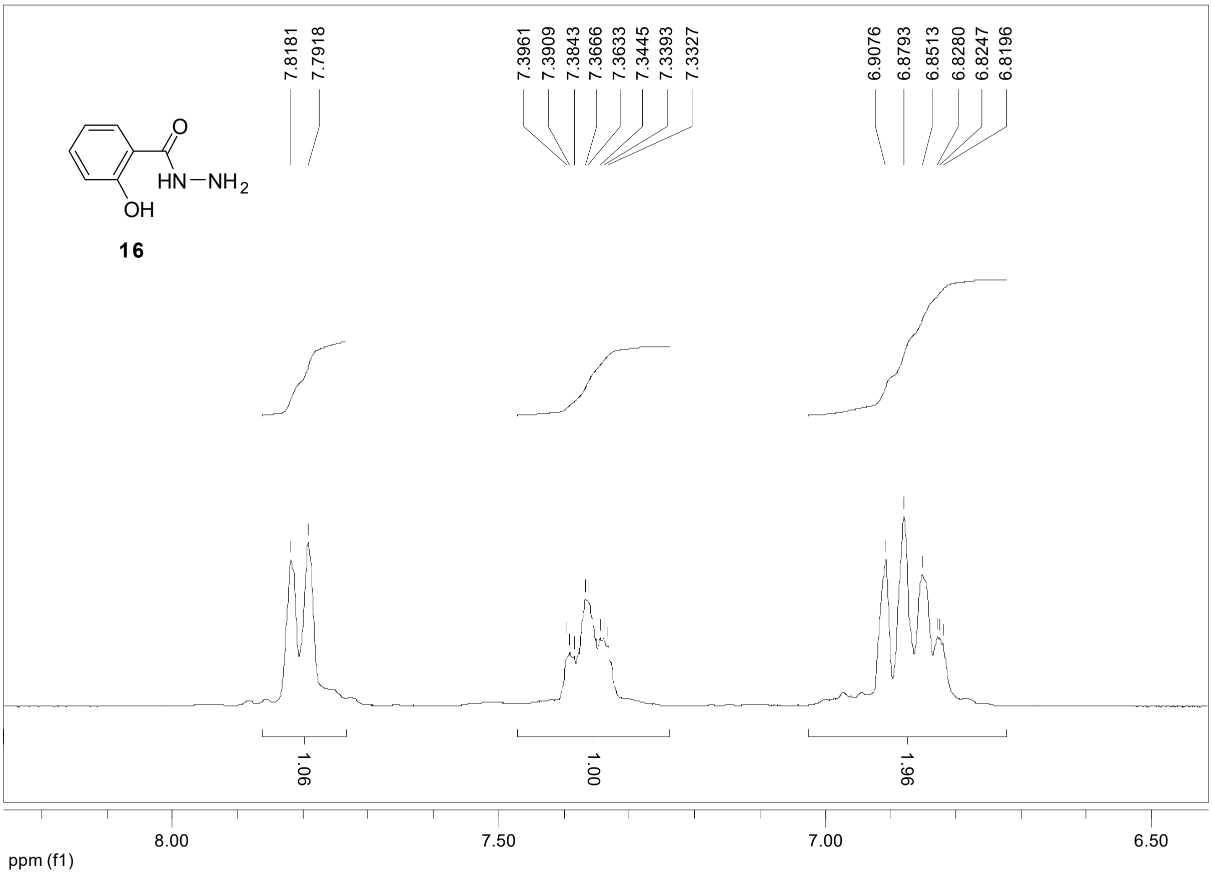

Figure S326. Expansion of ^{1}H-NMR (300 MHz, DMSO-d_6) spectrum of salicylic acid hydrazide (**16**)

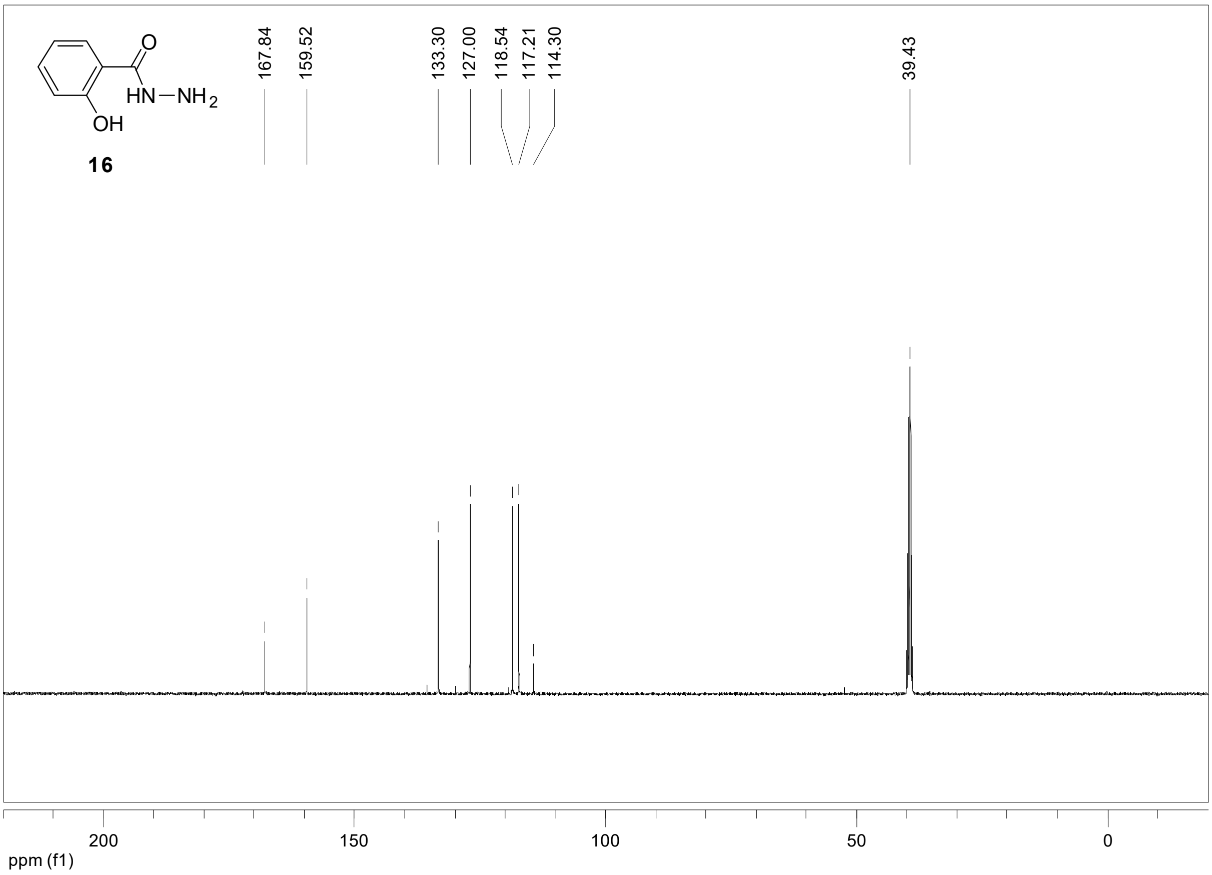

Figure S327. ^{13}C-NMR (100 MHz, DMSO-d_6) spectrum of salicylic acid hydrazide (**16**)

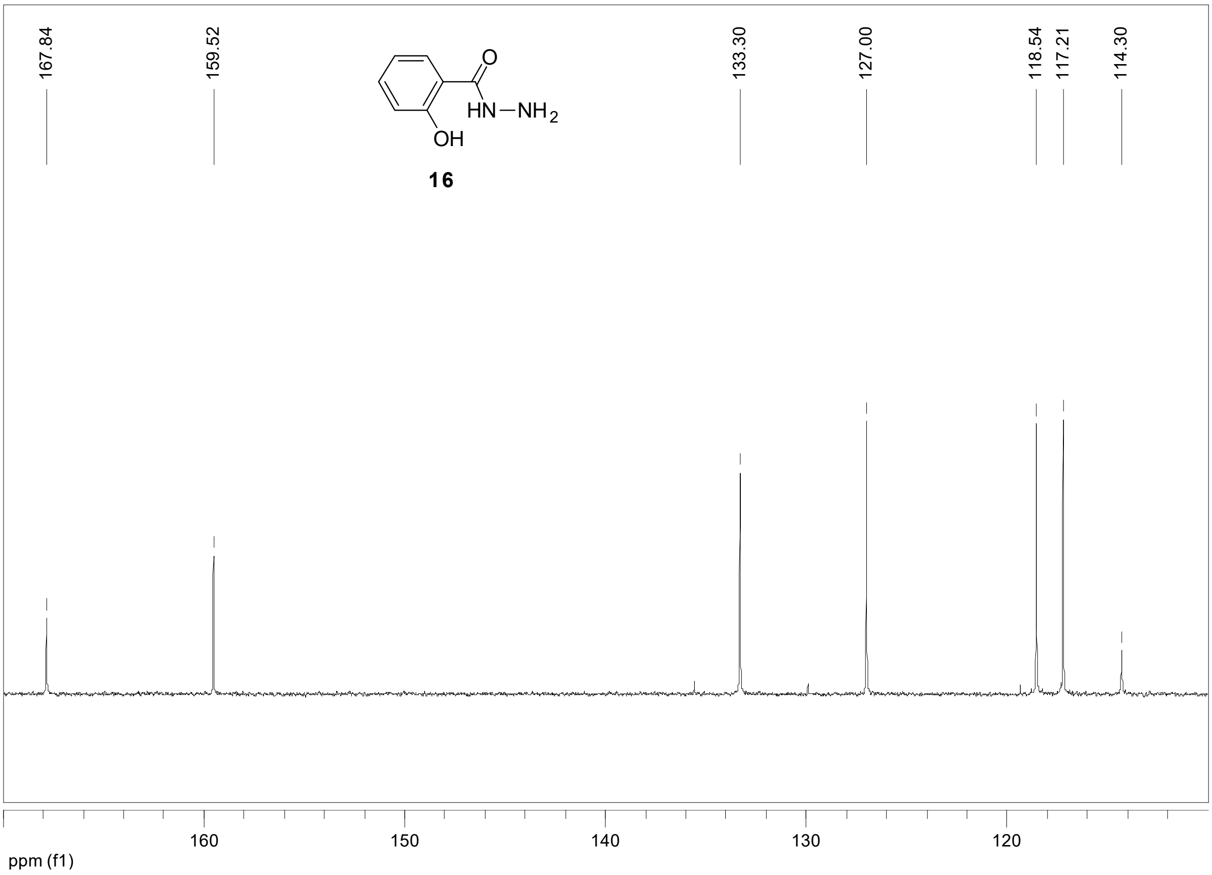

Figure S328. Expansion of ^{13}C-NMR (100 MHz, DMSO-d_6) spectrum of salicylic acid hydrazide (**16**)

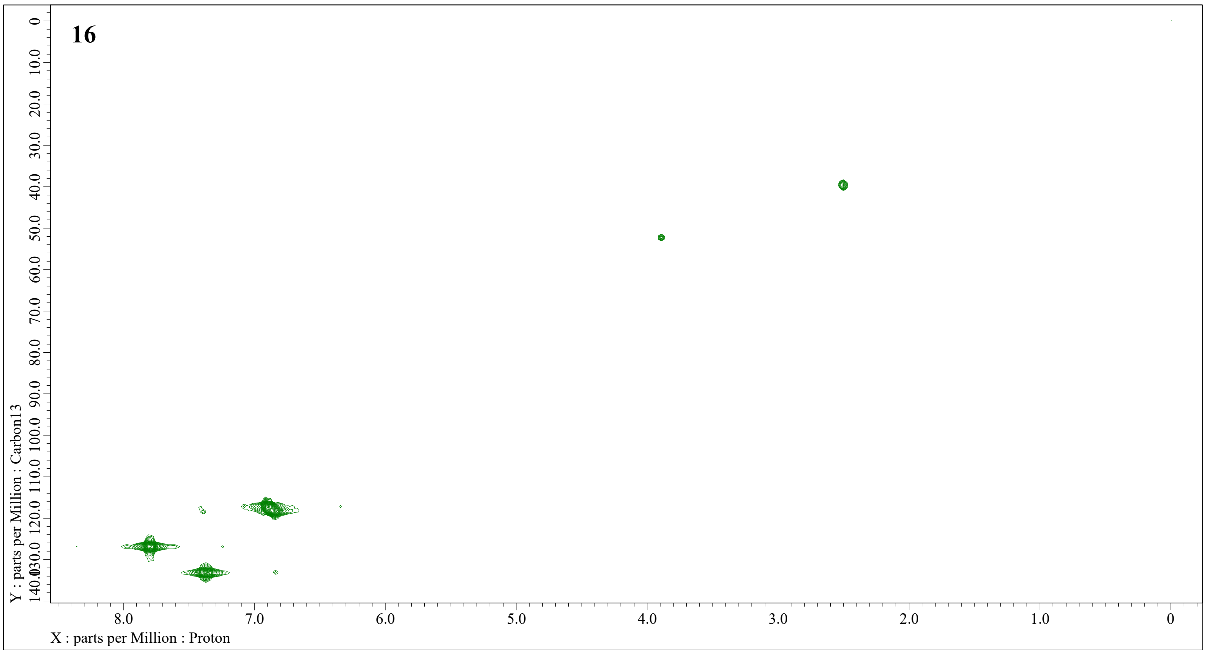

Figure S329. 2D-NMR (100 MHz, DMSO-d_6) HMQC experiment of salicylic acid hydrazide (**16**)

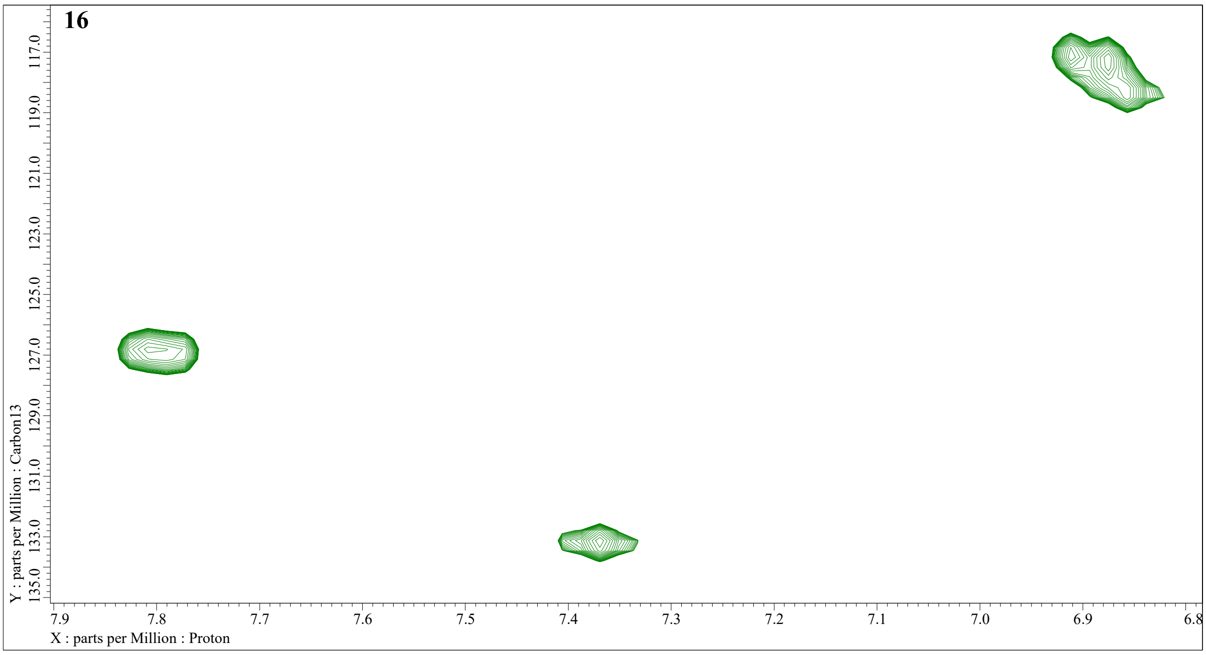

Figure S330. Expansion of 2D-NMR (100 MHz, DMSO-d_6) HMQC experiment of salicylic acid hydrazide (**16**)

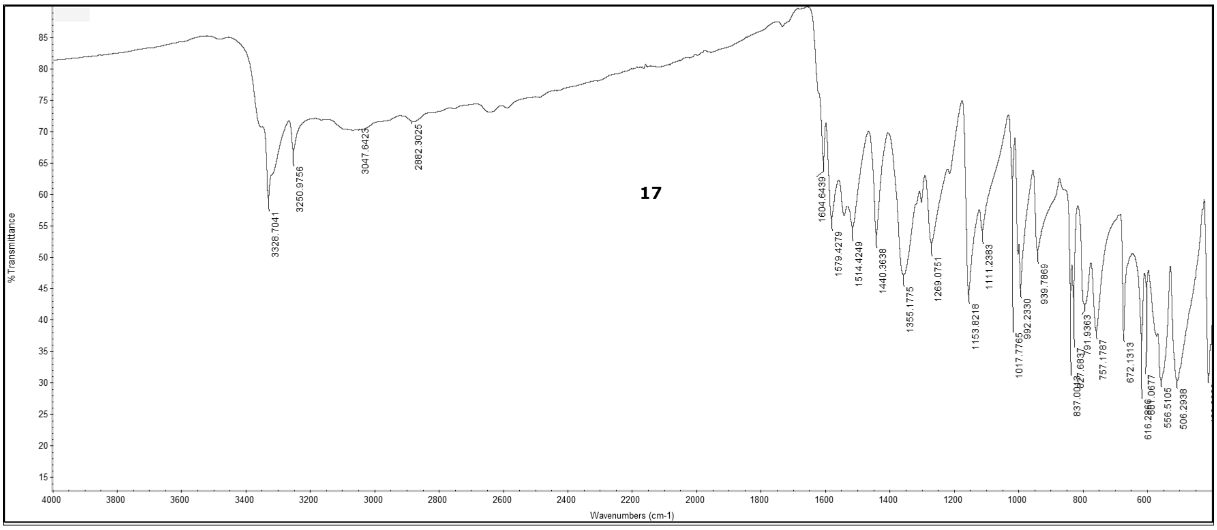

Figure S331. FT-IR (ATR) spectrum of α-resorcylic acid hydrazide (**17**)

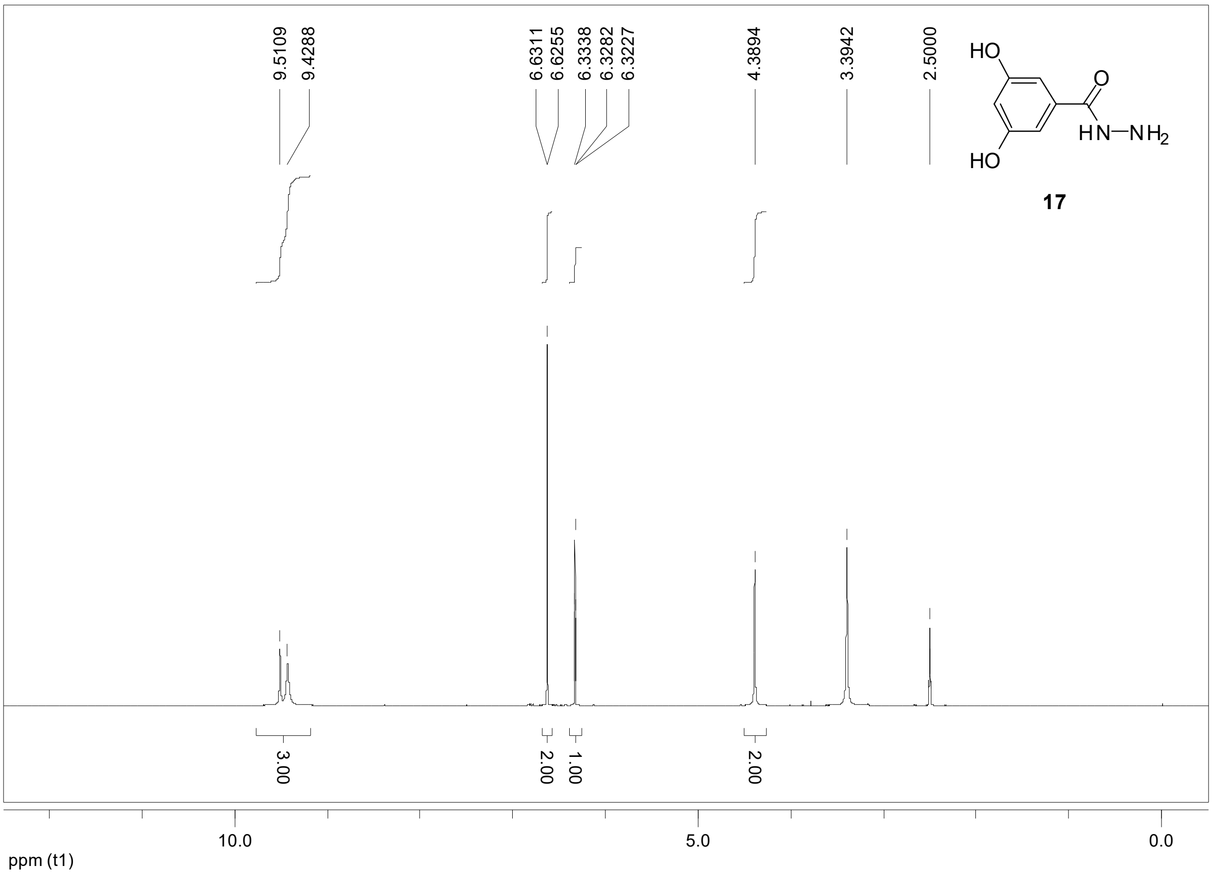

Figure S332. ^{1}H-NMR (400 MHz, DMSO-d_6) spectrum of α-resorcylic acid hydrazide (**17**)

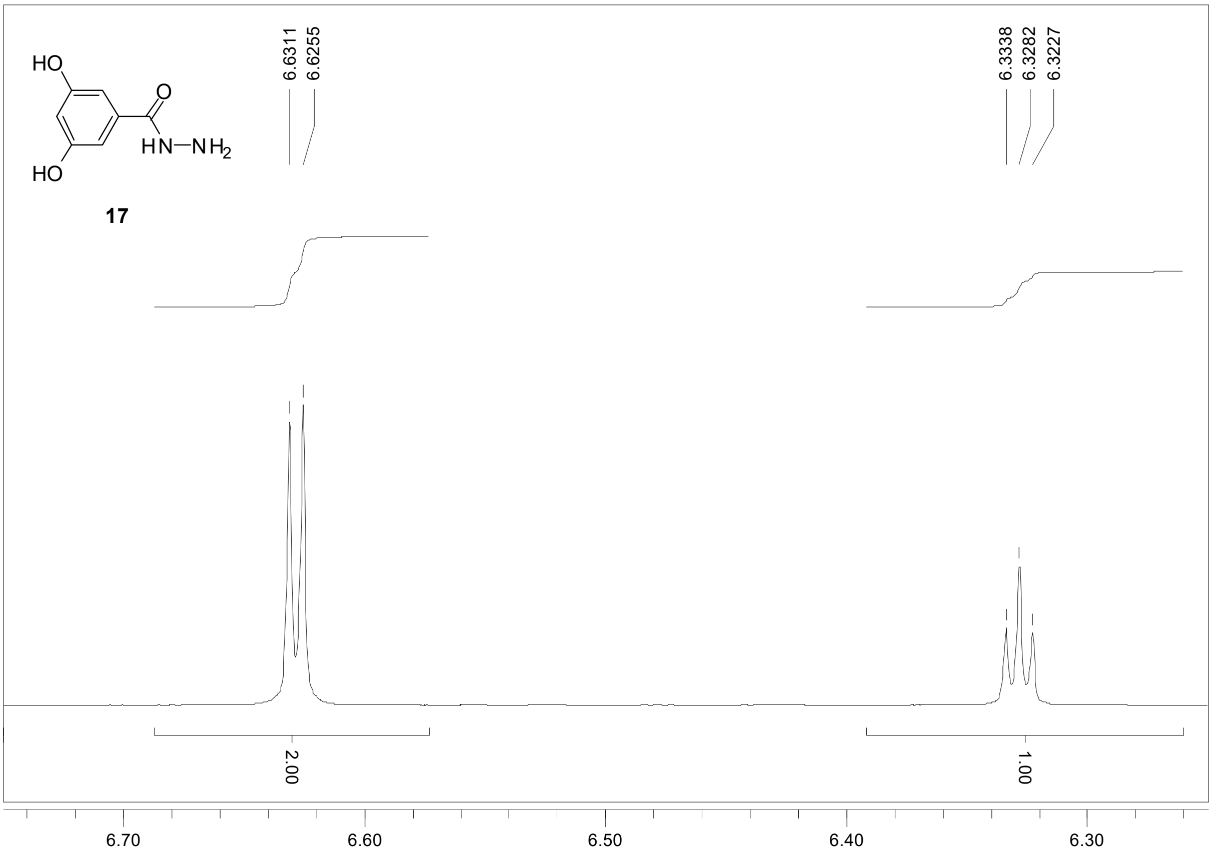

Figure S333. Expansion of ^{1}H-NMR (400 MHz, DMSO-d_6) spectrum of α-resorcylic acid hydrazide (**17**)

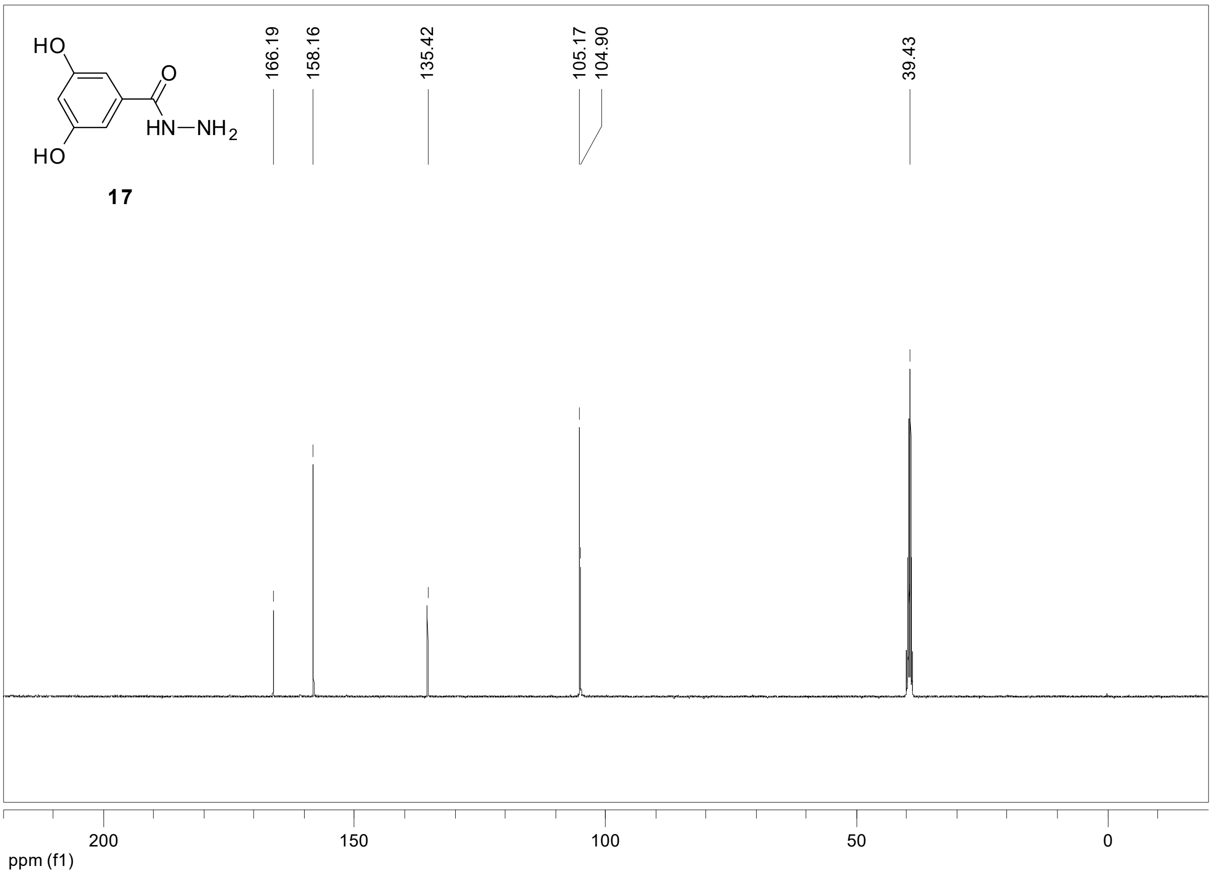

Figure S334. ^{13}C-NMR (100 MHz, DMSO-d_6) spectrum of α-resorcylic acid hydrazide (**17**)

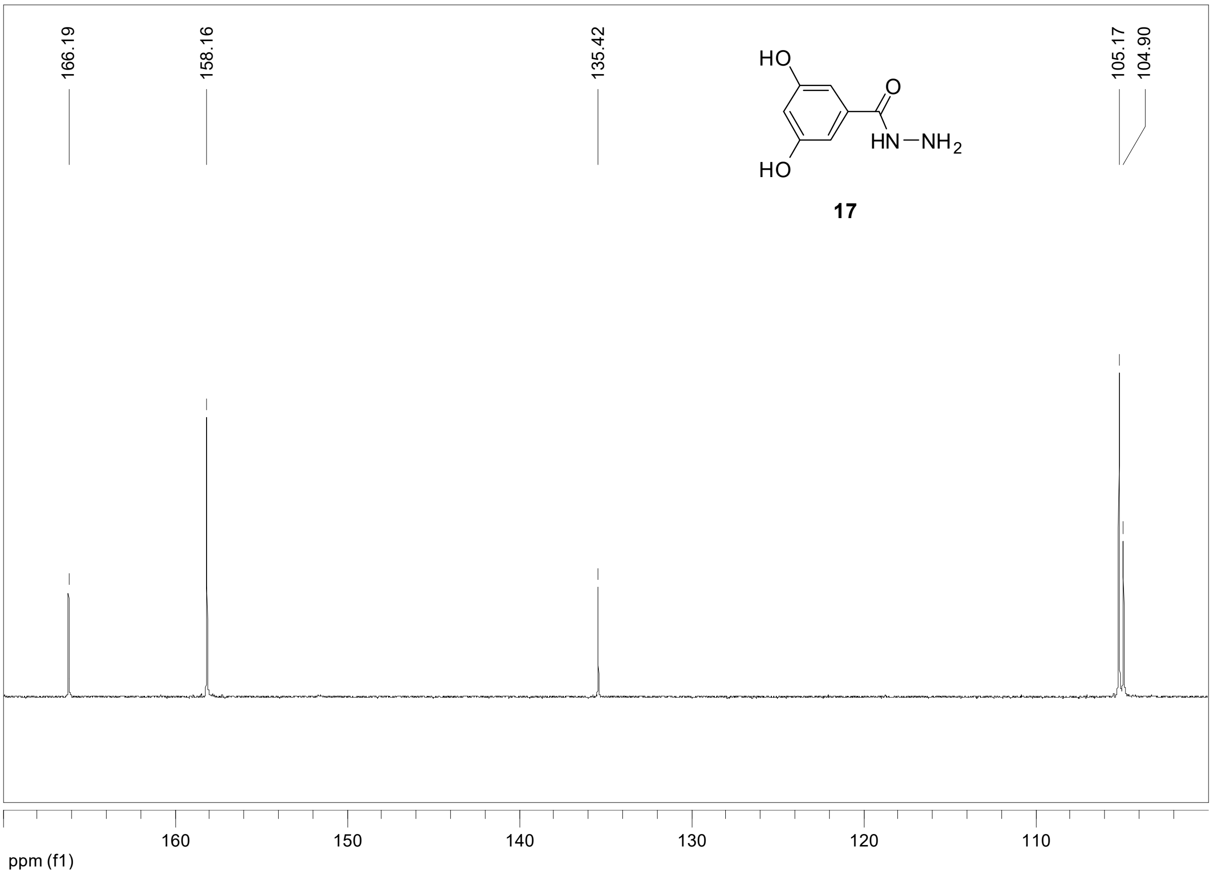

Figure S335. Expansion of ^{13}C-NMR (100 MHz, DMSO-d_6) spectrum of α-resorcylic acid hydrazide (**17**)

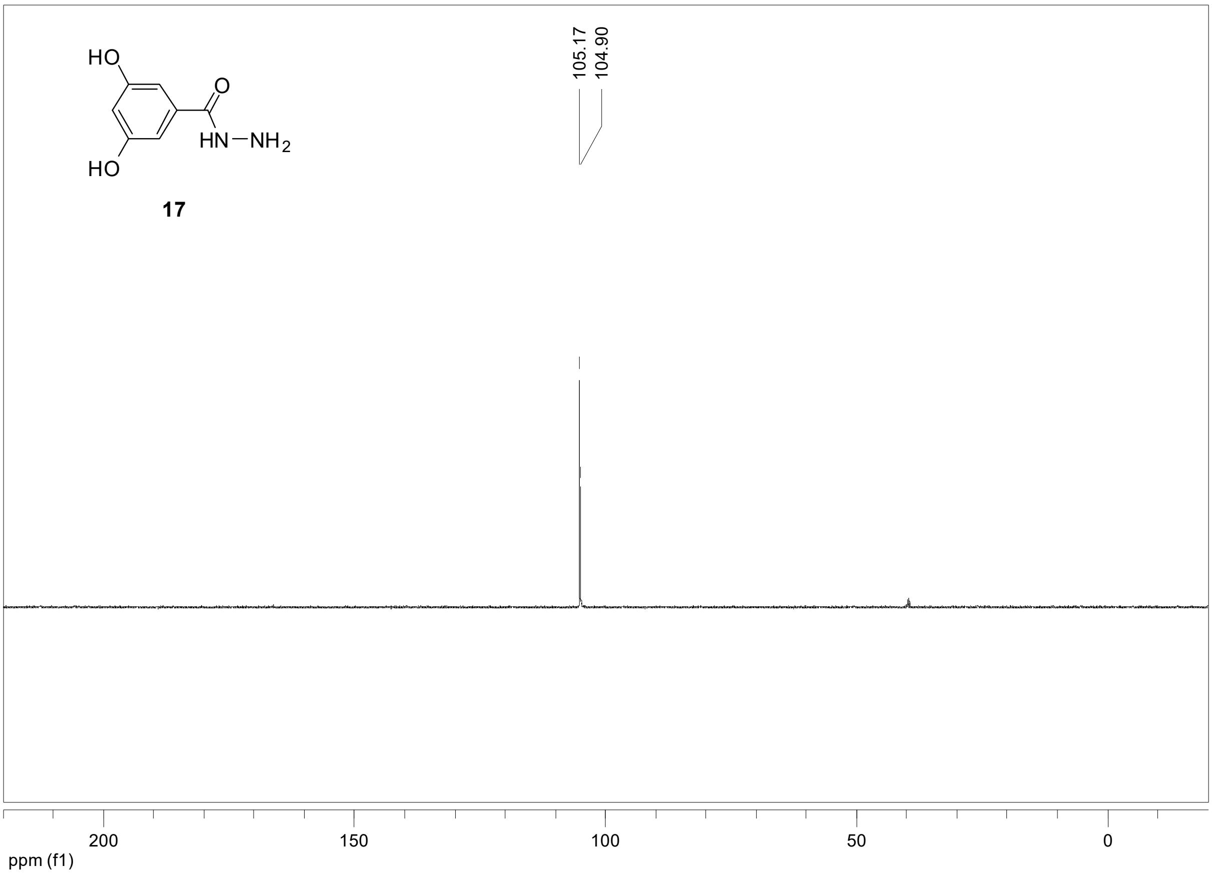

Figure S336. ^{13}C-NMR (100 MHz, DMSO-d_6) dept-135 experiment of α-resorcylic acid hydrazide (**17**)

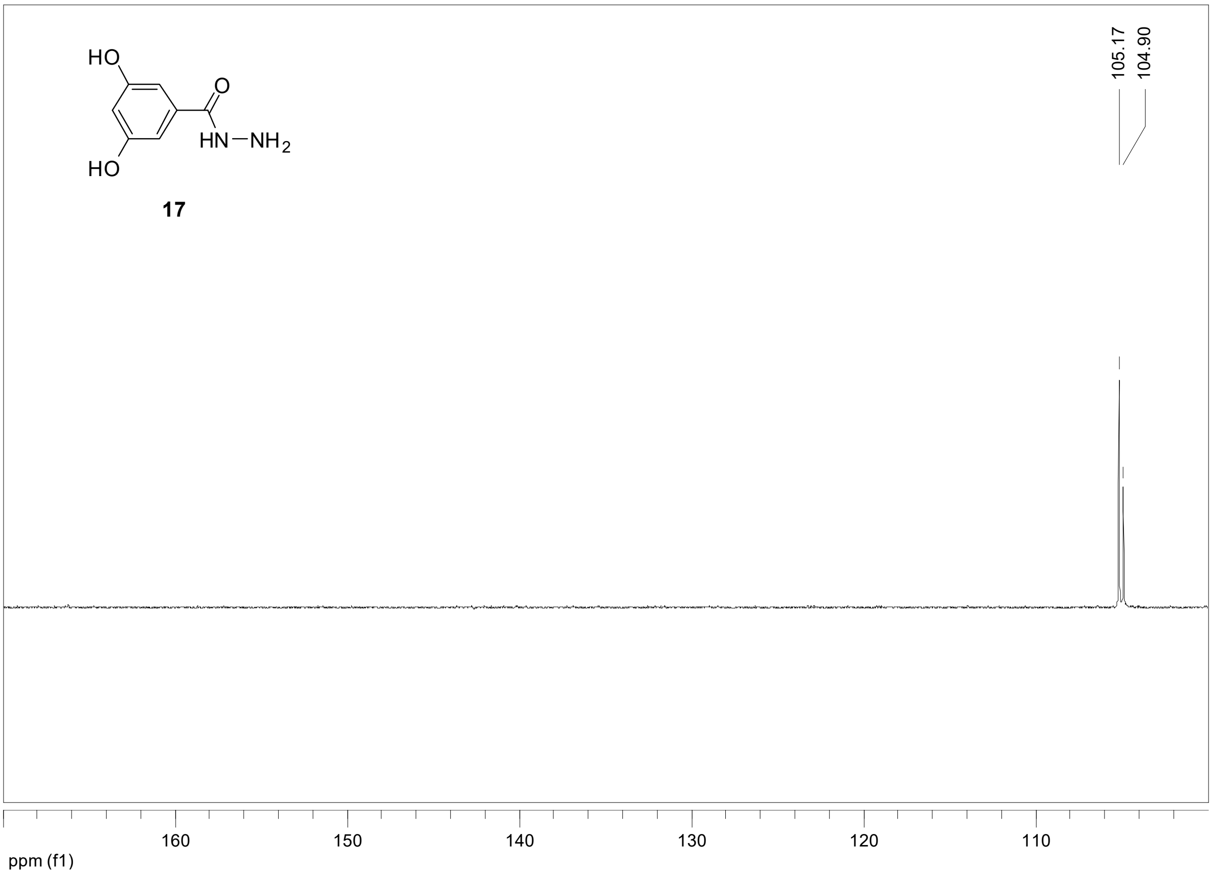

Figure S337. Expansion of ^{13}C-NMR (100 MHz, DMSO-d_6) dept-135 experiment of α-resorcylic hydrazide (**17**)

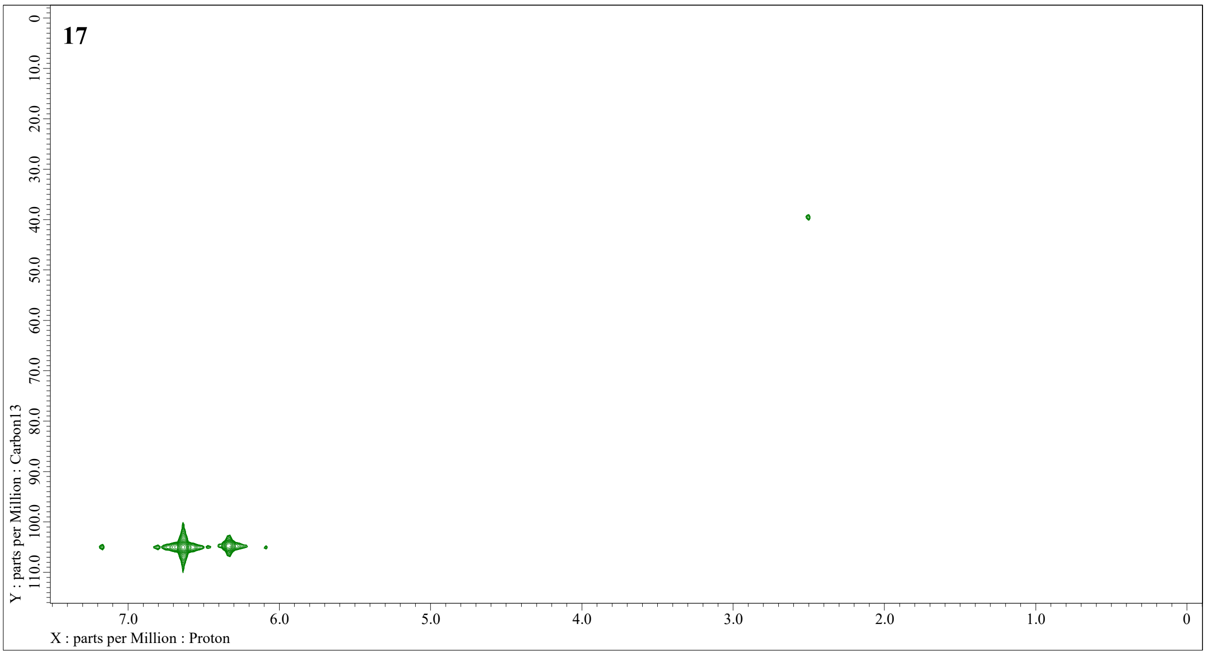

Figure S338. 2D-NMR (400 MHz, DMSO-d_6) HMQC experiment of *α*-resorcylic acid hydrazide (**17**)

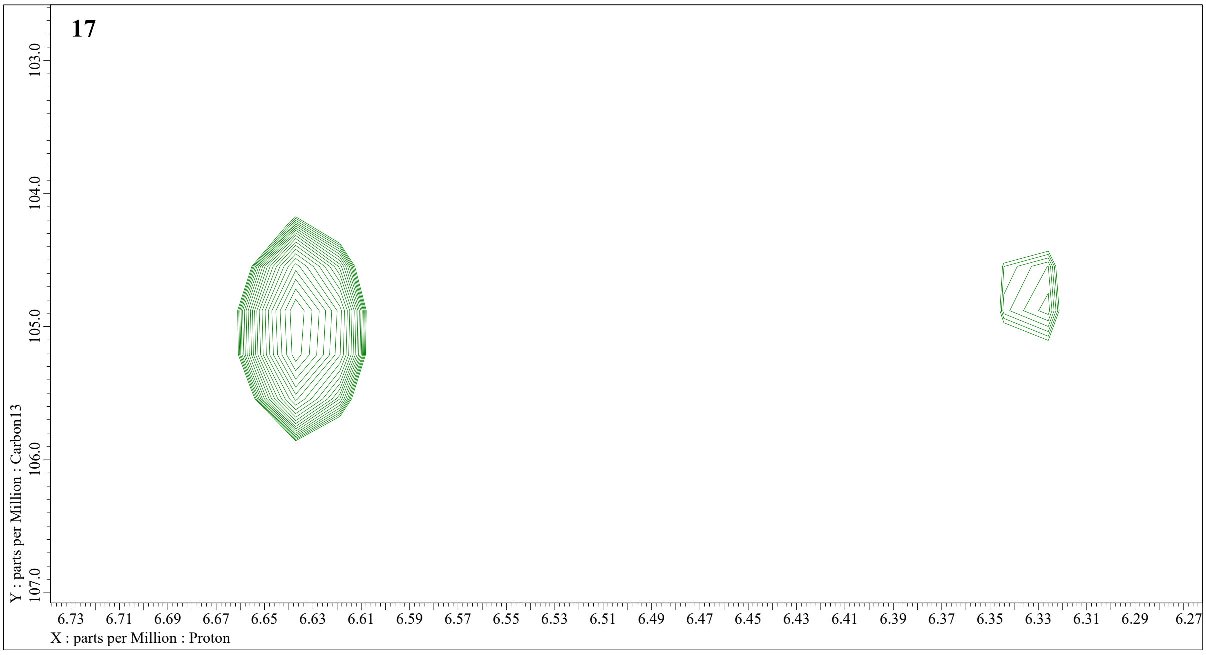

Figure S339. Expansion of 2D-NMR (400 MHz, DMSO-d_6) HMQC experiment of *α*-resorcylic acid hydrazide (**17**)

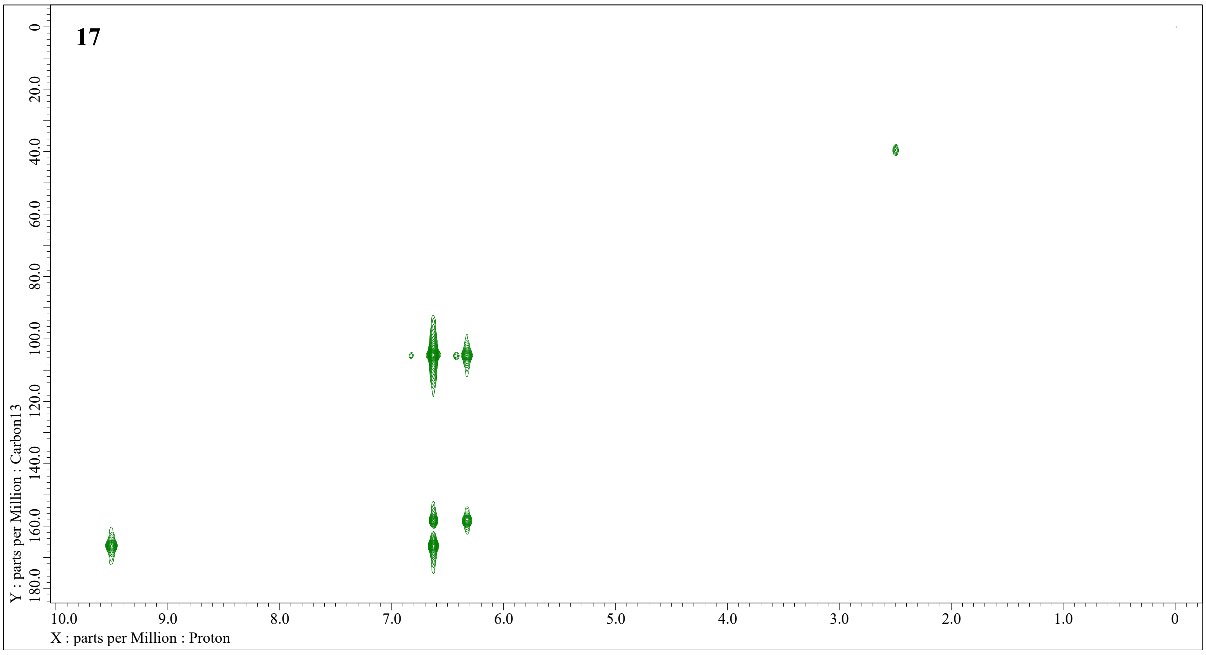

Figure S340. Expansion of 2D-NMR (400 MHz, DMSO-d_6) HMBC experiment of α-resorcylic acid hydrazide (**17**)

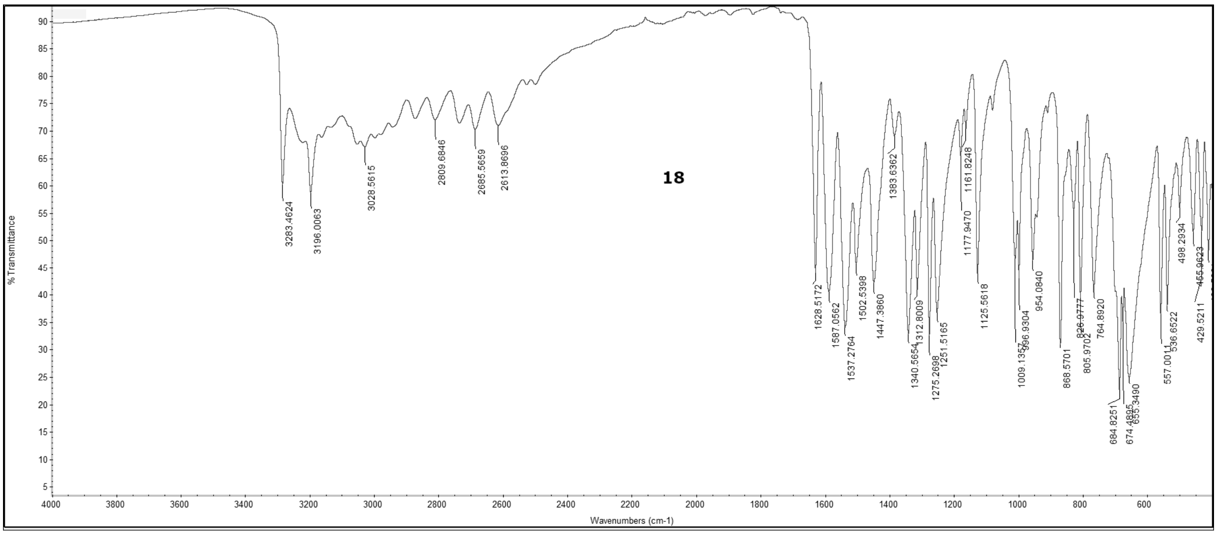

Figure S341. FT-IR (ATR) spectrum of 3-hydroxybenzoic acid hydrazide (**18**)

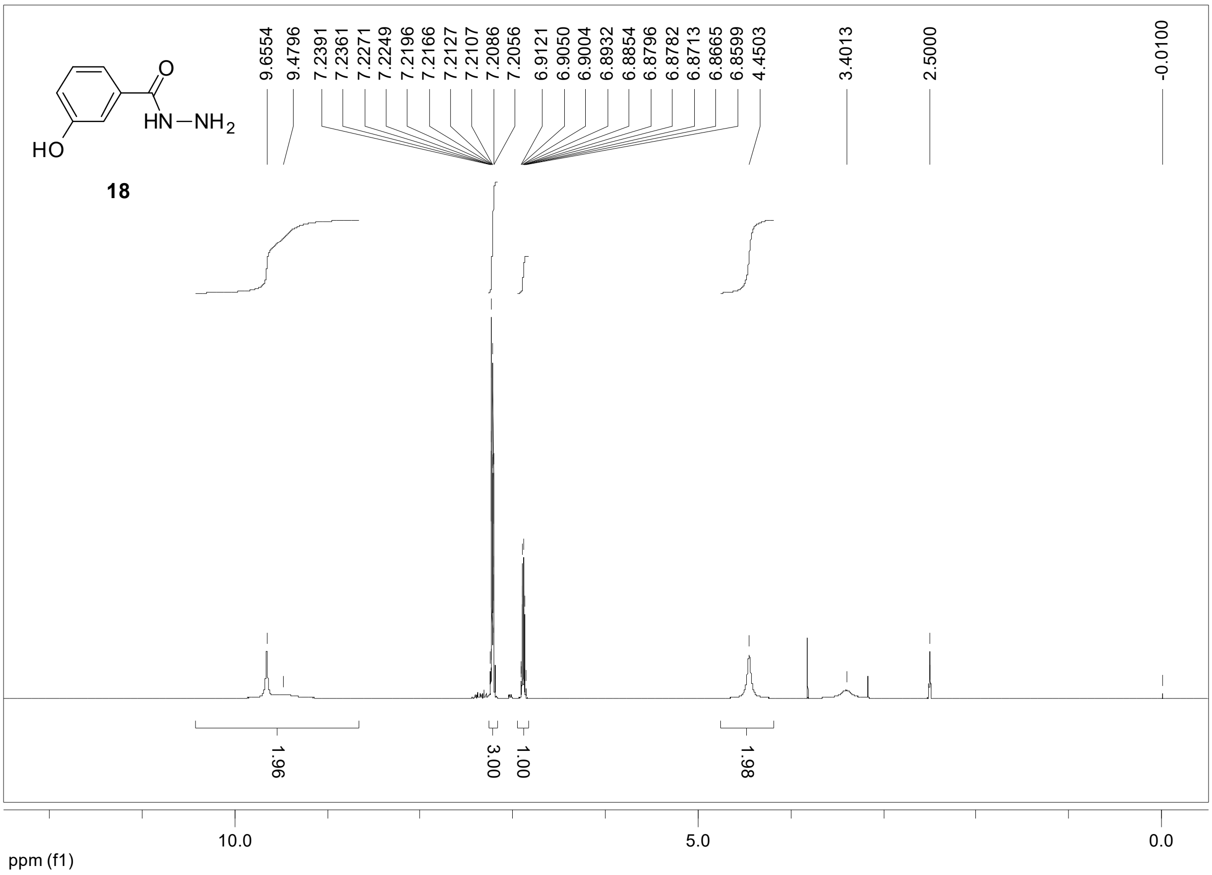

Figure S342. ^{1}H-NMR (400 MHz, DMSO-d_6) spectrum of 3-hydroxybenzoic acid hydrazide (**18**)

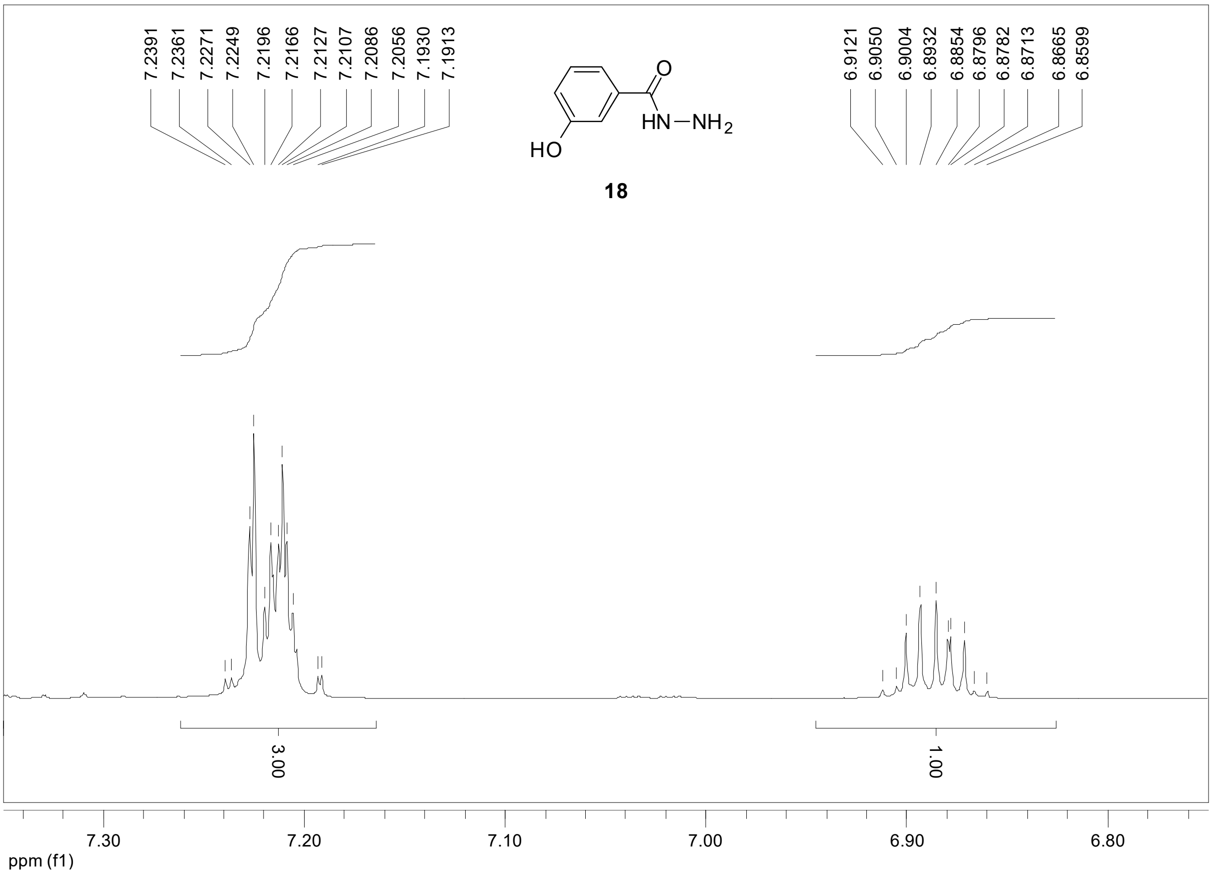

Figure S343. Expansion of ^{1}H-NMR (400 MHz, DMSO-d_6) spectrum of 3-hydroxybenzoic acid hydrazide (**18**)

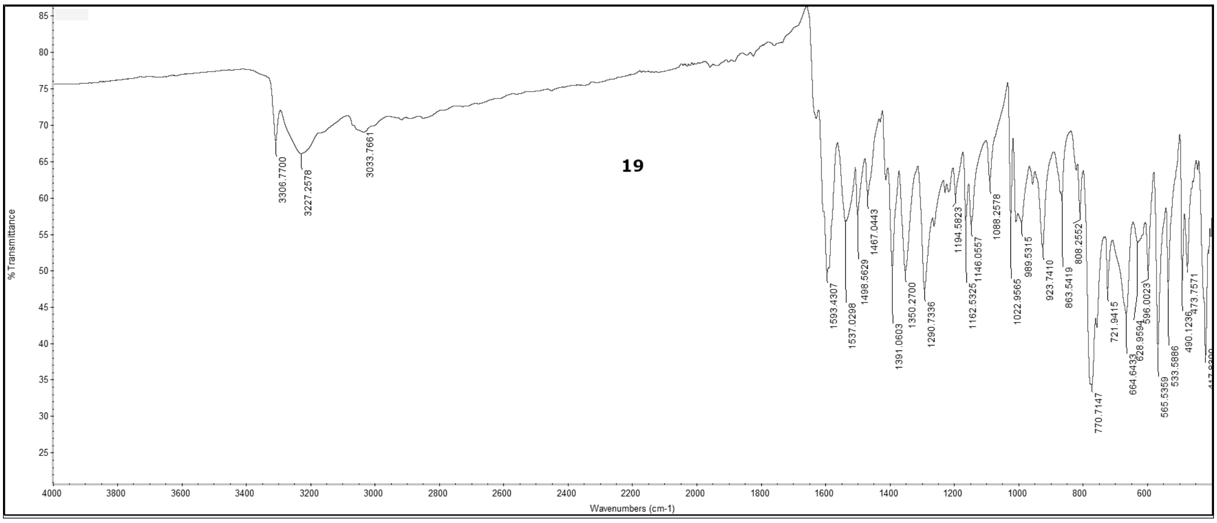

Figure S344. FT-IR (ATR) spectrum of 1-hydroxy-2-naphthoic acid hydrazide (**19**)

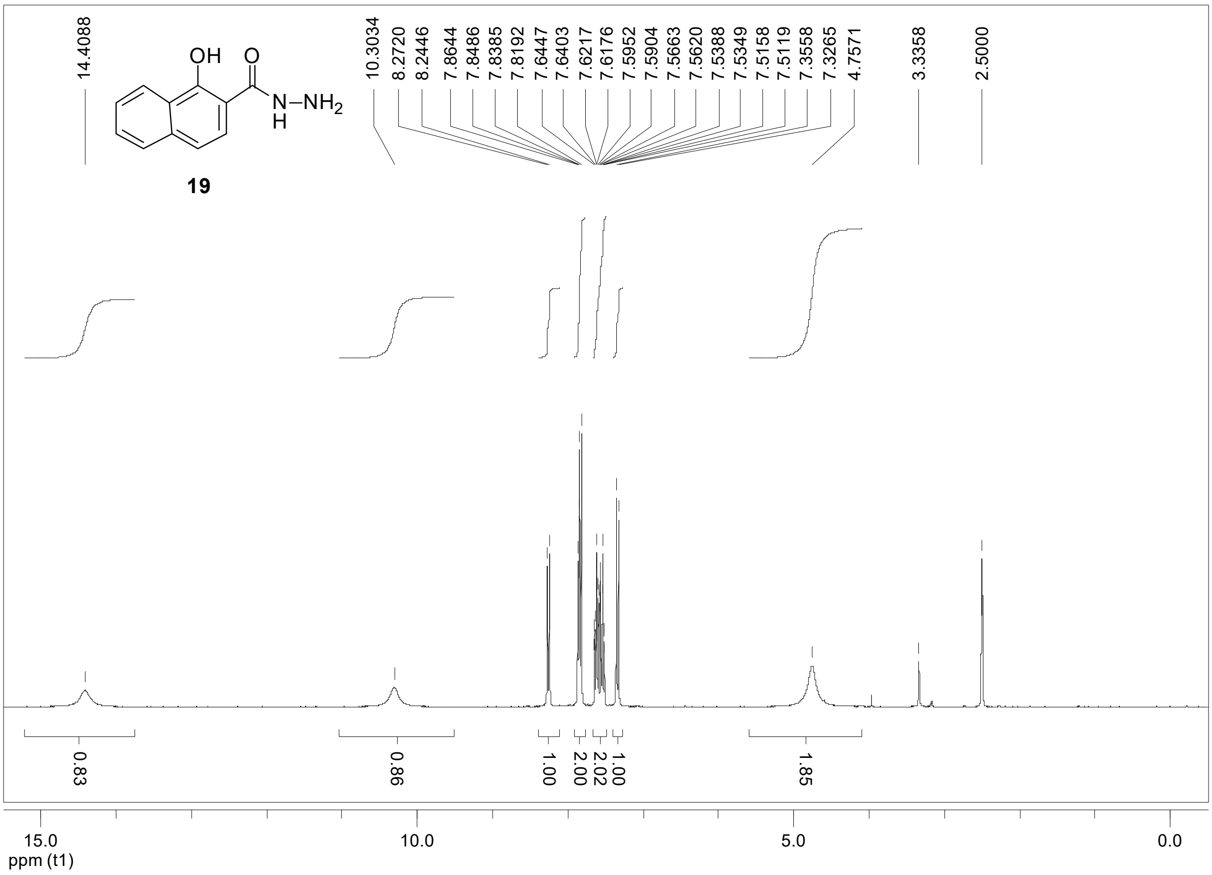

Figure S345. ^{1}H-NMR (300 MHz, DMSO-d_6) spectrum of 1-hydroxy-2-naphthoic acid hydrazide (**19**)

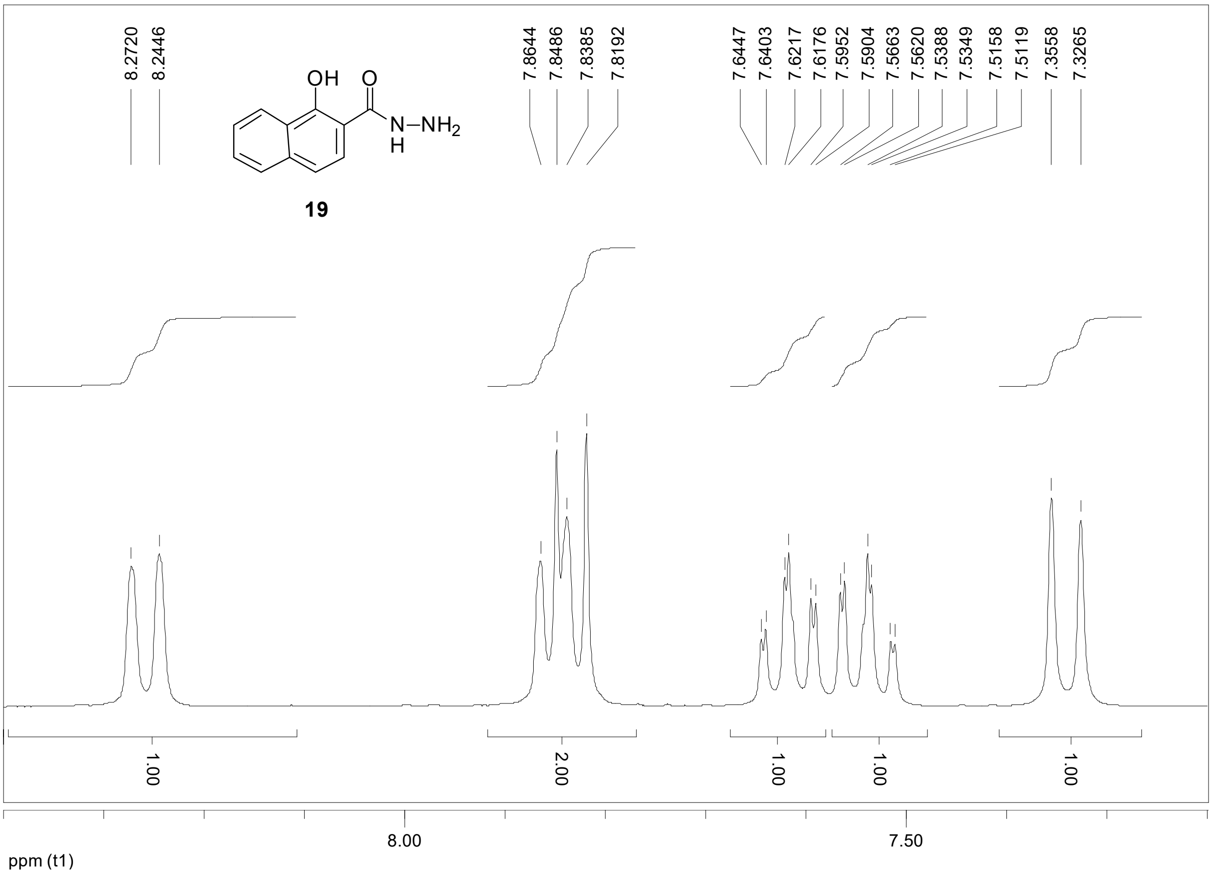

Figure S346. Expansion of ^{1}H-NMR (300 MHz, DMSO-d_6) spectrum of hydroxy-2-naphthoic acid hydrazide **19**

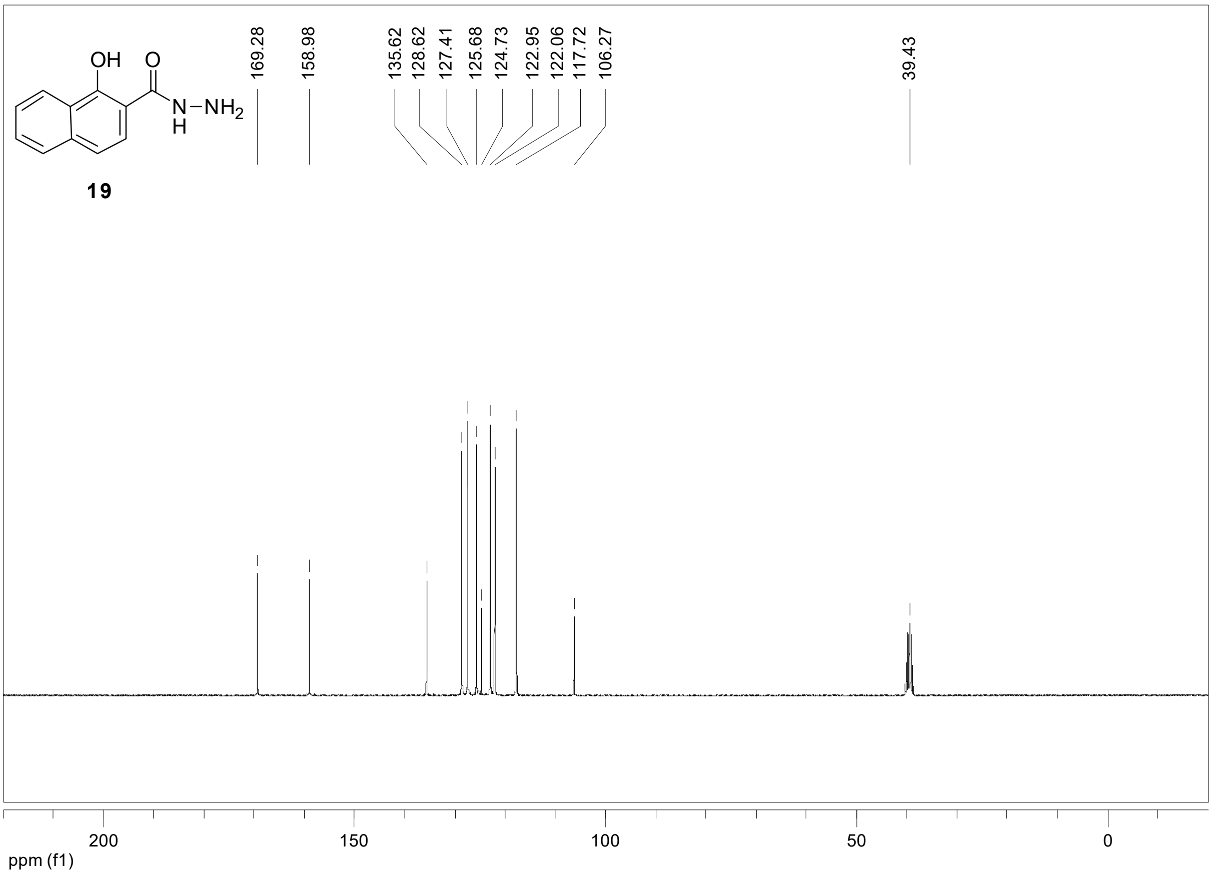

Figure S347. ^{13}C-NMR (75 MHz, DMSO-d_6) spectrum of 1-hydroxy-2-naphthoic acid hydrazide (**19**)

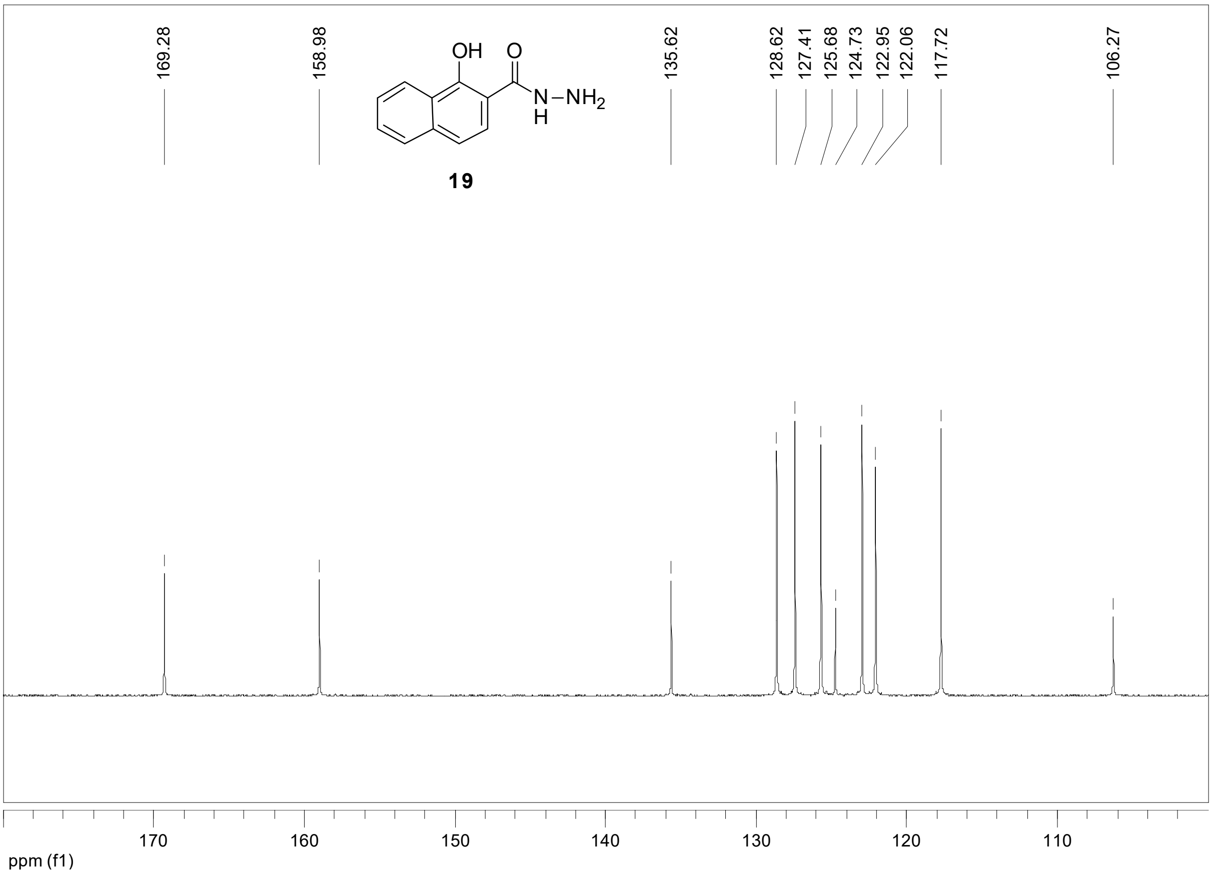

Figure S348. Expansion of ^{13}C-NMR (75 MHz, DMSO-d_6) spectrum of hydroxy-2-naphthoic acid hydrazide **19**

1. References

1. Khusnutdinov, R.I.; Shchadneva, N.A.; Mayakova, Y.Y. Methylation of aliphatic and aromatic carboxylic acids with dimethyl carbonate under the influence of manganese and iron carbonyls. *Russ. J. Gen. Chem.* **2018**, *88*, 15–19, doi:10.1134/S1070363218010036.

2. Bai, X.; Ye, F.; Zheng, L.; Lai, G.; Xia, C. Hydrosilane and bismuth-accelerated palladium catalyzed aerobic oxidative esterification of benzylic alcohols with air. *Chem. Commun.* **2012**, *48*, 8592, doi:10.1039/C2CC34117D.

3. Maniak, H.; Talma, M.; Matyja, K.; Trusek, A.; Giurg, M. Synthesis and structure-activity relationship studies of hydrazide-hydrazones as inhibitors of laccase from Trametes versicolor. *Molecules* **2020**, *25*, 1255, doi:10.3390/molecules25051255.

4. Dias, L.C.; Polo, E.C. Nhatrangin A: Total syntheses of the proposed structure and six of its diastereoisomers. *J. Org. Chem.* **2017**, *82*, 4072–4112, doi:10.1021/acs.joc.6b03060.

5. Giurg, M.; Muchalski, H.; Kowal, E. Oxofunctionalized trans-2-carboxycinnamic acids by catalytic domino oxidation of naphthols and hydronaphthoquinones. *Synth. Commun.* **2012**, *42*, 2526–2539, doi:10.1080/00397911.2011.561945.

6. Tien, C.N.; Van, T.N.; Duc, G. Le; Quoc, M.V. Synthesis, structure and in vitro cytotoxicity testing of some 1,3,4-oxadiazoline derivatives from 2-hydroxy-5-iodobenzoic acid. *Acta Cryst. C* **2018**, *C74*, 839–846, doi:10.1107/S2053229618008719.

7. Nolla-saltiel, R.; Robles-marín, E.; Porcel, S. Silver(I) and gold(I)-promoted synthesis of alkylidene lactones and 2H-chromenes from salicylic and anthranilic acid derivatives. *Tetrahedron Lett.* **2014**, *55*, 4484–4488, doi:10.1016/j.tetlet.2014.06.060.

8. Bica, K.; Rogers, R.D. Confused ionic liquid ions – A “liquification” and dosage strategy for pharmaceutically active salts. *Chem. Commun.* **2010**, *8*, 1215–1217, doi:DOI https://doi.org/10.1039/B925147B.

9. Said, S.B.; Skarzewski, J.; Mlochowski, J. Oxidative conversion of aldoximes into carboxylic acid esters. *Synth. Commun.* **1992**, *22*, 1851–1862, doi:10.1080/00397919208021316.

10. Harnying, W.; Sudkaow, P.; Biswas, A.; Berkessel, A. N-Heterocyclic carbene/carboxylic acid co-catalysis enables oxidative esterification of demanding aldehydes/enals, at low catalyst loading. *Angew. Chemie - Int. Ed.* **2021**, *60*, 19631–19636, doi:10.1002/anie.202104712.

11. Zhu, B.; Wang, J.R.; Zhang, Q.; Mei, X. Improving dissolution and photostability of vitamin K3 via cocrystallization with naphthoic acids and sulfamerazine. *Cryst. Growth Des.* **2016**, *16*, 483–492, doi:10.1021/acs.cgd.5b01491.

12. Wang, Y.; Gevorgyan, V. General method for the synthesis of salicylic acids from phenols through palladium-catalyzed silanol-directed C-H carboxylation. *Angew. Chemie - Int. Ed.* **2015**, *54*, 2255–2259, doi:10.1002/anie.201410375.

13. Zhang, J.; Zhang, J.; Kang, Y.; Shi, J.; Yao, C. A facile and practical total synthetic route for ampelopsin F and permethylated ε-viniferin. *Synlett* **2016**, *27*, 1587–1591, doi:10.1055/s-0035-1561419.

14. Nomura, N.; Ishii, R.; Yamamoto, Y.; Kondo, T. Stereoselective ring-opening polymerization of a racemic lactide by using achiral salen- and homosalen-aluminum complexes. *Chem. - A Eur. J.* **2007**, *13*, 4433–4451, doi:10.1002/chem.200601308.

15. Casiraghi, G.; Casnati, G.; Puglia, G.; Sartori, G.; Terenghi, G. Selective reaction between phenols and formaldehyde. A novel route to salicylaldehydes. *J. Chem. Soc. Perkin Trans. 1* **1980**, 1862–1865, doi:10.1039/P19800001862.

16. Dixit, A.; Kumar, P.; Singh, S. Synthesis of chiral salalen ligands and their in-situ generated Cu-complexes for asymmetric Henry reaction. *Chirality* **2018**, *30*, 1257–1268, doi:10.1002/chir.23019.

17. Skarżewski, J.; Ostrycharz, E.; Siedlecka, R.; Zielińska-Blajet, M.; Pisarski, B. Substituted N-salicylidene β-aminoalcohols: Preparation and use as chiral ligands in enantioselective sulfoxidation and conjugate addition. *J. Chem. Res. - Part S* **2001**, 263–264, doi:10.3184/030823401103169847.

18. Diciccio, A.M.; Longo, J.M.; Rodríguez-Calero, G.G.; Coates, G.W. Development of highly active and regioselective catalysts for the copolymerization of epoxides with cyclic anhydrides: An unanticipated effect of electronic variation. *J. Am. Chem. Soc.* **2016**, *138*, 7107–7113, doi:10.1021/jacs.6b03113.

19. Grimster, N.P.; Connelly, S.; Baranczak, A.; Dong, J.; Krasnova, L.B.; Sharpless, K.B.; Powers, E.T.; Wilson, I.A.; Kelly, J.W. Aromatic sulfonyl fluorides covalently kinetically stabilize transthyretin to prevent amyloidogenesis while affording a fluorescent conjugate. *J. Am. Chem. Soc.* **2013**, *135*, 5656–5668, doi:10.1021/ja311729d.

20. Yale, H.L.; Losee, K.; Martins, J.; Holsing, M.; Perry, F.M.; Bernstein, J. Chemotherapy of Experimental Tuberculosis. VIII. The Synthesis of Acid Hydrazides, their Derivatives and Related Compounds. *J. Am. Chem. Soc.* **1953**, *75*, 1933–1942, doi:10.1021/ja01104a046.

21. Zhang, F.-J.; Vojkovsky, T.; Huang, P.; Liang, C.; Do, A.H.; Koening, M.; Cui, J. Triazolotrazine compounds and uses thereof 2005, WO 2005/010005 A1.

22. Rodrigues, D.A.; Guerra, F.S.; Sagrillo, F.S.; de Sena M. Pinheiro, P.; Alves, M.A.; Thota, S.; Chaves, L.S.; Sant'Anna, C.M.R.; Fernandes, P.D.; Fraga, C.A.M. Design, Synthesis, and Pharmacological Evaluation of First-in-Class Multitarget N-Acylhydrazone Derivatives as Selective HDAC6/8 and PI3Kα Inhibitors. *ChemMedChem* **2020**, *15*, 539–551, doi:10.1002/cmdc.201900716.

23. Freitas, R.H.C.N.; Barbosa, J.M.C.; Bernardino, P.; Sueth-Santiago, V.; Wardell, S.M.S.V.; Wardell, J.L.; Decoté-Ricardo, D.; Melo, T.G.; da Silva, E.F.; Salomão, K.; et al. Synthesis and trypanocidal activity of novel pyridinyl-1,3,4-thiadiazole derivatives. *Biomed. Pharmacother.* **2020**, *127*, 110162, doi:10.1016/j.biopha.2020.110162.

24. Arjunan, V.; Rani, T.; Mythili, C. V.; Mohan, S. Synthesis, FTIR, FT-Raman, UV-visible, ab initio and DFT studies on benzohydrazide. *Spectrochim. Acta - Part A Mol. Biomol. Spectrosc.* **2011**, *79*, 486–496, doi:10.1016/j.saa.2011.03.018.

25. Pereira, T.M.; Vitório, F.; Amaral, R.C.; Zanoni, K.P.S.; Murakami Iha, N.Y.; Kümmerle, A.E. Microwave-assisted synthesis and photophysical studies of novel fluorescent N-acylhydrazone and semicarbazone-7-OH-coumarin dyes. *New J. Chem.* **2016**, *40*, 8846–8854, doi:10.1039/c6nj01532h.

26. Popiołek, Ł.; Biernasiuk, A. Hydrazide-hydrazones of 3-methoxybenzoic acid and 4-tert-butylbenzoic acid with promising antibacterial activity against Bacillus spp. *J. Enzyme Inhib. Med. Chem.* **2016**, *31*, 62–69, doi:10.3109/14756366.2016.1170012.

27. Karabanovich, G.; Zemanová, J.; Smutný, T.; Székely, R.; Šarkan, M.; Centárová, I.; Vocat, A.; Pávková, I.; Čonka, P.; Němeček, J.; et al. Development of 3,5-dinitrobenzylsulfanyl-1,3,4-oxadiazoles and thiadiazoles as selective antitubercular agents active against replicating and nonreplicating Mycobacterium tuberculosis. *J. Med. Chem.* **2016**, *59*, 2362–2380, doi:10.1021/acs.jmedchem.5b00608.

28. Ghareb, N.; Elshihawy, H.A.; Abdel-Daim, M.M.; Helal, M.A. Novel pyrazoles and pyrazolo[1,2-a]pyridazines as selective COX-2 inhibitors; Ultrasound-assisted synthesis, biological evaluation, and DFT calculations. *Bioorganic Med. Chem. Lett.* **2017**, *27*, 2377–2383, doi:10.1016/j.bmcl.2017.04.020.

29. Ameryckx, A.; Thabault, L.; Pochet, L.; Leimanis, S.; Poupaert, J.H.; Wouters, J.; Joris, B.; Van Bambeke, F.; Frédérick, R. 1-(2-Hydroxybenzoyl)-thiosemicarbazides are promising antimicrobial agents targeting D-alanine-D-alanine ligase in bacterio. *Eur. J. Med. Chem.* **2018**, *159*, 324–338, doi:10.1016/j.ejmech.2018.09.067.

30. White, E.H.; Bursey, M.M.; Roswell, D.F.; Hill, J.H.M. Chemiluminescence of some monoacylhydrazides. *J. Org. Chem.* **1967**, *32*, 1198–1202, doi:10.1021/jo01279a077.

31. Aixi, H.; Chen, A.; Ye, J.; Liu, A.; Xu, L. Aromatic acyl hydrazone derivative and application thereof as NA inhibitor 2021, CN112079744 (B).

32. Nisa, M.U.; Munawar, M.A.; Chattha, F.A.; Kousar, S.; Munir, J.; Ismail, T.; Ashraf, M.; Khan, M.A. Synthesis of novel triazoles and a tetrazole of escitalopram as cholinesterase inhibitors. *Bioorganic Med. Chem.* **2015**, *23*, 6014–6024, doi:10.1016/j.bmc.2015.06.051.
